# Supplementary material for: “On-water” photosensitization enables redox neutral acylation and alkylation of quinones
Source: Nat Commun. 2026 Feb 16;17:1813. doi: 10.1038/s41467-026-69343-x (PMC12917259; doi:10.1038/s41467-026-69343-x)
Supplement: Supplementary file 1 — Supplementary Information [file 41467_2026_69343_MOESM1_ESM.pdf]

<u>**Supplementary Information**</u>

"*On-Water*" Photosensitization Enables Redox Neutral Acylation and Alkylation of Quinones

Tanumoy Mandal[‡1], Rohan Sharma[‡1], Enrique Mendez-Vega[1], Julia Rehbein[1], and Burkhard König[1,*]

[1]Fakultät für Chemie und Pharmazie, Universität Regensburg, 93040 Regensburg, Germany

The correspondence may be addressed to: burkhard.koenig@ur.de (B.K)

[‡] These authors contributed equally to this work.

Primary research data availability link: https://radar4chem.radar-service.eu/radar/en/dataset/z1v8szzsfn45kdw6?token=zJlsXqLQVoKLSFHyxGrC

Table of Contents

1. General information S2
2. Photographs and details of photochemical set-ups S3
3. Optimization of reaction conditions S3
4. General procedures for the photochemical reactions S7
5. Control experiments and mechanistic studies S8
6. Spectroscopic investigations (UV-Vis, Fluorescence and NMR) S12
7. Synthesis of starting materials S18
8. Characterization data of the final products S19
9. Computational studies S73
10. NMR spectra of the final products S79
11. References S232

1. General information

Reagents and solvents:

Commercially available chemicals were purchased at the highest commercial quality and used without further purification unless noted otherwise. Eosin Y (Tetrabromofluorescein, 95%; CAS No: 15086-94-9) was purchased from TCI. All reactions were carried out in a mixture of deionized water and acetone (reagent grade). In other cases (where required), the dry organic solvents were purchased from Thermo Fischer Scientific/Sigma Aldrich and stored over 3 Å molecular sieves inside sealed glass bottle.

Gas chromatography:

Gas chromatography with a flame-ionization detector (GC-FID) and gas chromatography coupled to low-resolution mass spectrometry (GC-MS) were performed using a capillary column (length: 30 m; diam.: 0.25 mm; film: 0.25 μM) using He as a carrier gas. GC-MS was performed on a 5975 MSD single quadrupole detector. Standard heating procedure: The initial temperature was set to 40 °C and was held for 3 minutes. Then, the temperature was increased to 280 °C at a rate of 15 °C/min and was held for 5 minutes. Lastly, the temperature was increased to 300 °C at a rate of 25 °C/min.

TLC:

Thin-layer chromatography (TLC) was performed on silica gel coated alumina plates (Macherey-Nagel, TLC sheets ALUGRAM Xtra SIL G UV254). Detection of the spots was accomplished utilizing UV light (254 nm).

UV-Vis Absorption Spectroscopy:

Absorption spectra were measured at room temperature using either an Agilent Cary 60 UV-Vis spectrophotometer or an Agilent 8453 UV-Vis spectrometer with a 10 mm Hellma quartz fluorescence cuvette or a 10 mm Starna Spectrosil Far UV Quartz cuvette.

Fluorescence Spectroscopy:

Fluorescence absorption and emission measurements were conducted using a HORIBA Fluoromax-4 spectrofluorometer or a Horiba Fluorolog-3 spectrofluorometer with FluorEssence v3.9 software. For time-resolved emission spectra, a home-built TCSPC-setup was used. The excitation source was a Horiba NanoLED-370 centered around 367 nm and the emission was recorded at 410 nm. Measurements were performed in a 10 mm Hellma quartz fluorescence cuvette or a 10 mm Starna Spectrosil Far UV Quartz cuvette.

NMR:

The NMR spectra were recorded at room temperature using a Bruker Avance 400 (400 MHz for ^{1}H, 101 MHz for ^{13}C, 376 MHz for ^{19}F) NMR spectrometer. All chemical shifts are reported in δ-scale as parts per million [ppm] (multiplicity, coupling constant J, number of protons) relative to the solvent residual peaks as the internal standard. Coupling constants J are given in Hertz [Hz]. Abbreviations used for signal

multiplicity: ^{1}H-NMR: br = broad, s = singlet, d = doublet, t = triplet, q = quartet, dd = doublet of doublets, dt = doublet of triplets, and m = multiplet.

HRMS:

High-resolution mass spectra (HRMS) were obtained from the central analytic mass spectrometry facilities of the Faculty of Chemistry and Pharmacy of the University of Regensburg. The measurements were carried out on either a JEOL AccuTOF GCX or Agilent Q-TOF 6540 UHD.

2. Photographs and details of the photochemical reaction set-ups

The photochemical reactions were performed using 455 (±15) nm LEDs (OSRAM Oslon SSL 80 LDCQ7P-1U3U (blue, λ_{max} = 455 (±15) nm, I_{max} = 1000 mA, 1.12 W) were used.

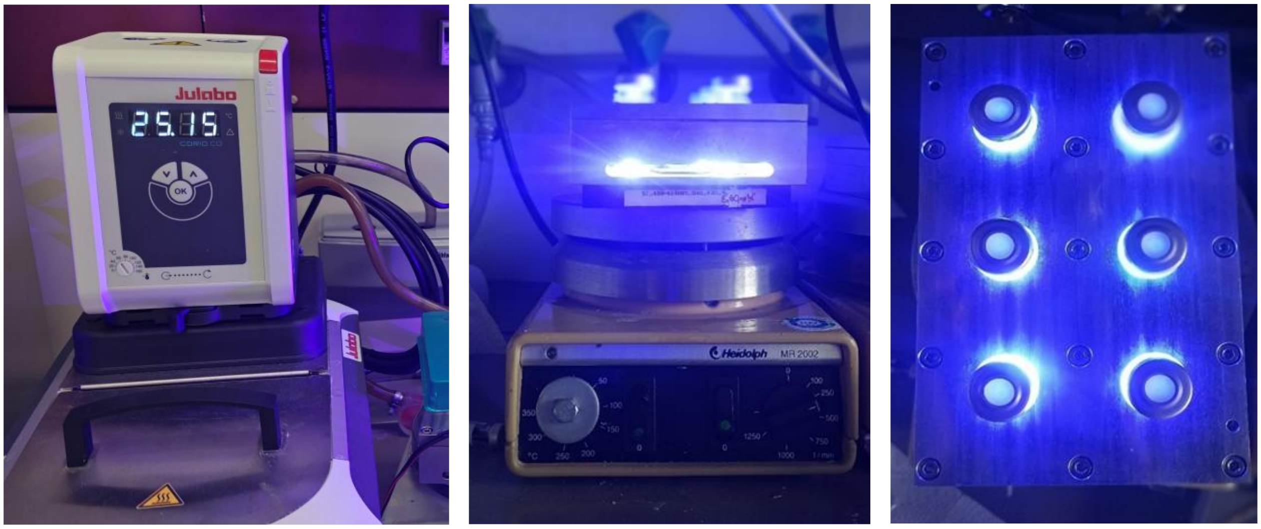

Fig. S1. Side and top view of the photochemical reaction setup. The reaction vials (5 mL crimp cap vials) were illuminated from the bottom side with blue LEDs (λ = 455 (± 15) nm). The reaction temperature was maintained at 25.0 °C by a custom-made thermostated aluminium cooling block.

3. Optimization of the reaction conditions and control reactions:

Table S1: Screening of the light souces and time of irradiation:[a,b]

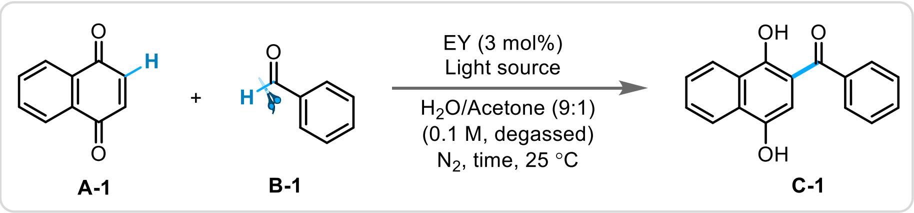

A 5 mL crimp top vial was charged with 1,4-naphthaquinone (**A-1**, 48 mg, 0.30 mmol, 1.0 equiv), benzaldehyde (**B-2**, 95 mg, 0.90 mmol, 3.0 equiv), and Eosin Y (6 mg, 0.009 mmol, 0.03 equiv). Then the vial was crimped. The vial was degassed and refilled with nitrogen using the Schlenk-line technique (three

times). A degassed (15 min) mixture of water/acetone (9:1, 3 mL) was added to the reaction vial and stirred at room temperature for 24 h under the irradiation of a single green LED (λ_{max} = 525 ± 15 nm). Upon completion, the reation mixture was diluted with EtOAc (20 mL), washed with saturated Na_2CO_3 solution (15 mL, two times) followed by brine (15 mL, one time) and extracted with EtOAc. The combined organic layer was dried over Na_2SO_4, concentrated under rotaevaporator and the residue was purified by silica gel (100-200 mesh) column chromatography using the mixture of PE/EtOAc as eluent to deliver pure **C-1** to check the isolated yield.

Entry	Light sources/Irradiation time	Yield
1.	525 nm / 24 h	80%
2.	525 nm / 12 h	63%
3.	525 nm/ 18 h	76%
4.	525 nm/ 36 h	81%
5.	470 nm / 24 h	85%
**6.**	**455 nm / 24 h**	**92%**
7.	390 nm / 24 h	64%

Reaction conditions: [a]**A-1** (0.3 mmol), **B-1** (0.9 mmol), EY (0.009 mmol), water/acetone (9:1) solvent mixture 3 mL (degassed), light source, N_2, 25 °C; [b]isolated yield.

Table S2: Screening of reaction solvents:[a,b]

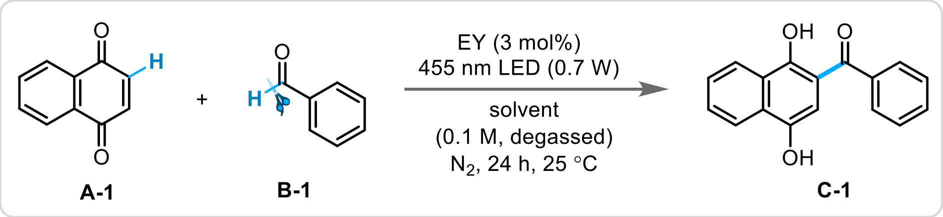

A 5 mL crimp top vial was charged with 1,4-naphthaquinone (**A-1**, 48 mg, 0.30 mmol, 1.0 equiv), benzaldehyde (**B-2**, 95 mg, 0.90 mmol, 3.0 equiv), and Eosin Y (6 mg, 0.009 mmol, 0.03 equiv). Then the vial was crimped. The vial was degassed and refilled with nitrogen using the Schlenk-line technique (three times). Degassed (15 min) dry solvent or a degassed mixture of water/dry solvent (9:1, 3 mL) was added to the reaction vial and stirred at room temperature for 24 h under the irradiation of a single blue LED (λ_{max} = 455 ± 15 nm). Upon completion, the reation mixture was diluted with EtOAc (20 mL), washed with saturated Na_2CO_3 solution (15 mL, two times) followed by brine (15 mL, one time) and extracted with EtOAc. The combined organic layer was dried over Na_2SO_4, concentrated under rotaevaporator and the residue was purified by silica gel (100-200 mesh) column chromatography using the mixture of PE/EtOAc as eluent to deliver pure **C-1** to check the isolated yield.

Entry	Solvent used (3 mL)	Yield
1.	dry acetone	23%
**2.**	**water/acetone (9:1)**	**92**
3.	dry MeCN	20%
4.	water/MeCN (9:1)	80%
5.	dry DMA	14%
6.	water/DMA (9:1)	59%
7.	dry DMSO	18%
8.	water/DMSO (9:1)	70%
9.	dry THF	15%
10.	water/THF (9:1)	65%
11.	water/EtOAc (9:1)	48%
12.	water/benzene (9:1)	52%
13.	water/dimethyl carbonate (9:1)	65%
14.	water/*tert*-butanol (9:1)	47%
15.	water/DCE (9:1)	<10%
16.	MeOH/acetone (9:1)	86%
17.	water/acetone (4:1)	67%
18.	water/acetone (1:1)	53%
19.	water/acetone (19:1)	64%
20.	water	68%

Reaction conditions: [a]**A-1** (0.3 mmol), **B-1** (0.9 mmol), EY (0.009 mmol), degassed dry solvent or degassed solvent mixture 3 mL, blue LED (455 nm, 0.7 W), N_2, 24 h, 25 ºC; [b]isolated yield.

Table S3: Screening of reaction photocatalysts:[a,b]

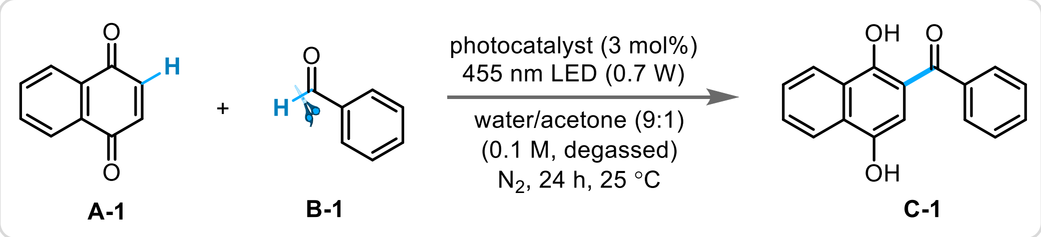

A 5 mL crimp top vial was charged with 1,4-naphthaquinone (**A-1**, 48 mg, 0.30 mmol, 1.0 equiv), benzaldehyde (**B-2**, 95 mg, 0.90 mmol, 3.0 equiv), and photocatalyst (0.009 mmol, 0.03 equiv). Then the vial was crimped. The vial was degassed and refilled with nitrogen using the Schlenk-line technique (three times). A degassed (15 min) mixture of water/acetone (9:1, 3 mL) was added to the reaction vial and stirred at room temperature for 24 h under the irradiation of a single blue LED (λ_{max} = 455 ± 15 nm). Upon

completion, the reation mixture was diluted with EtOAc (20 mL), washed with saturated Na_2CO_3 solution (15 mL, two times) followed by brine (15 mL, one time) and extracted with EtOAc. The combined organic layer was dried over Na_2SO_4, concentrated under rotaevaporator and the residue was purified by silica gel (100-200 mesh) column chromatography using the mixture of PE/EtOAc as eluent to deliver pure **C-1** to check the isolated yield.

Entry	Photocatalyst (3 mol%)	Yield
**1.**	**Eosin Y**	**92%**
2.	Rose Bengal	70%
3.	Fluorescein	76%
4.	Rhodamine 6G	55%
5.	Nile RED	34%
6.	Methylene blue	40%
7.	Eosin B	68%
8.	Na_2Eosin Y	81%
9.	Rhodamine B	60%
10.	Acridine Orange	57%

Reaction conditions: [a]**A-1** (0.3 mmol), **B-1** (0.9 mmol), photocatalyst (0.009 mmol), water/acetone (9:1) solvent mixture 3 mL (degassed), light source, N_2, 24 h, 25 °C; [b]isolated yield.

Table S4: Screening of stoichiometry of reagents and concentration:[a,b]

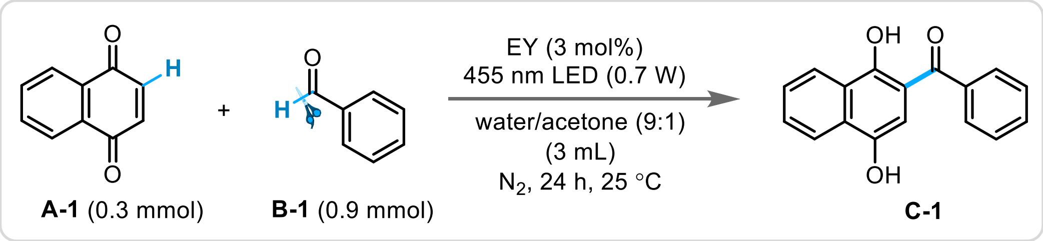

A 5 mL crimp top vial was charged with 1,4-naphthaquinone (**A-1**, 48 mg, 0.30 mmol, 1.0 equiv), benzaldehyde (**B-2**, 95 mg, 0.90 mmol, 3.0 equiv), and Eosin Y (6 mg, 0.009 mmol, 0.03 equiv). Then the vial was crimped. The vial was degassed and refilled with nitrogen using the Schlenk-line technique (three times). A degassed (15 min) mixture of water/acetone (9:1, 3 mL) was added to the reaction vial and stirred at room temperature for 24 h under the irradiation of a single blue LED (λ_{max} = 455 ± 15 nm). Upon completion, the reation mixture was diluted with EtOAc (20 mL), washed with saturated Na_2CO_3 solution (15 mL, two times) followed by brine (15 mL, one time) and extracted with EtOAc. The combined organic layer was dried over Na_2SO_4, concentrated under rotaevaporator and the residue was purified by silica gel

(100-200 mesh) column chromatography using the mixture of PE/EtOAc as eluent to deliver pure **C-1** to check the isolated yield.

Entry	Deviation from mentioned condition	Yield
1.	None	92%
2.	2 equiv of **B-1** was used	92%
**3.**	**1.5 equiv of B-1 was used**	**92%**
4.	1.0 equiv of **B-1** was used	83%
5.	5.0 equiv of **B-1** was used	66%
6.	10 mol% Eosin Y was used	82%
7.	5 mol% Eosin Y was used	87%
8.	1 mol% Eosin Y was used	90%
9.	5 mL solvent mixture was added	71%
10.	2 mL solvent mixture was added	84%

Reaction conditions: [a]**A-1** (0.3 mmol), **B-1**, Eosin Y (0.009 mmol), water/acetone (9:1) solvent mixture (degassed), light source, N_2, 24 h, 25 ºC; [b]isolated yield.

4. General Procedure for photochemical reactions:

(i) General Procedure for acylation (GP1):

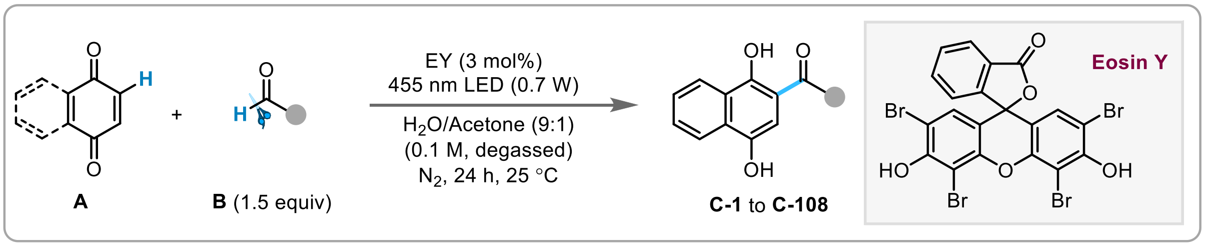

A 5 mL crimp top vial was charged with 1,4-quinone derivative (**A**, 0.30 mmol, 1.0 equiv), aldehyde (**B**, 0.45 mmol, 1.5 equiv), and Eosin Y (6 mg, 0.009 mmol, 0.03 equiv). Then the vial was crimped. The vial was degassed and refilled with nitrogen using the Schlenk-line technique (three times). A degassed (15 min) mixture of water/acetone (9:1, 3 mL) was added to the reaction vial and stirred at room temperature for 24 h under the irradiation of a single blue LED (λ_{max} = 455 ± 15 nm). Upon completion, the reation mixture was diluted with EtOAc (20 mL), washed with saturated Na_2CO_3 solution (15 mL, two times) followed by brine (15 mL, one time) and extracted with EtOAc. The combined organic layer was dried over Na_2SO_4, concentrated under rotaevaporator and the residue was purified by silica gel (100-200 mesh) column chromatography using the mixture of PE/EtOAc as eluent to deliver pure product **C-1** to **C-108**.

(ii) General proeduce for gram-scale synthesis (GP-2):

A 100 mL Schlenk flask was charged with 1,4-naphthaquinone (**A-1**, 1.11 g, 7.0 mmol, 1.0 equiv), aldehyde (**B**, 10.5 mmol, 1.5 equiv), and Eosin Y (142 mg, 0.21 mmol, 0.03 equiv). The flask was closed with septum. The flask was then degassed and refilled with nitrogen using the Schlenk-line technique (three times). A degassed (15 min) mixture of water/acetone (9:1, 70 mL) was added to the reaction vial and stirred at room temperature for 24 h under the irradiation with 455 nm LEDs (two LED plates were put at the two side of the flask). Upon completion, the reation mixture was diluted with EtOAc (150 mL), washed with saturated Na_2CO_3 solution (150 mL, two times) followed by brine (100 mL, one time) and extracted with EtOAc. The combined organic layer was dried over Na_2SO_4, concentrated under rotaevaporator and the residue was purified by silica gel (100-200 mesh) column chromatography using the mixture of PE/EtOAc as eluent to deliver pure product **C-1**, **C-41**, and **C-137**.

(iii) General Procedure for alkylation (GP3):

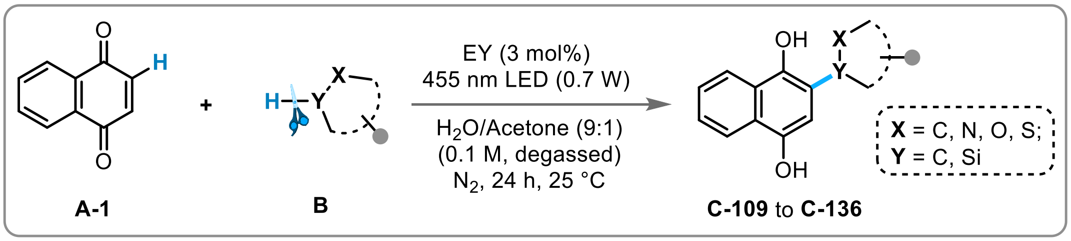

A 5 mL crimp top vial was charged with 1,4-quinone derivative (**A**, 0.30 mmol, 1.0 equiv), alkyl coupling partner (**B**, 0.9 mmol, 3.0 equiv), and Eosin Y (6 mg, 0.009 mmol, 0.03 equiv). Then the vial was crimped. The vial was degassed and refilled with nitrogen using the Schlenk-line technique (three times). A degassed (15 min) mixture of water/acetone (9:1, 3 mL) was added to the reaction vial and stirred at room temperature for 24 h under the irradiation of a single blue LED (λ_{max} = 455 ± 15 nm). Upon completion, the reation mixture was diluted with EtOAc (20 mL), washed with saturated Na_2CO_3 solution (15 mL, two times) followed by brine (15 mL, one time) and extracted with EtOAc. The combined organic layer was dried over Na_2SO_4, concentrated under rotaevaporator and the residue was purified by silica gel (100-200 mesh) column chromatography using the mixture of PE/EtOAc as eluent to deliver pure product **C-109** to **C-136**.

5. Control Experiments and mechanistic studies:

5.1. Importance of light sources, photocatalyst and innert atmosphere:[a,b]

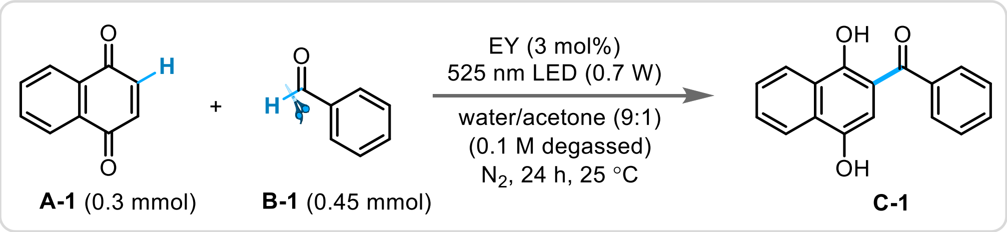

A 5 mL crimp top vial was charged with 1,4-naphthaquinone (**A-1**, 48 mg, 0.30 mmol, 1.0 equiv), benzaldehyde (**B-2**, 48 mg, 0.45 mmol, 1.5 equiv), and Eosin Y (6 mg, 0.009 mmol, 0.03 equiv). Then the

vial was crimped. The vial was degassed and refilled with nitrogen using the Schlenk-line technique (three times). Degassed (15 min) dry solvent or a degassed mixture of water/dry solvent (9:1, 3 mL) was added to the reaction vial and stirred at room temperature for 24 h under the irradiation of a single blue LED (λ_{max} = 525 ± 15 nm). Upon completion, the reation mixture was diluted with EtOAc (20 mL), washed with saturated Na_2CO_3 solution (15 mL, two times) followed by brine (15 mL, one time) and extracted with EtOAc. The combined organic layer was dried over Na_2SO_4, concentrated under rotaevaporator and the residue was purified by silica gel (100-200 mesh) column chromatography using the mixture of PE/EtOAc as eluent to deliver pure **C-1** to check the isolated yield.

Entry	Deviation from mentioned condition	Yield
1.	none	80%
2.	Under dark at 25 ºC	ND
3.	Under dark at 80 ºC	ND
4.	No photocatalyst was used	8%
5.	Under air	45%

Reaction conditions: [a]**A-1** (0.3 mmol), **B-1** (0.45 mmol), EY (0.009 mmol), water/acetone (9:1) solvent mixture 3 mL (degassed), green LED (525 nm, 0.7 W), N_2, 24 h, 25 ºC; bisolated yield; ND = not detected (in GC-MS).

5.2. Role of Eosin Y:

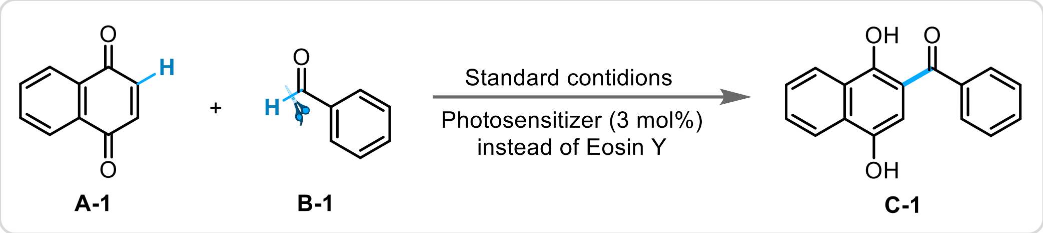

To confrim the role of Eosin Y in HAT or photosensitization, the standard reaction was carried out using common photosensitizers those are unable to participate in hydrogen atom transfer reactions.

A 5 mL crimp top vial was charged with 1,4-naphthaquinone (**A-1**, 48 mg, 0.30 mmol, 1.0 equiv), benzaldehyde (**B-2**, 48 mg, 0.45 mmol, 1.5 equiv), and photosensitizer (0.009 mmol, 0.03 equiv). Then the vial was crimped. The vial was degassed and refilled with nitrogen using the Schlenk-line technique (three times). Degassed (15 min) dry solvent or a degassed mixture of water/dry solvent (9:1, 3 mL) was added to the reaction vial and stirred at room temperature for 24 h under the irradiation of a single blue LED (λ_{max} = 455 ± 15 nm). Upon completion, the reation mixture was diluted with EtOAc (20 mL), washed with saturated Na_2CO_3 solution (15 mL, two times) followed by brine (15 mL, one time) and extracted with

EtOAc. The combined organic layer was dried over Na_2SO_4, concentrated under rotaevaporator and the residue was purified by silica gel (100-200 mesh) column chromatography using the mixture of PE/EtOAc as eluent to deliver pure **C-1** to check the isolated yield.

Entry	Photosensitizer (3 mol%)	E_T value	Isolated yield of C-1
1.	$[Ir(dF\text{-}CF_3\text{-}ppy)_2(dtbpy)]PF_6$	61.8 Kcal/mol	91%
2.	$[Ru(bpy)_3]Cl_2$	49.1 Kcal/mol	87%
3.	4-CzIPN	58.3	90%

5.3. Proof of photosensitization:

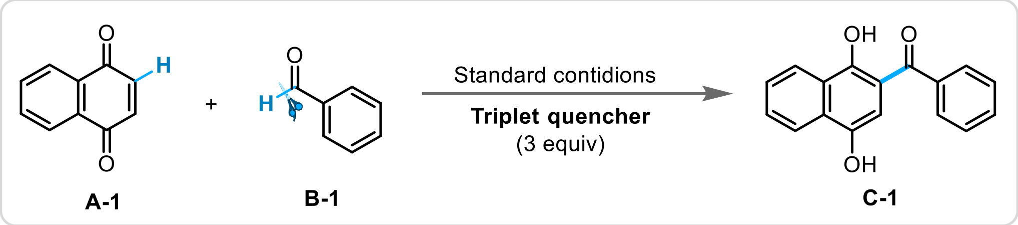

To confrim the event of photosensitization during this transformation, the standard reaction was carried out in presence of stoichiometric triplet quenchet to hinder the energy transfer step (to quinone).

A 5 mL crimp top vial was charged with 1,4-naphthaquinone (**A-1**, 48 mg, 0.30 mmol, 1.0 equiv), benzaldehyde (**B-2**, 48 mg, 0.45 mmol, 1.5 equiv), triplet quencher (3 equiv), and Eosin Y (6 mg, 0.009 mmol, 0.03 equiv). Then the vial was crimped. The vial was degassed and refilled with nitrogen using the Schlenk-line technique (three times). Degassed (15 min) dry solvent or a degassed mixture of water/dry solvent (9:1, 3 mL) was added to the reaction vial and stirred at room temperature for 24 h under the irradiation of a single blue LED (λ_{max} = 455 ± 15 nm). Upon completion, the reation mixture was diluted with EtOAc (20 mL), washed with saturated Na_2CO_3 solution (15 mL, two times) followed by brine (15 mL, one time) and extracted with EtOAc. The combined organic layer was dried over Na_2SO_4, concentrated under rotaevaporator and residue was purified by silica gel (100-200 mesh) column chromatography using the mixture of PE/EtOAc as eluent to deliver pure **C-1** to check the isolated yield.

Quencher used	Triplet energy	Yield of C-1
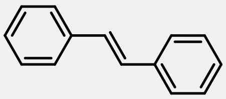	*trans*-Stilbene<br>E_T = 49.3 Kcal/mol	<10%
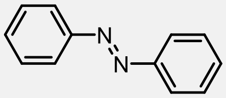	Azobenzene<br>E_T = 35.4 Kcal/mol	trace

5.4. Radical trapping experiments:

To check the involvement of radical intermediate(s) during the overall transformation, the standard reaction was carried out in presence of stoichiometric radical quencher.

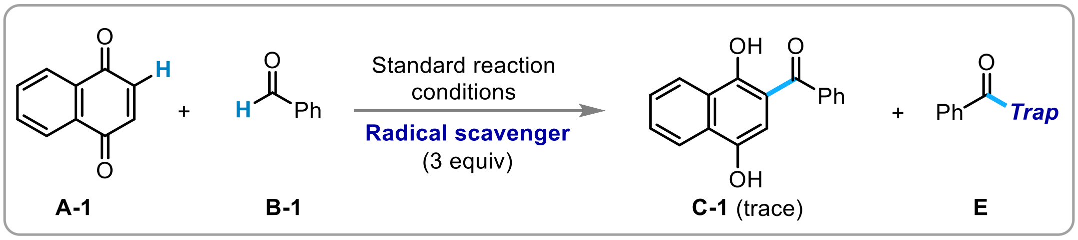

A 5 mL crimp top vial was charged with 1,4-naphthaquinone (**A-1**, 48 mg, 0.30 mmol, 1.0 equiv), benzaldehyde (**B-2**, 48 mg, 0.45 mmol, 1.5 equiv), radical scavenger (3 equiv), and Eosin Y (6 mg, 0.009 mmol, 0.03 equiv). Then the vial was crimped. The vial was degassed and refilled with nitrogen using the Schlenk-line technique (three times). Degassed (15 min) dry solvent or a degassed mixture of water/dry solvent (9:1, 3 mL) was added to the reaction vial and stirred at room temperature for 24 h under the irradiation of a single blue LED (λ_{max} = 455 ± 15 nm). Upon completion, the reation mixture was diluted with EtOAc (20 mL), washed with saturated Na_2CO_3 solution (15 mL, two times) followed by brine (15 mL, one time) and extracted with EtOAc. The combined organic layer was dried over Na_2SO_4, concentrated under rotaevaporator and the residue was purified by silica gel (100-200 mesh) column chromatography using the mixture of PE/EtOAc as eluent to deliver pure **C-1** to check the isolated yield. A fraction of the crude reaction mixture was also submitted for ESI-HRMS analysis to detect the radical intermediate **E**.

Scavenger	Trapped radical		
TEMPO	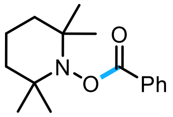	E-1	**Detected in ESI-HRMS**<br>Calcd. for $C_{17}H_{23}NO_2$ $[M+H]^+$: 262.1802; found: 262.1807
1,1-Diphenyl ethylene	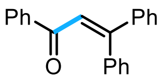	E-2	**Detected in EI-HRMS**<br>Calculated for $C_{21}H_{16}O$: 284.1201; found: 284.1212

5.5. Kinetic study:

To determine the rate-determining step of the overall transformation, two parallel reactions were run [using a mixture of D_2O/acetone-d_6 (9:1)] under standard reaction conditions using normal benzaldehyde (**B-1**) and benzaldehyde-d_6 (**B-1'**). After 12 hours, the crude reaction mixture was analyzed by ^1H NMR using CH_2Br_2 as the internal standard. 63% of **C-1** was formed, whereas **C-1'** was produced only in 24% at the same time, and a KIE of 2.63 was calculated. This experiment indicates that C–H bond activation may be the rate-determining step of the overall reaction.

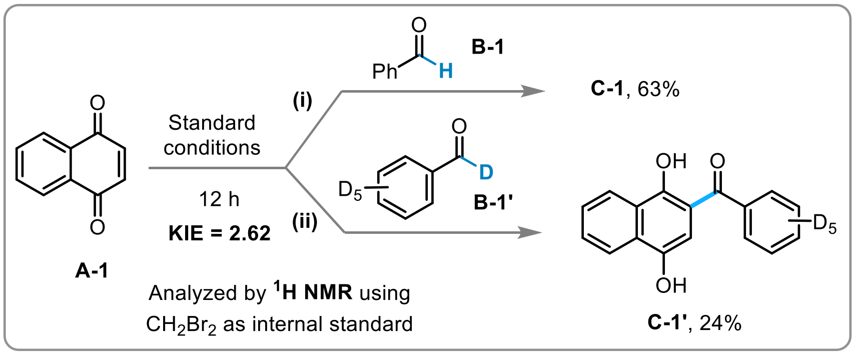

In addition, a kinetic profile of the reaction was monitored (with ^1H NMR) by measuring the amount of product formation (**C-1** and **C-1'**) with time. To do that the crude reaction mixture two aforementioned reactions were analyzed by ^1H NMR using CH_2Br_2 in the interval of every two hours.

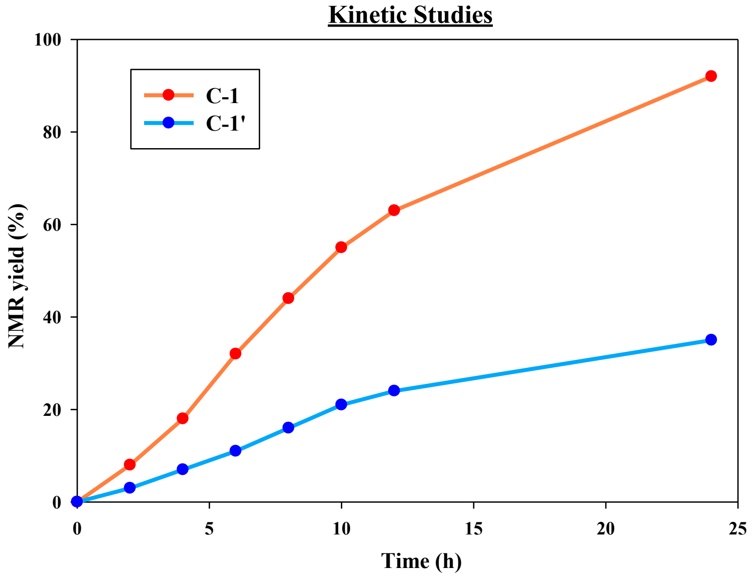

Fig. S2. Reaction profile diagram (Kinetic studies)

6. Spectroscopic findings:[1]

6.1. UV-Vis experiments:

The measurements were carried out by dissolving the 1,4-naphthaquinone (**A-1**) maintaining concentration 1 mM in different dry organic solvents (such as, MeCN, acetone, DMA, DMSO, THF) and a series of

solvent mixture containing water/organic solvent (9:1) and another solvent mixtiure containing MeOH/acetone (9:1). In case of other quinones derivatives (**A**), Eosin Y, and **C-1,** the componets were individually dissolved in dry acetone or dry MeCN and a solvent mixture containing water/acetone (9:1) or water/MeCN (9:1) by maintaining the concentration 1 mM.

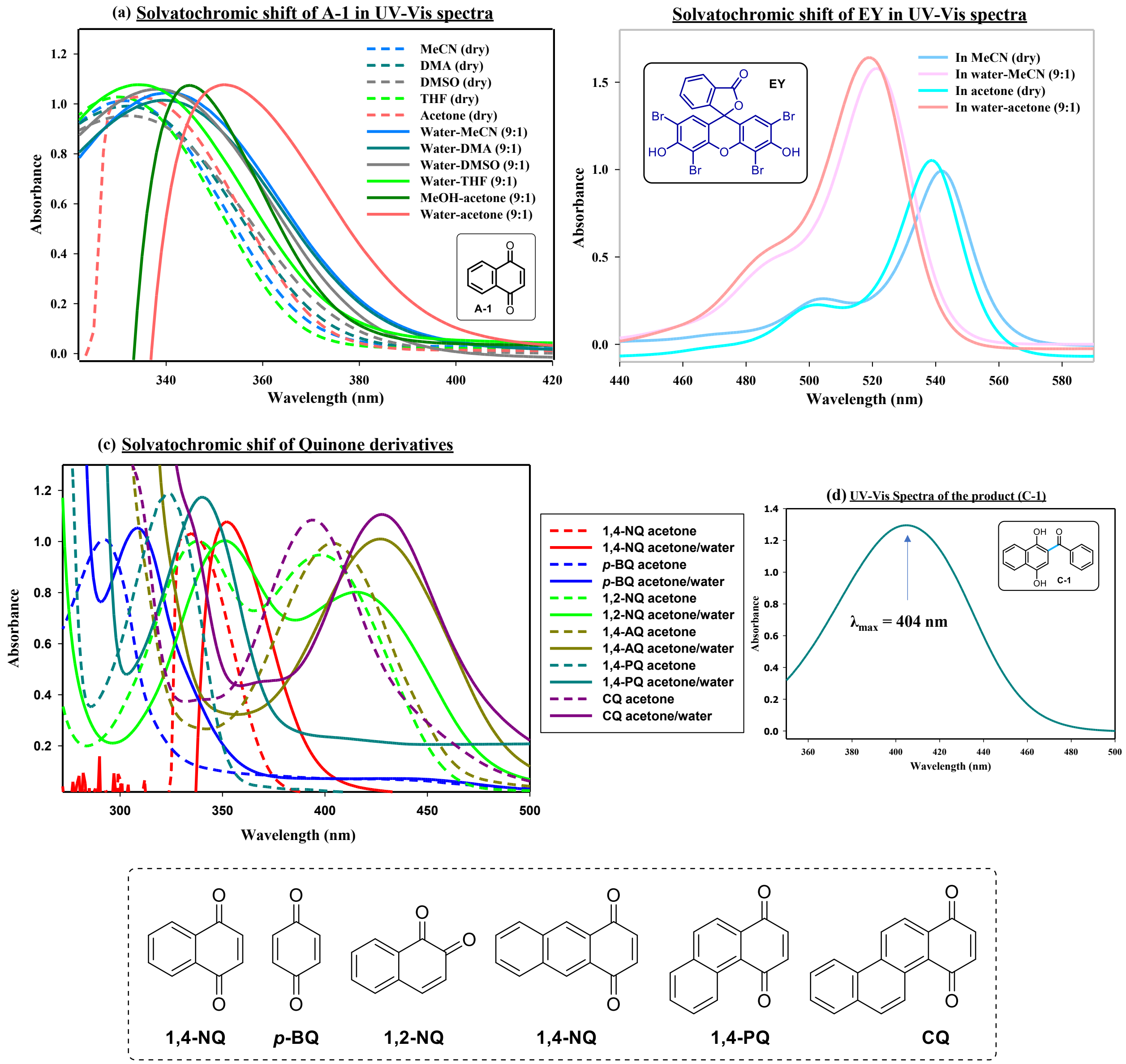

Fig. S3. UV-Vis studies: (a) Solvatochromic shift of **A-1** in different anhydrous organic solvent and water/organic solvent mixture (9:1); (b) solvatochromic shift of Eosin Y; (c) solvatochromic shif of other quinone derivatives in dry acetone and water/acetone mixture (9:1); (d) UV-Vis absorption spectra of **C-1** in acetone.

6.2. Fluorescence experiments:

6.2.1 Emission spectra and normalized merged abosorption emission spectra:

The emission spectra of **A-1, C-1** and Eosin Y were individually recorded in dry acetone and water/acetone (9:1) mixture using a 10.0 mm 3 mL quartz cuvette. To do that 1 mM solutions of each component were freshly prepared in dry acetone acetone and water/acetone (9:1) mixture. For **A-1**: an aliquot of 50 µL was taken insode the quartz cuvette and further diluted to 3 mL with dry acetone or water/acetone (9:1) mixture whereas, for Eosin Y: an aliquot of 20 µL was taken insode the quartz cuvette and further diluted to 3 mL with dry acetone or water/acetone (9:1) mixture before recording the emission spectra. For **C-1**: an aliquot of 30 µL was taken insode the quartz cuvette and further diluted to 3 mL with acetone before recording the emission spectra. The corresponding merged normalized absorption-emission spectra is also depicted.

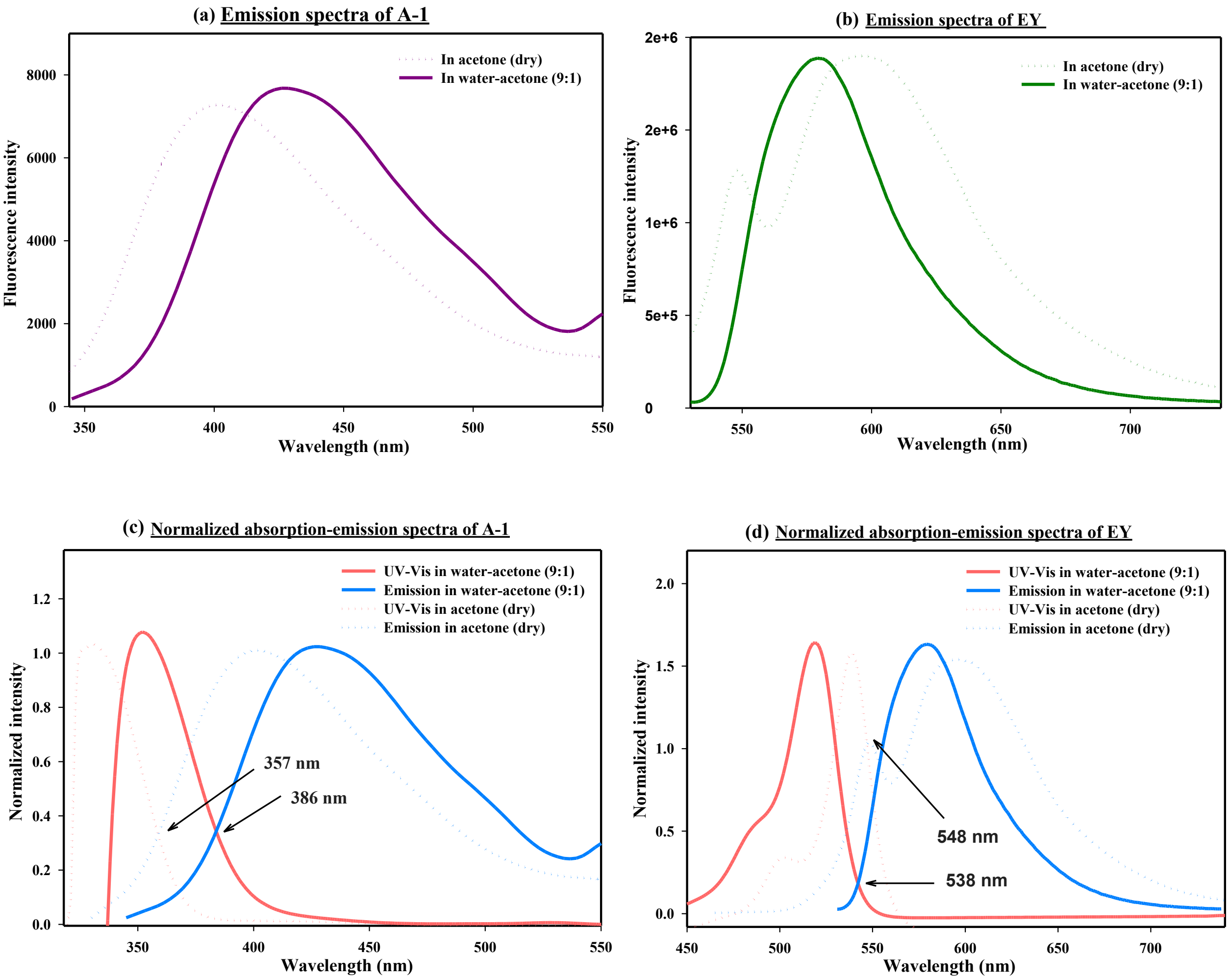

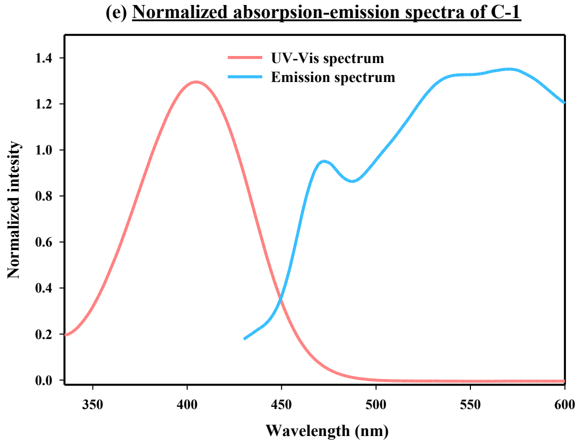

Fig. S4. Emission spectra: (a) emission spectra of **A-1** in dry acetone and water/acetone mixture (9:1); (b) emission spectra of Eosin Y in dry acetone and water/acetone mixture; (c) normalized merged absorption-emission spectra of **A-1** in dry acetone and water/acetone mixture (9:1); (d) normalized merged absorption-emission spectra of Eosin Y in dry acetone and water/acetone mixture (9:1); (d) normalized merged absorption-emission spectra of **C-1** in dry acetone and water/acetone mixture (9:1).

6.2.1 Emission spectra and normalized merged abosorption emission spectra:

6.2.2 Photoluminesces quenching study:

Photoluminescence quenching experiment of Eosin Y (PC) was performed by dissolving the Eosin Y individually in dry acetone and water/acetone mixture (9:1) at [1 µM] concentration and corresponding 1,4-naphthaquinone (**A-1**) and benzaldehyde **(B-1)** in individually in dry acetone and water/acetone mixture (9:1) at [0.1 M]. The photoluminescence quenching spectral data were collected by increasing amount of substates added in order to study the changing of the quencher concentration vs the I_o/I. The corresponding Stern-Volmer plot is depicted.

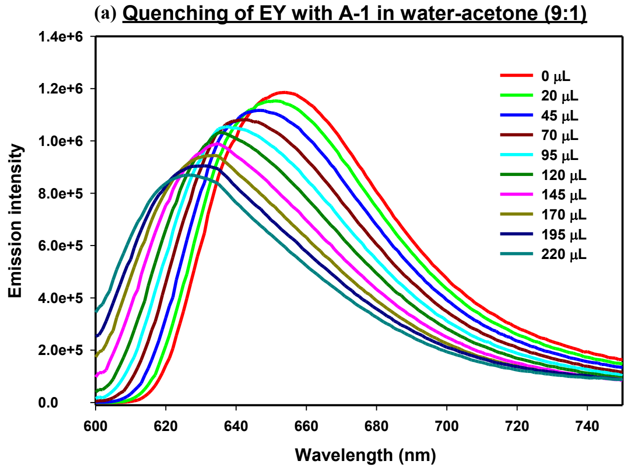

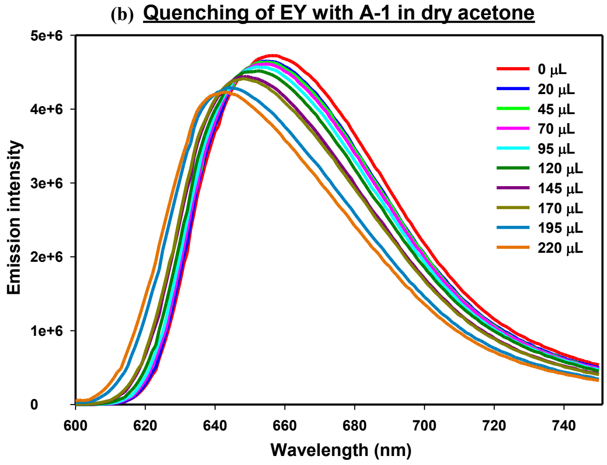

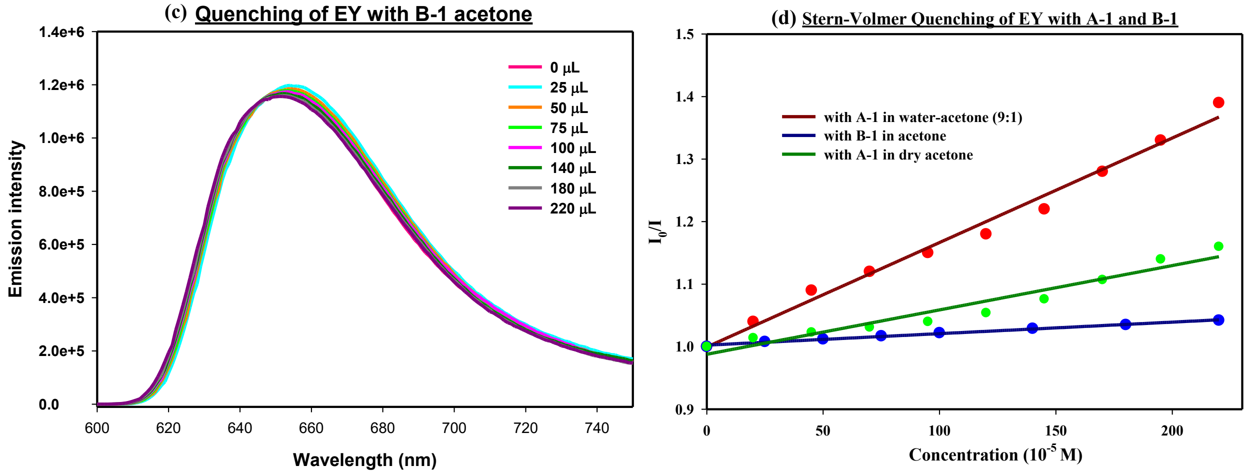

Fig. S5. Emission spectra: (a) photoluminescence quenching of EY against **A-1** in water/acetone mixture (9:1); (b) photoluminescence quenching of EY against **A-1** in dry acetone; (c) photoluminescence quenching of EY against **B-1** in acetone; (d) Stern-Volmer quenching plots.

6.3. NMR studies:

We performed 1H and 13C NMR studies using 1,4-naphthoquinone (A-1) individually in neat acetone-d6, 6:1 mixture of D2O/ acetone-d6, 5:2 mixture of H2O/ acetone-d6 and 5:2 mixture of MeOH/ acetone-d6, respectively to support the H-bonding interaction of quinone in water/acetone mixture. The following observation is now added to the revised manuscript and highlighted in yellow. It was observed that in the 1H NMR spectra, the alkene protons next to the carbonyl groups (Ha) exhibited a downfield shift from 7.05 ppm in neat acetone-d6 to 6.96 ppm in a 6:1 D_2O/acetone-d6 solvent mixture. Moreover, Ha shifted to approximately 7.00 ppm when the solvent system was changed to either 5:2 H_2O/ acetone-d6 or 5:2 MeOH/acetone-d6. A similar trend of downfield shift was observed in case of Hb as well and it exhibited the □ values of 8.05 ppm, 7.90 ppm, 7.95 ppm and 8.02 ppm, respectively in the solvent mixtures mentioned above. On the other hand, in the 13C NMR spectra, the carbonyl group of A-1 shifted from 185.7 ppm in neat acetone-d6 to 178.8, 181.2, and 183.9 ppm in 6:1 D_2O/ acetone-d6, 5:2 H_2O/ acetone-d6, and 5:2 MeOH/ acetone-d6, respectively. These downfield shifts of the olefinic and aromatic protons (ortho to the carbonyl group of A-1) and carbonyl carbons are consistent with hydrogen-bonding interactions involving the naphthoquinone moiety, which further supports our working hypothesis.

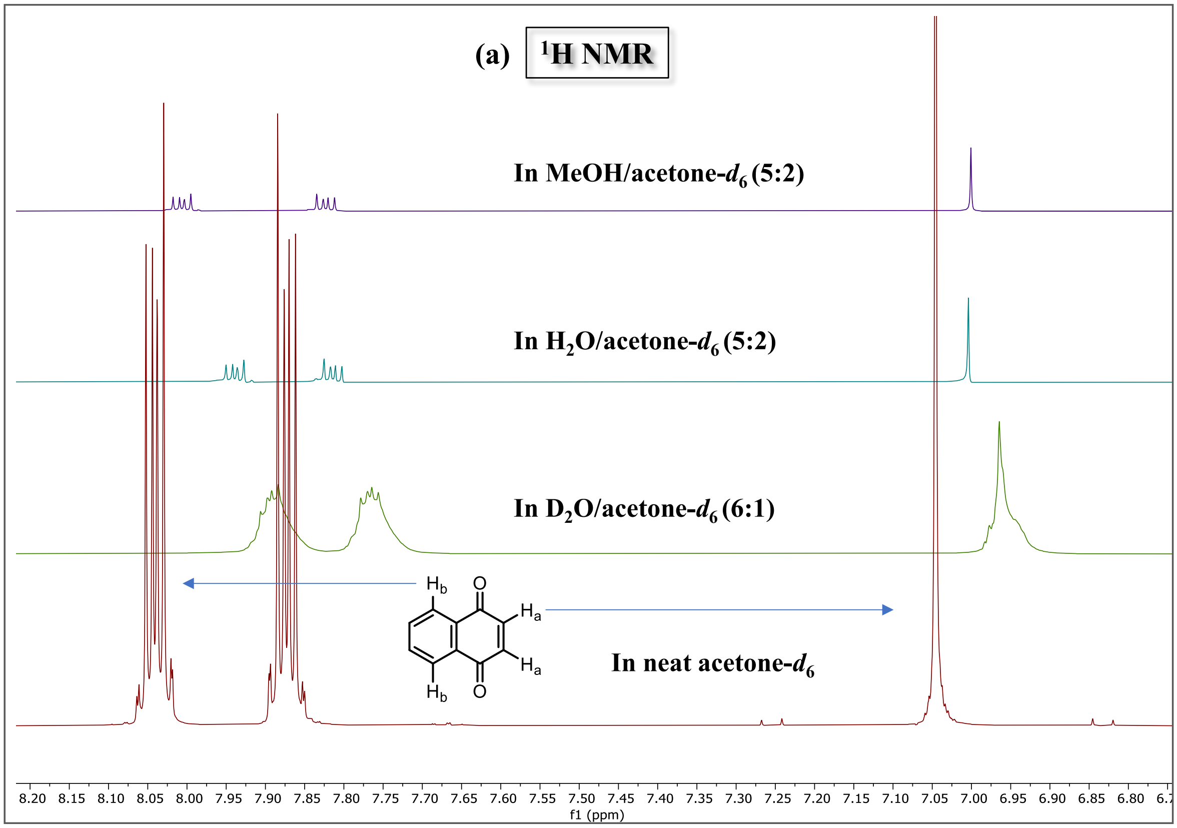

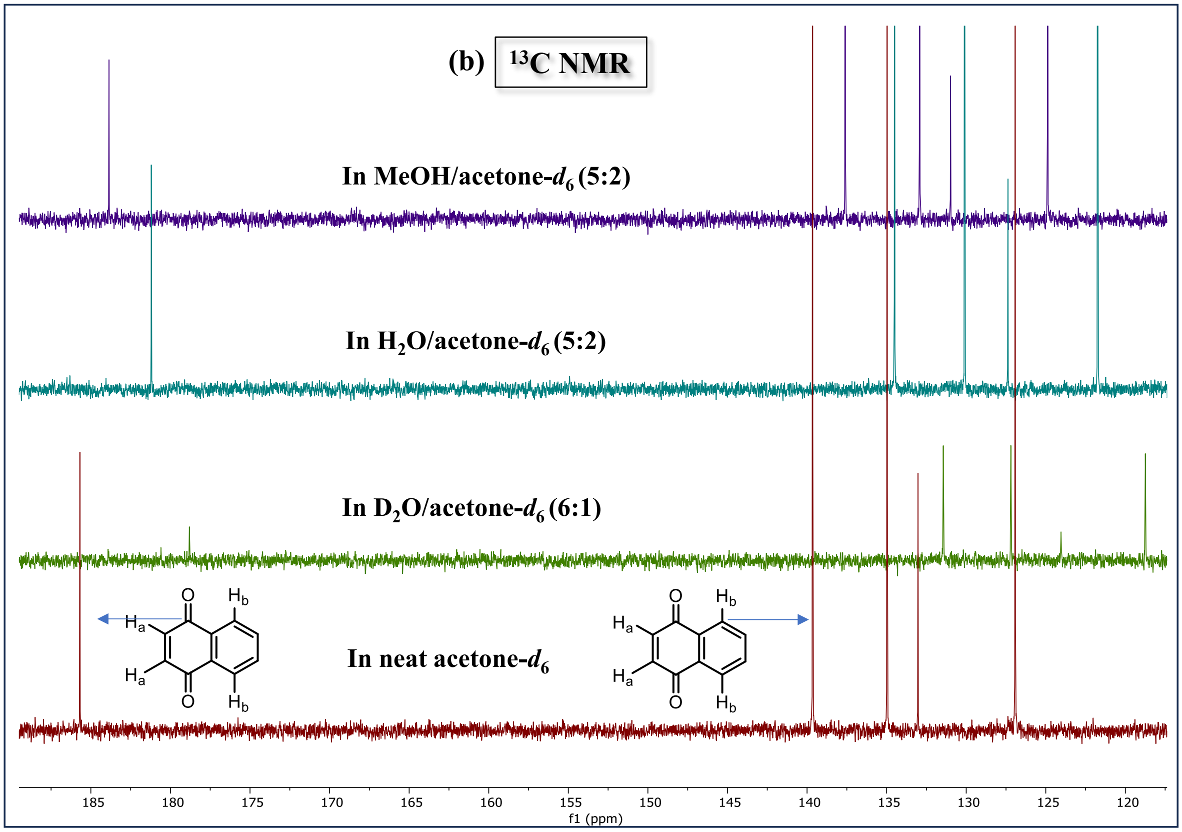

Fig. S6. NMR spectra: (a) ^{1}H NMR of **A-1** in different solvent mixture; (b) ^{13}C NMR of **A-1** in different solvent mixture.

7. Synthesis of starting materials:

7.1. General procedure of esterification reactions for the late-stage modification (GP-4):[2]

Following the literature report,

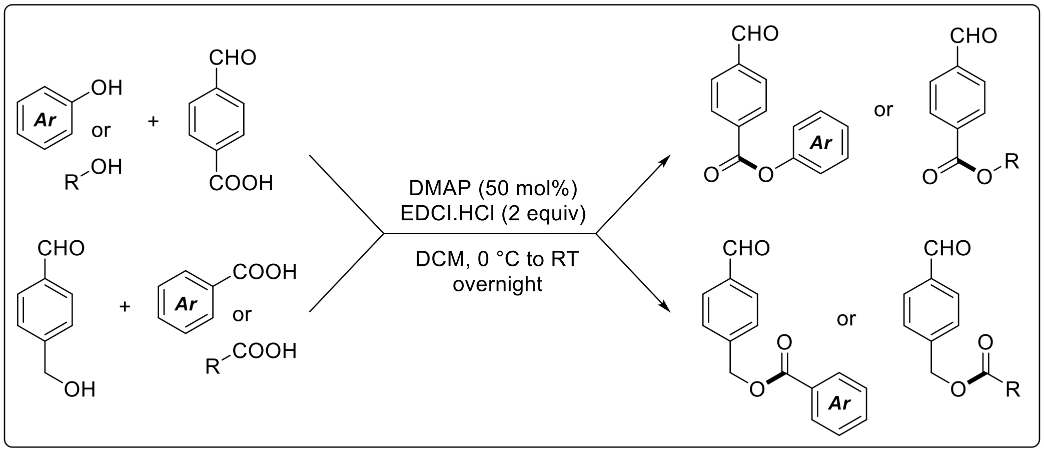

In a 100 mL round-bottom flask, to a stirred solution of corresponding carboxylic acid acid (5 mmol), and 4 dimethylamino)pyridine (2.5 mmol, 305 mg) in DCM (40 mL) at 0 °C was added 1-ethyl-3-(3 dimethylaminopropyl)carbodiimide hydrochloride (EDCI.HCl, 10 mmol, 1.92 g), and the mixture was allowed to stir at 0 °C on an ice bath for 30 mins. After that, the desired alcohol (5 mmol) was added to the reaction mixture and the ice bath was removed. The resulting reaction mixture was stirred at room temperature for overnight. After completion, water was added, the mixture was extracted with DCM, and the organic layer was successively washed with 1 M HCl and saturated $NaHCO_3$, and brine, and dried over anhydrous Na_2SO_4. The crude product was purified with silica gel column chromatography to deliver the desired ester in 65% to 90% isolated yield.

7.2. Synthetic procedure of etherification of juglone:[3]

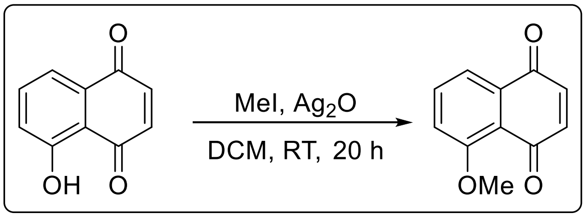

Following the literature report, to 5-hydroxy-1,4-naphthoquinone (400 mg, 2.3 mmol) and silver(I) oxide (400 mg, 1.85 mmol) in DCM (10 mL) was added iodomethane (0.3 mL, 4.6 mmol) and the mixture stirred at ambient temperature. After 20 h further iodomethane (0.12 mL, 0.1.85 mmol) and silver(I) oxide (400 mg, 1.85 mmol) were added and the reaction mixture stirred for a further 3 h. The reaction mixture was filtered through Celite, washed with DCM and concentrated in vacuo. The residue was purified by column

chromatography using EtOAc in PE (15%) as eluent to deliver 5-methoxy-1,4-naphthoquinone as orange solid (390 mg, 90%).

8. Characterization data of the final products:

(1,4-dihydroxynaphthalen-2-yl)(phenyl)methanone (C-1):[4]

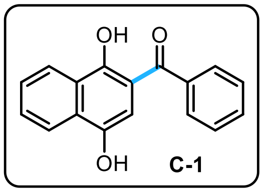

GP1 was followed using 1,4-naphthaquinone (0.3 mmol, 48 mg) and benzaldehyde (48 mg, 0.45 mmol). After 24 h, purification by column chromatography using 12% ethyl acetate in PE yielded **C-1** (73 mg, 92%) as a yellow solid.

^{1}H NMR (400 MHz, $CDCl_3$) δ 13.54 (s, 1H), 8.54 – 8.49 (m, 1H), 8.13 (dd, J = 8.2, 1.2 Hz, 1H), 7.72 – 7.64 (m, 3H), 7.60 (ddd, J = 8.2, 6.9, 1.3 Hz, 1H), 7.55 – 7.50 (m, 1H), 7.45 (dd, J = 8.1, 6.6 Hz, 2H), 6.85 (s, 1H), 5.43 (s, 1H).

^{13}C NMR{^{1}H} (101 MHz, $CDCl_3$) δ 201.0, 158.8, 142.8, 138.3, 131.7, 130.2, 129.6, 129.0, 128.4, 126.7, 126.2, 124.7, 121.9, 111.6, 108.1.

For gram-scale synthesis: **GP4** was followed using 1,4-naphthaquinone (7 mmol, 1.10 g) and benzaldehyde (1.13 g, 10.5 mmol). After 24 h, purification by column chromatography using 12% ethyl acetate in PE yielded **C-1** (1.43 g, 77%) as a yellow solid.

(1,4-dihydroxynaphthalen-2-yl)(4-octylphenyl)methanone (C-2):

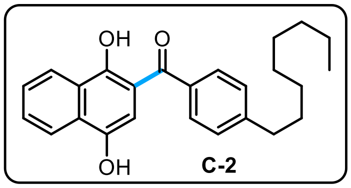

GP1 was followed using 1,4-naphthaquinone (0.3 mmol, 48 mg) and 4-octylbenzaldehyde (98 mg, 0.45 mmol). After 24 h, purification by column chromatography using 8% ethyl acetate in PE yielded **C-2** (105 mg, 93%) as a yellow solid.

^{1}H NMR (400 MHz, $CDCl_3$) δ 13.87 (s, 1H), 8.81 (ddd, J = 8.4, 1.3, 0.7 Hz, 1H), 8.45 – 8.41 (m, 1H), 7.99 (ddd, J = 8.3, 6.9, 1.3 Hz, 1H), 7.91 (dd, J = 7.8, 1.6 Hz, 3H), 7.57 (dd, J = 7.0, 1.3 Hz, 2H), 7.23 (s, 1H), 5.62 (s, 1H), 2.95 (dd, J = 8.8, 6.8 Hz, 2H), 1.93 (t, J = 7.5 Hz, 2H), 1.64 – 1.57 (m, 10H), 1.21 – 1.18 (m, 3H).

^{13}C NMR{^{1}H} (101 MHz, $CDCl_3$) δ 200.8, 158.6, 147.5, 142.7, 138.8, 135.7, 134.1, 130.0, 129.3, 128.5, 126.6, 124.7, 121.8, 111.7, 108.3, 36.1, 32.0, 31.3, 29.6, 29.4, 29.4, 22.8, 14.2.

ESI-HRMS: m/z calculated for $C_{25}H_{29}O_3$ $[M+H]^+$ calcd. 377.2111, found 377.2117.

[1,1'-biphenyl]-4-yl(1,4-dihydroxynaphthalen-2-yl)methanone (C-3):

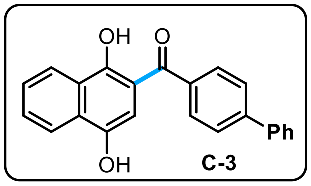

GP1 was followed using 1,4-naphthaquinone (0.3 mmol, 48 mg) and [1,1'-biphenyl]-4-carbaldehyde (82 mg, 0.45 mmol). After 24 h, purification by column chromatography using 12% ethyl acetate in PE yielded **C-3** (84 mg, 82%) as a yellow solid.

^{1}H NMR (400 MHz, DMSO-d_6) δ 12.98 (s, 1H), 9.82 (s, 1H), 8.38 (dt, J = 8.1, 1.1 Hz, 1H), 8.16 (dt, J = 8.2, 1.1 Hz, 1H), 7.91 – 7.81 (m, 4H), 7.79 – 7.72 (m, 3H), 7.67 – 7.63 (m, 1H), 7.55 – 7.50 (m, 2H), 7.46 – 7.42 (m, 1H), 6.98 (s, 1H).

^{13}C NMR{^{1}H} (101 MHz, DMSO-d_6) δ 200.0, 154.6, 144.7, 143.4, 139.0, 136.8, 129.6, 129.5, 129.2, 129.2, 128.3, 127.0, 126.6, 126.6, 125.4, 123.7, 122.3, 112.8, 106.6.

ESI-HRMS: m/z calculated for $C_{23}H_{17}O_3$ $[M+H]^+$ calcd. 341.1172, found 341.1174.

4-(*tert*-butyl)phenyl)(1,4-dihydroxynaphthalen-2-yl)methanone (C-4):

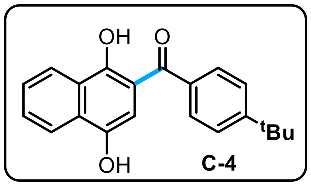

GP1 was followed using 1,4-naphthaquinone (0.3 mmol, 48 mg) and 4-(*tert*-butyl)benzaldehyde (73 mg, 0.45 mmol). After 24 h, purification by column chromatography using 8% ethyl acetate in PE yielded **C-4** (91 mg, 95%) as a yellow solid.

^{1}H NMR (400 MHz, $CDCl_3$) δ 13.59 (s, 1H), 8.51 (ddd, J = 8.3, 1.4, 0.7 Hz, 1H), 8.13 (ddd, J = 8.3, 1.3, 0.7 Hz, 1H), 7.69 – 7.56 (m, 4H), 7.49 – 7.45 (m, 2H), 6.95 (s, 1H), 5.63 (s, 1H), 1.33 (s, 9H).

^{13}C NMR{^{1}H} (101 MHz, $CDCl_3$) δ 200.9, 158.6, 155.5, 142.8, 135.5, 130.0, 129.1, 126.6, 126.2, 125.4, 124.7, 121.9, 114.9, 111.7, 108.4, 35.1, 31.2.

ESI-HRMS: m/z calculated for $C_{21}H_{21}O_3$ $[M+H]^+$ calcd. 321.1485, found 321.1490.

(1,4-dihydroxynaphthalen-2-yl)(4-methoxyphenyl)methanone (C-5):[3]

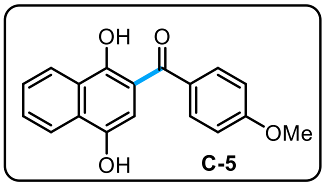

GP1 was followed using 1,4-naphthaquinone (0.3 mmol, 48 mg) and 4-methoxybenzaldehyde (61 mg, 0.45 mmol). After 24 h, purification by column chromatography using 15% ethyl acetate in PE yielded **C-5** (80 mg, 91%) as a yellow solid.

^{1}H NMR (400 MHz, DMSO-d_6) δ 12.82 (s, 1H), 9.77 (s, 1H), 8.34 (ddd, J = 8.3, 1.3, 0.7 Hz, 1H), 8.14 (ddd, J = 8.3, 1.3, 0.7 Hz, 1H), 7.78 – 7.74 (m, 2H), 7.70 (ddd, J = 8.3, 6.9, 1.3 Hz, 1H), 7.62 (ddd, J = 8.2, 6.9, 1.3 Hz, 1H), 7.16 – 7.11 (m, 2H), 6.97 (s, 1H), 3.88 (s, 3H).

^{13}C NMR{^{1}H} (101 MHz, DMSO-d_6) δ 198.8, 162.4, 153.9, 144.6, 131.5, 130.2, 129.1, 128.9, 126.4, 125.4, 123.6, 122.2, 113.8, 113.1, 106.8, 55.6.

(1,4-dihydroxynaphthalen-2-yl)(4-methoxyphenyl)methanone (C-6):

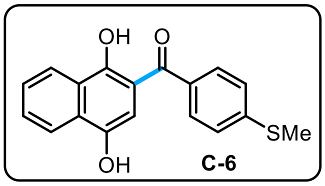

C-6

GP1 was followed using 1,4-naphthaquinone (0.3 mmol, 48 mg) and 4-methoxybenzaldehyde (68 mg, 0.45 mmol). After 24 h, purification by column chromatography using 12% ethyl acetate in PE yielded **C-6** (54 mg, 54%) as a yellow solid.

^{1}H NMR (400 MHz, DMSO-d_6) δ 12.79 (s, 1H), 10.10 (s, 1H), 8.14 – 8.09 (m, 2H), 7.88 (d, J = 3.2 Hz, 2H), 7.70 – 7.67 (m, 2H), 7.48 – 7.43 (m, 2H), 6.91 (s, 1H), 2.57 (s, 3H).

^{13}C NMR{^{1}H} (101 MHz, DMSO-d_6) δ 192.63, 154.09, 144.62, 137.11, 135.69, 134.90, 133.97, 130.93, 129.64, 126.51, 126.10, 124.80, 122.21, 113.00, 106.58, 14.05.

ESI-HRMS: m/z calculated for $C_{18}H_{14}O_3S$ NH_4 $[M+NH_4]^+$ calcd. 328.1002, found 328.0993.

(1,4-dihydroxynaphthalen-2-yl)(4-isopropoxyphenyl)methanone (C-7):

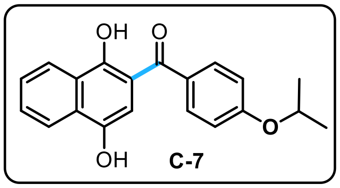

C-7

GP1 was followed using 1,4-naphthaquinone (0.3 mmol, 48 mg) and 4-isopropoxybenzaldehyde (74 mg, 0.45 mmol). After 24 h, purification by column chromatography using 13% ethyl acetate in PE yielded **C-7** (84 mg, 87%) as a yellow solid.

^{1}H NMR (400 MHz, $CDCl_3$) δ 13.48 (s, 1H), 8.49 (ddd, J = 8.3, 1.3, 0.7 Hz, 1H), 8.17 – 8.12 (m, 1H), 7.66 – 7.63 (m, 2H), 7.58 (ddd, J = 8.2, 6.9, 1.3 Hz, 1H), 6.94 (s, 1H), 6.88 – 6.84 (m, 2H), 6.77 – 6.71 (m, 1H), 5.94 (s, 1H), 4.53 (hept, J = 6.1 Hz, 1H), 1.32 (d, J = 6.1 Hz, 6H).

^{13}C NMR{^{1}H} (101 MHz, $CDCl_3$) δ 199.7, 161.2, 158.2, 142.8, 131.7, 129.9, 129.4, 126.6, 124.6, 121.9, 118.0, 116.2, 115.2, 111.8, 108.4, 70.4, 22.0.

ESI-HRMS: m/z calculated for $C_{20}H_{19}O_4$ $[M+H]^+$ calcd. 323.1278, found 323.1286.

(1,4-dihydroxynaphthalen-2-yl)(4-(trifluoromethoxy)phenyl)methanone (C-8):

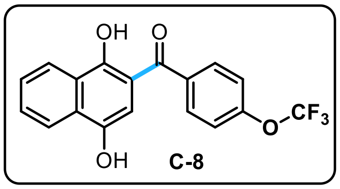

C-8

GP1 was followed using 1,4-naphthaquinone (0.3 mmol, 48 mg) and 4-(trifluoromethoxy)benzaldehyde (86 mg, 0.45 mmol). After 24 h, purification by column chromatography using 13% ethyl acetate in PE yielded **C-8** (89 mg, 85%) as a yellow solid.

^{1}H NMR (400 MHz, DMSO-d_6) δ 12.76 (s, 1H), 9.82 (s, 1H), 8.36 (ddd, J = 8.3, 1.3, 0.7 Hz, 1H), 8.14 (ddd, J = 8.4, 1.4, 0.7 Hz, 1H), 7.91 – 7.86 (m, 2H), 7.73 (ddd, J = 8.3, 6.9, 1.3 Hz, 1H), 7.64 (ddd, J = 8.2, 6.9, 1.3 Hz, 1H), 7.59 (ddt, J = 7.8, 2.2, 1.1 Hz, 2H), 6.85 (s, 1H).

^{13}C NMR{^{1}H} (101 MHz, DMSO-d_6) δ 198.9, 154.5, 150.49 (q, J_{C-F} = 2.1 Hz), 144.8, 136.9, 131.2, 129.6, 129.3, 126.6, 125.3, 123.7, 122.3, 120.8, 118.7, 112.8, 106.2.

^{19}F NMR (376 MHz, DMSO) δ -56.21.

ESI-HRMS: m/z calculated for $C_{18}H_{12}F_3O_4$ $[M+H]^+$ calcd. 349.0682, found 349.0683.

(1,4-dihydroxynaphthalen-2-yl)(4-fluorophenyl)methanone (C-9):[3]

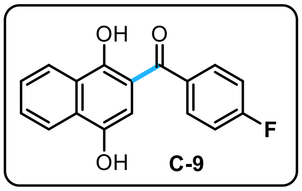

GP1 was followed using 1,4-naphthaquinone (0.3 mmol, 48 mg) and 4-fluorobenzaldehyde (56 mg, 0.45 mmol). After 24 h, purification by column chromatography using 10% ethyl acetate in PE yielded **C-9** (76 mg, 89%) as a yellow solid.

^{1}H NMR (400 MHz, DMSO-d_6) δ 12.78 (s, 1H), 9.80 (s, 1H), 8.35 (ddd, J = 8.3, 1.4, 0.7 Hz, 1H), 8.15 (ddd, J = 8.3, 1.3, 0.7 Hz, 1H), 7.86 – 7.80 (m, 2H), 7.72 (ddd, J = 8.3, 6.9, 1.3 Hz, 1H), 7.63 (ddd, J = 8.3, 6.9, 1.3 Hz, 1H), 7.47 – 7.40 (m, 2H), 6.89 (s, 1H).

^{13}C NMR{^{1}H} (101 MHz, DMSO-d_6) δ 198.9, 164.1 (d, $^{1}J_{C-F}$ = 250.3 Hz), 154.4, 144.8, 134.5 (d, $^{4}J_{C-F}$ = 3.0 Hz), 131.8 (d, $^{3}J_{C-F}$ = 9.2 Hz), 129.4, 129.2, 126.6, 125.4, 123.7, 122.3, 115.5 (d, $^{2}J_{C-F}$ = 21.9 Hz), 112.9, 106.5

^{19}F NMR (376 MHz, DMSO) δ -107.16.

(4-bromophenyl)(1,4-dihydroxynaphthalen-2-yl)methanone (C-10):

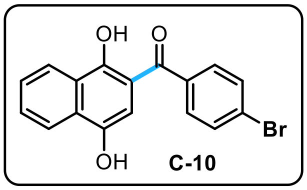

GP1 was followed using 1,4-naphthaquinone (0.3 mmol, 48 mg) and 4-bromobenzaldehyde (83 mg, 0.45 mmol). After 24 h, purification by column chromatography using 12% ethyl acetate in PE yielded **C-10** (90 mg, 88%) as a yellow solid.

^{1}H NMR (400 MHz, DMSO-d_6) δ 12.75 (s, 1H), 9.79 (s, 1H), 8.38 – 8.32 (m, 1H), 8.13 (dt, J = 8.3, 1.1 Hz, 1H), 7.84 – 7.78 (m, 2H), 7.74 – 7.61 (m, 4H), 6.84 (s, 1H).

^{13}C NMR{^{1}H} (101 MHz, DMSO-d_6) δ 199.2, 154.5, 144.8, 137.0, 131.5, 130.8, 129.6, 129.3, 126.6, 125.6, 125.3, 123.7, 122.3, 112.8, 106.3.

ESI-HRMS: m/z calculated for $C_{17}H_{12}BrO_3$ $[M+H]^+$ calcd. 342.9964, found 342.9965.

(1,4-dihydroxynaphthalen-2-yl)(4-iodophenyl)methanone (C-11):

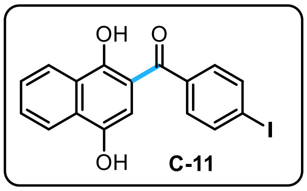

GP1 was followed using 1,4-naphthaquinone (0.3 mmol, 48 mg) and 4-iodobenzaldehyde (105 mg, 0.45 mmol). After 24 h, purification by column chromatography using 12% ethyl acetate in PE yielded **C-11** (84 mg, 72%) as a yellow solid.

^{1}H NMR (400 MHz, DMSO-*d*$_6$) δ 12.73 (s, 1H), 9.77 (s, 1H), 8.35 (ddd, *J* = 8.3, 1.4, 0.7 Hz, 1H), 8.15 – 8.11 (m, 1H), 8.02 – 7.97 (m, 2H), 7.73 (ddd, *J* = 8.3, 6.9, 1.3 Hz, 1H), 7.66 – 7.62 (m, 1H), 7.53 – 7.49 (m, 2H), 6.83 (s, 1H).

^{13}C NMR{^{1}H} (101 MHz, DMSO-*d*$_6$) δ 199.5, 154.4, 144.7, 137.3, 137.2, 130.5, 129.5, 129.2, 126.6, 125.3, 123.7, 122.2, 112.8, 106.3, 99.7.

ESI-HRMS: m/z calculated for $C_{17}H_{12}IO_3$ $[M+H]^+$ calcd. 390.9826, found 390.9823.

(1,4-dihydroxynaphthalen-2-yl)(4-(trifluoromethyl)phenyl)methanone (C-12):

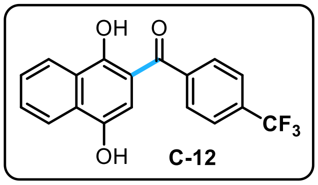

GP1 was followed using 1,4-naphthaquinone (0.3 mmol, 48 mg) and 4-(trifluoromethyl)benzaldehyde (78 mg, 0.45 mmol). After 24 h, purification by column chromatography using 10% ethyl acetate in PE yielded **C-12** (86 mg, 86%) as a yellow solid.

^{1}H NMR (400 MHz, $CDCl_3$) δ 13.43 (s, 1H), 8.54 – 8.49 (m, 1H), 8.12 (dt, *J* = 8.4, 0.9 Hz, 1H), 7.77 – 7.70 (m, 5H), 7.62 (ddd, *J* = 8.2, 6.9, 1.3 Hz, 1H), 6.73 (s, 1H), 5.26 (s, 1H).

^{13}C NMR{^{1}H} (101 MHz, $CDCl_3$) δ 199.6, 159.3, 143.1, 141.5, 138.8, 134.2, 133.2 (q, *J* = 32.8 Hz), 132.0, 130.6, 129.8, 129.1, 127.0, 126.6, 125.6 (q, *J* = 3.8 Hz), 125.1, 124.9, 122.4, 122.0, 119.7, 111.2, 107.3.

^{19}F NMR (376 MHz, $CDCl_3$) δ -63.45.

ESI-HRMS: m/z calculated for $C_{18}H_{12}F_3O_3$ $[M+H]^+$ calcd. 333.0733, found 333.0734.

4-(1,4-dihydroxy-2-naphthoyl)benzonitrile (C-13):

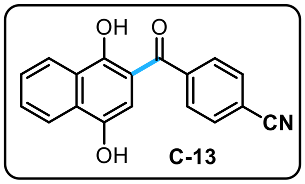

GP1 was followed using 1,4-naphthaquinone (0.3 mmol, 48 mg) and 4-formylbenzonitrile (59 mg, 0.45 mmol). After 24 h, purification by column chromatography using 18% ethyl acetate in PE yielded **C-13** (58 mg, 67%) as a yellow solid.

^{1}H NMR (400 MHz, DMSO-*d*$_6$) δ 12.68 (s, 1H), 9.84 (s, 1H), 8.36 (ddd, *J* = 8.3, 1.4, 0.7 Hz, 1H), 8.15 – 8.11 (m, 1H), 8.10 – 8.05 (m, 2H), 7.91 – 7.85 (m, 2H), 7.74 (ddd, *J* = 8.3, 6.9, 1.3 Hz, 1H), 7.65 (ddd, *J* = 8.2, 6.9, 1.3 Hz, 1H), 6.73 (s, 1H).

^{13}C NMR{^{1}H} (101 MHz, DMSO-d_6) δ 199.0, 154.8, 145.0, 142.0, 132.6, 129.9, 129.5, 129.3, 126.8, 125.3, 123.8, 122.4, 118.3, 113.9, 112.7, 106.0.

ESI-HRMS: m/z calculated for $C_{18}H_{12}NO_3$ $[M+H]^+$ calcd. 290.0812, found 290.0811.

methyl 4-(1,4-dihydroxy-2-naphthoyl)benzoate (C-14):

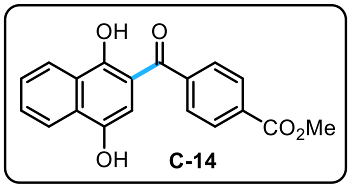

GP1 was followed using 1,4-naphthaquinone (0.3 mmol, 48 mg) and methyl 4-formylbenzoate (74 mg, 0.45 mmol). After 24 h, purification by column chromatography using 18% ethyl acetate in PE yielded **C-14** (71 mg, 73%) as a yellow solid.

^{1}H NMR (400 MHz, DMSO-d_6) δ 12.80 (s, 1H), 9.79 (s, 1H), 8.36 (d, J = 8.3 Hz, 1H), 8.14 (t, J = 8.2 Hz, 3H), 7.85 (d, J = 8.0 Hz, 2H), 7.74 (t, J = 7.5 Hz, 1H), 7.65 (t, J = 7.6 Hz, 1H), 6.80 (s, 1H), 3.92 (s, 3H).

^{13}C NMR{^{1}H} (101 MHz, DMSO-d_6) δ 199.7, 165.7, 154.8, 144.8, 141.9, 132.0, 129.7, 129.4, 129.2, 128.9, 126.7, 125.3, 123.8, 122.3, 112.7, 106.2, 52.5.

ESI-HRMS: m/z calculated for $C_{19}H_{15}O_5$ $[M+H]^+$ calcd. 323.0914, found 323.0914.

***p*-tolyl 4-(1,4-dihydroxy-2-naphthoyl)benzoate (C-15):**

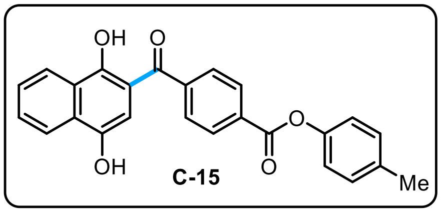

GP1 was followed using 1,4-naphthaquinone (0.3 mmol, 48 mg) and p-tolyl 4-formylbenzoate (108 mg, 0.45 mmol). After 24 h, purification by column chromatography using 18% ethyl acetate in PE yielded **C-15** (60 mg, 50%) as a yellow solid.

^{1}H NMR (400 MHz, DMSO-d_6) δ 9.33 (s, 1H), 8.12 – 7.92 (m, 4H), 7.89 – 7.80 (m, 3H), 7.31 – 7.22 (m, 1H), 7.17 – 7.00 (m, 3H), 6.72 – 6.69 (m, 2H), 6.66 (s, 1H), 2.48 (s, 3H).

^{13}C NMR{^{1}H} (101 MHz, DMSO-d_6) δ 184.8, 184.6, 156.1, 151.2, 134.9, 134.2, 134.2, 131.8, 131.6, 130.5, 130.4, 130.3, 129.8, 127.3, 126.2, 125.7, 122.0, 121.6, 115.9, 115.4, 34.1.

ESI-HRMS: m/z calculated for $C_{25}H_{19}O_5$ $[M+H]^+$ calcd. 399.1227, found 399.1225.

***N*-(4-(1,4-dihydroxy-2-naphthoyl)phenyl)acetamide (C-16):**

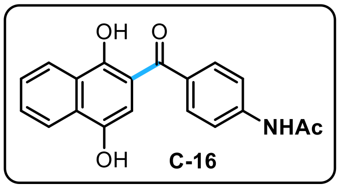

GP1 was followed using 1,4-naphthaquinone (0.3 mmol, 48 mg) and *N*-(4-formylphenyl)acetamide (74 mg, 0.45 mmol). After 24 h, purification by column chromatography using 60% ethyl acetate in PE yielded **C-16** (73 mg, 76%) as a yellow solid.

^{1}**H NMR** (400 MHz, DMSO-d_6) δ 12.89 (s, 1H), 10.33 (s, 1H), 9.77 (s, 1H), 8.38 – 8.31 (m, 1H), 8.16 – 8.11 (m, 1H), 7.80 (d, J = 8.7 Hz, 2H), 7.76 – 7.69 (m, 3H), 7.62 (ddd, J = 8.2, 6.8, 1.3 Hz, 1H), 6.96 (s, 1H), 2.12 (s, 3H).

^{13}**C NMR{^{1}H}** (101 MHz, DMSO-d_6) δ 199.0, 169.0, 154.2, 144.5, 142.8, 132.0, 130.8, 130.4, 129.2, 129.0, 126.5, 125.4, 123.6, 122.2, 118.6, 118.2, 112.9, 106.8, 24.2.

ESI-HRMS: m/z calculated for $C_{19}H_{16}NO_5$ $[M+H]^+$ calcd. 322.1074, found 322.1074.

(1,4-dihydroxynaphthalen-2-yl)(3-methoxyphenyl)methanone (C-17):

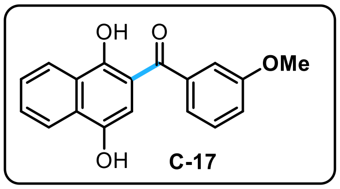

GP1 was followed using 1,4-naphthaquinone (0.3 mmol, 48 mg) and 3-methoxybenzaldehyde (61 mg, 0.45 mmol). After 24 h, purification by column chromatography using 15% ethyl acetate in PE yielded **C-17** (82 mg, 93%) as a yellow solid.

^{1}**H NMR** (400 MHz, $CDCl_3$+DMSO-d_6) δ 13.32 (s, 1H), 9.26 (s, 1H), 8.35 (dt, J = 8.1, 1.0 Hz, 1H), 8.13 – 8.07 (m, 1H), 7.59 (ddd, J = 8.3, 6.8, 1.3 Hz, 1H), 7.50 (ddd, J = 8.3, 6.9, 1.3 Hz, 1H), 7.39 (t, J = 7.9 Hz, 1H), 7.25 – 7.21 (m, 1H), 7.16 (dd, J = 2.7, 1.5 Hz, 1H), 7.07 (ddd, J = 8.3, 2.7, 1.0 Hz, 1H), 6.92 (s, 1H), 3.81 (s, 3H).

^{13}**C NMR{^{1}H}** (101 MHz, $CDCl_3$+DMSO-d_6) δ 200.1, 158.8, 156.5, 144.1, 139.1, 129.5, 129.0, 129.0, 125.7, 125.2, 123.6, 121.9, 120.7, 116.9, 113.5, 111.4, 106.4, 55.0.

ESI-HRMS: m/z calculated for $C_{18}H_{15}O_4$ $[M+H]^+$ calcd. 295.0965, found 295.0968.

(1,4-dihydroxynaphthalen-2-yl)(3-fluorophenyl)methanone (C-18):

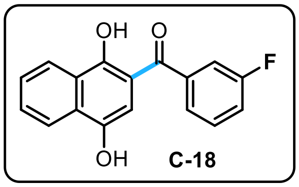

GP1 was followed using 1,4-naphthaquinone (0.3 mmol, 48 mg) and 3-fluorobenzaldehyde (56 mg, 0.45 mmol). After 24 h, purification by column chromatography using 12% ethyl acetate in PE yielded **C-18** (80 mg, 95%) as a yellow solid.

^{1}**H NMR** (400 MHz, DMSO-d_6) δ 12.84 (s, 1H), 9.81 (s, 1H), 8.36 (ddd, J = 8.3, 1.4, 0.7 Hz, 1H), 8.14 (ddd, J = 8.3, 1.3, 0.7 Hz, 1H), 7.76 – 7.70 (m, 1H), 7.67 – 7.61 (m, 2H), 7.58 – 7.49 (m, 3H), 6.86 (s, 1H).

^{13}**C NMR{^{1}H}** (101 MHz, DMSO-d_6) δ 198.89 (d, $^{4}J_{C\text{-}F}$ = 2.1 Hz), 161.74 (d, $^{1}J_{C\text{-}F}$ = 245.6 Hz), 154.89, 144.78, 140.08 (d, $^{3}J_{C\text{-}F}$ = 6.8 Hz), 130.76 (d, $^{3}J_{C\text{-}F}$ = 8.1 Hz), 129.68, 129.40, 126.65, 125.31, 124.91 (d, $^{4}J_{C\text{-}F}$ = 3.0 Hz), 123.76, 122.29, 118.58 (d, $^{2}J_{C\text{-}F}$ = 21.1 Hz), 115.44 (d, $^{2}J_{C\text{-}F}$ = 22.9 Hz), 112.56, 106.25.

^{19}**F NMR** (376 MHz, DMSO) δ -111.68.

ESI-HRMS: m/z calculated for $C_{17}H_{12}FO_3$ $[M+H]^+$ calcd. 283.0765, found 283.0763.

(1,4-dihydroxynaphthalen-2-yl)(3-chlorophenyl)methanone (C-19):

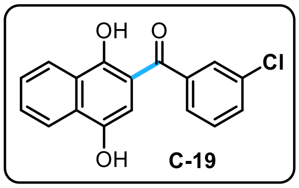

GP1 was followed using 1,4-naphthaquinone (0.3 mmol, 48 mg) and 3-chlorobenzaldehyde (63 mg, 0.45 mmol). After 24 h, purification by column chromatography using 12% ethyl acetate in PE yielded **C-19** (82 mg, 92%) as a yellow solid.

^{1}H NMR (400 MHz, DMSO-d_6) δ 12.79 (s, 1H), 9.83 (s, 1H), 8.36 (ddd, J = 8.4, 1.3, 0.7 Hz, 1H), 8.16 – 8.12 (m, 1H), 7.78 – 7.70 (m, 3H), 7.69 – 7.61 (m, 3H), 6.83 (s, 1H).

^{13}C NMR{^{1}H} (101 MHz, DMSO-d_6) δ 198.8, 154.8, 144.8, 139.9, 133.3, 131.4, 130.5, 129.7, 129.4, 128.2, 127.3, 126.7, 125.3, 123.8, 122.3, 112.6, 106.2.

ESI-HRMS: m/z calculated for $C_{17}H_{12}ClO_3$ $[M+H]^+$ calcd. 299.0469, found 299.0466.

(2-chlorophenyl)(1,4-dihydroxynaphthalen-2-yl)methanone (C-20):

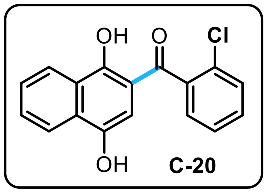

GP1 was followed using 1,4-naphthaquinone (0.3 mmol, 48 mg) and 2-chlorobenzaldehyde (63 mg, 0.45 mmol). After 24 h, purification by column chromatography using 12% ethyl acetate in PE yielded **C-20** (80 mg, 90%) as a yellow solid.

^{1}H NMR (400 MHz, $CDCl_3$) δ 13.38 (s, 1H), 8.52 (ddd, J = 8.3, 1.4, 0.7 Hz, 1H), 8.10 (ddd, J = 8.3, 1.2, 0.7 Hz, 1H), 7.70 (ddd, J = 8.3, 6.9, 1.4 Hz, 1H), 7.60 (ddd, J = 8.3, 6.9, 1.3 Hz, 1H), 7.43 (dt, J = 7.8, 1.1 Hz, 1H), 7.41 – 7.33 (m, 3H), 6.45 (s, 1H), 5.56 (s, 1H).

^{13}C NMR{^{1}H} (101 MHz, $CDCl_3$) δ 199.7, 159.0, 143.4, 137.7, 131.2, 130.8, 130.5, 130.1, 130.1, 128.5, 126.9, 126.8, 126.0, 124.8, 122.0, 112.2, 107.1.

ESI-HRMS: m/z calculated for $C_{17}H_{12}ClO_3$ $[M+H]^+$ calcd. 299.0469, found 299.0471.

(2-bromophenyl)(1,4-dihydroxynaphthalen-2-yl)methanone (C-21):

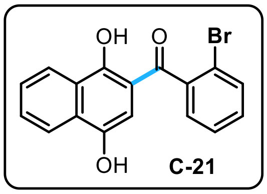

GP1 was followed using 1,4-naphthaquinone (0.3 mmol, 48 mg) and 2-bromobenzaldehyde (83 mg, 0.45 mmol). After 24 h, purification by column chromatography using 12% ethyl acetate in PE yielded **C-21** (86 mg, 84%) as a yellow solid.

^{1}H NMR (400 MHz, DMSO-d_6) δ 13.62 (s, 1H), 10.30 (d, J = 1.0 Hz, 1H), 8.95 – 8.87 (m, 1H), 8.68 – 8.61 (m, 1H), 8.34 (d, J = 8.1 Hz, 1H), 8.30 – 8.25 (m, 1H), 8.20 – 8.15 (m, 1H), 8.13 – 8.03 (m, 3H), 7.00 (s, 1H).

^{13}C NMR{^{1}H} (101 MHz, DMSO-d_6) δ 200.4, 155.9, 145.0, 139.5, 132.8, 131.7, 130.2, 129.9, 128.5, 128.0, 126.8, 125.1, 123.8, 122.3, 118.2, 112.2, 105.5.

ESI-HRMS: m/z calculated for $C_{17}H_{12}BrO_3$ $[M+H]^+$ calcd. 342.9964, found 342.9956.

(1,4-dihydroxynaphthalen-2-yl)(2-methoxyphenyl)methanone (C-22):

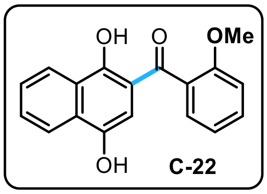

GP1 was followed using 1,4-naphthaquinone (0.3 mmol, 48 mg) and 2-methoxybenzaldehyde (61 mg, 0.45 mmol). After 24 h, purification by column chromatography using 15% ethyl acetate in PE yielded **C-22** (76 mg, 86%) as a yellow solid.

^{1}H NMR (400 MHz, DMSO-d_6) δ 13.37 (s, 1H), 9.69 (s, 1H), 8.36 (ddd, J = 8.3, 1.3, 0.7 Hz, 1H), 8.09 (dt, J = 8.3, 1.1 Hz, 1H), 7.72 (ddd, J = 8.3, 6.9, 1.3 Hz, 1H), 7.66 – 7.54 (m, 2H), 7.38 (dd, J = 7.4, 1.7 Hz, 1H), 7.25 (dd, J = 8.5, 0.9 Hz, 1H), 7.14 (td, J = 7.4, 0.9 Hz, 1H), 6.60 (s, 1H), 3.75 (s, 3H).

^{13}C NMR{^{1}H} (101 MHz, DMSO-d_6) δ 201.6, 155.9, 155.2, 144.6, 132.0, 129.9, 129.7, 128.2, 127.8, 126.6, 125.0, 123.8, 122.2, 120.7, 113.2, 112.1, 106.4, 55.8.

ESI-HRMS: m/z calculated for $C_{18}H_{15}O_4$ $[M+H]^+$ calcd. 295.0965, found 295.0971.

ethyl 2-(1,4-dihydroxy-2-naphthoyl)benzoate (C-23):

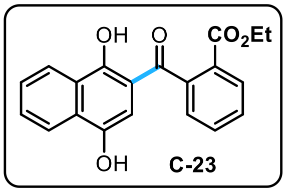

GP1 was followed using 1,4-naphthaquinone (0.3 mmol, 48 mg) and 2-methoxybenzaldehyde (80 mg, 0.45 mmol). After 24 h, the crude reaction mixture was analysed in ESI-HRMS (as the conversion was less). The expected mass of the desired compound (**C-23**) was detected in HRMS.

ESI-HRMS: m/z calculated for $C_{20}H_{16}O_5Na$ $[M+Na]^+$ calcd. 359.0890, found 359.0892.

((1,4-dihydroxynaphthalen-2-yl)(4-(hydroxymethyl)phenyl)methanone (C-24):

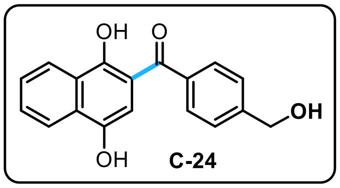

GP1 was followed using 1,4-naphthaquinone (0.3 mmol, 48 mg) and 4-(hydroxymethyl)benzaldehyde (62 mg, 0.45 mmol). After 24 h, purification by column chromatography using 45% ethyl acetate in PE yielded **C-24** (48 mg, 54%) as a yellow solid.

^{1}H NMR (400 MHz, DMSO-d_6) δ 12.98 (s, 1H), 9.77 (s, 1H), 8.36 (ddd, J = 8.3, 1.3, 0.7 Hz, 1H), 8.13 (ddd, J = 8.3, 1.3, 0.7 Hz, 1H), 7.75 – 7.69 (m, 3H), 7.66 – 7.62 (m, 1H), 7.56 – 7.52 (m, 2H), 6.91 (s, 1H), 5.41 (t, J = 5.7 Hz, 1H), 4.63 (d, J = 5.7 Hz, 2H).

^{13}C NMR{^{1}H} (101 MHz, DMSO-d_6) δ 200.3, 154.6, 146.8, 144.6, 136.2, 129.4, 129.1, 128.8, 126.5, 126.1, 125.3, 123.7, 122.2, 112.7, 106.7, 62.5.

ESI-HRMS: m/z calculated for $C_{18}H_{15}O_4$ $[M+H]^+$ calcd. 295.0965, found 295.0961.

(1,4-dihydroxynaphthalen-2-yl)(2,5-dimethoxyphenyl)methanone (C-25):

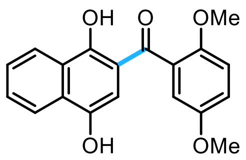

C-25

GP1 was followed using 1,4-naphthaquinone (0.3 mmol, 48 mg) and 2,5-dimethoxybenzaldehyde (75 mg, 0.45 mmol). After 24 h, purification by column chromatography using 18% ethyl acetate in PE yielded **C-25** (82 mg, 85%) as a yellow solid.

^{1}H NMR (400 MHz, DMSO-d_6) δ 13.31 (s, 1H), 9.67 (s, 1H), 8.35 (ddd, J = 8.3, 1.4, 0.7 Hz, 1H), 8.09 (dt, J = 8.2, 1.1 Hz, 1H), 7.73 (ddd, J = 8.3, 6.9, 1.4 Hz, 1H), 7.64 (ddd, J = 8.2, 6.9, 1.3 Hz, 1H), 7.19 (d, J = 9.1 Hz, 1H), 7.13 (dd, J = 9.1, 3.1 Hz, 1H), 6.98 (d, J = 3.0 Hz, 1H), 6.61 (s, 1H), 3.76 (s, 3H), 3.70 (s, 3H).

^{13}C NMR{^{1}H} (101 MHz, DMSO-d_6) δ 201.0, 155.1, 153.1, 149.8, 144.6, 129.8, 129.6, 128.3, 126.5, 124.9, 123.7, 122.2, 116.7, 113.4, 113.3, 113.0, 106.3, 56.2, 55.7.

ESI-HRMS: m/z calculated for $C_{19}H_{16}O_5Na$ $[M+Na]^+$ calcd. 347.089, found 347.0885.

(1,4-dihydroxynaphthalen-2-yl)(2-ethoxy-5-nitrophenyl)methanone (C-26):

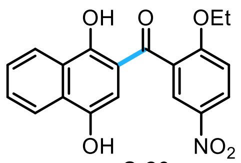

C-26

GP1 was followed using 1,4-naphthaquinone (0.3 mmol, 48 mg) and 2-ethoxy-5-nitrobenzaldehyde (88 mg, 0.45 mmol). After 24 h, purification by column chromatography using 18% ethyl acetate in PE yielded **C-26** (61 mg, 58%) as a yellow solid.

^{1}H NMR (400 MHz, DMSO-d_6) δ 12.98 (s, 1H), 9.67 (s, 1H), 8.45 (dd, J = 9.3, 2.9 Hz, 1H), 8.38 – 8.32 (m, 2H), 8.10 (ddd, J = 8.3, 1.3, 0.7 Hz, 1H), 7.73 (ddd, J = 8.3, 6.9, 1.3 Hz, 1H), 7.64 (ddd, J = 8.3, 6.9, 1.3 Hz, 1H), 7.47 (d, J = 9.3 Hz, 1H), 6.56 (s, 1H), 4.24 (q, J = 7.0 Hz, 2H), 1.13 (t, J = 6.9 Hz, 3H).

^{13}C NMR{^{1}H} (101 MHz, DMSO-d_6) δ 198.2, 160.3, 155.1, 144.8, 140.6, 130.0, 129.7, 128.3, 127.7, 126.6, 125.0, 124.4, 123.8, 122.2, 113.4, 113.0, 105.8, 65.4, 14.0.

ESI-HRMS: m/z calculated for $C_{19}H_{15}NO_6Na$ $[M+Na]^+$ calcd. 376.0792, found 376.0786.

2-bromo-4-methylphenyl)(1,4-dihydroxynaphthalen-2-yl)methanone (C-27):

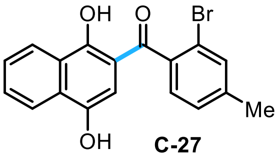

C-27

GP1 was followed using 1,4-naphthaquinone (0.3 mmol, 48 mg) and 2-bromo-4-methylbenzaldehyde (89 mg, 0.45 mmol). After 24 h, purification by column chromatography using 12% ethyl acetate in PE yielded **C-27** (82 mg, 77%) as a yellow solid.

^{1}**H NMR** (400 MHz, DMSO-d_6) δ 13.13 (s, 1H), 9.74 (s, 1H), 8.38 (ddd, J = 8.3, 1.4, 0.7 Hz, 1H), 8.11 (ddd, J = 8.4, 1.3, 0.8 Hz, 1H), 7.75 (ddd, J = 8.3, 6.9, 1.3 Hz, 1H), 7.68 – 7.63 (m, 2H), 7.47 – 7.39 (m, 2H), 6.50 (s, 1H), 2.41 (s, 3H).

^{13}**C NMR{^{1}H}** (101 MHz, DMSO-d_6) δ 200.6, 155.8, 144.9, 142.0, 136.6, 133.0, 130.1, 129.8, 128.5, 128.4, 126.8, 125.0, 123.8, 122.3, 118.1, 112.3, 105.7, 20.5.

ESI-HRMS: m/z calculated for $C_{18}H_{14}BrO_3$ $[M+H]^+$ calcd. 357.0121, found 357.0127.

2,6-dichlorophenyl)(1,4-dihydroxynaphthalen-2-yl)methanone (C-28):

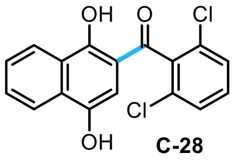

C-28

GP1 was followed using 1,4-naphthaquinone (0.3 mmol, 48 mg) and 2,6-dichlorobenzaldehyde (78 mg, 0.45 mmol). After 24 h, purification by column chromatography using 12% ethyl acetate in PE yielded **C-28** (59 mg, 59%) as a yellow solid.

^{1}**H NMR** (400 MHz, DMSO-d_6) δ 12.76 (s, 1H), 9.84 (s, 1H), 8.39 (d, J = 8.3 Hz, 1H), 8.12 (d, J = 8.3 Hz, 1H), 7.79 – 7.66 (m, 5H), 6.44 (s, 1H).

^{13}**C NMR{^{1}H}** (101 MHz, DMSO-d_6) δ 196.4, 155.9, 145.6, 132.3, 130.7, 130.6, 130.5, 130.2, 128.7, 127.1, 125.0, 123.9, 122.4, 120.4, 118.4, 107.1, 103.9.

ESI-HRMS: m/z calculated for $C_{17}H_{11}Cl_2O_3$ $[M+H]^+$ calcd. 333.0080, found 333.0079.

(1,4-dihydroxynaphthalen-2-yl)(3,5-dimethylphenyl)methanone (C-29):

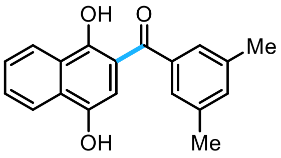

C-29

GP1 was followed using 1,4-naphthaquinone (0.3 mmol, 48 mg) and 3,5-dimethylbenzaldehyde (60 mg, 0.45 mmol). After 24 h, purification by column chromatography using 10% ethyl acetate in PE yielded **C-29** (62 mg, 90%) as a yellow solid.

^{1}**H NMR** (400 MHz, DMSO-d_6) δ 13.10 (s, 1H), 9.76 (s, 1H), 8.36 (ddd, J = 8.3, 1.3, 0.7 Hz, 1H), 8.13 (ddd, J = 8.3, 1.2, 0.7 Hz, 1H), 7.72 (ddd, J = 8.3, 6.9, 1.3 Hz, 1H), 7.63 (ddd, J = 8.2, 6.9, 1.3 Hz, 1H), 7.32 – 7.27 (m, 3H), 6.91 (s, 1H), 2.37 (d, J = 0.8 Hz, 6H).

^{13}**C NMR{^{1}H}** (101 MHz, DMSO-d_6) δ 200.9, 154.9, 144.5, 138.0, 137.7, 133.0, 129.5, 129.2, 126.5, 126.3, 125.3, 123.7, 122.2, 112.6, 106.7, 20.8.

ESI-HRMS: m/z calculated for $C_{19}H_{17}O_3$ $[M+H]^+$ calcd. 293.1172, found 293.1175.

(3,5-difluorophenyl)(1,4-dihydroxynaphthalen-2-yl)methanone (C-30):

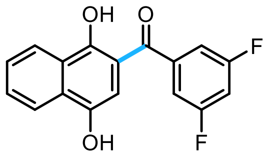

C-30

GP1 was followed using 1,4-naphthaquinone (0.3 mmol, 48 mg) and 3,5-difluorobenzaldehyde (63 mg, 0.45 mmol). After 24 h, purification by column chromatography using 12% ethyl acetate in PE yielded **C-30** (83 mg, 92%) as a yellow solid.

^{1}H NMR (400 MHz, DMSO-d_6) δ 12.66 (s, 1H), 9.83 (s, 1H), 8.36 (ddd, J = 8.3, 1.3, 0.7 Hz, 1H), 8.14 (ddd, J = 8.4, 1.3, 0.7 Hz, 1H), 7.74 (ddd, J = 8.3, 6.9, 1.3 Hz, 1H), 7.64 (ddd, J = 8.3, 6.9, 1.3 Hz, 1H), 7.58 (ddd, J = 9.3, 6.9, 2.4 Hz, 1H), 7.49 – 7.44 (m, 2H), 6.81 (s, 1H).

^{13}C NMR{^{1}H} (101 MHz, DMSO-d_6) δ 197.5 (t, $^4J_{C-F}$ = 2.4 Hz), 162.05 (dd, $^1J_{C-F}$ = 248.5, 12.5 Hz), 154.8, 144.9, 141.2 (t, $^3J_{C-F}$ = 8.5 Hz), 129.8, 129.5, 126.7, 125.3, 123.8, 122.3, 112.5, 112.25 – 111.77 (m), 107.0 (t, $^2J_{C-F}$ = 25.8 Hz), 106.0.

^{19}F NMR (376 MHz, DMSO) δ -107.90.

ESI-HRMS: m/z calculated for $C_{17}H_{11}F_2O_3$ $[M+H]^+$ calcd. 301.0671, found 301.0673.

(1,4-dihydroxynaphthalen-2-yl)(mesityl)methanone (C-31):

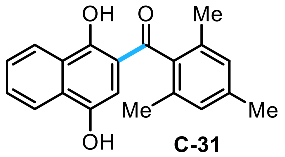

C-31

GP1 was followed using 1,4-naphthaquinone (0.3 mmol, 48 mg) and 2,4,6-trimethylbenzaldehyde (67 mg, 0.45 mmol). After 24 h, purification by column chromatography using 10% ethyl acetate in PE yielded **C-31** (59 mg, 64%) as a yellow solid.

^{1}H NMR (400 MHz, $CDCl_3$) δ 13.76 (s, 1H), 8.53 (ddd, J = 8.3, 1.4, 0.7 Hz, 1H), 8.13 – 8.09 (m, 1H), 7.70 (ddd, J = 8.3, 6.9, 1.4 Hz, 1H), 7.61 (ddd, J = 8.2, 6.9, 1.3 Hz, 1H), 6.91 – 6.87 (m, 2H), 6.40 (s, 1H), 5.20 (s, 1H), 2.31 (s, 3H), 2.13 (s, 6H).

^{13}C NMR{^{1}H} (101 MHz, $CDCl_3$) δ 205.8, 158.4, 143.5, 139.0, 138.8, 135.8, 134.1, 134.0, 130.2, 128.5, 126.7, 124.8, 122.0, 113.0, 106.5, 21.3, 19.4.

ESI-HRMS: m/z calculated for $C_{20}H_{19}O_3$ $[M+H]^+$ calcd. 307.1329, found 307.1333.

(1,4-dihydroxynaphthalen-2-yl)(4-methoxy-2,3-dimethylphenyl)methanone (C-32):

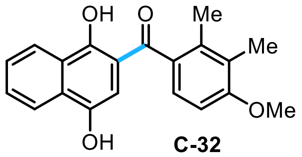

C-32

GP1 was followed using 1,4-naphthaquinone (0.3 mmol, 48 mg) and 4-methoxy-2,3-dimethylbenzaldehyde (74 mg, 0.45 mmol). After 24 h, purification by column chromatography using 15% ethyl acetate in PE yielded **C-32** (70 mg, 73%) as a yellow solid.

^{1}H NMR (400 MHz, DMSO-d_6) δ 13.56 (s, 1H), 9.65 (s, 1H), 8.37 (dt, J = 8.1, 1.0 Hz, 1H), 8.10 (dt, J = 8.2, 1.0 Hz, 1H), 7.73 (ddd, J = 8.3, 6.9, 1.3 Hz, 1H), 7.64 (ddd, J = 8.2, 6.9, 1.3 Hz, 1H), 7.22 (d, J = 8.4 Hz, 1H), 6.99 (d, J = 8.5 Hz, 1H), 6.61 (s, 1H), 3.87 (s, 3H), 2.17 (s, 3H), 2.15 (s, 3H).

^{13}C NMR{^{1}H} (101 MHz, DMSO-d_6) 203.7, 158.2, 155.5, 144.5, 134.7, 131.1, 129.8, 129.6, 126.6, 126.1, 125.9, 125.1, 123.7, 122.2, 113.2, 107.6, 106.4, 55.7, 17.0, 11.5.

ESI-HRMS: m/z calculated for $C_{20}H_{19}O_4$ $[M+H]^+$ calcd. 323.1278, found 323.1283.

(1,4-dihydroxynaphthalen-2-yl)(naphthalen-2-yl)methanone (C-33):

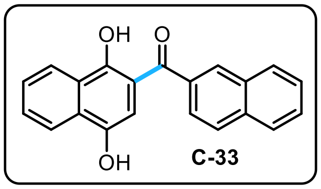

GP1 was followed using 1,4-naphthaquinone (0.3 mmol, 48 mg) and 2-naphthaldehyde (70 mg, 0.45 mmol). After 24 h, purification by column chromatography using 12% ethyl acetate in PE yielded **C-33** (62 mg, 66%) as a yellow solid.

^{1}H NMR (400 MHz, DMSO-d_6) δ 12.93 (s, 1H), 9.77 (s, 1H), 8.41 – 8.34 (m, 2H), 8.14 (d, J = 1.8 Hz, 2H), 8.08 – 8.05 (m, 1H), 7.84 (dd, J = 8.5, 1.7 Hz, 1H), 7.76 – 7.63 (m, 5H), 6.98 (s, 1H).

^{13}C NMR{^{1}H} (101 MHz, DMSO-d_6) δ 200.3, 154.6, 145.4, 144.7, 135.2, 134.2, 131.8, 129.8, 129.5, 129.2, 128.2, 127.8, 127.1, 126.6, 125.4, 125.1, 123.7, 122.3, 113.0, 107.9, 106.8.

ESI-HRMS: m/z calculated for $C_{21}H_{15}O_3$ $[M+H]^+$ calcd. 315.1016, found 315.1015.

(1,4-dihydroxynaphthalen-2-yl)(naphthalen-1-yl)methanone (C-34):

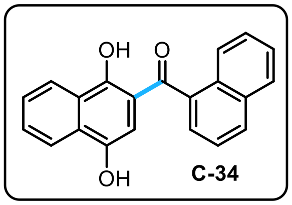

GP1 was followed using 1,4-naphthaquinone (0.3 mmol, 48 mg) and 1-naphthaldehyde (70 mg, 0.45 mmol). After 24 h, purification by column chromatography using 12% ethyl acetate in PE yielded **C-34** (61 mg, 65%) as a yellow solid.

^{1}H NMR (400 MHz, $CDCl_3$) δ 13.81 (s, 1H), 8.57 (ddd, J = 8.3, 1.3, 0.7 Hz, 1H), 8.12 – 8.08 (m, 1H), 8.01 – 7.96 (m, 1H), 7.93 – 7.86 (m, 2H), 7.71 (ddd, J = 8.3, 6.9, 1.4 Hz, 1H), 7.65 – 7.60 (m, 1H), 7.55 – 7.50 (m, 3H), 7.47 (ddd, J = 8.2, 6.8, 1.5 Hz, 1H), 6.49 (s, 1H), 2.13 (s, 1H).

^{13}C NMR{^{1}H} (101 MHz, $CDCl_3$) δ 202.8, 159.0, 143.0, 138.8, 136.2, 133.6, 130.7, 130.4, 129.9, 128.6, 127.4, 126.8, 126.7, 126.2, 126.0, 125.6, 124.9, 124.8, 122.0, 113.0, 107.8.

ESI-HRMS: m/z calculated for $C_{21}H_{15}O_3$ $[M+H]^+$ calcd. 315.1016, found 315.1018.

(1,4-dihydroxynaphthalen-2-yl)(thiazol-2-yl)methanone (C-35):

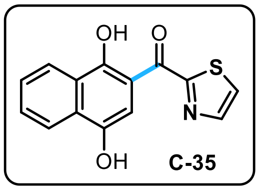

GP1 was followed using 1,4-naphthaquinone (0.3 mmol, 48 mg) and thiazole-2-carbaldehyde (51 mg, 0.45 mmol). After 24 h, purification by column chromatography using 22% ethyl acetate in PE yielded **C-35** (73 mg, 90%) as a yellow solid.

^{1}H NMR (400 MHz, DMSO-*d*$_6$) δ 13.84 (s, 1H), 9.95 (s, 1H), 8.41 – 8.31 (m, 4H), 8.15 – 8.12 (m, 1H), 7.75 (ddd, *J* = 8.3, 6.9, 1.3 Hz, 1H), 7.63 (ddd, *J* = 8.3, 6.9, 1.3 Hz, 1H).

^{13}C NMR{^{1}H} (101 MHz, DMSO-*d*$_6$) δ 184.6, 167.9, 157.5, 145.0, 144.9, 130.3, 130.1, 128.6, 126.6, 125.3, 124.2, 122.2, 111.4, 105.7.

ESI-HRMS: m/z calculated for $C_{14}H_{10}NO_3S$ $[M+H]^+$ calcd. 272.0376, found 272.0373.

((1,4-dihydroxynaphthalen-2-yl)(1H-pyrrol-2-yl)methanone (C-36):

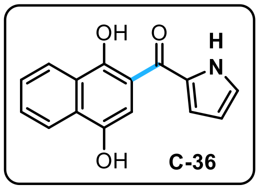

GP1 was followed using 1,4-naphthaquinone (0.3 mmol, 48 mg) and thiazole-2-carbaldehyde (43 mg, 0.45 mmol). After 24 h, purification by column chromatography using 22% ethyl acetate in PE yielded **C-36** (52 mg, 68%) as a yellow solid.

^{1}H NMR (400 MHz, DMSO-*d*$_6$) δ 13.42 (s, 1H), 12.18 (s, 1H), 9.79 (s, 1H), 8.30 (ddd, *J* = 8.3, 1.3, 0.7 Hz, 1H), 8.12 (dt, *J* = 8.1, 1.1 Hz, 1H), 7.68 (ddd, *J* = 8.3, 6.8, 1.3 Hz, 1H), 7.60 (ddd, *J* = 8.2, 6.9, 1.3 Hz, 1H), 7.51 (s, 1H), 7.29 (ddd, *J* = 3.5, 2.4, 1.3 Hz, 1H), 7.16 (ddd, *J* = 3.9, 2.5, 1.3 Hz, 1H), 6.40 (dt, *J* = 3.9, 2.3 Hz, 1H).

^{13}C NMR{^{1}H} (101 MHz, DMSO-*d*$_6$) δ 185.6, 154.1, 144.7, 129.7, 128.8, 128.5, 126.8, 126.3, 125.4, 123.4, 122.1, 119.4, 112.2, 111.0, 105.7.

ESI-HRMS: m/z calculated for $C_{15}H_{12}NO_3$ $[M+H]^+$ calcd. 254.0812, found 254.0812.

(1,4-dihydroxynaphthalen-2-yl)(furan-2-yl)methanone (C-37):[5]

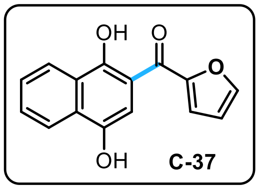

GP1 was followed using 1,4-naphthaquinone (0.3 mmol, 48 mg) and furan-2-carbaldehyde (44 mg, 0.45 mmol). After 24 h, purification by column chromatography using 12% ethyl acetate in PE yielded **C-37** (65 mg, 85%) as a yellow solid.

^{1}H NMR (400 MHz, DMSO-*d*$_6$) δ 13.25 (s, 1H), 9.88 (s, 1H), 8.35 – 8.31 (m, 1H), 8.21 (dd, *J* = 1.7, 0.8 Hz, 1H), 8.13 (dt, *J* = 8.2, 1.1 Hz, 1H), 7.74 – 7.70 (m, 1H), 7.63 (ddd, *J* = 8.2, 6.8, 1.3 Hz, 1H), 7.59 – 7.56 (m, 2H), 6.89 (dd, *J* = 3.6, 1.7 Hz, 1H).

^{13}C NMR{^{1}H} (101 MHz, DMSO-d_6) δ 183.6, 155.4, 151.3, 148.6, 145.0, 129.5, 129.1, 126.5, 125.3, 123.7, 122.1, 121.4, 113.0, 111.9, 105.0.

1,4-dihydroxynaphthalen-2-yl)(thiophen-2-yl)methanone (C-38):[5]

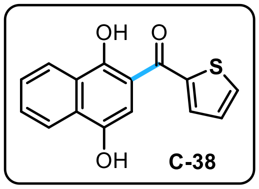

GP1 was followed using 1,4-naphthaquinone (0.3 mmol, 48 mg) and thiophene-2-carbaldehyde (50 mg, 0.45 mmol). After 24 h, purification by column chromatography using 12% ethyl acetate in PE yielded **C-38** (71 mg, 87%) as a yellow solid.

^{1}H NMR (400 MHz, DMSO-d_6) δ 13.42 (s, 1H), 12.18 (s, 1H), 9.79 (s, 1H), 8.30 (ddd, J = 8.3, 1.3, 0.7 Hz, 1H), 8.12 (dt, J = 8.1, 1.1 Hz, 1H), 7.68 (ddd, J = 8.3, 6.8, 1.3 Hz, 1H), 7.60 (ddd, J = 8.2, 6.9, 1.3 Hz, 1H), 7.51 (s, 1H), 7.29 (ddd, J = 3.5, 2.4, 1.3 Hz, 1H), 7.16 (ddd, J = 3.9, 2.5, 1.3 Hz, 1H), 6.40 (dt, J = 3.9, 2.3 Hz, 1H).

^{13}C NMR{^{1}H} (101 MHz, DMSO-d_6) 185.6, 154.1, 144.7, 129.7, 128.8, 128.5, 126.8, 126.3, 125.4, 123.4, 122.1, 119.4, 112.2, 111.0, 105.7.

(1,4-dihydroxynaphthalen-2-yl)(9-ethyl-9*H*-carbazol-3-yl)methanone (C-39):

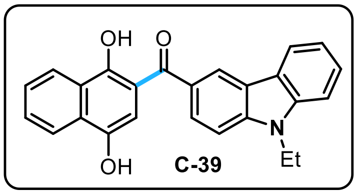

GP1 was followed using 1,4-naphthaquinone (0.3 mmol, 48 mg) and 9-ethyl-9*H*-carbazole-3-carbaldehyde (100 mg, 0.45 mmol). After 24 h, purification by column chromatography using 15% ethyl acetate in PE yielded **C-39** (70 mg, 61%) as a yellow solid.

^{1}H NMR (400 MHz, DMSO-d_6) δ 12.96 (s, 1H), 9.76 (s, 1H), 8.65 (dd, J = 1.7, 0.6 Hz, 1H), 8.37 (ddd, J = 8.3, 1.4, 0.7 Hz, 1H), 8.29 (dt, J = 7.8, 1.0 Hz, 1H), 8.16 – 8.13 (m, 1H), 7.91 (dd, J = 8.6, 1.7 Hz, 1H), 7.81 (dd, J = 8.6, 0.7 Hz, 1H), 7.75 – 7.70 (m, 2H), 7.65 (ddd, J = 8.2, 6.9, 1.3 Hz, 1H), 7.56 – 7.53 (m, 1H), 7.28 (ddd, J = 8.0, 7.1, 0.9 Hz, 1H), 7.09 (s, 1H), 4.55 (q, J = 7.1 Hz, 2H), 1.37 (t, J = 7.1 Hz, 3H).

^{13}C NMR{^{1}H} (101 MHz, DMSO-d_6) δ 199.8, 153.9, 144.5, 141.7, 140.3, 129.1, 128.8, 128.5, 127.4, 126.7, 126.4, 125.5, 123.6, 122.8, 122.3, 122.2, 121.8, 121.0, 119.8, 113.5, 109.7, 108.9, 107.4, 37.3, 13.8.

ESI-HRMS: m/z calculated for $C_{25}H_{20}NO_3$ $[M+H]^+$ calcd. 382.1438, found 382.1440.

(2,3-dihydrobenzofuran-5-yl)(1,4-dihydroxynaphthalen-2-yl)methanone (C-40):

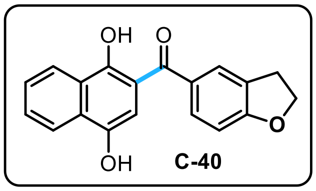

GP1 was followed using 1,4-naphthaquinone (0.3 mmol, 48 mg) and 2,3-dihydrobenzofuran-5-carbaldehyde (67 mg, 0.45 mmol). After 24 h, purification by column chromatography using 20% ethyl acetate in PE yielded **C-40** (58 mg, 63%) as a yellow solid.

^{1}H NMR (400 MHz, DMSO-d_6) δ 12.71 (s, 1H), 9.75 (s, 1H), 8.32 (ddd, J = 8.3, 1.4, 0.7 Hz, 1H), 8.13 (ddd, J = 8.3, 1.3, 0.7 Hz, 1H), 7.72 – 7.67 (m, 2H), 7.64 – 7.56 (m, 2H), 6.97 – 6.94 (m, 2H), 4.67 (t, J = 8.8 Hz, 2H), 3.28 (t, J = 8.8 Hz, 2H).

^{13}C NMR{^{1}H} (101 MHz, DMSO-d_6) δ 198.7, 163.3, 153.4, 144.5, 131.0, 130.3, 129.0, 128.7, 128.0, 126.7, 126.4, 125.4, 123.5, 122.2, 113.4, 108.7, 106.9, 72.1, 28.6.

ESI-HRMS: m/z calculated for $C_{19}H_{15}O_4$ $[M+H]^+$ calcd. 307.0965, found 307.0967.

1-(1,4-dihydroxynaphthalen-2-yl)ethan-1-one (C-41):[3]

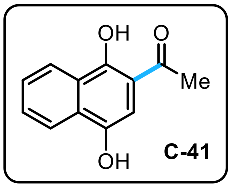

GP1 was followed using 1,4-naphthaquinone (0.3 mmol, 48 mg) and acetaldehyde (20 mg, 0.45 mmol). After 24 h, purification by column chromatography using 12% ethyl acetate in PE yielded **C-41** (57 mg, 93%) as a yellow solid.

^{1}H NMR (400 MHz, DMSO-d_6) δ 13.49 (s, 1H), 9.83 (s, 1H), 8.32 – 8.27 (m, 1H), 8.13 (dt, J = 8.4, 1.0 Hz, 1H), 7.69 (ddd, J = 8.3, 6.9, 1.3 Hz, 1H), 7.59 (ddd, J = 8.2, 6.9, 1.3 Hz, 1H), 7.09 (s, 1H), 2.65 (s, 3H).

^{13}C NMR{^{1}H} (101 MHz, DMSO-d_6) δ 204.7, 154.3, 144.7, 129.5, 129.3, 126.4, 125.1, 123.7, 122.2, 112.5, 105.1, 27.1.

For gram-scale synthesis: **GP4** was followed using 1,4-naphthaquinone (7 mmol, 1.10 g) and acetaldehyde (462 mg, 10.5 mmol). After 24 h, purification by column chromatography using 12% ethyl acetate in PE yielded **C-41** (848 mg, 60%) as a yellow solid.

1-(1,4-dihydroxynaphthalen-2-yl)propan-1-one (C-42):[3]

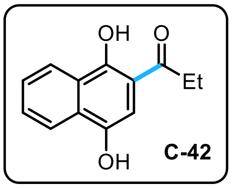

GP1 was followed using 1,4-naphthaquinone (0.3 mmol, 48 mg) and propionaldehyde (26 mg, 0.45 mmol). After 24 h, purification by column chromatography using 12% ethyl acetate in PE yielded **C-42** (60 mg, 93%) as a yellow solid.

^{1}H NMR (400 MHz, DMSO-d_6) δ 12.96 (s, 1H), 9.26 (s, 1H), 7.74 (ddd, J = 8.3, 1.3, 0.7 Hz, 1H), 7.57 (ddd, J = 8.3, 1.2, 0.7 Hz, 1H), 7.13 (ddd, J = 8.3, 6.9, 1.3 Hz, 1H), 7.03 (ddd, J = 8.2, 6.9, 1.3 Hz, 1H), 6.56 (s, 1H), 2.53 (q, J = 7.2 Hz, 2H), 0.59 (t, J = 7.2 Hz, 3H).

^{13}C NMR{^{1}H} (101 MHz, DMSO-d_6) δ 206.9, 154.1, 144.7, 129.4, 129.2, 126.4, 125.2, 123.7, 122.2, 112.0, 104.3, 31.6, 8.1.

1-(1,4-dihydroxynaphthalen-2-yl)nonan-1-one (C-43):

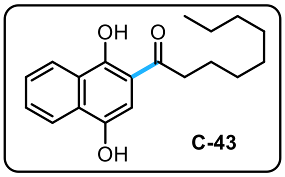

GP1 was followed using 1,4-naphthaquinone (0.3 mmol, 48 mg) and nonanal (64 mg, 0.45 mmol). After 24 h, purification by column chromatography using 12% ethyl acetate in PE yielded **C-43** (85 mg, 94%) as a yellow solid.

^{1}**H NMR** (400 MHz, $CDCl_3$) δ 13.74 (s, 1H), 9.76 (s, 1H), 8.48 – 8.40 (m, 1H), 8.12 (d, *J* = 8.3 Hz, 1H), 7.64 (ddd, *J* = 8.3, 6.8, 1.3 Hz, 1H), 7.55 (ddd, *J* = 8.2, 6.9, 1.3 Hz, 1H), 7.01 (s, 1H), 2.93 (t, *J* = 7.5 Hz, 2H), 1.77 – 1.74 (m, 2H), 1.26 (s, 10H), 0.88 (s, 3H).

^{13}**C NMR{^{1}H}** (101 MHz, $CDCl_3$) δ 206.1, 157.3, 143.5, 129.7, 129.6, 126.5, 126.3, 124.7, 121.9, 111.9, 105.4, 38.9, 34.5, 32.0, 29.6, 28.0, 26.1, 22.8, 14.2.

ESI-HRMS: m/z calculated for $C_{19}H_{25}O_3$ $[M+H]^+$ calcd. 301.1803, found 301.1798.

1-(1,4-dihydroxynaphthalen-2-yl)-3,3-dimethylbutan-1-one (C-44):[6]

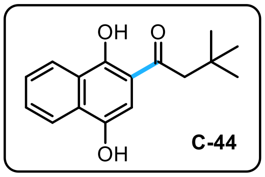

GP1 was followed using 1,4-naphthaquinone (0.3 mmol, 48 mg) and 3,3-dimethylbutanal (45 mg, 0.45 mmol). After 24 h, purification by column chromatography using 12% ethyl acetate in PE yielded **C-44** (75 mg, 97%) as a yellow solid.

^{1}**H NMR** (400 MHz, DMSO-d_6) δ 13.88 (s, 1H), 9.75 (s, 1H), 8.31 (ddd, *J* = 8.4, 1.4, 0.7 Hz, 1H), 8.15 – 8.09 (m, 1H), 7.69 (ddd, *J* = 8.4, 6.9, 1.4 Hz, 1H), 7.58 (ddd, *J* = 8.3, 6.9, 1.3 Hz, 1H), 7.18 (s, 1H), 2.90 (s, 2H), 1.06 (s, 9H).

^{13}**C NMR{^{1}H}** (101 MHz, DMSO-d_6) δ 206.5, 155.0, 144.4, 129.5, 129.4, 126.4, 125.2, 123.7, 122.1, 113.3, 105.3, 49.3, 31.5, 29.8.

5-chloro-1-(1,4-dihydroxynaphthalen-2-yl)pentan-1-one (C-45):

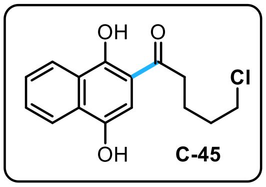

GP1 was followed using 1,4-naphthaquinone (0.3 mmol, 48 mg) and 5-chloropentanal (54 mg, 0.45 mmol). After 24 h, purification by column chromatography using 12% ethyl acetate in PE yielded **C-45** (66 mg, 79%) as a yellow solid.

^{1}**H NMR** (400 MHz, DMSO-d_6) δ 13.49 (s, 1H), 9.79 (s, 1H), 8.30 (dt, *J* = 8.2, 1.1 Hz, 1H), 8.12 (dt, *J* = 8.3, 1.0 Hz, 1H), 7.69 (ddd, *J* = 8.3, 6.9, 1.3 Hz, 1H), 7.59 (ddd, *J* = 8.3, 6.9, 1.3 Hz, 1H), 7.12 (s, 1H), 3.69 (t, *J* = 6.1 Hz, 2H), 3.10 (t, *J* = 6.7 Hz, 2H), 1.82 (ttd, *J* = 10.1, 4.5, 2.5 Hz, 4H).

^{13}**C NMR{^{1}H}** (101 MHz, DMSO-d_6) δ 206.1, 154.3, 144.7, 129.4, 129.3, 126.4, 125.2, 123.7, 122.2, 112.1, 104.4, 45.2, 37.3, 31.5, 21.1.

ESI-HRMS: m/z calculated for $C_{15}H_{16}ClO_3$ $[M+H]^+$ calcd. 279.0782, found 279.0782.

1-(1,4-dihydroxynaphthalen-2-yl)-2-methylpentan-1-one (C-46):

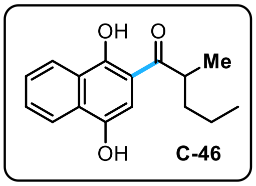

GP1 was followed using 1,4-naphthaquinone (0.3 mmol, 48 mg) and 2-methylpentanal (45 mg, 0.45 mmol). After 24 h, purification by column chromatography using 12% ethyl acetate in PE yielded **C-46** (71 mg, 91%) as a yellow solid.

^{1}H NMR (400 MHz, $CDCl_3$) δ 14.07 (s, 1H), 8.46 (ddd, *J* = 8.4, 1.3, 0.7 Hz, 1H), 8.17 – 8.11 (m, 1H), 7.66 (ddd, *J* = 8.3, 6.9, 1.3 Hz, 1H), 7.56 (ddd, *J* = 8.2, 6.9, 1.2 Hz, 1H), 7.05 (s, 1H), 6.12 (s, 1H), 3.35 (q, *J* = 6.8 Hz, 1H), 1.83 – 1.75 (m, 1H), 1.46 – 1.39 (m, 1H), 1.33 – 1.28 (m, 2H), 1.19 (d, *J* = 6.8 Hz, 3H), 0.88 (d, *J* = 7.3 Hz, 3H).

^{13}C NMR{^{1}H} (101 MHz, $CDCl_3$) δ 210.4, 158.2, 143.3, 129.9, 129.7, 126.6, 126.4, 124.7, 121.8, 111.3, 105.4, 40.2, 36.0, 20.7, 17.4, 14.3.

ESI-HRMS: m/z calculated for $C_{16}H_{18}O_3Na$ $[M+Na]^+$ calcd. 281.1148, found 281.1148.

1-(1,4-dihydroxynaphthalen-2-yl)-2-methylpropan-1-one (C-47):[6]

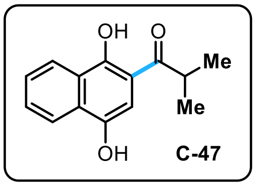

GP1 was followed using 1,4-naphthaquinone (0.3 mmol, 48 mg) and isobutyraldehyde (33 mg, 0.45 mmol). After 24 h, purification by column chromatography using 12% ethyl acetate in PE yielded **C-47** (66 mg, 95%) as a yellow solid.

^{1}H NMR (400 MHz, $CDCl_3$) δ 13.96 (s, 1H), 8.45 (ddd, *J* = 8.3, 1.4, 0.7 Hz, 1H), 8.13 (ddd, *J* = 8.3, 1.2, 0.7 Hz, 1H), 7.66 (ddd, *J* = 8.3, 6.9, 1.3 Hz, 1H), 7.56 (ddd, *J* = 8.2, 6.9, 1.3 Hz, 1H), 7.01 (s, 1H), 6.08 – 5.96 (m, 1H), 3.40 (pd, *J* = 6.8, 0.6 Hz, 1H), 1.21 (d, *J* = 6.8 Hz, 6H).

^{13}C NMR{^{1}H} (101 MHz, $CDCl_3$) δ 210.3, 158.1, 143.3, 129.9, 129.6, 126.6, 126.4, 124.6, 121.8, 110.8, 105.4, 35.3, 19.2.

1-(1,4-dihydroxynaphthalen-2-yl)-2-ethylbutan-1-one (C-48):

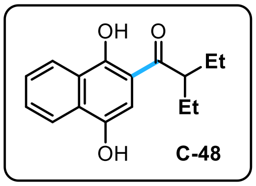

GP1 was followed using 1,4-naphthaquinone (0.3 mmol, 48 mg) and 2-ethylbutanal (45 mg, 0.45 mmol). After 24 h, purification by column chromatography using 12% ethyl acetate in PE yielded **C-48** (72 mg, 93%) as a yellow solid.

^{1}H NMR (400 MHz, DMSO-d_6) δ 13.91 (s, 1H), 9.80 (s, 1H), 8.32 (ddd, *J* = 8.4, 1.4, 0.7 Hz, 1H), 8.15 – 8.11 (m, 1H), 7.70 (ddd, *J* = 8.3, 6.9, 1.3 Hz, 1H), 7.60 (ddd, *J* = 8.3, 6.8, 1.3 Hz, 1H), 7.19 (s, 1H), 3.31 (tt,

J = 7.5, 5.5 Hz, 1H), 1.75 (dt, J = 13.6, 7.5 Hz, 2H), 1.56 (ddd, J = 13.3, 7.4, 5.6 Hz, 2H), 0.86 (t, J = 7.4 Hz, 6H).

^{13}C NMR{^{1}H} (101 MHz, DMSO-d_6) δ 210.0, 155.3, 144.8, 129.6, 129.5, 126.5, 125.3, 123.8, 122.1, 112.4, 104.2, 48.2, 24.5, 11.7.

ESI-HRMS: m/z calculated for $C_{16}H_{19}O_3$ $[M+H]^+$ calcd. 259.1329, found 259.1329.

cyclopropyl(1,4-dihydroxynaphthalen-2-yl)methanone (C-49):

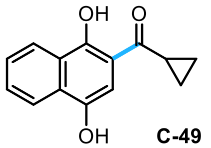

C-49

GP1 was followed using 1,4-naphthaquinone (0.3 mmol, 48 mg) and cyclopropanecarbaldehyde (32 mg, 0.45 mmol). After 24 h, purification by column chromatography using 12% ethyl acetate in PE yielded **C-49** (57 mg, 83%) as a yellow solid.

^{1}H NMR (400 MHz, DMSO-d_6) δ 13.72 (s, 1H), 9.83 (s, 1H), 8.30 (ddd, J = 8.3, 1.3, 0.7 Hz, 1H), 8.15 (ddd, J = 8.4, 1.3, 0.7 Hz, 1H), 7.70 (ddd, J = 8.3, 6.9, 1.3 Hz, 1H), 7.59 (ddd, J = 8.2, 6.9, 1.3 Hz, 1H), 7.37 (s, 1H), 2.77 (tt, J = 7.4, 5.0 Hz, 1H), 1.21 – 1.16 (m, 4H).

^{13}C NMR{^{1}H} (101 MHz, DMSO-d_6) δ 205.2, 154.2, 144.9, 129.5, 129.3, 126.4, 125.2, 123.8, 122.2, 112.6, 104.5, 16.7, 12.1.

ESI-HRMS: m/z calculated for $C_{14}H_{13}O_3$ $[M+H]^+$ calcd. 229.0865, found 229.0859.

cyclobutyl(1,4-dihydroxynaphthalen-2-yl)methanone (C-50):

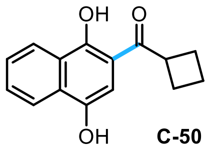

C-50

GP1 was followed using 1,4-naphthaquinone (0.3 mmol, 48 mg) and cyclobutanecarbaldehyde (38 mg, 0.45 mmol). After 24 h, purification by column chromatography using 12% ethyl acetate in PE yielded **C-50** (61 mg, 84%) as a yellow solid.

^{1}H NMR (400 MHz, DMSO-d_6) δ 13.51 (s, 1H), 9.77 (s, 1H), 8.29 (ddd, J = 8.3, 1.4, 0.7 Hz, 1H), 8.11 (ddd, J = 8.4, 1.3, 0.7 Hz, 1H), 7.68 (ddd, J = 8.3, 6.9, 1.3 Hz, 1H), 7.58 (ddd, J = 8.2, 6.9, 1.3 Hz, 1H), 6.96 (s, 1H), 4.15 – 4.04 (m, 1H), 2.40 – 2.28 (m, 4H), 2.10 – 2.02 (m, 1H), 1.87 – 1.78 (m, 1H).

^{13}C NMR{^{1}H} (101 MHz, DMSO-d_6) δ 207.2, 155.1, 145.1, 129.8, 129.7, 126.8, 125.7, 124.1, 122.6, 111.2, 104.7, 42.4, 25.1, 18.0.

ESI-HRMS: m/z calculated for $C_{14}H_{15}O_3Na$ $[M+Na]^+$ calcd. 265.0835, found 265.0840.

1-(1,4-dihydroxynaphthalen-2-yl)-2,2-dimethylpropan-1-one (C-51):[6]

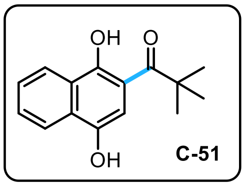

GP1 was followed using 1,4-naphthaquinone (0.3 mmol, 48 mg) and pivalaldehyde (39 mg, 0.45 mmol). After 24 h, purification by column chromatography using 12% ethyl acetate in PE yielded **C-51** (60 mg, 82%) as a yellow solid.

^{1}H NMR (400 MHz, $CDCl_3$) δ 14.31 (s, 1H), 8.48 (ddd, J = 8.3, 1.3, 0.7 Hz, 1H), 8.08 (ddd, J = 8.4, 1.3, 0.7 Hz, 1H), 7.66 (ddd, J = 8.3, 6.9, 1.3 Hz, 1H), 7.55 (ddd, J = 8.2, 6.9, 1.3 Hz, 1H), 7.33 (s, 1H), 5.29 (s, 1H), 1.46 (s, 9H).

^{13}C NMR{^{1}H} (101 MHz, $CDCl_3$) δ 211.3, 159.2, 141.7, 129.9, 129.0, 126.8, 126.5, 124.9, 121.4, 110.2, 107.1, 44.4, 28.6.

bicyclo[2.2.1]hept-5-en-2-yl(1,4-dihydroxynaphthalen-2-yl)methanone (C-52):

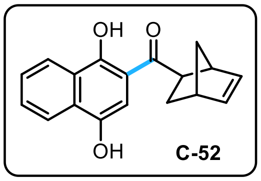

GP1 was followed using 1,4-naphthaquinone (0.3 mmol, 48 mg) and bicyclo[2.2.1]hept-5-ene-2-carbaldehyde (55 mg, 0.45 mmol). After 24 h, purification by column chromatography using 12% ethyl acetate in PE yielded **C-52** (57 mg, 68%) as a yellow solid.

^{1}H NMR (400 MHz, $CDCl_3$) δ 13.74 (s, 1H), 8.46 – 8.41 (m, 1H), 8.12 (dt, J = 8.4, 1.0 Hz, 1H), 7.65 (ddd, J = 8.3, 6.9, 1.3 Hz, 1H), 7.55 (ddd, J = 8.2, 6.9, 1.3 Hz, 1H), 7.21 (s, 1H), 6.23 (dd, J = 5.6, 3.1 Hz, 1H), 5.89 (s, 1H), 5.85 (dd, J = 5.6, 2.9 Hz, 1H), 3.82 (ddd, J = 9.1, 4.5, 3.4 Hz, 1H), 3.33 (dh, J = 3.3, 1.5 Hz, 1H), 2.98 (dq, J = 3.4, 1.7 Hz, 1H), 1.99 – 1.94 (m, 1H), 1.68 – 1.65 (m, 1H), 1.50 – 1.46 (m, 2H).

^{13}C NMR{^{1}H} (101 MHz, $CDCl_3$) δ 206.3, 143.2, 140.2, 137.5, 131.9, 129.6, 129.4, 126.5, 126.3, 124.7, 121.7, 112.0, 105.8, 50.3, 47.9, 47.4, 43.2, 29.3.

ESI-HRMS: m/z calculated for $C_{18}H_{16}O_3Na$ $[M+Na]^+$ calcd. 303.0992, found 303.0995.

tert-butyl 4-(1,4-dihydroxy-2-naphthoyl)piperidine-1-carboxylate (C-53):

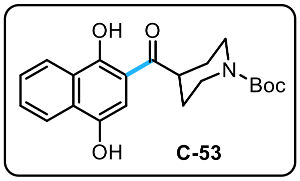

GP1 was followed using 1,4-naphthaquinone (0.3 mmol, 48 mg) and tert-butyl 4-formylpiperidine-1-carboxylate (96 mg, 0.45 mmol). After 24 h, purification by column chromatography using 25% ethyl acetate in PE yielded **C-53** (85 mg, 76%) as a yellow solid.

^{1}H NMR (400 MHz, DMSO-d_6) δ 13.59 (s, 1H), 9.81 (s, 1H), 8.33 – 8.28 (m, 1H), 8.12 (dt, J = 8.3, 1.1 Hz, 1H), 7.71 (ddd, J = 8.3, 6.9, 1.3 Hz, 1H), 7.61 (ddd, J = 8.2, 6.9, 1.3 Hz, 1H), 7.19 (s, 1H), 4.10 – 4.00 (m, 2H), 3.55 (tt, J = 11.5, 3.6 Hz, 1H), 1.87 (d, J = 13.2 Hz, 2H), 1.58 – 1.50 (m, 2H), 1.42 (s, 9H), 1.38 (dd, J = 2.5, 1.7 Hz, 1H), 1.23 – 1.14 (m, 1H).

^{13}C NMR{^{1}H} (101 MHz, DMSO-d_6) δ 207.9, 155.3, 153.8, 144.8, 129.6, 129.5, 126.5, 125.3, 123.7, 122.2, 111.0, 104.2, 78.8, 42.8, 40.1, 28.1, 28.1.

ESI-HRMS: m/z calculated for $C_{21}H_{25}NO_5Na$ [M+Na]$^+$ calcd. 394.1625, found 394.1622.

(*E*)-1-(1,4-dihydroxynaphthalen-2-yl)-3-phenylprop-2-en-1-one (C-54):

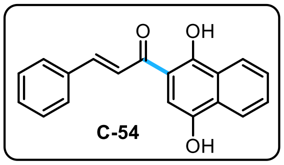

GP1 was followed using 1,4-naphthaquinone (0.3 mmol, 48 mg) and (*E*)-cinnamaldehyde (60 mg, 0.45 mmol). After 24 h, purification by column chromatography using 12% ethyl acetate in PE yielded **C-54** (50 mg, 57%) as a yellow solid.

^{1}H NMR (400 MHz, $CDCl_3$) δ 8.16 – 8.13 (m, 1H), 8.00 (d, J = 16.0 Hz, 1H), 7.86 – 7.82 (m, 1H), 7.67 – 7.62 (m, 2H), 7.54 – 7.49 (m, 2H), 7.46 (ddd, J = 6.4, 2.8, 1.4 Hz, 3H), 7.09 (d, J = 8.1 Hz, 1H), 6.80 (d, J = 16.0 Hz, 1H), 6.68 (d, J = 8.1 Hz, 1H), 5.82 (s, 1H).

^{13}C NMR{^{1}H} (101 MHz, $CDCl_3$) δ 199.8, 166.4, 149.8, 147.2, 140.2, 134.3, 131.0, 129.2, 128.6, 127.1, 125.8, 125.3, 122.4, 121.3, 118.1, 117.2, 108.0.

ESI-HRMS: m/z calculated for $C_{19}H_{15}O_3$ [M+H]$^+$ calcd. 291.1016, found 291.1017.

(*E*)-1-(1,4-dihydroxynaphthalen-2-yl)-2-phenylpent-2-en-1-one (C-55):

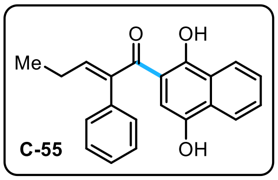

GP1 was followed using 1,4-naphthaquinone (0.3 mmol, 48 mg) and (*E*)-2-phenylpent-2-enal (72 mg, 0.45 mmol). After 24 h, purification by column chromatography using 12% ethyl acetate in PE yielded **C-55** (74 mg, 78%) as a yellow solid.

^{1}H NMR (400 MHz, $CDCl_3$) δ 13.62 (s, 1H), 8.46 (ddd, J = 8.3, 1.4, 0.7 Hz, 1H), 8.10 – 8.06 (m, 1H), 7.66 (ddd, J = 8.3, 6.9, 1.4 Hz, 1H), 7.56 (ddd, J = 8.2, 6.9, 1.2 Hz, 1H), 7.36 – 7.28 (m, 5H), 6.96 (s, 1H), 6.21 (t, J = 7.5 Hz, 1H), 5.44 (s, 1H), 2.32 (p, J = 7.5 Hz, 2H), 1.09 (t, J = 7.5 Hz, 3H).

^{13}C NMR{^{1}H} (101 MHz, $CDCl_3$) δ 201.9, 158.9, 142.6, 141.0, 139.8, 135.9, 130.0, 129.6, 129.2, 128.7, 128.0, 126.5, 126.2, 124.6, 121.8, 111.8, 108.0, 22.7, 13.9.

ESI-HRMS: m/z calculated for $C_{21}H_{19}O_3$ [M+H]$^+$ calcd. 319.1329, found 319.1328.

3-(4-(*tert*-butyl)phenyl)-1-(1,4-dihydroxynaphthalen-2-yl)-2-methylpropan-1-one (C-56):

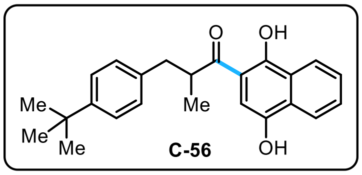

GP1 was followed using 1,4-naphthaquinone (0.3 mmol, 48 mg) and 3-(4-(*tert*-butyl)phenyl)-2-methylpropanal (92 mg, 0.45 mmol). After 24 h, purification by column chromatography using 12% ethyl acetate in PE yielded **C-56** (89 mg, 80%) as a yellow solid.

^{1}H NMR (400 MHz, $CDCl_3$) δ 13.85 (s, 1H), 8.32 (ddd, *J* = 8.3, 1.3, 0.7 Hz, 1H), 7.97 (dt, *J* = 8.3, 1.0 Hz, 1H), 7.52 (ddd, *J* = 8.3, 6.9, 1.4 Hz, 1H), 7.42 (ddd, *J* = 8.2, 6.9, 1.3 Hz, 1H), 7.14 – 7.11 (m, 2H), 7.01 – 6.97 (m, 2H), 6.87 (s, 1H), 3.54 (h, *J* = 6.9 Hz, 1H), 3.04 (dd, *J* = 13.8, 6.3 Hz, 1H), 2.58 (dd, *J* = 13.8, 7.7 Hz, 1H), 1.14 (s, 9H), 1.11 (d, *J* = 6.9 Hz, 3H).

^{13}C NMR{^{1}H} (101 MHz, $CDCl_3$) δ 209.2, 158.2, 149.3, 143.2, 136.5, 129.9, 129.6, 128.8, 126.6, 126.3, 125.4, 124.7, 121.7, 111.2, 105.3, 42.5, 38.9, 34.5, 31.5, 17.7.

ESI-HRMS: m/z calculated for $C_{24}H_{27}O_3$ $[M+H]^+$ calcd. 363.1955, found 363.1958.

(*E*)-1-(1,4-dihydroxynaphthalen-2-yl)-5-methyl-2-phenylhex-2-en-1-one (C-57):

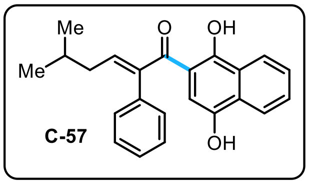

GP1 was followed using 1,4-naphthaquinone (0.3 mmol, 48 mg) and (*E*)-5-methyl-2-phenylhex-2-enal (85 mg, 0.45 mmol). After 24 h, purification by column chromatography using 12% ethyl acetate in PE yielded **C-57** (63 mg, 60%) as a yellow solid.

^{1}H NMR (400 MHz, $CDCl_3$) δ 13.61 (s, 1H), 8.46 (ddd, *J* = 8.3, 1.3, 0.7 Hz, 1H), 8.08 (dt, *J* = 8.2, 1.0 Hz, 1H), 7.66 (ddd, *J* = 8.3, 6.9, 1.3 Hz, 1H), 7.56 (ddd, *J* = 8.2, 6.9, 1.3 Hz, 1H), 7.36 – 7.30 (m, 5H), 6.98 (s, 1H), 6.25 (t, *J* = 7.4 Hz, 1H), 5.54 (s, 1H), 2.22 – 2.18 (m, 2H), 1.80 – 1.74 (m, 1H), 0.93 (d, *J* = 6.7 Hz, 6H).

^{13}C NMR{^{1}H} (101 MHz, $CDCl_3$) δ 201.9, 158.8, 142.7, 140.9, 138.7, 136.0, 130.0, 129.6, 129.3, 128.6, 127.9, 126.5, 124.6, 121.8, 111.8, 108.0, 68.5, 38.0, 28.9, 22.7, 22.5.

ESI-HRMS: m/z calculated for $C_{23}H_{23}O_3$ $[M+H]^+$ calcd. 347.1642, found 347.1646.

1-(1,4-dihydroxynaphthalen-2-yl)-3-(4-isopropylphenyl)-2-methylpropan-1-one (C-58):

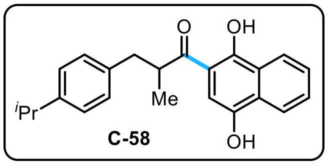

GP1 was followed using 1,4-naphthaquinone (0.3 mmol, 48 mg) and 3-(4-isopropylphenyl)-2-methylpropanal (86 mg, 0.45 mmol). After 24 h, purification by column chromatography using 12% ethyl acetate in PE yielded **C-58** (84 mg, 80%) as a yellow solid.

^{1}H NMR (400 MHz, $CDCl_3$) δ 14.00 (s, 1H), 8.46 (ddd, *J* = 8.3, 1.4, 0.7 Hz, 1H), 8.11 (ddd, *J* = 8.3, 1.2, 0.7 Hz, 1H), 7.66 (ddd, *J* = 8.3, 6.9, 1.3 Hz, 1H), 7.56 (ddd, *J* = 8.3, 6.9, 1.3 Hz, 1H), 7.12 (d, *J* = 1.5 Hz, 4H), 7.00 (s, 1H), 5.63 (s, 1H), 3.72 – 3.64 (m, 1H), 3.19 (dd, *J* = 13.8, 6.3 Hz, 1H), 2.88 – 2.82 (m, 1H), 2.72 (dd, *J* = 13.8, 7.7 Hz, 1H), 1.25 (d, *J* = 6.9 Hz, 3H), 1.22 (d, *J* = 0.9 Hz, 3H), 1.20 (d, *J* = 0.9 Hz, 3H).

^{13}C NMR{^{1}H} (101 MHz, $CDCl_3$) δ 209.2, 158.2, 147.0, 143.2, 136.9, 129.9, 129.6, 129.1, 126.6, 126.6, 126.3, 124.7, 121.7, 111.2, 105.3, 42.5, 39.0, 33.8, 24.1, 17.7.

ESI-HRMS: m/z calculated for $C_{23}H_{25}O_3$ $[M+H]^+$ calcd. 349.1798, found 349.1796.

(*S*)-(1,4-dihydroxynaphthalen-2-yl)(4-(prop-1-en-2-yl)cyclohex-1-en-1-yl)methanone (C-59):

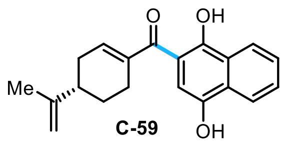

C-59

GP1 was followed using 1,4-naphthaquinone (0.3 mmol, 48 mg) and (*S*)-4-(prop-1-en-2-yl)cyclohex-1-ene-1-carbaldehyde (67 mg, 0.45 mmol). After 24 h, purification by column chromatography using 12% ethyl acetate in PE yielded **C-59** (84 mg, 72%) as a yellow solid.

^{1}H NMR (400 MHz, $CDCl_3$) δ 13.41 (s, 1H), 8.51 – 8.41 (m, 1H), 8.14 – 8.09 (m, 1H), 7.67 (ddd, *J* = 8.3, 6.9, 1.4 Hz, 1H), 7.56 (ddd, *J* = 8.2, 6.9, 1.3 Hz, 1H), 7.04 (s, 1H), 6.42 – 6.31 (m, 1H), 5.38 (s, 1H), 4.81 – 4.74 (m, 2H), 2.63 – 2.53 (m, 1H), 2.49 – 2.36 (m, 2H), 2.29 – 2.18 (m, 2H), 2.03 – 1.88 (m, 2H), 1.76 – 1.72 (m, 1H), 1.59 (ddd, *J* = 11.1, 5.5, 1.9 Hz, 1H), 1.29 (t, *J* = 3.5 Hz, 1H).

^{13}C NMR{^{1}H} (101 MHz, $CDCl_3$) δ 202.3, 158.1, 148.9, 142.5, 137.4, 137.1, 129.8, 129.4, 126.5, 126.3, 124.6, 121.7, 111.3, 109.5, 108.2, 40.3, 31.0, 27.1, 25.6, 20.9.

ESI-HRMS: m/z calculated for $C_{20}H_{21}O_3$ $[M+H]^+$ calcd. 309.1485, found 309.1492.

(1,4-dihydroxynaphthalen-2-yl)(2,6,6-trimethylcyclohex-1-en-1-yl)methanone (C-60):

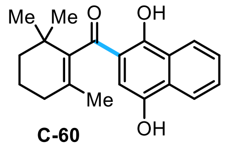

C-60

GP1 was followed using 1,4-naphthaquinone (0.3 mmol, 48 mg) and 2,6,6-trimethylcyclohex-1-ene-1-carbaldehyde (69 mg, 0.45 mmol). After 24 h, purification by column chromatography using 12% ethyl acetate in PE yielded **C-60** (62 mg, 66%) as a yellow solid.

^{1}H NMR (400 MHz, $CDCl_3$ + DMSO-d_6) δ 14.08 (s, 1H), 9.36 (s, 1H), 8.66 (t, *J* = 7.5 Hz, 1H), 8.42 (t, *J* = 7.5 Hz, 1H), 7.92 (d, *J* = 3.9 Hz, 1H), 7.81 (t, *J* = 7.4 Hz, 1H), 7.28 (s, 1H), 2.43 – 2.31 (m, 2H), 1.78 (s, 3H), 1.49 (d, *J* = 6.7 Hz, 4H), 1.40 (s, 3H), 1.25 (s, 3H).

^{13}C NMR{^{1}H} (101 MHz, $CDCl_3$ + DMSO-d_6) δ 206.4, 155.4, 143.9, 137.3, 130.7, 129.5, 128.5, 125.2, 125.0, 123.3, 121.5, 113.3, 106.6, 37.9, 33.3, 30.3, 27.7, 20.8, 18.1.

ESI-HRMS: m/z calculated for $C_{20}H_{23}O_3$ $[M+H]^+$ calcd. 311.1642, found 311.1643.

(1,4-dihydroxynaphthalen-2-yl)(6,6-dimethylbicyclo[3.1.1]hept-2-en-2-yl)methanone (C-61):

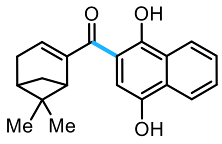

C-61

GP1 was followed using 1,4-naphthaquinone (0.3 mmol, 48 mg) and 6,6-dimethylbicyclo[3.1.1]hept-2-ene-2-carbaldehyde (68 mg, 0.45 mmol). After 24 h, purification by column chromatography using 12% ethyl acetate in PE yielded **C-61** (69 mg, 75%) as a yellow solid.

^{1}H NMR (400 MHz, $CDCl_3$) δ 13.41 (s, 1H), 8.46 (d, *J* = 8.3 Hz, 1H), 8.11 (d, *J* = 8.4 Hz, 1H), 7.67 (ddd, *J* = 8.2, 6.9, 1.3 Hz, 1H), 7.57 (ddd, *J* = 8.2, 6.8, 1.2 Hz, 1H), 7.09 (s, 1H), 6.35 (t, *J* = 1.6 Hz, 1H), 5.06 (s, 1H), 2.81 (td, *J* = 5.6, 1.5 Hz, 1H), 2.65 – 2.55 (m, 2H), 2.52 – 2.46 (m, 1H), 2.21 (td, *J* = 6.8, 3.1 Hz, 1H), 1.39 (s, 3H), 1.34 (d, *J* = 9.2 Hz, 1H), 0.94 (s, 3H).

^{13}C NMR{^{1}H} (101 MHz, $CDCl_3$) δ 199.6, 157.8, 147.8, 142.4, 136.0, 129.7, 129.2, 126.5, 126.3, 124.6, 121.6, 111.3, 108.1, 43.6, 40.4, 38.0, 32.6, 31.6, 26.0, 21.3.

ESI-HRMS: m/z calculated for $C_{20}H_{21}O_3$ $[M+H]^+$ calcd. 309.1485, found 309.1482.

(1,4-dihydroxynaphthalen-2-yl)(3,4-dimethoxyphenyl)methanone (C-62):

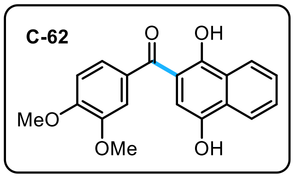

GP1 was followed using 1,4-naphthaquinone (0.3 mmol, 48 mg) 3,4-dimethoxybenzaldehyde (75 mg, 0.45 mmol). After 24 h, purification by column chromatography using 20% ethyl acetate in PE yielded **C-62** (81 mg, 83%) as a yellow solid.

^{1}H NMR (400 MHz, DMSO-d_6) δ 12.83 (s, 1H), 9.77 (s, 1H), 8.34 (ddd, *J* = 8.3, 1.4, 0.7 Hz, 1H), 8.16 – 8.10 (m, 1H), 7.71 (ddd, *J* = 8.3, 6.9, 1.4 Hz, 1H), 7.63 (ddd, *J* = 8.2, 6.8, 1.3 Hz, 1H), 7.41 – 7.32 (m, 2H), 7.16 (d, *J* = 8.2 Hz, 1H), 7.02 (s, 1H), 3.88 (s, 3H), 3.85 (s, 3H).

^{13}C NMR{^{1}H} (101 MHz, DMSO-d_6) δ 198.7, 153.9, 152.2, 148.4, 144.5, 130.0, 129.1, 128.8, 126.4, 125.4, 123.6, 123.6, 122.2, 113.1, 112.2, 110.9, 106.8, 55.8, 55.6.

ESI-HRMS: m/z calculated for $C_{19}H_{17}O_5$ $[M+H]^+$ calcd. 325.1071, found 325.1071.

1-(1,4-dihydroxynaphthalen-2-yl)-6-methoxy-2,6-dimethylheptan-1-one (C-63):

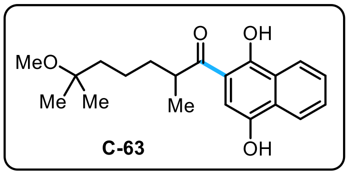

GP1 was followed using 1,4-naphthaquinone (0.3 mmol, 48 mg) 6-methoxy-2,6-dimethylheptanal (78 mg, 0.45 mmol). After 24 h, purification by column chromatography using 18% ethyl acetate in PE yielded **C-63** (93 mg, 94%) as a yellow solid.

^{1}H NMR (400 MHz, DMSO-d_6) δ 13.79 (s, 1H), 9.81 (s, 1H), 8.31 (ddd, *J* = 8.4, 1.3, 0.7 Hz, 1H), 8.13 (ddd, *J* = 8.4, 1.2, 0.7 Hz, 1H), 7.70 (ddd, *J* = 8.3, 6.9, 1.3 Hz, 1H), 7.59 (ddd, *J* = 8.3, 6.9, 1.3 Hz, 1H), 7.17 (s, 1H), 3.51 (q, *J* = 6.7 Hz, 1H), 3.00 (s, 3H), 1.76 (ddt, *J* = 13.3, 8.3, 6.4 Hz, 1H), 1.46 – 1.24 (m, 6H), 1.18 (d, *J* = 6.8 Hz, 3H), 1.01 (s, 6H).

^{13}C NMR{^{1}H} (101 MHz, DMSO-d_6) δ 210.1, 155.3, 144.8, 129.5, 129.4, 126.4, 125.4, 123.7, 122.1, 111.4, 104.1, 73.7, 48.4, 39.9, 33.8, 24.7, 24.6, 21.1, 17.1.

ESI-HRMS: m/z calculated for $C_{20}H_{26}O_4Na$ $[M+Na]^+$ calcd. 353.1723, found 353.1722.

(1,4-dihydroxynaphthalen-2-yl)(4-isopropylphenyl)methanone (C-64):

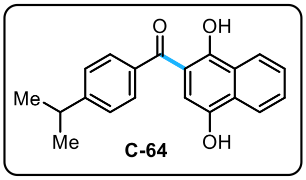

GP1 was followed using 1,4-naphthaquinone (0.3 mmol, 48 mg) 4-isopropylbenzaldehyde (67 mg, 0.45 mmol). After 24 h, purification by column chromatography using 10% ethyl acetate in PE yielded **C-64** (83 mg, 90%) as a yellow solid.

^{1}H NMR (400 MHz, $CDCl_3$) δ 13.57 (s, 1H), 8.51 (dt, *J* = 8.2, 1.0 Hz, 1H), 8.13 (dt, *J* = 8.3, 1.0 Hz, 1H), 7.68 (ddd, *J* = 8.3, 6.9, 1.4 Hz, 1H), 7.63 – 7.55 (m, 3H), 7.30 (dd, *J* = 8.2, 1.8 Hz, 2H), 6.93 (s, 1H), 5.70 – 5.43 (m, 1H), 2.94 (hep, *J* = 6.9 Hz, 1H), 1.26 (d, *J* = 6.9 Hz, 6H).

^{13}C NMR{^{1}H} (101 MHz, $CDCl_3$) δ 200.8, 158.6, 153.2, 142.8, 135.9, 130.0, 129.5, 129.4, 126.6, 126.5, 126.2, 124.7, 121.9, 111.7, 108.4, 34.3, 23.8.

ESI-HRMS: m/z calculated for $C_{20}H_{19}O_3$ $[M+H]^+$ calcd. 307.1328, found 307.1335.

(1,4-dihydroxynaphthalen-2-yl)(3,4,5-trimethoxyphenyl)methanone (C-65):[5]

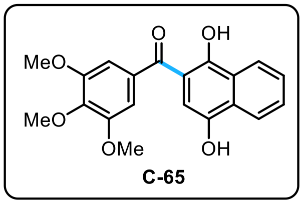

GP1 was followed using 1,4-naphthaquinone (0.3 mmol, 48 mg) 3,4,5-trimethoxybenzaldehyde (88 mg, 0.45 mmol). After 24 h, purification by column chromatography using 26% ethyl acetate in PE yielded **C-65** (85 mg, 80%) as a yellow solid.

^{1}H NMR (400 MHz, DMSO-*d*$_6$) δ 13.04 (s, 1H), 9.79 (s, 1H), 8.35 (ddd, *J* = 8.4, 1.3, 0.7 Hz, 1H), 8.12 (dt, *J* = 8.1, 1.0 Hz, 1H), 7.73 (ddd, *J* = 8.3, 6.9, 1.3 Hz, 1H), 7.64 (ddd, *J* = 8.2, 6.9, 1.3 Hz, 1H), 7.05 (s, 2H), 7.04 (s, 1H), 3.86 (s, 6H), 3.79 (s, 3H).

^{13}C NMR{^{1}H} (101 MHz, DMSO-*d*$_6$) δ 199.4, 154.9, 152.6, 144.5, 140.5, 132.9, 129.5, 129.1, 126.6, 125.3, 123.7, 122.2, 112.4, 106.7, 106.6, 60.2, 56.1.

ESI-HRMS: m/z calculated for $C_{20}H_{19}O_6$ $[M+H]^+$ calcd. 355.1176, found 355.1175.

Benzo[d][1,3]dioxol-5-yl(1,4-dihydroxynaphthalen-2-yl)methanone (C-66):

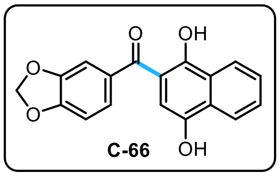

GP1 was followed using 1,4-naphthaquinone (0.3 mmol, 48 mg) benzo[d][1,3]dioxole-5-carbaldehyde (68 mg, 0.45 mmol). After 24 h, purification by column chromatography using 22% ethyl acetate in PE yielded **C-67** (57 mg, 62%) as a yellow solid.

^{1}H NMR (400 MHz, DMSO-*d*$_6$) δ 12.66 (s, 1H), 9.78 (s, 1H), 8.33 (ddd, *J* = 8.3, 1.4, 0.7 Hz, 1H), 8.12 (ddd, *J* = 8.3, 1.3, 0.7 Hz, 1H), 8.04 – 7.97 (m, 1H), 7.72 – 7.68 (m, 1H), 7.65 – 7.60 (m, 1H), 7.31 (dd, *J* = 2.2, 1.7 Hz, 1H), 7.12 (dd, *J* = 7.9, 0.5 Hz, 1H), 6.94 (s, 1H), 6.18 (s, 2H).

^{13}C NMR{^{1}H} (101 MHz, DMSO-d_6) δ 198.3, 153.7, 150.6, 147.4, 144.6, 131.8, 129.2, 128.9, 126.5, 125.4, 125.2, 123.6, 122.2, 113.2, 109.0, 108.1, 106.7, 102.0.

ESI-HRMS: m/z calculated for $C_{18}H_{13}O_5$ $[M+H]^+$ calcd. 309.0750, found 309.0740.

1-(2,5-dihydroxyphenyl)-3-methylbut-3-en-1-one (C-67):

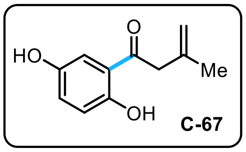

C-67

GP1 was followed using *p*-benzoquinone (0.3 mmol, 33 mg) 3-methylbut-3-enal (38 mg, 0.45 mmol). After 24 h, purification by column chromatography using 12% ethyl acetate in PE yielded **C-67** (40 mg, 69%) as a yellow solid.

^{1}H NMR (400 MHz, $CDCl_3$) δ 11.43 (s, 1H), 7.21 (d, *J* = 3.1 Hz, 1H), 7.03 (dd, *J* = 8.9, 3.1 Hz, 1H), 7.00 – 6.93 (m, 1H), 6.91 (d, *J* = 8.9 Hz, 1H), 6.85 – 6.74 (m, 1H), 5.70 (dt, *J* = 1.6, 0.8 Hz, 1H), 5.47 – 5.42 (m, 1H), 4.97 (s, 1H), 2.07 (dd, *J* = 1.6, 1.0 Hz, 3H).

^{13}C NMR{^{1}H} (101 MHz, $CDCl_3$) δ 203.1, 157.2, 147.3, 142.7, 124.9, 123.2, 119.3, 117.8, 116.1, 93.1, 19.6.

ESI-HRMS: m/z calculated for $C_{11}H_{13}O_3$ $[M+H]^+$ calcd. 193.0985, found 193.1199.

1-(2,5-dihydroxyphenyl)-3,7-dimethyloct-6-en-1-one (C-68):

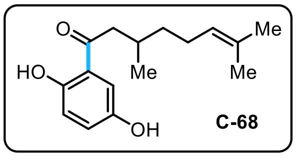

C-68

GP1 was followed using *p*-benzoquinone (0.3 mmol, 33 mg) 3,7-dimethyloct-6-enal (70 mg, 0.45 mmol). After 24 h, purification by column chromatography using 15% ethyl acetate in PE yielded **C-68** (56 mg, 71%) as a yellow solid.

^{1}H NMR (400 MHz, DMSO-d_6) δ 11.36 (s, 1H), 9.16 (s, 1H), 7.18 (d, *J* = 3.0 Hz, 1H), 6.98 (dd, *J* = 8.9, 2.9 Hz, 1H), 6.79 (d, *J* = 8.9 Hz, 1H), 5.07 (tt, *J* = 5.6, 2.8 Hz, 1H), 2.95 (d, *J* = 5.7 Hz, 1H), 2.78 (dd, *J* = 15.9, 7.9 Hz, 1H), 2.06 – 1.93 (m, 3H), 1.63 (d, *J* = 1.4 Hz, 3H), 1.55 (d, *J* = 1.4 Hz, 3H), 1.37 – 1.32 (m, 1H), 1.23 (dt, *J* = 4.5, 1.9 Hz, 1H), 0.90 (d, *J* = 6.6 Hz, 3H).

^{13}C NMR{^{1}H} (101 MHz, DMSO-d_6) δ 243.5, 191.3, 187.0, 168.4, 162.0, 161.8, 158.0, 156.0, 152.5, 83.6, 74.1, 66.8, 63.1, 62.6, 57.2, 55.2.

ESI-HRMS: m/z calculated for $C_{16}H_{23}O_3$ $[M+H]^+$ calcd. 263.1642, found 263.1642.

1-(2,5-dihydroxyphenyl)-2,6-dimethylhept-5-en-1-one (C-69):

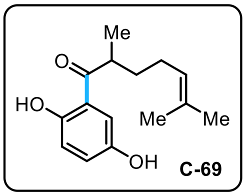

C-69

GP1 was followed using *p*-benzoquinone (0.3 mmol, 33 mg) 2,6-dimethylhept-5-enal (63 mg, 0.45 mmol). After 24 h, purification by column chromatography using 12% ethyl acetate in PE yielded **C-69** (54 mg, 72%) as a yellow solid.

^{1}H NMR (400 MHz, DMSO-d_6) δ 11.41 (s, 1H), 9.17 (s, 1H), 7.17 (d, *J* = 3.0 Hz, 1H), 6.99 (dd, *J* = 8.9, 2.9 Hz, 1H), 6.81 (d, *J* = 8.9 Hz, 1H), 5.06 (tdd, *J* = 5.8, 2.9, 1.4 Hz, 1H), 3.51 (h, *J*

= 6.8 Hz, 1H), 1.93 (t, J = 7.4 Hz, 2H), 1.78 – 1.72 (m, 1H), 1.60 (d, J = 1.3 Hz, 3H), 1.46 (d, J = 1.3 Hz, 3H), 1.39 (dt, J = 7.6, 6.3 Hz, 1H), 1.11 (d, J = 6.8 Hz, 3H).

^{13}C NMR{^{1}H} (101 MHz, DMSO-d_6) δ 209.6, 154.0, 149.4, 131.5, 124.2, 123.9, 119.5, 118.5, 114.5, 33.4, 25.4, 25.3, 17.4, 17.2.

ESI-HRMS: m/z calculated for $C_{15}H_{21}O_3$ $[M+H]^+$ calcd. 249.1485, found 249.1483.

(*E*)-2-benzylidene-1-(2,5-dihydroxyphenyl)heptan-1-one (C-70):

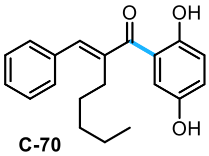

C-70

GP1 was followed using *p*-benzoquinone (0.3 mmol, 33 mg) (*E*)-2-benzylideneheptanal (91 mg, 0.45 mmol). After 24 h, purification by column chromatography using 12% ethyl acetate in PE yielded **C-70** (63 mg, 68%) as a yellow solid.

^{1}H NMR (400 MHz, $CDCl_3$) δ 7.44 (d, J = 4.3 Hz, 2H), 7.37 – 7.35 (m, 3H), 7.00 – 6.96 (m, 1H), 6.76 (dt, J = 8.6, 2.0 Hz, 3H), 6.73 – 6.68 (m, 2H), 2.58 – 2.54 (m, 2H), 1.70 – 1.62 (m, 3H), 1.43 – 1.39 (m, 3H), 0.96 – 0.92 (m, 3H).

^{13}C NMR{^{1}H} (101 MHz, $CDCl_3$) δ 211.0, 169.6, 168.4, 153.8, 143.7, 136.3, 134.3, 129.5, 128.7, 128.4, 122.5, 122.1, 116.1, 35.6, 31.3, 28.1, 22.6, 14.1.

ESI-HRMS: m/z calculated for $C_{20}H_{23}O_3$ $[M+H]^+$ calcd. 311.1642, found 311.1645.

1-(2,5-dihydroxyphenyl)-2-(2,6,6-trimethylcyclohex-1-en-1-yl)ethan-1-one (C-71):

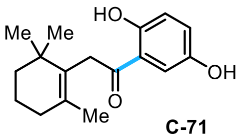

C-71

GP1 was followed using *p*-benzoquinone (0.3 mmol, 33 mg) 2-(2,6,6-trimethylcyclohex-1-en-1-yl)acetaldehyde (75 mg, 0.45 mmol). After 24 h, purification by column chromatography using 15% ethyl acetate in PE yielded **C-71** (62 mg, 75%) as a yellow solid.

^{1}H NMR (400 MHz, $CDCl_3$) 11.93 (s, 1H), 7.37 (d, J = 3.0 Hz, 1H), 7.04 (dd, J = 8.9, 3.0 Hz, 1H), 6.89 (d, J = 8.9 Hz, 1H), 5.14 (s, 1H), 3.72 (s, 2H), 2.09 – 2.03 (m, 2H), 1.69 – 1.62 (m, 2H), 1.54 – 1.50 (m, 2H), 1.49 (s, 3H), 0.93 (s, 6H).

^{13}C NMR{^{1}H} (101 MHz, $CDCl_3$) 204.1, 156.7, 147.6, 132.1, 129.8, 124.8, 119.5, 119.2, 114.7, 39.2, 37.8, 34.7, 32.6, 28.2, 20.6, 19.5.

ESI-HRMS: m/z calculated for $C_{17}H_{23}O_3$ $[M+H]^+$ calcd. 275.1642, found 275.1644.

4-(1,4-dihydroxy-2-naphthoyl)phenyl 2-(4-isobutylphenyl)propanoate (C-72):

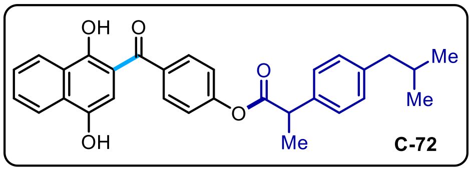

GP1 was followed using 1,4-naphthaquinone (0.3 mmol, 48 mg) and 4-formylphenyl 2-(4-isobutylphenyl)propanoate (140 mg, 0.45 mmol). After 24 h, purification by column chromatography using 12% ethyl acetate in PE yielded **C-72** (120 mg, 85%) as a yellow solid.

^{1}H NMR (400 MHz, $CDCl_3$) δ 13.42 (bs, 1H), 8.49 (ddd, *J* = 8.3, 1.3, 0.7 Hz, 1H), 8.13 (dt, *J* = 8.2, 1.0 Hz, 1H), 7.78 – 7.69 (m, 1H), 7.69 – 7.66 (m, 2H), 7.59 (ddd, *J* = 8.2, 6.9, 1.3 Hz, 1H), 7.34 – 7.28 (m, 2H), 7.18 – 7.14 (m, 2H), 7.12 – 7.07 (m, 2H), 6.81 (s, 1H), 5.61 (bs, 1H), 3.97 (q, *J* = 7.1 Hz, 1H), 2.48 (d, *J* = 7.2 Hz, 2H), 1.87 (dt, *J* = 13.5, 6.7 Hz, 1H), 1.63 (d, *J* = 7.1 Hz, 3H), 0.92 (s, 3H), 0.91 (s, 3H).

^{13}C NMR{^{1}H} (101 MHz, $CDCl_3$) δ 199.7, 173.2, 158.6, 153.4, 143.1, 141.2, 138.8, 137.0, 135.8, 134.1, 130.6, 130.2, 129.8, 127.3, 126.7, 124.7, 122.0, 121.6, 111.5, 107.7, 45.4, 45.2, 30.3, 22.5, 18.6.

ESI-HRMS: m/z calculated for $C_{30}H_{29}O_5$ $[M+H]^+$ calcd. 469.2010, found 469.2005.

4-(1,4-dihydroxy-2-naphthoyl)phenyl 5-(2,5-dimethylphenoxy)-2,2-dimethylpentanoate (C-73):

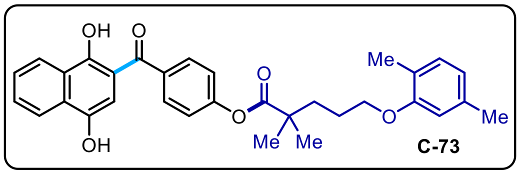

GP1 was followed using 1,4-naphthaquinone (0.3 mmol, 48 mg) and 4-formylphenyl 5-(2,5-dimethylphenoxy)-2,2-dimethylpentanoate (160 mg, 0.45 mmol). After 24 h, purification by column chromatography using 12% ethyl acetate in PE yielded **C-73** (58 mg, 38%) as a yellow solid.

^{1}H NMR (400 MHz, $CDCl_3$) δ 13.45 (s, 1H), 8.52 (dt, *J* = 8.2, 1.1 Hz, 1H), 8.14 (dt, *J* = 8.4, 1.0 Hz, 1H), 7.76 – 7.68 (m, 3H), 7.61 (ddd, *J* = 8.2, 6.9, 1.3 Hz, 1H), 7.20 – 7.16 (m, 2H), 7.01 (d, *J* = 7.4 Hz, 1H), 6.86 (s, 1H), 6.67 (d, *J* = 7.4 Hz, 1H), 6.64 (d, *J* = 1.6 Hz, 1H), 5.14 (s, 1H), 4.00 (q, *J* = 5.2 Hz, 2H), 2.31 (s, 3H), 2.18 (s, 3H), 1.90 (dt, *J* = 11.2, 3.8 Hz, 4H), 1.41 (s, 6H).

^{13}C NMR{^{1}H} (101 MHz, $CDCl_3$) δ 199.7, 176.3, 158.8, 156.9, 153.7, 142.9, 136.7, 135.8, 130.6, 130.5, 130.2, 129.5, 126.8, 126.2, 124.8, 123.7, 121.9, 121.8, 121.0, 112.1, 111.5, 107.8, 67.8, 42.8, 37.3, 28.0, 25.4, 25.3, 21.6, 16.0.

ESI-HRMS: m/z calculated for $C_{32}H_{33}O_6$ $[M+H]^+$ calcd. 513.2272, found 513.2270.

4-(1,4-dihydroxy-2-naphthoyl)phenyl 2-(2-fluoro-[1,1'-biphenyl]-4-yl)propanoate (C-74):

GP1 was followed using 1,4-naphthaquinone (0.3 mmol, 48 mg) and 4-formylphenyl 2-(2-fluoro-[1,1'-biphenyl]-4-yl)propanoate (157 mg, 0.45 mmol). After 24 h, purification by column chromatography using 15% ethyl acetate in PE yielded **C-74** (89 mg, 59%) as a yellow solid.

^{1}H NMR (400 MHz, $CDCl_3$) δ 13.44 (s, 1H), 8.51 (ddd, *J* = 8.4, 1.3, 0.7 Hz, 1H), 8.13 (dt, *J* = 8.3, 1.0 Hz, 1H), 8.04 – 7.99 (m, 1H), 7.82 – 7.78 (m, 1H), 7.74 – 7.70 (m, 2H), 7.56 (ddt, *J* = 7.6, 6.2, 1.5 Hz, 3H), 7.48 – 7.44 (m, 4H), 7.40 – 7.38 (m, 1H), 7.20 (dd, *J* = 8.7, 1.5 Hz, 3H), 6.85 (s, 1H), 4.06 – 4.02 (m, 1H), 1.68 (d, *J* = 3.0 Hz, 3H).

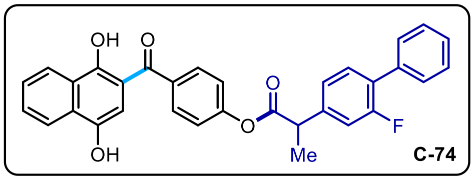

^{13}C NMR{^{1}H} (101 MHz, $CDCl_3$) δ 199.6, 172.4, 160.0 (d, $^{1}J_{C-F}$ = 239.4 Hz), 153.3, 142.9, 141.9 (d, $^{3}J_{C-F}$ = 7.6 Hz), 136.0, 135.5 (d, $^{3}J_{C-F}$ = 7.1 Hz), 132.5, 131.3, 130.6, 130.3, 129.6, 129.1 (d, $^{4}J_{C-F}$ = 3.9 Hz), 128.7, 128.0, 127.8 (d, $^{2}J_{C-F}$ = 21.3 Hz), 126.8, 126.2, 124.8, 123.7 (d, $^{4}J_{C-F}$ = 3.7 Hz), 122.1, 121.6, 116.0, 115.5 (d, $^{2}J_{C-F}$ = 23.7 Hz), 107.8, 45.3, 18.5.

ESI-HRMS: m/z calculated for $C_{32}H_{24}FO_5$ $[M+H]^+$ calcd. 507.1602, found 507.1597.

4-(1,4-dihydroxy-2-naphthoyl)phenyl 2-(4-((2-oxocyclopentyl)methyl)phenyl)propanoate (C-75):

GP1 was followed using 1,4-naphthaquinone (0.3 mmol, 48 mg) and 4-formylphenyl 2-(4-((2-oxocyclopentyl)methyl)phenyl)propanoate (158 mg, 0.45 mmol). After 24 h, purification by column chromatography using 22% ethyl acetate in PE yielded **C-75** (72 mg, 47%) as a yellow solid.

^{1}H NMR (400 MHz, DMSO-d_6) δ 12.79 (s, 1H), 9.80 (s, 1H), 8.35 (ddd, *J* = 8.4, 1.3, 0.7 Hz, 1H), 8.16 – 8.11 (m, 1H), 7.82 – 7.76 (m, 2H), 7.72 (ddd, *J* = 8.3, 6.9, 1.3 Hz, 1H), 7.64 (ddd, *J* = 8.2, 6.8, 1.3 Hz, 1H), 7.35 – 7.33 (m, 2H), 7.28 – 7.25 (m, 2H), 7.24 – 7.21 (m, 2H), 6.87 (s, 1H), 4.13 – 4.09 (m, 1H), 3.02 – 2.91 (m, 2H), 2.45 – 2.39 (m, 2H), 2.24 – 2.20 (m, 1H), 2.10 – 2.07 (m, 1H), 1.87 – 1.85 (m, 1H), 1.70 – 1.68 (m, 2H), 1.53 (d, *J* = 7.1 Hz, 3H).

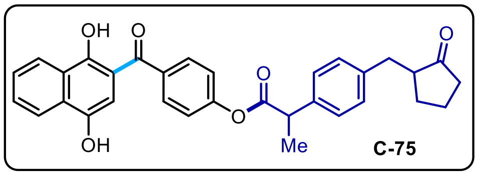

^{13}C NMR{^{1}H} (101 MHz, DMSO-d_6) δ 219.2, 199.2, 172.6, 154.4, 153.1, 144.7, 139.2, 137.6, 135.4, 131.1, 130.6, 129.5, 129.2, 127.4, 126.6, 125.3, 123.7, 122.2, 121.7, 112.8, 106.4, 49.9, 44.2, 37.5, 34.6, 28.7, 24.5, 20.0.

ESI-HRMS: m/z calculated for $C_{32}H_{28}O_6$ $[M+H]^+$ calcd. 509.1959, found 509.1962.

4-(1,4-dihydroxy-2-naphthoyl)phenyl 2-(6-methoxynaphthalen-2-yl)propanoate (C-76):

GP1 was followed using 1,4-naphthaquinone (0.3 mmol, 48 mg) and 4-formylphenyl 2-(6-methoxynaphthalen-2-yl)propanoate (150 mg, 0.45 mmol). After 24 h, purification by column chromatography using 20% ethyl acetate in PE yielded **C-76** (63 mg, 43%) as a yellow solid.

^{1}H NMR (400 MHz, $CDCl_3$) δ 13.36 (s, 1H), 8.47 (dd, *J* = 8.3, 1.2 Hz, 1H), 8.18 – 8.14 (m, 1H), 7.78 (s, 1H), 7.76 (d, *J* = 3.6 Hz, 2H), 7.72 – 7.70 (m, 2H), 7.57 (ddd, *J* = 8.2, 6.9, 1.3 Hz, 1H), 7.49 (dd, *J* = 8.6, 1.8 Hz, 1H), 7.20 – 7.14 (m, 3H), 7.13 – 7.09 (m, 3H), 6.94 (s, 1H), 4.14 (t, *J* = 6.8 Hz, 1H), 3.93 (s, 3H), 1.73 (s, 3H).

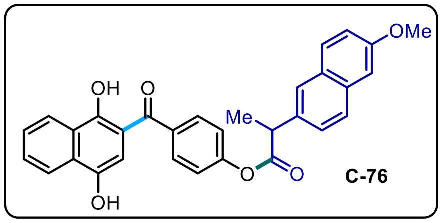

^{13}C NMR{^{1}H} (101 MHz, $CDCl_3$) 199.7, 173.8, 158.2, 158.0, 153.1, 143.9, 136.3, 134.6, 134.1, 132.4, 130.7, 130.0, 129.9, 129.4, 129.1, 127.7, 126.5, 126.4, 126.0, 124.5, 122.3, 121.4, 119.5, 115.2, 111.7, 107.3, 54.7, 45.7, 24.3.

ESI-HRMS: m/z calculated for $C_{31}H_{25}O_6$ $[M+H]^+$ calcd. 493.1646, found 493.1650.

4-(1,4-dihydroxy-2-naphthoyl)benzyl 2-(3-benzoylphenyl)propanoate (C-77):

GP1 was followed using 1,4-naphthaquinone (0.3 mmol, 48 mg) and 4-formylbenzyl 2-(3-benzoylphenyl)propanoate (167 mg, 0.45 mmol). After 24 h, purification by column chromatography using 20% ethyl acetate in PE yielded **C-77** (116 mg, 73%) as a yellow solid.

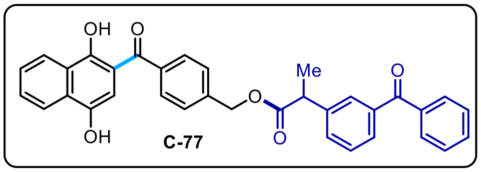

^{1}H NMR (400 MHz, $CDCl_3$) δ 13.52 (s, 1H), 8.48 (ddd, *J* = 8.4, 1.3, 0.7 Hz, 1H), 8.24 (dt, *J* = 8.2, 1.1 Hz, 1H), 7.75 – 7.72 (m, 2H), 7.67 (ddd, *J* = 7.3, 3.3, 1.3 Hz, 3H), 7.59 – 7.54 (m, 5H), 7.48 – 7.44 (m, 2H), 7.42 (s, 1H), 7.16 (d, *J* = 8.3 Hz, 2H), 6.86 (s, 1H), 5.30 (d, *J* = 12.6 Hz, 1H), 4.98 (d, *J* = 12.6 Hz, 1H), 3.90 (d, *J* = 7.2 Hz, 1H), 1.59 (d, *J* = 7.1 Hz, 3H).

^{13}C NMR{^{1}H} (101 MHz, $CDCl_3$) 200.2, 198.5, 173.5, 158.4, 143.8, 140.9, 139.5, 138.1, 137.9, 137.1, 133.2, 132.3, 130.3, 130.0, 129.9, 129.5, 129.3, 129.0, 128.9, 128.5, 127.6, 126.5, 126.1, 124.5, 122.4, 111.6, 107.3, 65.8, 45.5, 17.8.

ESI-HRMS: m/z calculated for $C_{34}H_{27}O_6$ $[M+H]^+$ calcd. 531.1802, found 531.1806.

4-(1,4-dihydroxy-2-naphthoyl)benzyl-2-(3-cyano-4-isobutoxyphenyl)-5-methylthiazole-4-carboxylate (C-78):

GP1 was followed using 1,4-naphthaquinone (0.3 mmol, 48 mg) and 4-formylbenzyl 2-(3-cyano-4-isobutoxyphenyl)-5-methylthiazole-4-carboxylate (195 mg, 0.45 mmol). After 24 h, purification by column chromatography using 25% ethyl acetate in PE yielded **C-78** (133 mg, 75%) as a yellow solid.

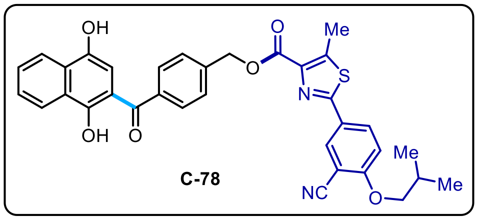

^{1}H NMR (400 MHz, DMSO-d_6) δ 12.97 (s, 1H), 9.78 (s, 1H), 8.35 (ddd, *J* = 8.3, 1.3, 0.7 Hz, 1H), 8.29 (d, *J* = 2.3 Hz, 1H), 8.22 (dd, *J* = 8.9, 2.4 Hz, 1H), 8.12 (ddd, *J* = 8.3, 1.3, 0.7 Hz, 1H), 7.80 – 7.76 (m, 2H), 7.72 (ddd, *J* = 8.3, 6.9, 1.3 Hz, 1H),

7.68 (d, *J* = 1.9 Hz, 1H), 7.66 – 7.61 (m, 2H), 7.34 (d, *J* = 9.0 Hz, 1H), 6.89 (s, 1H), 5.47 (s, 2H), 3.98 (d, *J* = 6.5 Hz, 2H), 2.71 (s, 3H), 2.10 – 2.04 (m, 1H), 1.01 (d, *J* = 6.7 Hz, 6H).

^{13}C NMR{^{1}H} (101 MHz, DMSO-*d*$_6$) 200.0, 167.2, 162.2, 161.1, 160.9, 154.8, 144.7, 139.5, 137.7, 133.1, 131.7, 129.5, 129.3, 129.1, 127.7, 126.6, 125.3, 125.0, 123.7, 122.2, 120.7, 115.3, 113.9, 112.6, 106.4, 101.6, 75.1, 66.0, 27.6, 18.7, 17.3.

ESI-HRMS: m/z calculated for $C_{34}H_{29}N_2O_6S$ $[M+H]^+$ calcd. 593.1741, found 593.1750.

4-(1,4-dihydroxy-2-naphthoyl)phenyl 2-(4-(4-chlorobenzoyl)phenoxy)-2-methylpropanoate (C-79):

GP1 was followed using 1,4-naphthaquinone (0.3 mmol, 48 mg) and 4-formylphenyl 2-(4-(4-chlorobenzoyl)phenoxy)-2-methylpropanoate (190 mg, 0.45 mmol). After 24 h, purification by column chromatography using 22% ethyl acetate in PE yielded **C-79** (133 mg, 78%) as a yellow solid.

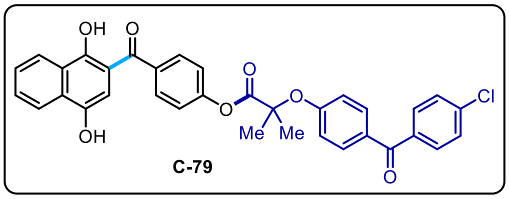

^{1}H NMR (400 MHz, $CDCl_3$) δ 13.40 (s, 1H), 8.50 – 8.46 (m, 1H), 8.17 – 8.13 (m, 1H), 7.79 – 7.76 (m, 2H), 7.69 (dt, *J* = 8.4, 1.9 Hz, 5H), 7.58 (ddd, *J* = 8.2, 6.9, 1.2 Hz, 1H), 7.45 – 7.43 (m, 2H), 7.09 – 7.05 (m, 2H), 7.01 – 6.97 (m, 2H), 6.84 (s, 1H), 2.15 (s, 1H), 1.85 (s, 6H).

^{13}C NMR{^{1}H} (101 MHz, $CDCl_3$) 199.4, 194.7, 172.2, 159.7, 158.7, 152.8, 143.4, 138.9, 136.4, 136.1, 132.4, 131.4, 130.9, 130.7, 130.2, 129.8, 128.8, 126.7, 126.1, 124.7, 122.1, 121.2, 117.5, 111.5, 107.5, 79.7, 25.6.

ESI-HRMS: m/z calculated for $C_{34}H_{26}ClO_7$ $[M+H]^+$ calcd. 581.1362, found 581.1364.

4-(1,4-dihydroxy-2-naphthoyl)benzyl 3-(4,5-diphenyloxazol-2-yl)propanoate (C-80):

GP1 was followed using 1,4-naphthaquinone (0.3 mmol, 48 mg) and 4-formylbenzyl 3-(4,5-diphenyloxazol-2-yl)propanoate (185 mg, 0.45 mmol). After 24 h, purification by column chromatography using 25% ethyl acetate in PE yielded **C-80** (104 mg, 61%) as a yellow solid.

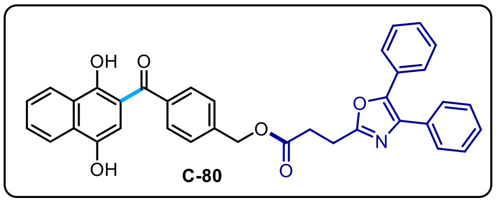

^{1}H NMR (400 MHz, DMSO-*d*$_6$) δ 12.87 (s, 1H), 9.77 (s, 1H), 8.38 – 8.34 (m, 1H), 8.14 (dt, *J* = 8.2, 1.1 Hz, 1H), 7.75 – 7.71 (m, 1H), 7.66 – 7.63 (m, 1H), 7.58 (d, *J* = 2.0 Hz, 2H), 7.55 (d, *J* = 1.3 Hz, 2H), 7.53 (d, *J* = 1.1 Hz, 2H), 7.50 (d, *J* = 1.2 Hz, 1H), 7.43 – 7.39 (m, 3H), 7.38 – 7.34 (m, 3H), 7.27 – 7.24 (m, 1H), 6.84 (s, 1H), 5.29 (s, 2H), 3.18 (dd, *J* = 7.6, 6.1 Hz, 2H), 3.02 (dd, *J* = 7.6, 6.1 Hz, 2H).

^{13}C NMR{^{1}H} (101 MHz, DMSO-d_6) 200.0, 171.7, 162.1, 154.5, 144.6, 140.1, 137.3, 135.2, 134.6, 134.4, 131.9, 129.5, 129.2, 128.9, 128.9, 128.6, 128.3, 128.1, 127.4, 127.2, 126.9, 126.3, 125.3, 123.7, 122.2, 112.7, 106.5, 65.0, 30.1, 24.1.

ESI-HRMS: m/z calculated for $C_{36}H_{28}NO_6$ $[M+H]^+$ calcd. 570.1911, found 570.1920.

4-(1,4-dihydroxy-2-naphthoyl)benzyl 4-([1,1'-biphenyl]-4-yl)-4-oxobutanoate (C-81):

GP1 was followed using 1,4-naphthaquinone (0.3 mmol, 48 mg) and 4-formylbenzyl 4-([1,1'-biphenyl]-4-yl)-4-oxobutanoate (167 mg, 0.45 mmol). After 24 h, purification by column chromatography using 22% ethyl acetate in PE yielded **C-81** (117 mg, 74%) as a yellow solid.

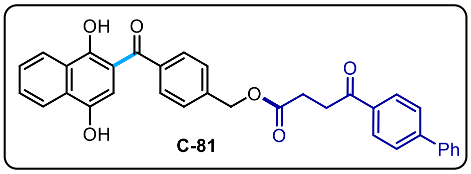

C-81

^{1}H NMR (400 MHz, DMSO-d_6) δ 12.98 (s, 1H), 9.80 (s, 1H), 8.37 (dt, J = 8.2, 1.0 Hz, 1H), 8.14 (dt, J = 8.3, 1.0 Hz, 1H), 8.10 – 8.07 (m, 2H), 7.84 – 7.82 (m, 2H), 7.76 – 7.70 (m, 6H), 7.66 – 7.62 (m, 1H), 7.59 (d, J = 8.2 Hz, 2H), 7.52 – 7.46 (m, 3H), 7.44 – 7.42 (m, 1H), 6.90 (s, 1H), 5.26 (s, 2H), 3.41 (t, J = 6.2 Hz, 2H), 2.84 – 2.80 (m, 2H).

^{13}C NMR{^{1}H} (101 MHz, DMSO-d_6) 200.1, 197.9, 172.3, 154.8, 144.7, 144.6, 140.1, 138.9, 137.4, 135.2, 135.1, 134.6, 129.5, 129.1, 128.9, 128.7, 128.4, 127.4, 127.0, 126.9, 125.3, 123.7, 122.2, 112.6, 106.5, 64.9, 33.1, 27.9.

ESI-HRMS: m/z calculated for $C_{34}H_{27}O_6$ $[M+H]^+$ calcd. 531.1802, found 531.1802.

4-(1,4-dihydroxy-2-naphthoyl)benzyl nicotinate (C-82):

GP1 was followed using 1,4-naphthaquinone (0.3 mmol, 48 mg) and 4-formylbenzyl nicotinate (1087 mg, 0.45 mmol). After 24 h, purification by column chromatography using 35% ethyl acetate in PE yielded **C-82** (97 mg, 81%) as a yellow solid.

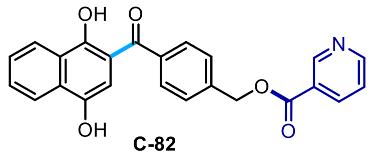

C-82

^{1}H NMR (400 MHz, DMSO-d_6) δ 12.96 (s, 1H), 9.79 (s, 1H), 9.19 (dd, J = 2.2, 0.9 Hz, 1H), 8.85 (dd, J = 4.8, 1.7 Hz, 1H), 8.40 – 8.35 (m, 2H), 8.13 (dt, J = 8.2, 1.1 Hz, 1H), 7.79 – 7.76 (m, 2H), 7.72 (dt, J = 7.8, 1.2 Hz, 3H), 7.66 – 7.61 (m, 2H), 6.89 (s, 1H), 5.53 (s, 2H).

^{13}C NMR{^{1}H} (101 MHz, DMSO-d_6) 200.1, 164.6, 154.8, 153.9, 150.1, 144.7, 139.7, 137.6, 137.1, 129.6, 129.3, 129.1, 127.7, 126.6, 125.5, 125.3, 124.1, 123.7, 122.3, 112.7, 106.5, 66.0.

ESI-HRMS: m/z calculated for $C_{24}H_{18}NO_5$ $[M+H]^+$ calcd. 400.1179, found 400.1184.

4-(1,4-dihydroxy-2-naphthoyl)phenyl-2-(4-(2,2-dichlorocyclopropyl)phenoxy)-2 methylpropanoate (C-83):

GP1 was followed using 1,4-naphthaquinone (0.3 mmol, 48 mg) and 4-formylphenyl 2-(4-(2,2-dichlorocyclopropyl)phenoxy)-2-methylpropanoate (176 mg, 0.45 mmol). After 24 h, purification by column chromatography using 30% ethyl acetate in PE yielded **C-83** (125 mg, 76%) as a yellow solid.

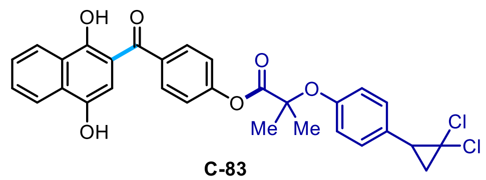

C-83

^{1}H NMR (400 MHz, $CDCl_3$ + DMSO-d_6) δ 13.12 (s, 1H), 8.92 (s, 1H), 8.24 (t, *J* = 8.7 Hz, 1H), 8.00 (d, *J* = 8.6 Hz, 1H), 7.57 (d, *J* = 8.3 Hz, 2H), 7.45 (d, *J* = 7.8 Hz, 1H), 7.39 – 7.33 (m, 1H), 7.01 – 6.92 (m, 4H), 6.73 (t, *J* = 4.9 Hz, 3H), 2.68 (t, *J* = 9.4 Hz, 1H), 2.56 – 2.53 (m, 1H), 1.80 – 1.74 (m, 1H), 1.59 (s, 6H).

^{13}C NMR{^{1}H} (101 MHz, $CDCl_3$ + DMSO-d_6) δ 199.2, 172.2, 157.0, 154.4, 152.4, 144.4, 136.1, 130.2, 129.8, 129.5, 129.3, 128.3, 125.9, 125.5, 123.9, 122.1, 121.0, 118.3, 111.4, 106.4, 78.9, 60.6, 34.4, 25.4, 25.2, 25.1.

ESI-HRMS: m/z calculated for $C_{30}H_{25}Cl_2O_6$ $[M+H]^+$ calcd. 551.1023, found 551.1027.

4-(1,4-dihydroxy-2-naphthoyl)phenyl 2-(1-(4-chlorobenzoyl)-5-methoxy-3-methyl-1*H*-indol-2 yl)acetate (C-84):

GP1 was followed using 1,4-naphthaquinone (0.3 mmol, 48 mg) and 4-formylphenyl 2-(1-(4-chlorobenzoyl)-5-methoxy-3-methyl-1*H*-indol-2-yl)acetate (207 mg, 0.45 mmol). After 24 h, purification by column chromatography using 30% ethyl acetate in PE yielded **C-84** (97 mg, 52%) as a yellow solid.

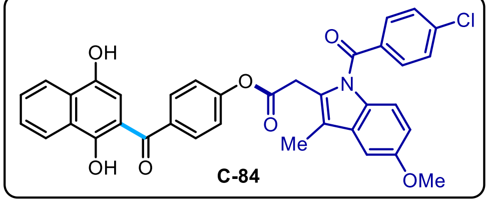

C-84

^{1}H NMR (400 MHz, DMSO-d_6) δ 12.79 (s, 1H), 9.80 (s, 1H), 8.35 (ddd, *J* = 8.3, 1.3, 0.7 Hz, 1H), 8.14 – 8.10 (m, 1H), 7.82 (d, *J* = 6.7 Hz, 2H), 7.77 – 7.75 (m, 1H), 7.73 – 7.71 (m, 1H), 7.70 (d, *J* = 2.4 Hz, 2H), 7.67 (d, *J* = 2.2 Hz, 2H), 7.40 – 7.36 (m, 2H), 7.21 (d, *J* = 2.5 Hz, 1H), 6.94 (d, *J* = 2.8 Hz, 1H), 6.88 (s, 1H), 6.75 (dd, *J* = 9.0, 2.6 Hz, 1H), 4.16 (s, 2H), 3.79 (s, 3H), 2.33 (s, 3H).

^{13}C NMR{^{1}H} (101 MHz, DMSO-d_6) δ 199.2, 169.3, 167.9, 163.3, 155.7, 154.3, 153.1, 144.7, 137.7, 135.9, 135.5, 134.1, 132.1, 131.2, 130.6, 130.5, 130.3, 129.5, 129.1, 126.6, 125.3, 121.9, 115.8, 114.7, 112.9, 112.1, 111.5, 106.4, 101.8, 55.5, 24.1, 13.3.

ESI-HRMS: m/z calculated for $C_{36}H_{27}ClNO_7$ $[M+H]^+$ calcd. 620.1471, found 620.1466.

1,7,7-trimethylbicyclo[2.2.1]heptan-2-yl 4-(1,4-dihydroxy-2-naphthoyl)benzoate (C-85):

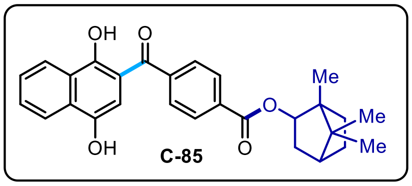

GP1 was followed using 1,4-naphthaquinone (0.3 mmol, 48 mg) and 1,7,7-trimethylbicyclo[2.2.1]heptan-2-yl 4-formylbenzoate (129 mg, 0.45 mmol). After 24 h, purification by column chromatography using 15% ethyl acetate in PE yielded **C-85** (64 mg, 48%) as a yellow solid.

^{1}H NMR (400 MHz, $CDCl_3$) δ 13.48 (s, 1H), 8.53 (d, *J* = 8.3 Hz, 1H), 8.18 – 8.08 (m, 3H), 7.76 – 7.70 (m, 3H), 7.62 (ddd, *J* = 8.2, 7.0, 1.3 Hz, 1H), 6.83 (s, 1H), 4.95 (dd, *J* = 7.4, 4.1 Hz, 1H), 1.92 (d, *J* = 4.1 Hz, 1H), 1.84 – 1.69 (m, 3H), 1.64 – 1.50 (m, 3H), 1.13 (s, 3H), 0.94 (s, 3H), 0.90 (s, 3H).

^{13}C NMR{^{1}H} (101 MHz, $CDCl_3$) δ 200.0, 165.6, 159.1, 143.3, 142.2, 133.2, 130.4, 129.8, 129.6, 128.9, 126.9, 126.2, 124.8, 122.1, 111.5, 107.3, 82.5, 49.3, 47.2, 45.2, 39.0, 33.8, 27.2, 24.0, 20.3, 11.8.

ESI-HRMS: m/z calculated for $C_{28}H_{29}O_5$ $[M+H]^+$ calcd. 445.2010, found 445.2017.

2,6-diisopropylphenyl 4-(1,4-dihydroxy-2-naphthoyl)benzoate (C-86):

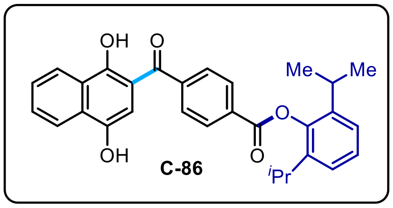

GP1 was followed using 1,4-naphthaquinone (0.3 mmol, 48 mg) and 2,6-diisopropylphenyl 4-formylbenzoate (139 mg, 0.45 mmol). After 24 h, purification by column chromatography using 15% ethyl acetate in PE yielded **C-86** (76 mg, 54%) as a yellow solid.

^{1}H NMR (400 MHz, $CDCl_3$) δ 13.50 (s, 1H), 8.54 (dt, *J* = 8.1, 1.1 Hz, 1H), 8.41 – 8.36 (m, 2H), 8.14 (dt, *J* = 8.3, 1.0 Hz, 1H), 7.87 – 7.83 (m, 2H), 7.75 – 7.71 (m, 1H), 7.66 – 7.61 (m, 1H), 7.30 – 7.27 (m, 1H), 7.26 (s, 1H), 7.25 – 7.22 (m, 2H), 6.84 (s, 1H), 5.30 (bs, 1H), 2.97 (hep, *J* = 6.9 Hz, 2H), 1.32 (d, *J* = 6.9 Hz, 3H), 1.25 (d, *J* = 6.9 Hz, 3H), 1.24 (d, *J* = 6.9 Hz, 3H), 1.22 (d, *J* = 6.9 Hz, 3H).

^{13}C NMR{^{1}H} (101 MHz, $CDCl_3$) δ 199.9, 164.8, 159.3, 145.8, 143.2, 143.1, 140.5, 133.9, 131.8, 130.6, 130.3, 129.9, 129.1, 127.0, 127.0, 126.2, 124.9, 124.3, 124.3, 122.0, 111.4, 107.3, 27.9, 24.1, 24.0, 22.8.

ESI-HRMS: m/z calculated for $C_{30}H_{29}O_5$ $[M+H]^+$ calcd. 469.2010, found 469.2013.

2-isopropyl-5-methylphenyl 4-(1,4-dihydroxy-2-naphthoyl)benzoate (C-87):

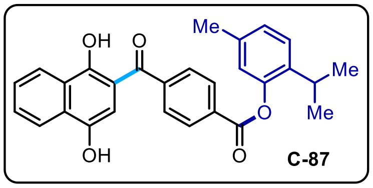

GP1 was followed using 1,4-naphthaquinone (0.3 mmol, 48 mg) and 2-isopropyl-5-methylphenyl 4-formylbenzoate (127 mg, 0.45 mmol). After 24 h, purification by column chromatography using 15% ethyl acetate in PE yielded **C-87** (66 mg, 50%) as a yellow solid.

^{1}H NMR (400 MHz, $CDCl_3$) δ 13.50 (s, 1H), 8.54 (ddd, *J* = 8.4, 1.4, 0.7 Hz, 1H), 8.39 – 8.32 (m, 2H), 8.14 (dt, *J* = 8.3, 1.1 Hz, 1H), 7.88 – 7.79 (m, 2H), 7.74 (ddd, *J* = 8.3, 6.9, 1.3 Hz, 1H), 7.63 (ddd, *J* = 8.2, 6.9,

1.2 Hz, 1H), 7.28 (s, 1H), 7.10 (dd, J = 8.0, 1.8 Hz, 1H), 6.97 – 6.95 (m, 1H), 6.82 (s, 1H), 5.16 (s, 1H), 3.06 (hep, J = 6.9 Hz, 1H), 2.36 (s, 3H), 1.24 (d, J = 6.9 Hz, 6H).

^{13}C NMR{^{1}H} (101 MHz, $CDCl_3$) δ 199.9, 164.9, 159.3, 148.1, 143.1, 142.9, 137.2, 137.0, 132.1, 130.6, 130.3, 129.0, 127.6, 127.0, 126.8, 124.9, 122.8, 122.0, 120.5, 118.3, 111.4, 107.3, 30.5, 27.5, 23.2, 21.0.

ESI-HRMS: m/z calculated for $C_{28}H_{25}O_5$ $[M+H]^+$ calcd. 441.1697, found 441.1692.

(1*S*,2*S*,5*S*)-2-isopropyl-5-methylcyclohexyl 4-(1,4-dihydroxy-2-naphthoyl)benzoate (C-88):

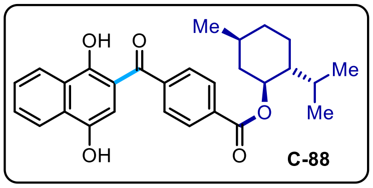

GP1 was followed using 1,4-naphthaquinone (0.3 mmol, 48 mg) and (1*S*,2*S*,5*S*)-2-isopropyl-5-methylcyclohexyl 4-formylbenzoate (130 mg, 0.45 mmol). After 24 h, purification by column chromatography using 15% ethyl acetate in PE yielded **C-88** (120 mg, 90%) as a yellow solid.

^{1}H NMR (400 MHz, $CDCl_3$) δ 13.48 (s, 1H), 8.53 (ddd, J = 8.4, 1.3, 0.7 Hz, 1H), 8.17 (dt, J = 8.3, 1.0 Hz, 1H), 8.14 – 8.10 (m, 2H), 7.76 – 7.71 (m, 3H), 7.62 (ddd, J = 8.3, 6.9, 1.3 Hz, 1H), 6.83 (s, 1H), 5.71 (s, 1H), 4.98 (td, J = 10.9, 4.4 Hz, 1H), 2.16 – 2.10 (m, 1H), 1.94 (tt, J = 7.0, 3.4 Hz, 1H), 1.78 – 1.72 (m, 2H), 1.62 – 1.54 (m, 3H), 1.16 (d, J = 2.1 Hz, 1H), 1.13 (t, J = 2.3 Hz, 1H), 0.95 (d, J = 2.2 Hz, 3H), 0.93 (d, J = 2.6 Hz, 3H), 0.91 – 0.89 (m, 1H), 0.81 (d, J = 6.9 Hz, 3H).

^{13}C NMR{^{1}H} (101 MHz, $CDCl_3$) δ 200.0, 165.7, 159.1, 143.3, 142.2, 133.1, 130.5, 129.8, 129.6, 128.8, 126.9, 126.2, 124.8, 122.1, 111.5, 107.3, 75.9, 47.4, 41.1, 34.4, 31.6, 26.7, 23.7, 22.2, 20.9, 16.7.

ESI-HRMS: m/z calculated for $C_{28}H_{31}O_5$ $[M+H]^+$ calcd. 447.2166, found 447.2168.

5-chloro-2-(2,4-dichlorophenoxy)phenyl 4-(1,4-dihydroxy-2-naphthoyl)benzoate (C-89):

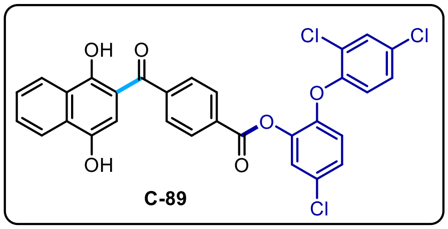

GP1 was followed using 1,4-naphthaquinone (0.3 mmol, 48 mg) and 5-chloro-2-(2,4-dichlorophenoxy)phenyl 4-formylbenzoate (188 mg, 0.45 mmol). After 24 h, purification by column chromatography using 15% ethyl acetate in PE yielded **C-89** (85 mg, 49%) as a yellow solid.

^{1}H NMR (400 MHz, $CDCl_3$) δ 13.47 (s, 1H), 8.53 (ddd, J = 8.4, 1.3, 0.7 Hz, 1H), 8.22 – 8.19 (m, 2H), 8.14 (dt, J = 8.4, 0.9 Hz, 1H), 7.79 – 7.71 (m, 4H), 7.66 – 7.61 (m, 1H), 7.57 – 7.51 (m, 1H), 7.38 (d, J = 1.4 Hz, 1H), 7.23 (d, J = 2.5 Hz, 1H), 7.18 (dd, J = 8.8, 2.5 Hz, 1H), 6.96 (d, J = 8.8 Hz, 1H), 6.91 (d, J = 8.8 Hz, 1H), 6.75 (s, 1H).

^{13}C NMR{^{1}H} (101 MHz, $CDCl_3$) δ 199.7, 163.5, 159.4, 151.1, 147.0, 143.0, 141.7, 130.6, 130.6, 130.4, 129.8, 129.5, 128.9, 128.3, 127.5, 127.0, 126.2, 124.9, 124.6, 122.2, 122.0, 120.8, 120.3, 118.8, 114.9, 111.3, 108.0, 107.3.

ESI-HRMS: m/z calculated for $C_{30}H_{21}Cl_3NO_6$ [M+NH4]+ calcd. 596.0429, found 596.0427.

((1*R*,5*S*)-6,6-dimethylbicyclo[3.1.1]hept-2-en-3-yl)methyl 4-(1,4-dihydroxy-2-naphthoyl)benzoate (C-90):

GP1 was followed using 1,4-naphthaquinone (0.3 mmol, 48 mg) and ((1*R*,5*S*)-6,6-dimethylbicyclo[3.1.1]hept-2-en-3-yl)methyl 4-formylbenzoate (128 mg, 0.45 mmol). After 24 h, purification by column chromatography using 12% ethyl acetate in PE yielded **C-90** (97 mg, 73%) as a yellow solid.

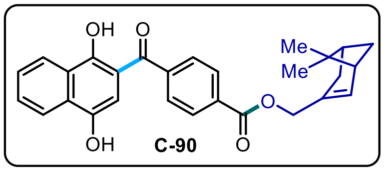

^{1}H NMR (400 MHz, $CDCl_3$) 13.49 (s, 1H), 8.53 (ddd, *J* = 8.4, 1.3, 0.7 Hz, 1H), 8.21 – 8.19 (m, 1H), 8.17 – 8.15 (m, 2H), 7.95 (dt, *J* = 8.0, 0.8 Hz, 1H), 7.76 – 7.75 (m, 1H), 7.74 (t, *J* = 1.2 Hz, 1H), 7.63 (ddd, *J* = 8.2, 6.9, 1.2 Hz, 1H), 6.98 (s, 1H), 6.80 (s, 1H), 5.69 (td, *J* = 3.0, 1.5 Hz, 1H), 4.75 (q, *J* = 1.5 Hz, 2H), 2.33 (d, *J* = 13.8 Hz, 2H), 2.26 – 2.20 (m, 2H), 2.16 – 2.10 (m, 2H), 1.32 (s, 3H), 0.88 (s, 3H).

^{13}C NMR{^{1}H} (101 MHz, $CDCl_3$) δ 200.1, 165.9, 159.2, 143.0, 138.8, 134.1, 130.5, 129.7, 128.8, 126.9, 126.6, 124.9, 122.4, 122.0, 111.4, 107.4, 68.2, 66.7, 53.7, 43.9, 40.9, 38.3, 31.7, 31.5, 26.3, 21.4.

ESI-HRMS: m/z calculated for $C_{28}H_{27}O_5$ [M+H]+ calcd. 443.1853, found 443.1853.

2-methoxy-4-(3-oxobutyl)phenyl 4-(1,4-dihydroxy-2-naphthoyl)benzoate (C-91):

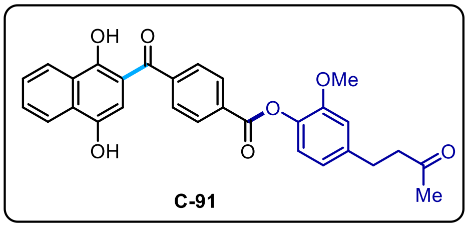

GP1 was followed using 1,4-naphthaquinone (0.3 mmol, 48 mg) and 2-methoxy-4-(3-oxobutyl)phenyl 4-formylbenzoate (148 mg, 0.45 mmol). After 24 h, purification by column chromatography using 15% ethyl acetate in PE yielded **C-91** (46 mg, 32%) as a yellow solid.

^{1}H NMR (400 MHz, DMSO-d_6) δ 12.79 (s, 1H), 9.80 (s, 1H), 8.38 (dt, *J* = 8.4, 0.9 Hz, 1H), 8.32 – 8.28 (m, 2H), 8.16 – 8.13 (m, 1H), 7.94 – 7.91 (m, 2H), 7.77 – 7.73 (m, 1H), 7.68 – 7.65 (m, 1H), 7.16 (d, *J* = 8.0 Hz, 1H), 7.08 (d, *J* = 1.9 Hz, 1H), 6.86 (dd, *J* = 8.1, 1.9 Hz, 1H), 6.82 (s, 1H), 3.78 (s, 3H), 2.83 (dt, *J* = 5.3, 2.6 Hz, 4H), 2.13 (s, 3H).

^{13}C NMR{^{1}H} (126 MHz, DMSO-d_6) δ 207.7, 199.7, 163.5, 154.8, 150.5, 144.9, 142.5, 140.7, 137.3, 134.6, 131.1, 129.9, 129.4, 129.1, 126.8, 125.3, 123.8, 122.5, 122.3, 120.2, 113.0, 112.7, 106.2, 55.8, 44.2, 29.8, 29.0.

ESI-HRMS: m/z calculated for $C_{29}H_{25}O_7$ [M+H]+ calcd. 485.1595, found 485.1595.

(2,5-dihydroxyphenyl)(phenyl)methanone (C-92):[7]

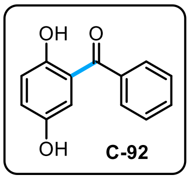

GP1 was followed using *p*-benzoquinone (0.3 mmol, 33 mg) benzaldehyde (48 mg, 0.45 mmol). After 24 h, purification by column chromatography using 12% ethyl acetate in PE yielded **C-92** (52 mg, 80%) as a yellow solid.

^{1}H NMR (400 MHz, $CDCl_3$) δ 11.58 (s, 1H), 7.66 – 7.62 (m, 2H), 7.58 – 7.52 (m, 1H), 7.50 – 7.44 (m, 2H), 7.06 – 7.01 (m, 2H), 6.95 (dd, *J* = 8.7, 0.7 Hz, 1H), 5.45 (s, 1H).

^{13}C NMR{^{1}H} (101 MHz, $CDCl_3$) δ 201.4, 157.3, 147.5, 137.8, 132.1, 129.2, 128.5, 125.0, 119.3, 118.5, 116.2.

(2,5-dihydroxyphenyl)(4-ethylphenyl)methanone (C-93):[7]

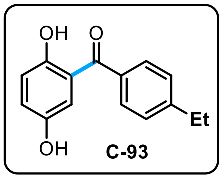

GP1 was followed using *p*-benzoquinone (0.3 mmol, 33 mg) 4-ethylbenzaldehyde (60 mg, 0.45 mmol). After 24 h, purification by column chromatography using 12% ethyl acetate in PE yielded **C-93** (58 mg, 79%) as a yellow solid.

^{1}H NMR (400 MHz, $CDCl_3$) δ 11.51 (s, 1H), 7.51 – 7.45 (m, 2H), 7.21 – 7.16 (m, 2H), 6.98 – 6.92 (m, 2H), 6.84 (dd, *J* = 8.7, 0.6 Hz, 1H), 5.52 (s, 1H), 2.61 (q, *J* = 7.6 Hz, 2H), 1.16 (t, *J* = 7.6 Hz, 3H).

^{13}C NMR{^{1}H} (101 MHz, $CDCl_3$) δ 201.2, 157.1, 149.2, 147.5, 135.2, 129.5, 128.0, 124.8, 119.2, 119.1, 118.6, 29.0, 15.3.

(4-chlorophenyl)(2,5-dihydroxyphenyl)methanone (C-94):[7]

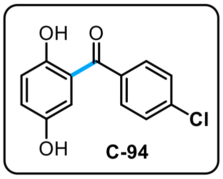

GP1 was followed using *p*-benzoquinone (0.3 mmol, 33 mg) 4-chlorobenzaldehyde (63 mg, 0.45 mmol). After 24 h, purification by column chromatography using 12% ethyl acetate in PE yielded **C-94** (55 mg, 74%) as a yellow solid.

^{1}H NMR (400 MHz, DMSO-d_6) δ 9.80 (s, 1H), 9.12 (s, 1H), 7.75 – 7.68 (m, 2H), 7.61 – 7.55 (m, 2H), 6.91 (dd, *J* = 8.8, 3.0 Hz, 1H), 6.82 (dd, *J* = 8.9, 3.6 Hz, 1H), 6.74 (d, *J* = 3.0 Hz, 1H).

^{13}C NMR{^{1}H} (101 MHz, DMSO-d_6) δ 196.4, 150.0, 149.7, 137.5, 136.3, 131.0, 128.6, 123.9, 121.3, 117.9, 115.7.

(2,5-dihydroxyphenyl)(2-fluorophenyl)methanone (C-95):[7]

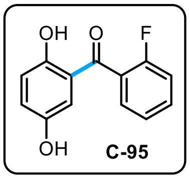

GP1 was followed using *p*-benzoquinone (0.3 mmol, 33 mg) 24-fluorobenzaldehyde (47 mg, 0.45 mmol). After 24 h, purification by column chromatography using 12% ethyl acetate in PE yielded **C-95** (56 mg, 67%) as a yellow solid.

¹H NMR (400 MHz, $CDCl_3$) δ 11.52 (s, 1H), 7.49 – 7.42 (m, 1H), 7.37 (td, *J* = 7.2, 1.8 Hz, 1H), 7.23 – 7.19 (m, 1H), 7.10 (ddd, *J* = 9.1, 8.2, 1.0 Hz, 1H), 7.01 (dd, *J* = 8.9, 3.1 Hz, 1H), 6.89 (d, *J* = 8.9 Hz, 1H), 6.80 (t, *J* = 2.8 Hz, 1H), 5.41 (s, 1H).

^{13}C NMR{^{1}H} (101 MHz, $CDCl_3$) δ 198.4, 159.1 (d, $^1J_{C\text{-}F}$ = 251.7 Hz), 157.2, 147.8, 133.1 (d, $^3J_{C\text{-}F}$ = 8.2 Hz), 129.8 (d, $^4J_{C\text{-}F}$ = 2.9 Hz), 126.2 (d, $^2J_{C\text{-}F}$ = 15.7 Hz), 126.0, 124.5 (d, $^3J_{C\text{-}F}$ = 7.6 Hz), 122.6, 119.2, 116.4 (d, $^2J_{C\text{-}F}$ = 21.2 Hz), 116.2.

^{19}F NMR (376 MHz, $CDCl_3$) δ -112.91.

(2,5-dihydroxyphenyl)(3,5-dimethoxyphenyl)methanone (C-96):

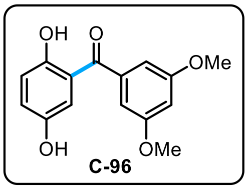

GP1 was followed using *p*-benzoquinone (0.3 mmol, 33 mg) 3,5-dimethoxybenzaldehyde (75 mg, 0.45 mmol). After 24 h, purification by column chromatography using 18% ethyl acetate in PE yielded **C-94** (56 mg, 68%) as a yellow solid.

¹H NMR (400 MHz, DMSO-d_6) δ 10.04 (s, 1H), 9.10 (s, 1H), 6.91 (dd, *J* = 8.8, 3.0 Hz, 1H), 6.82 (d, *J* = 8.8 Hz, 1H), 6.80 – 6.76 (m, 4H), 3.78 (s, 6H).

^{13}C NMR{^{1}H} (101 MHz, DMSO-d_6) δ 197.7, 160.3, 151.0, 149.4, 139.4, 123.1, 121.8, 117.9, 115.9, 106.9, 104.0, 55.5.

ESI-HRMS: m/z calculated for $C_{15}H_{15}O_5$ $[M+H]^+$ calcd. 275.0914, found 275.0918.

(2,5-dihydroxyphenyl)(thiophen-2-yl)methanone (C-97):[7]

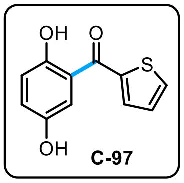

GP1 was followed using *p*-benzoquinone (0.3 mmol, 33 mg) thiophene-2-carbaldehyde (50 mg, 0.45 mmol). After 24 h, purification by column chromatography using 15% ethyl acetate in PE yielded **C-97** (48 mg, 72%) as a yellow solid.

¹H NMR (400 MHz, DMSO-d_6) δ 9.59 (s, 1H), 9.10 (s, 1H), 8.05 (dd, *J* = 4.9, 1.2 Hz, 1H), 7.60 (dd, *J* = 3.8, 1.2 Hz, 1H), 7.24 (dd, *J* = 4.9, 3.8 Hz, 1H), 6.87 – 6.80 (m, 3H).

^{13}C NMR{^{1}H} (101 MHz, DMSO-d_6) δ 188.3, 149.5, 149.0, 143.4, 135.3, 135.3, 128.6, 124.9, 120.3, 117.8, 115.0.

cyclohex-3-en-1-yl(2,5-dihydroxyphenyl)methanone (C-98):

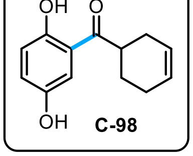

GP1 was followed using *p*-benzoquinone (0.3 mmol, 33 mg) cyclohex-3-ene-1-carbaldehyde (50 mg, 0.45 mmol). After 24 h, purification by column chromatography using 15% ethyl acetate in PE yielded **C-98** (43 mg, 65%) as a yellow solid.

^{1}H NMR (400 MHz, DMSO-d_6) δ 11.25 (s, 1H), 9.15 (s, 1H), 7.19 (d, J = 3.0 Hz, 1H), 6.98 (dd, J = 8.8, 3.0 Hz, 1H), 6.81 (d, J = 8.8 Hz, 1H), 5.72 (d, J = 1.9 Hz, 2H), 3.56 (tdd, J = 11.3, 7.6, 2.6 Hz, 1H), 2.21 – 2.08 (m, 4H), 1.94 – 1.88 (m, 1H), 1.58 – 1.49 (m, 1H).

^{13}C NMR{^{1}H} (101 MHz, DMSO-d_6) δ 208.3, 153.7, 149.5, 126.5, 125.6, 124.0, 119.6, 118.6, 114.6, 41.6, 27.3, 25.5, 24.4.

ESI-HRMS: m/z calculated for $C_{13}H_{15}O_3$ $[M+H]^+$ calcd. 219.1016, found 219.1016.

(2,5-dihydroxy-4-methylphenyl)(phenyl)methanone and (3,6-dihydroxy-2-methylphenyl)(phenyl)methanone (C-99 & C-99'):

GP1 was followed using 2-methylcyclohexa-2,5-diene-1,4-dione (0.3 mmol, 37 mg) benzaldehyde (48 mg, 0.45 mmol). After 24 h, purification by column chromatography using 15% ethyl acetate in PE yielded (**C-99 + C-99'**) (as an inseparable regioisomeric mixture, 52 mg, 76%) as a yellow solid.

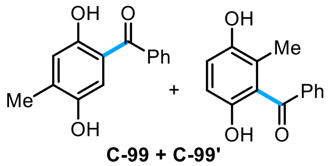

C-99 + C-99'

^{1}H NMR (400 MHz, $CDCl_3$) δ 11.88 (s, 0.79 H), 11.69 (s, 1H), 7.63 (ddd, J = 8.4, 4.1, 1.4 Hz, 3.63H), 7.54 (dd, J = 7.6, 1.9 Hz, 1.82H), 7.48 – 7.44 (m, 3.63H), 6.94 (d, J = 7.2 Hz, 1.84H), 6.86 – 6.84 (m, 1.8H), 2.28 (d, J = 0.9 Hz, 5.45H).

^{13}C NMR{^{1}H} (101 MHz, $CDCl_3$) δ 201.6, 200.9, 157.6, 156.1, 146.6, 146.0, 138.1, 136.1, 131.9, 131.9, 130.3, 130.3, 129.1, 129.0, 128.5, 128.4, 126.0, 120.2, 118.2, 117.7, 117.0, 115.7, 16.8, 15.9.

ESI-HRMS: m/z calculated for $C_{14}H_{13}O_3$ $[M+H]^+$ calcd. 229.0859, found 229.0864.

2-benzoyl-3-chloronaphthalene-1,4-dione (C-100):

GP1 was followed using 2-chloronaphthalene-1,4-dione (0.3 mmol, 58 mg) benzaldehyde (48 mg, 0.45 mmol). After 24 h, purification by column chromatography using 15% ethyl acetate in PE yielded **C-100** (60 mg, 67%) as a brown solid.

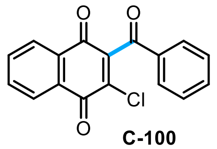

C-100

^{1}H NMR (400 MHz, $CDCl_3$) δ 8.27 – 8.22 (m, 1H), 8.14 – 8.10 (m, 1H), 7.96 – 7.90 (m, 2H), 7.86 – 7.82 (m, 2H), 7.69 – 7.64 (m, 1H), 7.54 – 7.50 (m, 2H).

^{13}C NMR{^{1}H} (101 MHz, $CDCl_3$) δ 189.8, 181.4, 177.5, 144.4, 141.8, 135.0, 134.8, 134.7, 131.4, 131.3, 129.5, 129.4, 129.3, 127.8, 127.3.

ESI-HRMS: m/z calculated for $C_{17}H_{10}ClO_3$ $[M+H]^+$ calcd. 297.0313, found 297.0318.

2-bromo-3-(4-chlorobenzoyl)naphthalene-1,4-dione (C-101):

GP1 was followed using 2-bromonaphthalene-1,4-dione (0.3 mmol, 58 mg) 4-chlorobenzaldehyde (63 mg, 0.45 mmol). After 24 h, purification by column chromatography using 15% ethyl acetate in PE yielded **C-101** (73 mg, 65%) as a brown solid.

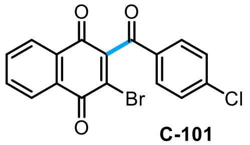

C-101

^{1}H NMR (400 MHz, $CDCl_3$) δ 8.28 – 8.23 (m, 1H), 8.14 – 8.09 (m, 1H), 7.91 – 7.86 (m, 2H), 7.84 (dd, *J* = 5.8, 3.3 Hz, 2H), 7.53 – 7.47 (m, 2H).

^{13}C NMR{^{1}H} (101 MHz, $CDCl_3$) δ 189.3, 180.9, 177.2, 147.7, 141.7, 136.5, 135.0, 134.9, 132.6, 131.3, 131.0, 130.9, 129.8, 128.2, 127.4.

ESI-HRMS: m/z calculated for $C_{17}H_9BrClO_3$ $[M+H]^+$ calcd. 374.9418, found 374.9416.

(1,4-dihydroxy-3-methylnaphthalen-2-yl)(4-methoxyphenyl)methanone (C-102):

GP1 was followed using 2-methylnaphthalene-1,4-dione (0.3 mmol, 51 mg) 4-methoxybenzaldehyde (61 mg, 0.45 mmol). After 24 h, purification by column chromatography using 15% ethyl acetate in PE yielded **C-102** (36 mg, 39%) as a yellow solid.

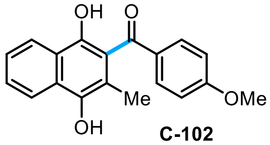

C-102

^{1}H NMR (400 MHz, $CDCl_3$) δ 8.33 – 8.29 (m, 2H), 8.05 (dd, *J* = 7.9, 1.0 Hz, 1H), 7.72 – 7.69 (m, 1H), 7.46 – 7.39 (m, 2H), 7.07 – 7.04 (m, 2H), 6.56 (s, 1H), 5.77 (s, 1H), 3.93 (s, 3H), 2.25 (s, 3H).

^{13}C NMR{^{1}H} (101 MHz, $CDCl_3$) δ 190.9, 165.4, 164.3, 149.5, 132.7, 128.1, 127.1, 126.9, 124.8, 124.0, 122.2, 121.6, 120.9, 114.2, 111.2, 55.7, 16.6.

ESI-HRMS: m/z calculated for $C_{19}H_{17}O_4$ $[M+H]^+$ calcd. 309.1121, found 309.1124.

1-(1,4-dihydroxy-3-methylnaphthalen-2-yl)-2-methylpropan-1-one (C-103):

GP1 was followed using 2-methylnaphthalene-1,4-dione (0.3 mmol, 51 mg) isobutyraldehyde (32 mg, 0.45 mmol). After 24 h, purification by column chromatography using 15% ethyl acetate in PE yielded **C-103** (18 mg, 25%) as a yellow solid.

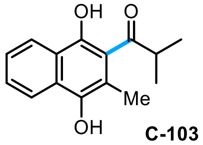

C-103

^{1}H NMR (400 MHz, $CDCl_3$) δ 8.08 (q, *J* = 1.0 Hz, 1H), 7.78 – 7.76 (m, 1H), 7.50 – 7.46 (m, 1H), 7.42 (ddd, *J* = 8.2, 6.8, 1.3 Hz, 1H), 6.60 (s, 1H), 5.35 (s, 1H), 3.05 – 2.98 (m, 1H), 2.22 (s, 3H), 1.46 (d, *J* = 7.0 Hz, 6H).

^{13}C NMR{^{1}H} (101 MHz, $CDCl_3$) δ 207.3, 172.9, 149.1, 134.0, 127.0, 126.7, 124.7, 123.7, 122.0, 120.5, 111.0, 34.3, 29.7, 19.3.

ESI-HRMS: m/z calculated for $C_{15}H_{17}O_3$ $[M+H]^+$ calcd. 245.1172, found 245.1175.

(1,4-dihydroxy-5-methoxynaphthalen-2-yl)(4-methoxyphenyl)methanone (C-104):

GP1 was followed using 5-methoxynaphthalene-1,4-dione (0.3 mmol, 57 mg) 4-methoxybenzaldehyde (62 mg, 0.45 mmol). After 24 h, purification by column chromatography using 25% ethyl acetate in PE yielded **C-104** (64 mg, 66%) as a brown solid.

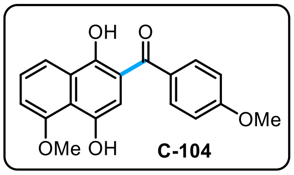

^{1}H NMR (400 MHz, DMSO-d_6) δ 10.06 (s, 1H), 9.70 (s, 1H), 7.78 – 7.72 (m, 3H), 7.48 (dd, J = 8.5, 7.7 Hz, 1H), 7.06 – 7.03 (m, 3H), 6.78 (s, 1H), 3.99 (s, 3H), 3.84 (s, 3H).

^{13}C NMR{^{1}H} (101 MHz, DMSO-d_6) δ 195.6, 162.9, 157.0, 147.0, 144.8, 131.7, 130.7, 128.8, 127.4, 118.9, 115.2, 115.1, 113.7, 108.2, 106.2, 56.2, 55.5.

ESI-HRMS: m/z calculated for $C_{19}H_{17}O_5$ [M+H]$^+$ calcd. 325.1071, found 325.1076.

(1,4-dihydroxyanthracen-2-yl)(4-methoxyphenyl)methanone (C-105):

GP1 was followed using anthracene-1,4-dione (0.3 mmol, 62 mg) 4-methoxybenzaldehyde (62 mg, 0.45 mmol). After 24 h, purification by column chromatography using 18% ethyl acetate in PE yielded **C-105** (23 mg, 22%) as a brown solid.

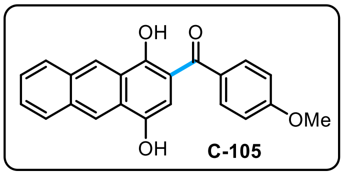

^{1}H NMR (400 MHz, DMSO-d_6) δ 13.80 (s, 1H), 9.92 (s, 1H), 9.07 (s, 1H), 8.73 (s, 1H), 8.26 – 8.17 (m, 2H), 7.81 – 7.78 (m, 2H), 7.64 – 7.56 (m, 2H), 7.19 – 7.14 (m, 2H), 6.89 (s, 1H), 3.89 (s, 3H).

^{13}C NMR{^{1}H} (101 MHz, DMSO-d_6) δ 198.5, 162.3, 156.7, 144.4, 135.3, 133.0, 131.4, 129.0, 128.4, 127.5, 127.0, 126.3, 124.3, 124.2, 122.0, 121.2, 113.8, 111.0, 103.6, 55.6.

ESI-HRMS: m/z calculated for $C_{22}H_{17}O_4$ [M+H]$^+$ calcd. 345.1151, found 345.1156.

(1,4-dihydroxychrysen-3-yl)(4-methoxyphenyl)methanone (C-106):

GP1 was followed using chrysene-1,4-dione (0.3 mmol, 77 mg) 4-methoxybenzaldehyde (62 mg, 0.45 mmol). After 24 h, purification by column chromatography using 20% ethyl acetate in PE yielded **C-106** (86 mg, 73%) as a orange solid.

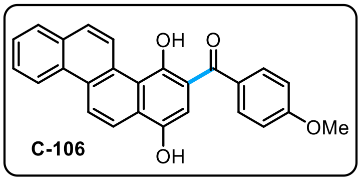

^{1}H NMR (400 MHz, DMSO-d_6) δ 9.32 (d, J = 8.8 Hz, 1H), 8.38 (d, J = 7.4 Hz, 2H), 8.32 – 8.29 (m, 2H), 8.02 – 7.99 (m, 2H), 7.83 – 7.80 (m, 1H), 7.72 (ddd, J = 8.6, 6.8, 1.5 Hz, 2H), 7.62 – 7.55 (m, 3H), 7.26 – 7.18 (m, 2H), 3.93 (s, 3H).

^{13}C NMR{^{1}H} (101 MHz, DMSO-d_6) δ 182.5, 180.0, 163.8, 137.4, 136.8, 135.9, 135.8, 133.4, 132.5, 131.5, 130.7, 130.3, 129.9, 129.0, 128.9, 128.3, 127.3, 126.5, 125.9, 125.5, 124.4, 121.9, 114.2, 55.7.

ESI-HRMS: m/z calculated for $C_{26}H_{19}O_5$ [M+H]$^+$ calcd. 395.1278, found 395.1275.

3-benzoyl-1-(*p*-tolyl)pyrrolidine-2,5-dione (C-107):

GP1 was followed using 1-(*p*-tolyl)-1H-pyrrole-2,5-dione (0.3 mmol, 56 mg) benzaldehyde (48 mg, 0.45 mmol). After 24 h, purification by column chromatography using 20% ethyl acetate in PE yielded **C-107** (54 mg, 61%) as an orange solid.

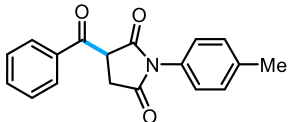

C-107

^{1}H NMR (400 MHz, $CDCl_3$) δ 8.16 – 8.13 (m, 2H), 7.68 – 7.59 (m, 2H), 7.57 – 7.50 (m, 3H), 7.18 – 7.14 (m, 2H), 4.99 (dd, *J* = 9.1, 4.1 Hz, 1H), 3.57 – 3.48 (m, 1H), 3.07 – 2.98 (m, 1H), 2.37 (s, 3H).

^{13}C NMR{^{1}H} (101 MHz, $CDCl_3$) δ 192.6, 175.0, 172.1, 139.0, 134.5, 133.8, 130.3, 130.0, 128.9, 128.6, 126.4, 48.7, 32.0, 21.3.

ESI-HRMS: m/z calculated for $C_{18}H_{16}NO_3$ $[M+H]^+$ calcd. 294.1125, found 294.1128.

2-(tetrahydrofuran-2-yl)naphthalene-1,4-diol (C-109):

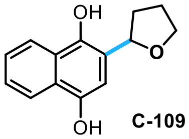

C-109

GP3 was followed using 1,4-naphthaquinone (0.3 mmol, 48 mg) and tetrahydrofuran (65 mg, 0.9 mmol). After 24 h, purification by column chromatography using 20% ethyl acetate in PE yielded **C-109** (53 mg, 77%) as a dark grey solid.

^{1}H NMR (400 MHz, $CDCl_3$) δ 13.66 (s, 1H), 8.47 – 8.41 (m, 1H), 8.11 (dt, *J* = 8.1, 1.0 Hz, 1H), 7.67 (ddd, *J* = 8.3, 6.9, 1.3 Hz, 1H), 7.56 (ddd, *J* = 8.2, 6.9, 1.2 Hz, 1H), 7.07 (s, 1H), 5.78 (s, 1H), 5.14 (dd, *J* = 4.3, 1.8 Hz, 1H), 3.95 – 3.85 (m, 2H), 3.74 (ddd, *J* = 9.8, 6.8, 5.7 Hz, 1H), 3.55 – 3.49 (m, 1H), 3.12 – 2.96 (m, 2H).

^{13}C NMR{^{1}H} (101 MHz, $CDCl_3$) δ 178.3, 157.3, 143.3, 129.8, 126.6, 124.7, 121.8, 112.0, 105.5, 104.2, 67.3, 66.4, 35.6, 32.4.

EI-HRMS: m/z calculated for $C_{14}H_{14}O_3$ calcd. 230.0937, found 230.0929.

2-(1,3-dihydroisobenzofuran-1-yl)naphthalene-1,4-diol (C-110):

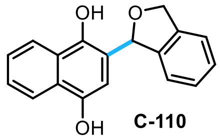

C-110

GP3 was followed using 1,4-naphthaquinone (0.3 mmol, 48 mg) and 1,3-dihydroisobenzofuran (108 mg, 0.9 mmol). After 24 h, purification by column chromatography using 20% ethyl acetate in PE yielded **C-110** (44 mg, 63%) as a dark brown solid.

^{1}H NMR (400 MHz, $CDCl_3$) δ 8.09 (dd, *J* = 5.7, 3.3 Hz, 1H), 7.93 (dt, *J* = 7.7, 1.0 Hz, 1H), 7.80 – 7.73 (m, 2H), 7.69 (td, *J* = 7.5, 1.1 Hz, 1H), 7.56 – 7.52 (m, 1H), 7.50 (dt, *J* = 7.7, 0.9 Hz, 1H), 7.36 – 7.31 (m, 1H), 7.24 – 7.17 (m, 1H), 6.98 (s, 1H), 5.33 (s, 2H), 5.00 (s, 1H), 1.62 (s, 1H).

^{13}C NMR{^{1}H} (101 MHz, $CDCl_3$) δ 185.2, 171.2, 146.7, 138.8, 134.5, 134.1, 134.1, 132.1, 129.2, 128.6, 128.1, 127.2, 126.6, 126.3, 125.9, 122.2, 84.9, 69.8.

EI-HRMS: m/z calculated for $C_{18}H_{14}O_3$ calcd. 278.093, found 278.0931.

2-(tetrahydro-2*H*-pyran-2-yl)naphthalene-1,4-diol (C-111):

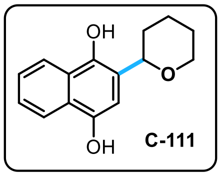

GP3 was followed using 1,4-naphthaquinone (0.3 mmol, 48 mg) and tetrahydro-2*H*-pyran (78 mg, 0.9 mmol). After 24 h, purification by column chromatography using 20% ethyl acetate in PE yielded **C-111** (46 mg, 62%) as a dark grey solid.

^{1}H NMR (400 MHz, DMSO-d_6) δ 9.67 (s, 1H), 8.13 – 8.06 (m, 2H), 7.53 – 7.44 (m, 2H), 6.96 (d, *J* = 8.3 Hz, 1H), 6.75 (d, *J* = 8.3 Hz, 1H), 5.46 (t, *J* = 3.4 Hz, 1H), 3.82 (ddd, *J* = 11.2, 8.8, 3.9 Hz, 1H), 3.58 – 3.50 (m, 1H), 2.00 (ddd, *J* = 13.9, 7.8, 3.6 Hz, 1H), 1.88 (ddd, *J* = 7.8, 4.5, 2.3 Hz, 2H), 1.69 – 1.55 (m, 3H).

^{13}C NMR{^{1}H} (101 MHz, DMSO-d_6) δ 147.6, 144.5, 126.6, 125.6, 125.1, 124.9, 122.1, 121.4, 110.1, 107.2, 96.7, 61.5, 30.2, 24.9, 18.8.

EI-HRMS: m/z calculated for $C_{15}H_{16}O_3$ calcd. 244.1094, found 244.1089.

2-(isochroman-1-yl)naphthalene-1,4-diol (C-112):

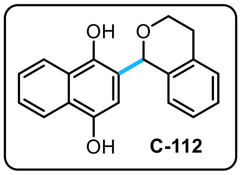

GP3 was followed using 1,4-naphthaquinone (0.3 mmol, 48 mg) and isochroman (121 mg, 0.9 mmol). After 24 h, purification by column chromatography using 20% ethyl acetate in PE yielded **C-112** (68 mg, 78%) as a dark brown solid.

^{1}H NMR (400 MHz, DMSO-d_6) δ 9.78 (s, 1H), 8.15 – 8.11 (m, 1H), 8.03 – 7.98 (m, 1H), 7.46 (ddd, *J* = 10.6, 6.8, 3.7 Hz, 3H), 7.34 (td, *J* = 7.4, 1.8 Hz, 2H), 7.26 (dd, *J* = 7.6, 5.4 Hz, 2H), 6.83 (d, *J* = 8.2 Hz, 1H), 6.37 (s, 1H), 4.23 – 4.17 (m, 1H), 3.98 (ddd, *J* = 11.2, 6.1, 1.5 Hz, 1H), 2.98 (dt, *J* = 11.9, 5.6 Hz, 1H), 2.76 (dt, *J* = 15.9, 2.4 Hz, 1H).

^{13}C NMR{^{1}H} (101 MHz, DMSO-d_6) δ 148.1, 145.5, 134.3, 133.7, 129.4, 128.5, 127.8, 127.7, 127.4, 126.4, 125.8, 125.0, 122.1, 121.5, 111.2, 107.3, 96.1, 67.2, 27.0.

EI-HRMS: m/z calculated for $C_{19}H_{16}O_3$ calcd. 292.1094, found 292.1087.

2-(1,4-dioxan-2-yl)naphthalene-1,4-diol (C-113):

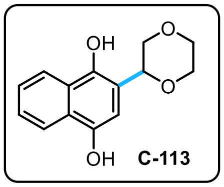

GP3 was followed using 1,4-naphthaquinone (0.3 mmol, 48 mg) and 1,4-dioxane (80 mg, 0.9 mmol). After 24 h, purification by column chromatography using 25% ethyl acetate in PE yielded **C-113** (48 mg, 65%) as a dark brown liquid.

^{1}H NMR (400 MHz, DMSO-d_6) δ 9.74 (s, 1H), 8.11 – 8.08 (m, 2H), 7.52 – 7.46 (m, 2H), 7.00 (d, *J* = 8.3 Hz, 1H), 6.76 (d, *J* = 8.2 Hz, 1H), 5.38 (t, *J* = 2.6 Hz, 1H), 4.01 (td, *J* = 7.4, 3.6 Hz, 1H), 3.86 (d, *J* = 2.6 Hz, 2H), 3.73 – 3.69 (m, 2H), 3.59 – 3.55 (m, 1H).

^{13}C NMR{^{1}H} (101 MHz, DMSO-d_6) δ 148.0, 144.3, 126.6, 125.8, 125.1, 125.0, 122.1, 121.4, 110.6, 107.2, 94.3, 68.1, 65.4, 60.9.

ESI-HRMS: m/z calculated for $C_{14}H_{15}O_4$ $[M+H]^+$ calcd. 247.0965, found 247.0970.

2-(1,3,5-trioxan-2-yl)naphthalene-1,4-diol (C-114):

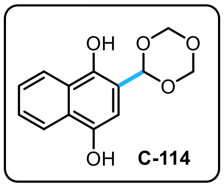

GP3 was followed using 1,4-naphthaquinone (0.3 mmol, 48 mg) and 1,3,5-trioxane (81 mg, 0.9 mmol). After 24 h, purification by column chromatography using 35% ethyl acetate in PE yielded **C-114** (60 mg, 80%) as a dark brown solid.

^{1}H NMR (400 MHz, DMSO-d_6) δ 9.97 (s, 1H), 8.14 – 8.05 (m, 2H), 7.58 – 7.46 (m, 2H), 7.03 (d, J = 8.2 Hz, 1H), 6.77 (d, J = 8.2 Hz, 1H), 6.26 (s, 1H), 5.46 (d, J = 6.3 Hz, 2H), 5.22 (d, J = 6.7 Hz, 2H).

^{13}C NMR{^{1}H} (101 MHz, DMSO-d_6) δ 149.3, 141.7, 126.9, 126.3, 125.3, 125.1, 122.2, 121.3, 113.3, 109.6, 107.0, 89.3.

ESI-HRMS: m/z calculated for $C_{13}H_{12}O_5Na$ $[M+Na]^+$ calcd. 271.0577, found 271.0575.

3-(2-(1,4-dihydroxynaphthalen-2-yl)benzo[d][1,3]dioxol-5-yl)-2-methylpropanal (C-115):

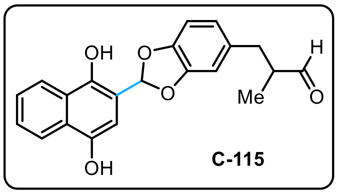

GP3 was followed using 1,4-naphthaquinone (0.3 mmol, 48 mg) and 3-(benzo[d][1,3]dioxol-5-yl)-2-methylpropanal (173 mg, 0.9 mmol). After 24 h, purification by column chromatography using 40% ethyl acetate in PE yielded **C-115** (72 mg, 69%) as a dark brown liquid.

^{1}H NMR (400 MHz, DMSO-d_6) δ 9.66 – 9.62 (m, 1H), 8.74 (s, 1H), 8.68 (s, 1H), 8.20 – 8.01 (m, 1H), 7.91 (tddd, J = 11.0, 8.4, 7.0, 4.0 Hz, 1H), 7.57 – 7.39 (m, 1H), 6.86 – 6.68 (m, 2H), 6.63 (d, J = 7.9 Hz, 1H), 6.56 (d, J = 2.1 Hz, 1H), 6.42 (dd, J = 8.0, 2.2 Hz, 1H), 5.96 (s, 1H), 2.83 (dd, J = 13.7, 6.2 Hz, 1H), 2.62 – 2.56 (m, 1H), 2.43 (dd, J = 13.7, 7.8 Hz, 1H), 0.93 (d, J = 6.9 Hz, 3H).

^{13}C NMR{^{1}H} (101 MHz, DMSO-d_6) δ 205.4, 150.0, 145.0, 143.6, 143.3, 133.6, 129.7, 126.5, 125.4, 122.6, 122.2, 119.6, 118.7, 116.3, 115.5, 109.6, 108.4, 106.9, 47.4, 35.3, 12.8.

ESI-HRMS: m/z calculated for $C_{21}H_{18}O_5$ $[M+Na]^+$ calcd. 373.1046, found 373.1044.

(1,4-dihydroxynaphthalen-2-yl)(phenyl)methyl acetate (C-116):

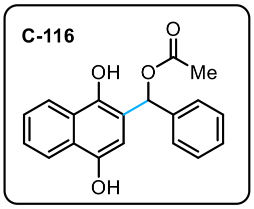

GP3 was followed using 1,4-naphthaquinone (0.3 mmol, 48 mg) and benzyl acetate (135 mg, 0.9 mmol). After 24 h, purification by column chromatography using 30% ethyl acetate in PE yielded **C-116** (50 mg, 54%) as a dark brown liquid.

^{1}**H NMR** (400 MHz, DMSO-d_6) δ 9.95 (s, 1H), 8.12 – 8.09 (m, 1H), 8.04 – 8.02 (m, 1H), 7.70 – 7.67 (m, 2H), 7.52 – 7.46 (m, 5H), 7.30 (s, 1H), 7.03 (d, J = 8.2 Hz, 1H), 6.77 (d, J = 8.2 Hz, 1H), 2.03 (s, 3H).

^{13}**C NMR{^{1}H}** (101 MHz, DMSO-d_6) δ 169.5, 149.1, 143.9, 137.0, 129.5, 128.7, 126.9, 126.5, 126.2, 125.3, 125.2, 122.2, 121.3, 112.2, 107.1, 96.1, 20.8.

ESI-HRMS: m/z calculated for $C_{19}H_{16}O_4$ $[M+Na]^+$ calcd. 331.0941, found 331.0943.

2-(2-phenoxypropan-2-yl)naphthalene-1,4-diol (C-117):

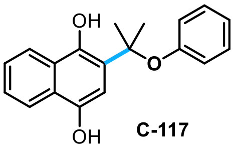

C-117

GP3 was followed using 1,4-naphthaquinone (0.3 mmol, 48 mg) and isopropoxybenzene (122 mg, 0.9 mmol). After 24 h, purification by column chromatography using 18% ethyl acetate in PE yielded **C-117** (44 mg, 50%) as a dark brown liquid.

^{1}**H NMR** (400 MHz, DMSO-d_6) δ 9.95 (s, 1H), 8.12 – 8.09 (m, 1H), 8.04 – 8.02 (m, 1H), 7.70 – 7.67 (m, 2H), 7.52 – 7.46 (m, 5H), 7.30 (s, 1H), 7.03 (d, J = 8.2 Hz, 1H), 6.77 (d, J = 8.2 Hz, 1H), 2.03 (s, 3H).

^{13}**C NMR{^{1}H}** (101 MHz, DMSO-d_6) δ 184.9, 154.6, 149.0, 141.9, 138.7, 134.2, 131.6, 129.4, 125.9, 123.0, 122.0, 121.1, 116.2, 107.3, 105.2, 25.5.

ESI-HRMS: m/z calculated for $C_{19}H_{19}O_3$ $[M+H]^+$ calcd. 295.1329, found 295.1324.

2-(tetrahydrothiophen-2-yl)naphthalene-1,4-diol (C-118):

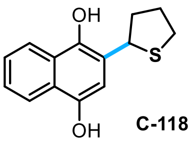

C-118

GP3 was followed using 1,4-naphthaquinone (0.3 mmol, 48 mg) and tetrahydrothiophen (80 mg, 0.9 mmol). After 24 h, purification by column chromatography using 20% ethyl acetate in PE yielded **C-118** (56 mg, 76%) as a yellow solid.

^{1}**H NMR** (400 MHz, DMSO-d_6) δ 9.68 (s, 1H), 8.10 – 8.07 (m, 1H), 8.03 – 7.99 (m, 1H), 7.49 – 7.44 (m, 2H), 6.75 (q, J = 8.2 Hz, 2H), 6.08 (dd, J = 4.8, 1.7 Hz, 1H), 2.98 (td, J = 6.8, 3.4 Hz, 1H), 2.88 – 2.82 (m, 1H), 2.27 – 2.17 (m, 2H), 2.11 – 1.98 (m, 2H).

^{13}**C NMR{^{1}H}** (101 MHz, DMSO-d_6) δ 147.3, 144.7, 138.7, 126.5, 125.6, 125.2, 122.0, 121.5, 108.2, 107.0, 87.0, 38.0, 31.8, 28.3.

EI-HRMS: m/z calculated for $C_{14}H_{14}O_2S$ calcd. 246.0709, found 246.0705.

2-(2-(phenylthio)propan-2-yl)naphthalene-1,4-diol (C-119):

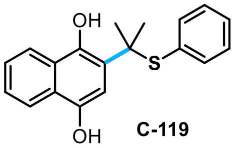

C-119

GP3 was followed using 1,4-naphthaquinone (0.3 mmol, 48 mg) and isopropyl(phenyl)sulfane (137 mg, 0.9 mmol). After 24 h, purification by column chromatography using 18% ethyl acetate in PE yielded **C-119** (57 mg, 61%) as a dark brown liquid.

^{1}H NMR (400 MHz, DMSO-d_6) δ 9.93 (s, 1H), 8.11 (ddd, J = 6.3, 2.3, 0.7 Hz, 1H), 7.95 – 7.92 (m, 1H), 7.68 (d, J = 8.3 Hz, 1H), 7.61 – 7.58 (m, 2H), 7.48 – 7.44 (m, 2H), 7.40 – 7.38 (m, 2H), 7.09 (s, 1H), 6.83 (d, J = 8.3 Hz, 1H), 1.61 (s, 6H).

^{13}C NMR{^{1}H} (101 MHz, DMSO-d_6) δ 149.2, 142.5, 138.7, 134.8, 134.2, 129.6, 128.9, 128.5, 125.9, 125.2, 124.8, 122.1, 116.6, 107.0, 90.8, 28.6.

ESI-HRMS: m/z calculated for $C_{19}H_{19}O_2S$ $[M+H]^+$ calcd. 311.1100, found 311.1102.

2-(dimethyl(phenyl)silyl)naphthalene-1,4-diol (C-120):

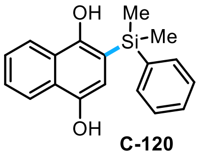

C-120

GP3 was followed using 1,4-naphthaquinone (0.3 mmol, 48 mg) and dimethyl(phenyl)silane (122 mg, 0.9 mmol). After 24 h, purification by column chromatography using 8% ethyl acetate in PE yielded **C-120** (52 mg, 58%) as a brown liquid.

^{1}H NMR (400 MHz, $CDCl_3$) δ 8.19 – 8.07 (m, 2H), 7.72 – 7.69 (m, 1H), 7.62 – 7.59 (m, 1H), 7.51 – 7.48 (m, 1H), 7.46 – 7.36 (m, 4H), 6.60 (s, 1H), 0.56 (s, 3H), 0.42 (s, 3H).

^{13}C NMR{^{1}H} (101 MHz, $CDCl_3$) δ 145.9, 145.2, 138.8, 133.6, 133.2, 130.1, 128.1, 126.6, 125.9, 125.7, 122.8, 121.7, 112.5, 108.3, 0.1, -1.0.

ESI-HRMS: m/z calculated for $C_{18}H_{18}O_2SiNa$ $[M+Na]^+$ calcd. 317.0869, found 317.0809.

2-(methyldiphenylsilyl)naphthalene-1,4-diol (C-121):

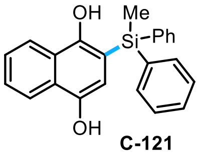

C-121

GP3 was followed using 1,4-naphthaquinone (0.3 mmol, 48 mg) and methyldiphenylsilane (178 mg, 0.9 mmol). After 24 h, purification by column chromatography using 8% ethyl acetate in PE yielded **C-121** (72 mg, 67%) as a brown liquid.

^{1}H NMR (400 MHz, $CDCl_3$) δ 7.77 – 7.73 (m, 4H), 7.65 – 7.62 (m, 4H), 7.47 – 7.40 (m, 9H), 0.70 (s, 3H).

^{13}C NMR{^{1}H} (101 MHz, $CDCl_3$) δ 145.9, 145.1, 137.1, 135.8, 134.5, 134.1, 130.3, 130.0, 128.4, 128.2, 128.1, 126.0, 125.7, 125.5, 122.8, 121.7, 112.4, 108.2, -2.6.

ESI-HRMS: m/z calculated for $C_{23}H_{21}O_2Si$ $[M+H]^+$ calcd. 357.1305, found 357.1297.

2-(triethylsilyl)naphthalene-1,4-diol (C-122):

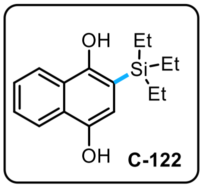

GP3 was followed using 1,4-naphthaquinone (0.3 mmol, 48 mg) and triethylsilane (104 mg, 0.9 mmol). After 24 h, purification by column chromatography using 5% ethyl acetate in PE yielded **C-122** (65 mg, 78%) as a pale-yellow liquid.

^{1}H NMR (400 MHz, $CDCl_3$) δ 8.14 – 8.09 (m, 2H), 7.76 (dd, *J* = 5.7, 3.3 Hz, 1H), 7.49 (dt, *J* = 6.4, 3.4 Hz, 2H), 6.98 (s, 1H), 6.69 (d, *J* = 3.7 Hz, 1H), 1.02 (t, *J* = 7.8 Hz, 9H), 0.81 (q, *J* = 7.8 Hz, 6H).

^{13}C NMR{^{1}H} (101 MHz, $CDCl_3$) δ 145.6, 138.8, 134.1, 126.6, 125.8, 125.7, 122.7, 121.6, 111.9, 108.4, 6.9, 5.3.

ESI-HRMS: m/z calculated for $C_{16}H_{23}O_2Si$ $[M+H]^+$ calcd. 275.1462, found 275.1461.

10-(1,4-dihydroxynaphthalen-2-yl)anthracen-9(10*H*)-one (C-124):

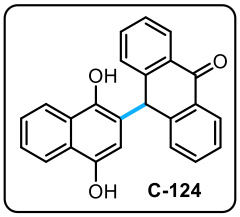

GP3 was followed using 1,4-naphthaquinone (0.3 mmol, 48 mg) and anthracen-9(10*H*)-one (174 mg, 0.9 mmol). After 24 h, purification by column chromatography using 12% ethyl acetate in PE yielded **C-124** (90 mg, 85%) as a yellow solid.

^{1}H NMR (400 MHz, $CDCl_3$) δ 8.40 – 8.38 (m, 2H), 8.12 – 8.09 (m, 1H), 8.01 – 7.98 (m, 1H), 7.75 – 7.71 (m, 2H), 7.62 – 7.46 (m, 6H), 7.40 (ddd, *J* = 7.0, 1.5, 0.7 Hz, 2H), 6.47 (s, 1H), 5.93 (s, 1H).

^{13}C NMR{^{1}H} (101 MHz, $CDCl_3$) δ 184.9, 152.7, 141.7, 137.1, 134.3, 134.2, 134.1, 133.6, 132.0, 131.9, 131.8, 128.6, 128.2, 128.1, 127.2, 126.7, 126.3, 41.2.

ESI-HRMS: m/z calculated for $C_{24}H_{17}O_3$ $[M+H]^+$ calcd. 353.1172, found 353.1169.

2-(9*H*-fluoren-9-yl)naphthalene-1,4-diol (C-125):

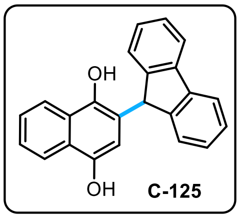

GP3 was followed using 1,4-naphthaquinone (0.3 mmol, 48 mg) and 9*H*-fluorene (149 mg, 0.9 mmol). After 24 h, purification by column chromatography using 8% ethyl acetate in PE yielded **C-125** (54 mg, 56%) as a yellow liquid.

^{1}H NMR (400 MHz, $CDCl_3$) δ 8.58 (d, *J* = 8.4 Hz, 1H), 8.20 (dd, *J* = 7.7, 1.4 Hz, 1H), 8.03 (d, *J* = 8.4 Hz, 1H), 7.90 (d, *J* = 7.6 Hz, 2H), 7.76 – 7.54 (m, 5H), 7.42 (td, *J* = 7.4, 1.4 Hz, 2H), 7.25 (s, 1H), 7.21 (td, *J* = 7.4, 1.1 Hz, 2H), 5.50 (s, 1H), 4.96 (s, 1H).

^{13}C NMR{^{1}H} (101 MHz, $CDCl_3$) δ 154.3, 148.8, 140.5, 134.5, 133.4, 132.3, 130.7, 128.3, 127.7, 126.9, 126.4, 124.9, 121.9, 121.7, 120.3, 105.9, 51.5.

ESI-HRMS: m/z calculated for $C_{23}H_{17}O_2$ $[M+H]^+$ calcd. 325.1223, found 325.1224.

3-(1,4-dihydroxynaphthalen-2-yl)-2,3-dihydro-1*H*-inden-1-one (C-126):

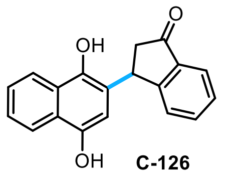

C-126

GP3 was followed using 1,4-naphthaquinone (0.3 mmol, 48 mg) and 2,3-dihydro-1*H*-inden-1-one (119 mg, 0.9 mmol). After 24 h, purification by column chromatography using 15% ethyl acetate in PE yielded **C-126** (63 mg, 72%) as a yellow liquid.

^{1}H NMR (400 MHz, DMSO-d_6) δ 8.00 – 7.95 (m, 2H), 7.88 – 7.85 (m, 2H), 7.74 – 7.67 (m, 2H), 7.55 – 7.49 (m, 2H), 6.75 (s, 1H), 4.76 (dd, J = 8.4, 3.4 Hz, 1H), 3.13 – 3.06 (m, 1H), 2.78 – 2.72 (m, 1H).

^{13}C NMR{^{1}H} (101 MHz, DMSO-d_6) δ 204.4, 184.7, 154.7, 151.5, 150.9, 136.9, 135.0, 134.9, 134.2, 134.2, 132.0, 131.5, 128.3, 126.7, 126.4, 126.2, 125.6, 123.1, 43.0.

ESI-HRMS: m/z calculated for $C_{19}H_{15}O_3$ $[M+H]^+$ calcd. 291.1016, found 291.1014.

1-(6-(tert-butyl)-3-(1,4-dihydroxynaphthalen-2-yl)-1,1-dimethyl-2,3-dihydro-1*H*-inden-4-yl)ethan-1-one (C-127):

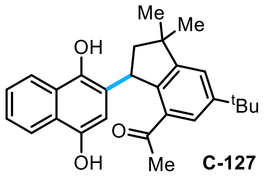

C-127

GP3 was followed using 1,4-naphthaquinone (0.3 mmol, 48 mg) and 1-(6-(tert-butyl)-1,1-dimethyl-2,3-dihydro-1*H*-inden-4-yl)ethan-1-one (219 mg, 0.9 mmol). After 24 h, purification by column chromatography using 12% ethyl acetate in PE yielded **C-127** (72 mg, 60%) as a yellow liquid.

^{1}H NMR (400 MHz, $CDCl_3$) δ 8.10 (ddd, J = 8.3, 1.3, 0.7 Hz, 1H), 8.01 (ddd, J = 8.4, 1.4, 0.7 Hz, 1H), 7.74 (d, J = 1.8 Hz, 1H), 7.47 (d, J = 1.8 Hz, 1H), 7.45 – 7.41 (m, 1H), 7.38 – 7.34 (m, 1H), 6.77 (d, J = 8.2 Hz, 1H), 6.68 (d, J = 8.2 Hz, 1H), 6.31 (dd, J = 6.5, 2.3 Hz, 1H), 5.92 (s, 1H), 2.52 (s, 3H), 1.42 (s, 9H), 1.41 – 1.39 (m, 5H), 1.38 (s, 3H).

^{13}C NMR{^{1}H} (101 MHz, $CDCl_3$) δ 201.5, 155.1, 153.2, 148.1, 145.5, 136.9, 136.0, 127.1, 125.6, 125.5, 125.2, 125.1, 123.4, 122.4, 121.6, 108.2, 106.2, 78.7, 47.4, 43.2, 35.2, 31.6, 28.9, 21.2, 14.3.

ESI-HRMS: m/z calculated for $C_{27}H_{31}O_3$ $[M+H]^+$ calcd. 403.2268, found 403.2273.

2-(1-phenylcyclopropyl)naphthalene-1,4-diol (C-128):

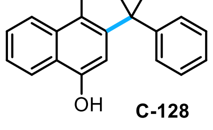

C-128

GP3 was followed using 1,4-naphthaquinone (0.3 mmol, 48 mg) and cyclopropylbenzene (106 mg, 0.9 mmol). After 24 h, purification by column chromatography using 6% ethyl acetate in PE yielded **C-128** (51 mg, 61%) as a colourless sticky liquid.

^{1}H NMR (400 MHz, $CDCl_3$) δ 8.19 (dq, J = 7.9, 3.1 Hz, 1H), 8.11 – 8.06 (m, 2H), 7.77 (dd, J = 5.8, 3.3 Hz, 1H), 7.51 – 7.49 (m, 1H), 7.32 – 7.28 (m, 2H), 7.25 – 7.23 (m, 1H), 7.19 – 7.14 (m, 2H), 6.98 (s, 1H), 6.28 (s, 1H), 4.32 (t, J = 5.2 Hz, 2H), 2.48 – 2.38 (m, 1H), 2.14 (ddt, J = 13.8, 6.4, 5.0 Hz, 1H).

^{13}C NMR{^{1}H} (101 MHz, $CDCl_3$) δ 185.21, 168.29, 138.83, 134.10, 132.06, 128.92, 128.62, 126.66, 126.58, 126.10, 125.68, 121.83, 121.59, 117.40, 64.02, 41.29, 32.32.

EI-HRMS: m/z calculated for $C_{19}H_{16}O_2$ calcd. 276.1144, found 276.1141.

2-benzhydrylnaphthalene-1,4-diol (C-129):

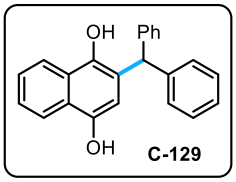

GP3 was followed using 1,4-naphthaquinone (0.3 mmol, 48 mg) and diphenylmethane (151 mg, 0.9 mmol). After 24 h, purification by column chromatography using 12% ethyl acetate in PE yielded **C-129** (52 mg, 53%) as a yellow sticky liquid.

^{1}H NMR (400 MHz, $CDCl_3$) δ 8.13 (t, *J* = 8.2 Hz, 1H), 8.09 (dd, *J* = 5.8, 3.3 Hz, 2H), 7.76 (dd, *J* = 5.8, 3.3 Hz, 2H), 7.58 – 7.49 (m, 2H), 7.40 – 7.32 (m, 2H), 7.25 – 7.17 (m, 2H), 7.14 – 7.08 (m, 2H), 6.97 (s, 2H), 6.52 – 6.25 (m, 3H).

^{13}C NMR{^{1}H} (101 MHz, $CDCl_3$) δ 156.6, 145.0, 128.7, 127.7, 126.9, 126.7, 126.4, 126.2, 125.4, 122.5, 121.4, 120.5, 103.8, 100.7, 57.8.

ESI-HRMS: m/z calculated for $C_{23}H_{19}O_2$ $[M+H]^+$ calcd. 327.1380, found 327.1383.

2-(2-phenylpropan-2-yl)naphthalene-1,4-diol (C-130):

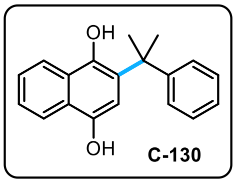

GP3 was followed using 1,4-naphthaquinone (0.3 mmol, 48 mg) and cumene (108 mg, 0.9 mmol). After 24 h, purification by column chromatography using 6% ethyl acetate in PE yielded **C-130** (77 mg, 92%) as a colourless sticky liquid.

^{1}H NMR (400 MHz, $CDCl_3$) δ 8.35 – 8.32 (m, 1H), 8.10 – 8.08 (m, 1H), 7.76 (dd, *J* = 5.8, 3.3 Hz, 1H), 7.58 – 7.55 (m, 2H), 7.38 (ddd, *J* = 7.7, 6.8, 1.3 Hz, 2H), 7.32 – 7.28 (m, 1H), 6.98 (s, 1H), 6.45 (d, *J* = 8.2 Hz, 1H), 6.19 (d, *J* = 8.2 Hz, 1H), 5.07 (s, 1H), 1.78 (s, 6H).

^{13}C NMR{^{1}H} (101 MHz, $CDCl_3$) δ 147.1, 145.6, 138.7, 134.0, 128.4, 127.0, 126.5, 125.7, 125.5, 125.4, 123.0, 121.4, 112.8, 107.7, 80.8, 29.3.

EI-HRMS: m/z calculated for $C_{19}H_{18}O_2$ calcd. 278.1292, found 278.1301.

2-(2-phenylbutan-2-yl)naphthalene-1,4-diol (C-131):

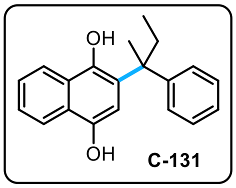

GP3 was followed using 1,4-naphthaquinone (0.3 mmol, 48 mg) and cumene (120 mg, 0.9 mmol). After 24 h, purification by column chromatography using 6% ethyl acetate in PE yielded **C-131** (47 mg, 54%) as a colourless sticky liquid.

^{1}H NMR (400 MHz, $CDCl_3$) δ 8.10 (d, *J* = 2.5 Hz, 2H), 7.76 (dd, *J* = 5.8, 3.3 Hz, 2H), 7.53 (t, *J* = 1.7 Hz, 1H), 7.51 (d, *J* = 1.0 Hz, 1H), 7.36 – 7.30 (m, 3H), 6.98 (s, 2H), 6.64 (s, 1H), 2.22 – 2.08 (m, 1H), 1.71 – 1.70 (m, 4H), 1.42 (d, *J* = 7.2 Hz, 3H).

^{13}C NMR{^{1}H} (101 MHz, $CDCl_3$) δ 163.5, 151.6, 138.8, 134.1, 132.1, 128.4, 128.4, 127.1, 127.0, 126.6, 126.0, 125.6, 124.5, 121.5, 49.0, 37.8, 23.2, 16.1.

ESI-HRMS: m/z calculated for $C_{20}H_{21}O_2$ $[M+H]^+$ calcd. 293.1536, found 293.1534.

2-(2-(4-fluorophenyl)propan-2-yl)naphthalene-1,4-diol (C-132):

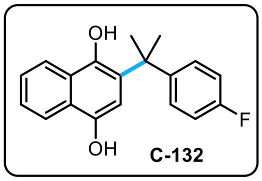

GP3 was followed using 1,4-naphthaquinone (0.3 mmol, 48 mg) and 1-fluoro-4-isopropylbenzene (124 mg, 0.9 mmol). After 24 h, purification by column chromatography using 6% ethyl acetate in PE yielded **C-132** (57 mg, 65%) as a colourless sticky liquid.

^{1}H NMR (400 MHz, $CDCl_3$) δ 8.53 – 8.49 (m, 1H), 8.32 (ddd, *J* = 6.6, 2.2, 0.7 Hz, 1H), 7.75 – 7.69 (m, 4H), 7.29 – 7.24 (m, 2H), 6.66 (d, *J* = 8.2 Hz, 1H), 6.38 (d, *J* = 8.2 Hz, 1H), 5.36 (s, 1H), 1.97 (s, 6H).

^{13}C NMR{^{1}H} (101 MHz, $CDCl_3$) δ 162.0 (d, $^1J_{C-F}$ = 245.4 Hz), 145.9, 145.5, 143.0 (d, $^4J_{C-F}$ = 3.2 Hz), 129.5, 127.3 (d, $^3J_{C-F}$ = 7.8 Hz), 125.9, 125.6, 125.4, 123.0, 121.6, 115.3 (d, $^2J_{C-F}$ = 21.2 Hz), 113.0, 107.7, 80.5, 29.5.

^{19}F NMR (376 MHz, $CDCl_3$) δ -118.60.

EI-HRMS: m/z calculated for $C_{19}H_{17}O_2F$ calcd. 296.1207, found 296.1201.

1-ethoxy-1-oxopropan-2-yl 4-(2-(1,4-dihydroxynaphthalen-2-yl)propan-2-yl)benzoate (C-133):

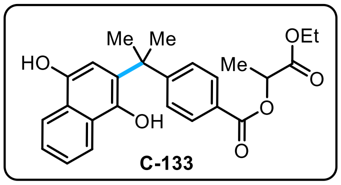

GP3 was followed using 1,4-naphthaquinone (0.3 mmol, 48 mg) and 1-ethoxy-1-oxopropan-2-yl 4-isopropylbenzoate (237 mg, 0.9 mmol). After 24 h, purification by column chromatography using 15% ethyl acetate in PE yielded **C-133** (70 mg, 55%) as a brown sticky liquid.

^{1}H NMR (400 MHz, $CDCl_3$) δ 8.11 – 8.09 (m, 2H), 8.01 (dd, *J* = 8.6, 1.8 Hz, 2H), 7.78 – 7.76 (m, 2H), 7.71 – 7.68 (m, 1H), 7.36 – 7.34 (m, 1H), 6.98 (s, 3H), 5.28 (q, *J* = 7.0 Hz, 1H), 4.22 (q, *J* = 7.2 Hz, 2H), 1.68 (s, 6H), 1.59 (d, *J* = 7.1 Hz, 3H), 1.27 (s, 3H).

^{13}C NMR{^{1}H} (101 MHz, $CDCl_3$) δ 185.2, 171.0, 165.8, 156.9, 153.5, 138.8, 134.5, 134.1, 133.8, 132.1, 130.3, 128.4, 127.2, 126.6, 126.0, 125.4, 69.2, 61.5, 42.8, 29.1, 28.5, 17.2, 14.3.

ESI-HRMS: m/z calculated for $C_{25}H_{30}NO_6$ $[M+NH_4]^+$ calcd. 440.2008, found 440.1984.

tert-butyl 2-(1,4-dihydroxynaphthalen-2-yl)-4-oxopiperidine-1-carboxylate (C-134):

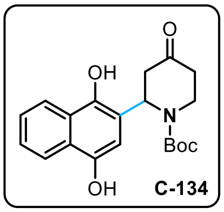

GP3 was followed using 1,4-naphthaquinone (0.3 mmol, 48 mg) and tert-butyl 4-oxopiperidine-1-carboxylate (179 mg, 0.9 mmol). After 24 h, purification by column chromatography using 40% ethyl acetate in PE yielded **C-134** (71 mg, 66%) as a brown solid.

^{1}H NMR (400 MHz, DMSO-d_6) δ 9.28 (s, 1H), 8.05 – 8.01 (m, 1H), 7.84 – 7.82 (m, 3H), 7.40 (dd, J = 6.4, 3.3 Hz, 1H), 6.65 (s, 1H), 5.19 – 5.17 (m, 1H), 3.90 – 3.86 (m, 1H), 3.62 – 3.59 (m, 1H), 2.47 – 2.43 (m, 2H), 2.36 – 2.33 (m, 2H), 1.48 (s, 9H).

^{13}C NMR{^{1}H} (101 MHz, DMSO-d_6) δ 207.5, 192.9, 153.8, 145.4, 144.0, 138.7, 134.2, 125.9, 124.6, 121.9, 107.8, 106.2, 82.6, 79.2, 40.4, 35.3, 28.0, 27.6.

ESI-HRMS: m/z calculated for $C_{20}H_{23}NO_5Na$ $[M+Na]^+$ calcd. 380.1468, found 380.1465.

2-(4-phenylmorpholin-3-yl)naphthalene-1,4-diol (C-135):

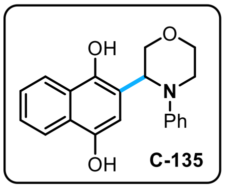

GP3 was followed using 1,4-naphthaquinone (0.3 mmol, 48 mg) and 4-phenylmorpholine (146 mg, 0.9 mmol). After 24 h, purification by column chromatography using 40% ethyl acetate in PE yielded **C-135** (52 mg, 54%) as a brown solid.

^{1}H NMR (400 MHz, DMSO-d_6) δ 8.01 – 7.99 (m, 1H), 7.90 – 7.88 (m, 1H), 7.22 – 7.16 (m, 2H), 7.13 (dtd, J = 8.9, 4.2, 0.8 Hz, 1H), 7.09 (s, 1H), 6.91 (s, 1H), 6.89 (s, 1H), 6.79 (d, J = 8.0 Hz, 1H), 6.73 – 6.63 (m, 3H), 4.60 (d, J = 3.5 Hz, 1H), 3.86 (ddd, J = 10.8, 7.1, 3.8 Hz, 2H), 3.69 – 3.62 (m, 2H), 2.84 (td, J = 11.9, 3.4 Hz, 1H), 2.66 (dt, J = 11.1, 3.0 Hz, 1H).

^{13}C NMR{^{1}H} (101 MHz, DMSO-d_6) δ 149.6, 147.2, 138.7, 134.2, 131.6, 129.3, 127.2, 125.9, 125.9, 124.0, 118.8, 117.4, 112.7, 112.1, 77.7, 69.4, 66.1, 52.2.

ESI-HRMS: m/z calculated for $C_{20}H_{20}NO_3$ $[M+Na]^+$ calcd. 322.1441, found 322.1438.

(1,4-dihydroxynaphthalen-2-yl)(2-fluorophenyl)methanone (C-137):

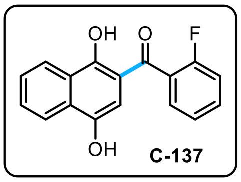

GP4 was followed using 1,4-naphthaquinone (7 mmol, 1.1 g) and 2-fluorobenzaldehyde (1.3 g, 10.5 mmol). After 24 h, purification by column chromatography using 12% ethyl acetate in PE yielded **C-137** (1.4 g, 72%) as a yellow solid.

^{1}H NMR (400 MHz, DMSO-d_6) δ 13.06 (s, 1H), 9.81 (s, 1H), 8.38 (d, J = 8.3 Hz, 1H), 8.13 (d, J = 8.3 Hz, 1H), 7.79 – 7.62 (m, 4H), 7.50 – 7.41 (m, 2H), 6.68 (d, J = 2.4 Hz, 1H).

^{13}C NMR{^{1}H} (101 MHz, DMSO-d_6) δ 197.5, 158.2 (d, $^{1}J_{C\text{-}F}$ = 248.1 Hz), 155.4, 145.0, 133.1 (d, $^{3}J_{C\text{-}F}$ = 8.3 Hz), 130.1, 129.8, 129.7 (d, $^{4}J_{C\text{-}F}$ = 3.0 Hz), 126.8, 126.3 (d, $^{2}J_{C\text{-}F}$ = 16.3 Hz), 125.0 (d, $^{3}J_{C\text{-}F}$ = 5.3 Hz), 124.9, 123.9, 122.3, 116.2 (d, $^{2}J_{C\text{-}F}$ = 21.0 Hz), 112.9, 105.6.

^{19}F NMR (376 MHz, DMSO-d_6) δ -113.71.

ESI-HRMS: m/z calculated for $C_{17}H_{12}FO_3$ $[M+H]^+$ calcd. 283.0765, found 283.0767.

2-benzoylnaphthalene-1,4-dione (D-1):[8]

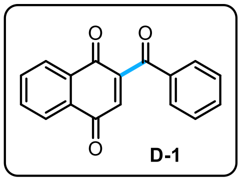

Following the literature,[8] (1,4-dihydroxynaphthalen-2-yl)(phenyl)methanone **C-1** (0.3 mmol, 79 mg) was dissolved in dry ether (5 mL). Afterwards, anhydrous Na_2SO_4 (142 mg, 1.5 mmol) was added, followed in small portions by Ag_2O (104 mg, 0.45 mmol). The suspension was stirred for 1 h at room temperature, filtered, and concentrated to a smaller volume. The residue was purified by column chromatography using 12% ethyl acetate in PE yielded **D-1** (78 mg, 99%) as a brown solid.

^{1}H NMR (400 MHz, $CDCl_3$/DMSO-d_6) δ 8.00 – 7.94 (m, 2H), 7.80 – 7.75 (m, 2H), 7.71 – 7.67 (m, 2H), 7.54 – 7.49 (m, 1H), 7.39 – 7.35 (m, 2H), 6.85 (s, 1H).

^{13}C NMR{^{1}H} (101 MHz, $CDCl_3$/DMSO-d_6) δ 191.7, 184.1, 183.0, 146.8, 135.4, 135.2, 134.4, 134.3, 134.3, 131.6, 131.2, 129.4, 128.7, 126.6, 126.2.

EI-HRMS: m/z calculated for $C_{17}H_{10}O_3$ calcd. 262.0623, found 262.0624.

2-(hydroxy(phenyl)methyl)naphthalene-1,4-dione (D-2):[9]

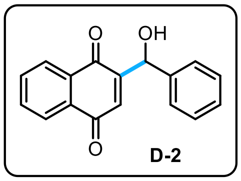

Following the literature,[7] (1,4-dihydroxynaphthalen-2-yl)(phenyl)methanone **C-1** (0.3 mmol, 79 mg) and sodium borohydride (46 mg, 1.2 mmol) were dissolved in methanol (5 mL). The resulting solution was stirred for 1 h at room temperature. After completion of the reaction, the solvent was evaporated, and the reaction was extracted by ethyl acetate. The organic layers were combined, dried over Na_2SO_4, and concentrated. The residue was purified by column chromatography using 15% ethyl acetate in PE yielded **D-2** (72 mg, 90%) as a brown solid.

^{1}H NMR (400 MHz, DMSO-d_6) δ 7.93 – 7.86 (m, 4H), 7.48 – 7.44 (m, 2H), 7.36 – 7.32 (m, 2H), 7.30 – 7.26 (m, 1H), 5.89 (d, J = 5.0 Hz, 1H), 5.47 (d, J = 4.9 Hz, 1H), 3.93 (s, 1H).

^{13}C NMR{^{1}H} (101 MHz, DMSO-d_6) δ 190.9, 190.4, 140.0, 135.1, 135.0, 132.0, 131.2, 128.1, 127.8, 127.5, 126.9, 126.5, 67.1, 64.8, 57.6.

6-hydroxy-3,4-diphenyl-2H-benzo[*h*]chromen-2-one (D-3):

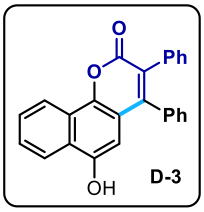

D-3

Following the literature,[7] (1,4-dihydroxynaphthalen-2-yl)(phenyl)methanone **C-1** (0.3 mmol, 79 mg) and phenylacetyl chloride (93 mg, 0.6 mmol) were dissolved in dry acetone (5 mL). Afterwards, anhydrous K_2CO_3 (149 mg, 1.08 mmol) was added and the suspension was refluxed for 6 h. Upon completion, the reaction mixture was cooled, and the solvent was removed under reduced pressure. Then the mixture was extracted with ethyl acetate (3× 10 mL). The extracts were washed with brine and dried over Na_2SO_4; the mixture was concentrated under vacuum, and the residue was purified by column chromatography using 15% ethyl acetate in PE yielded **D-3** (74 mg, 68%) as an orange solid.

^{1}H NMR (400 MHz, $CDCl_3$/DMSO-*d*$_6$) δ 9.64 (s, 1H), 8.54 – 8.45 (m, 1H), 8.21 (td, *J* = 8.2, 2.2 Hz, 1H), 7.62 – 7.54 (m, 2H), 7.22 – 7.09 (m, 10H), 6.54 (s, 1H).

^{13}C NMR{^{1}H} (101 MHz, $CDCl_3$/DMSO-*d*$_6$) δ 173.1, 161.0, 152.1, 149.4, 143.4, 135.0, 134.4, 134.1, 130.3, 129.0, 129.0, 128.0, 127.9, 127.7, 127.3, 127.0, 126.4, 123.4, 121.7, 115.5, 103.0.

ESI-HRMS: m/z calculated for $C_{25}H_{16}O_3$ $[M+H]^+$ calcd. 365.1172, found 365.1180.

5-hydroxy-7H-benzo[c]xanthen-7-one (D-4):[10]

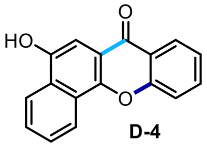

D-4

Following the literature,[7] (1,4-dihydroxynaphthalen-2-yl)(2-fluorophenyl)methanone **C-137** (0.3 mmol, 85 mg) and and K_2CO_3 (83 mg, 0.6 mmol), which were dissolved in acetone (5 mL) at room temperature. The reaction mixture was heated to 50 °C in an oil bath for 4 h; then, the resulting mixture was allowed to cool to room temperature and extracted with DCM. The extracted solution was then washed with brine and dried over anhydrous Na_2SO_4, and the mixture was concentrated under vacuum, and the residue was purified by column chromatography using 15% ethyl acetate in PE yielded **D-4** (66 mg, 84%) as an orange-yellow solid.

^{1}H NMR (400 MHz, $CDCl_3$/DMSO-*d*$_6$) δ 10.18 (s, 1H), 8.61 – 8.58 (m, 1H), 8.29 – 8.25 (m, 1H), 8.22 (dd, *J* = 8.0, 1.7 Hz, 1H), 7.71 – 7.68 (m, 4H), 7.42 (s, 1H).

^{13}C NMR{^{1}H} (101 MHz, $CDCl_3$/DMSO-*d*$_6$) δ 175.6, 155.0, 149.6, 146.9, 133.8, 128.6, 128.4, 126.9, 125.5, 124.5, 123.7, 122.5, 121.1, 117.8, 117.5, 99.4.

6-acetyl-5-hydroxydinaphtho[*1,2-b:2',3'-d*]furan-7,12-dione (D-5a):[11]

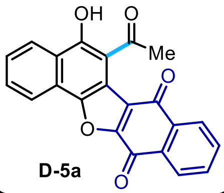

D-5a

Following the literature,[11] a mixture of 2,3-dichloro-1,4-naphthoquinone (75 mg, 0.33 mmol), 2-acetyl-1,4-dihydronaphthalene **C-41** (100 mg, 0.5 mmol), and K_2CO_3 (462 mg, 3.35 mmol) in pyridine (8 mL) was heated to 90 ºC for 16 h. The reaction mixture was cooled to room temperature, ice-cooled water (50 mL) was added, and

the mixture was extracted with DCM (100 mL). The organic layer was washed with hydrochloric acid solution (2 M; 2 x 100 mL) and brine (100 mL), dried over Na_2SO_4, and concentrated under reduced pressure. The residue was purified by column chromatography using 25% ethyl acetate in PE yielded **D-5a** (115 mg, 65%) as a bright red solid.

^{1}H NMR (400 MHz, DMSO-d_6) δ 10.43 (s, 1H), 8.38 – 8.30 (m, 2H), 8.12 – 8.04 (m, 2H), 7.88 – 7.79 (m, 3H), 7.75 (ddd, J = 8.3, 7.0, 1.4 Hz, 1H), 2.66 (s, 3H).

^{13}C NMR{^{1}H} (101 MHz, DMSO-d_6) δ 201.4, 180.2, 173.8, 152.9, 148.6, 147.2, 134.3, 134.1, 132.8, 132.0, 129.2, 128.0, 126.5, 126.3, 126.1, 124.5, 124.1, 120.9, 120.7, 117.7, 115.1, 32.4.

6-acetyl-5-(benzyloxy)dinaphtho[*1,2-b:2′,3′-d*]furan-7,12-dione (D-5b):[11]

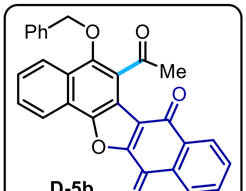

D-5b

Following the literature,[11] To a solution of phenol **D-5a** (90 mg, 0.25 mmol) in acetone (5 mL) were added K_2CO_3 (105 mg, 0.75 mmol) and benzyl bromide (128 mg, 0.75 mmol). The reaction mixture was heated to reflux for 4 h with constant stirring. The acetone was removed under reduced pressure, and the residue was diluted with dichloromethane (30 mL). The organic layer was washed with water (20 mL) and brine (20 mL), dried over Na_2SO_4, and concentrated in vacuo. The residue was purified by column chromatography using 20% ethyl acetate in PE yielded **D-5b** (76 mg, 57%) as a bright red solid.

^{1}H NMR (400 MHz, DMSO-d_6) δ 8.40 (d, J = 8.1 Hz, 1H), 8.20 (d, J = 8.3 Hz, 1H), 8.12 (d, J = 8.0 Hz, 2H), 7.90 – 7.79 (m, 4H), 7.58 – 7.54 (m, 2H), 7.51 – 7.41 (m, 3H), 5.05 (s, 2H), 2.66 (s, 3H).

^{13}C NMR{^{1}H} (101 MHz, DMSO-d_6) δ 200.9, 191.6, 180.7, 153.2, 150.5, 149.1, 142.1, 136.9, 135.0, 134.8, 133.1, 132.9, 132.5, 129.6, 129.1, 128.9, 128.8, 128.6, 128.4, 128.1, 127.4, 127.1, 126.8, 124.1, 121.7, 78.9, 33.4.

(1,4-dihydroxynaphthalen-2-yl)(phenyl-d_5)methanone:

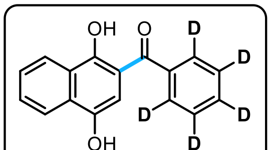

C-1'

GP3 was followed using 1,4-naphthaquinone (0.3 mmol, 48 mg) and benzaldehyde-d_6 (51 mg, 0.45 mmol). After 24 h, purification by column chromatography using 40% ethyl acetate in PE yielded **C-1'** (30 mg, 24%) as a yellow solid.

^{1}H NMR (400 MHz, DMSO-d$_6$) δ 13.06 (s, 1H), 9.79 (s, 1H), 8.37 (ddd, J = 8.3, 1.3, 0.7 Hz, 1H), 8.14 (ddd, J = 8.3, 1.3, 0.7 Hz, 1H), 7.73 (ddd, J = 8.3, 6.9, 1.3 Hz, 1H), 7.64 (ddd, J = 8.2, 6.9, 1.3 Hz, 1H), 6.91 (s, 1H).

ESI-HRMS: m/z calculated for $C_{17}H_8D_5O_3$ $[M+H]^+$ calcd. 270.1173, found 270.1167.

9. Computational studies:

9.1. Methodology:

Geometry optimizations and vibrational frequencies were conducted at the B3LYP/6-311++G** level of theory including the D3 dispersion correction. Solvents effects were considered implicitly by using the polarizable continuum model (PCM) but also explicitly by including one or two solvent molecules in the gas phase (discrete model) or coupled to PCM (hybrid discrete/continuum). The multiple minima hypersurface (MMH) approach was used to localize the minima of the **A-1**···H_2O complexes.[12] Over 100 randomly arranged complexes were generated by placing one/two molecules of water in a 5 Å box centered at certain binding positions of **A-1** and preoptimized with B3LYP/6-31+G(d,p). The most stable complexes were verified to be true minima by frequency analysis and then re optimized with B3LYP-D3/6-311++G** in both the gas phase and water PCM. All DFT calculations were performed using Gaussian 16, Rev. C01.[13]

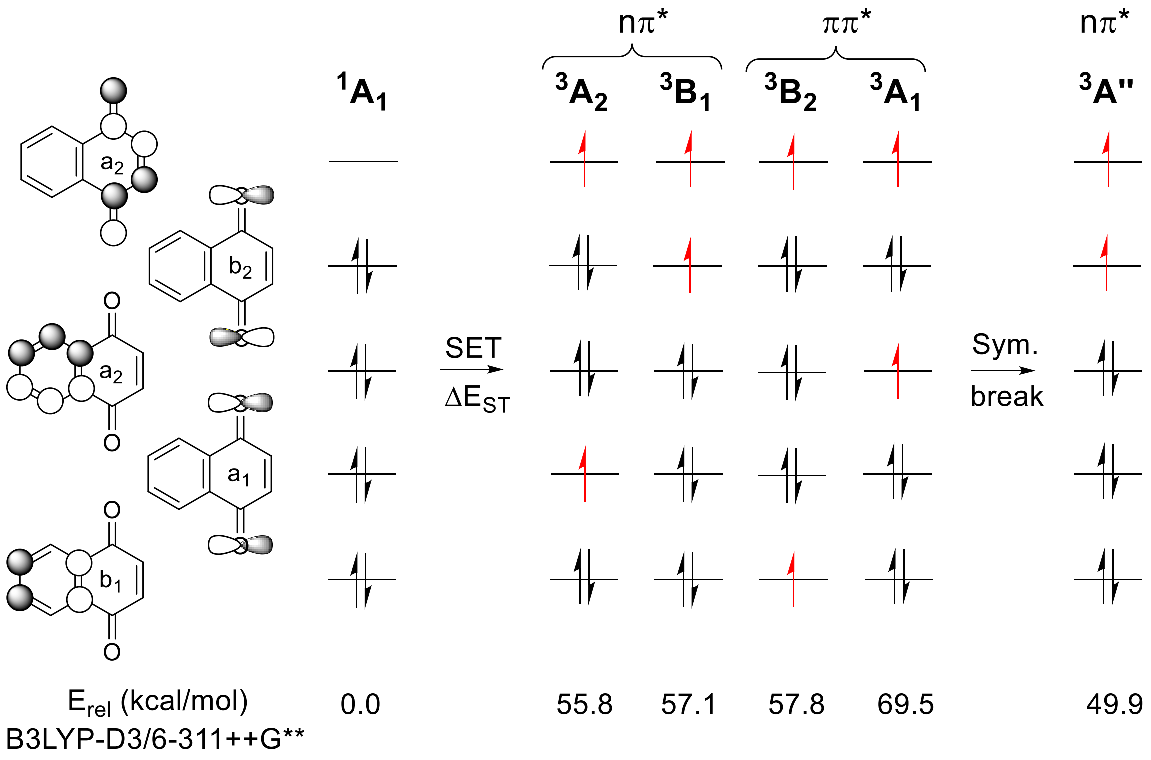

Fig. S7. Excited states of A-1: Electronic configuration, relevant molecular orbitals (MOs), and relative electronic energy (in kcal/mol) of the lowest-energy triplet states of 1,4-naphthoquinone **A-1**, as compared to the singlet ground state, calculated at the B3LYP-D3/6-311++G** level of theory and including ZPE correction.

9.2. Electronic structure and solvation of 1,4-naphthoquinone (A-1):

1,4-Naphthoquinone **A-1** exhibits a robust closed-shell singlet ground state with C2v symmetry and electronic configuration of $(b1)^2$ $(a1)^2$ $(a2)^2$ $(b2)^2$ $(a2)^0$ (Fig. S7). The presence of closely-lying molecular

orbitals (MOs) opens the door to a variety of accessible electronic excitations. Triplet states are relevant to this study because spin conservation governs triplet-triplet energy transfer from the photocatalyst to quinone **A-1**. Single electron transfers from the doubly occupied (b1, a1, a2, and b2) MOs into the empty a2 orbital (LUMO) leads to energetically close nπ* and ππ* triplet states, as predicted by DFT calculations. Interestingly, the triplet quinone undergoes a distortion from C2v to Cs symmetry, resulting in non-equivalent C=O moieties. Such geometric distortions have previously been reported in the excited states of quinones. Hence, we performed the subsequent calculations based on the lowest-energy triplet (3A'') state, albeit additional triplet states could also be populated during photosensitization of quinone **A-1**.

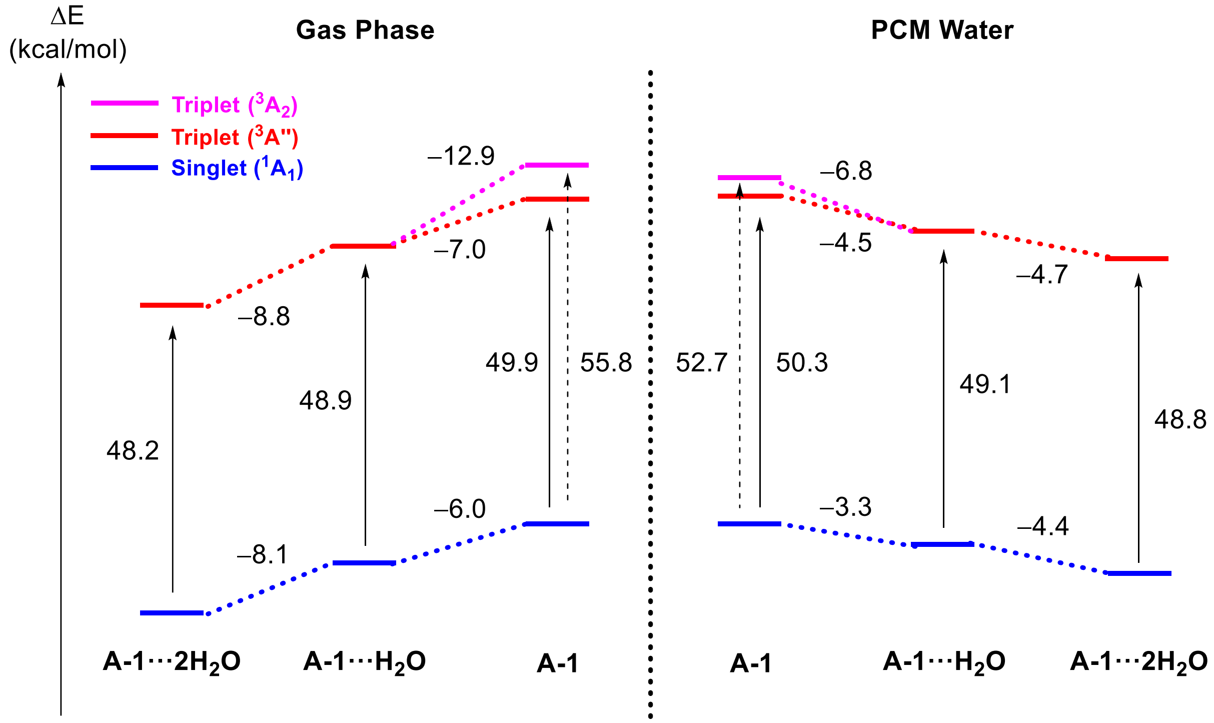

Fig. S8. Solvation of A-1: Energy diagram depicting the stabilization of both the lowest-energy singlet and triplet states of quinone **A-1** upon interaction with one and two explicit water molecules in the gas phase and implicit (water PCM) solvation models. Triplet energies are also derived using ZPE corrected energies at the B3LYP-D3/6-311++G** level of theory.

The energic difference between the excited triplet (^{3}A'') and the singlet ground state of quinone **A-1**, also known as triplet energy, does not change significantly when switching from vacuum to water solvation conditions, as estimated by DFT computations. Indeed, both states are stabilized to nearly the same extent by up to 15 kcal/mol through interaction with two water molecules (complexes A-1···2H_2O) leading to a strong hydrogen-bonding complex. A similar trend is observed in the solvation of the equilibrated ground and excited singlet states of quinone **A-1**, as evidenced by the closely bathochromic shifts of the absorption and emission maxima.

Hence, the enhancing effect of water in the photosensitization of the quinone is presumably due to a general solvating effect lowering the energy of its ground and excited states. Moreover, accessible higher-lying triplet states such as the $^3A_2$ state might scatter the triplet energy into a broad range of values, averaged in solution. On the other hand, the effect of water on the electronic structure of Eosin Y is more complex, as it may affect tautomeric equilibria and their individual electronics and goes beyond the scope of this work. Spectroscopic studies on the solvation of Eosin Y are cited herein.[14-17]

**Singlet A-1** ($^1A_1$)	**Triplet A-1** ($^3A''$)	**Triplet A-1** ($^3A_2$)

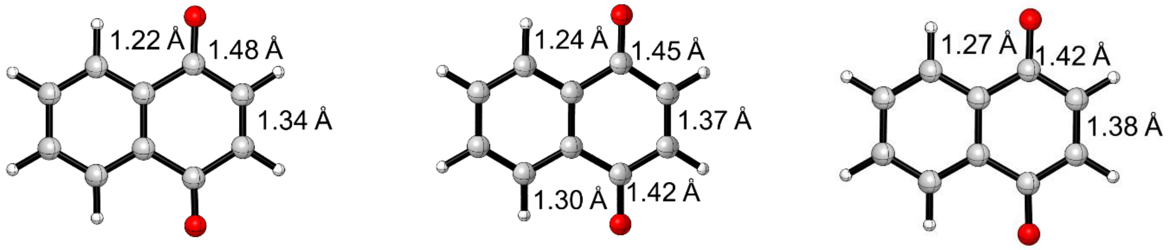

**Singlet A-1**···H_2O	**Triplet A-1**···H_2O

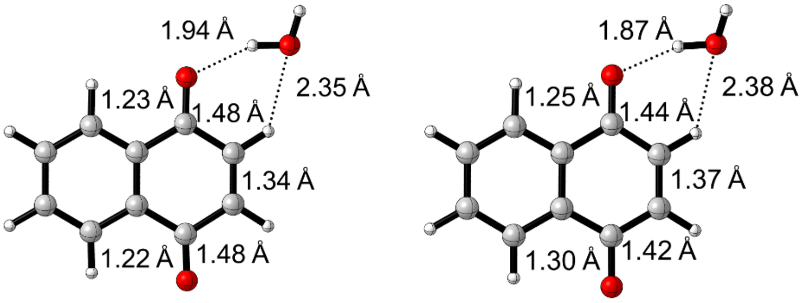

**Singlet A-1**···$2H_2O$	**Triplet A-1**···$2H_2O$

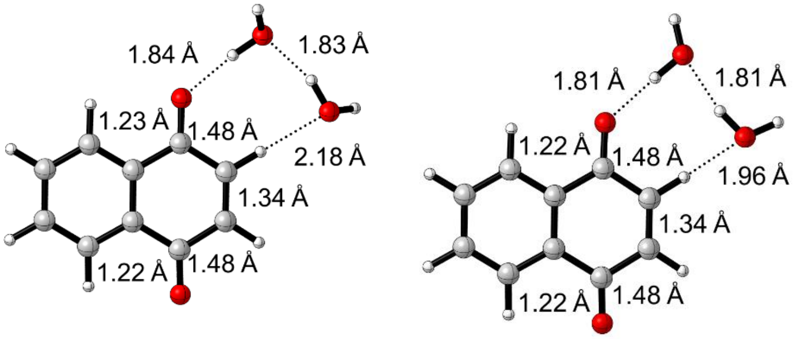

Fig. S9. Water complexes of A-1: Geometry and selected bond distances of the lowest-energy water complexes of 1,4-naphthoquinone **A-1**, optimized at the B3LYP-D3/6-311+G** level of theory.

9.3. Cartesian coordinates:

Geometries were optimized at the B3LYP-D3/6-311++G** level of theory.

Electronic energies (E) including the ZPE correction are given in Hartrees.

Singlet A-1 ($^1A_1$)

E = -535.140181

C	0.00000000	0.69869192	2.67526815
C	0.00000000	1.39816841	1.47273471
C	0.00000000	0.70338800	0.26124971
C	0.00000000	-0.70338800	0.26124971
C	0.00000000	-1.39816841	1.47273471
C	0.00000000	-0.69869192	2.67526815
H	0.00000000	1.23994684	3.61443961
H	0.00000000	2.48112859	1.44981737
C	0.00000000	1.46149608	-1.02419823
C	0.00000000	-1.46149608	-1.02419823
H	0.00000000	-2.48112859	1.44981737
H	0.00000000	-1.23994684	3.61443961
C	0.00000000	-0.66998876	-2.27980401
C	0.00000000	0.66998876	-2.27980401
H	0.00000000	-1.25064040	-3.19588192
H	0.00000000	1.25064040	-3.19588192
O	0.00000000	-2.68098989	-1.06243174
O	0.00000000	2.68098989	-1.06243174

Triplet A-1 ($^3A''$)

E = -535.060736

C	2.63812130	0.76401377	0.00000000
C	1.42128151	1.42559265	0.00000000
C	0.21550796	0.70951480	0.00000000
C	0.26319446	-0.70182892	0.00000000
C	1.50536172	-1.37084263	0.00000000
C	2.67831780	-0.63971442	0.00000000
H	3.56216287	1.33050854	0.00000000
H	1.36117975	2.50746732	0.00000000
C	-1.07341980	1.44330135	0.00000000
C	-0.99196918	-1.43779189	0.00000000
H	1.53020500	-2.45420225	0.00000000
H	3.63201508	-1.15490462	0.00000000
C	-2.23154873	-0.74968511	0.00000000
C	-2.26605869	0.62333073	0.00000000
H	-3.13766236	-1.34099123	0.00000000
H	-3.21523564	1.14576799	0.00000000
O	-0.96447259	-2.73517062	0.00000000
O	-1.12120175	2.68154716	0.00000000

Triplet A-1 ($^3A_2$)

E = -535.051304

C	0.00000000	0.70195700	2.66229873
C	0.00000000	1.40193000	1.46904073
C	0.00000000	0.71383800	0.24150973
C	0.00000000	-0.71383800	0.24150973
C	0.00000000	-1.40193000	1.46904073
C	0.00000000	-0.70195700	2.66229873
H	0.00000000	1.24124300	3.60240473
H	0.00000000	2.48398100	1.46457373
C	0.00000000	1.42971500	-1.02899027
C	0.00000000	-1.42971500	-1.02899027
H	0.00000000	-2.48398100	1.46457373
H	0.00000000	-1.24124300	3.60240473
C	0.00000000	-0.69050000	-2.24621627

C	-0.00000000	0.69050000	-2.24621627				
H	0.00000000	-1.24658900	-3.17672027				
H	-0.00000000	1.24658900	-3.17672027				
O	0.00000000	-2.69808600	-1.05951427				
O	-0.00000000	2.69808600	-1.05951427				
**Singlet A-1**···H_2O<br>E = -611.587053				**Triplet A-1**···H_2O<br>E = -611.509197			
C	2.24497584	-2.12433793	-0.00412801	C	2.19201898	-2.14588698	-0.00393316
C	0.86596985	-1.94077284	0.00205296	C	0.82306198	-1.93637501	0.00146092
C	0.33406194	-0.64899680	0.00457592	C	0.29628996	-0.63617002	0.00472495
C	1.19571001	0.46296714	0.00040591	C	1.19053294	0.45927200	0.00117089
C	2.57805300	0.27031905	-0.00570107	C	2.58387994	0.23904402	-0.00420219
C	3.10034591	-1.01962499	-0.00791003	C	3.07416096	-1.05326497	-0.00670422
H	2.65583577	-3.12734396	-0.00625198	H	2.58457300	-3.15608798	-0.00648718
H	0.18567979	-2.78346079	0.00480297	H	0.12232599	-2.76239602	0.00311797
C	-1.14194005	-0.45716871	0.01101890	C	-1.16632105	-0.43309104	0.01028004
C	0.64129710	1.84911218	0.00176487	C	0.63435491	1.80141699	0.00221292
H	3.22314705	1.14045100	-0.00887808	H	3.26097793	1.08492304	-0.00699723
H	4.17425990	-1.16677606	-0.01281101	H	4.14495497	-1.22114095	-0.01137428
C	-0.83535389	1.98893327	0.00615984	C	-0.76831809	2.00646196	0.00659501
C	-1.65998496	0.93157333	0.00961186	C	-1.62890607	0.93530495	0.00940606
H	-1.20637382	3.00802530	0.00601581	H	-1.13272411	3.02521496	0.00660403
H	-2.73972295	1.03659240	0.01440684	H	-2.70163207	1.09589293	0.01507613
O	1.35828317	2.83600213	-0.00048315	O	1.43851990	2.82010200	-0.00037913
O	-1.91134812	-1.41247666	0.01599491	O	-1.95228803	-1.40232806	0.01289909
H	-3.80623809	-1.00639753	-0.01515613	H	-3.76244604	-0.92717909	-0.03794080
O	-4.58391505	-0.42577248	-0.05645915	O	-4.56768505	-0.37750010	-0.07118276
H	-5.32955609	-0.95513643	0.23834385	H	-5.24893102	-0.88168911	0.38123629
**Singlet A-1**···$2H_2O$<br>E = -688.037147				**Triplet A-1**···$2H_2O$<br>E = -687.960385			
C	2.81346088	-2.05372822	-0.01693399	C	2.81992396	-2.02295804	-0.02020712
C	1.42883589	-1.91839209	-0.02171899	C	1.44027997	-1.90347900	-0.03162908
C	0.85180902	-0.64598703	-0.01045389	C	0.82876400	-0.64059198	-0.01785706
C	1.67421313	0.49512988	0.00527421	C	1.64996903	0.51068099	0.00713291
C	3.06211611	0.35106075	0.01045521	C	3.05455503	0.38200796	0.01917787

C	3.62945799	-0.91988331	-0.00054489	C	3.62866500	-0.87524906	0.00553186
H	3.25916878	-3.04169827	-0.02621706	H	3.27775894	-3.00524905	-0.03153713
H	0.77908281	-2.78467802	-0.03503906	H	0.79612894	-2.77397598	-0.05229406
C	-0.62951197	-0.50408989	-0.01553989	C	-0.64293500	-0.53227794	-0.03041002
C	1.07022226	1.86093494	0.01539232	C	1.00696307	1.81320401	0.01967693
H	3.67616120	1.24330669	0.02285929	H	3.67461706	1.27030594	0.03848985
H	4.70788598	-1.02930741	0.00327211	H	4.70811299	-0.97276409	0.01434982
C	-0.40965573	1.94612809	0.00625131	C	-0.40669393	1.92394905	0.00384597
C	-1.19882684	0.86106217	-0.01121078	C	-1.19596896	0.79927107	-0.02290401
H	-0.82072063	2.94969513	0.01419739	H	-0.84141590	2.91464606	0.01414898
H	-2.28207183	0.94830827	-0.01980678	H	-2.27644496	0.91541610	-0.03699697
O	1.75289136	2.87183888	0.02957340	O	1.74012010	2.88224799	0.04565390
O	-1.35931807	-1.49272682	-0.02140297	O	-1.35797502	-1.55822392	-0.04542999
H	-3.19025708	-1.64577964	-0.03291400	H	-3.13040902	-1.62939488	-0.01986094
O	-4.16627208	-1.62281654	-0.01146201	O	-4.11037002	-1.58622785	0.03340909
H	-4.43103515	-2.23606757	0.67982394	H	-4.36236302	-2.18135184	0.74477110
H	-4.54681591	0.16380549	0.02973213	H	-4.49463297	0.17801616	0.04948509
O	-4.45282381	1.13608048	0.02853621	O	-4.44593995	1.15491716	0.01209509
H	-5.09994177	1.45999560	-0.60368876	H	-5.05916596	1.41530217	-0.68052989

10. NMR spectra of the synthesized compound

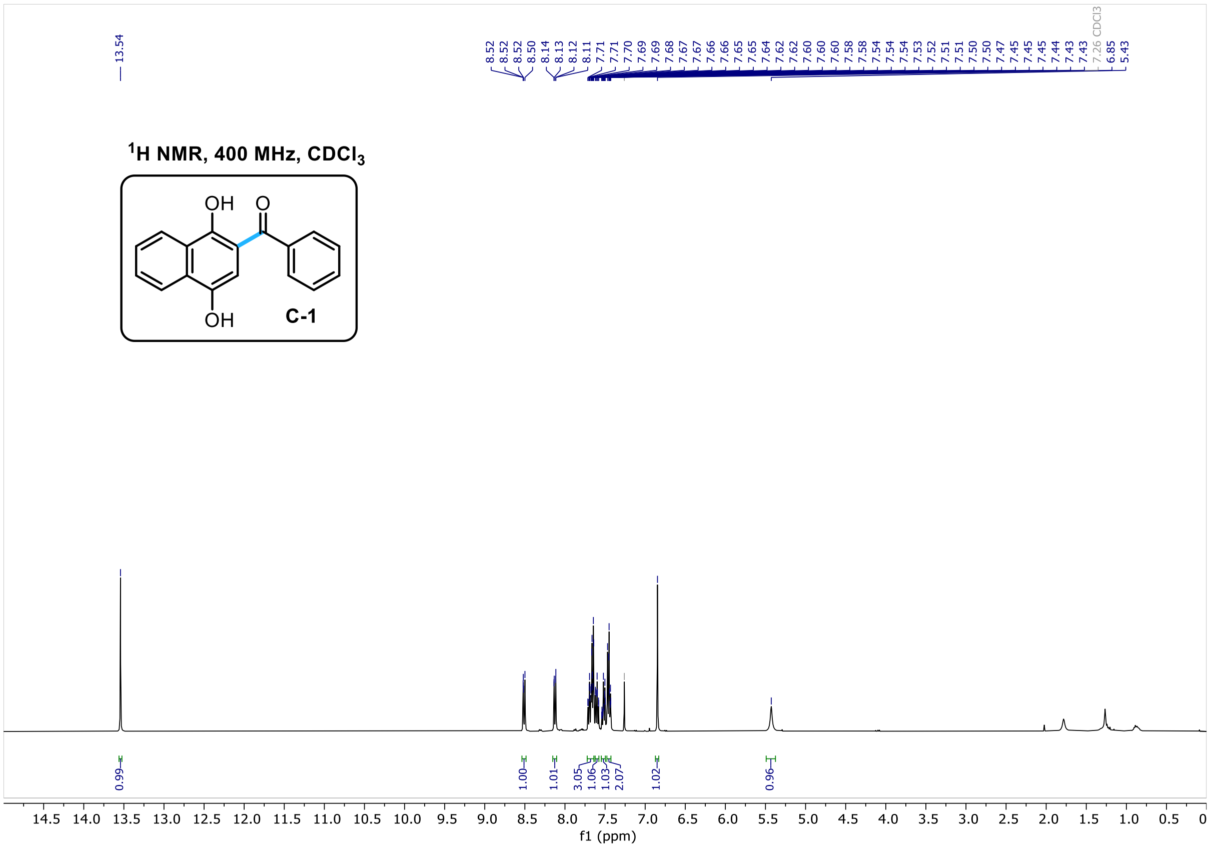

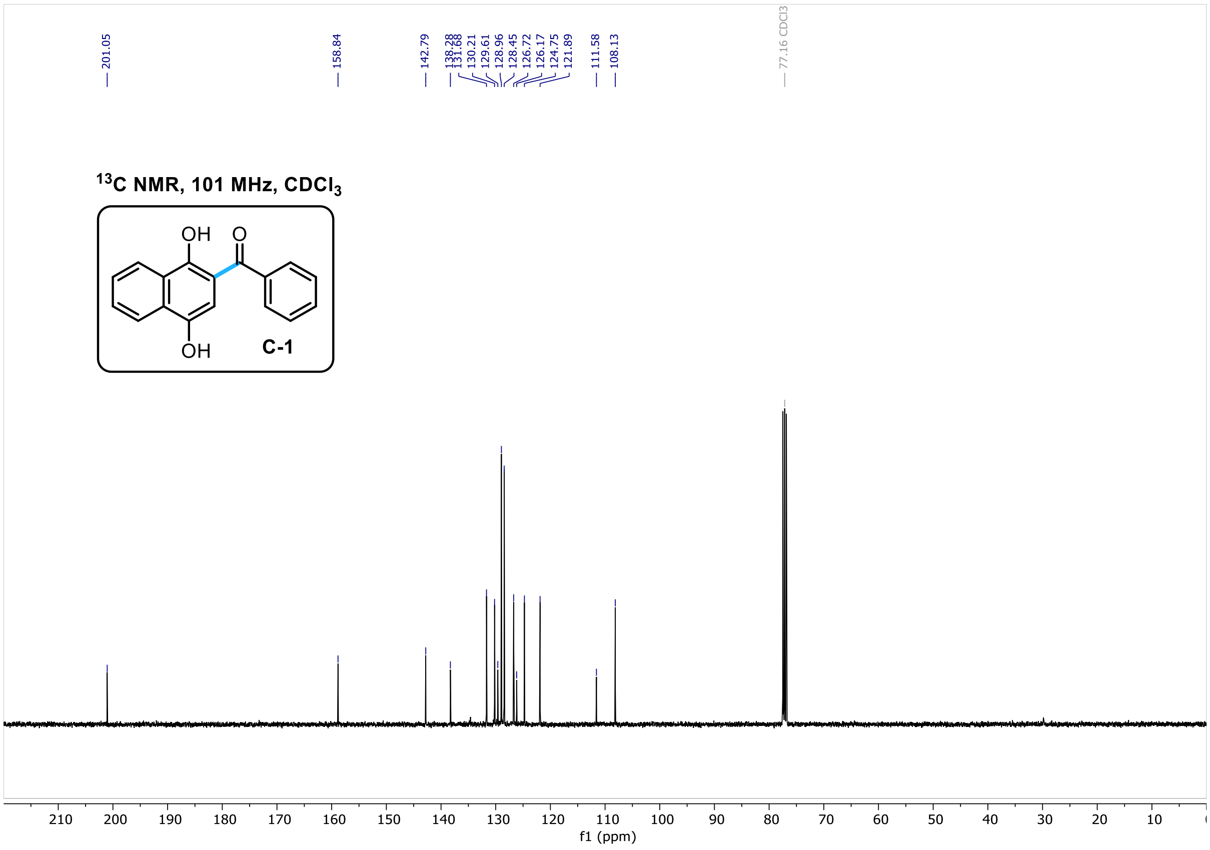

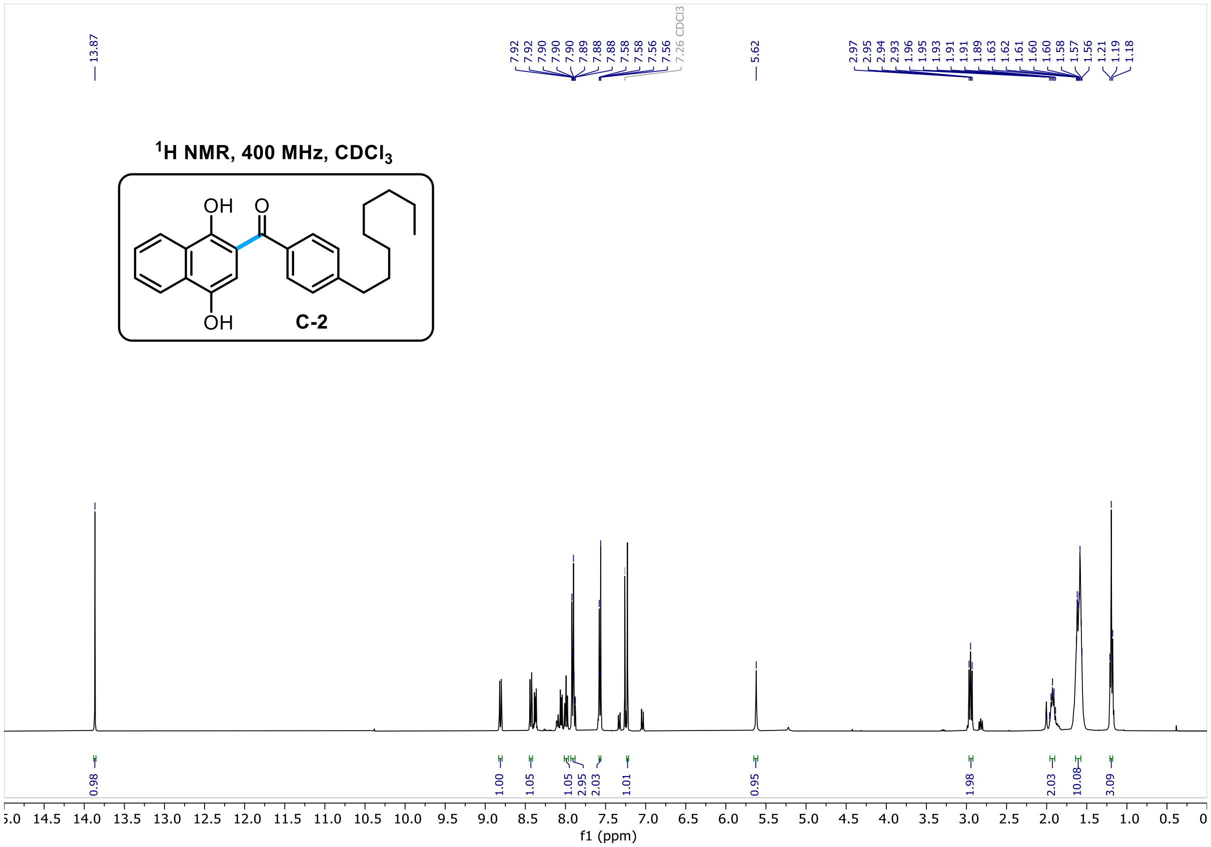
1H NMR, 400 MHz, CDCl3
OH
O
OH
C-2
f1 (ppm)

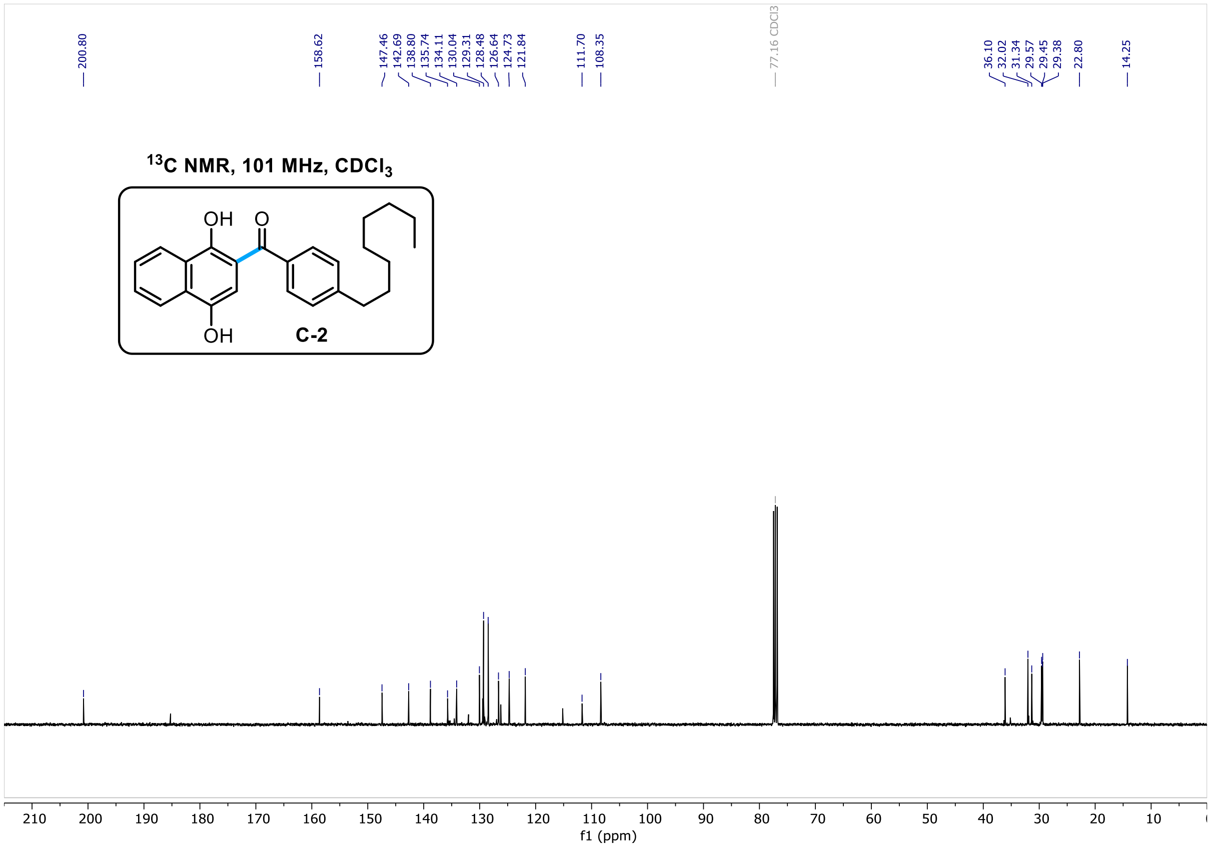
13C NMR, 101 MHz, CDCl3
OH
O
OH
C-2
f1 (ppm)

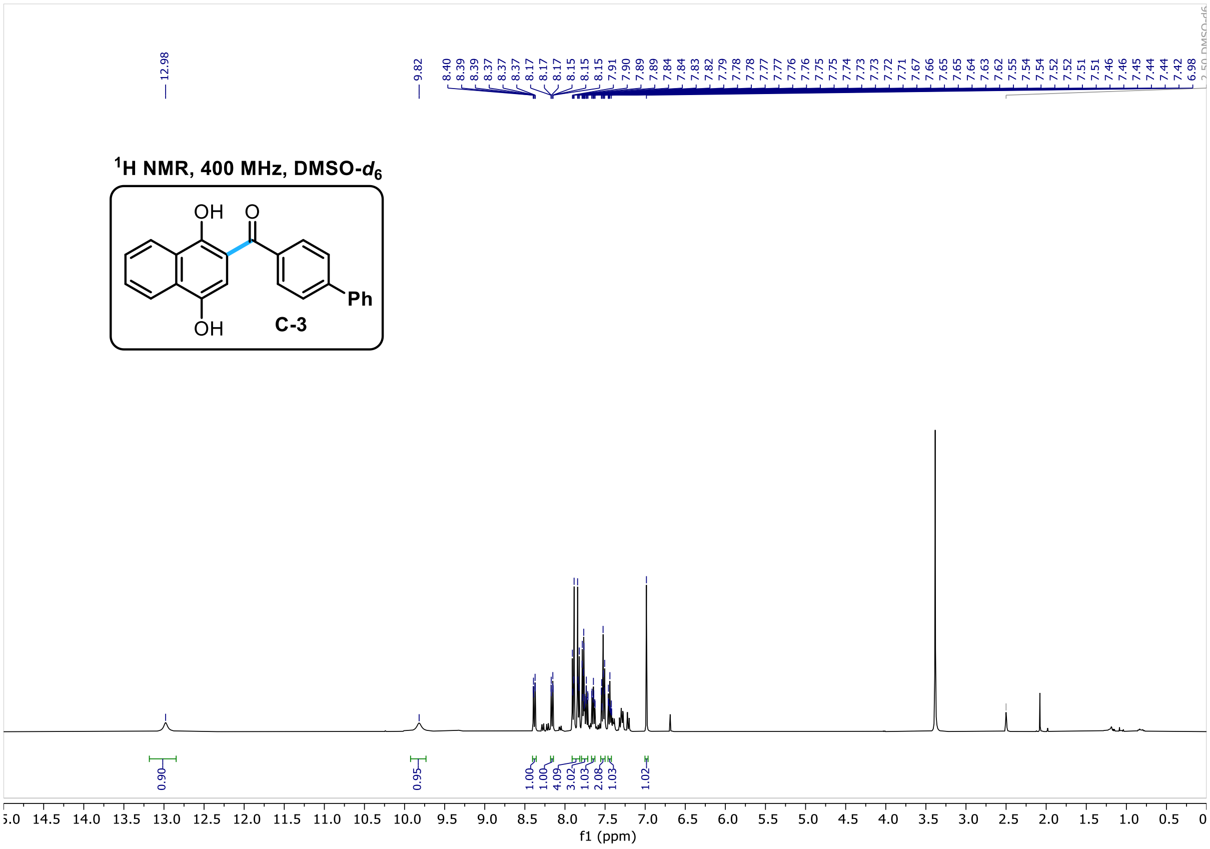
12.98
9.82
8.40 8.39 8.39 8.37 8.37 8.37 8.17 8.17 8.17 8.15 8.15 8.15 7.91 7.90 7.89 7.89 7.84 7.83 7.82 7.79 7.78 7.78 7.77 7.77 7.76 7.75 7.75 7.74 7.73 7.73 7.72 7.71 7.67 7.66 7.65 7.65 7.64 7.63 7.62 7.55 7.54 7.54 7.52 7.52 7.51 7.51 7.46 7.45 7.44 7.44 7.42 6.98
1H NMR, 400 MHz, DMSO-d6
OH
O
OH
Ph
C-3
0.90
0.95
1.00
1.00
4.09
3.02
1.03
2.08
1.03
1.02
f1 (ppm)

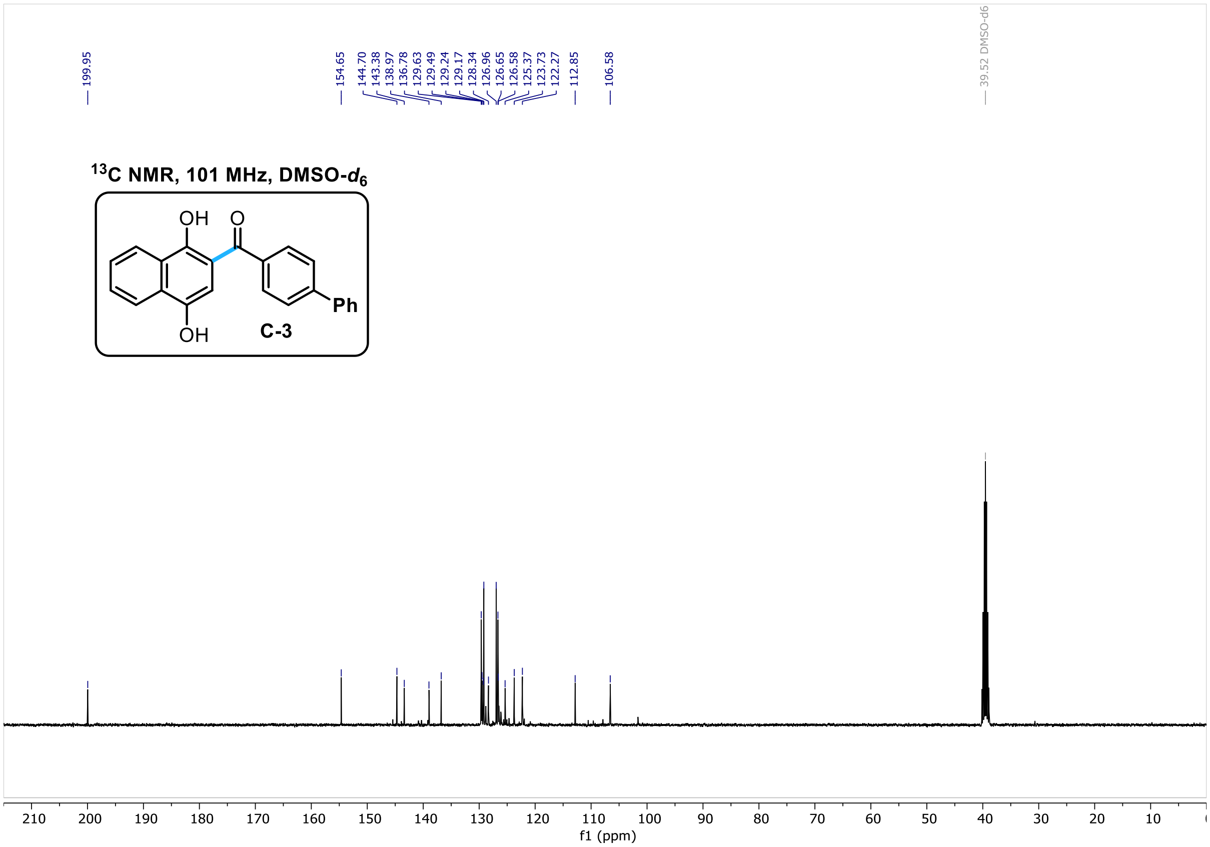
199.95
154.65 144.70 143.38 138.97 136.78 129.63 129.49 129.24 129.17 128.34 126.96 126.65 126.58 125.37 123.73 122.27
112.85
106.58
39.52 DMSO-d6
13C NMR, 101 MHz, DMSO-d6
OH
O
OH
Ph
C-3
f1 (ppm)

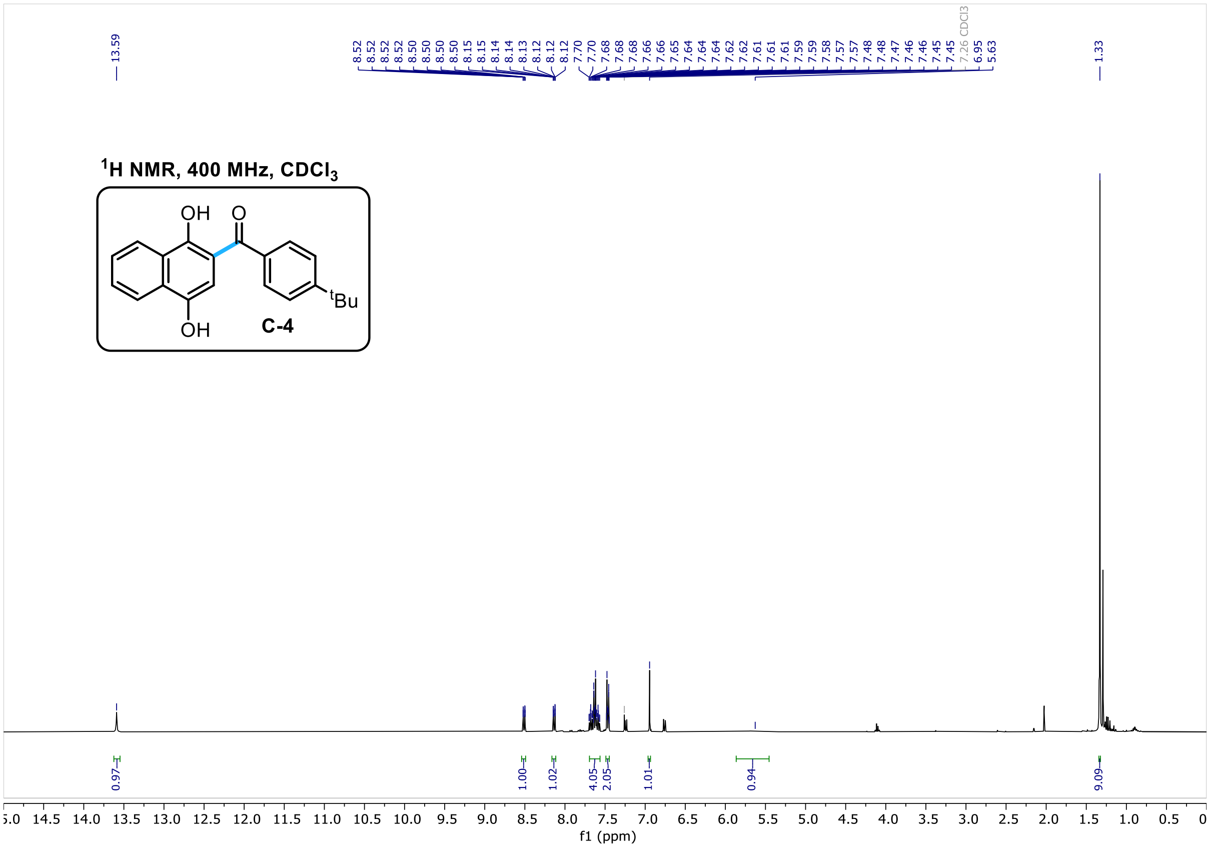
13.59
8.52 8.52 8.52 8.52 8.50 8.50 8.50 8.50 8.15 8.15 8.14 8.14 8.13 8.12 8.12 8.12 7.70 7.70 7.68 7.68 7.66 7.66 7.65 7.64 7.64 7.62 7.62 7.61 7.61 7.61 7.59 7.59 7.58 7.57 7.57 7.48 7.48 7.47 7.46 7.46 7.45 7.45 7.26 CDCl3 6.95 5.63
1.33
¹H NMR, 400 MHz, CDCl3
OH
O
OH
tBu
C-4
0.97
1.00
1.02
4.05
2.05
1.01
0.94
9.09
f1 (ppm)

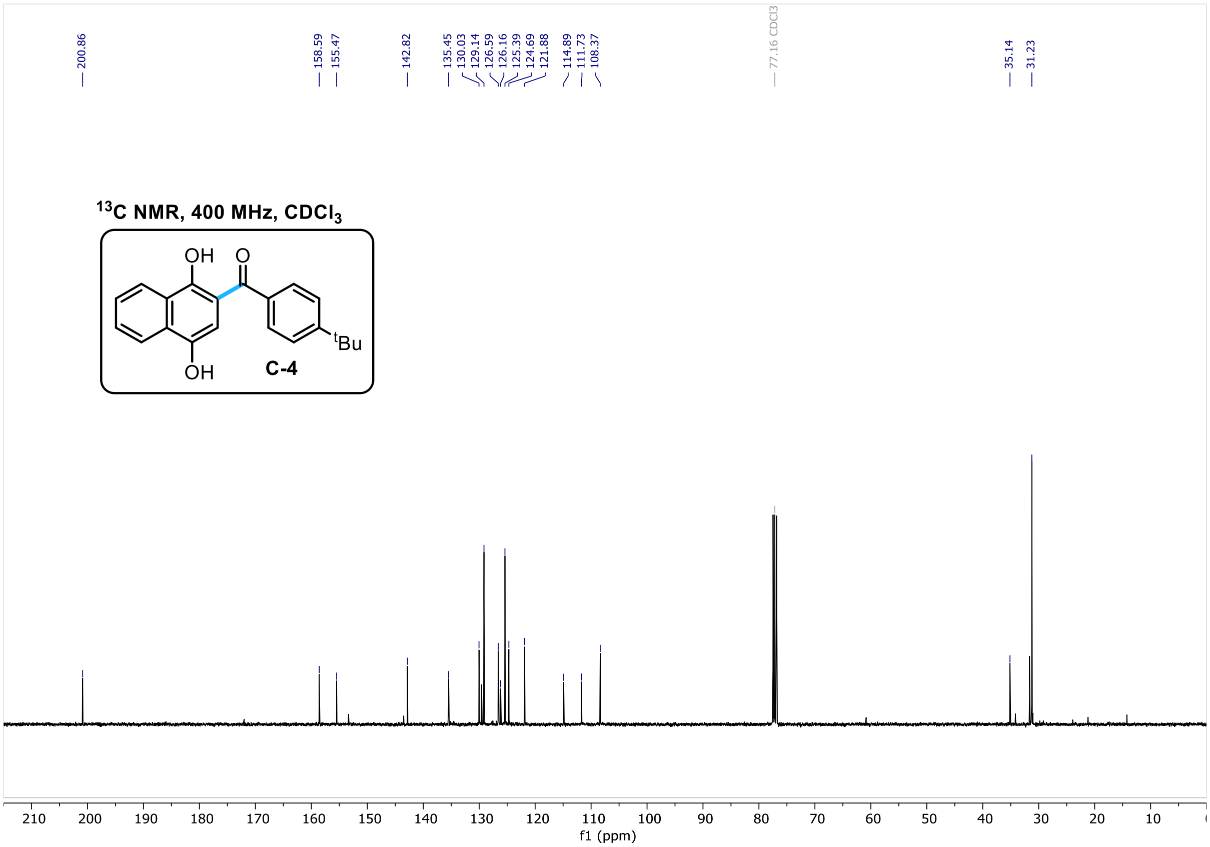
200.86
158.59
155.47
142.82
135.45 130.03 129.14 126.59 126.16 125.39 124.69 121.88
114.89 111.73 108.37
77.16 CDCl3
35.14
31.23
¹³C NMR, 400 MHz, CDCl3
OH
O
OH
tBu
C-4
f1 (ppm)

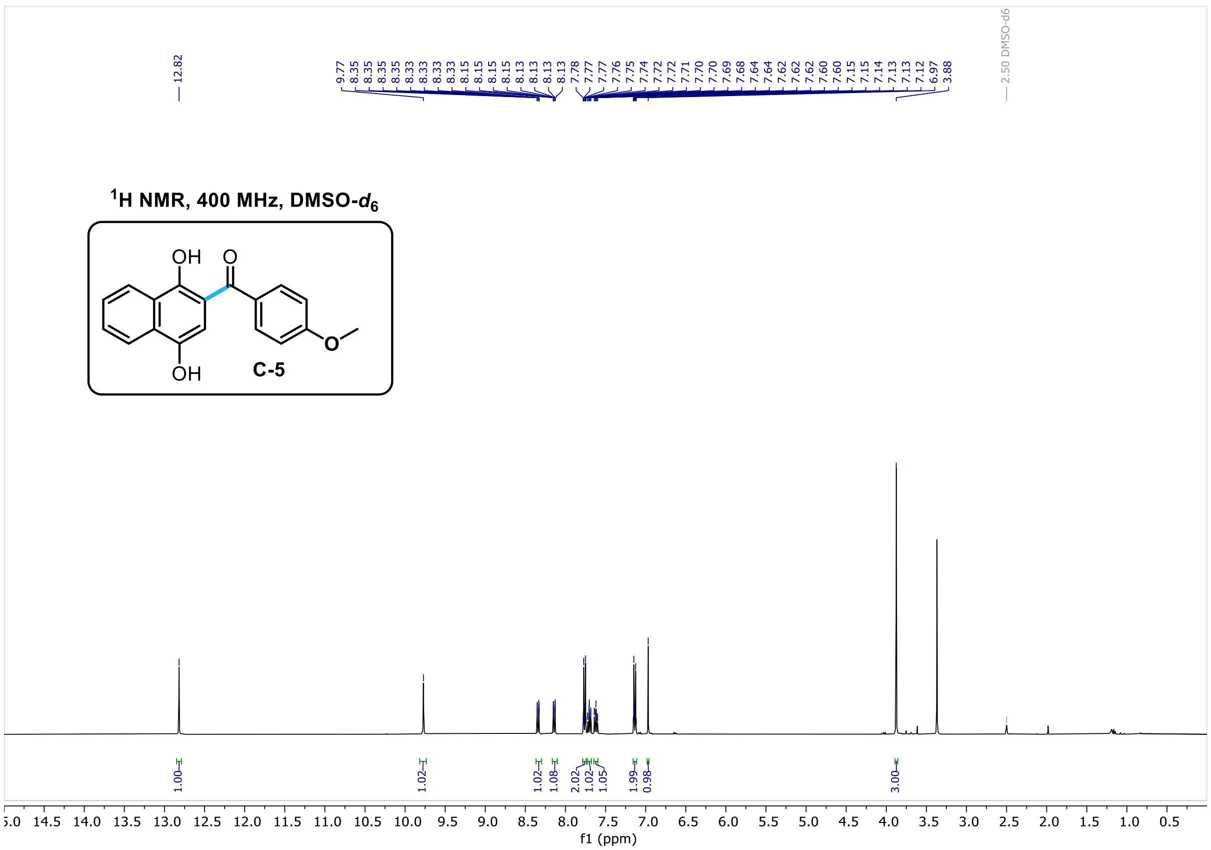
12.82
9.77 8.35 8.35 8.35 8.35 8.33 8.33 8.33 8.33 8.15 8.15 8.15 8.15 8.13 8.13 8.13 7.78 7.77 7.77 7.76 7.75 7.74 7.72 7.72 7.71 7.70 7.70 7.69 7.68 7.64 7.62 7.62 7.60 7.60 7.15 7.15 7.14 7.13 7.13 7.12 6.97 3.88
2.50 DMSO-d6
1H NMR, 400 MHz, DMSO-d6
OH
O
OH
C-5
1.00
1.02
1.02
1.08
2.02
1.02
1.05
1.99
0.98
3.00
f1 (ppm)

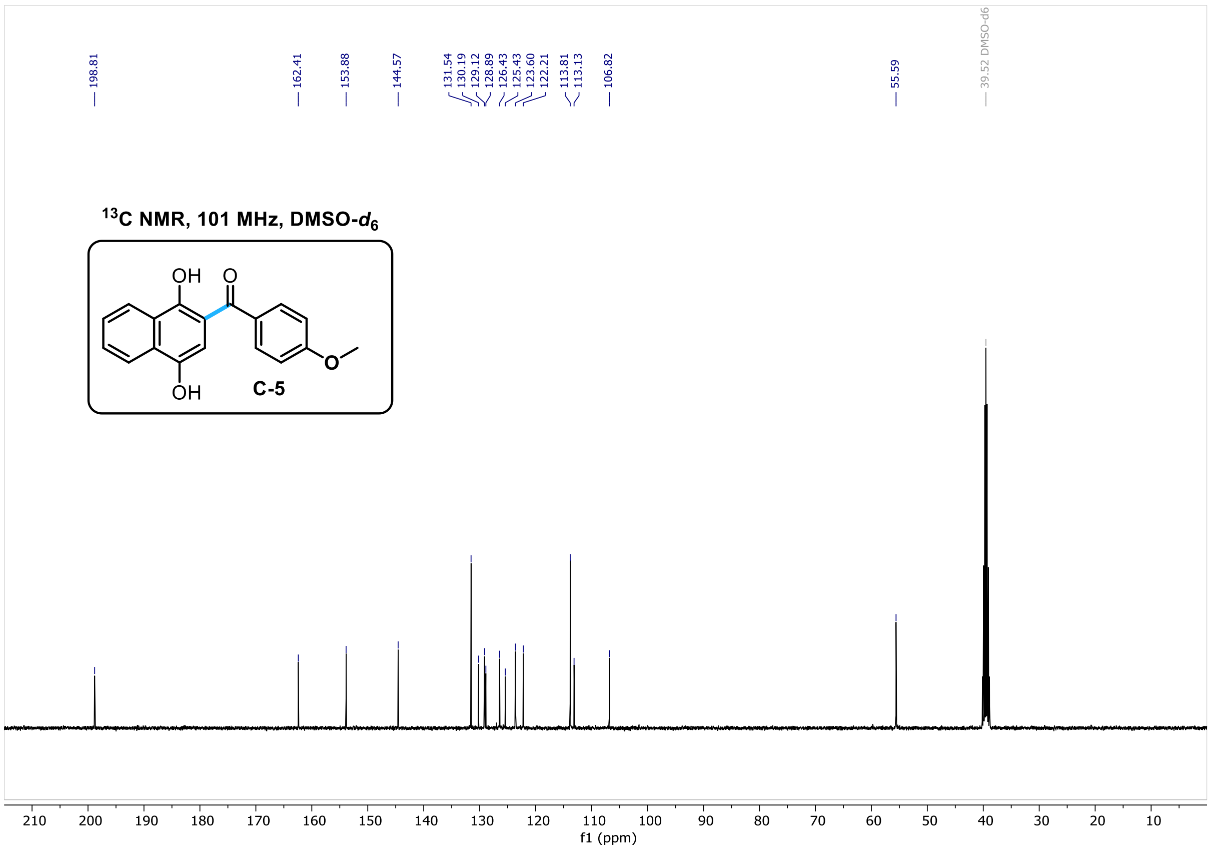
198.81
162.41
153.88
144.57
131.54 130.19 129.12 128.89 126.43 125.43 123.60 122.21
113.81 113.13
106.82
55.59
39.52 DMSO-d6
13C NMR, 101 MHz, DMSO-d6
OH
O
OH
C-5
f1 (ppm)

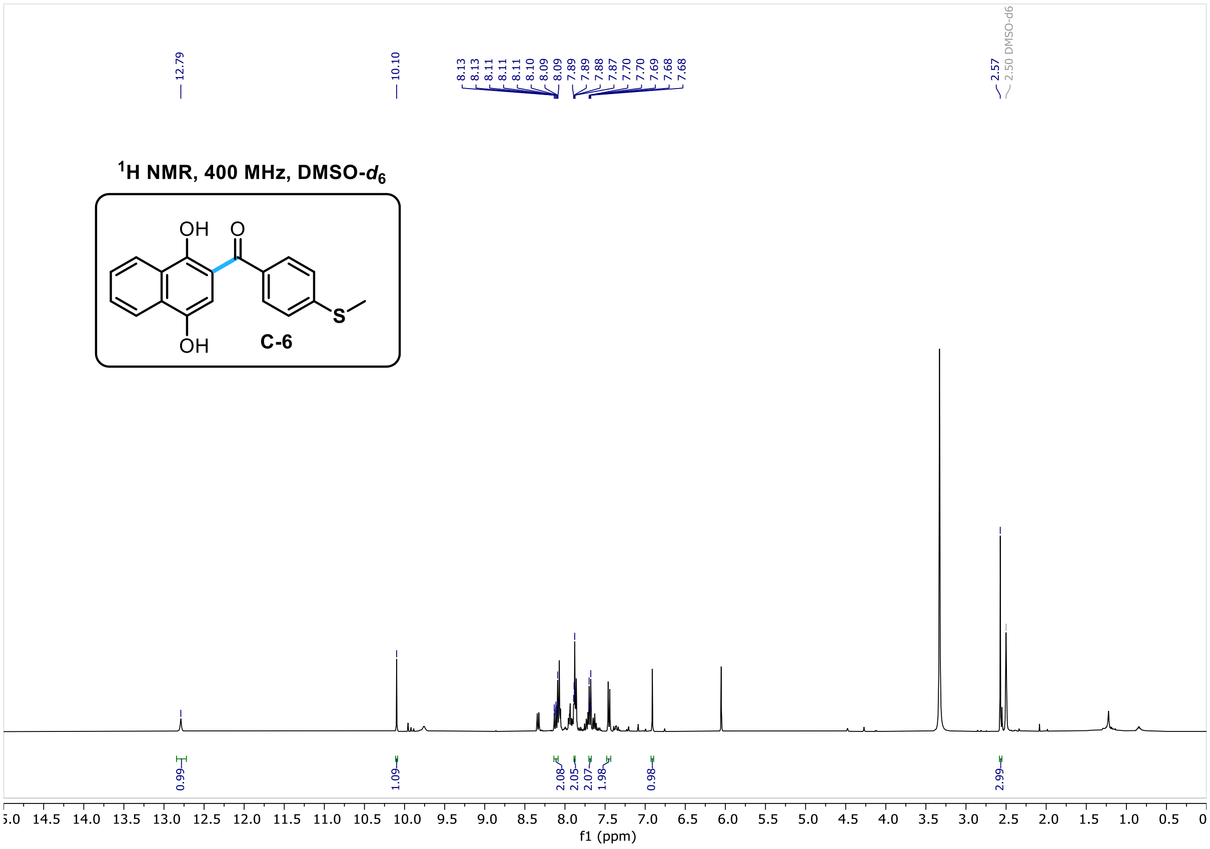

^{1}H NMR, 400 MHz, DMSO-d_6
OH
O
OH
S
C-6
12.79
10.10
8.13 8.13 8.11 8.11 8.10 8.09 8.09 7.89 7.89 7.88 7.87 7.70 7.70 7.69 7.68 7.68
2.57
2.50 DMSO-d6
0.99
1.09
2.08
2.05
2.07
1.98
0.98
2.99
f1 (ppm)

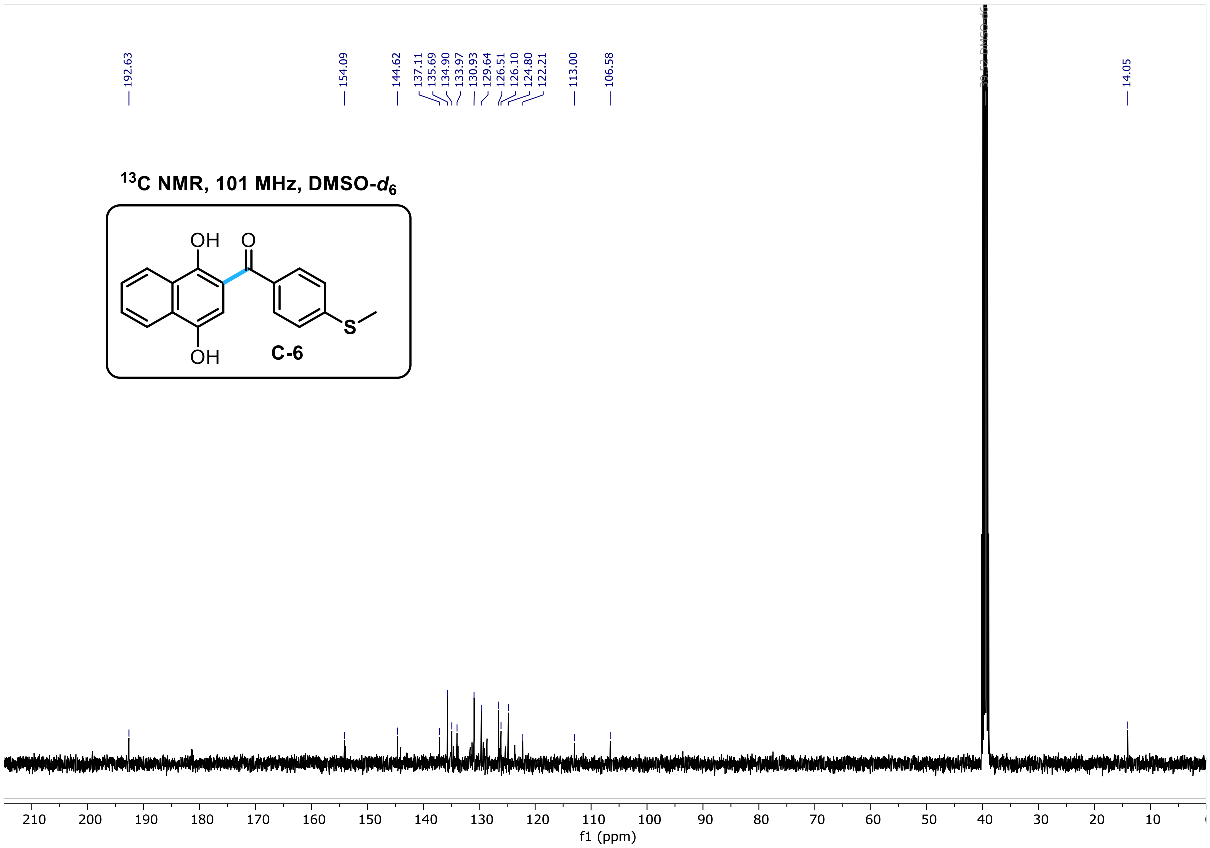

^{13}C NMR, 101 MHz, DMSO-d_6
OH
O
OH
S
C-6
192.63
154.09
144.62
137.11 135.69 134.90 133.97 130.93 129.64 126.51 126.10 124.80 122.21
113.00
106.58
14.05
f1 (ppm)

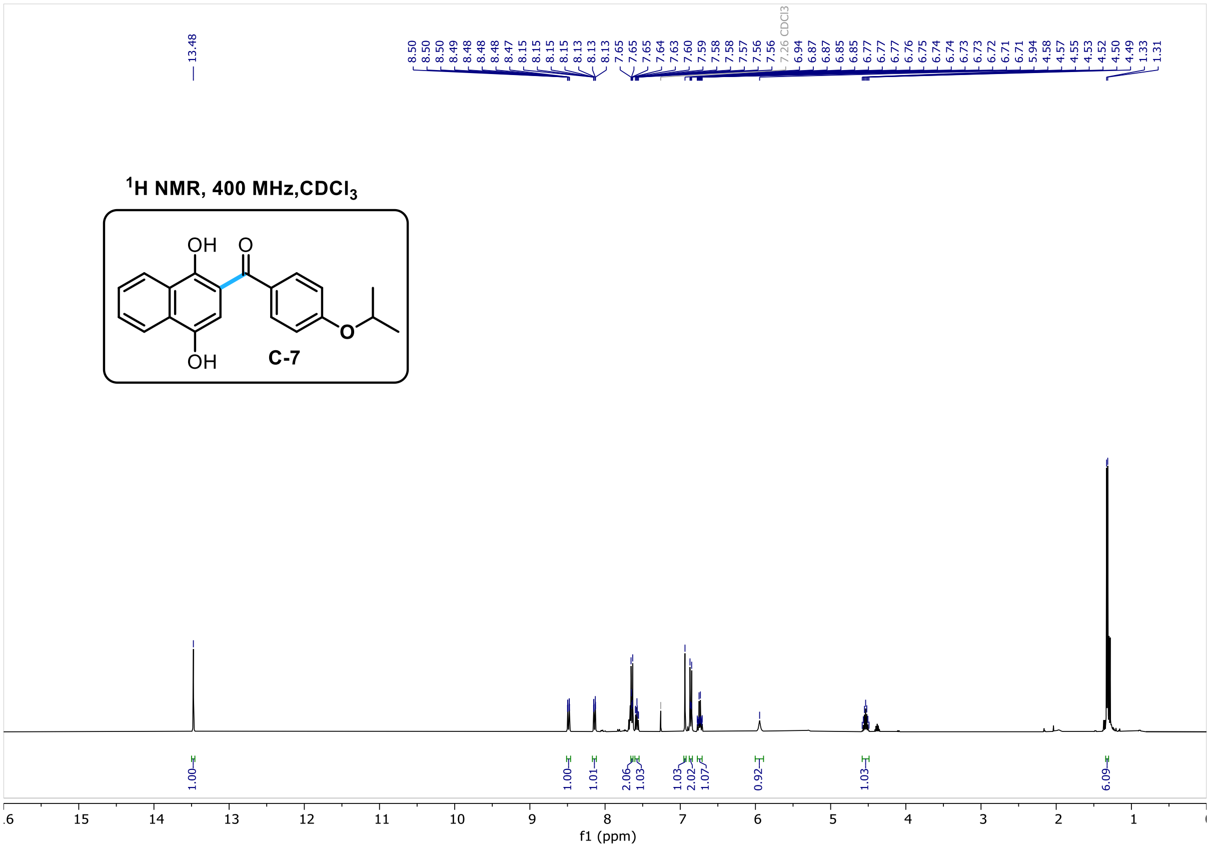
13.48
8.50 8.50 8.50 8.49 8.48 8.48 8.48 8.47 8.15 8.15 8.15 8.15 8.13 8.13 8.13 7.65 7.65 7.64 7.63 7.60 7.59 7.58 7.58 7.57 7.56 7.56
7.26 CDCl3
6.94 6.87 6.87 6.85 6.85 6.77 6.77 6.76 6.75 6.74 6.74 6.73 6.73 6.72 6.71 6.71 5.94 4.58 4.57 4.55 4.53 4.52 4.50 4.49 1.33 1.31
1H NMR, 400 MHz,CDCl3
OH
O
OH
O
C-7
1.00 1.00 1.01 2.06 1.03 1.03 2.02 1.07 0.92 1.03 6.09
f1 (ppm)

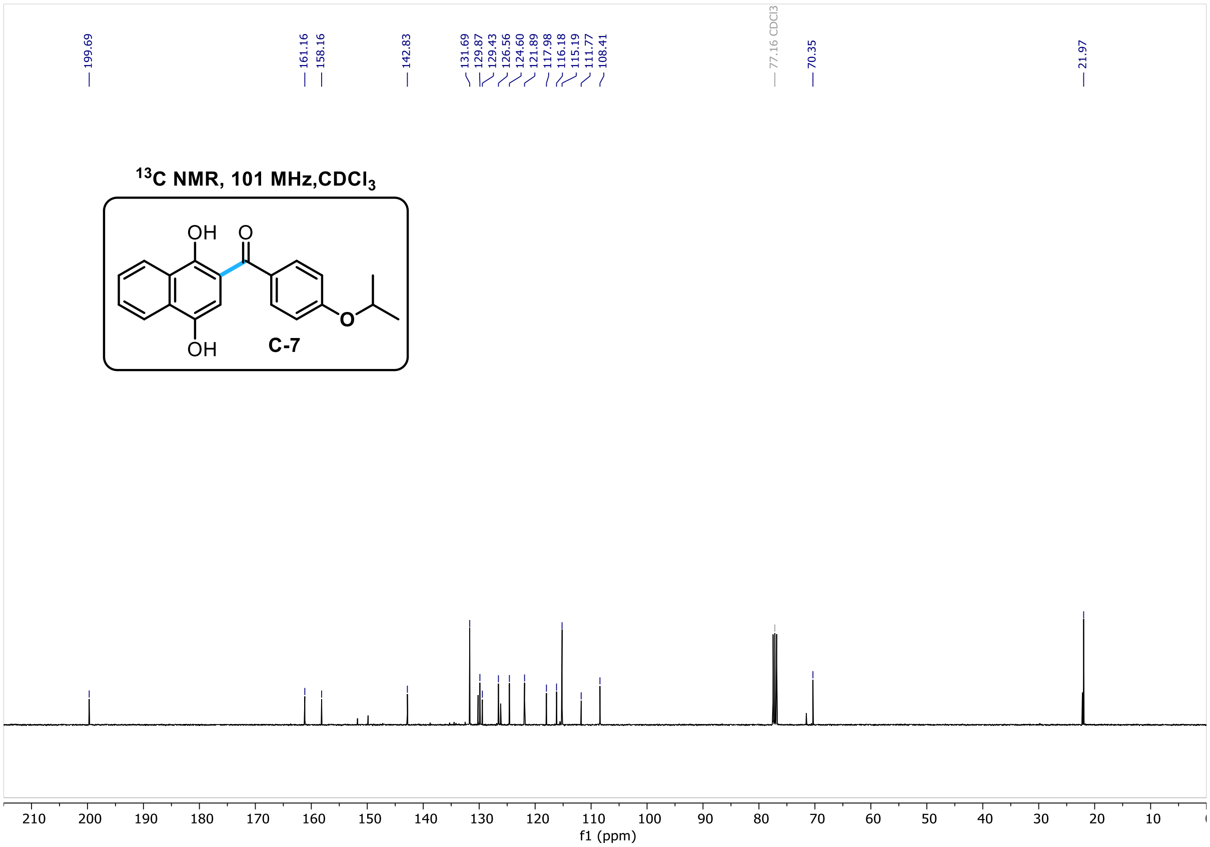
199.69
161.16 158.16
142.83
131.69 129.87 129.43 126.56 124.60 121.89 117.98 116.18 115.19 111.77 108.41
77.16 CDCl3
70.35
21.97
13C NMR, 101 MHz,CDCl3
OH
O
OH
O
C-7
f1 (ppm)

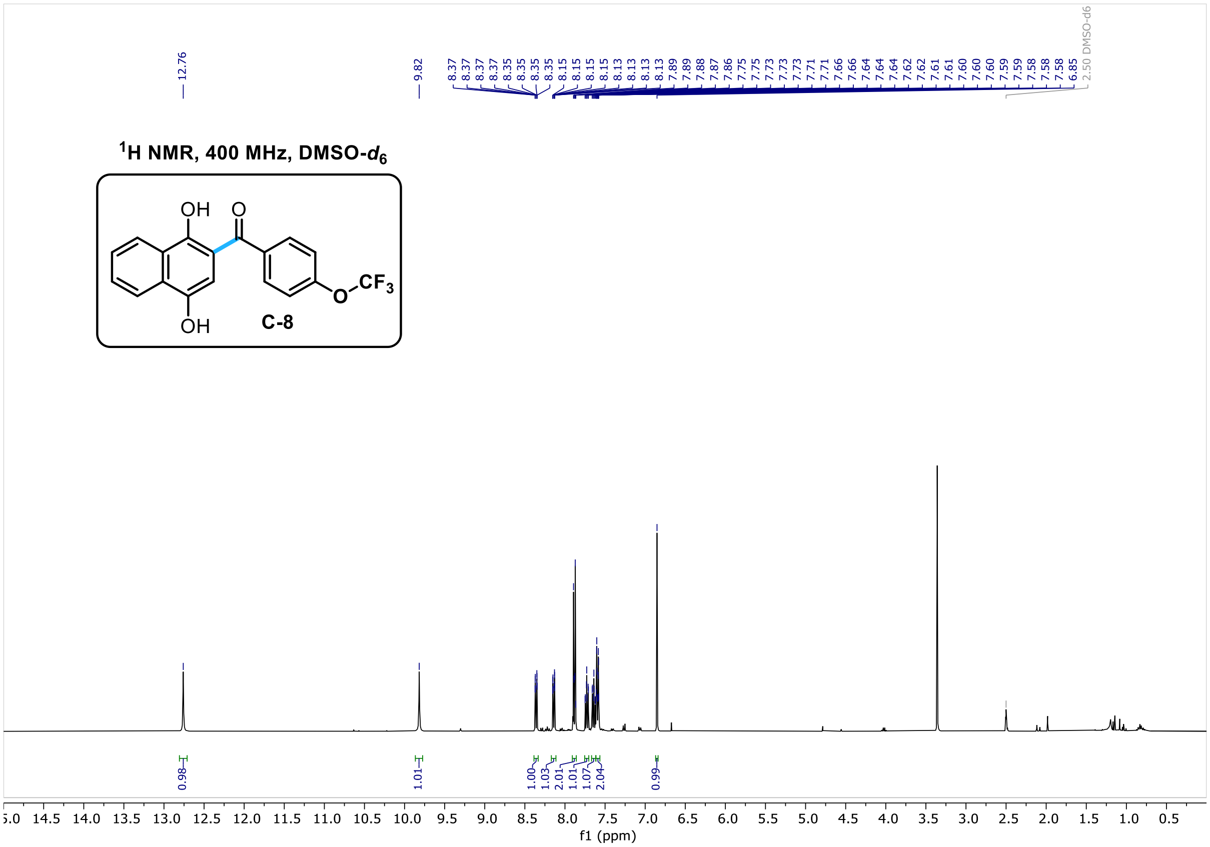
12.76
9.82
8.37 8.37 8.37 8.37 8.35 8.35 8.35 8.35 8.15 8.15 8.15 8.15 8.13 8.13 8.13 8.13 7.89 7.89 7.88 7.87 7.86 7.75 7.75 7.73 7.73 7.73 7.71 7.71 7.66 7.66 7.64 7.64 7.62 7.62 7.61 7.60 7.60 7.59 7.58 7.58 6.85
2.50 DMSO-d6
1H NMR, 400 MHz, DMSO-d6
OH
O
OH
O
CF3
C-8
0.98
1.01
1.00
1.03
2.01
1.01
1.07
2.04
0.99
f1 (ppm)

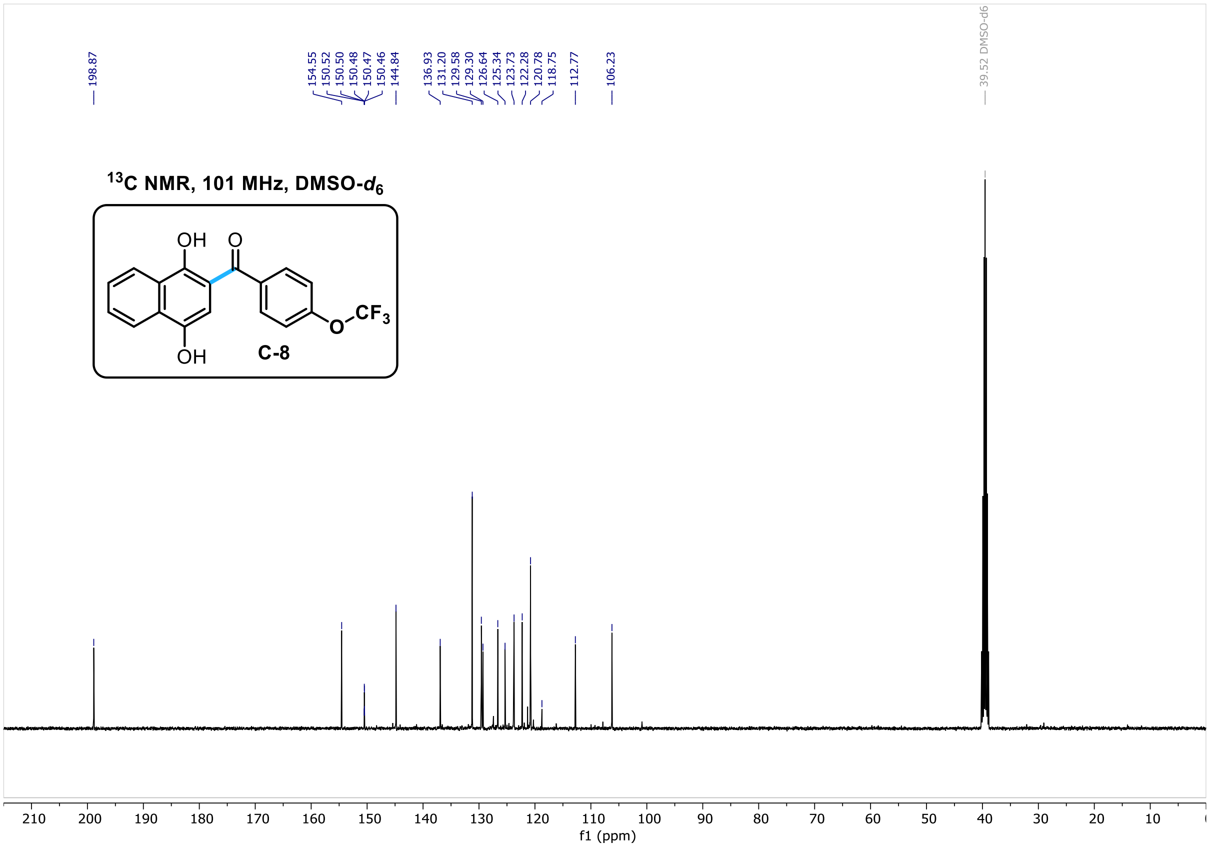
198.87
154.55 150.52 150.50 150.48 150.47 150.46 144.84
136.93 131.20 129.58 129.30 126.64 125.34 123.73 122.28 120.78 118.75
112.77
106.23
39.52 DMSO-d6
13C NMR, 101 MHz, DMSO-d6
OH
O
OH
O
CF3
C-8
f1 (ppm)

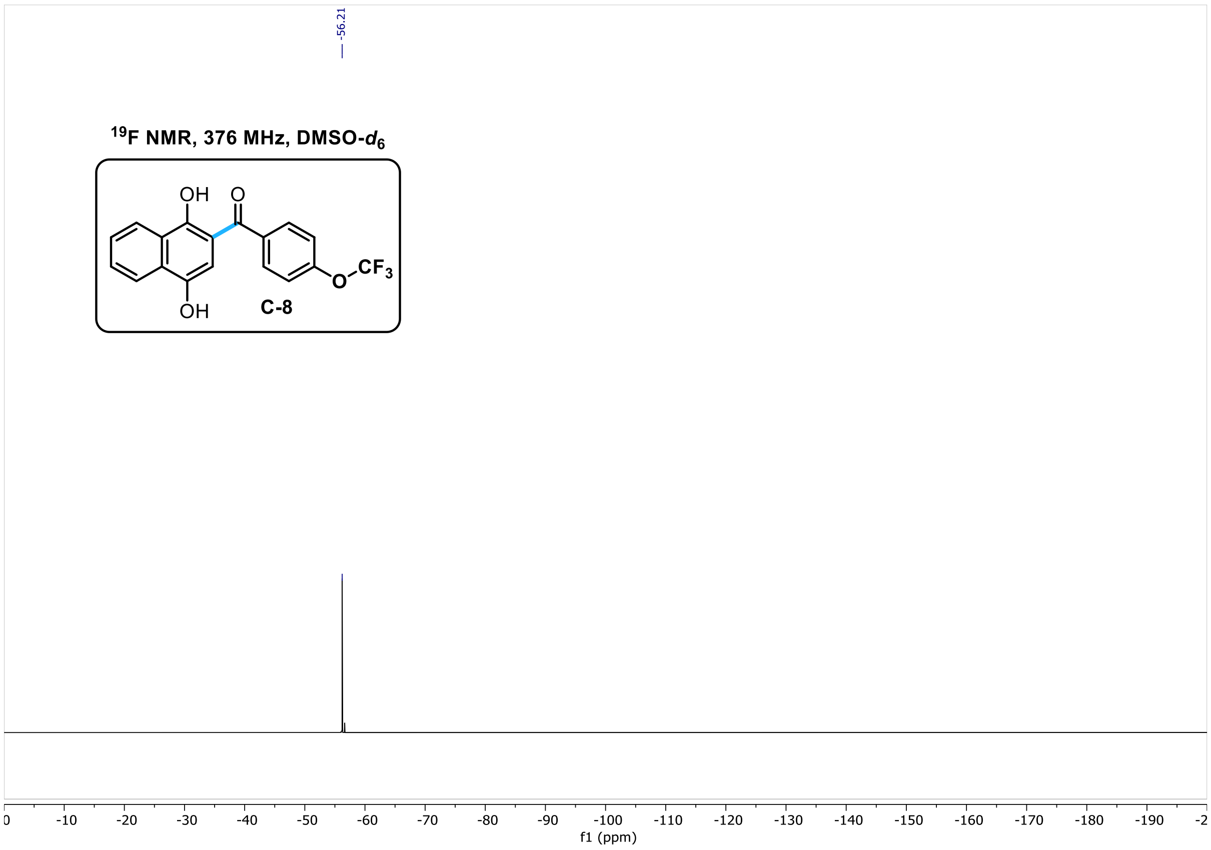
-56.21
^{19}F NMR, 376 MHz, DMSO-d_6
OH
O
OH
O
CF$_3$
C-8
0 -10 -20 -30 -40 -50 -60 -70 -80 -90 -100 -110 -120 -130 -140 -150 -160 -170 -180 -190 -2
f1 (ppm)

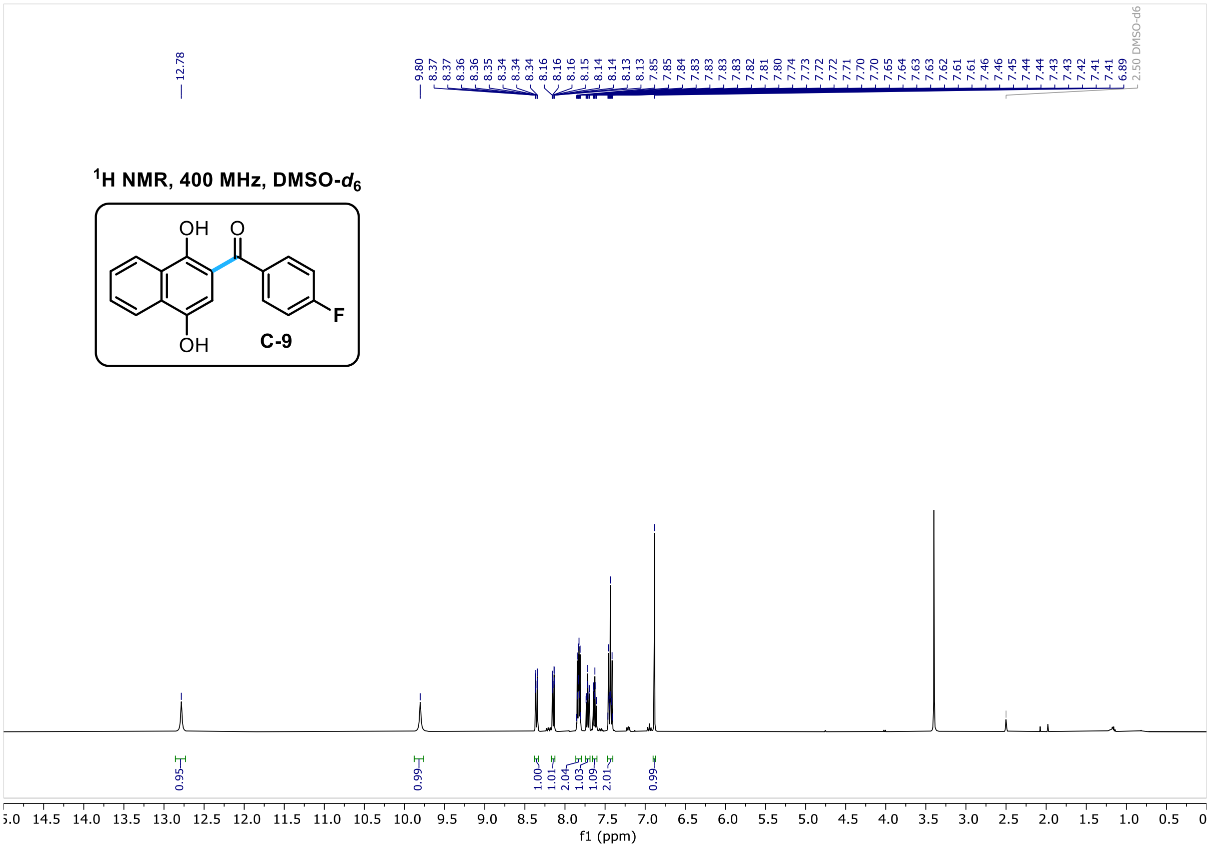

12.78
9.80 8.37 8.37 8.36 8.36 8.35 8.34 8.34 8.34 8.16 8.16 8.16 8.15 8.14 8.14 8.13 8.13 7.85 7.85 7.84 7.83 7.83 7.83 7.83 7.82 7.81 7.80 7.74 7.73 7.72 7.72 7.71 7.70 7.70 7.65 7.64 7.63 7.63 7.62 7.61 7.61 7.46 7.45 7.44 7.44 7.43 7.42 7.41 7.41 6.89
2.50 DMSO-d6
¹H NMR, 400 MHz, DMSO-d6
OH
O
F
OH
C-9
0.95 0.99 1.00 1.01 2.04 1.03 1.09 2.01 0.99
f1 (ppm)

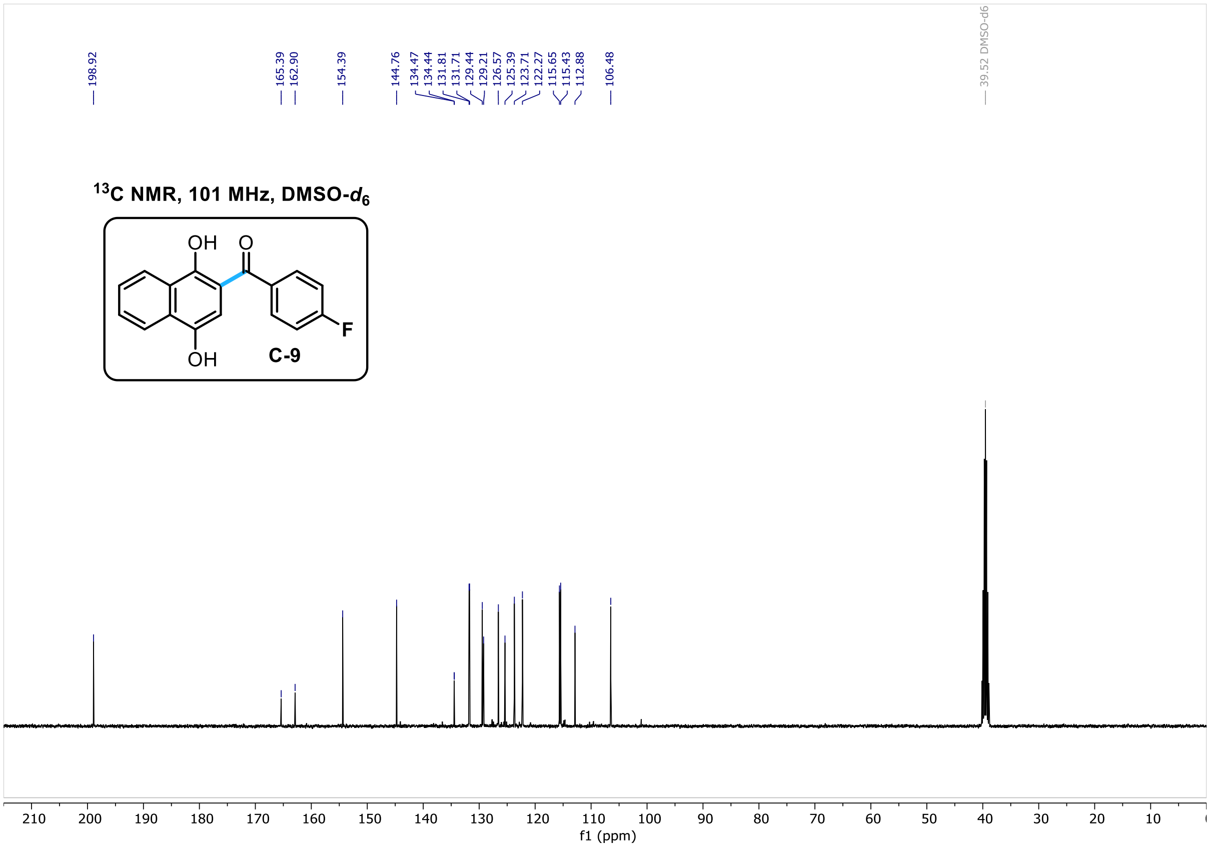

198.92
165.39 162.90
154.39
144.76 134.47 134.44 131.81 131.71 129.44 129.21 126.57 125.39 123.71 122.27 115.65 115.43 112.88 106.48
39.52 DMSO-d6
¹³C NMR, 101 MHz, DMSO-d6
OH
O
F
OH
C-9
f1 (ppm)

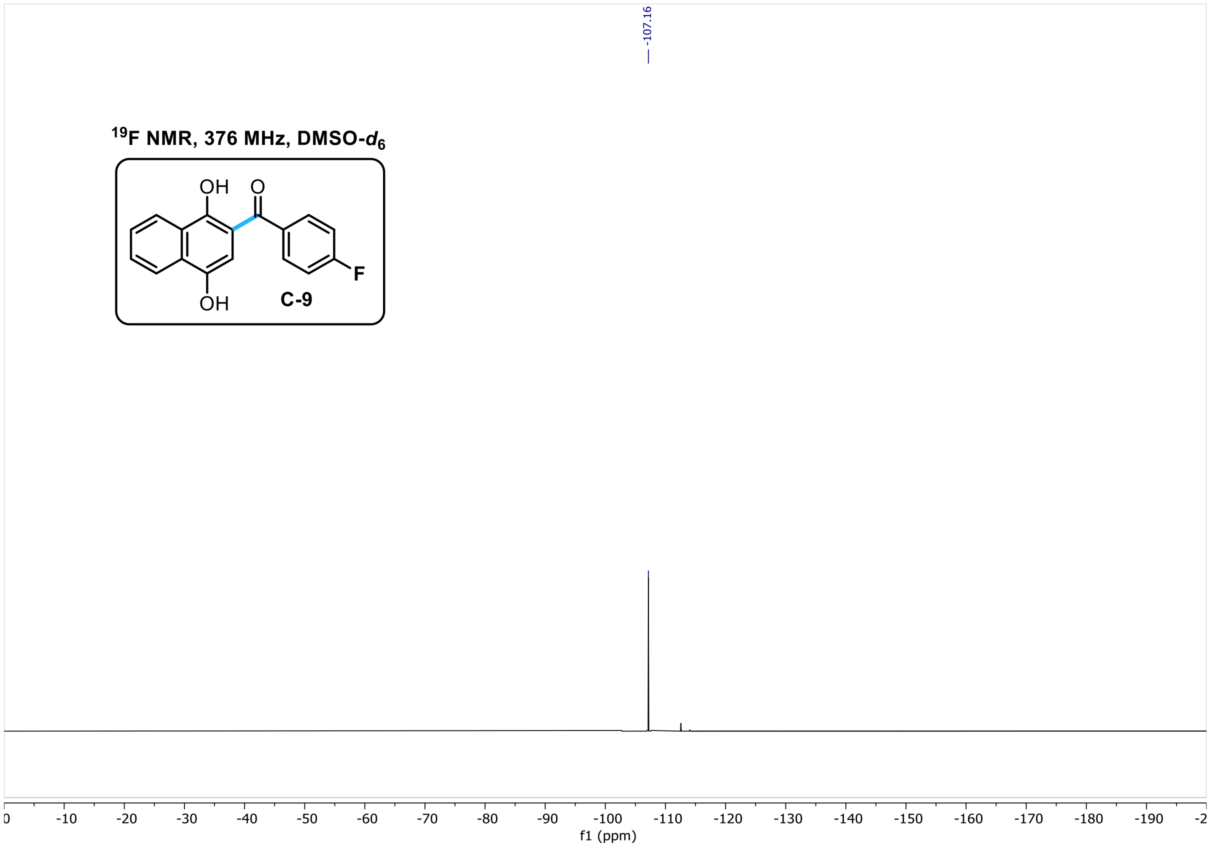
-107.16
19F NMR, 376 MHz, DMSO-d6
OH
O
F
OH
C-9
0
-10
-20
-30
-40
-50
-60
-70
-80
-90
-100
-110
-120
-130
-140
-150
-160
-170
-180
-190
-2
f1 (ppm)

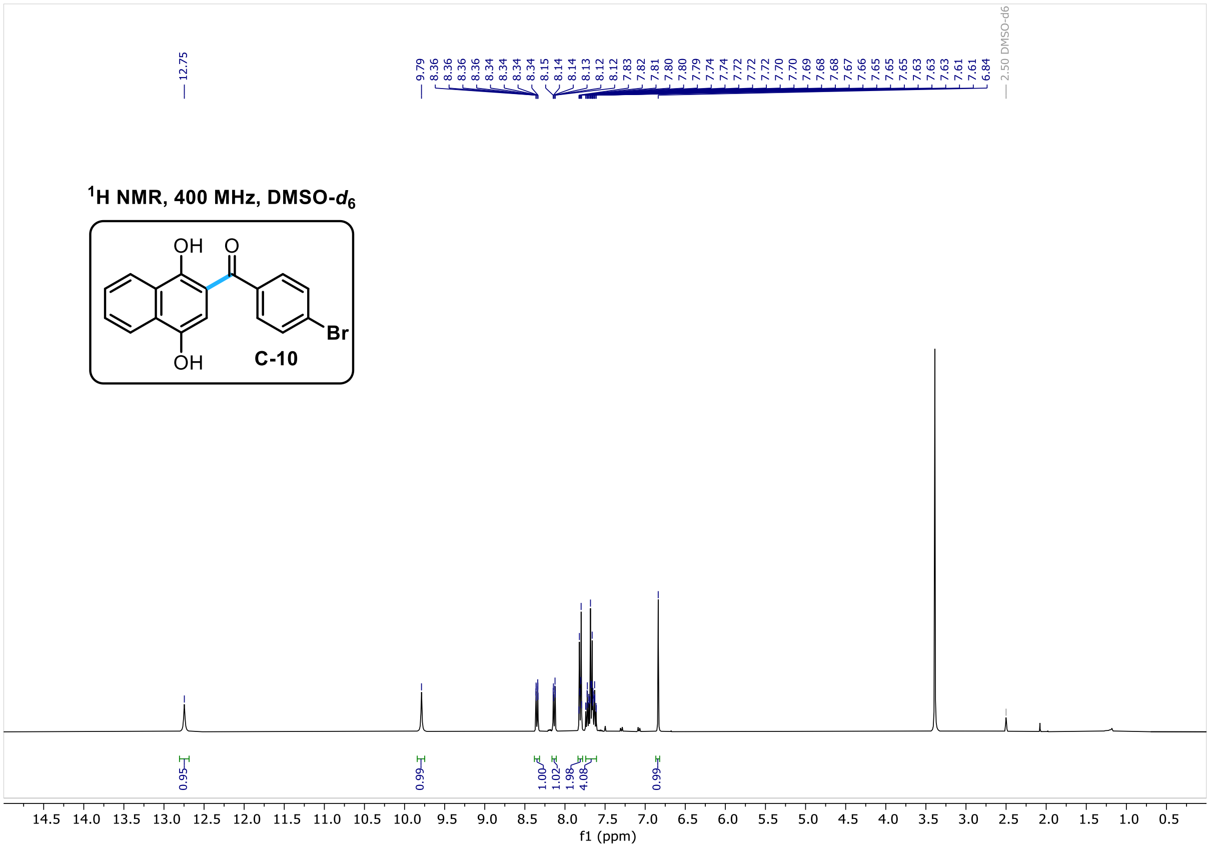
1H NMR, 400 MHz, DMSO-d6
OH
O
Br
OH
C-10
12.75
9.79
8.36 8.36 8.36 8.36 8.34 8.34 8.34 8.34 8.15 8.14 8.14 8.13 8.12 8.12 7.83 7.82 7.81 7.80 7.80 7.79 7.74 7.74 7.72 7.72 7.72 7.70 7.70 7.69 7.68 7.68 7.67 7.66 7.65 7.65 7.65 7.63 7.63 7.63 7.61 7.61 6.84
2.50 DMSO-d6
0.95
0.99
1.00
1.02
1.98
4.08
0.99
f1 (ppm)

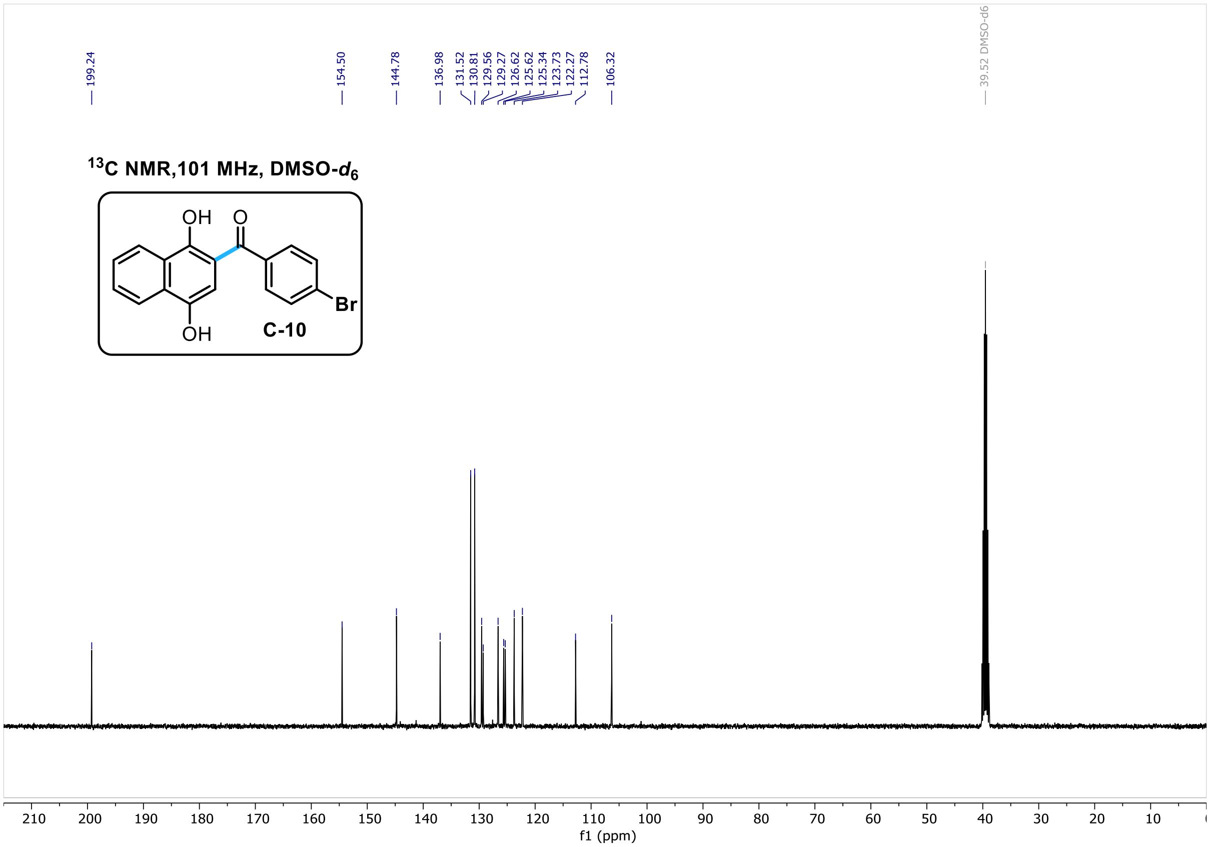
13C NMR,101 MHz, DMSO-d6
OH
O
Br
OH
C-10
199.24
154.50
144.78
136.98
131.52
130.81
129.56
129.27
126.62
125.62
125.34
123.73
122.27
112.78
106.32
39.52 DMSO-d6
f1 (ppm)

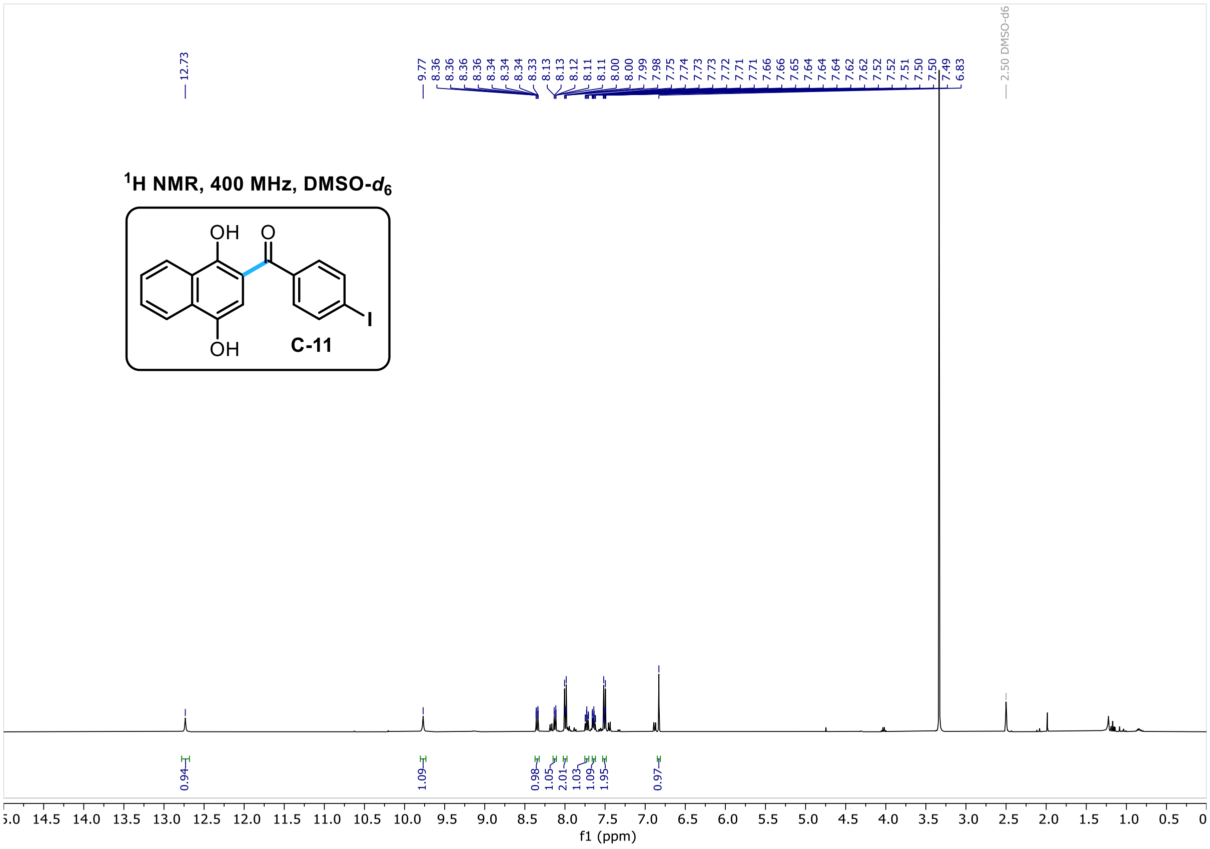
12.73
9.77
8.36
8.36
8.36
8.36
8.34
8.34
8.34
8.33
8.13
8.13
8.12
8.11
8.11
8.11
8.00
8.00
7.99
7.98
7.75
7.74
7.73
7.73
7.72
7.71
7.71
7.66
7.66
7.65
7.64
7.64
7.62
7.62
7.52
7.52
7.51
7.50
7.50
7.49
6.83
2.50 DMSO-d6
^{1}H NMR, 400 MHz, DMSO-d_6
OH
O
OH
I
C-11
0.94
1.09
0.98
1.05
2.01
1.03
1.09
1.95
0.97
f1 (ppm)

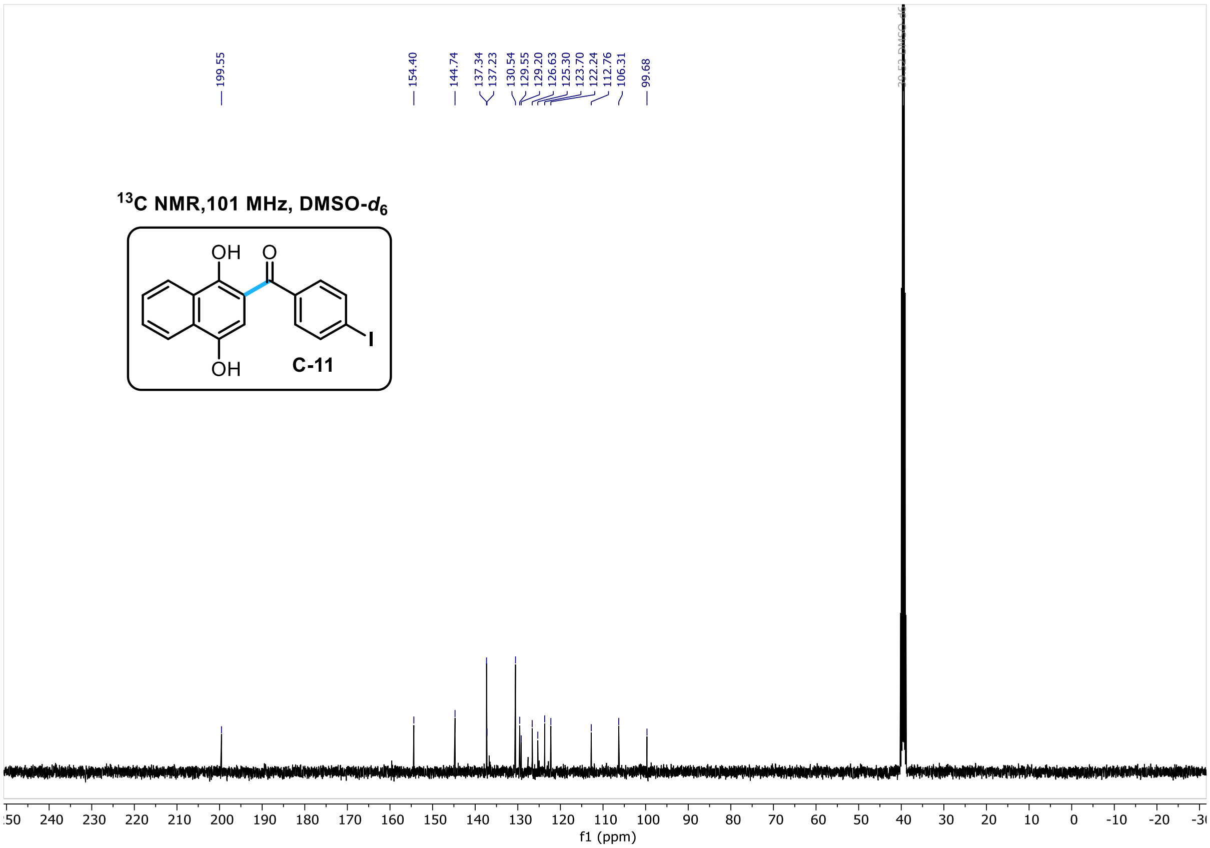
199.55
154.40
144.74
137.34
137.23
130.54
129.55
129.20
126.63
125.30
123.70
122.24
112.76
106.31
99.68
^{13}C NMR,101 MHz, DMSO-d_6
OH
O
OH
I
C-11
f1 (ppm)

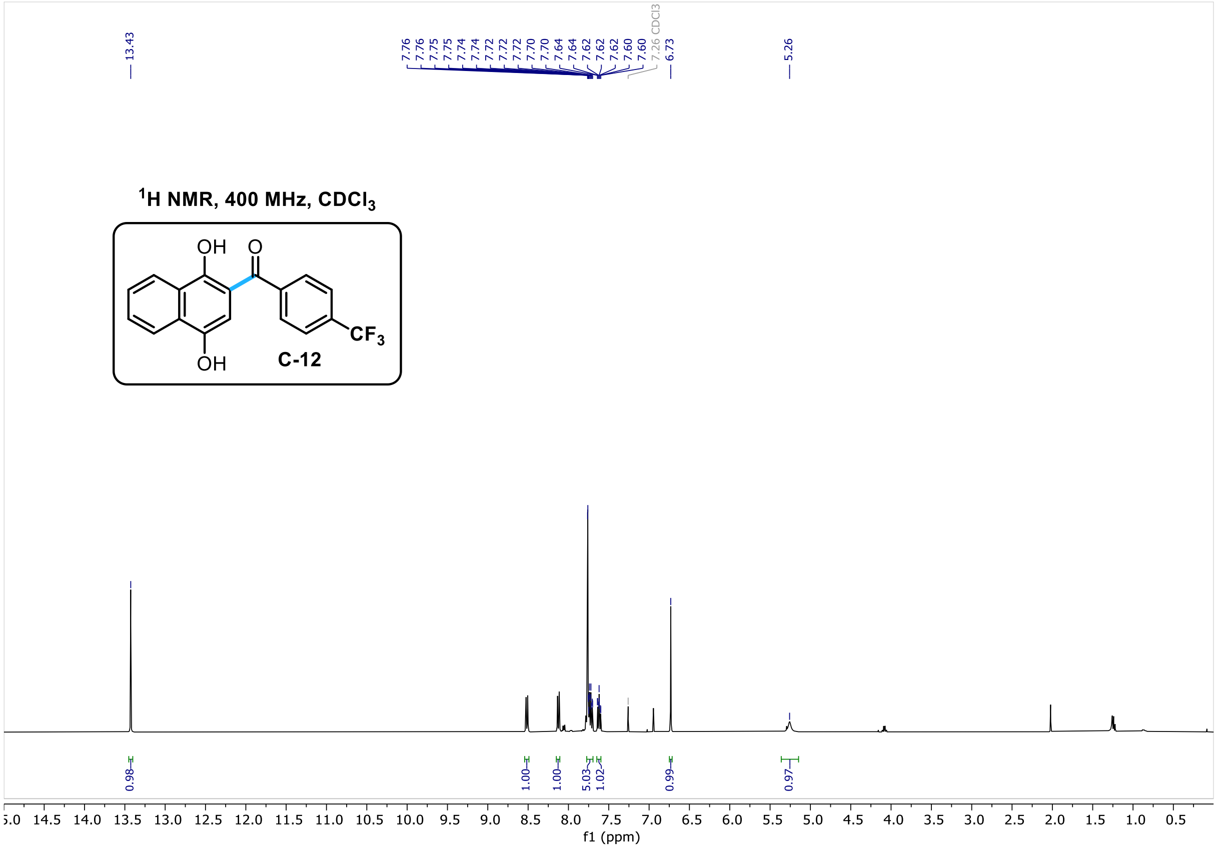
13.43
7.76 7.76 7.75 7.75 7.74 7.74 7.72 7.72 7.72 7.72 7.70 7.70 7.64 7.64 7.62 7.62 7.62 7.62 7.60 7.60
7.26 CDCl3
6.73
5.26
^{1}H NMR, 400 MHz, CDCl$_3$
OH
O
CF$_3$
OH
C-12
0.98
1.00
1.00
5.03
1.02
0.99
0.97
f1 (ppm)

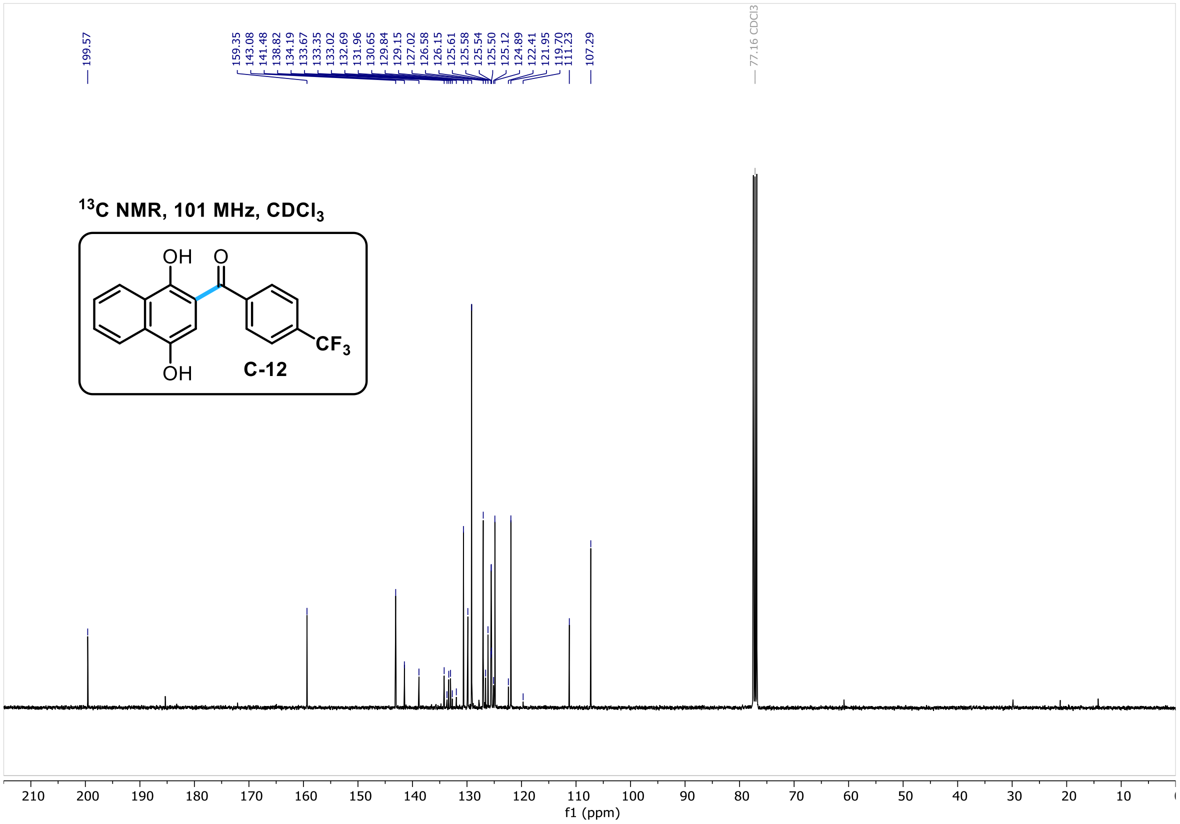
199.57
159.35 143.08 141.48 138.82 134.19 133.67 133.35 133.02 132.69 131.96 130.65 129.84 129.15 127.02 126.58 126.15 125.61 125.58 125.54 125.50 125.12 124.89 122.41 121.95 119.70 111.23 107.29
77.16 CDCl3
^{13}C NMR, 101 MHz, CDCl$_3$
OH
O
CF$_3$
OH
C-12
f1 (ppm)

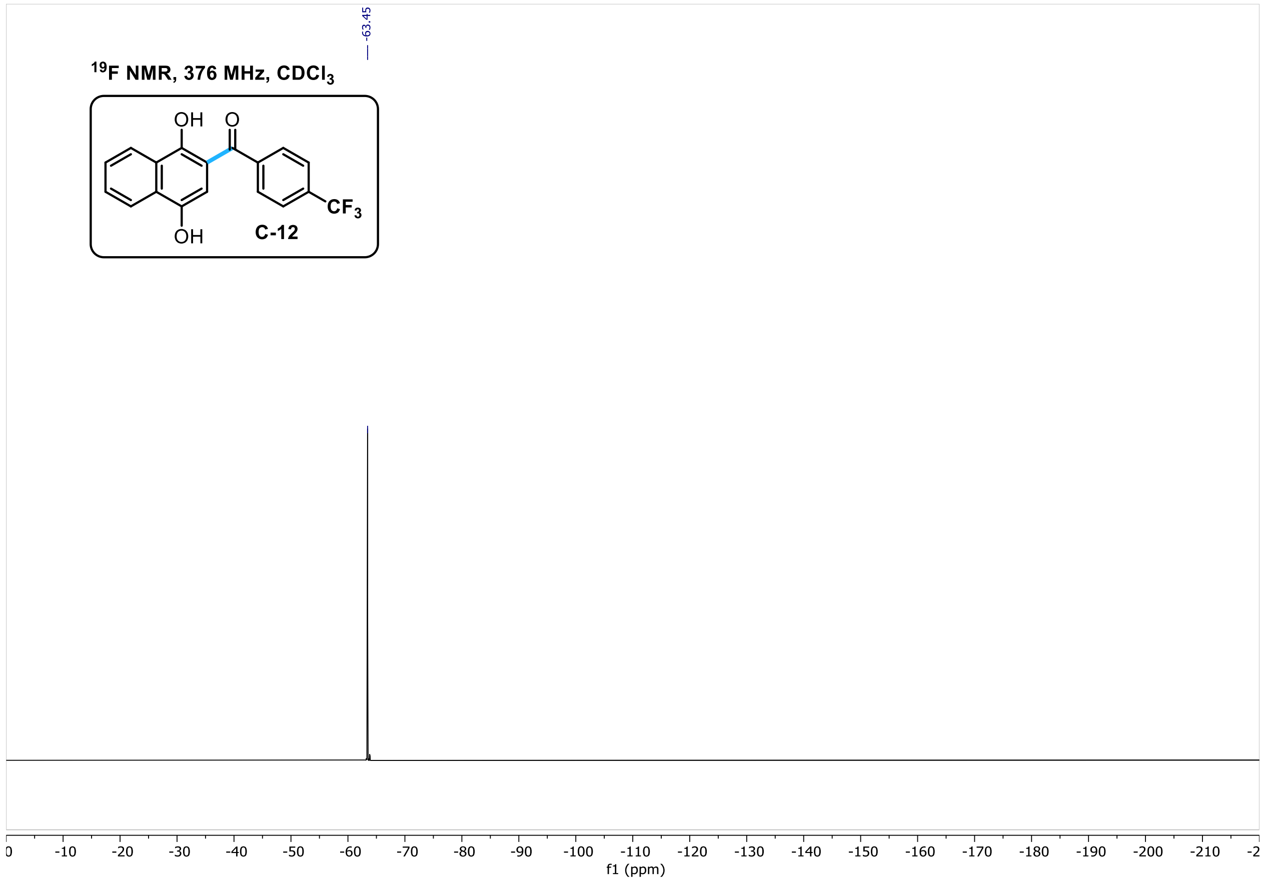
-63.45
19F NMR, 376 MHz, CDCl3
OH
O
CF3
OH
C-12
0
-10
-20
-30
-40
-50
-60
-70
-80
-90
-100
-110
-120
-130
-140
-150
-160
-170
-180
-190
-200
-210
f1 (ppm)

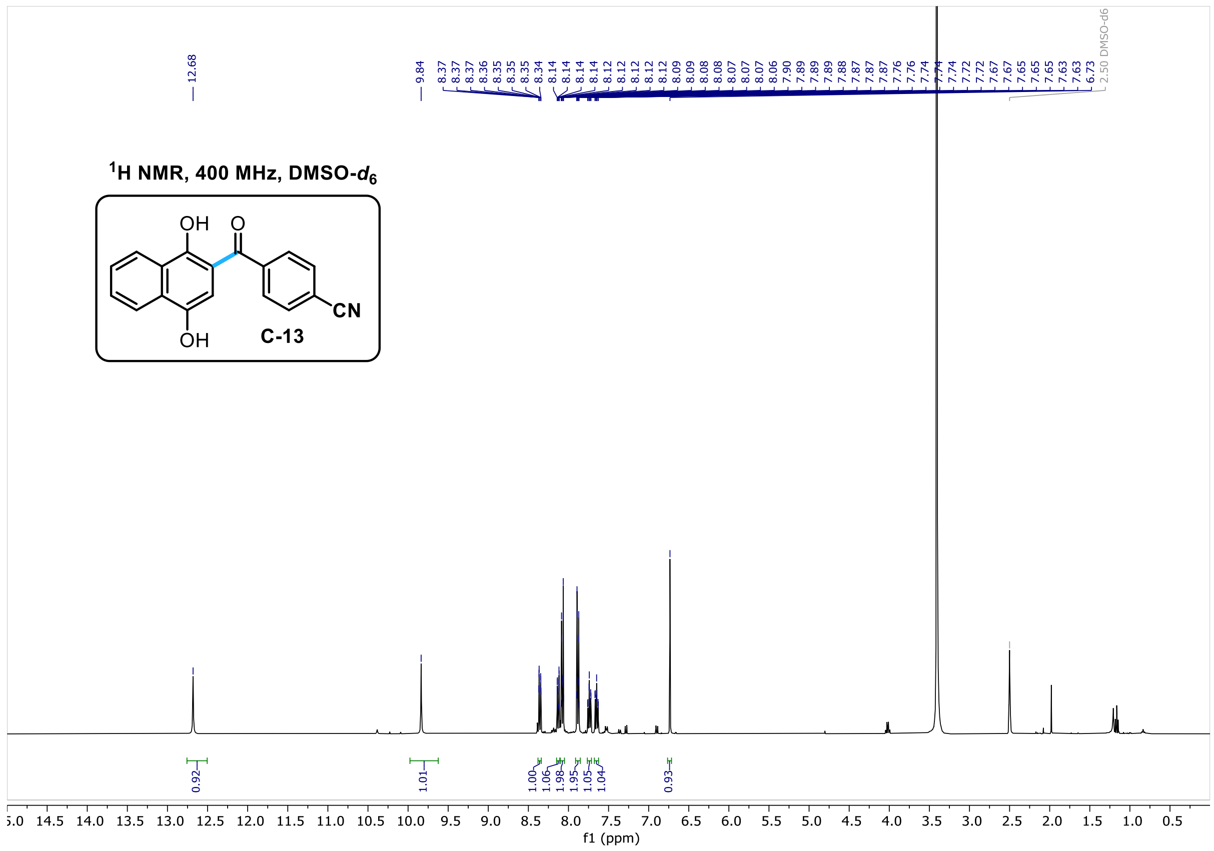
1H NMR, 400 MHz, DMSO-d6
OH
O
OH
CN
C-13
12.68
9.84
2.50 DMSO-d6
0.92
1.01
1.00
1.06
1.98
1.95
1.05
1.04
0.93
f1 (ppm)

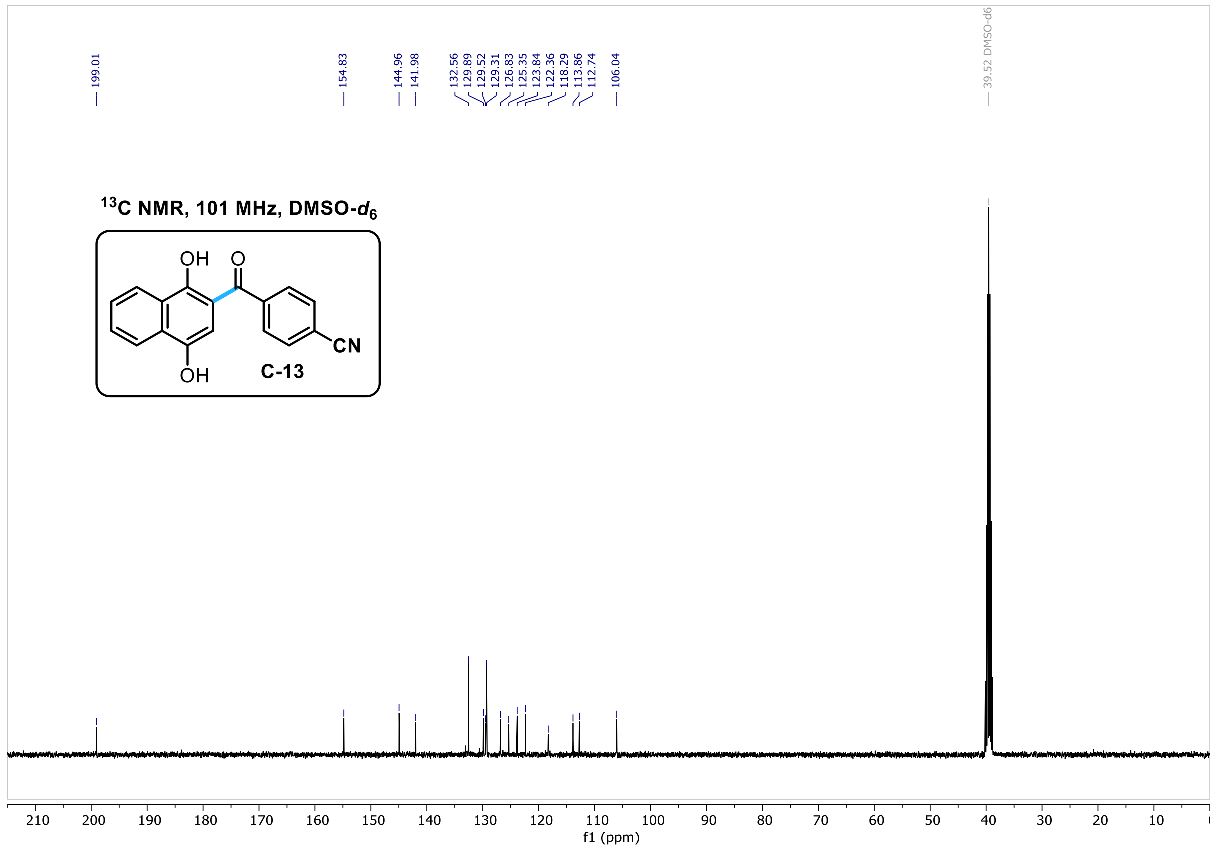
13C NMR, 101 MHz, DMSO-d6
OH
O
OH
CN
C-13
199.01
154.83
144.96
141.98
132.56
129.89
129.52
129.31
126.83
125.35
123.84
122.36
118.29
113.86
112.74
106.04
39.52 DMSO-d6
f1 (ppm)

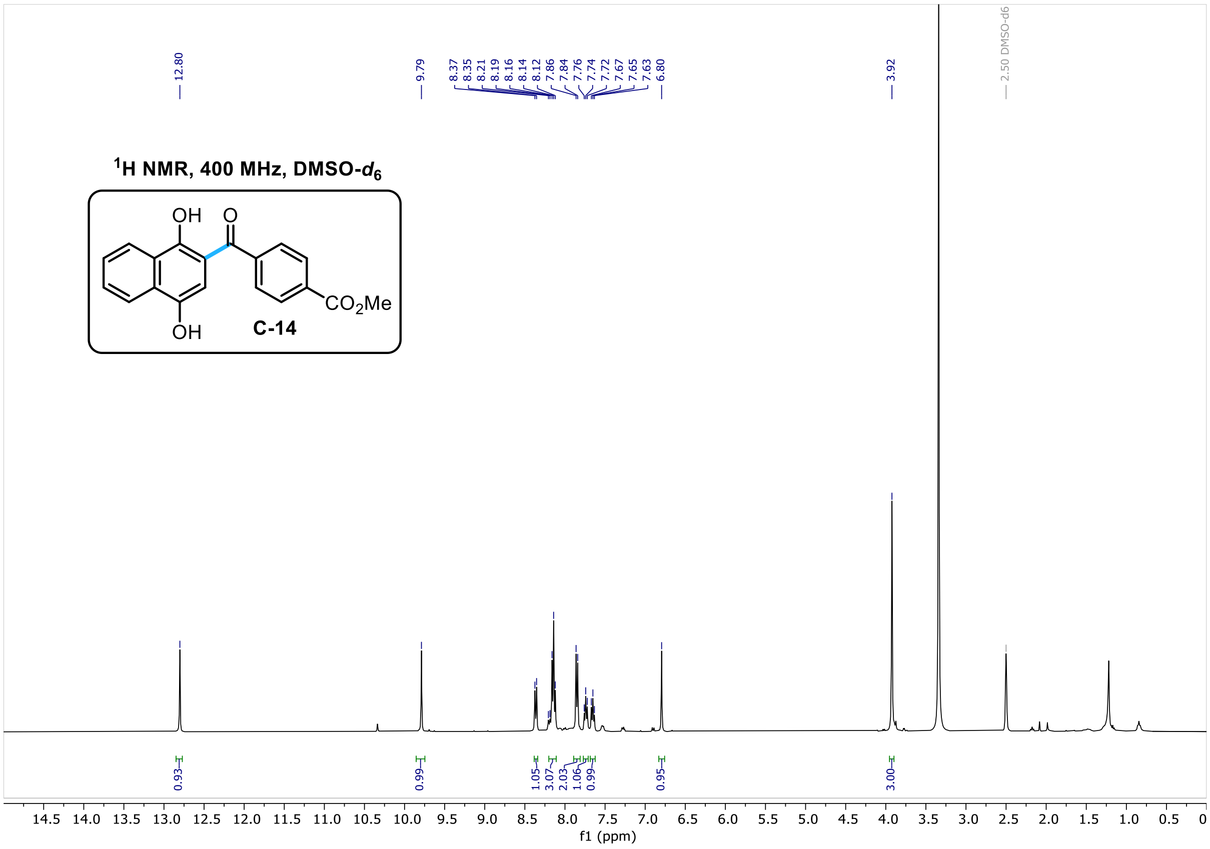

12.80
9.79
8.37
8.35
8.21
8.19
8.16
8.14
8.12
7.86
7.84
7.76
7.74
7.72
7.67
7.65
7.63
6.80
3.92
2.50 DMSO-d6
^{1}H NMR, 400 MHz, DMSO-d_6
OH
O
CO$_2$Me
OH
C-14
0.93
0.99
1.05
3.07
2.03
1.06
0.99
0.95
3.00
f1 (ppm)

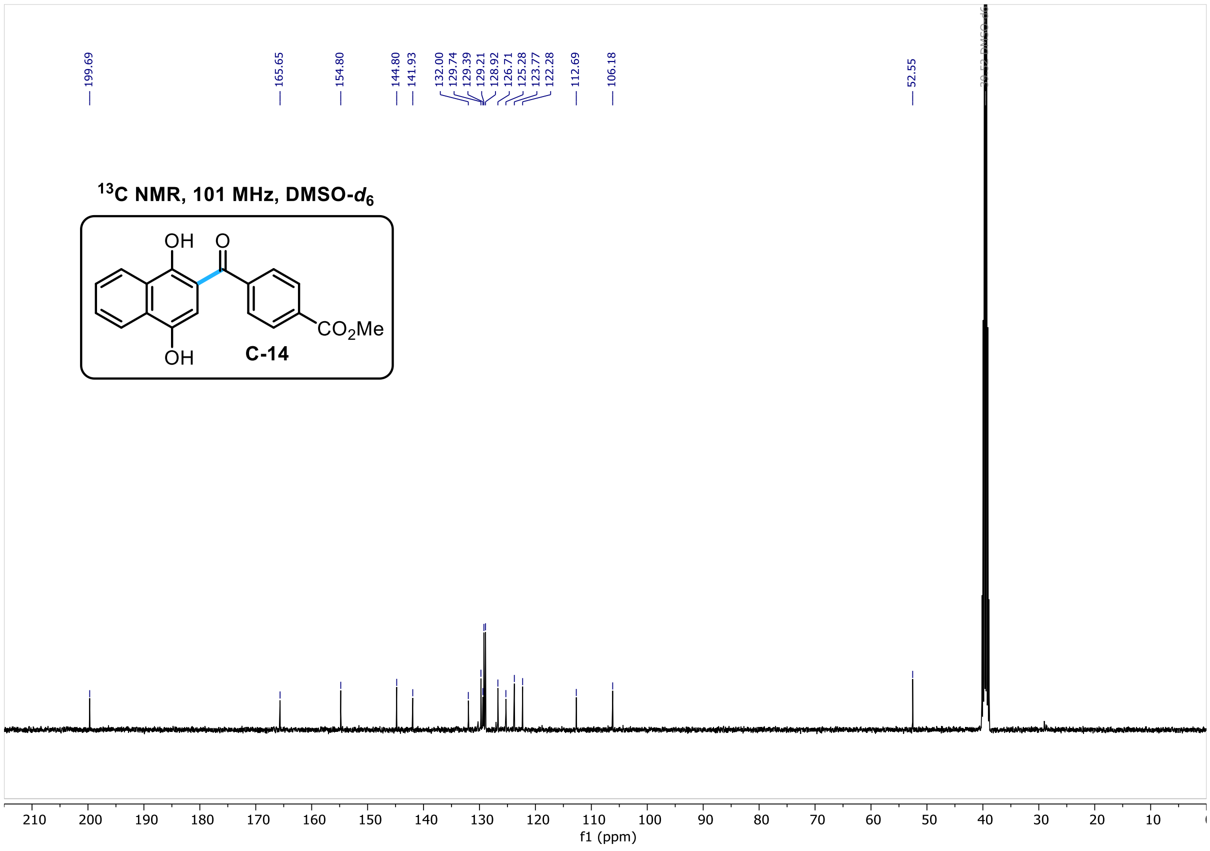

199.69
165.65
154.80
144.80
141.93
132.00
129.74
129.39
129.21
128.92
126.71
125.28
123.77
122.28
112.69
106.18
52.55
^{13}C NMR, 101 MHz, DMSO-d_6
OH
O
CO$_2$Me
OH
C-14
f1 (ppm)

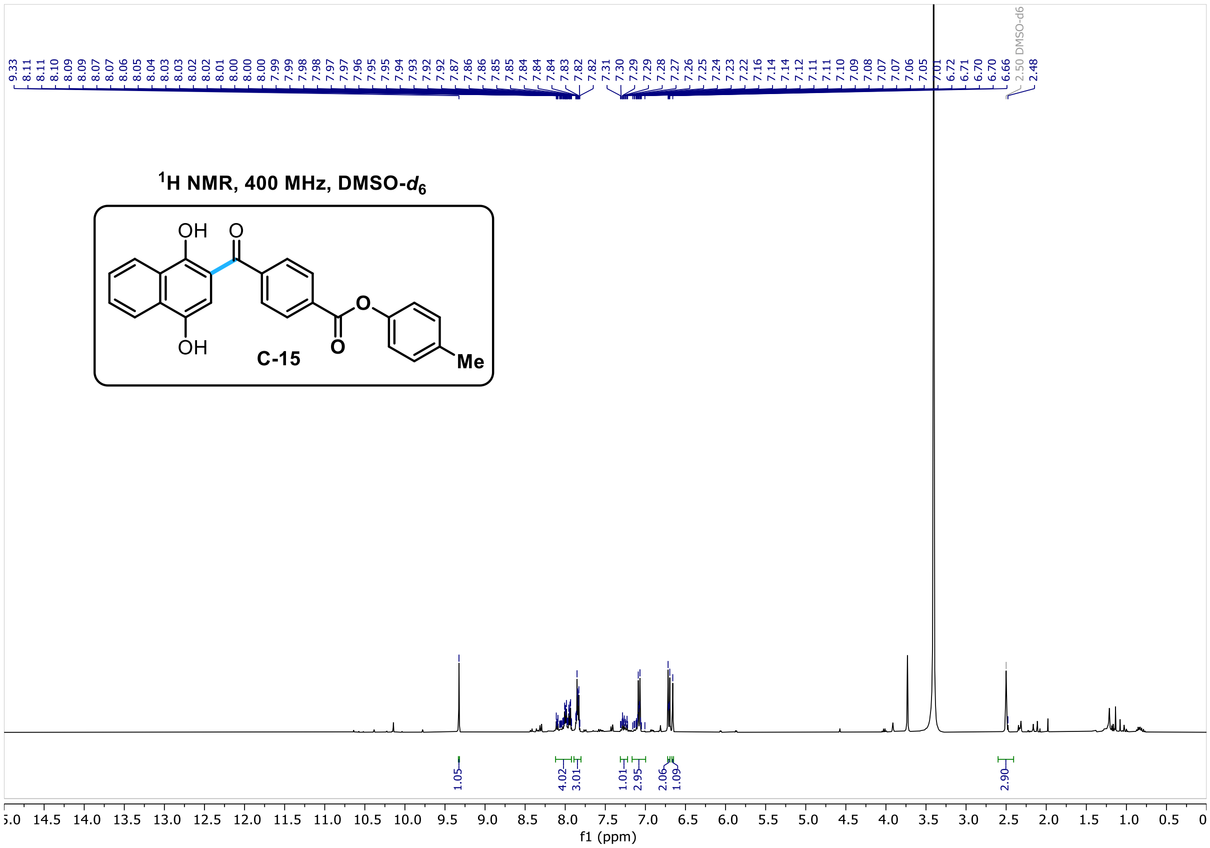

1H NMR, 400 MHz, DMSO-d6
C-15
f1 (ppm)

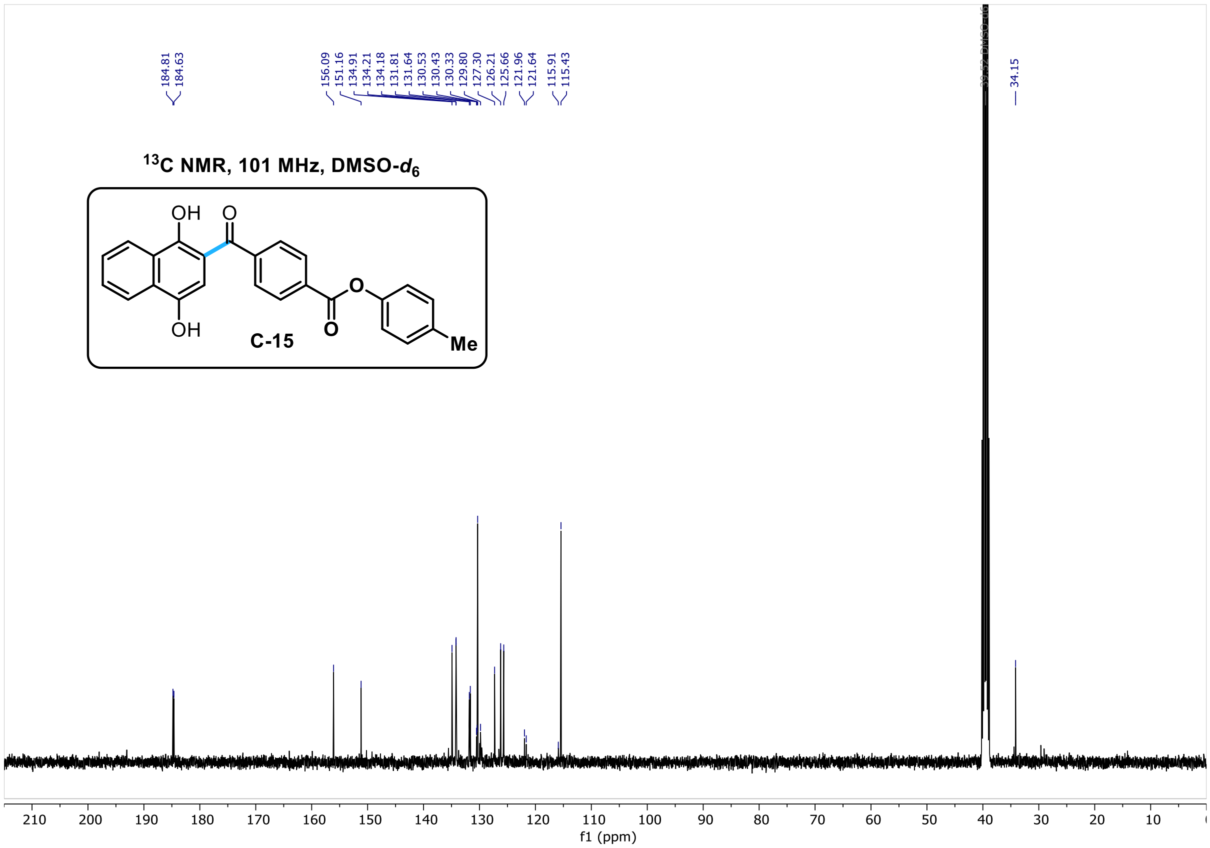

13C NMR, 101 MHz, DMSO-d6
C-15
f1 (ppm)

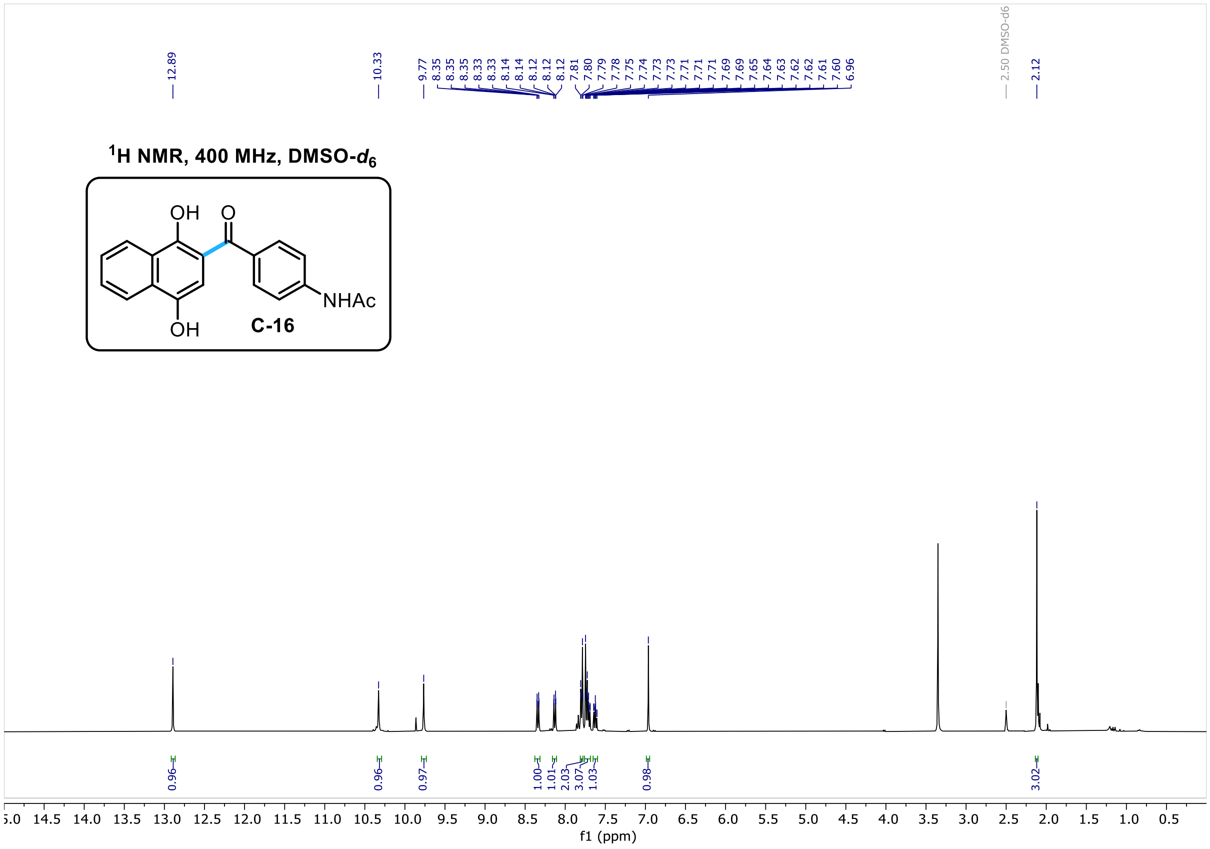

^{1}H NMR, 400 MHz, DMSO-d6
C-16

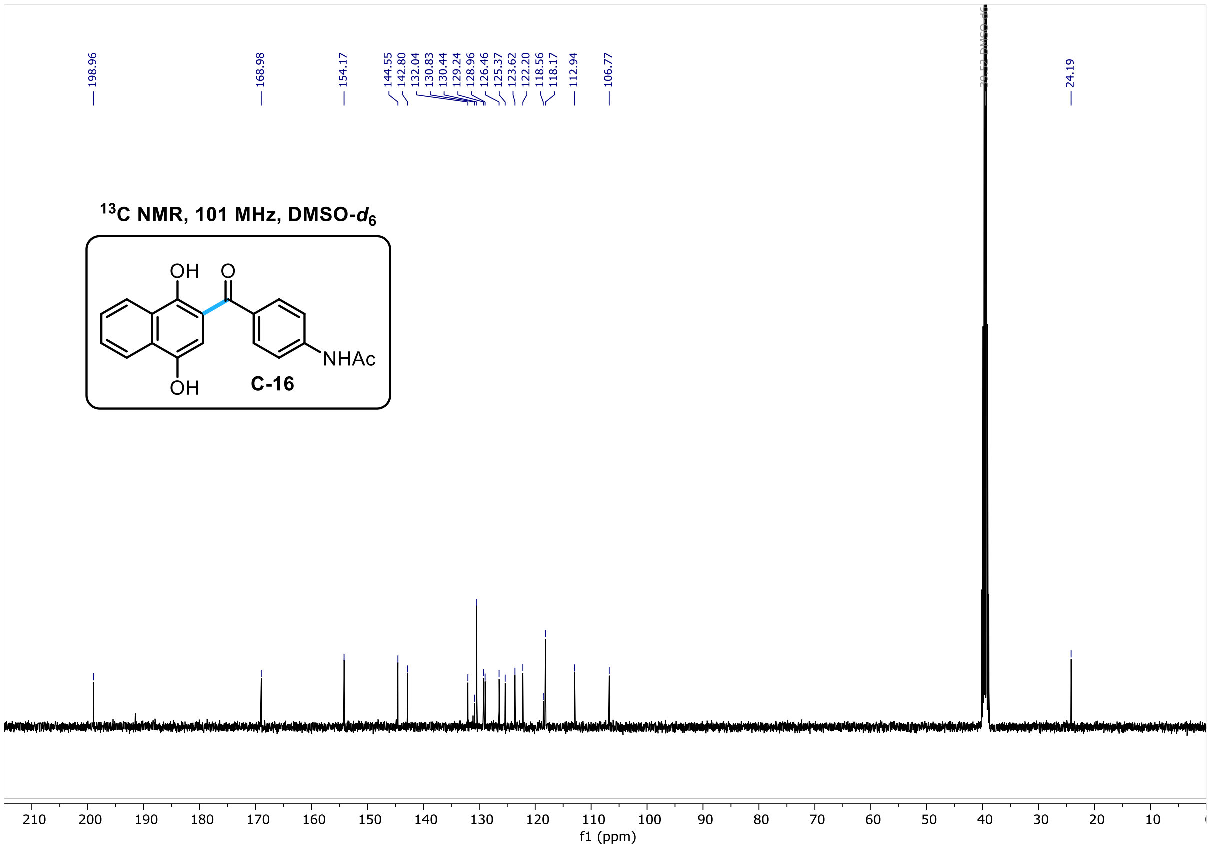

^{13}C NMR, 101 MHz, DMSO-d6
C-16

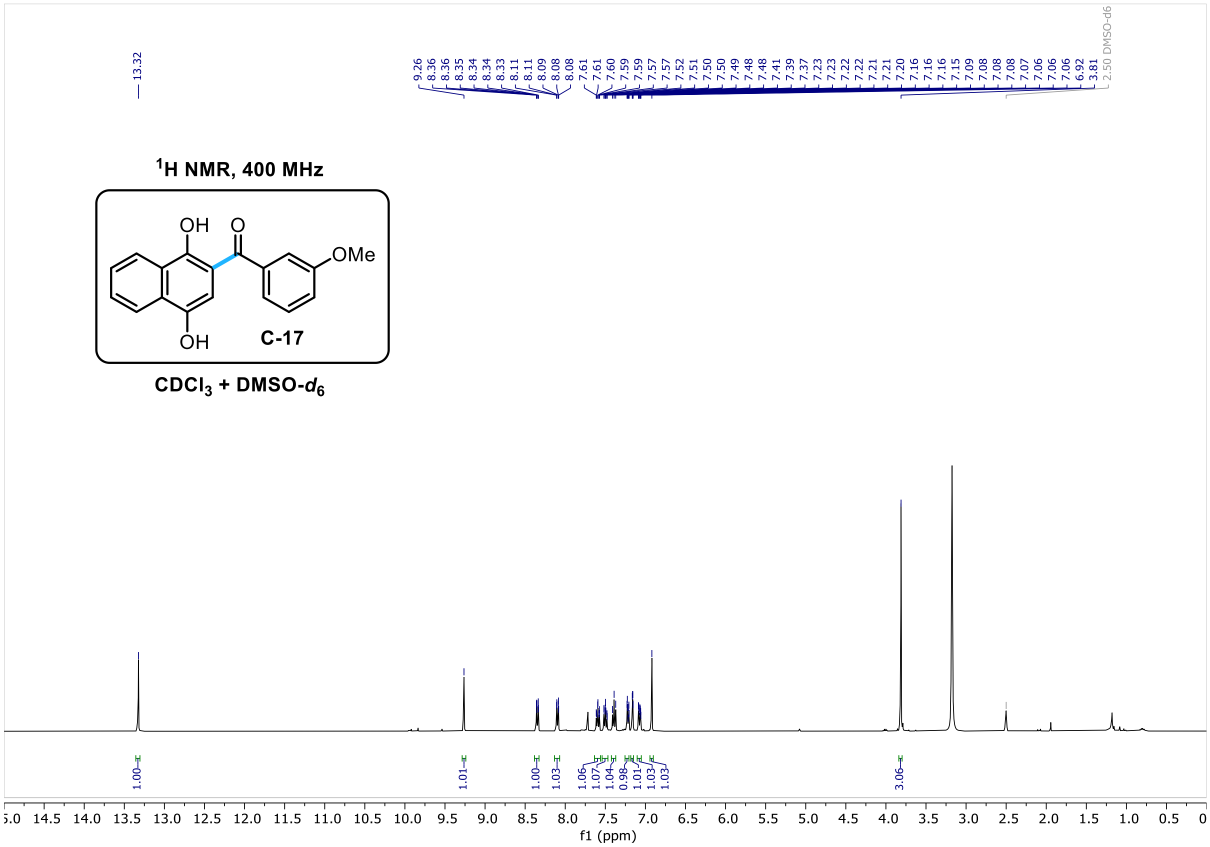
13.32
9.26 8.36 8.36 8.35 8.34 8.34 8.33 8.11 8.11 8.09 8.08 8.08 7.61 7.61 7.60 7.59 7.59 7.57 7.57 7.52 7.51 7.50 7.50 7.49 7.48 7.48 7.41 7.39 7.37 7.23 7.23 7.22 7.22 7.21 7.21 7.20 7.16 7.16 7.16 7.15 7.09 7.08 7.08 7.08 7.07 7.06 7.06 7.06 6.92 3.81
2.50 DMSO-d6
^{1}H NMR, 400 MHz
OH
O
OMe
OH
C-17
$CDCl_3$ + DMSO-d_6
1.00
1.01
1.00
1.03
1.06
1.07
1.04
0.98
1.01
1.03
1.03
3.06
f1 (ppm)

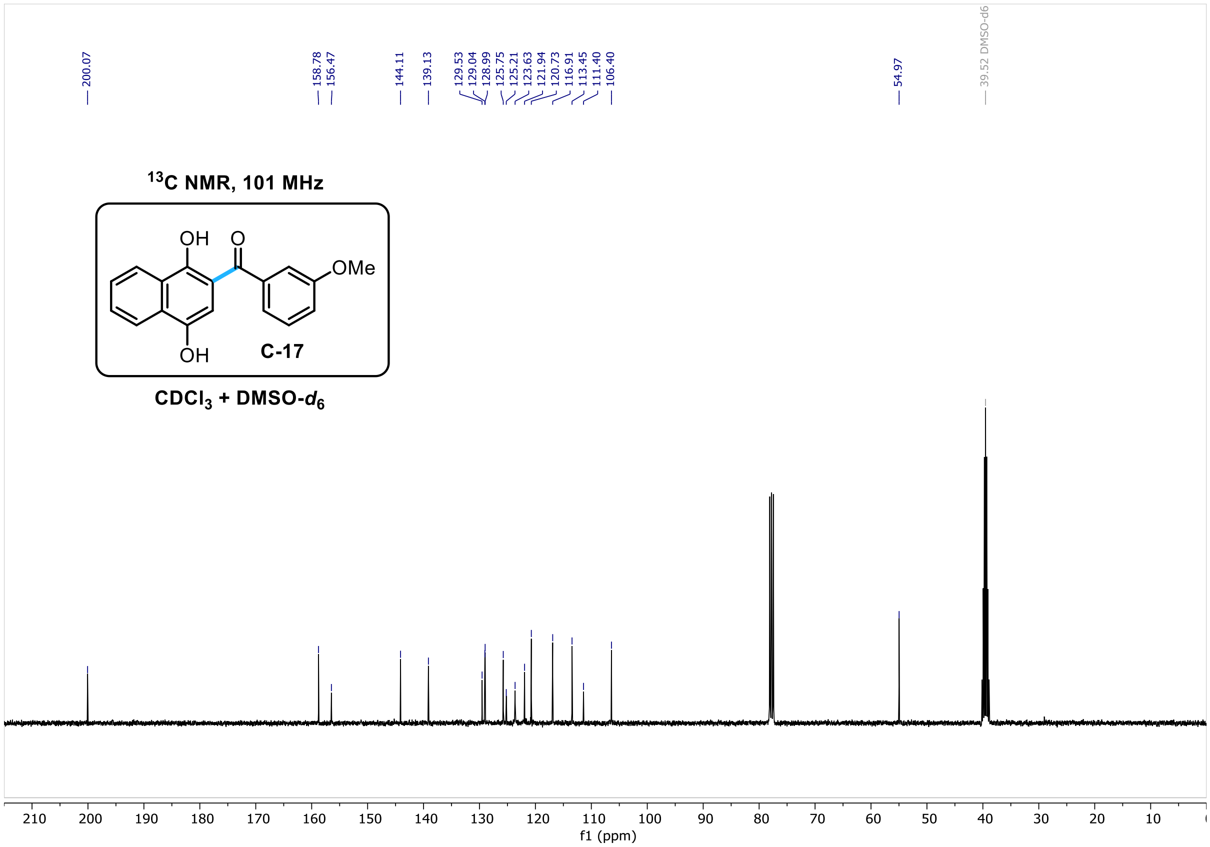
200.07
158.78 156.47
144.11
139.13
129.53 129.04 128.99 125.75 125.21 123.63 121.94 120.73 116.91 113.45 111.40 106.40
54.97
39.52 DMSO-d6
^{13}C NMR, 101 MHz
OH
O
OMe
OH
C-17
$CDCl_3$ + DMSO-d_6
f1 (ppm)

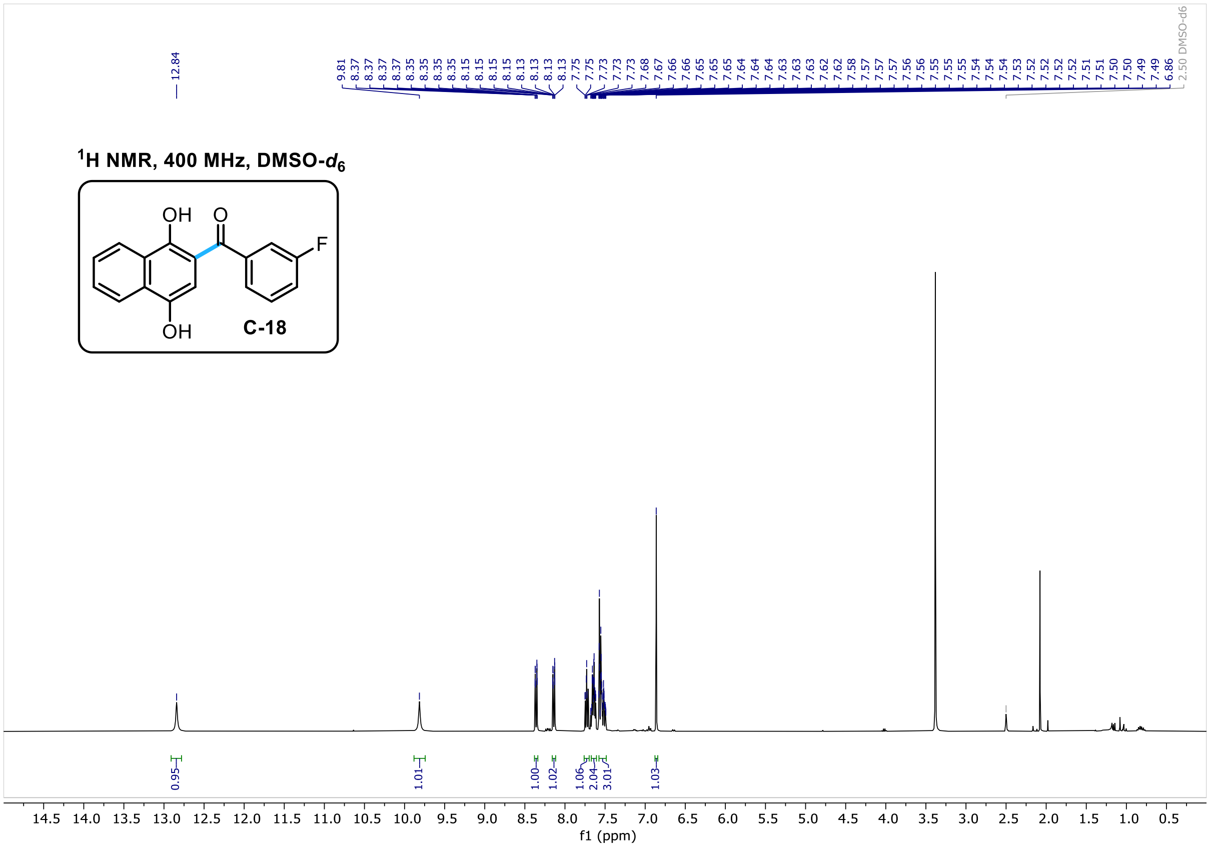

12.84
9.81
8.37
8.35
8.15
8.13
7.75
7.73
7.68
7.67
7.66
7.65
7.64
7.63
7.62
7.58
7.57
7.56
7.55
7.54
7.53
7.52
7.51
7.50
7.49
6.86
2.50 DMSO-d6
^{1}H NMR, 400 MHz, DMSO-d_6
OH
O
F
OH
C-18
0.95
1.01
1.00
1.02
1.06
2.04
3.01
1.03
f1 (ppm)

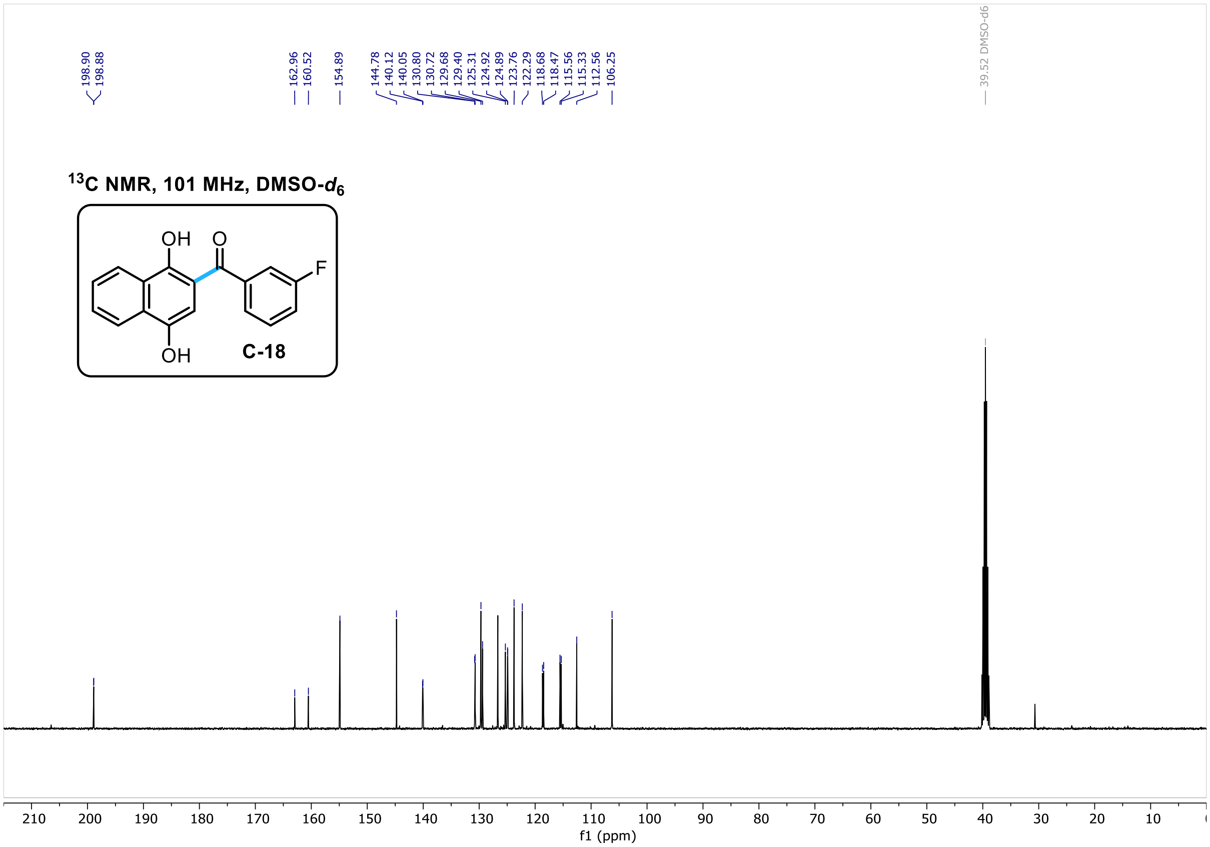

198.90
198.88
162.96
160.52
154.89
144.78
140.12
140.05
130.80
130.72
129.68
129.40
125.31
124.92
124.89
123.76
122.29
118.68
118.47
115.56
115.33
112.56
106.25
39.52 DMSO-d6
^{13}C NMR, 101 MHz, DMSO-d_6
OH
O
F
OH
C-18
f1 (ppm)

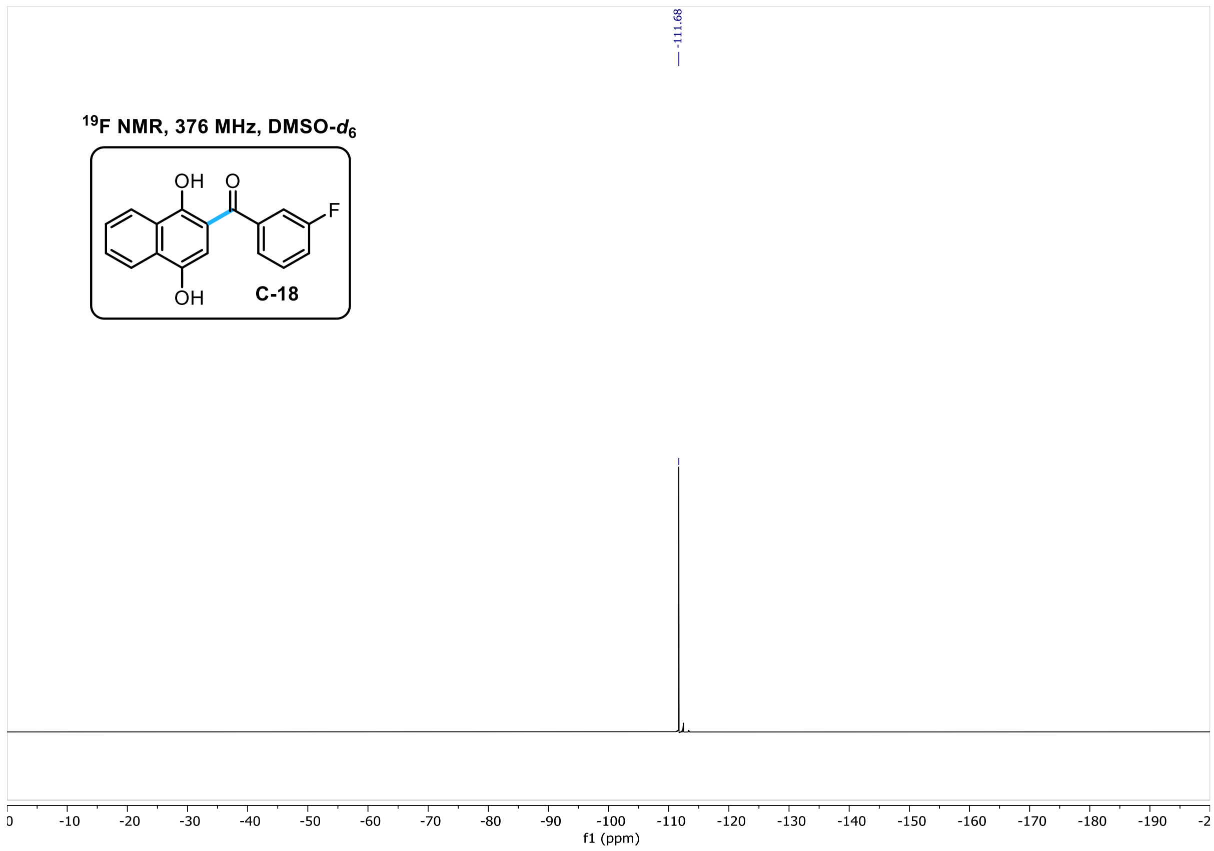

-111.68
19F NMR, 376 MHz, DMSO-d6
OH
O
F
OH
C-18
0
-10
-20
-30
-40
-50
-60
-70
-80
-90
-100
-110
-120
-130
-140
-150
-160
-170
-180
-190
-2
f1 (ppm)

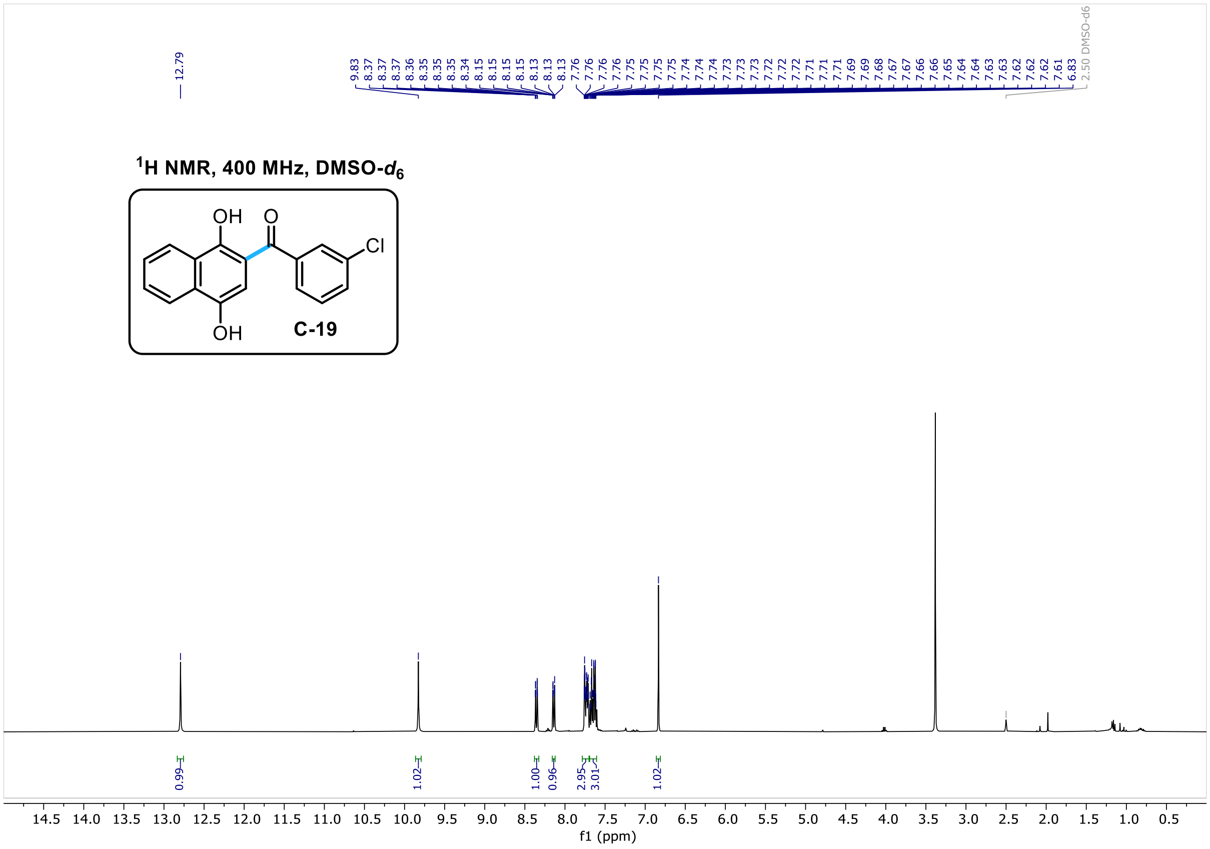
12.79
9.83
8.37
8.37
8.37
8.36
8.35
8.35
8.35
8.34
8.15
8.15
8.15
8.15
8.13
8.13
8.13
7.76
7.76
7.76
7.76
7.75
7.75
7.75
7.74
7.74
7.74
7.73
7.73
7.73
7.72
7.72
7.72
7.71
7.71
7.71
7.69
7.69
7.68
7.67
7.67
7.66
7.66
7.65
7.64
7.64
7.63
7.63
7.62
7.62
7.62
7.61
6.83
2.50 DMSO-d6
^{1}H NMR, 400 MHz, DMSO-d_6
OH
O
Cl
OH
C-19
0.99
1.02
1.00
0.96
2.95
3.01
1.02
14.5 14.0 13.5 13.0 12.5 12.0 11.5 11.0 10.5 10.0 9.5 9.0 8.5 8.0 7.5 7.0 6.5 6.0 5.5 5.0 4.5 4.0 3.5 3.0 2.5 2.0 1.5 1.0 0.5
f1 (ppm)

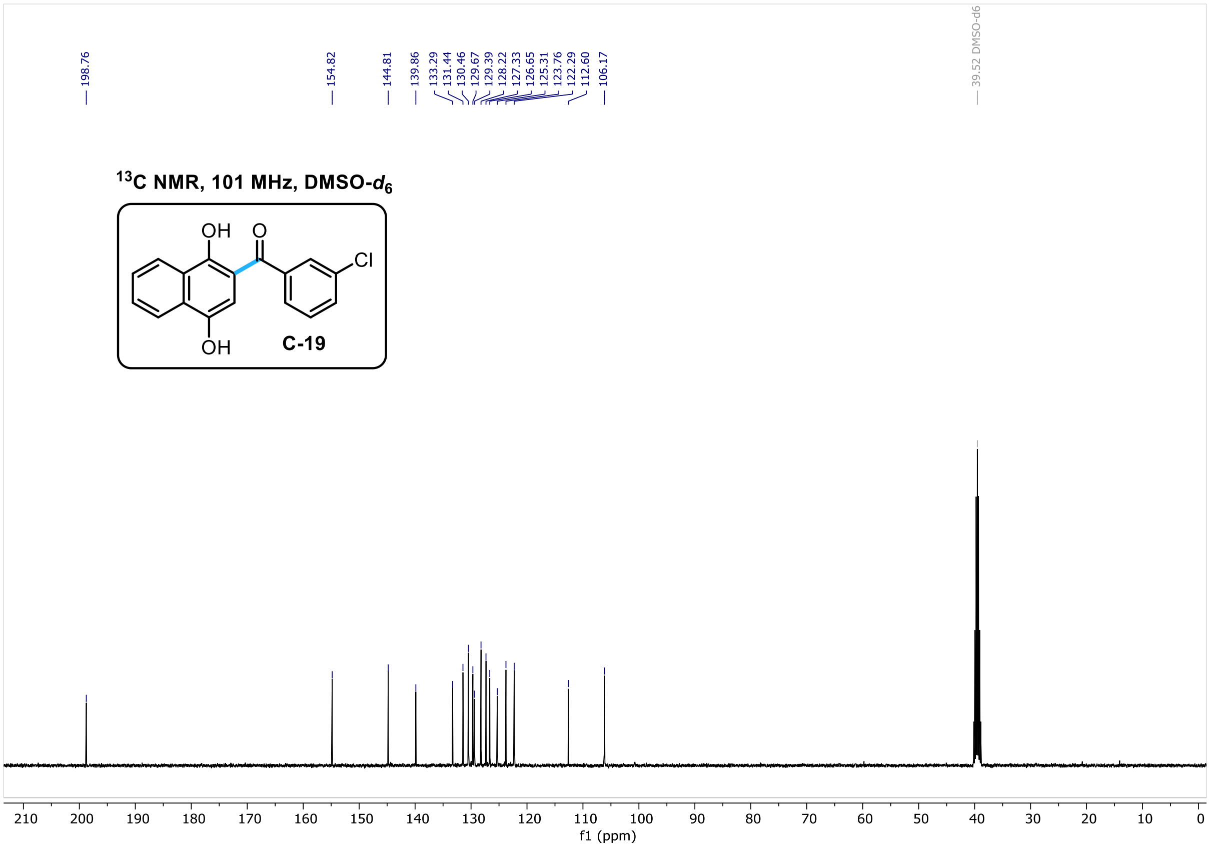
198.76
154.82
144.81
139.86
133.29
131.44
130.46
129.67
129.39
128.22
127.33
126.65
125.31
123.76
122.29
112.60
106.17
39.52 DMSO-d6
^{13}C NMR, 101 MHz, DMSO-d_6
OH
O
Cl
OH
C-19
210 200 190 180 170 160 150 140 130 120 110 100 90 80 70 60 50 40 30 20 10 0
f1 (ppm)

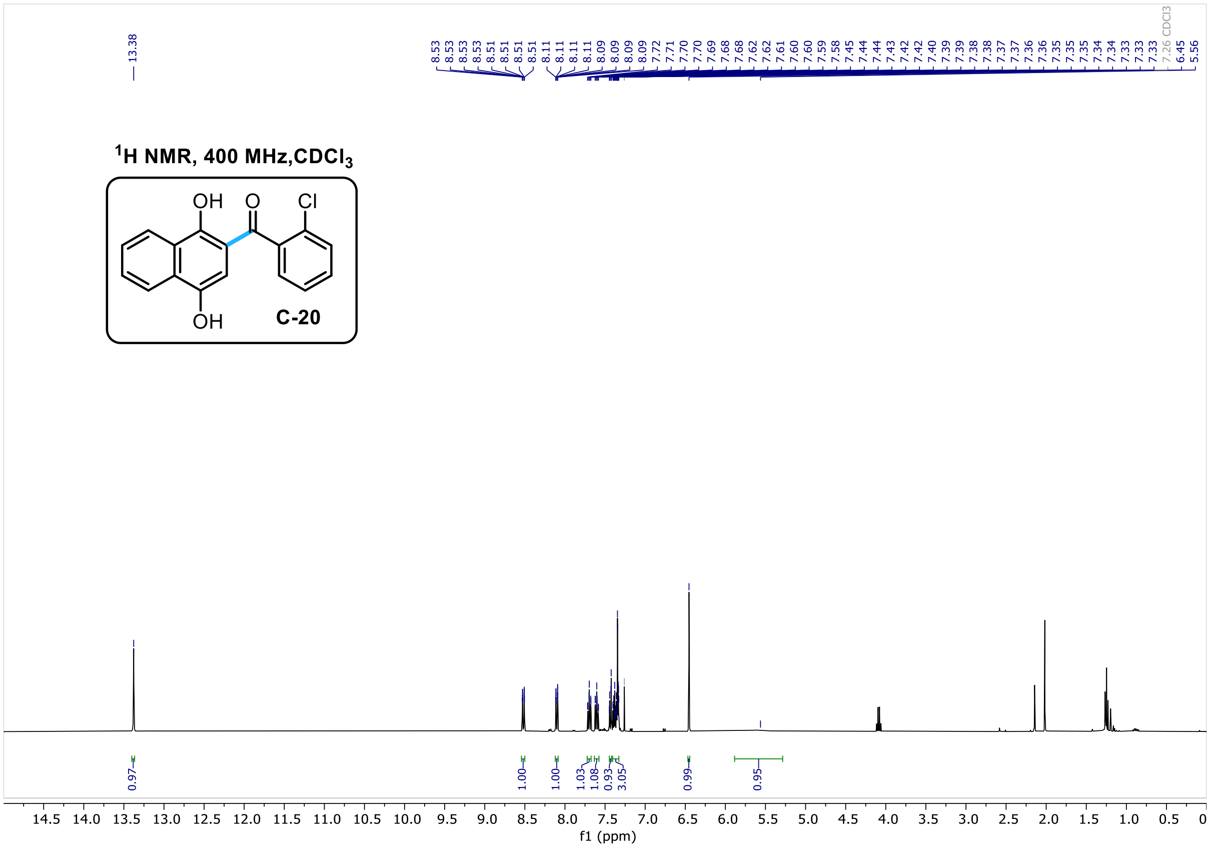

13.38
8.53 8.53 8.53 8.53 8.51 8.51 8.51 8.51 8.11 8.11 8.11 8.11 8.09 8.09 8.09 8.09 7.72 7.71 7.70 7.69 7.68 7.68 7.62 7.62 7.61 7.60 7.60 7.59 7.58 7.45 7.44 7.44 7.43 7.42 7.42 7.40 7.39 7.39 7.38 7.38 7.37 7.37 7.36 7.36 7.35 7.35 7.35 7.34 7.34 7.33 7.33 7.33 7.26 CDCl3 6.45 5.56
1H NMR, 400 MHz,CDCl3
OH
O
Cl
OH
C-20
0.97
1.00
1.00
1.03
1.08
0.93
3.05
0.99
0.95
f1 (ppm)

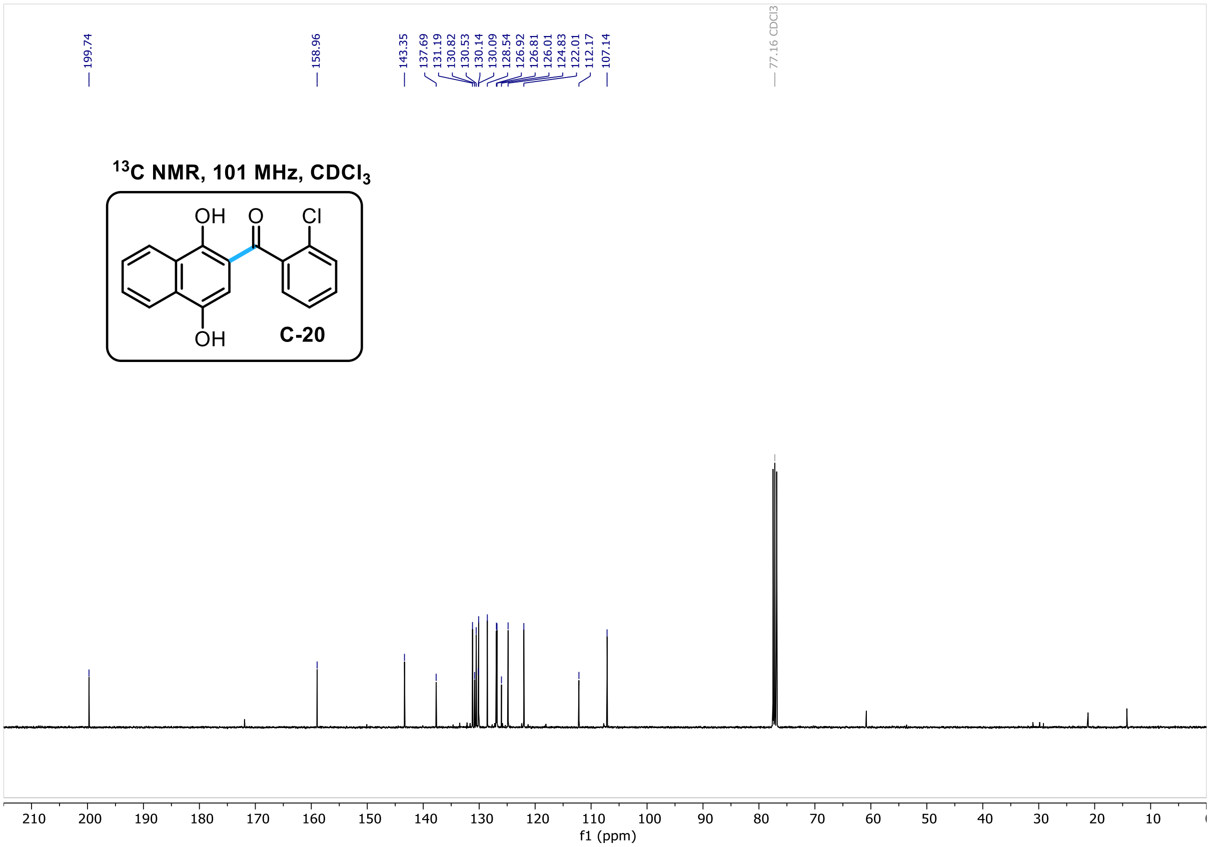

199.74
158.96
143.35 137.69 131.19 130.82 130.53 130.14 130.09 128.54 126.92 126.81 126.01 124.83 122.01 112.17 107.14
77.16 CDCl3
13C NMR, 101 MHz, CDCl3
OH
O
Cl
OH
C-20
f1 (ppm)

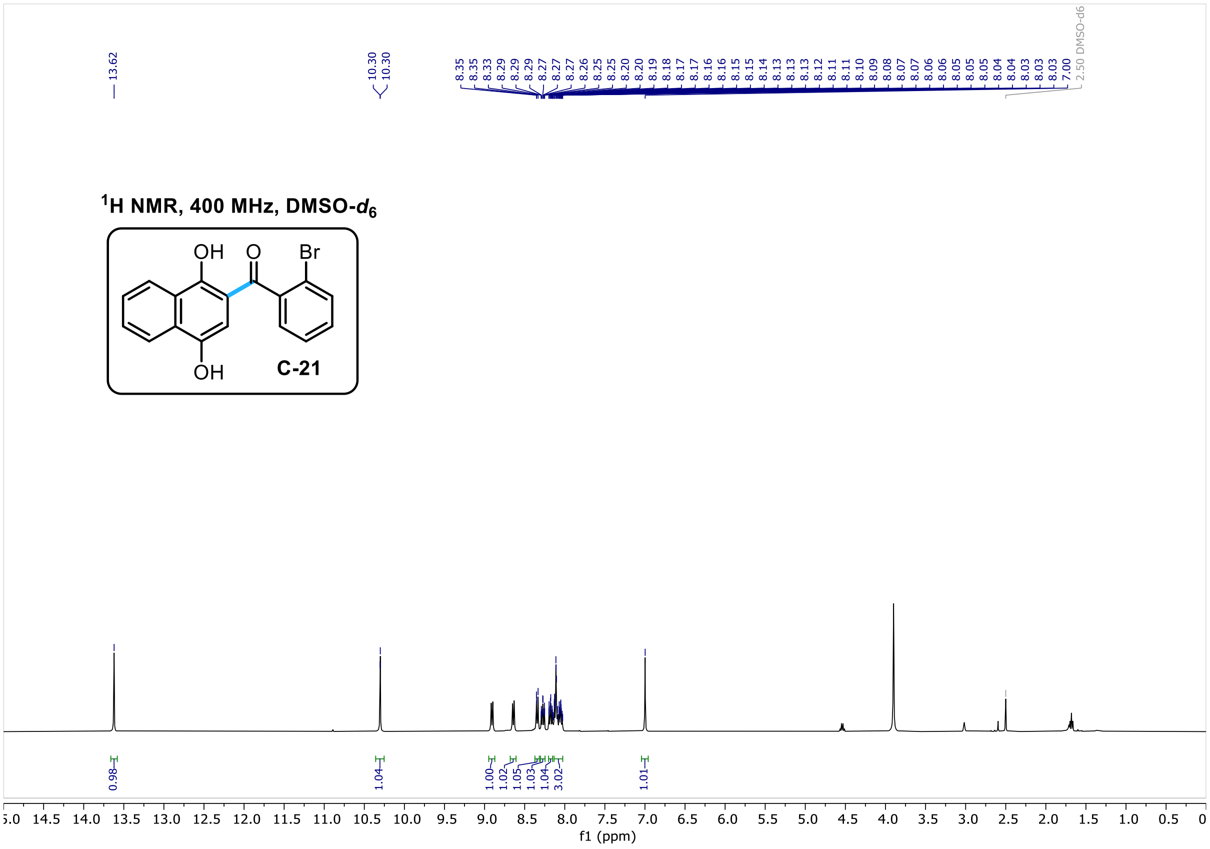
13.62
10.30
10.30
8.35 8.35 8.33 8.29 8.29 8.27 8.27 8.27 8.26 8.25 8.25 8.20 8.20 8.19 8.18 8.17 8.17 8.16 8.16 8.15 8.15 8.14 8.13 8.13 8.13 8.12 8.11 8.11 8.10 8.09 8.08 8.07 8.07 8.06 8.06 8.05 8.05 8.05 8.04 8.04 8.03 8.03 8.03 7.00
2.50 DMSO-d6
¹H NMR, 400 MHz, DMSO-d6
OH
O
Br
OH
C-21
0.98
1.04
1.00
1.02
1.05
1.03
1.04
3.02
1.01
f1 (ppm)

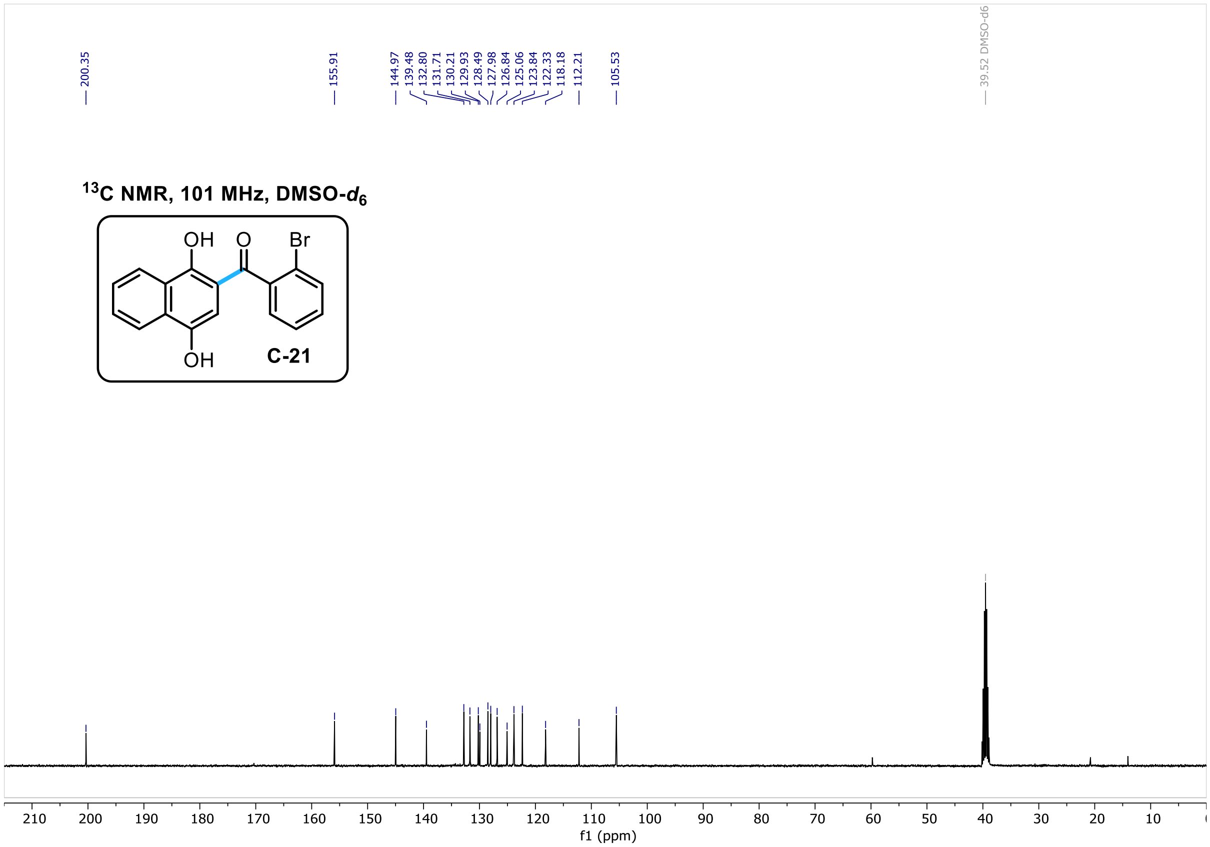
200.35
155.91
144.97 139.48 132.80 131.71 130.21 129.93 128.49 127.98 126.84 125.06 123.84 122.33 118.18
112.21
105.53
39.52 DMSO-d6
¹³C NMR, 101 MHz, DMSO-d6
OH
O
Br
OH
C-21
f1 (ppm)

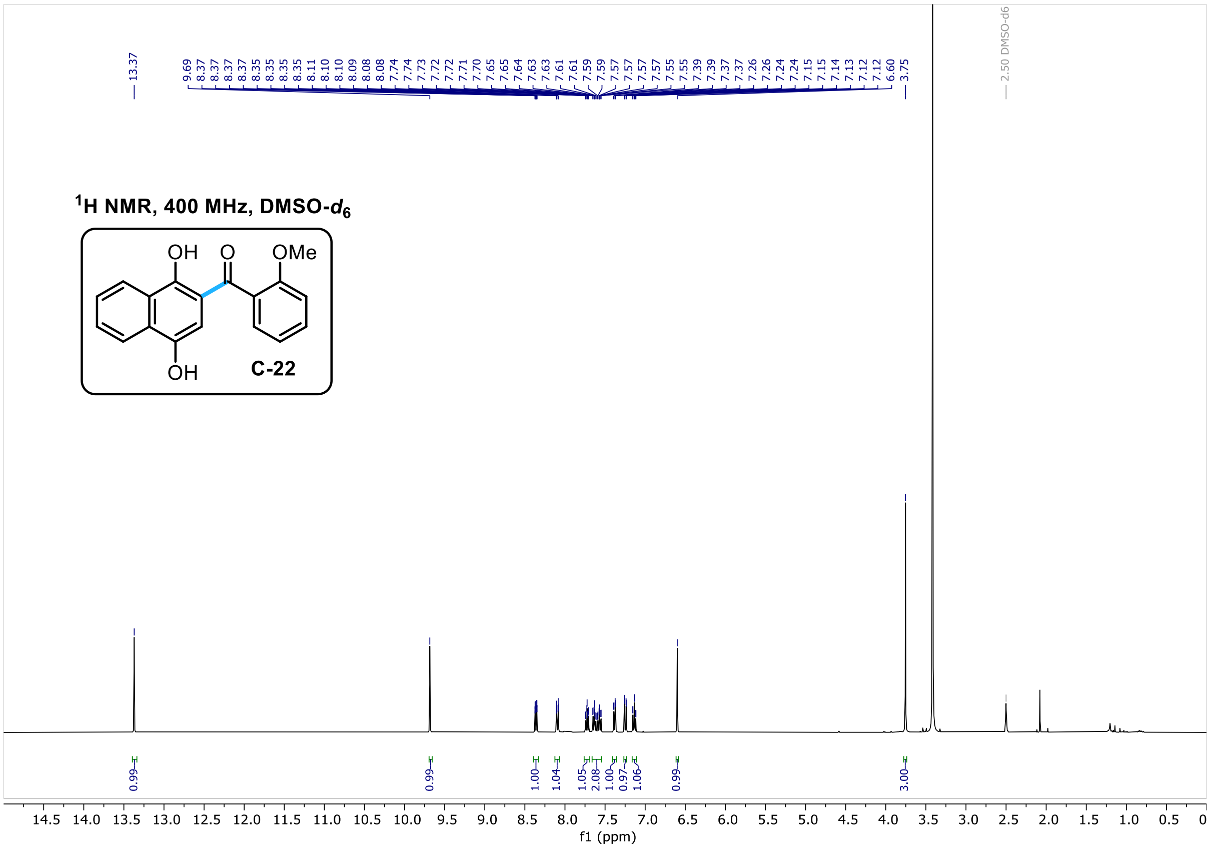
13.37
9.69 8.37 8.37 8.37 8.37 8.35 8.35 8.35 8.35 8.11 8.10 8.10 8.09 8.08 8.08 7.74 7.74 7.73 7.72 7.72 7.71 7.70 7.65 7.65 7.64 7.63 7.63 7.61 7.61 7.59 7.59 7.57 7.57 7.57 7.57 7.55 7.55 7.39 7.39 7.37 7.37 7.26 7.26 7.24 7.24 7.15 7.15 7.14 7.13 7.12 7.12 6.60
3.75
2.50 DMSO-d6
1H NMR, 400 MHz, DMSO-d6
OH
O
OMe
OH
C-22
0.99 0.99 1.00 1.04 1.05 2.08 1.00 0.97 1.06 0.99 3.00
14.5 14.0 13.5 13.0 12.5 12.0 11.5 11.0 10.5 10.0 9.5 9.0 8.5 8.0 7.5 7.0 6.5 6.0 5.5 5.0 4.5 4.0 3.5 3.0 2.5 2.0 1.5 1.0 0.5 0
f1 (ppm)

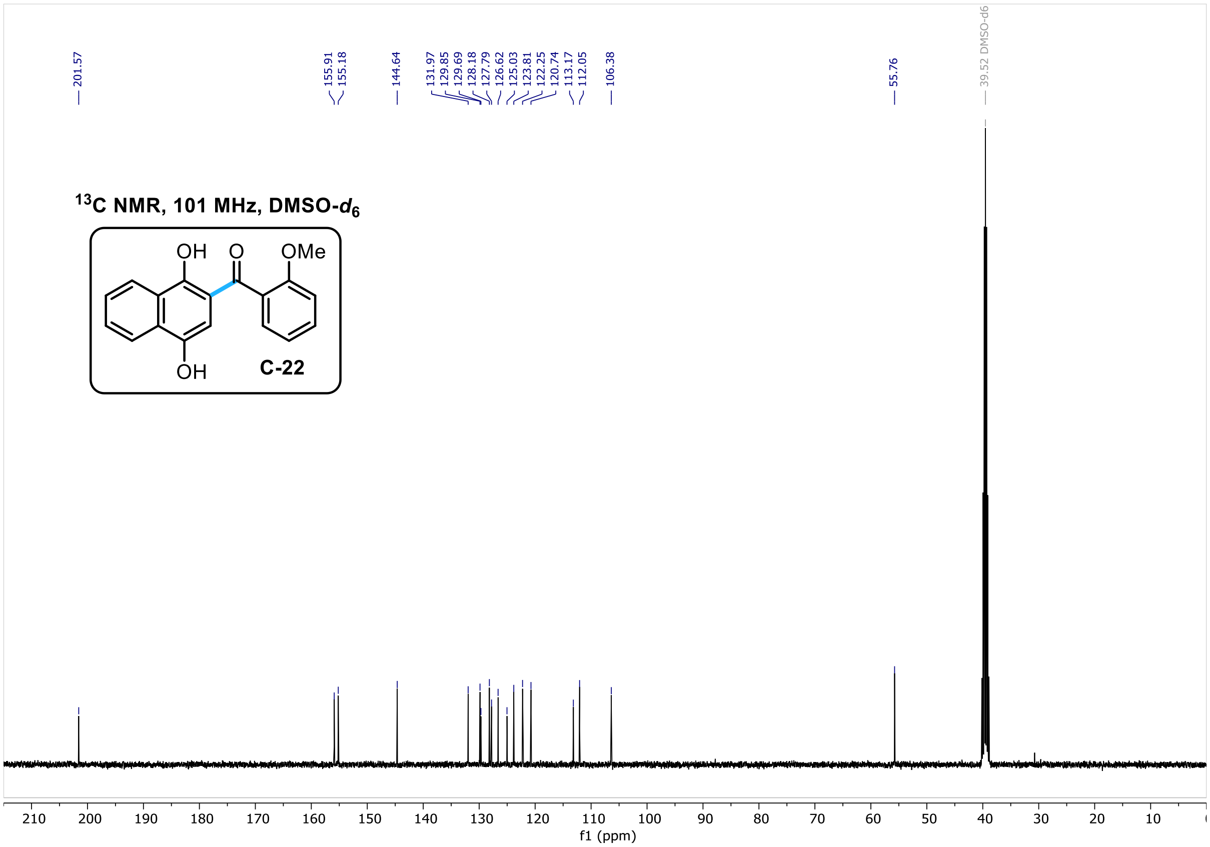
201.57
155.91 155.18
144.64
131.97 129.85 129.69 128.18 127.79 126.62 125.03 123.81 122.25 120.74 113.17 112.05
106.38
55.76
39.52 DMSO-d6
13C NMR, 101 MHz, DMSO-d6
OH
O
OMe
OH
C-22
210 200 190 180 170 160 150 140 130 120 110 100 90 80 70 60 50 40 30 20 10
f1 (ppm)

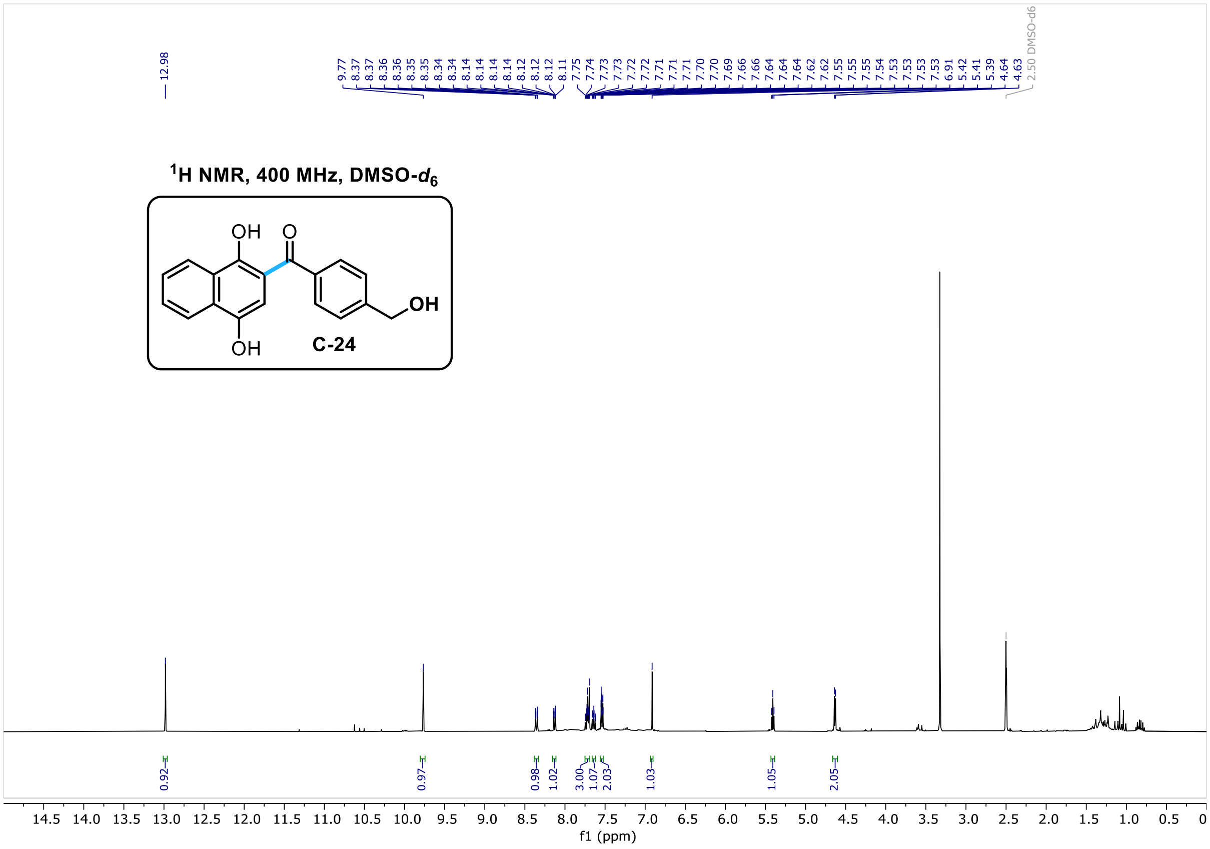
12.98
9.77 8.37 8.37 8.36 8.36 8.35 8.35 8.34 8.34 8.14 8.14 8.14 8.14 8.12 8.12 8.11 7.75 7.74 7.73 7.73 7.72 7.72 7.71 7.71 7.71 7.70 7.69 7.66 7.66 7.64 7.64 7.64 7.62 7.62 7.55 7.55 7.55 7.54 7.53 7.53 7.53 6.91 5.42 5.41 5.39 4.64 4.63
2.50 DMSO-d6
1H NMR, 400 MHz, DMSO-d6
OH
O
OH
OH
C-24
0.92
0.97
0.98
1.02
3.00
1.07
2.03
1.03
1.05
2.05
f1 (ppm)

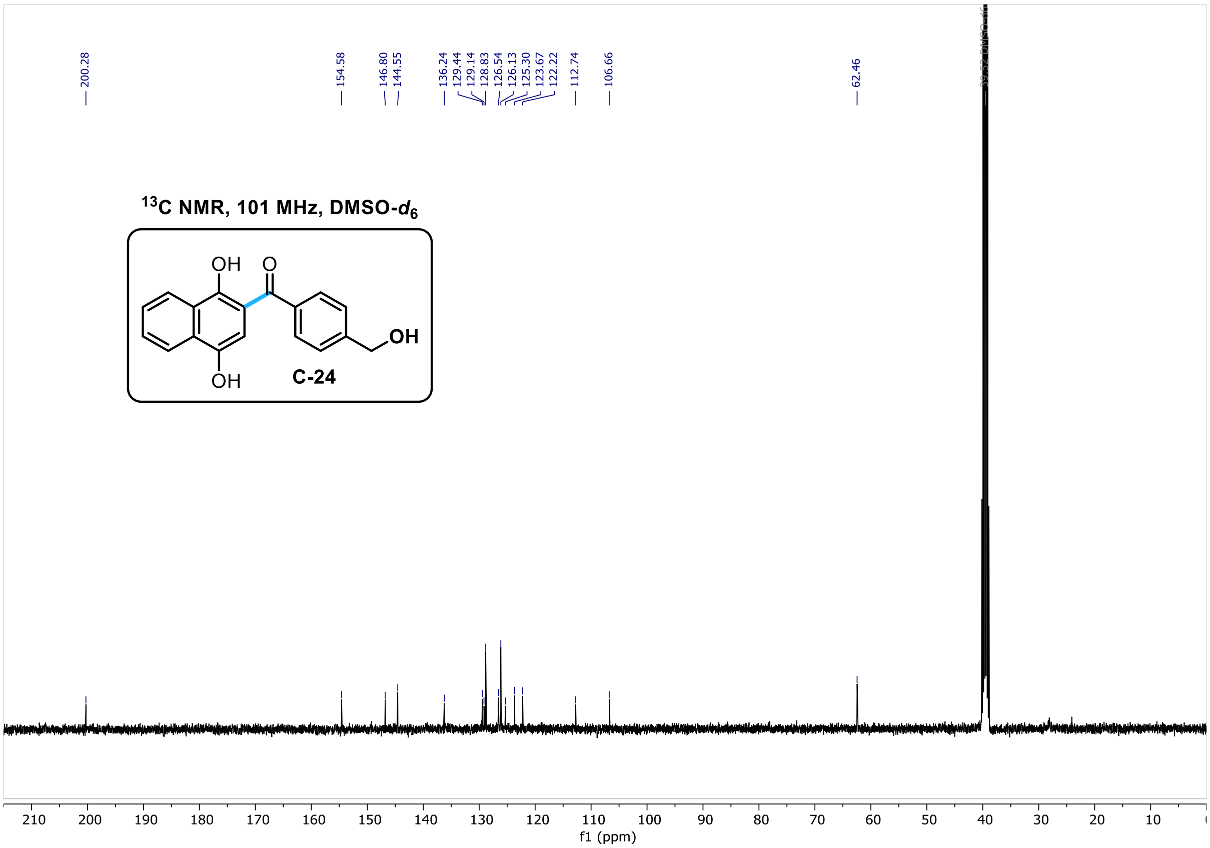
200.28
154.58
146.80
144.55
136.24
129.44
129.14
128.83
126.54
126.13
125.30
123.67
122.22
112.74
106.66
62.46
13C NMR, 101 MHz, DMSO-d6
OH
O
OH
OH
C-24
f1 (ppm)

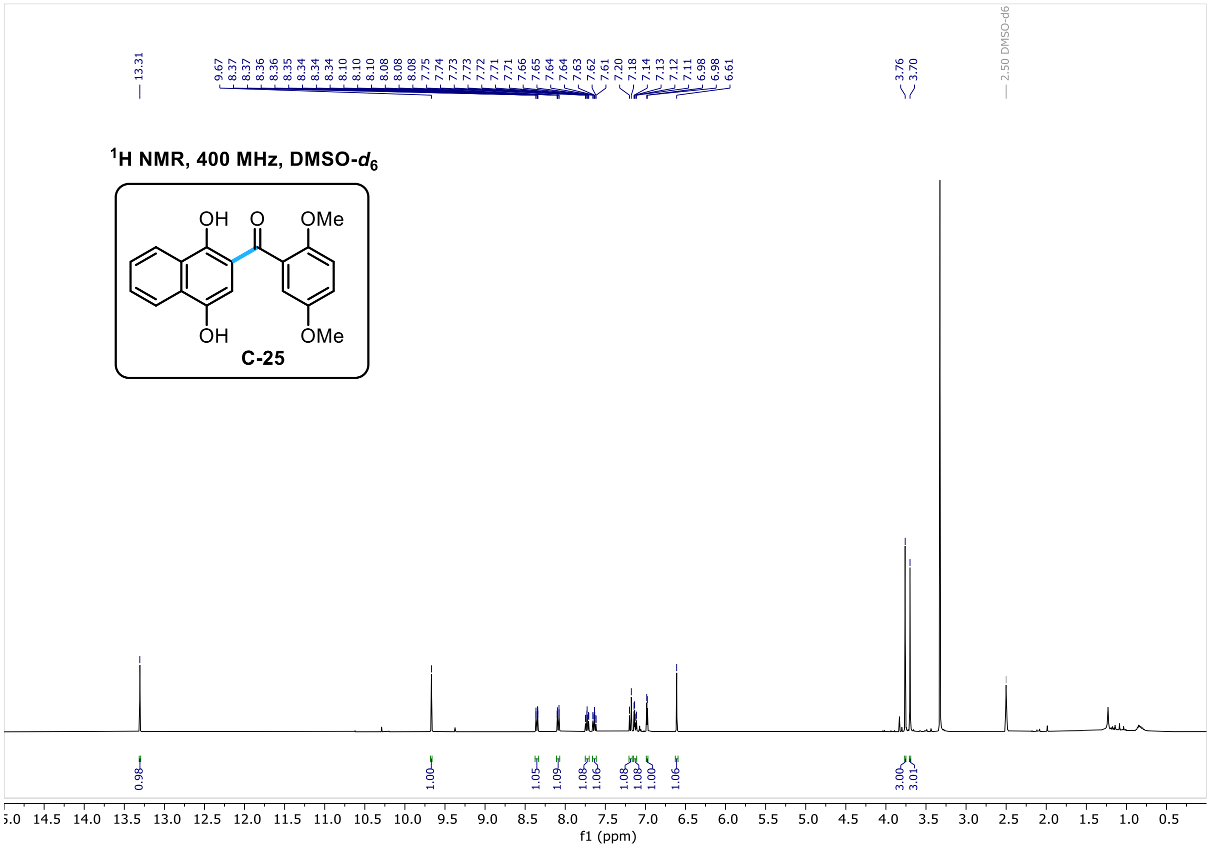
1H NMR, 400 MHz, DMSO-d6
C-25
f1 (ppm)

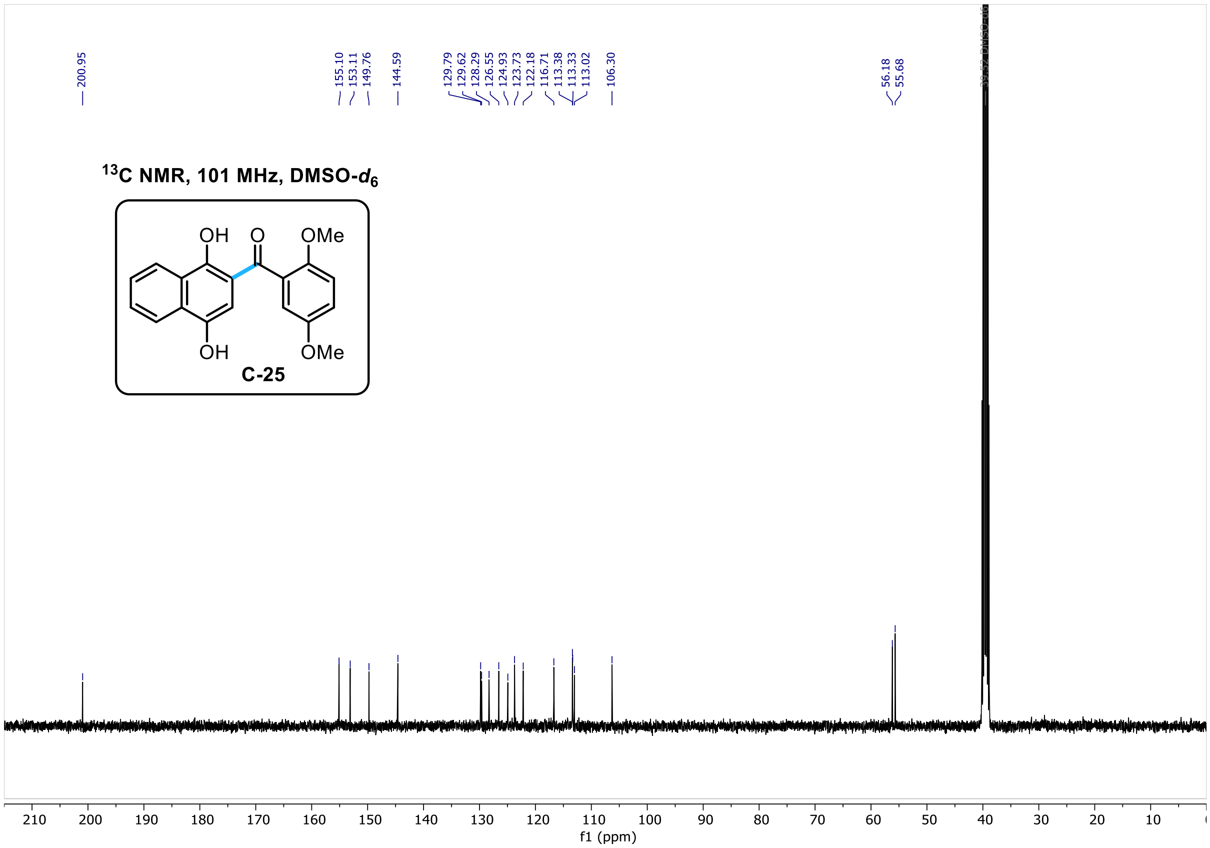
13C NMR, 101 MHz, DMSO-d6
C-25
f1 (ppm)

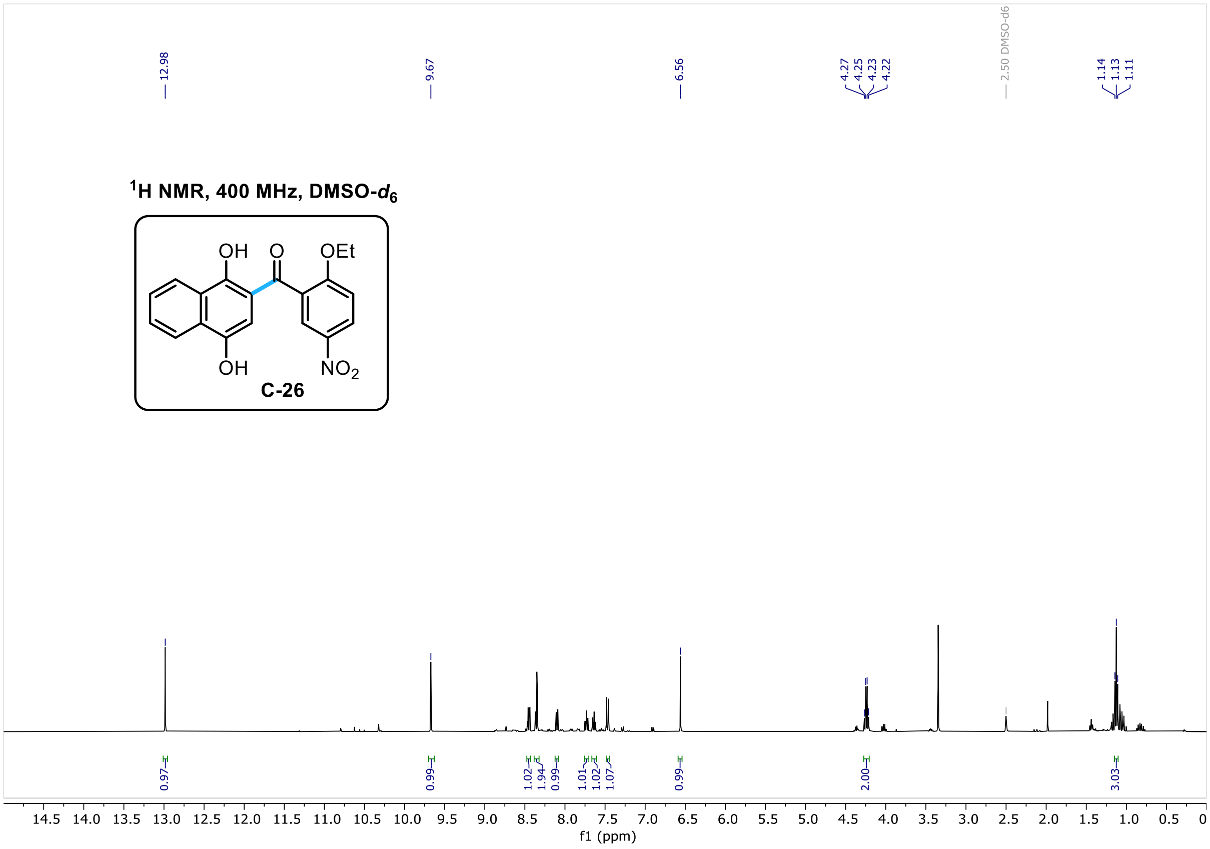

12.98
9.67
6.56
4.27
4.25
4.23
4.22
2.50 DMSO-d6
1.14
1.13
1.11
^{1}H NMR, 400 MHz, DMSO-d_6
OH
O
OEt
OH
NO_2
C-26
0.97
0.99
1.02
1.94
0.99
1.01
1.02
1.07
0.99
2.00
3.03
f1 (ppm)

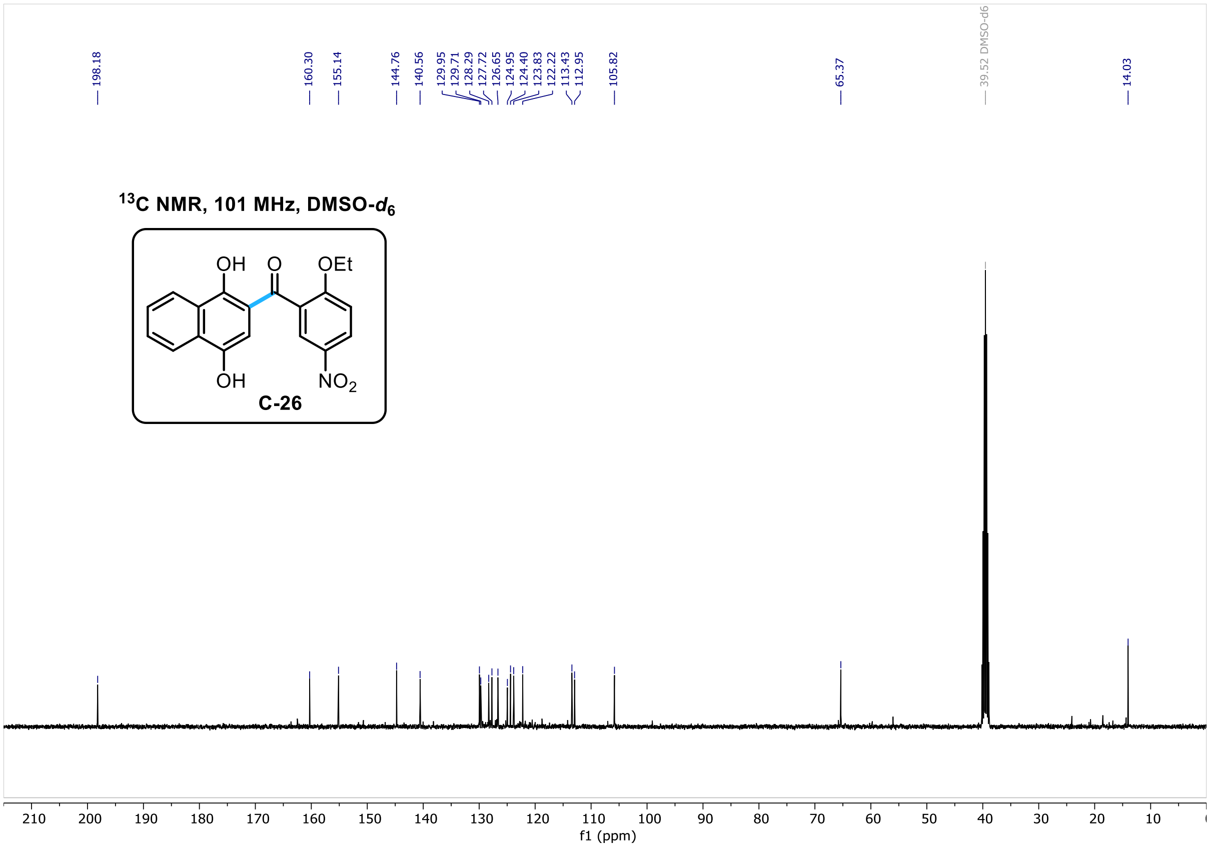

198.18
160.30
155.14
144.76
140.56
129.95
129.71
128.29
127.72
126.65
124.95
124.40
123.83
122.22
113.43
112.95
105.82
65.37
39.52 DMSO-d6
14.03
^{13}C NMR, 101 MHz, DMSO-d_6
OH
O
OEt
OH
NO_2
C-26
f1 (ppm)

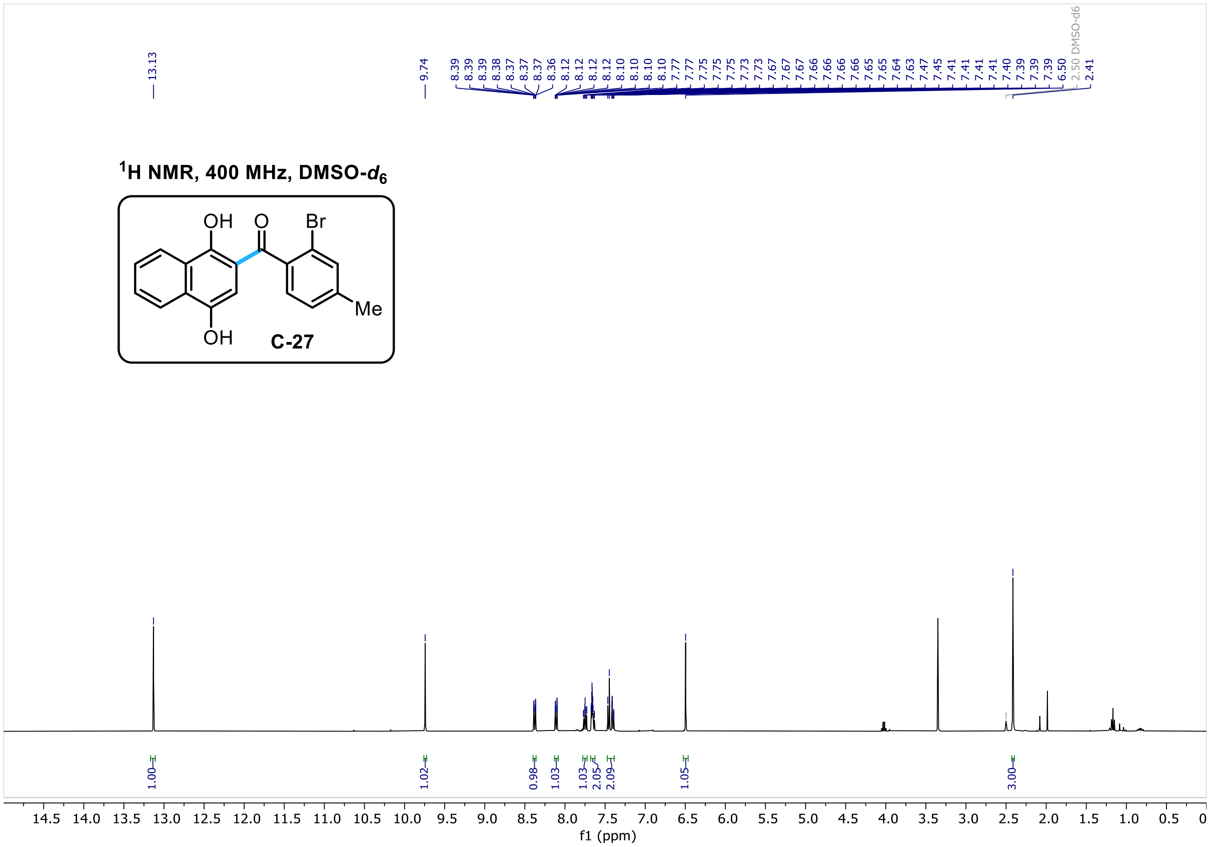

13.13
9.74
8.39 8.39 8.39 8.38 8.37 8.37 8.37 8.36 8.12 8.12 8.12 8.12 8.10 8.10 8.10 8.10 7.77 7.77 7.75 7.75 7.73 7.73 7.67 7.67 7.67 7.66 7.66 7.66 7.65 7.65 7.64 7.63 7.47 7.45 7.41 7.41 7.41 7.41 7.40 7.39 7.39 7.39 6.50
2.50 DMSO-d6
2.41
1H NMR, 400 MHz, DMSO-d6
OH O Br
Me
OH
C-27
1.00 1.02 0.98 1.03 1.03 2.05 2.09 1.05 3.00
f1 (ppm)

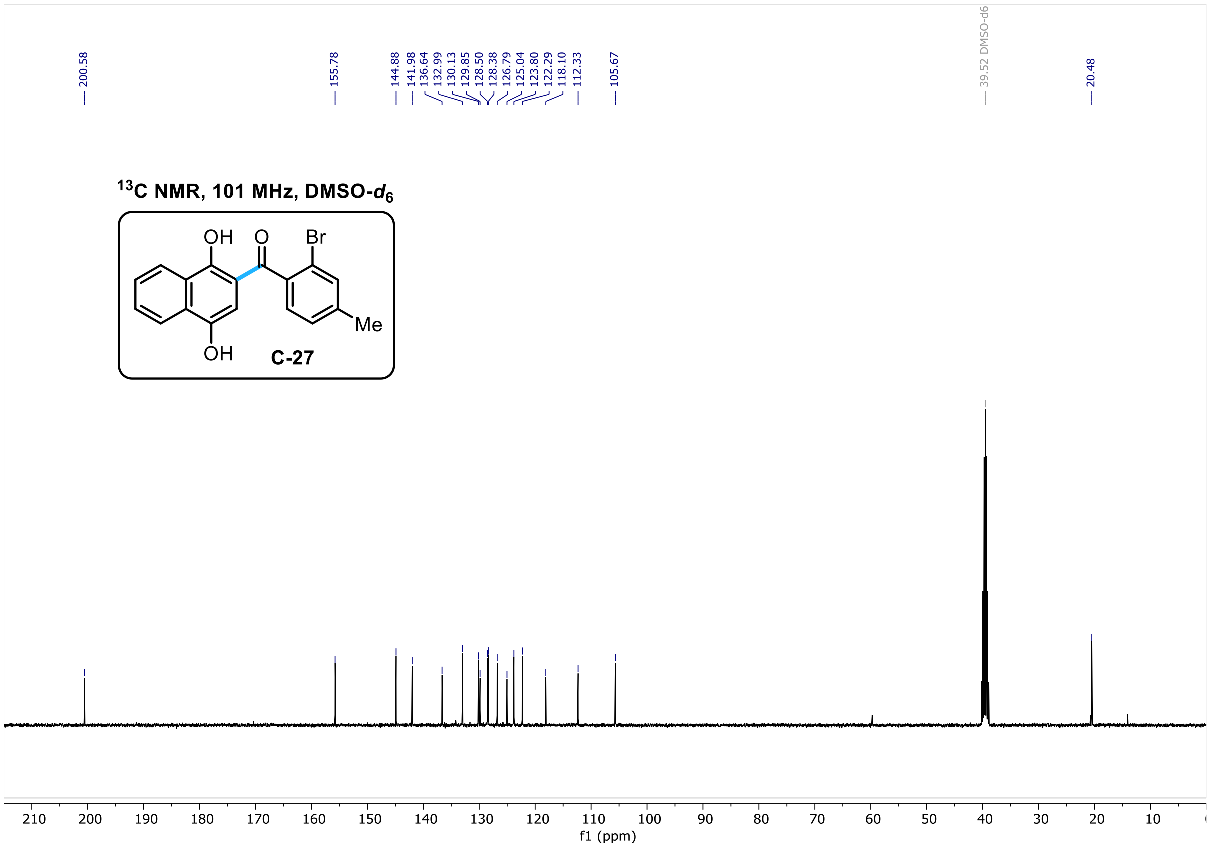

200.58
155.78
144.88 141.98 136.64 132.99 130.13 129.85 128.50 128.38 126.79 125.04 123.80 122.29 118.10 112.33
105.67
39.52 DMSO-d6
20.48
13C NMR, 101 MHz, DMSO-d6
OH O Br
Me
OH
C-27
f1 (ppm)

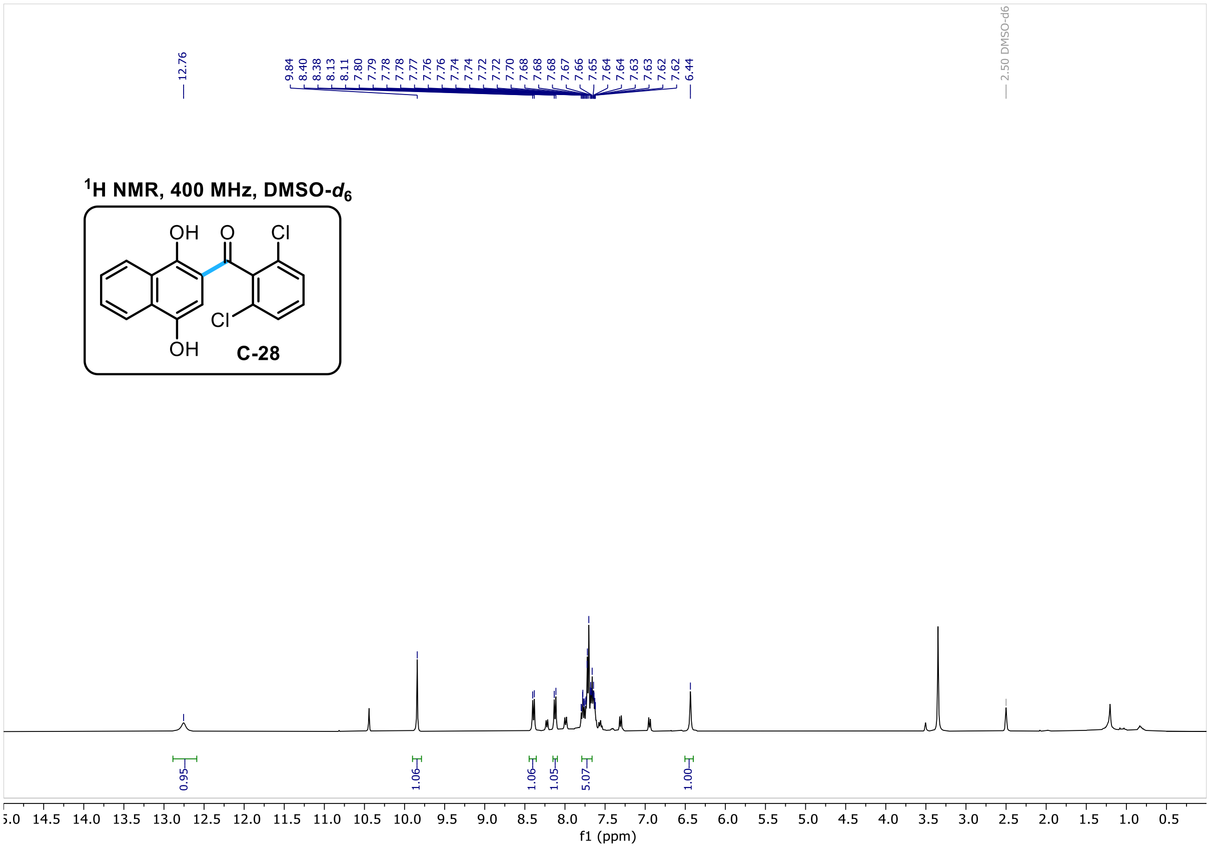

12.76
9.84 8.40 8.38 8.13 8.11 7.80 7.79 7.78 7.78 7.77 7.77 7.76 7.76 7.74 7.74 7.72 7.72 7.70 7.68 7.68 7.68 7.67 7.66 7.65 7.64 7.64 7.63 7.63 7.62 7.62 6.44
2.50 DMSO-d6
1H NMR, 400 MHz, DMSO-d6
OH
O
Cl
Cl
OH
C-28
0.95
1.06
1.06
1.05
5.07
1.00
f1 (ppm)

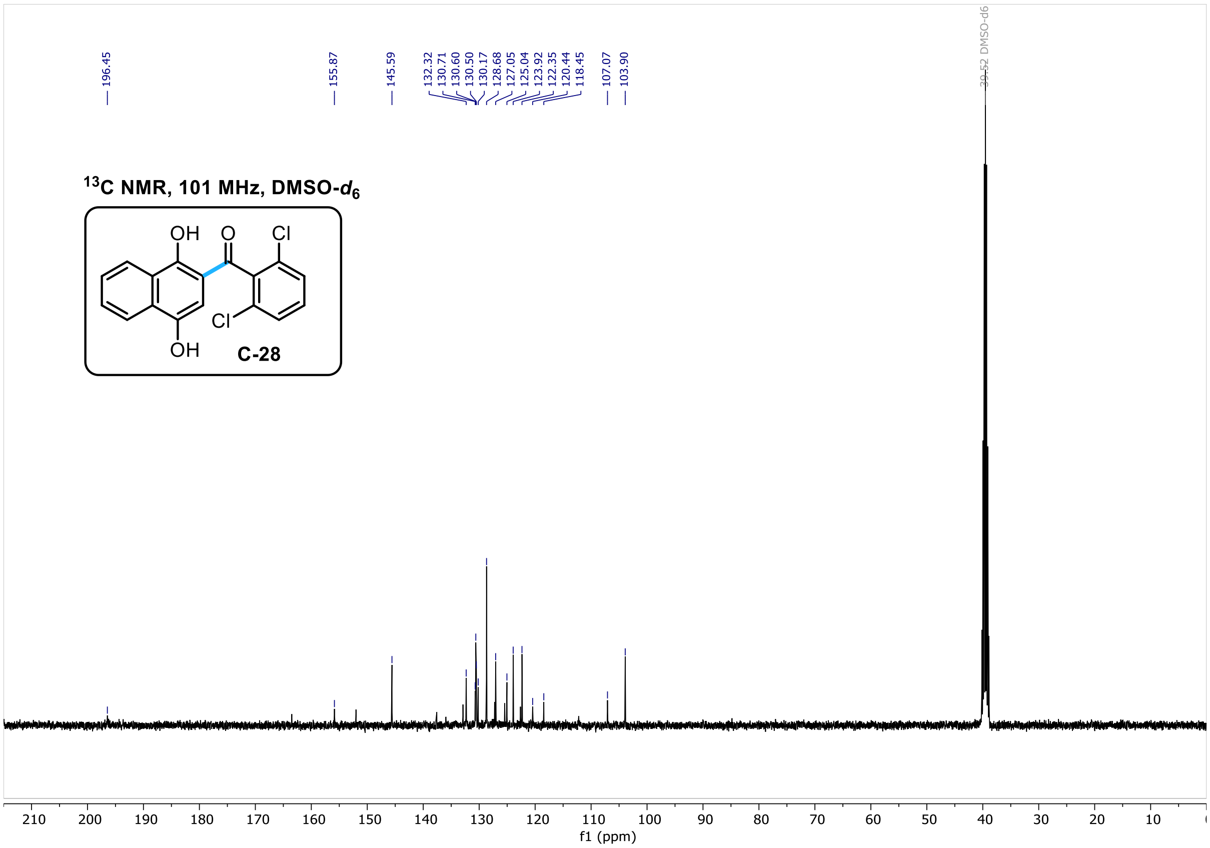

196.45
155.87
145.59
132.32 130.71 130.60 130.50 130.17 128.68 127.05 125.04 123.92 122.35 120.44 118.45
107.07
103.90
39.52 DMSO-d6
13C NMR, 101 MHz, DMSO-d6
OH
O
Cl
Cl
OH
C-28
f1 (ppm)

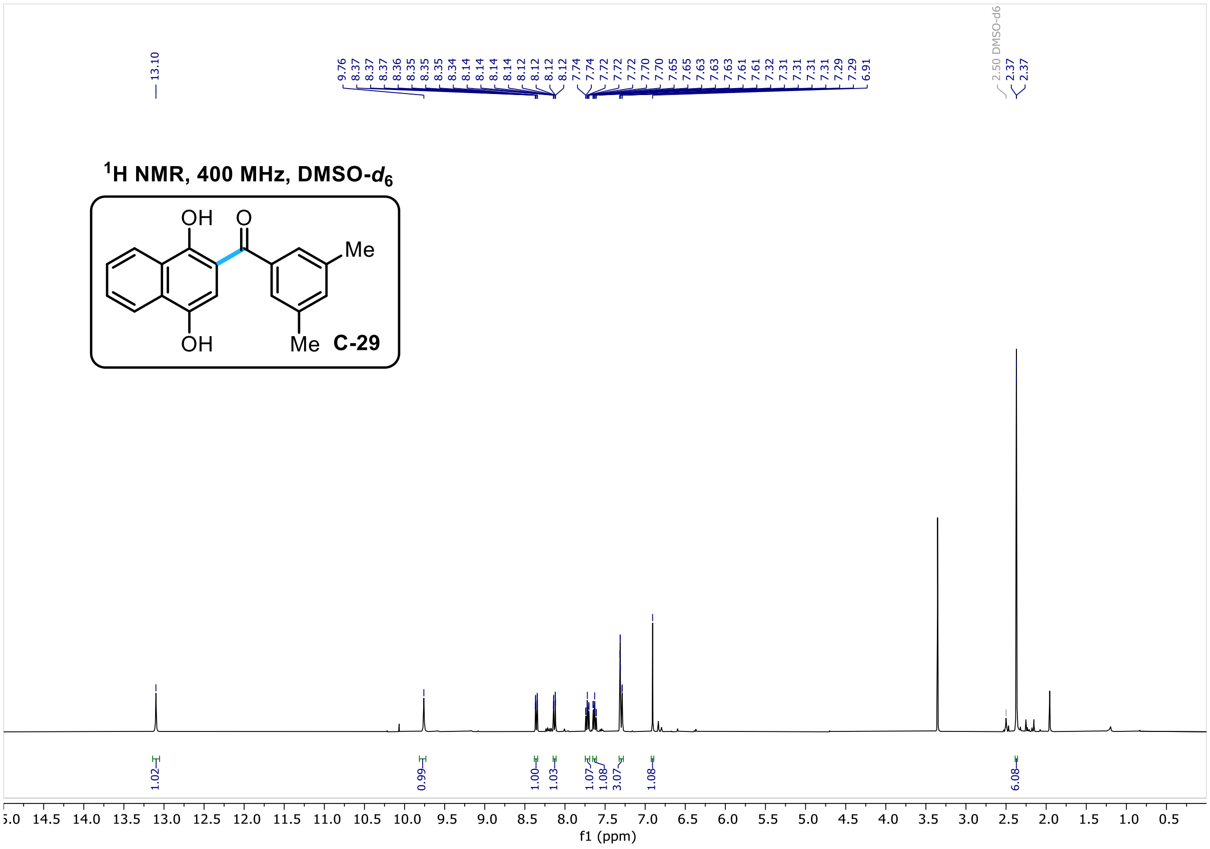
13.10
9.76
8.37
8.37
8.37
8.36
8.35
8.35
8.35
8.34
8.14
8.14
8.14
8.14
8.12
8.12
8.12
8.12
7.74
7.74
7.72
7.72
7.72
7.70
7.70
7.65
7.65
7.63
7.63
7.63
7.61
7.61
7.32
7.31
7.31
7.31
7.31
7.29
7.29
6.91
2.50 DMSO-d6
2.37
2.37
1H NMR, 400 MHz, DMSO-d6
OH
O
Me
OH
Me
C-29
1.02
0.99
1.00
1.03
1.07
1.08
3.07
1.08
6.08
f1 (ppm)

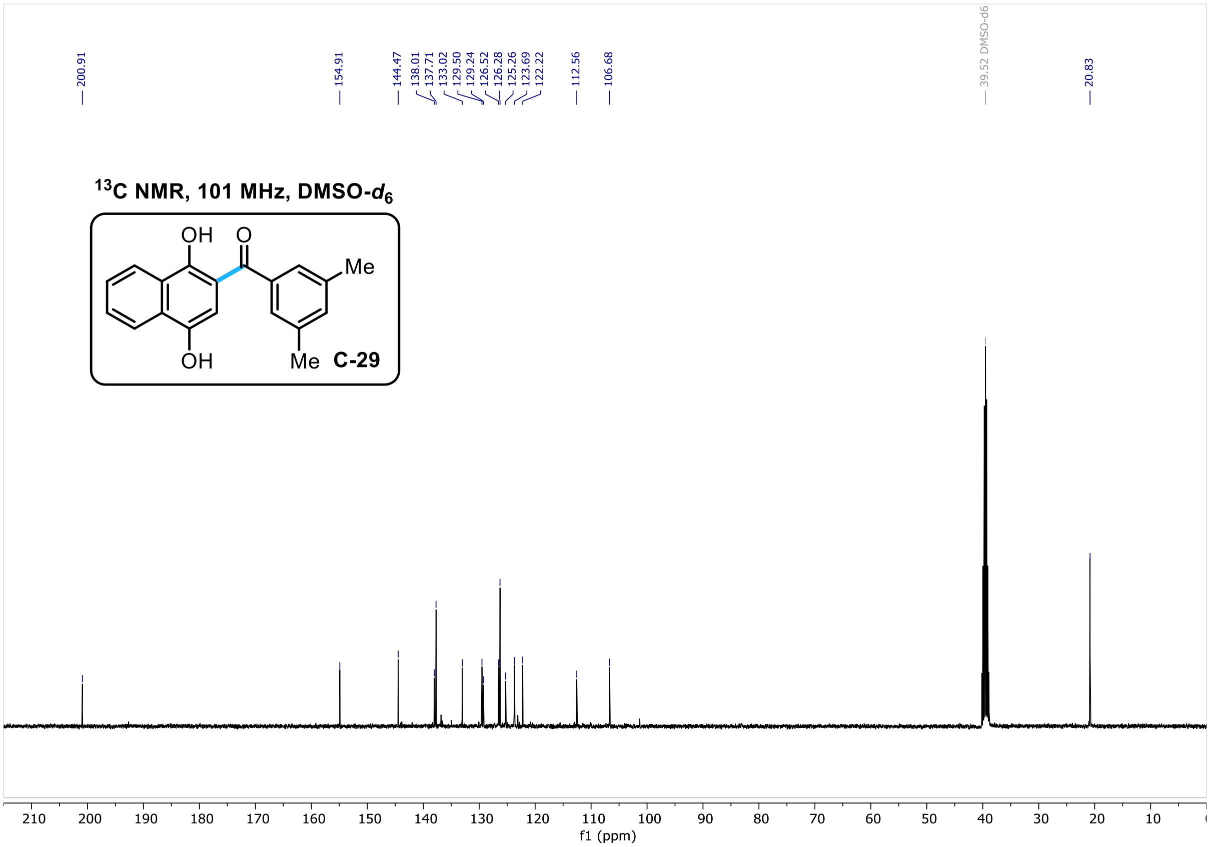
200.91
154.91
144.47
138.01
137.71
133.02
129.50
129.24
126.52
126.28
125.26
123.69
122.22
112.56
106.68
39.52 DMSO-d6
20.83
13C NMR, 101 MHz, DMSO-d6
OH
O
Me
OH
Me
C-29
f1 (ppm)

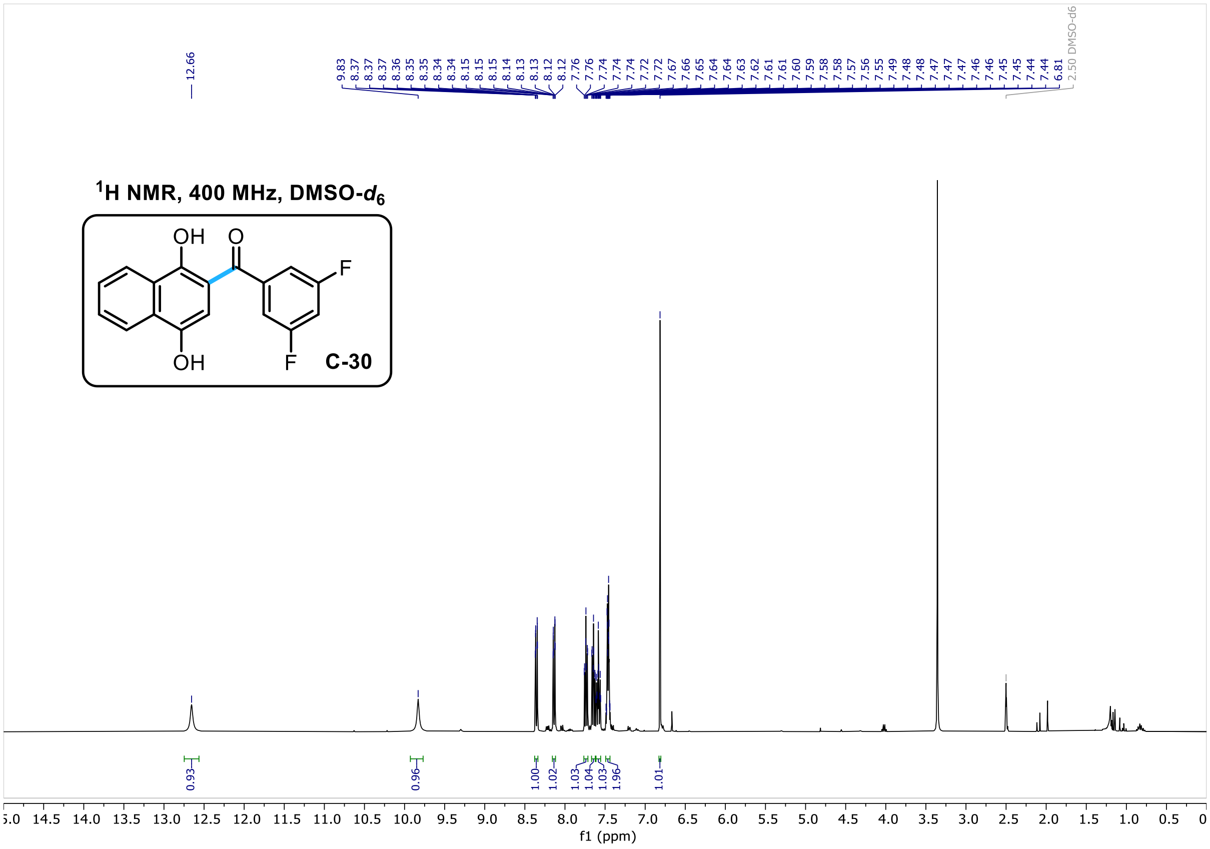
12.66
9.83 8.37 8.37 8.37 8.36 8.35 8.35 8.34 8.34 8.15 8.15 8.15 8.14 8.13 8.13 8.12 8.12 7.76 7.76 7.74 7.74 7.74 7.72 7.72 7.67 7.66 7.65 7.64 7.64 7.63 7.62 7.61 7.60 7.59 7.58 7.58 7.57 7.56 7.55 7.49 7.48 7.47 7.47 7.47 7.46 7.46 7.45 7.45 7.44 7.44 6.81
2.50 DMSO-d6
1H NMR, 400 MHz, DMSO-d6
OH
O
F
OH
F
C-30
0.93
0.96
1.00
1.02
1.03
1.04
1.03
1.96
1.01
f1 (ppm)

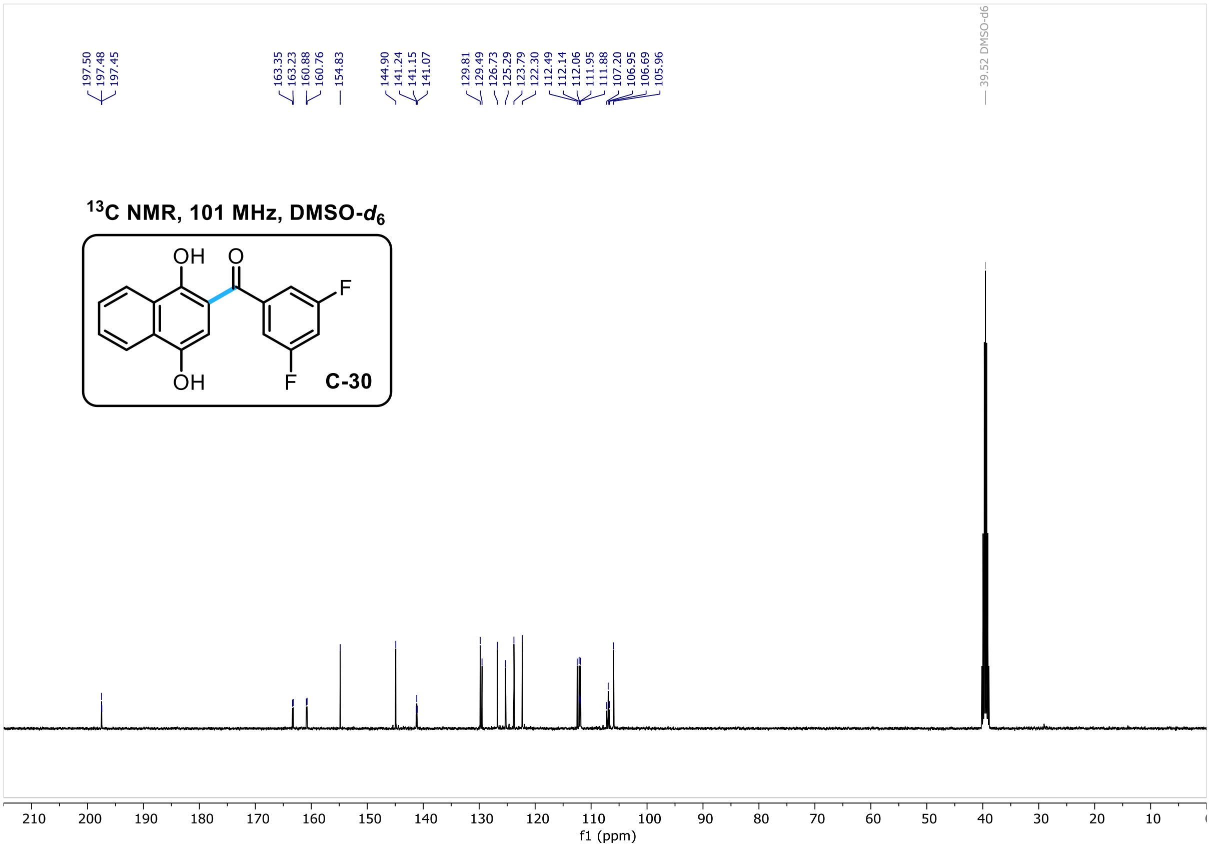
197.50 197.48 197.45
163.35 163.23 160.88 160.76
154.83
144.90 141.24 141.15 141.07
129.81 129.49 126.73 125.29 123.79 122.30 112.49 112.14 112.06 111.95 111.88 107.20 106.95 106.69 105.96
39.52 DMSO-d6
13C NMR, 101 MHz, DMSO-d6
OH
O
F
OH
F
C-30
f1 (ppm)

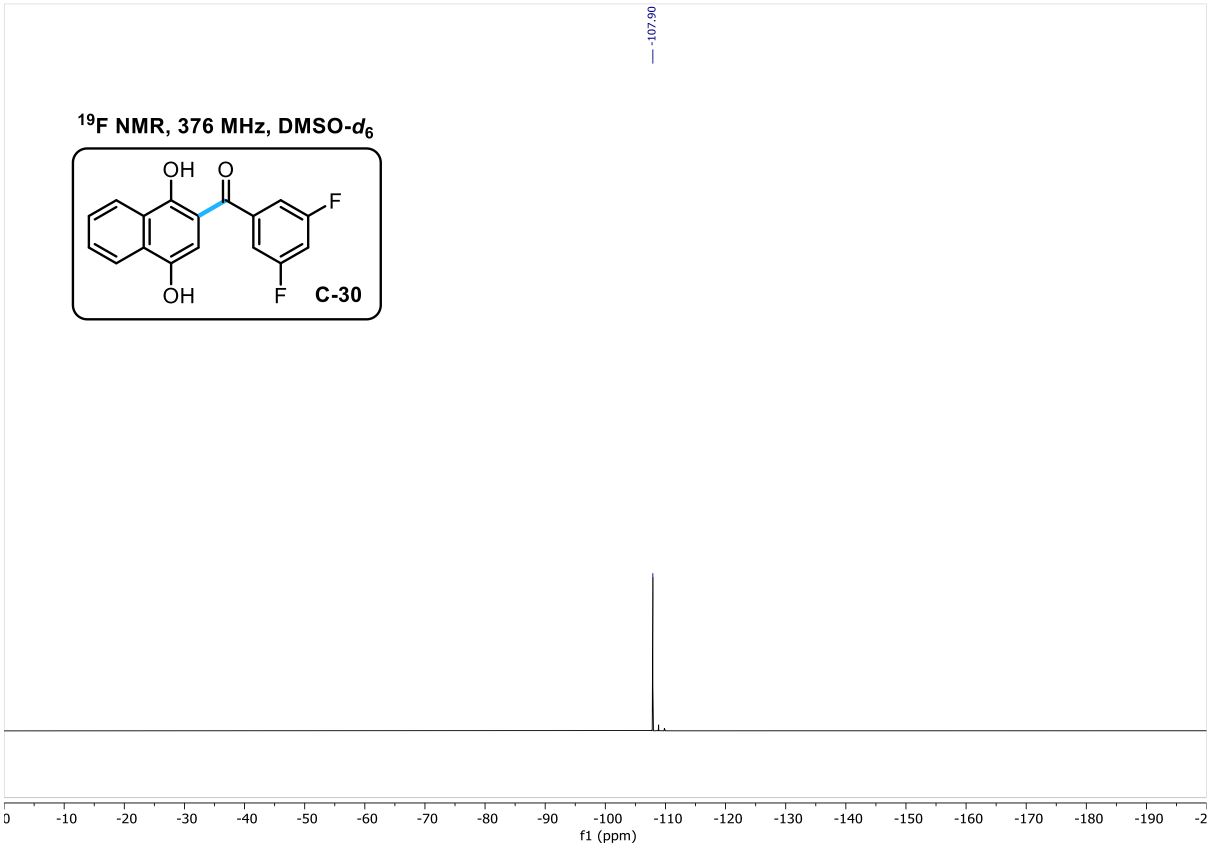
-107.90
^{19}F NMR, 376 MHz, DMSO-d_6
OH
O
F
OH
F
C-30
0
-10
-20
-30
-40
-50
-60
-70
-80
-90
-100
-110
-120
-130
-140
-150
-160
-170
-180
-190
-2
f1 (ppm)

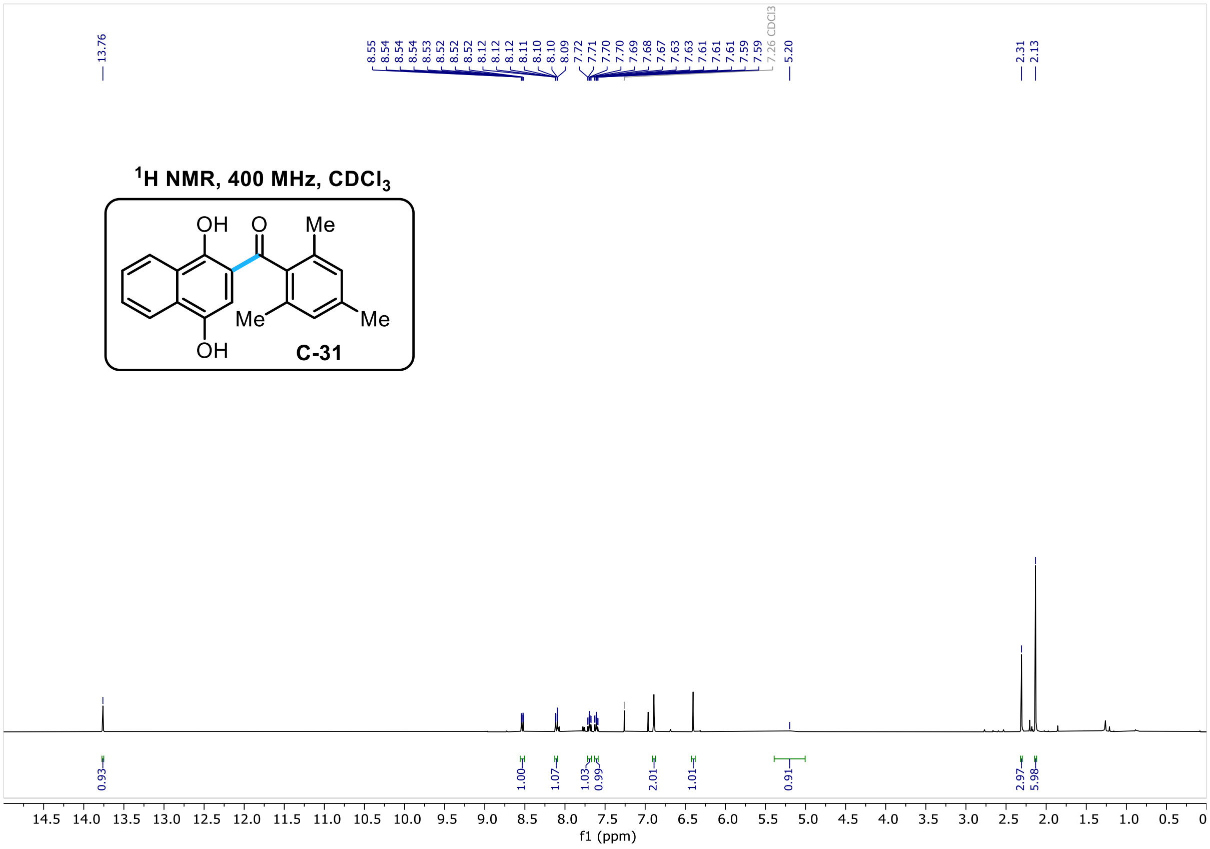
13.76
8.55 8.54 8.54 8.53 8.52 8.52 8.52 8.12 8.12 8.12 8.11 8.10 8.10 8.09 7.72 7.71 7.70 7.70 7.69 7.68 7.67 7.63 7.63 7.61 7.61 7.59 7.59
7.26 CDCl3
5.20
2.31
2.13
1H NMR, 400 MHz, CDCl3
OH
O
Me
Me
Me
OH
C-31
0.93
1.00
1.07
1.03
0.99
2.01
1.01
0.91
2.97
5.98
14.5 14.0 13.5 13.0 12.5 12.0 11.5 11.0 10.5 10.0 9.5 9.0 8.5 8.0 7.5 7.0 6.5 6.0 5.5 5.0 4.5 4.0 3.5 3.0 2.5 2.0 1.5 1.0 0.5 0
f1 (ppm)

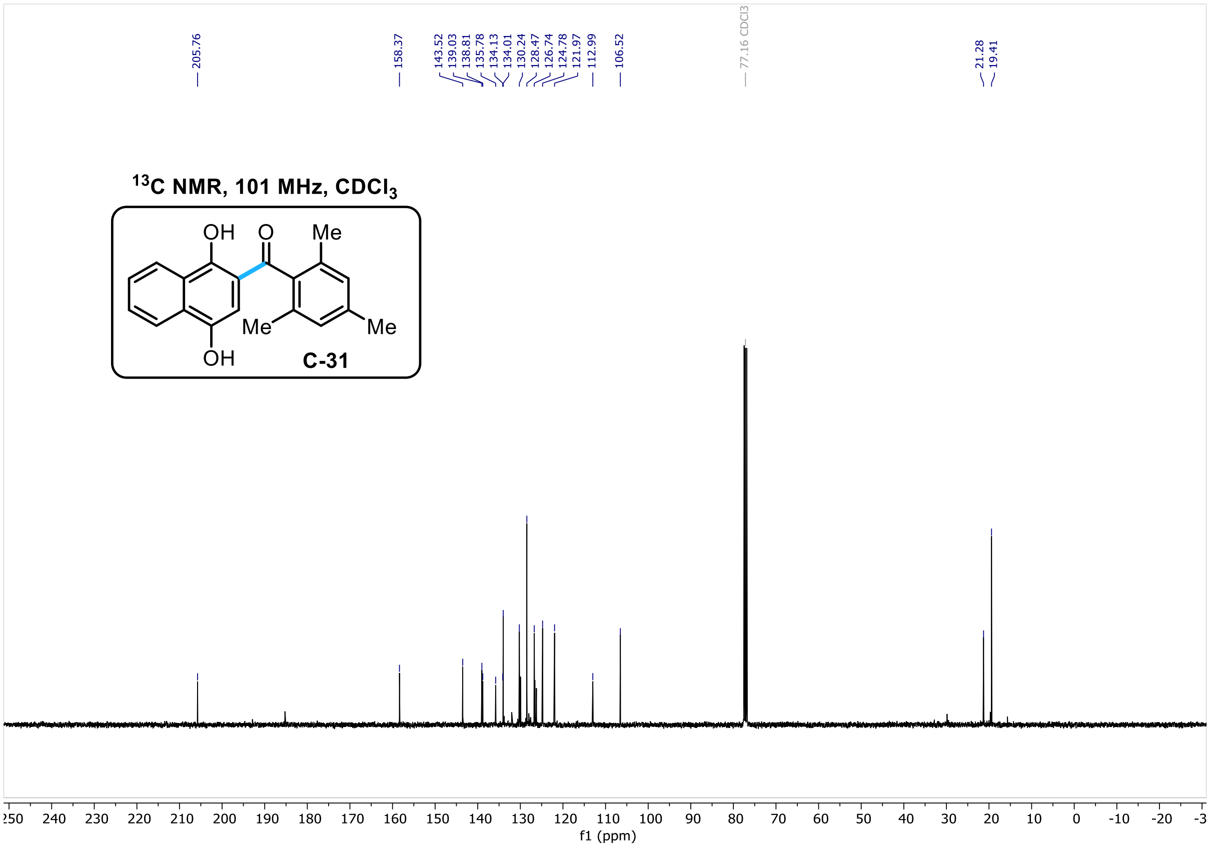
205.76
158.37
143.52 139.03 138.81 135.78 134.13 134.01 130.24 128.47 126.74 124.78 121.97 112.99
106.52
77.16 CDCl3
21.28
19.41
13C NMR, 101 MHz, CDCl3
OH
O
Me
Me
Me
OH
C-31
250 240 230 220 210 200 190 180 170 160 150 140 130 120 110 100 90 80 70 60 50 40 30 20 10 0 -10 -20 -3
f1 (ppm)

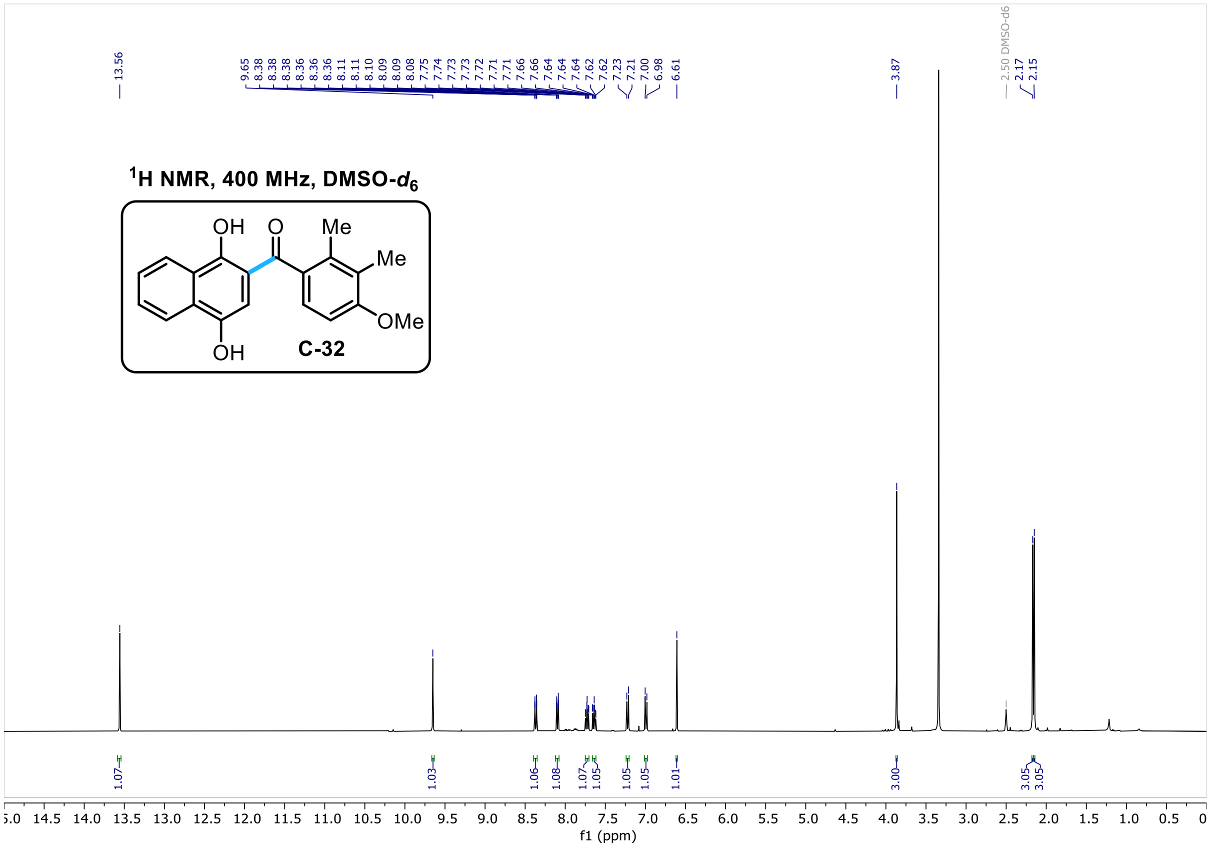
13.56
9.65
8.38
8.38
8.38
8.36
8.36
8.36
8.11
8.11
8.10
8.09
8.09
8.08
7.75
7.74
7.73
7.73
7.72
7.71
7.71
7.66
7.66
7.64
7.64
7.62
7.62
7.23
7.21
7.00
6.98
6.61
3.87
2.50 DMSO-d6
2.17
2.15
1H NMR, 400 MHz, DMSO-d6
OH
O
Me
Me
OMe
OH
C-32
1.07
1.03
1.06
1.08
1.07
1.05
1.05
1.05
1.01
3.00
3.05
3.05
f1 (ppm)

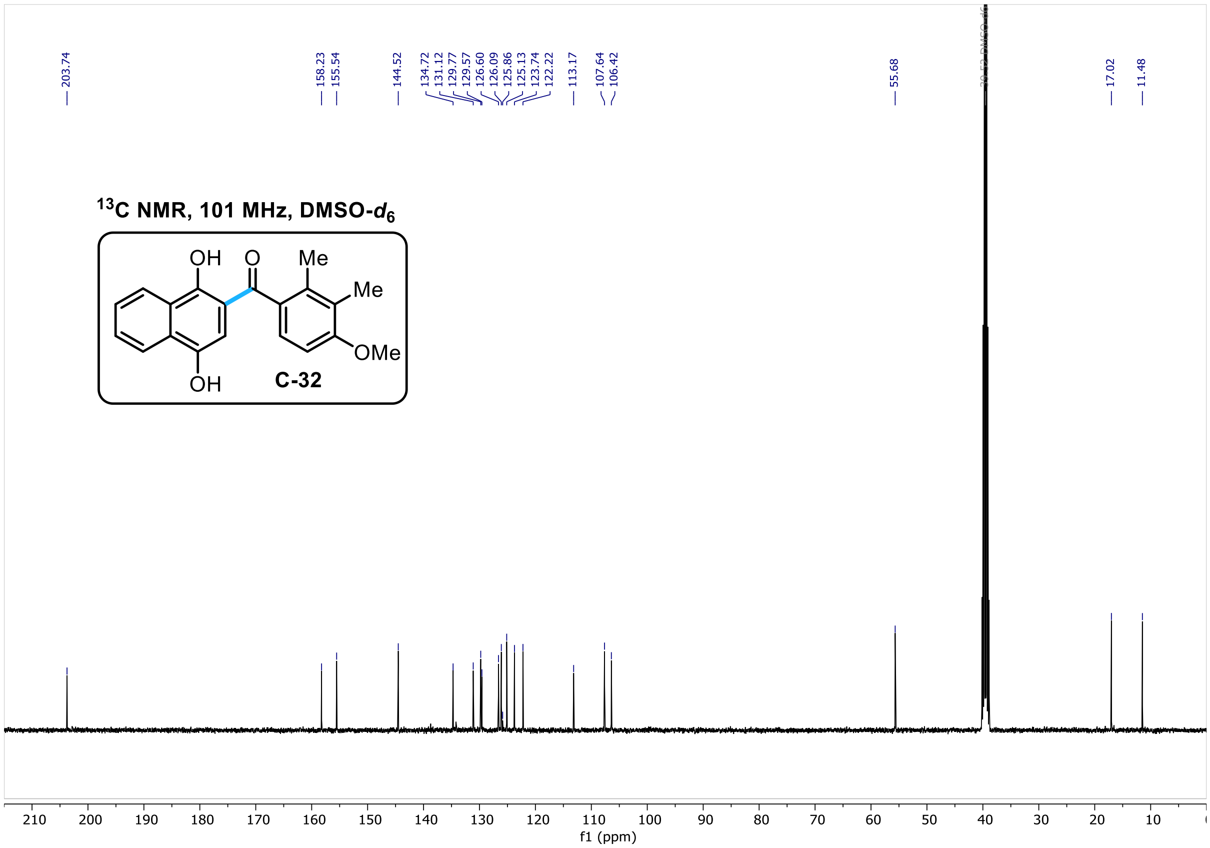
203.74
158.23
155.54
144.52
134.72
131.12
129.77
129.57
126.60
126.09
125.86
125.13
123.74
122.22
113.17
107.64
106.42
55.68
17.02
11.48
13C NMR, 101 MHz, DMSO-d6
OH
O
Me
Me
OMe
OH
C-32
f1 (ppm)

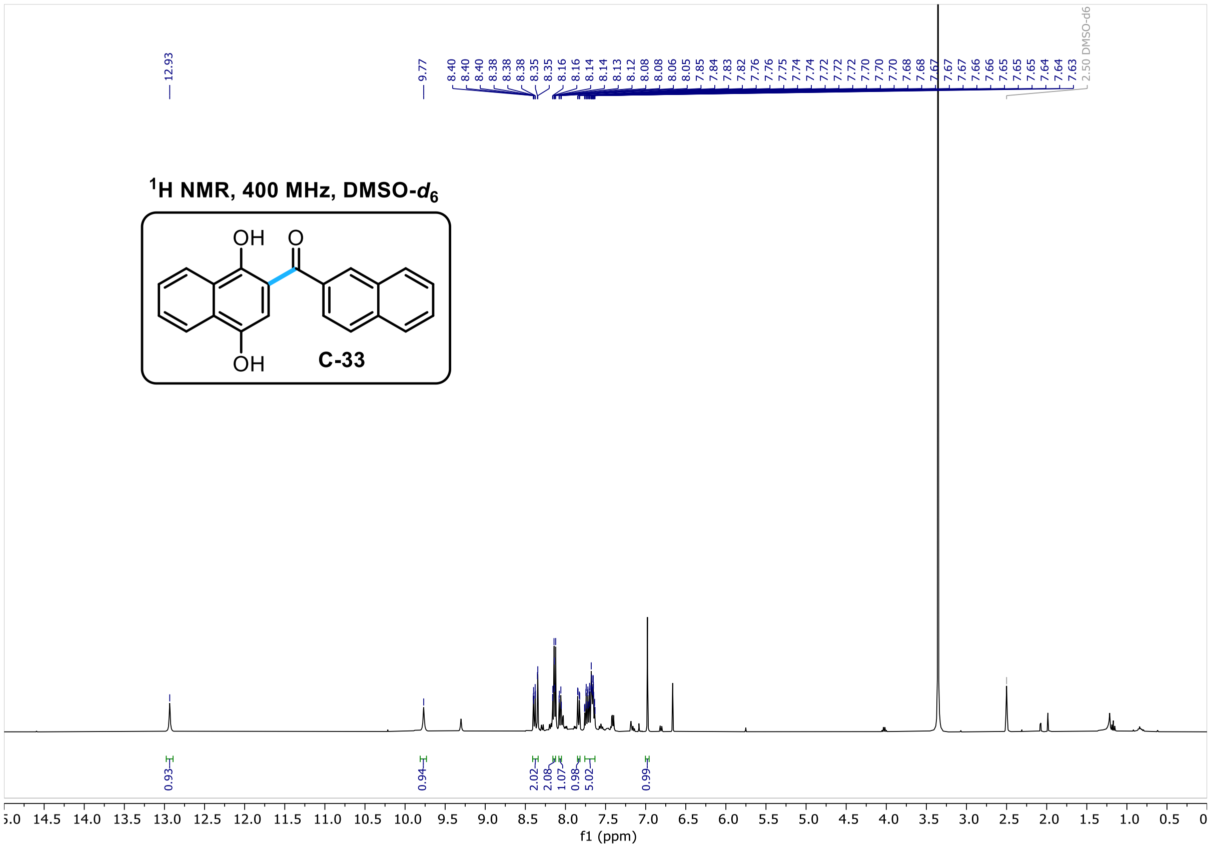

1H NMR, 400 MHz, DMSO-d6
OH
O
OH
C-33
f1 (ppm)

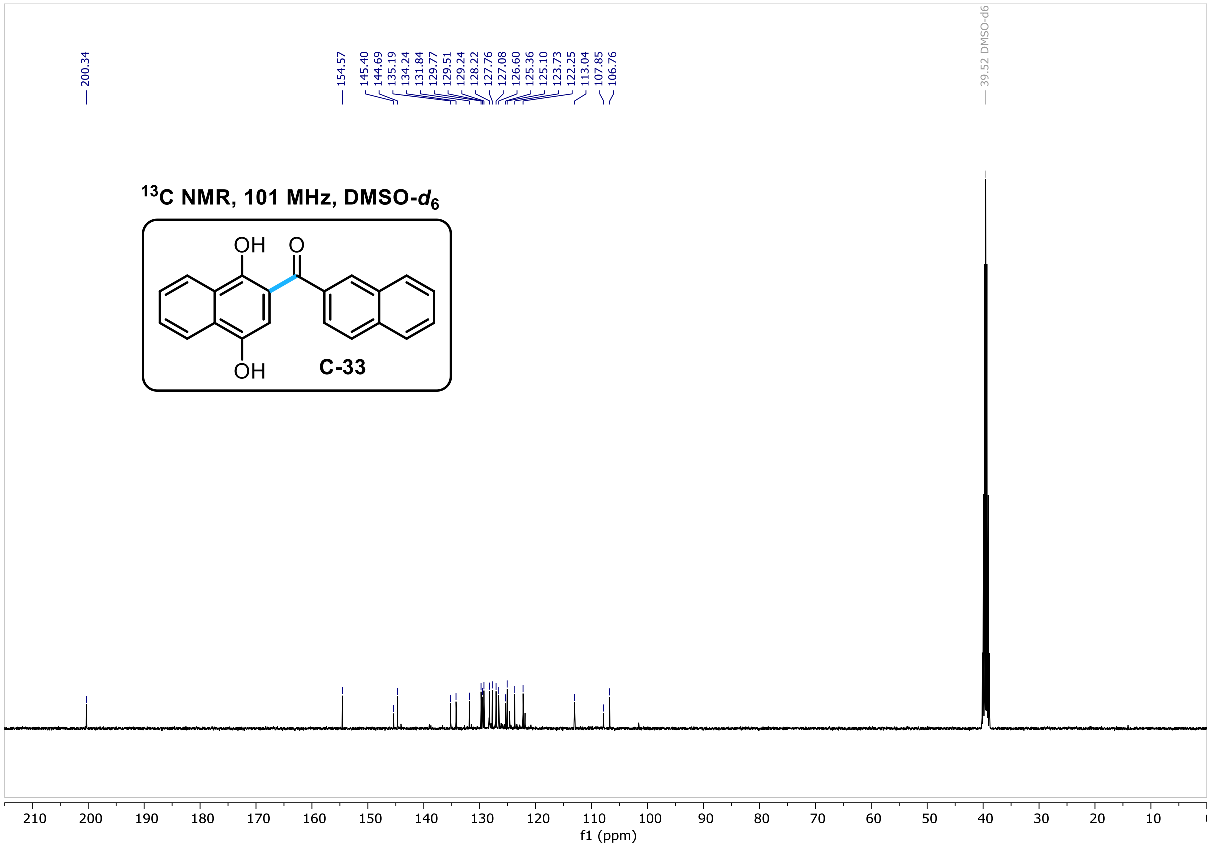

13C NMR, 101 MHz, DMSO-d6
OH
O
OH
C-33
f1 (ppm)

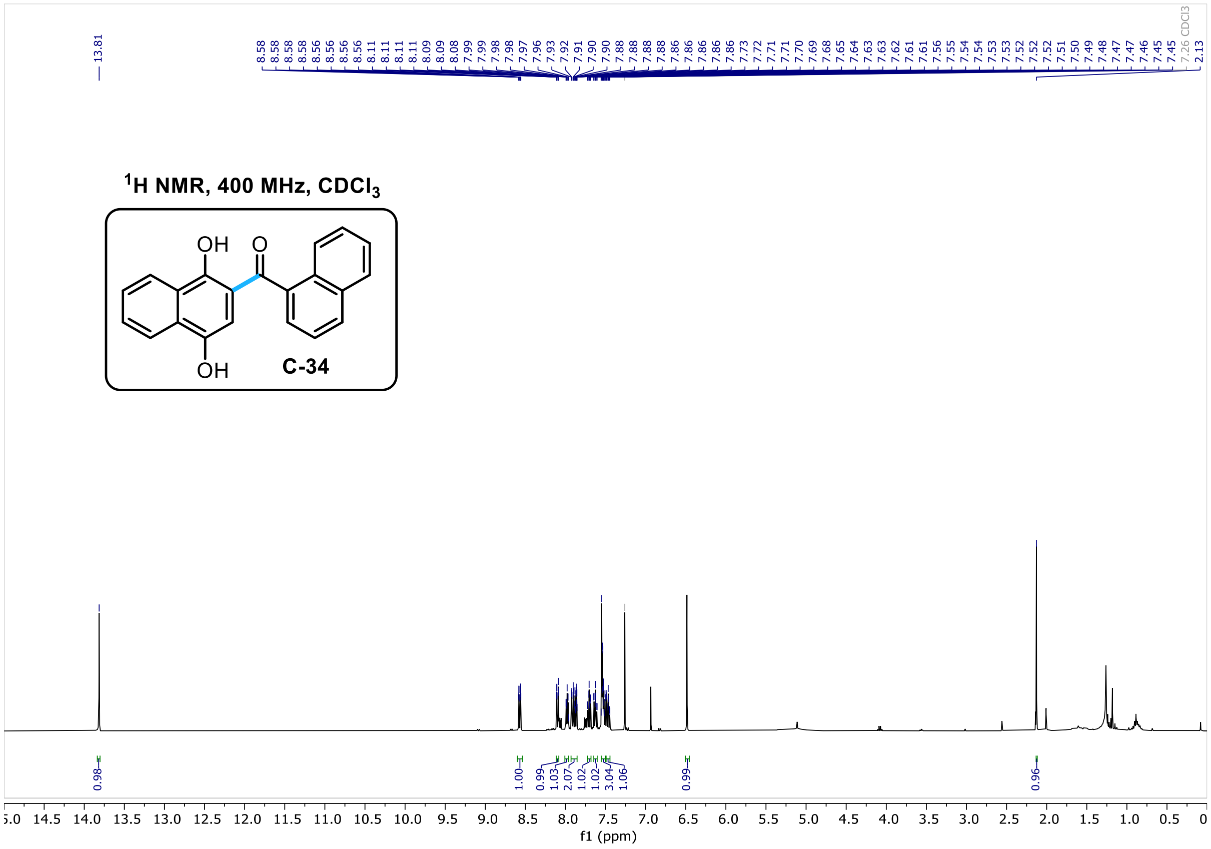
1H NMR, 400 MHz, CDCl3
C-34
f1 (ppm)

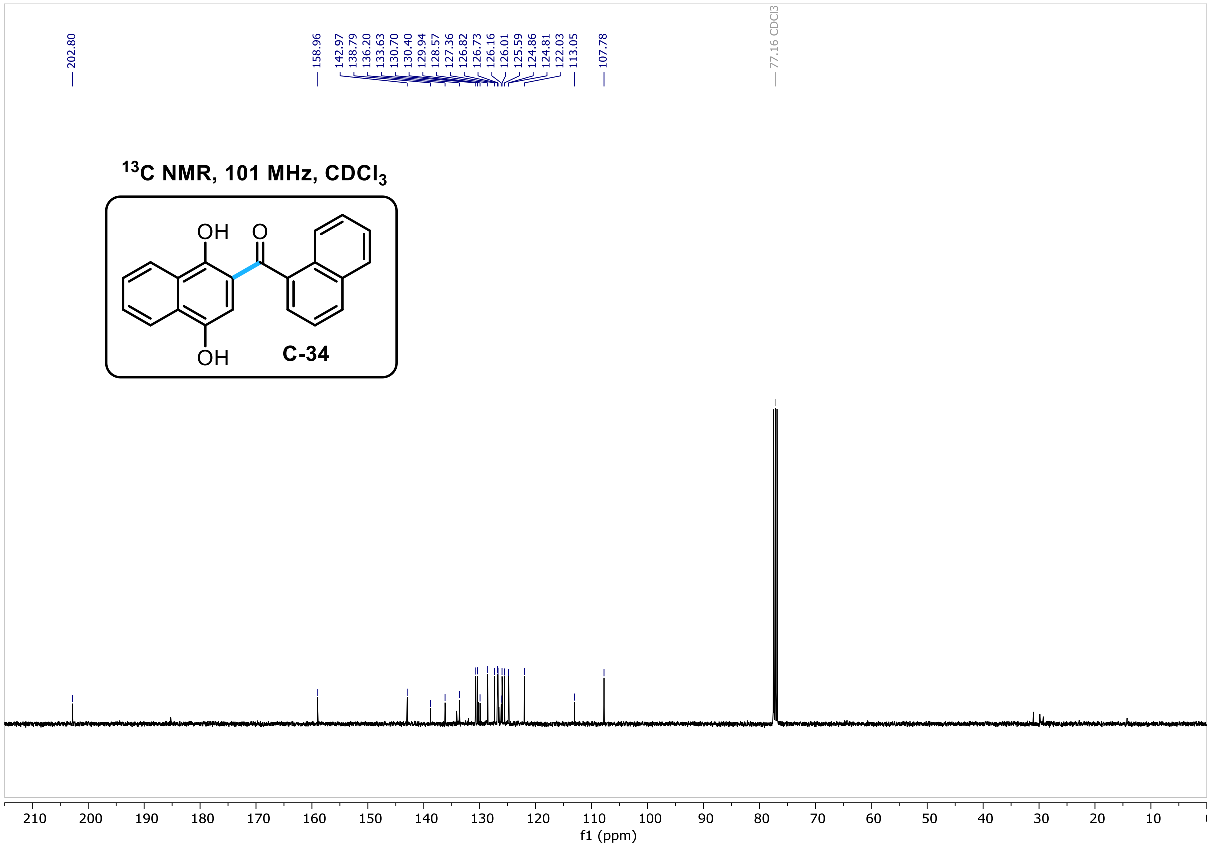
13C NMR, 101 MHz, CDCl3
C-34
f1 (ppm)

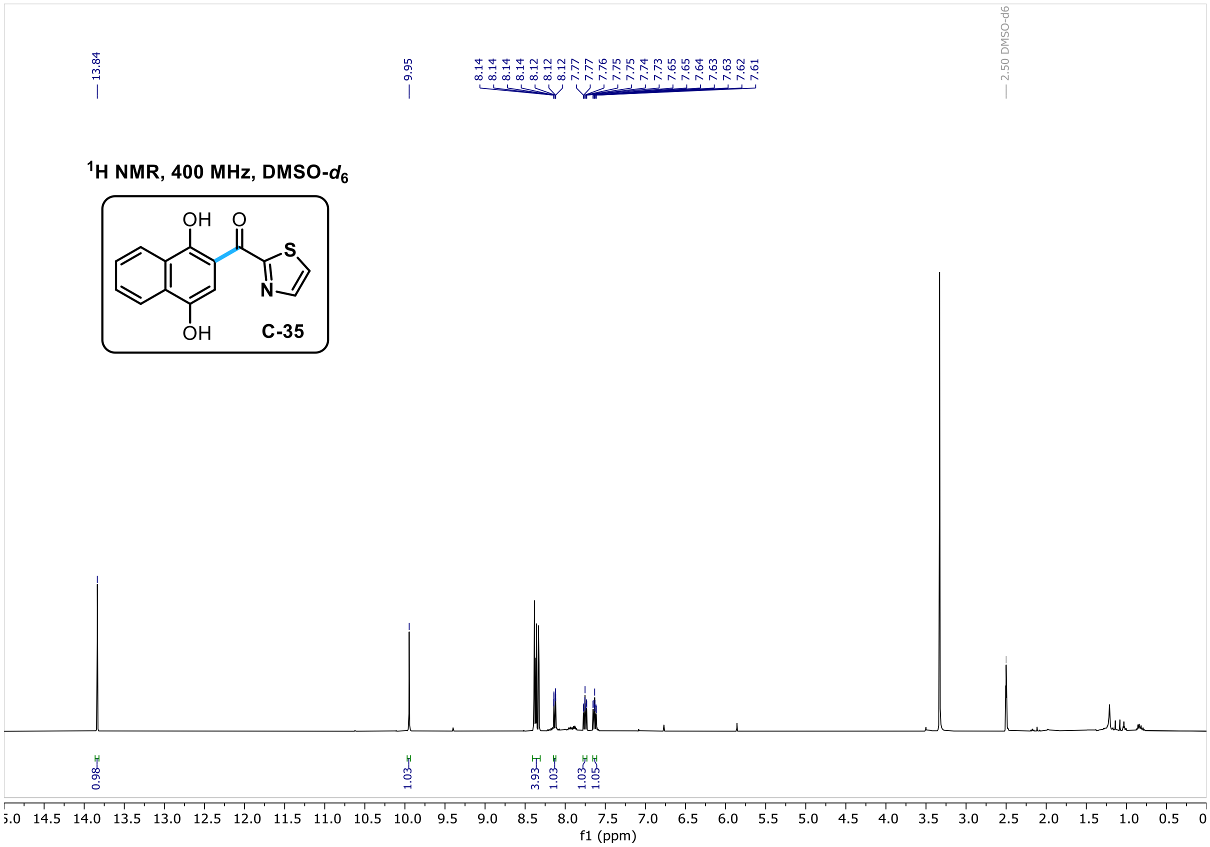
13.84
9.95
8.14
8.14
8.14
8.14
8.12
8.12
8.12
7.77
7.77
7.76
7.75
7.75
7.74
7.73
7.65
7.65
7.64
7.63
7.63
7.62
7.61
2.50 DMSO-d6
1H NMR, 400 MHz, DMSO-d6
OH
O
S
N
OH
C-35
0.98
1.03
3.93
1.03
1.03
1.05
f1 (ppm)

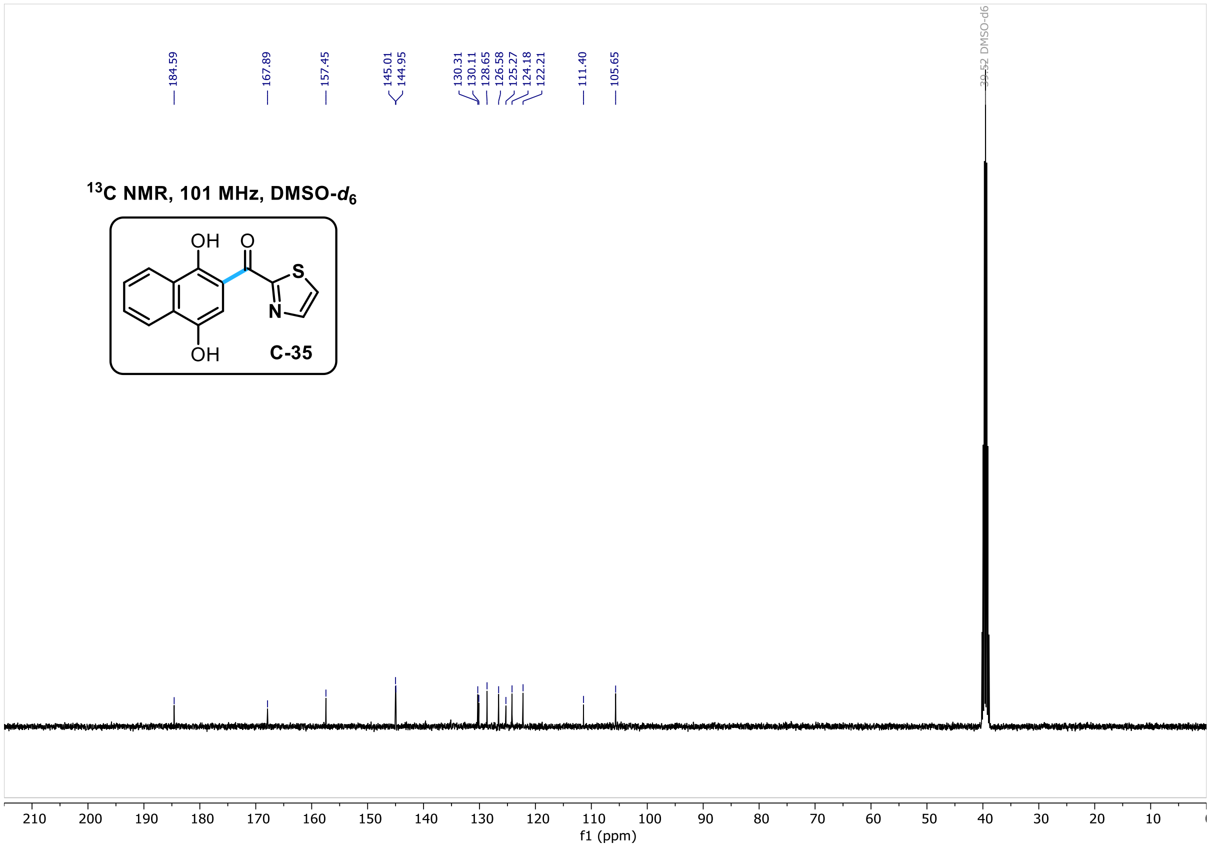
184.59
167.89
157.45
145.01
144.95
130.31
130.11
128.65
126.58
125.27
124.18
122.21
111.40
105.65
39.52 DMSO-d6
13C NMR, 101 MHz, DMSO-d6
OH
O
S
N
OH
C-35
f1 (ppm)

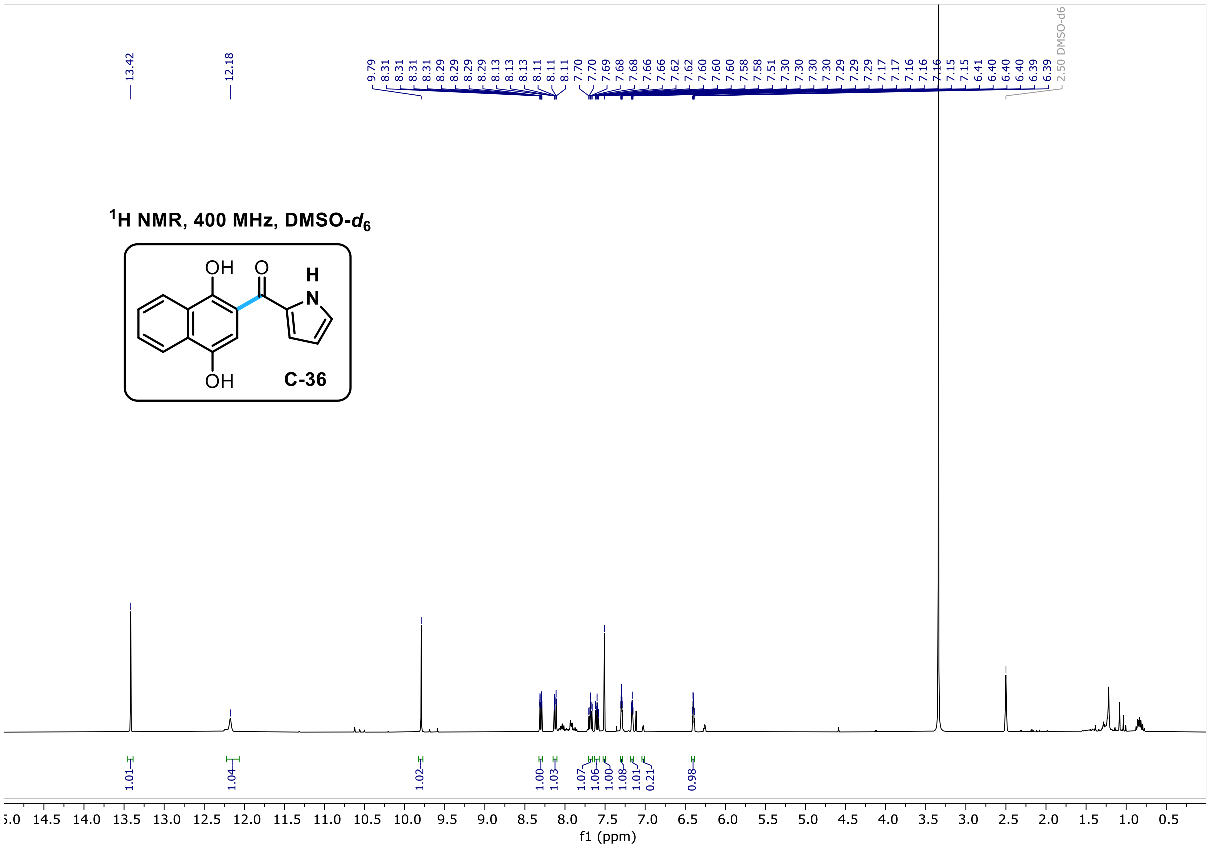
1H NMR, 400 MHz, DMSO-d6
OH O H N
OH C-36
13.42
12.18
9.79 8.31 8.31 8.31 8.31 8.29 8.29 8.29 8.29 8.13 8.13 8.13 8.11 8.11 8.11 7.70 7.70 7.69 7.68 7.68 7.66 7.66 7.62 7.62 7.60 7.60 7.60 7.58 7.58 7.51 7.30 7.30 7.30 7.30 7.29 7.29 7.29 7.17 7.17 7.16 7.16 7.15 7.15 6.41 6.40 6.40 6.39 6.39
2.50 DMSO-d6
1.01 1.04 1.02 1.00 1.03 1.07 1.06 1.00 1.08 1.01 0.21 0.98
f1 (ppm)

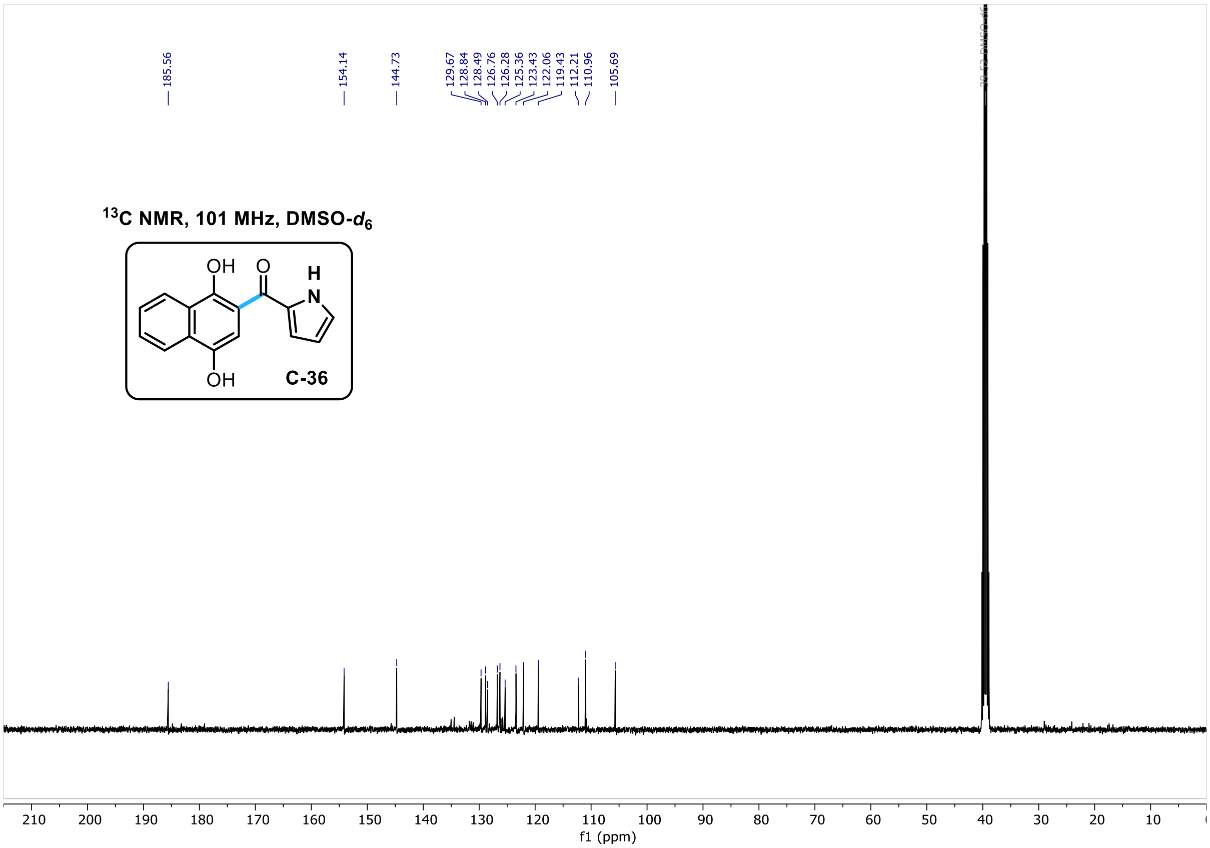
13C NMR, 101 MHz, DMSO-d6
OH O H N
OH C-36
185.56
154.14
144.73
129.67 128.84 128.49 126.76 126.28 125.36 123.43 122.06 119.43 112.21 110.96
105.69
f1 (ppm)

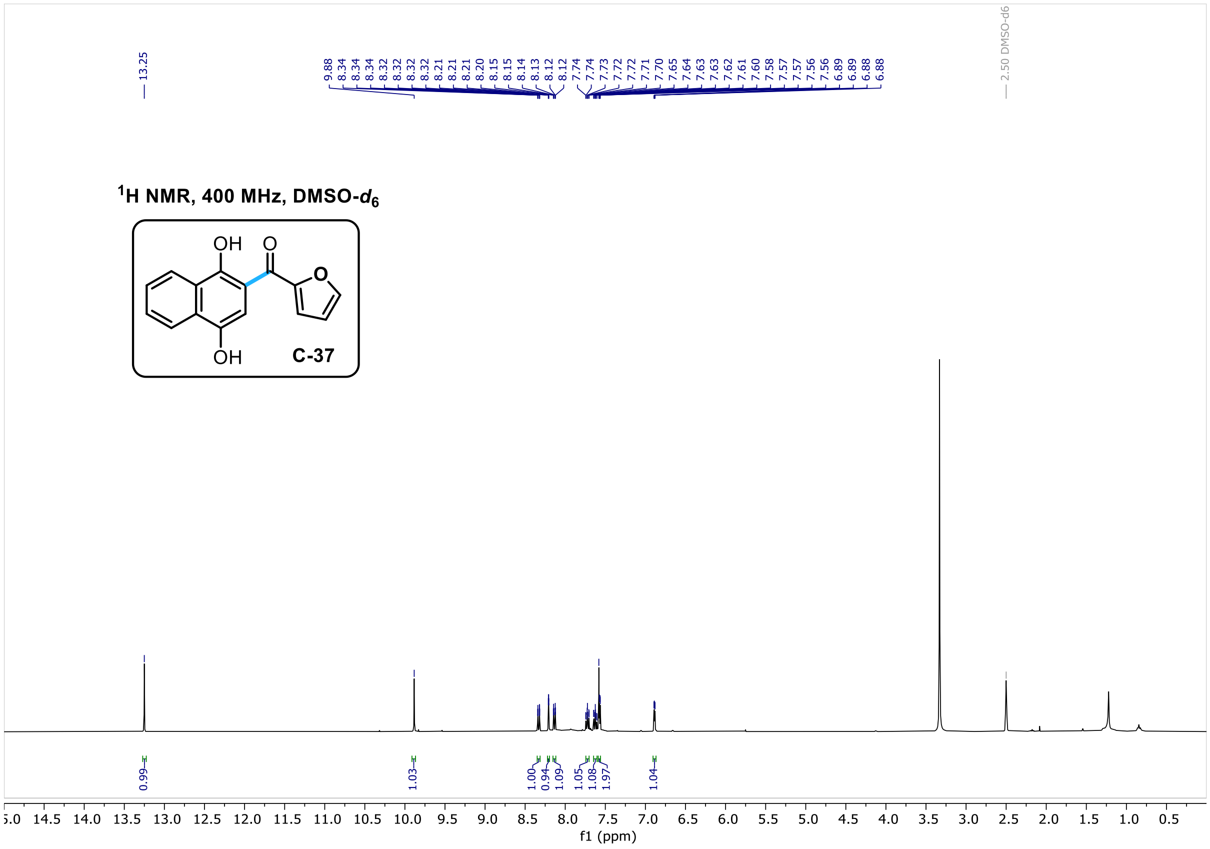
13.25
9.88 8.34 8.34 8.34 8.32 8.32 8.32 8.21 8.21 8.21 8.20 8.15 8.15 8.14 8.13 8.12 8.12 7.74 7.74 7.73 7.72 7.72 7.71 7.70 7.65 7.64 7.63 7.63 7.62 7.61 7.60 7.58 7.57 7.57 7.56 7.56 6.89 6.89 6.88 6.88
2.50 DMSO-d6
^{1}H NMR, 400 MHz, DMSO-d_6
OH
O
OH
C-37
0.99
1.03
1.00
0.94
1.09
1.05
1.08
1.97
1.04
f1 (ppm)

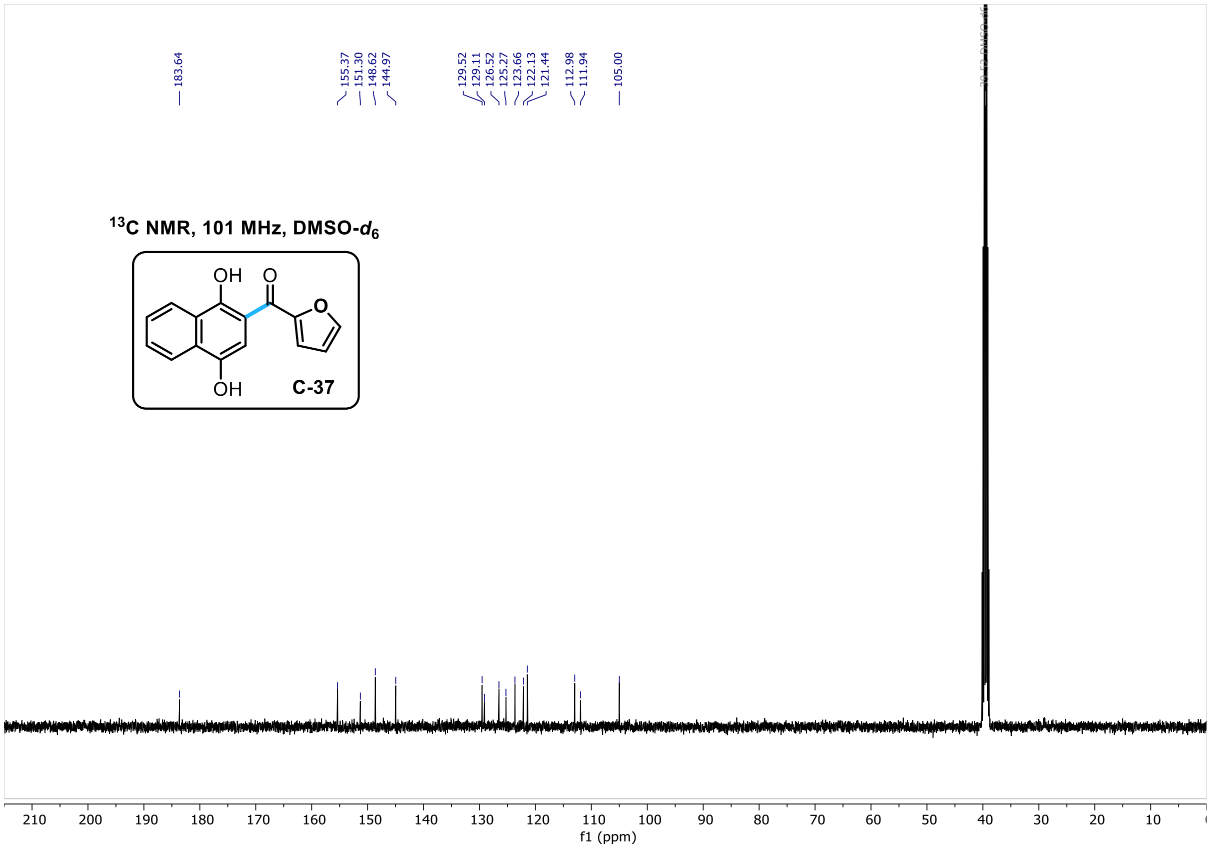
183.64
155.37 151.30 148.62 144.97
129.52 129.11 126.52 125.27 123.66 122.13 121.44
112.98 111.94
105.00
^{13}C NMR, 101 MHz, DMSO-d_6
OH
O
OH
C-37
f1 (ppm)

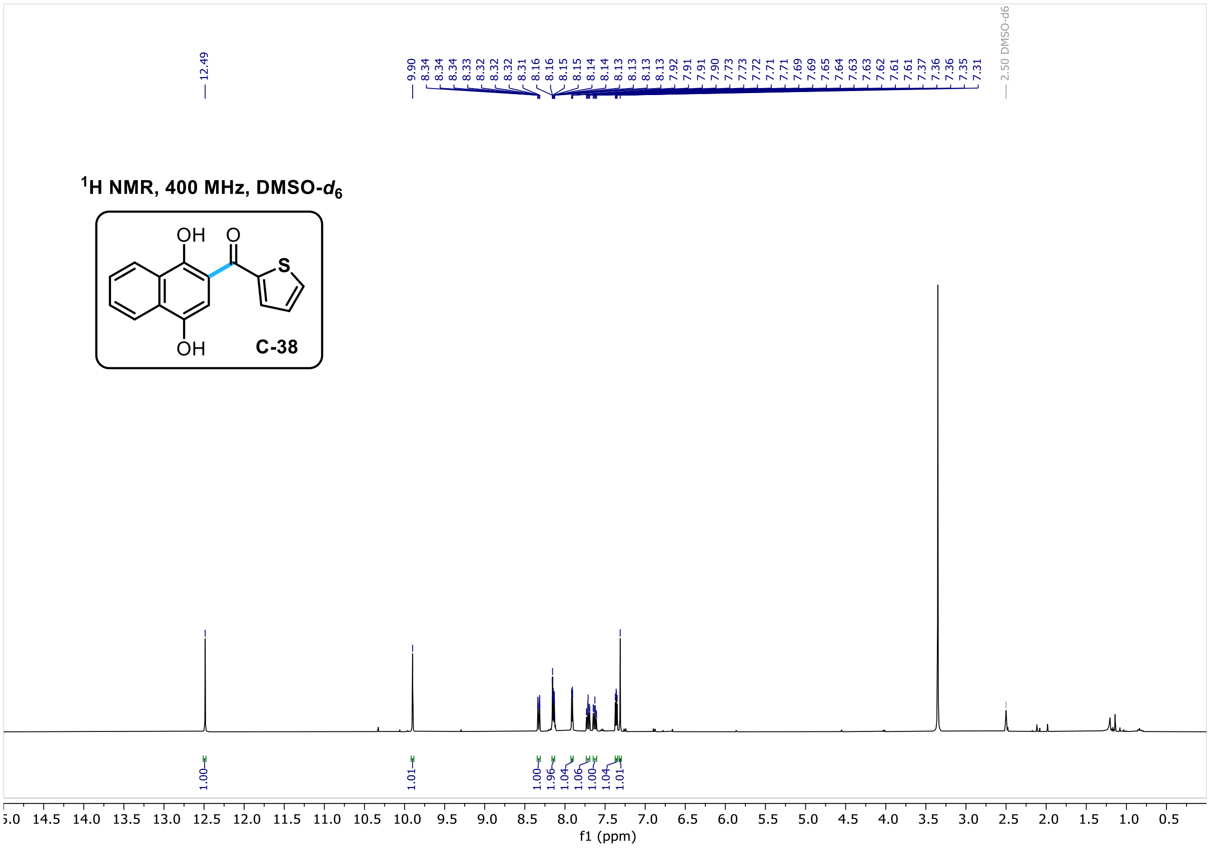
1H NMR, 400 MHz, DMSO-d6
OH O
S
OH
C-38
12.49
9.90 8.34 8.34 8.34 8.33 8.32 8.32 8.32 8.31 8.16 8.16 8.15 8.15 8.14 8.14 8.13 8.13 8.13 8.13 7.92 7.91 7.91 7.90 7.73 7.73 7.72 7.71 7.71 7.69 7.69 7.65 7.64 7.63 7.63 7.62 7.61 7.61 7.37 7.36 7.35 7.31
2.50 DMSO-d6
1.00 1.01 1.00 1.96 1.04 1.06 1.00 1.04 1.01
f1 (ppm)

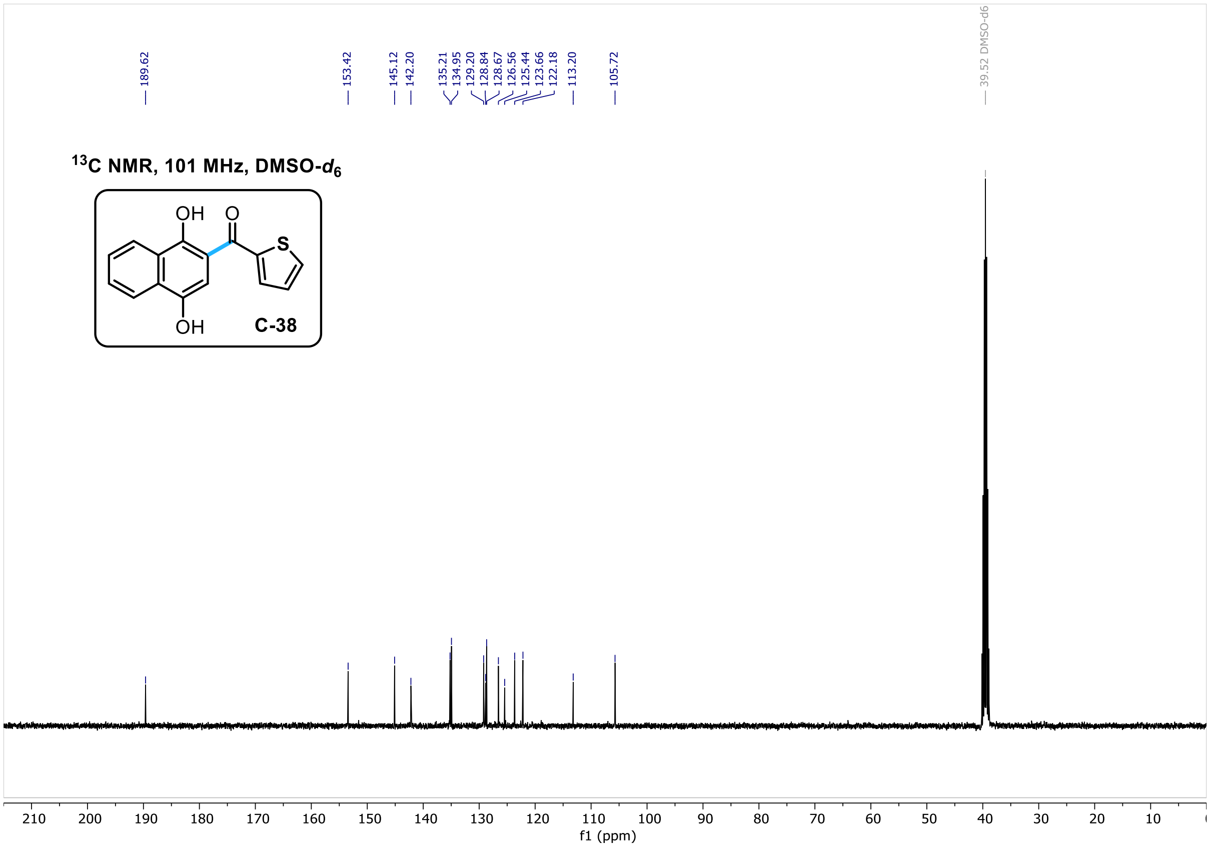
13C NMR, 101 MHz, DMSO-d6
OH O
S
OH
C-38
189.62
153.42
145.12 142.20
135.21 134.95 129.20 128.84 128.67 126.56 125.44 123.66 122.18
113.20
105.72
39.52 DMSO-d6
f1 (ppm)

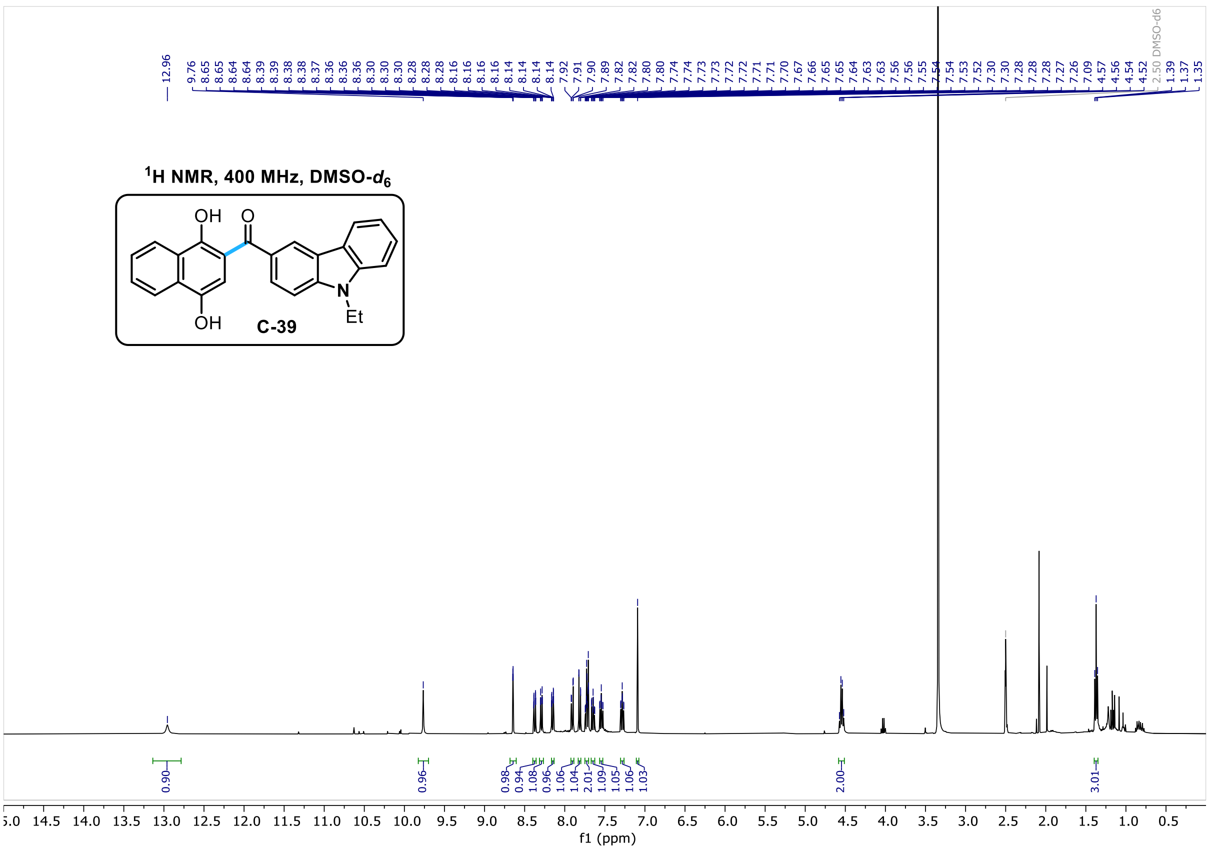
1H NMR, 400 MHz, DMSO-d6
OH
O
OH
N
Et
C-39
f1 (ppm)

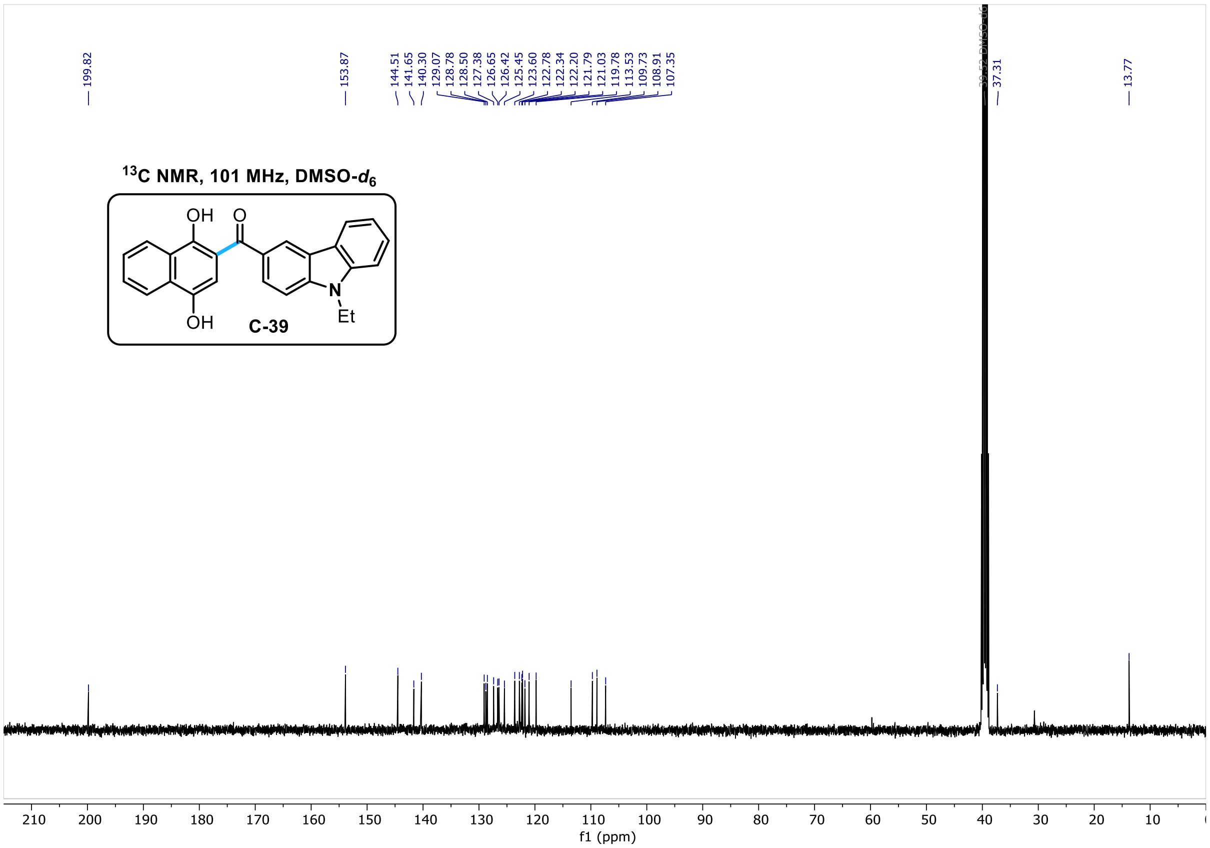
13C NMR, 101 MHz, DMSO-d6
OH
O
OH
N
Et
C-39
199.82
153.87
144.51
141.65
140.30
37.31
13.77
f1 (ppm)

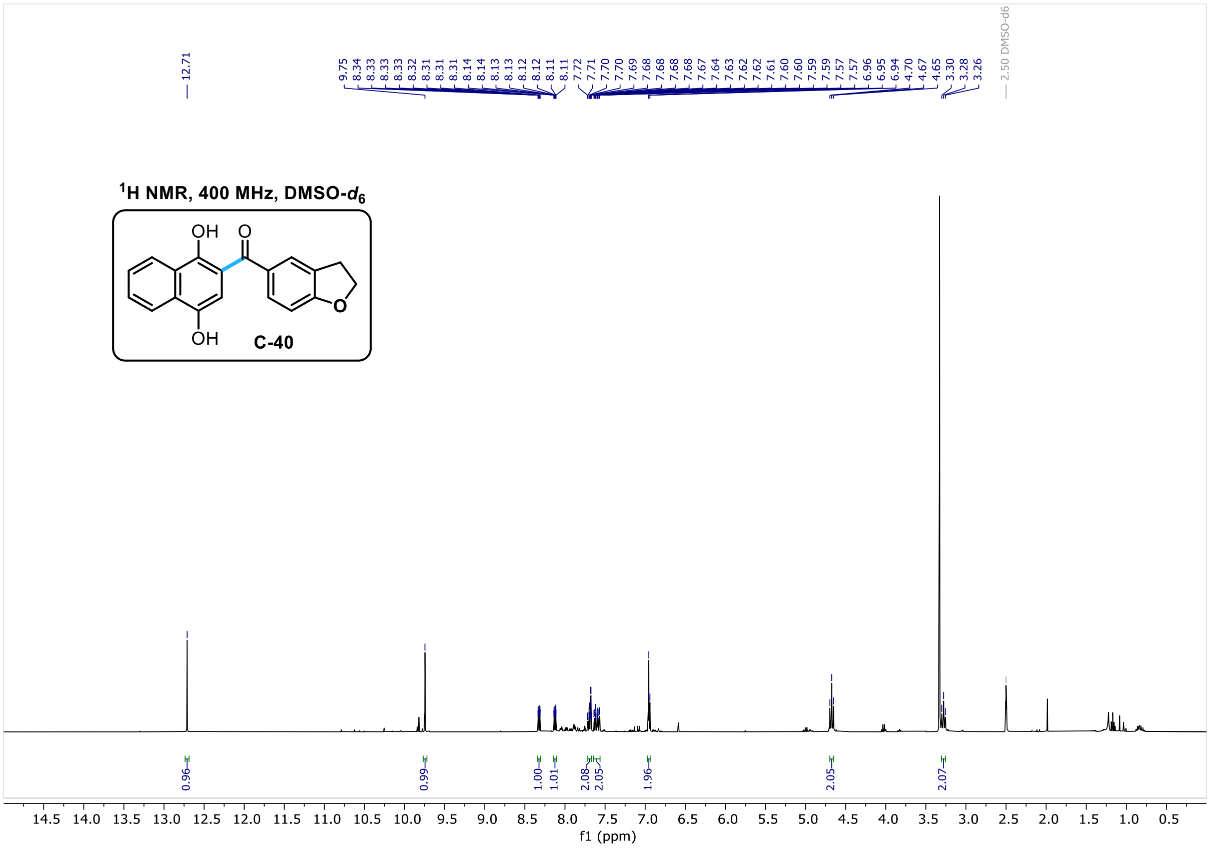
1H NMR, 400 MHz, DMSO-d6
C-40
12.71
9.75
8.34
8.33
8.33
8.33
8.32
8.31
8.31
8.31
8.31
8.14
8.14
8.13
8.13
8.12
8.12
8.11
8.11
7.72
7.71
7.70
7.70
7.69
7.68
7.68
7.68
7.67
7.64
7.63
7.62
7.62
7.61
7.60
7.60
7.59
7.59
7.57
7.57
6.96
6.95
6.94
4.70
4.67
4.65
3.30
3.28
3.26
2.50 DMSO-d6
0.96
0.99
1.00
1.01
2.08
2.05
1.96
2.05
2.07
f1 (ppm)

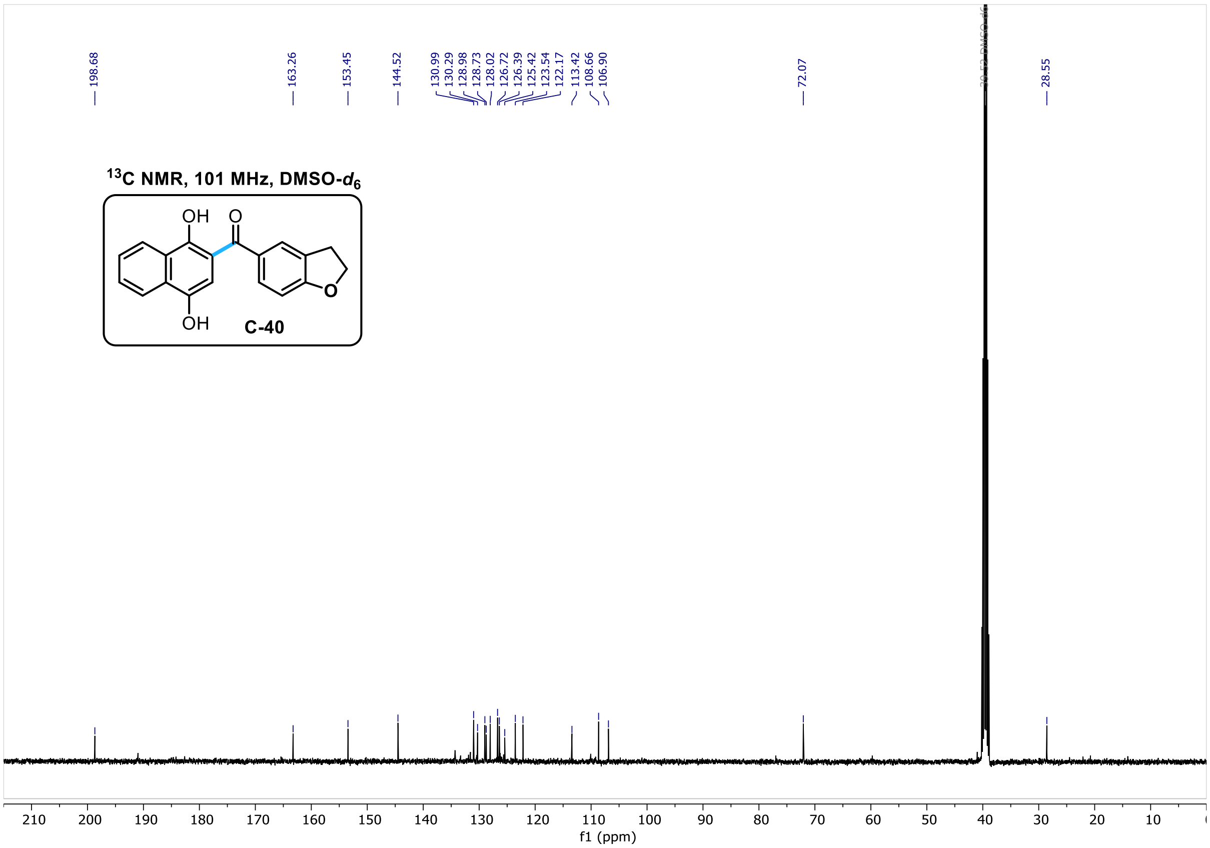
13C NMR, 101 MHz, DMSO-d6
C-40
198.68
163.26
153.45
144.52
130.99
130.29
128.98
128.73
128.02
126.72
126.39
125.42
123.54
122.17
113.42
108.66
106.90
72.07
28.55
f1 (ppm)

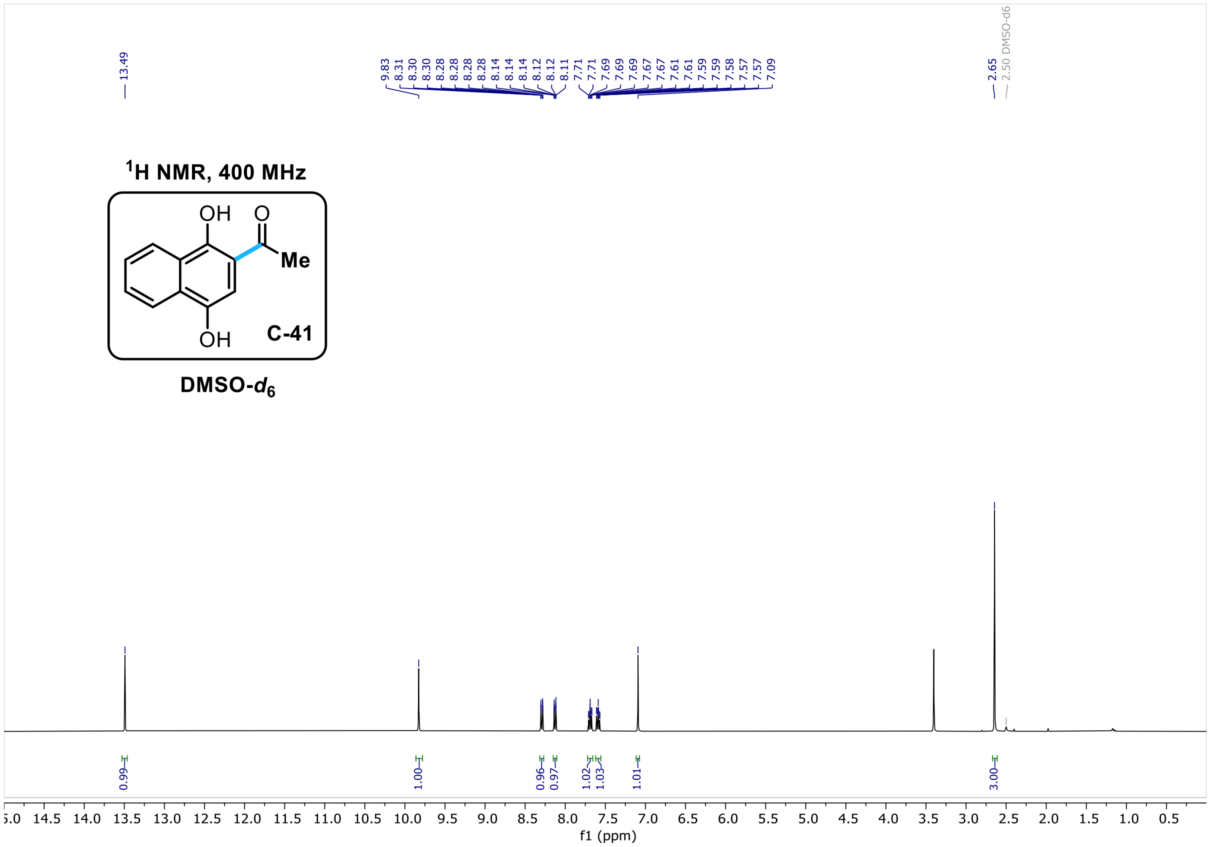

13.49
9.83
8.31
8.30
8.30
8.28
8.28
8.28
8.14
8.14
8.14
8.12
8.12
8.11
7.71
7.71
7.69
7.69
7.69
7.67
7.67
7.61
7.61
7.59
7.58
7.57
7.57
7.09
2.65
2.50 DMSO-d6
1H NMR, 400 MHz
OH
O
Me
OH
C-41
DMSO-d6
0.99
1.00
0.96
0.97
1.02
1.03
1.01
3.00
f1 (ppm)

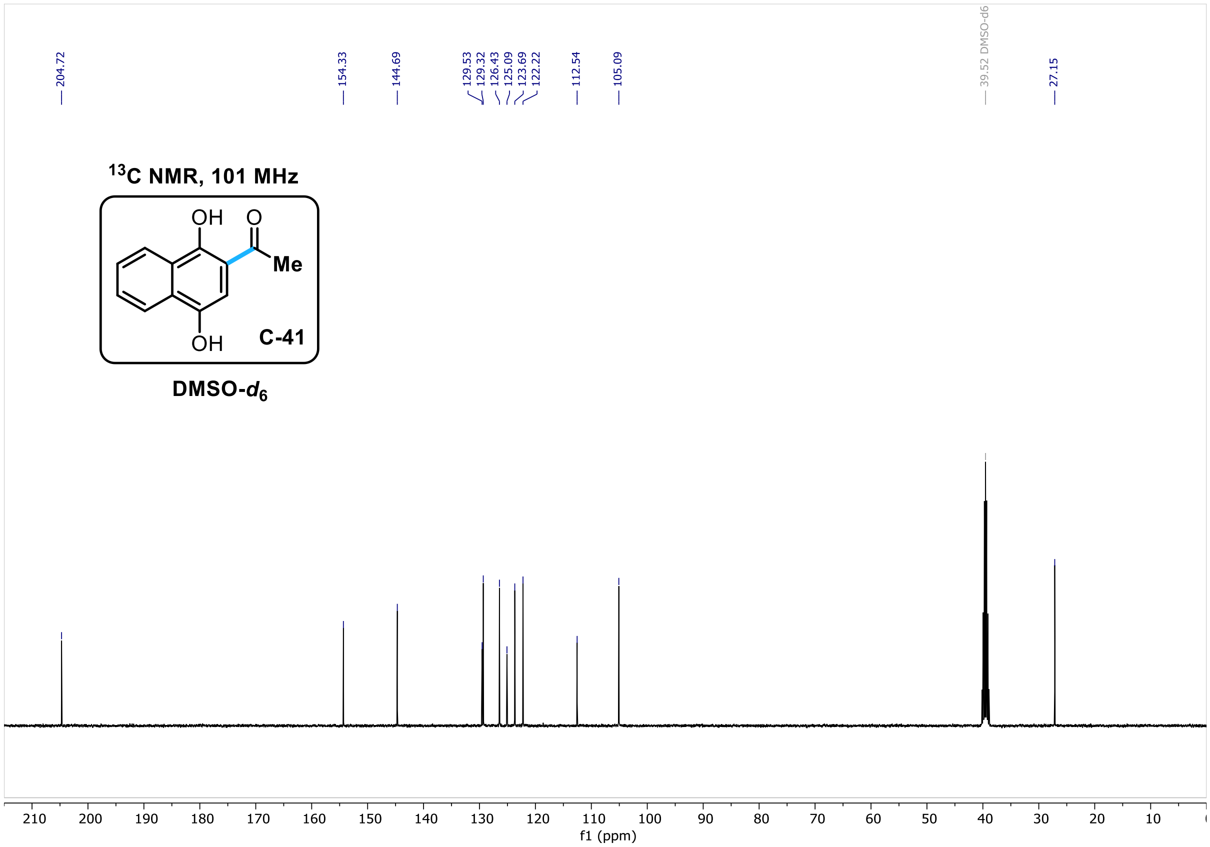

204.72
154.33
144.69
129.53
129.32
126.43
125.09
123.69
122.22
112.54
105.09
39.52 DMSO-d6
27.15
13C NMR, 101 MHz
OH
O
Me
OH
C-41
DMSO-d6
f1 (ppm)

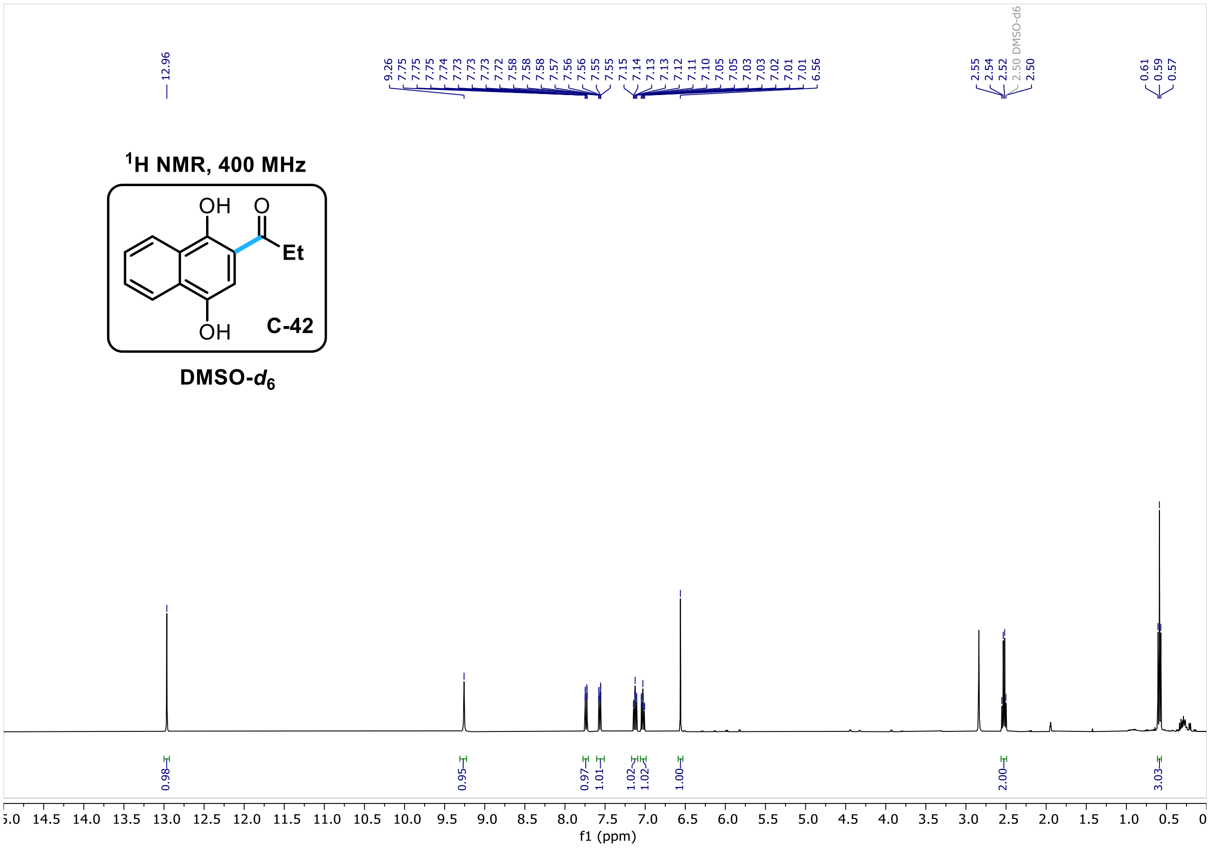
1H NMR, 400 MHz
C-42
DMSO-d6
12.96
9.26 7.75 7.75 7.75 7.74 7.73 7.73 7.72 7.58 7.58 7.58 7.57 7.56 7.56 7.55 7.55 7.15 7.14 7.13 7.13 7.12 7.11 7.10 7.05 7.05 7.03 7.03 7.02 7.01 7.01 6.56
2.55 2.54 2.52 2.50 DMSO-d6 2.50
0.61 0.59 0.57
0.98 0.95 0.97 1.01 1.02 1.02 1.00 2.00 3.03
f1 (ppm)

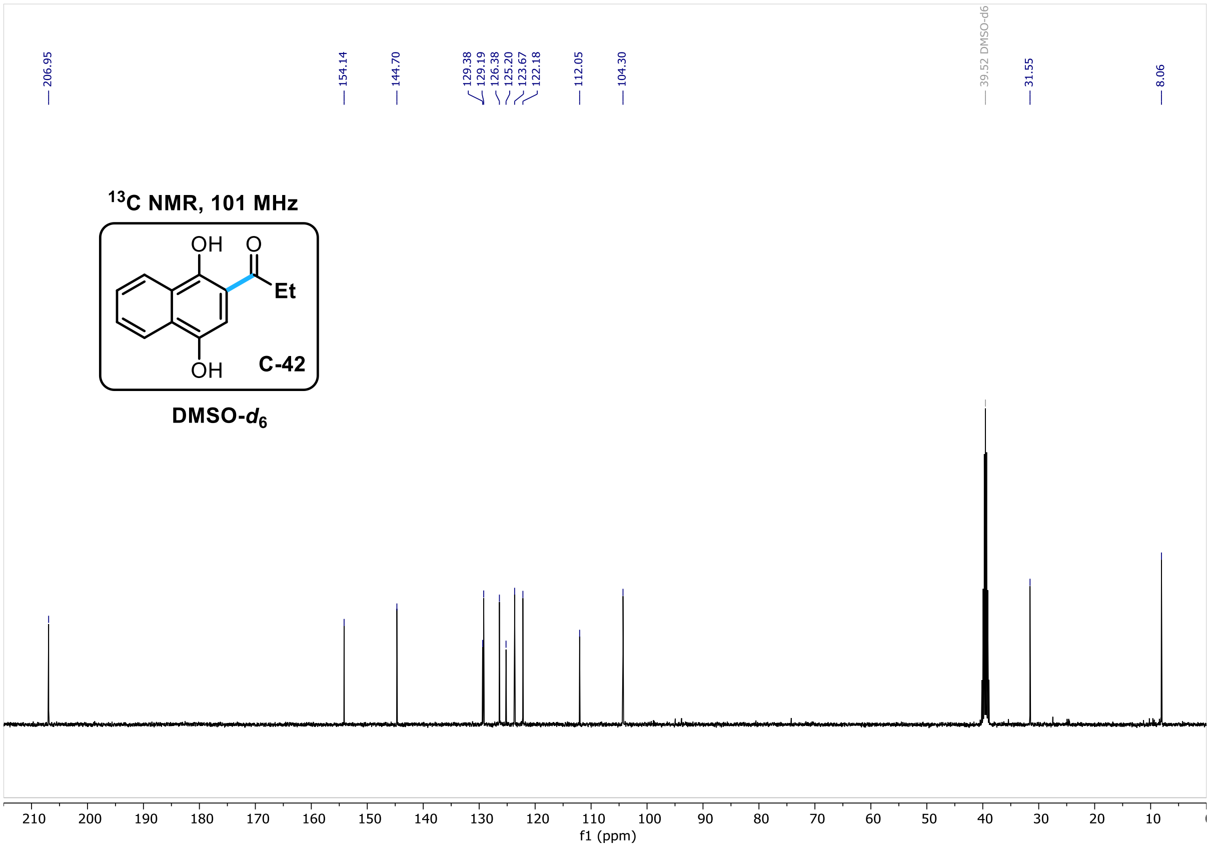
13C NMR, 101 MHz
C-42
DMSO-d6
206.95
154.14
144.70
129.38 129.19 126.38 125.20 123.67 122.18
112.05
104.30
39.52 DMSO-d6
31.55
8.06
f1 (ppm)

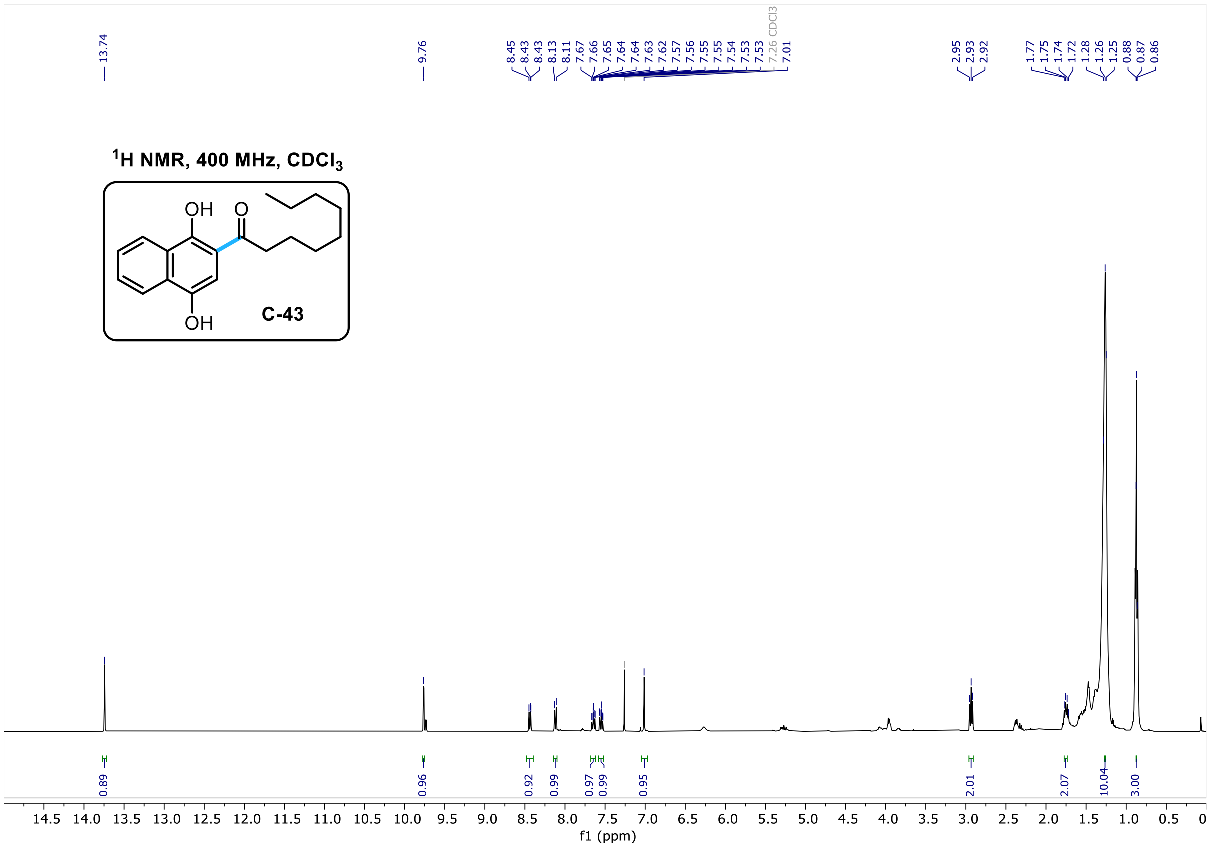
13.74
9.76
8.45 8.43 8.43 8.13 8.11 7.67 7.66 7.65 7.64 7.64 7.63 7.62 7.57 7.56 7.55 7.55 7.54 7.53 7.53
7.26 CDCl3
7.01
2.95 2.93 2.92
1.77 1.75 1.74 1.72 1.28 1.26 1.25 0.88 0.87 0.86
¹H NMR, 400 MHz, CDCl₃
OH
O
OH
C-43
0.89
0.96
0.92
0.99
0.97
0.99
0.95
2.01
2.07
10.04
3.00
14.5 14.0 13.5 13.0 12.5 12.0 11.5 11.0 10.5 10.0 9.5 9.0 8.5 8.0 7.5 7.0 6.5 6.0 5.5 5.0 4.5 4.0 3.5 3.0 2.5 2.0 1.5 1.0 0.5 0
f1 (ppm)

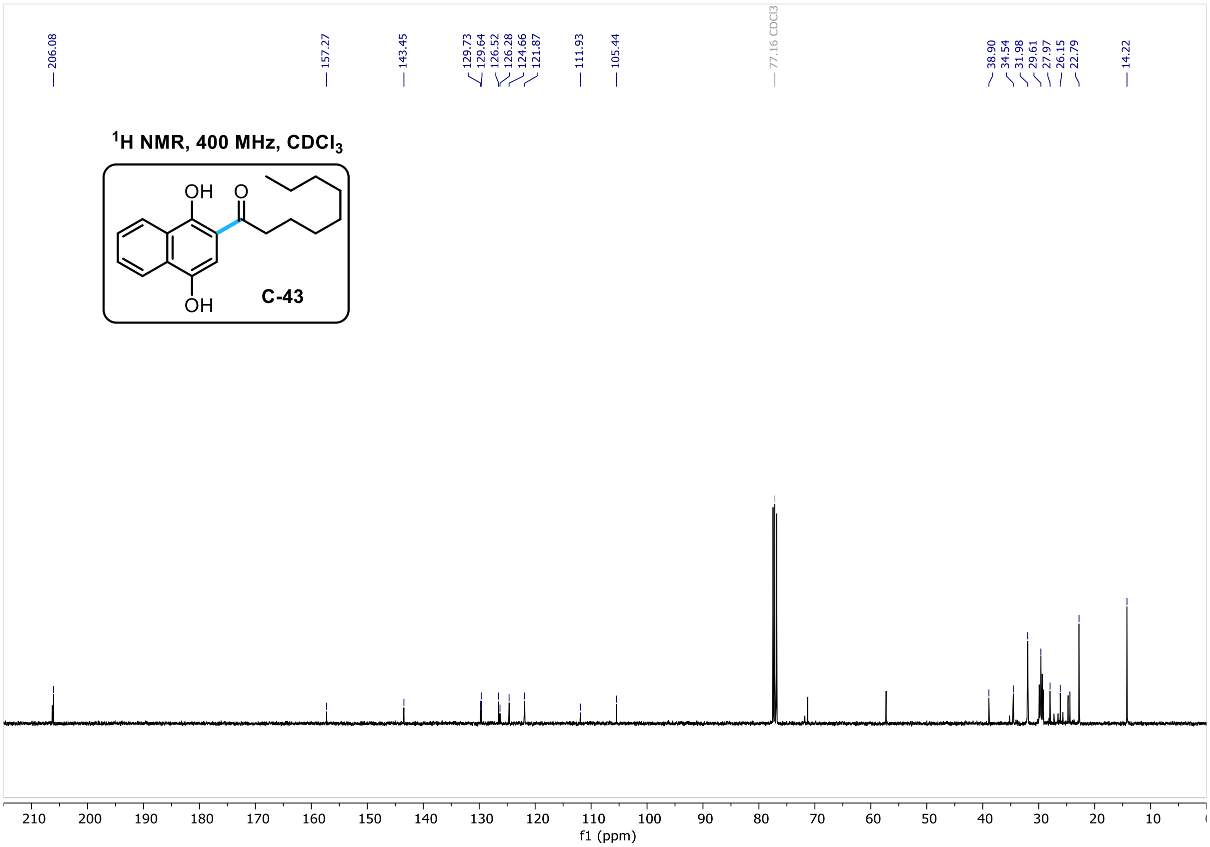
206.08
157.27
143.45
129.73 129.64 126.52 126.28 124.66 121.87
111.93
105.44
77.16 CDCl3
38.90 34.54 31.98 29.61 27.97 26.15 22.79
14.22
¹H NMR, 400 MHz, CDCl₃
OH
O
OH
C-43
210 200 190 180 170 160 150 140 130 120 110 100 90 80 70 60 50 40 30 20 10
f1 (ppm)

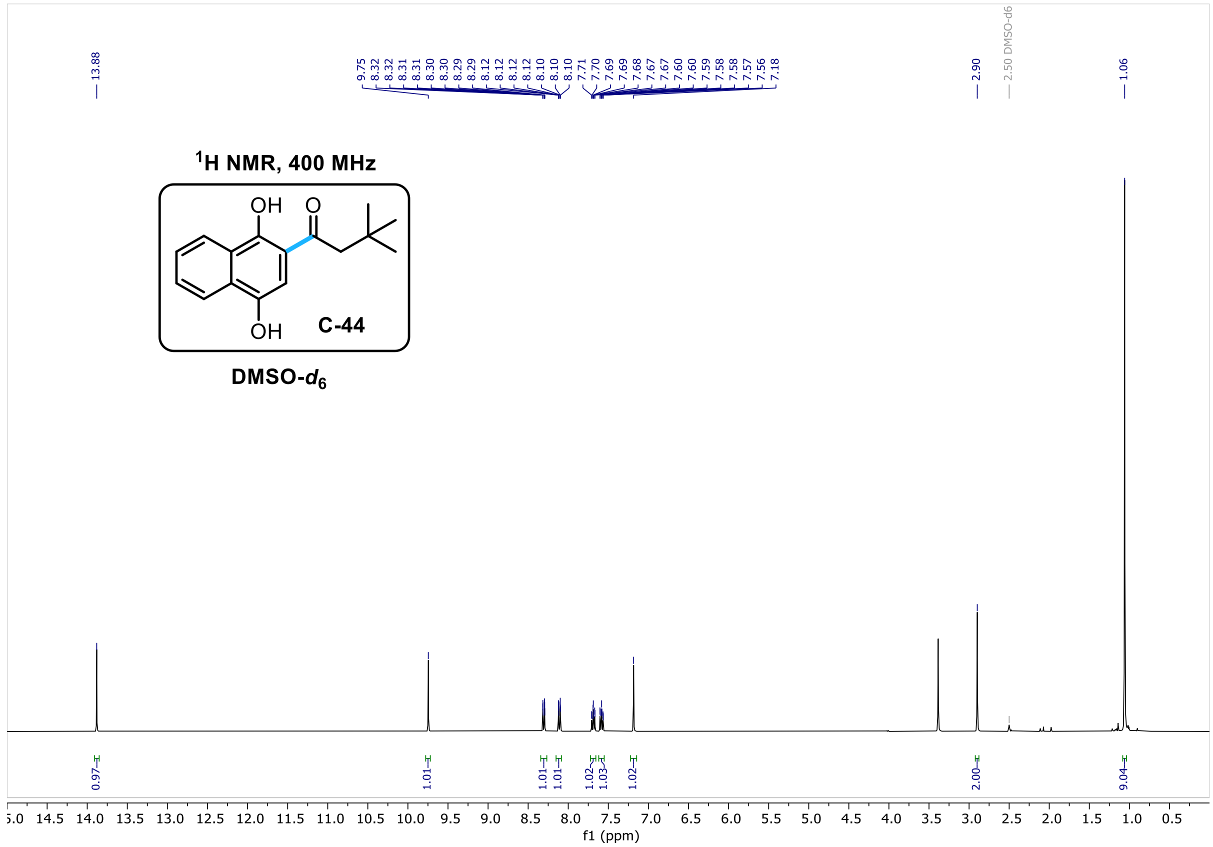
13.88
9.75 8.32 8.32 8.31 8.31 8.30 8.30 8.29 8.29 8.12 8.12 8.12 8.12 8.10 8.10 8.10 7.71 7.70 7.69 7.69 7.68 7.67 7.67 7.60 7.59 7.58 7.58 7.57 7.56 7.18
2.90
2.50 DMSO-d6
1.06
¹H NMR, 400 MHz
OH
O
OH
C-44
DMSO-d6
0.97
1.01
1.01
1.01
1.02
1.03
1.02
2.00
9.04
f1 (ppm)

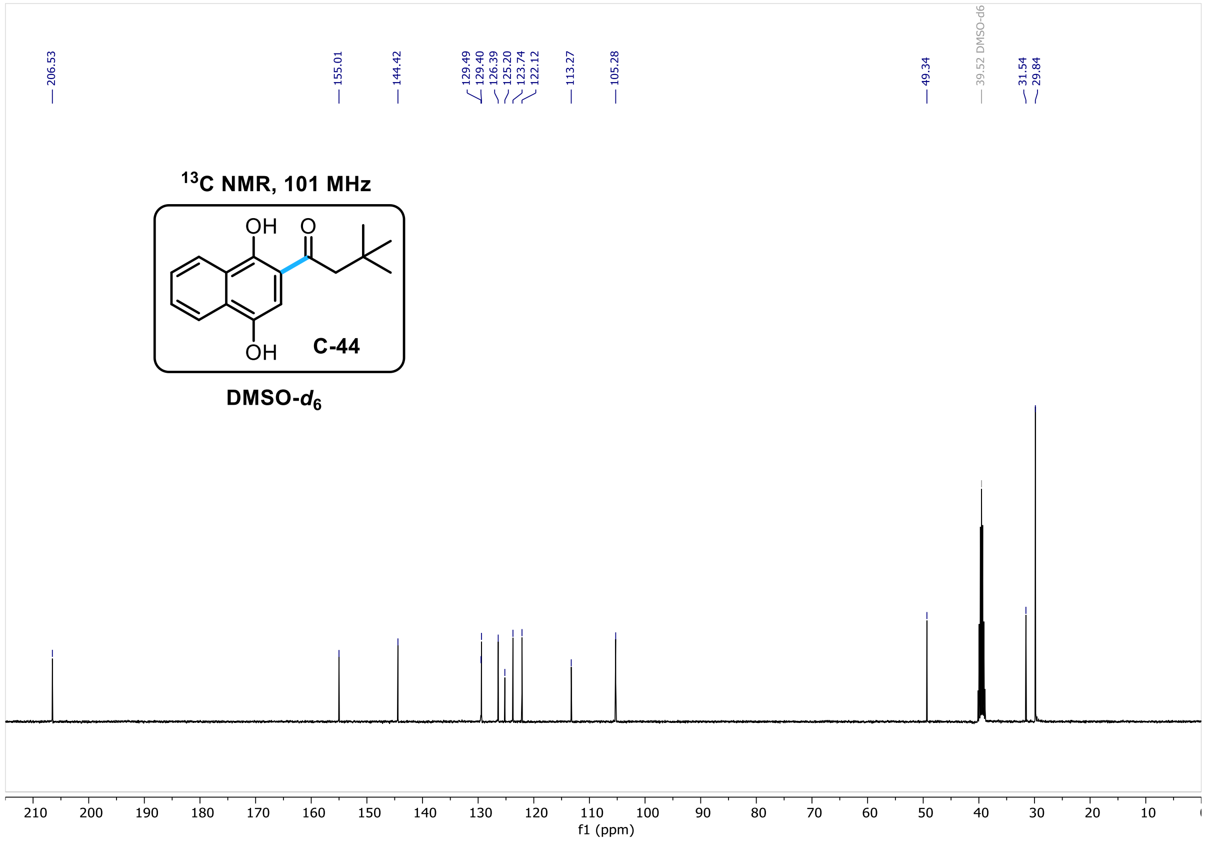
206.53
155.01
144.42
129.49 129.40 126.39 125.20 123.74 122.12
113.27
105.28
49.34
39.52 DMSO-d6
31.54 29.84
¹³C NMR, 101 MHz
OH
O
OH
C-44
DMSO-d6
f1 (ppm)

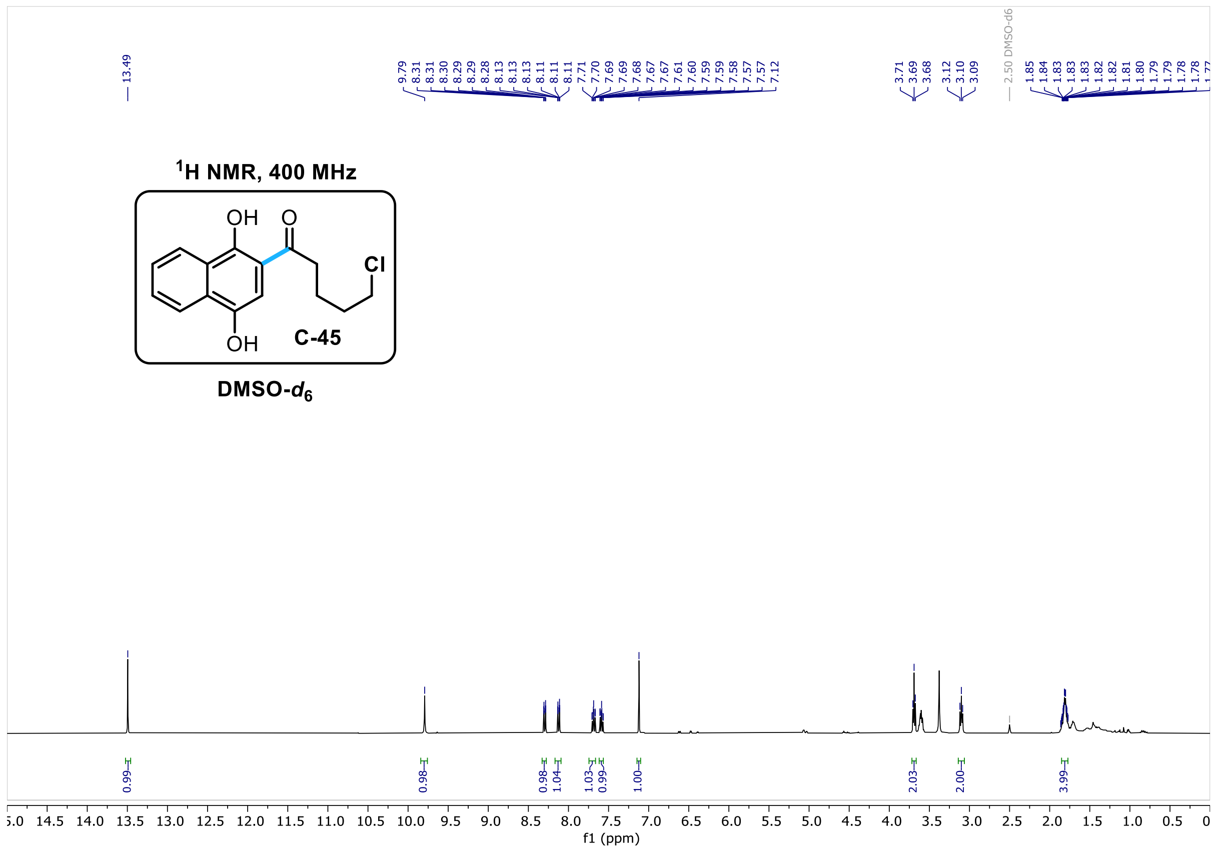

^{1}H NMR, 400 MHz
C-45
DMSO-d_6
f1 (ppm)

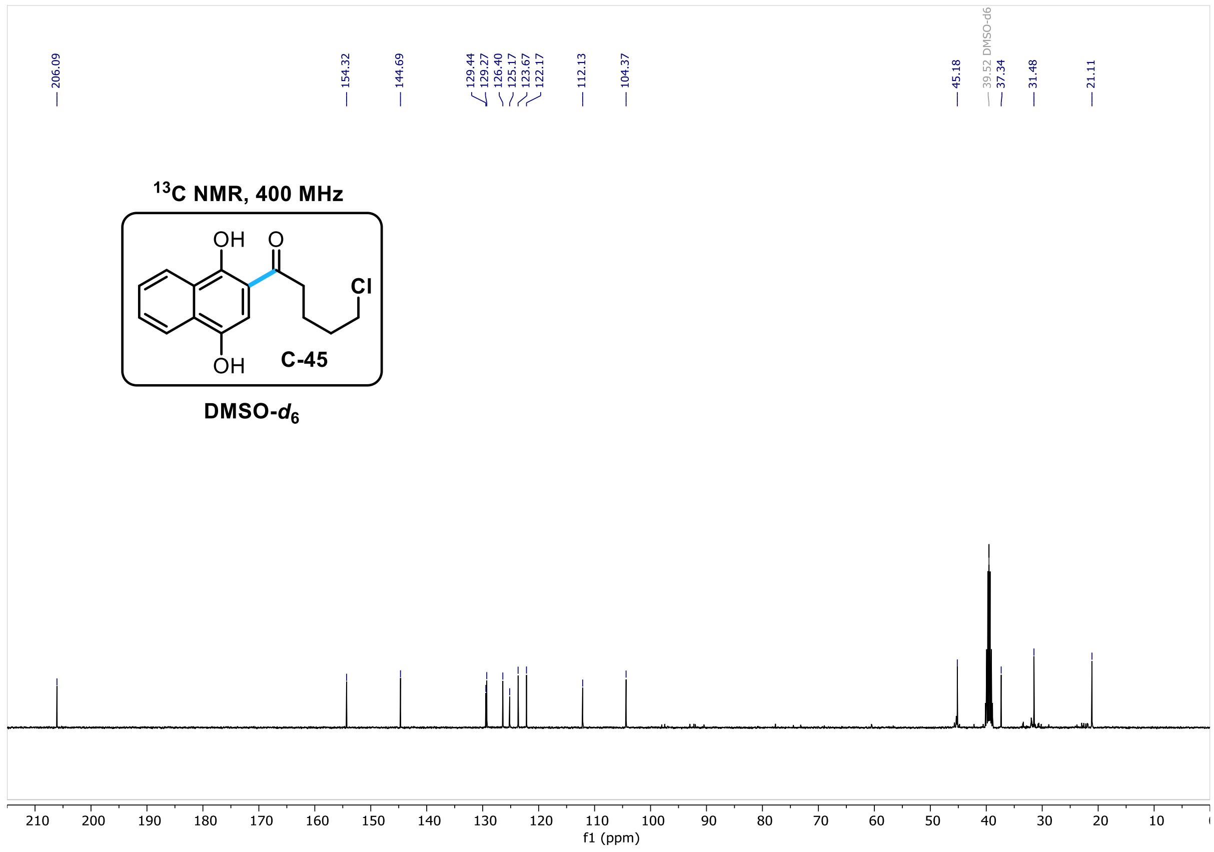

^{13}C NMR, 400 MHz
C-45
DMSO-d_6
f1 (ppm)

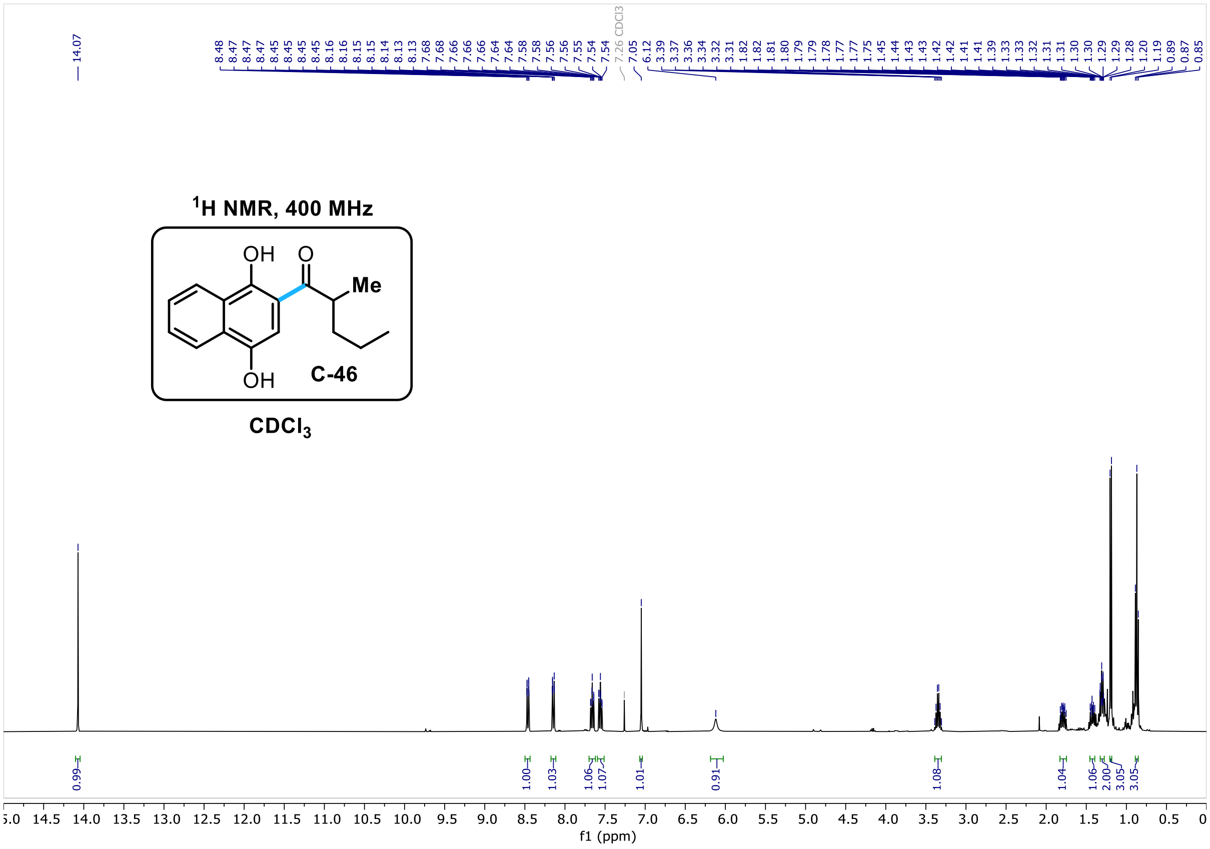

1H NMR, 400 MHz
OH
O
Me
OH
C-46
CDCl3
14.07
7.26 CDCl3
7.05
6.12
0.99
1.00
1.03
1.06
1.07
1.01
0.91
1.08
1.04
1.06
2.00
3.05
3.05
f1 (ppm)

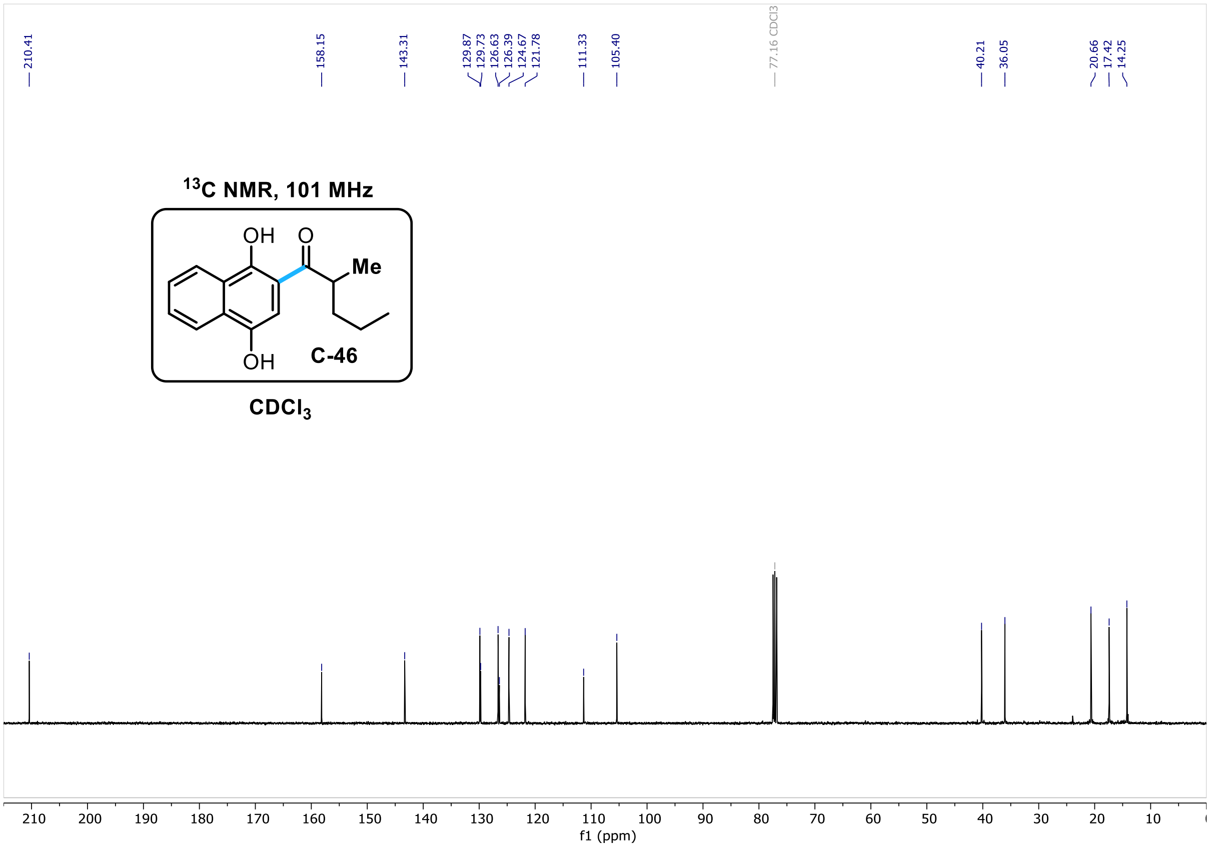

13C NMR, 101 MHz
OH
O
Me
OH
C-46
CDCl3
210.41
158.15
143.31
129.87
129.73
126.63
126.39
124.67
121.78
111.33
105.40
77.16 CDCl3
40.21
36.05
20.66
17.42
14.25
f1 (ppm)

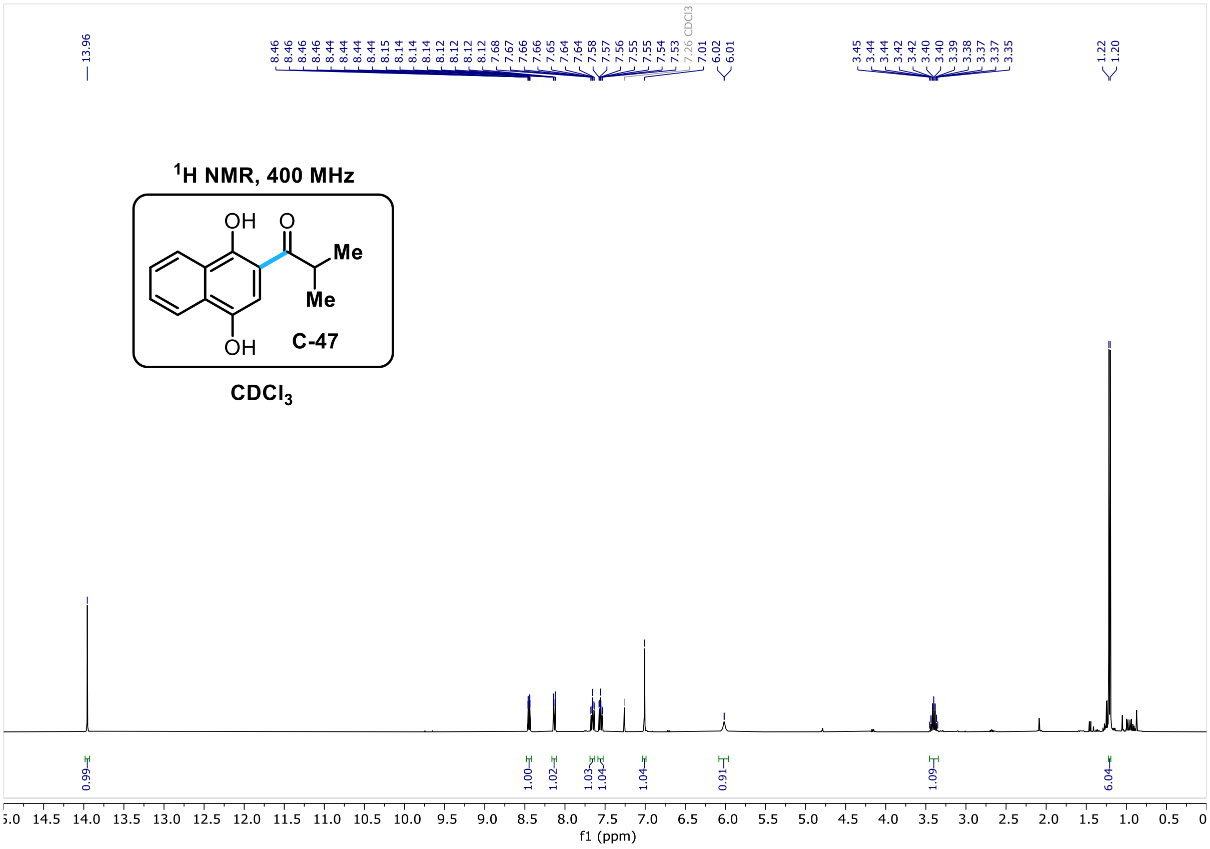

13.96
8.46 8.46 8.46 8.46 8.44 8.44 8.44 8.44 8.15 8.14 8.14 8.14 8.12 8.12 8.12 8.12 7.68 7.67 7.66 7.66 7.65 7.64 7.64 7.58 7.57 7.56 7.55 7.55 7.54 7.53 7.26 CDCl3 7.01 6.02 6.01
3.45 3.44 3.44 3.42 3.42 3.40 3.40 3.39 3.38 3.37 3.37 3.35
1.22 1.20
1H NMR, 400 MHz
OH
O
Me
Me
OH
C-47
CDCl3
0.99
1.00
1.02
1.03
1.04
1.04
0.91
1.09
6.04
f1 (ppm)

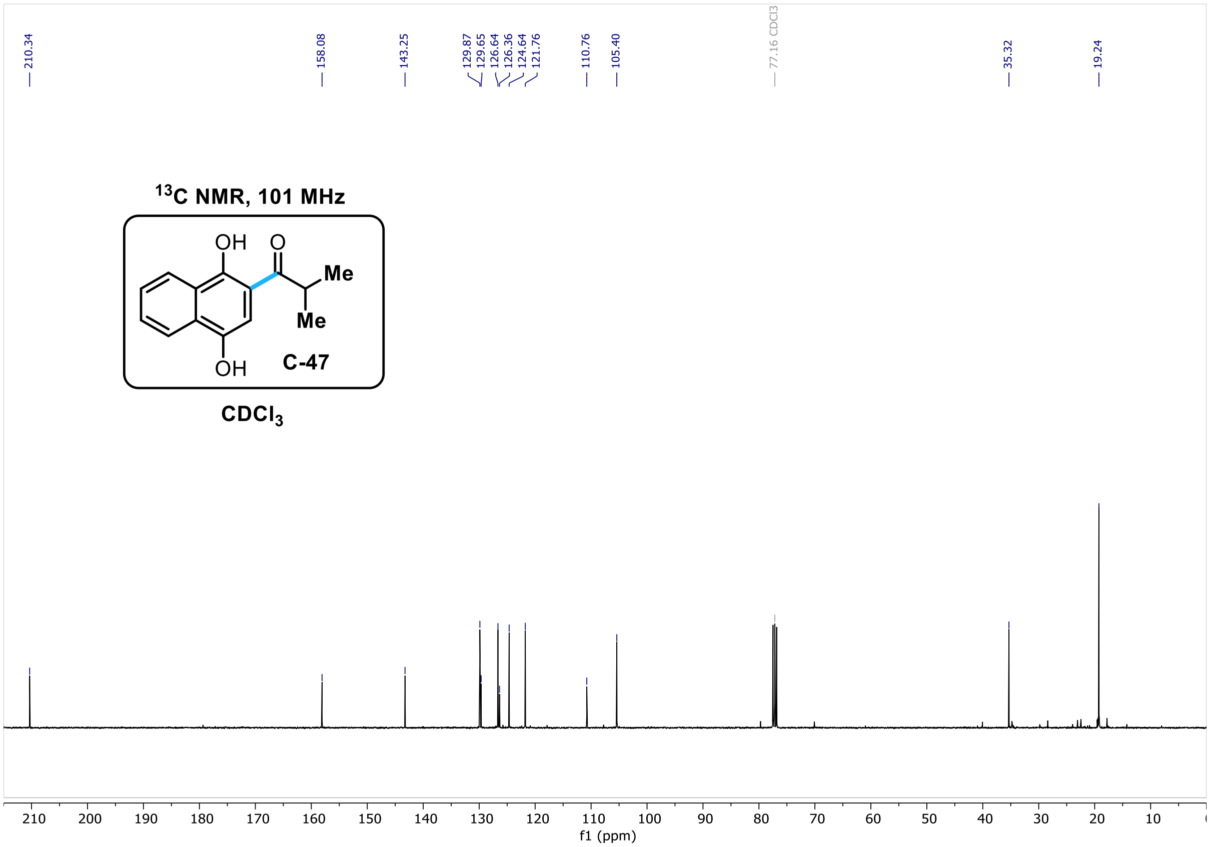

210.34
158.08
143.25
129.87 129.65 126.64 126.36 124.64 121.76
110.76
105.40
77.16 CDCl3
35.32
19.24
13C NMR, 101 MHz
OH
O
Me
Me
OH
C-47
CDCl3
f1 (ppm)

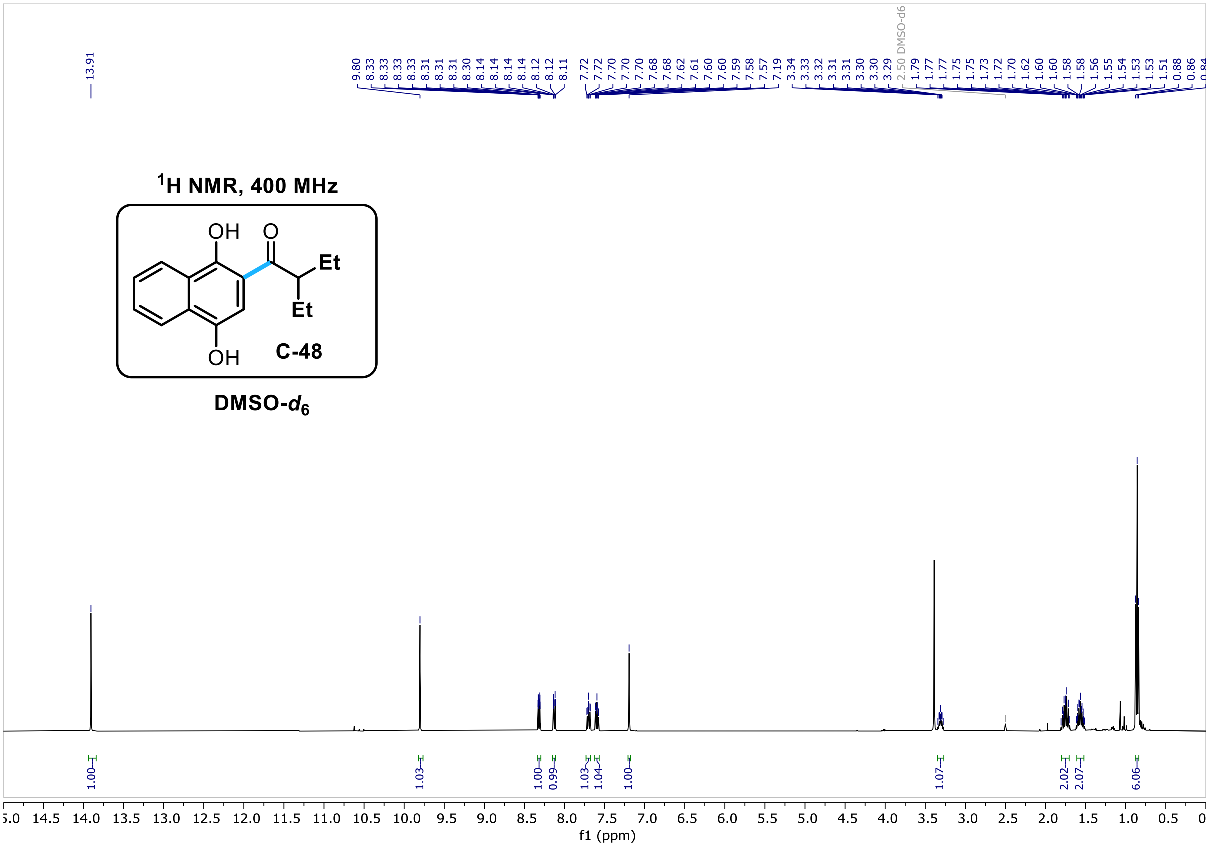
13.91
9.80 8.33 8.33 8.33 8.33 8.31 8.31 8.31 8.30 8.14 8.14 8.14 8.14 8.12 8.12 8.11
7.72 7.72 7.70 7.70 7.70 7.68 7.62 7.61 7.60 7.60 7.59 7.58 7.57 7.19
3.34 3.33 3.32 3.31 3.31 3.30 3.29
2.50 DMSO-d6
1.79 1.77 1.77 1.75 1.75 1.73 1.72 1.70 1.62 1.60 1.60 1.58 1.58 1.56 1.55 1.54 1.53 1.53 1.51 0.88 0.86
1H NMR, 400 MHz
OH
O
Et
Et
OH
C-48
DMSO-d6
1.00
1.03
1.00
0.99
1.03
1.04
1.00
1.07
2.02
2.07
6.06
f1 (ppm)

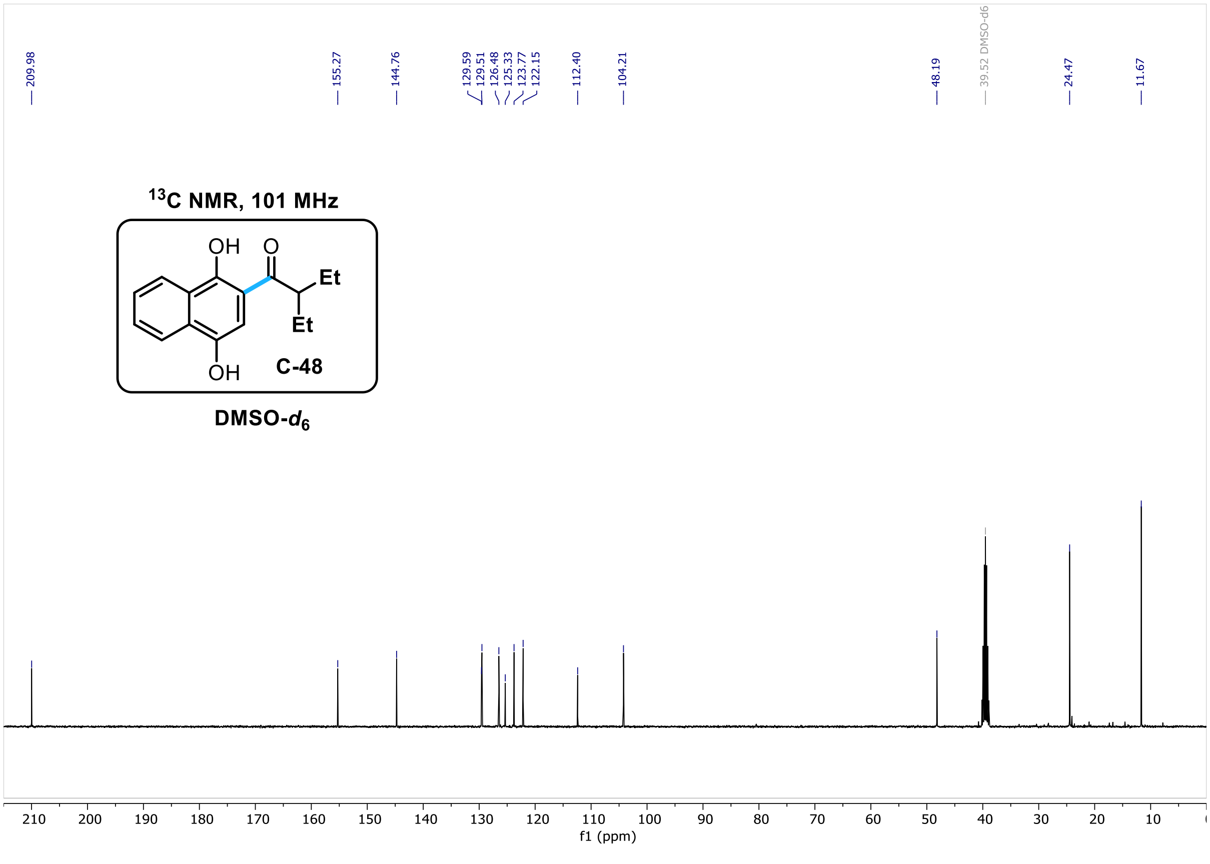
209.98
155.27
144.76
129.59 129.51 126.48 125.33 123.77 122.15
112.40
104.21
48.19
39.52 DMSO-d6
24.47
11.67
13C NMR, 101 MHz
OH
O
Et
Et
OH
C-48
DMSO-d6
f1 (ppm)

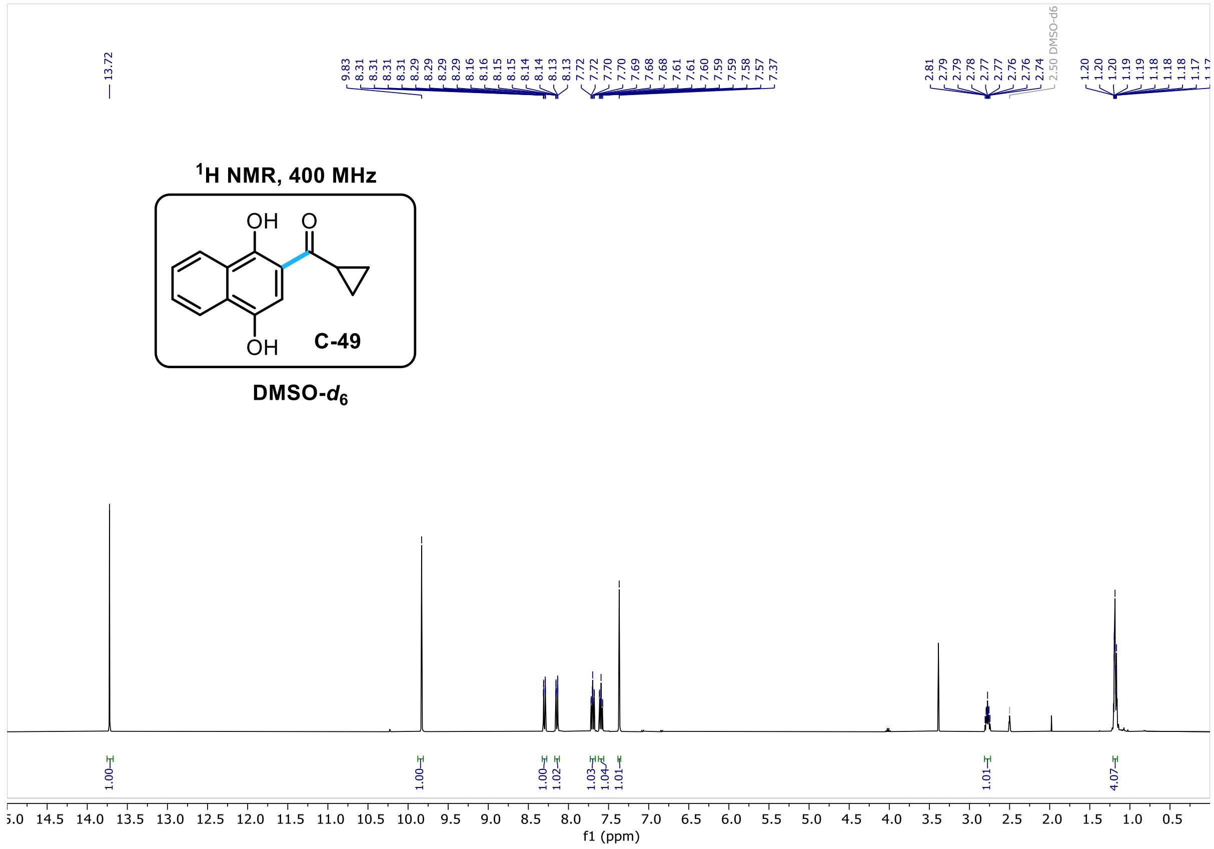
13.72
9.83 8.31 8.31 8.31 8.31 8.29 8.29 8.29 8.29 8.16 8.16 8.15 8.15 8.14 8.14 8.13 8.13 7.72 7.72 7.70 7.70 7.69 7.68 7.68 7.61 7.61 7.60 7.59 7.59 7.58 7.57 7.37
2.81 2.79 2.79 2.78 2.77 2.77 2.76 2.76 2.74 2.50 DMSO-d6
1.20 1.20 1.20 1.19 1.19 1.18 1.18 1.18 1.17
1H NMR, 400 MHz
OH
O
OH
C-49
DMSO-d6
1.00
1.00
1.00
1.02
1.03
1.04
1.01
1.01
4.07
f1 (ppm)

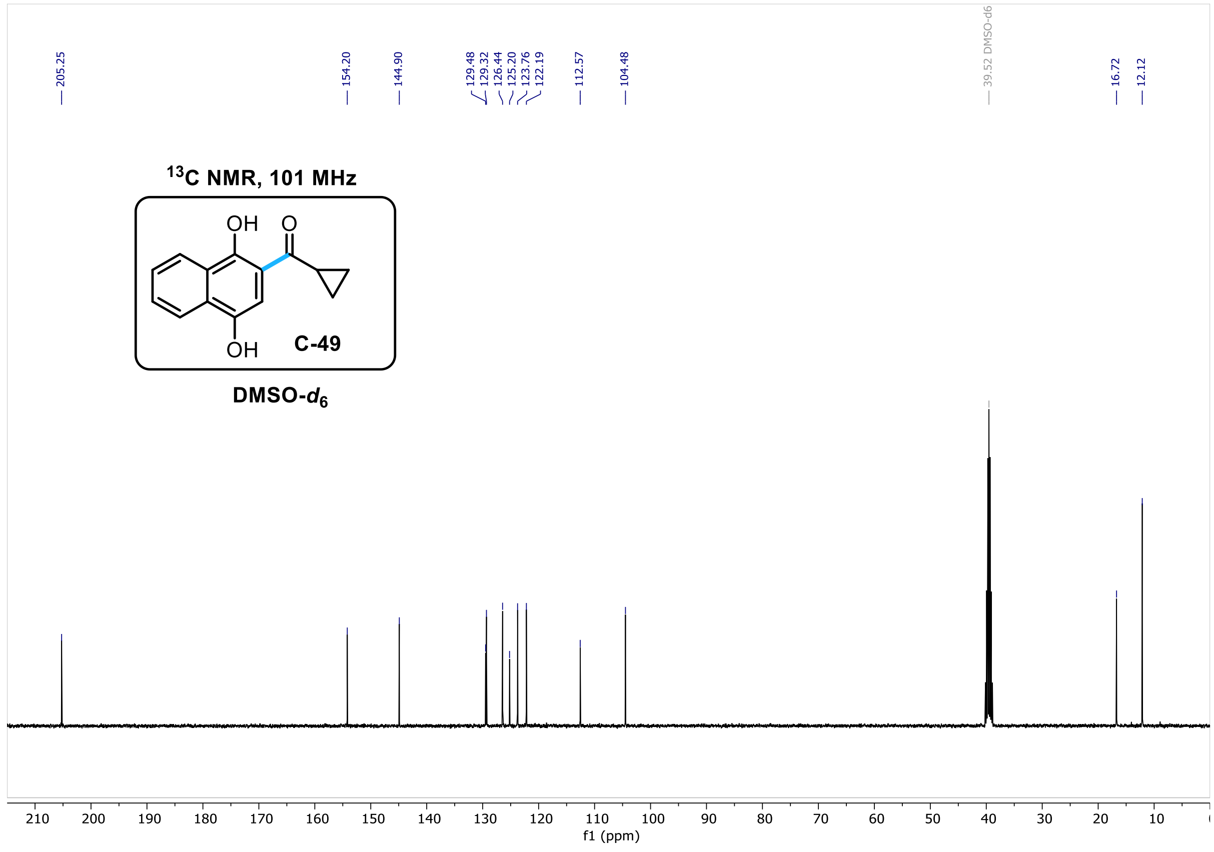
205.25
154.20
144.90
129.48 129.32 126.44 125.20 123.76 122.19
112.57
104.48
39.52 DMSO-d6
16.72
12.12
13C NMR, 101 MHz
OH
O
OH
C-49
DMSO-d6
f1 (ppm)

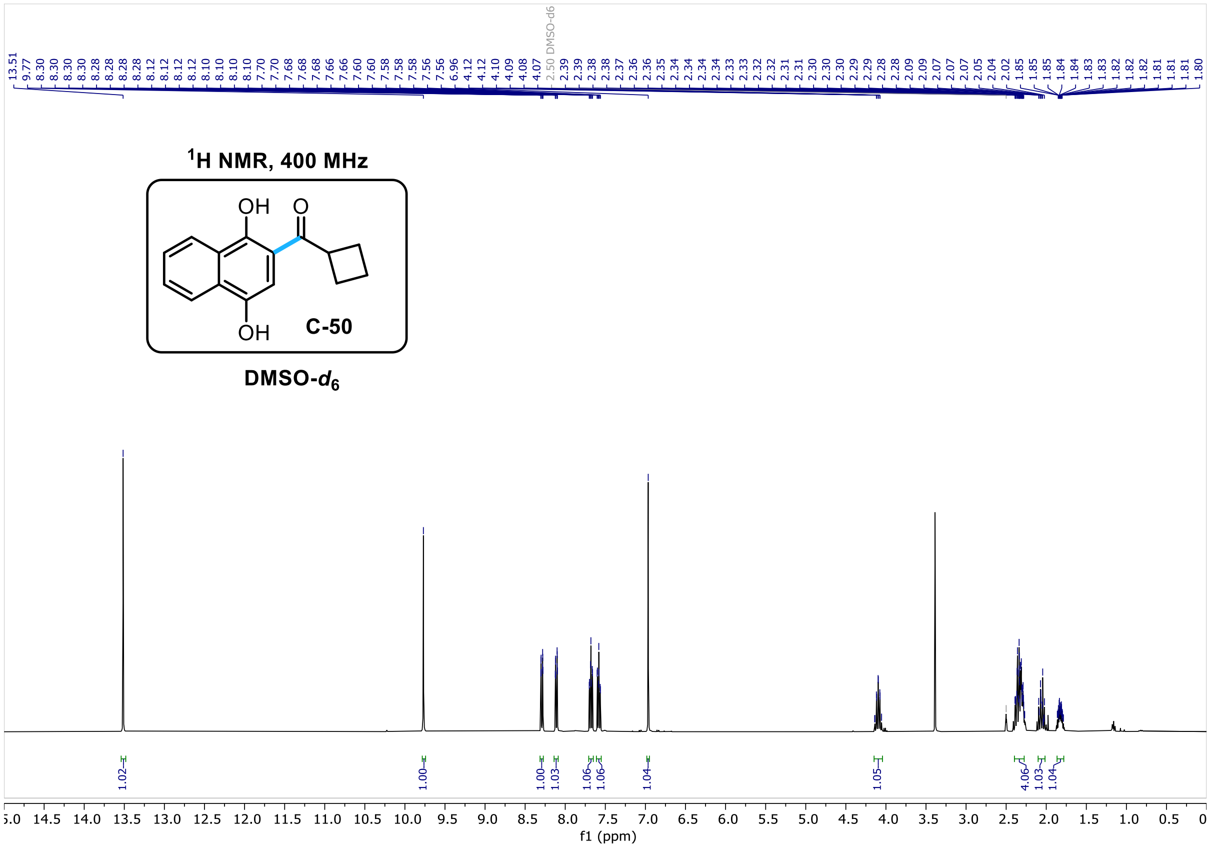

1H NMR, 400 MHz
OH
O
OH
C-50
DMSO-d6
13.51
9.77
2.50 DMSO-d6
1.02
1.00
1.00
1.03
1.06
1.06
1.04
1.05
4.06
1.03
1.04
f1 (ppm)

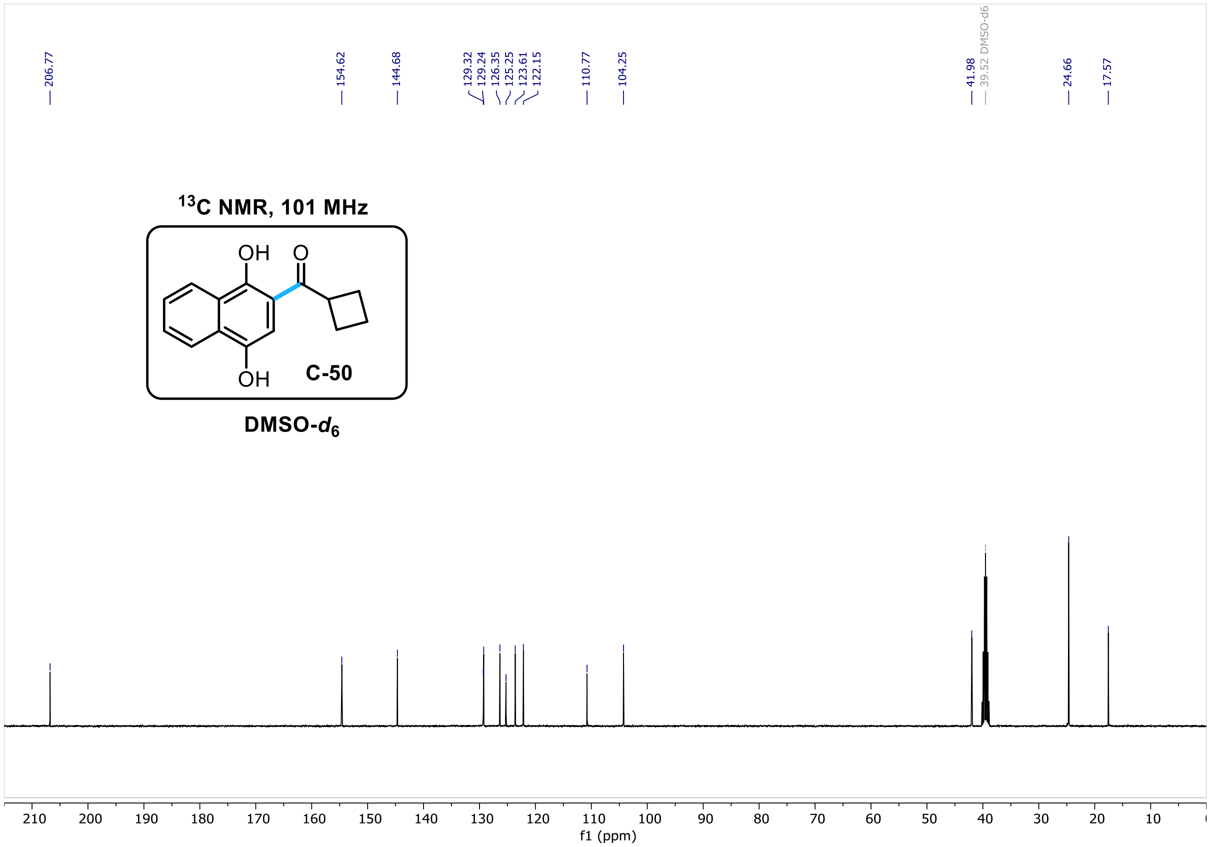

206.77
154.62
144.68
129.32
129.24
126.35
125.25
123.61
122.15
110.77
104.25
41.98
39.52 DMSO-d6
24.66
17.57
13C NMR, 101 MHz
OH
O
OH
C-50
DMSO-d6
f1 (ppm)

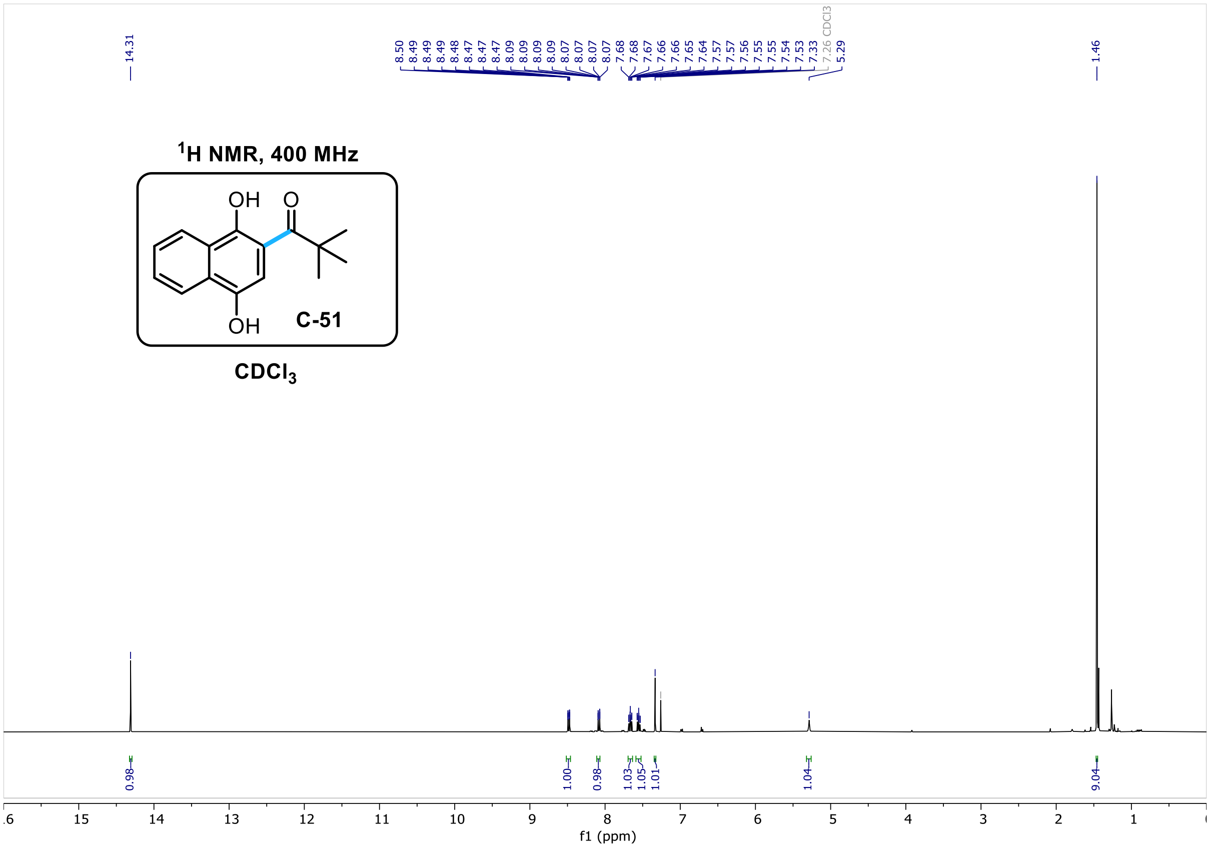
14.31
8.50 8.49 8.49 8.49 8.48 8.47 8.47 8.47 8.09 8.09 8.09 8.09 8.09 8.07 8.07 8.07 8.07 7.68 7.68 7.67 7.66 7.66 7.65 7.64 7.57 7.57 7.56 7.55 7.55 7.54 7.53 7.33 7.26 CDCl3 5.29
1.46
1H NMR, 400 MHz
OH
O
OH
C-51
CDCl3
0.98
1.00
0.98
1.03
1.05
1.01
1.04
9.04
f1 (ppm)

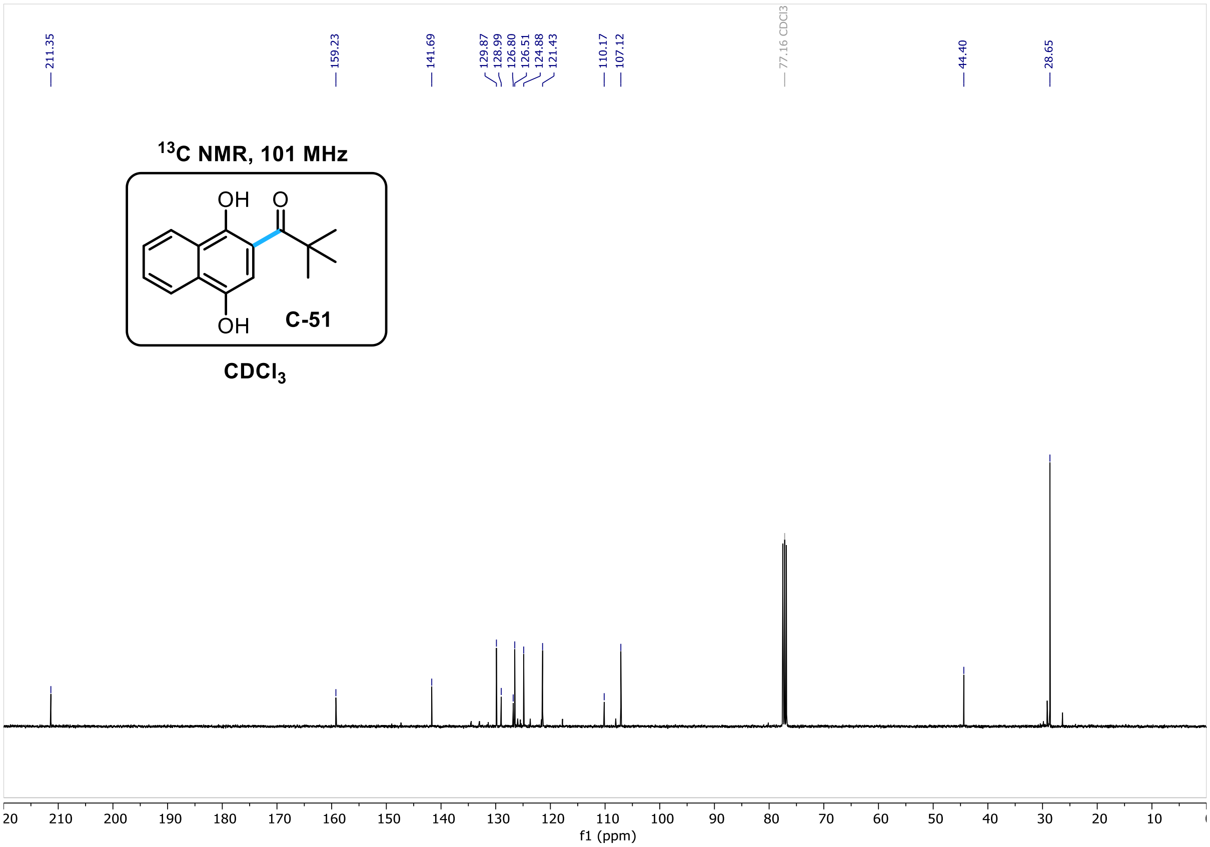
211.35
159.23
141.69
129.87 128.99 126.80 126.51 124.88 121.43
110.17
107.12
77.16 CDCl3
44.40
28.65
13C NMR, 101 MHz
OH
O
OH
C-51
CDCl3
f1 (ppm)

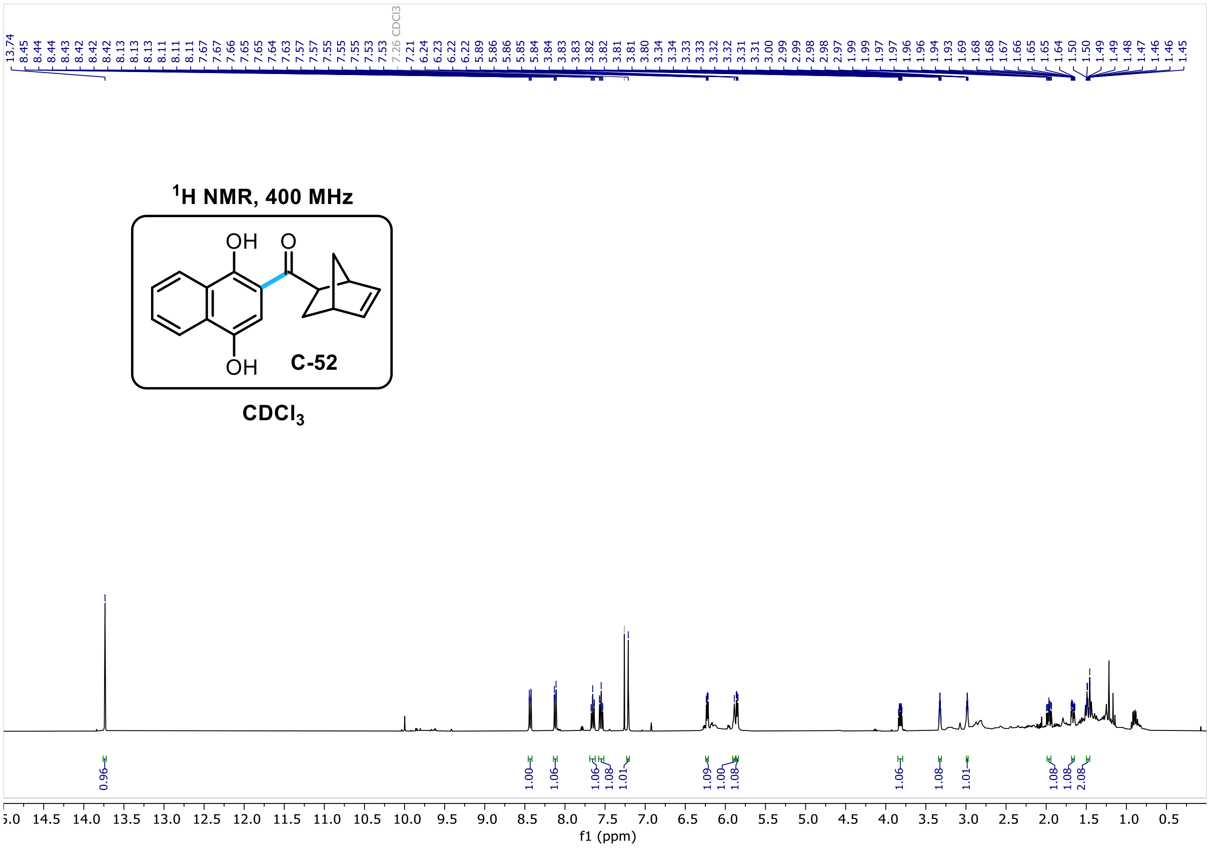
1H NMR, 400 MHz
OH
O
OH
C-52
CDCl3
f1 (ppm)

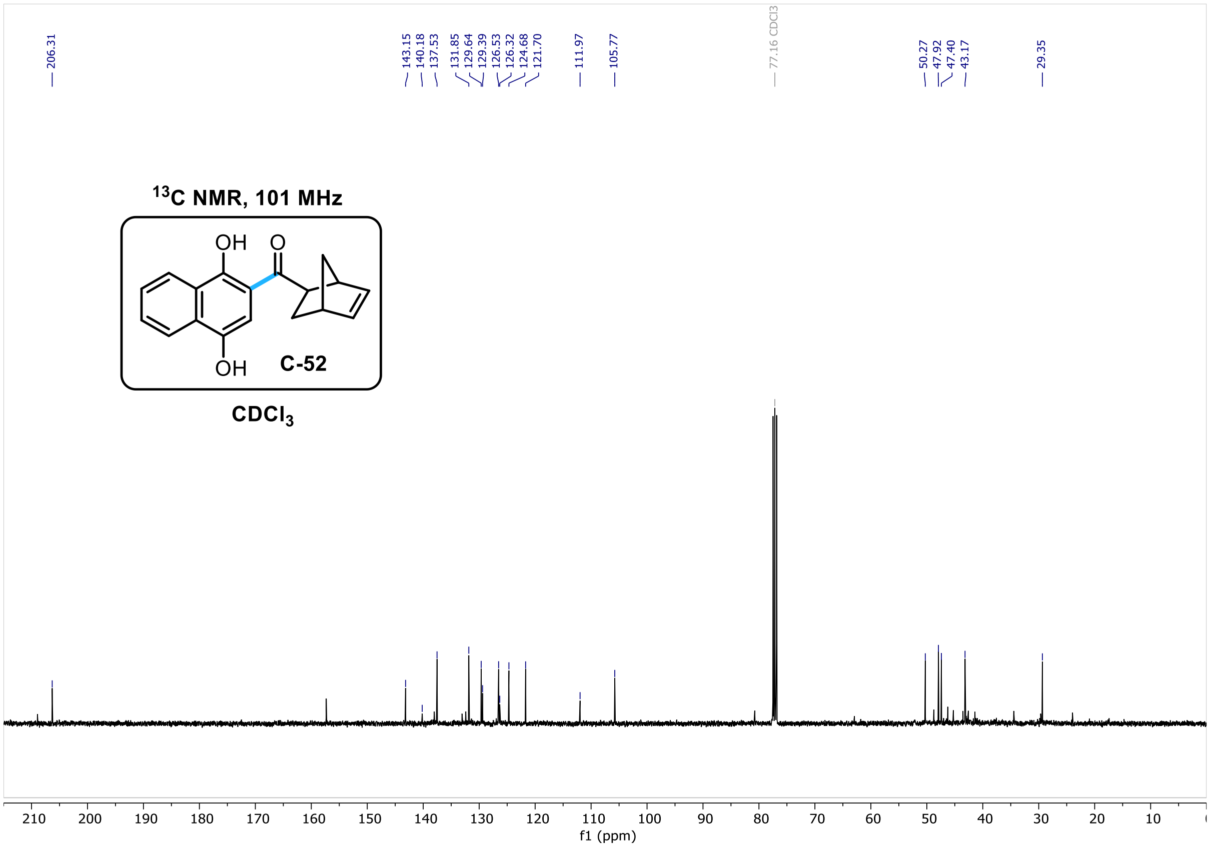
206.31
143.15
140.18
137.53
131.85
129.64
129.39
126.53
126.32
124.68
121.70
111.97
105.77
77.16 CDCl3
50.27
47.92
47.40
43.17
29.35
13C NMR, 101 MHz
OH
O
OH
C-52
CDCl3
f1 (ppm)

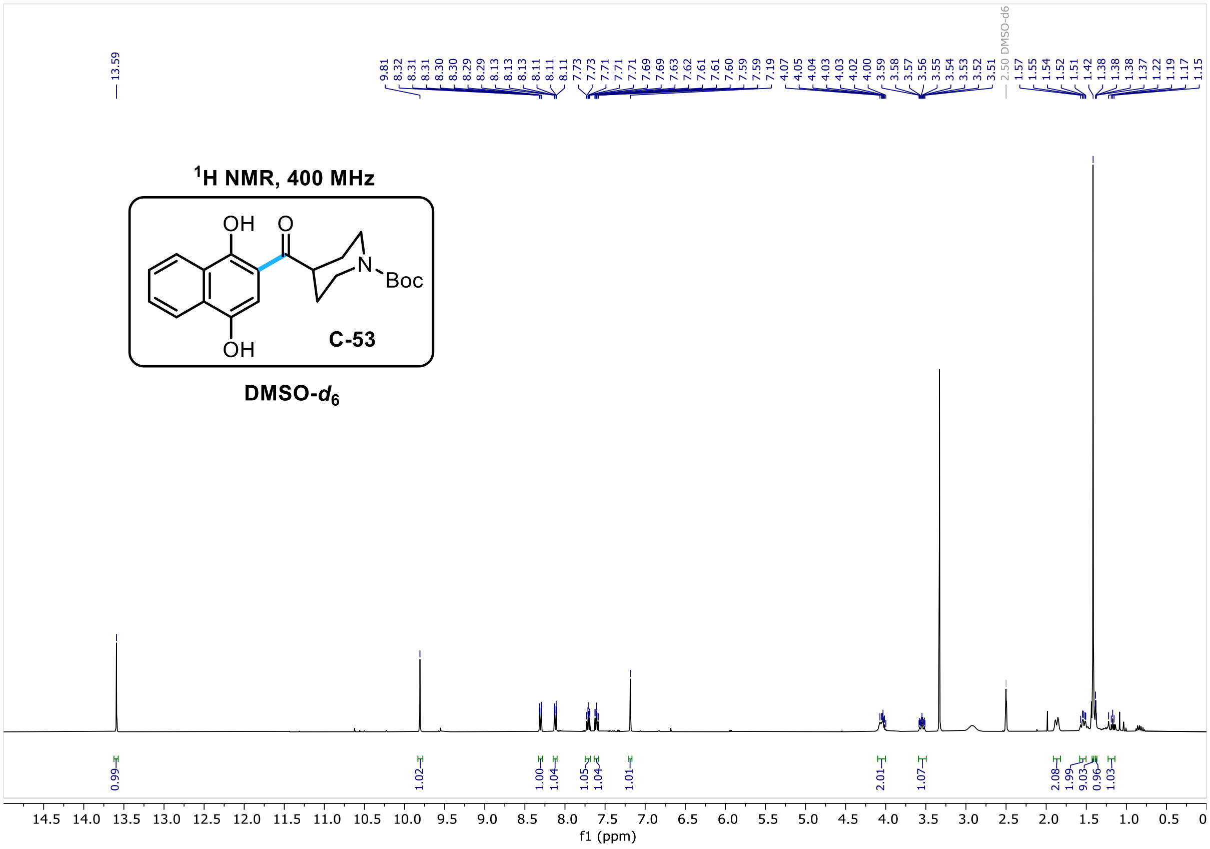
1H NMR, 400 MHz
OH
O
N
Boc
OH
C-53
DMSO-d6
13.59
9.81 8.32 8.31 8.31 8.30 8.30 8.29 8.29 8.13 8.13 8.13 8.11 8.11 8.11 7.73 7.73 7.71 7.71 7.71 7.69 7.69 7.63 7.62 7.61 7.61 7.60 7.59 7.59 7.19
4.07 4.05 4.04 4.03 4.03 4.02 4.00 3.59 3.58 3.57 3.56 3.55 3.54 3.53 3.52 3.51
2.50 DMSO-d6
1.57 1.55 1.54 1.52 1.51 1.42 1.38 1.38 1.38 1.37 1.22 1.19 1.17 1.15
0.99 1.02 1.00 1.04 1.05 1.04 1.01 2.01 1.07 2.08 1.99 9.03 0.96 1.03
f1 (ppm)

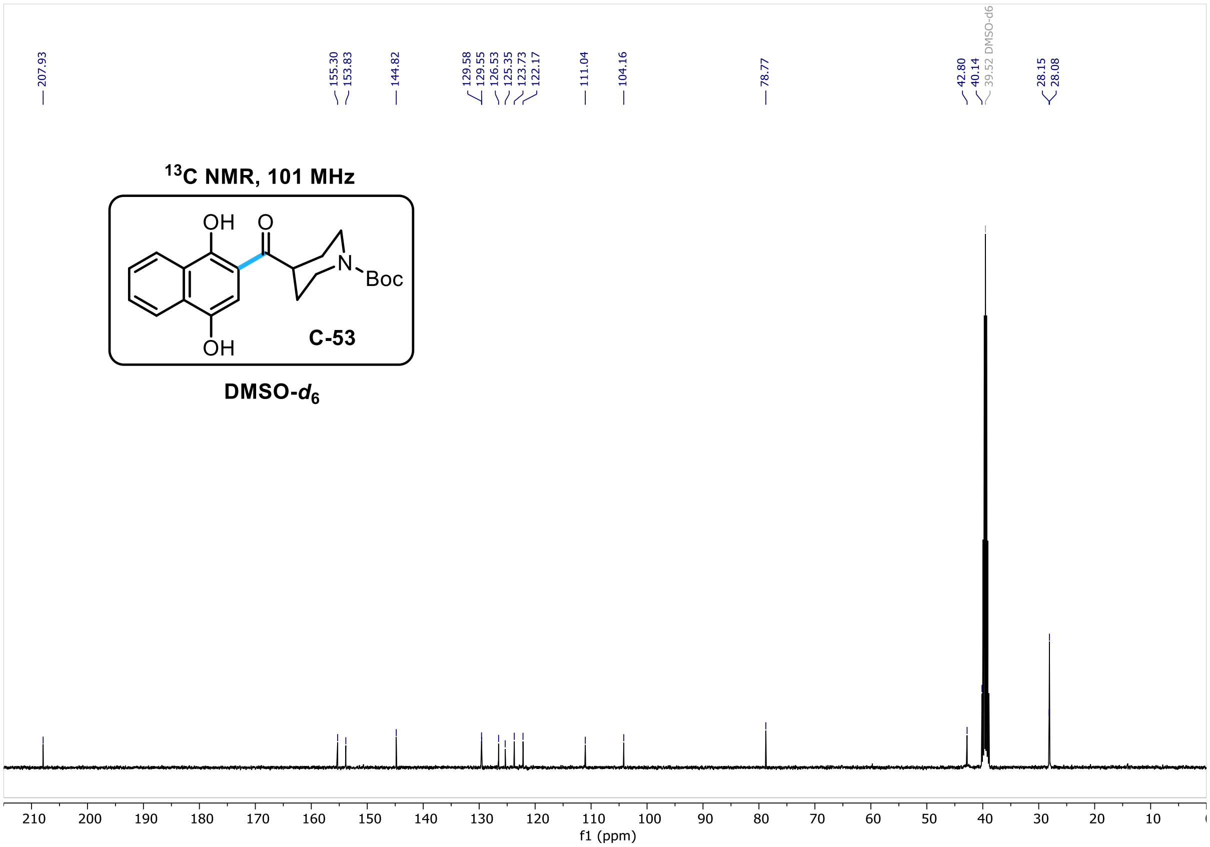
13C NMR, 101 MHz
OH
O
N
Boc
OH
C-53
DMSO-d6
207.93
155.30 153.83 144.82
129.58 129.55 126.53 125.35 123.73 122.17 111.04 104.16
78.77
42.80 40.14 39.52 DMSO-d6
28.15 28.08
f1 (ppm)

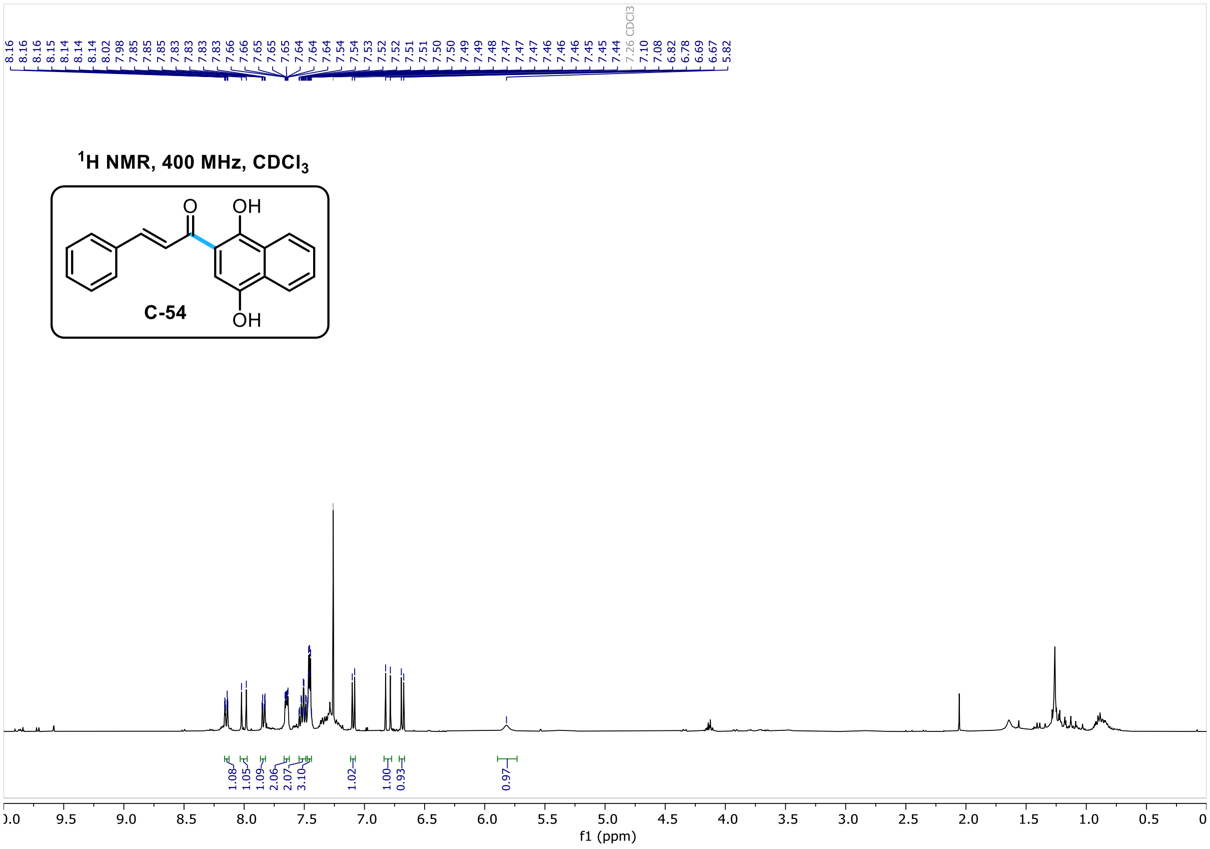

8.16 8.16 8.15 8.14 8.14 8.14 8.02 7.98 7.85 7.85 7.83 7.83 7.83 7.66 7.66 7.65 7.65 7.64 7.64 7.54 7.54 7.53 7.52 7.52 7.51 7.51 7.50 7.49 7.49 7.48 7.47 7.47 7.46 7.46 7.45 7.45 7.44 7.26 CDCl3 7.10 7.08 6.82 6.78 6.69 6.67 5.82
^{1}H NMR, 400 MHz, $CDCl_3$
O
OH
OH
C-54
1.08 1.05 1.09 2.06 2.07 3.10 1.02 1.00 0.93 0.97
f1 (ppm)

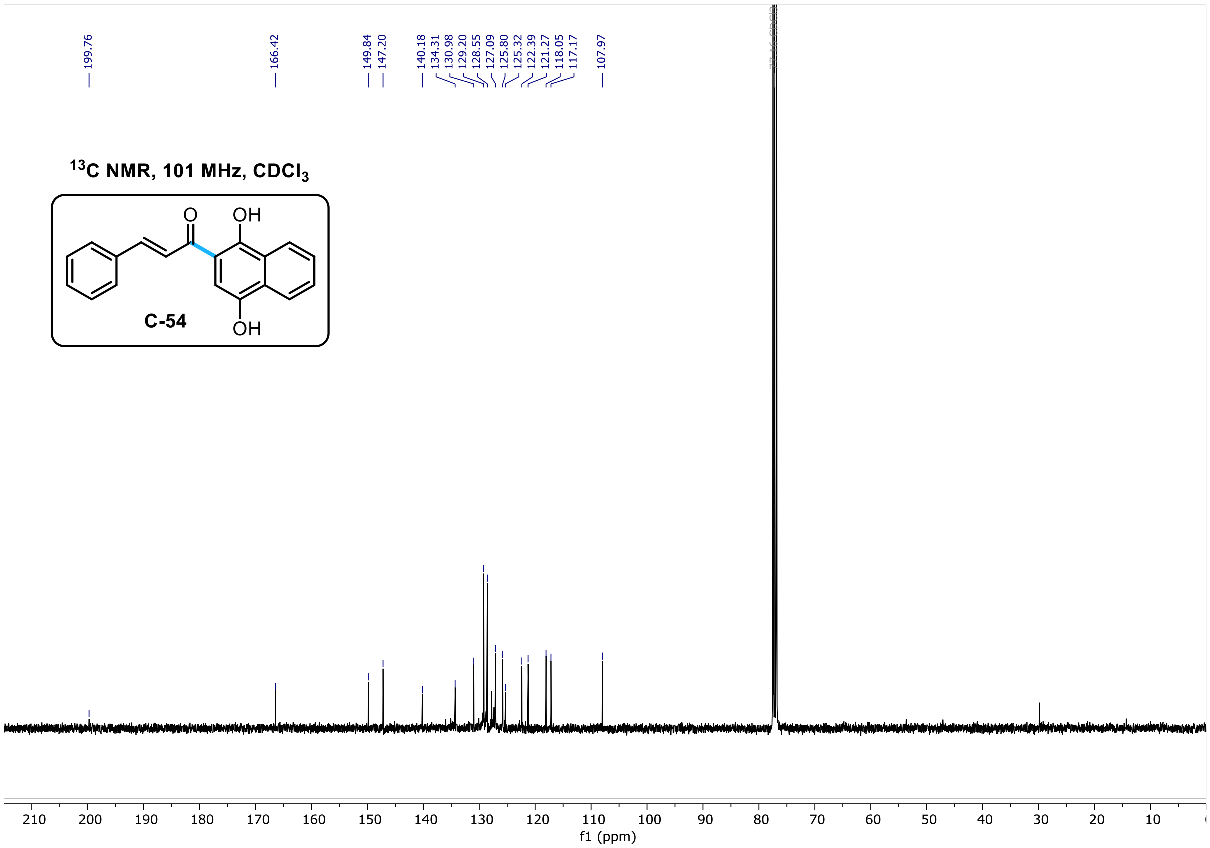

199.76
166.42
149.84 147.20
140.18 134.31 130.98 129.20 128.55 127.09 125.80 125.32 122.39 121.27 118.05 117.17
107.97
^{13}C NMR, 101 MHz, $CDCl_3$
O
OH
OH
C-54
f1 (ppm)

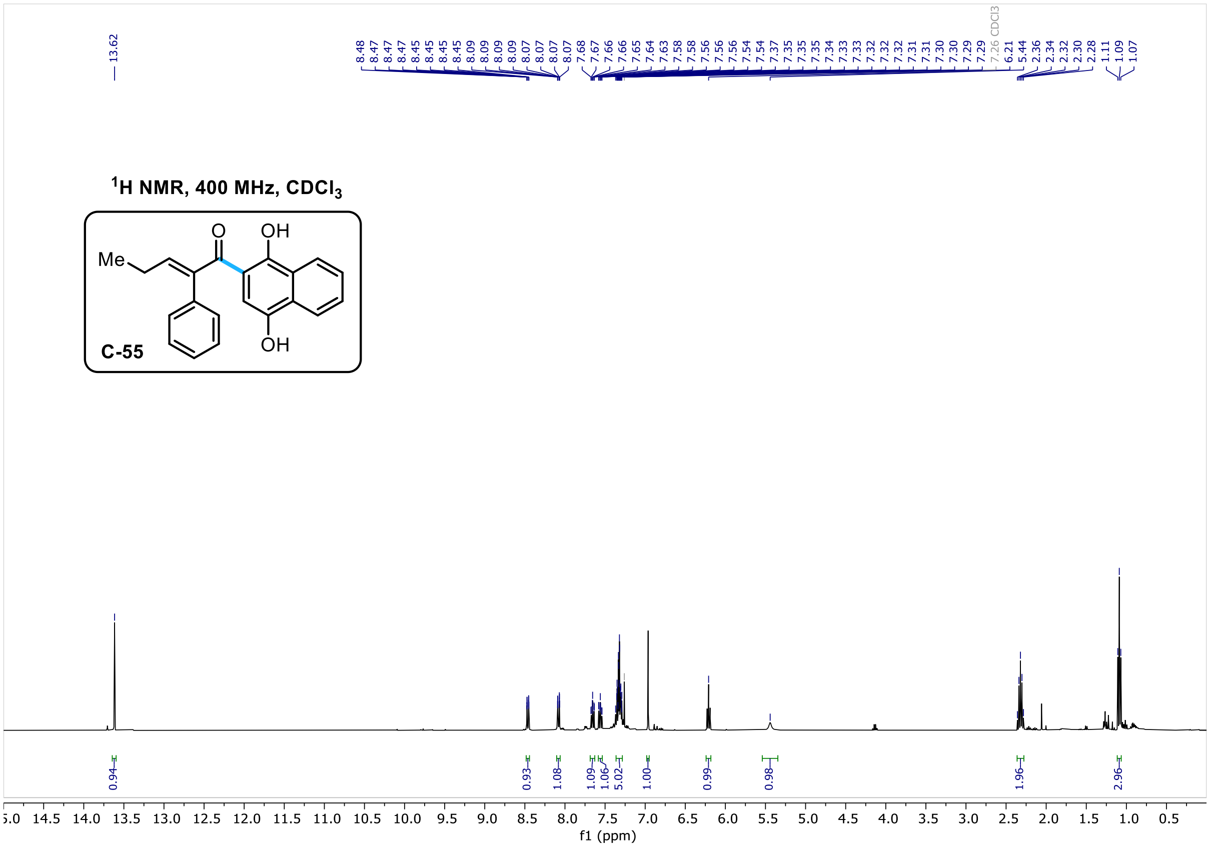

^{1}H NMR, 400 MHz, CDCl$_3$
C-55
f1 (ppm)

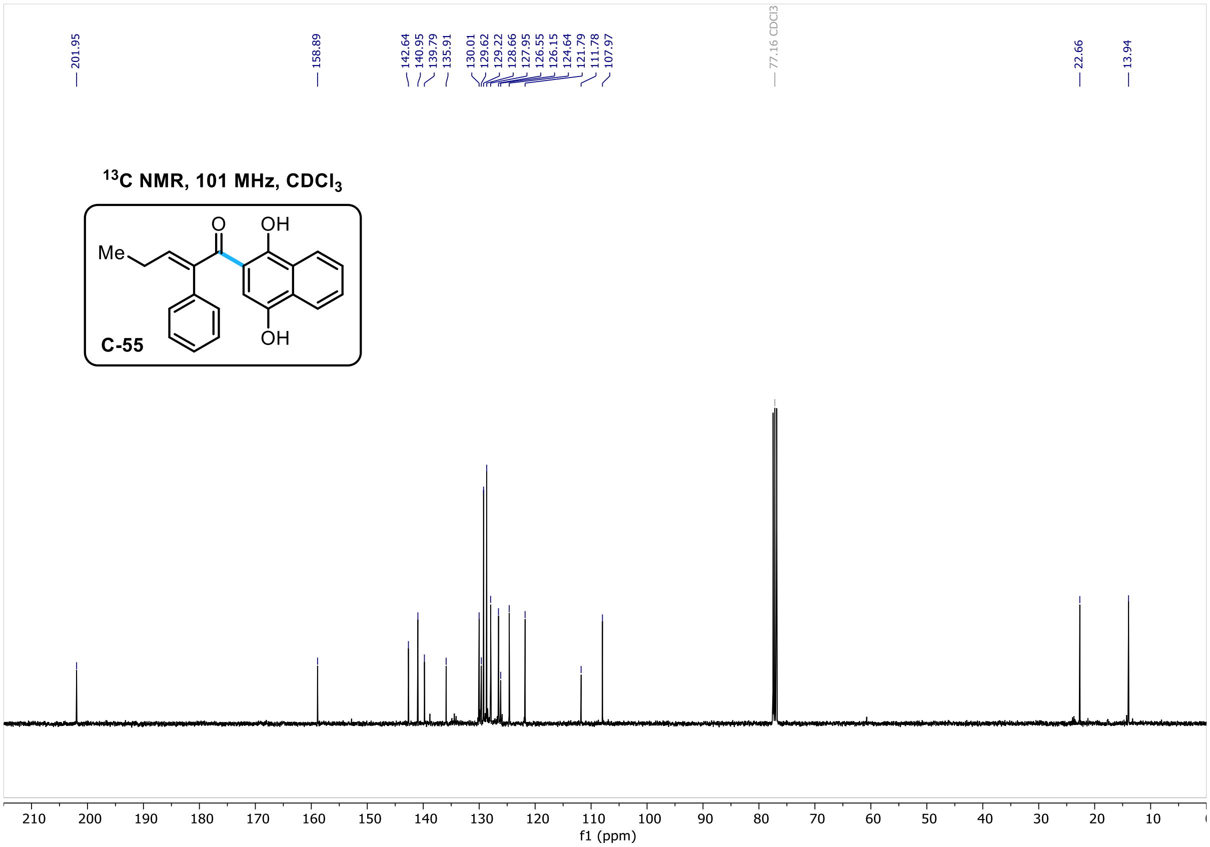

^{13}C NMR, 101 MHz, CDCl$_3$
C-55
f1 (ppm)

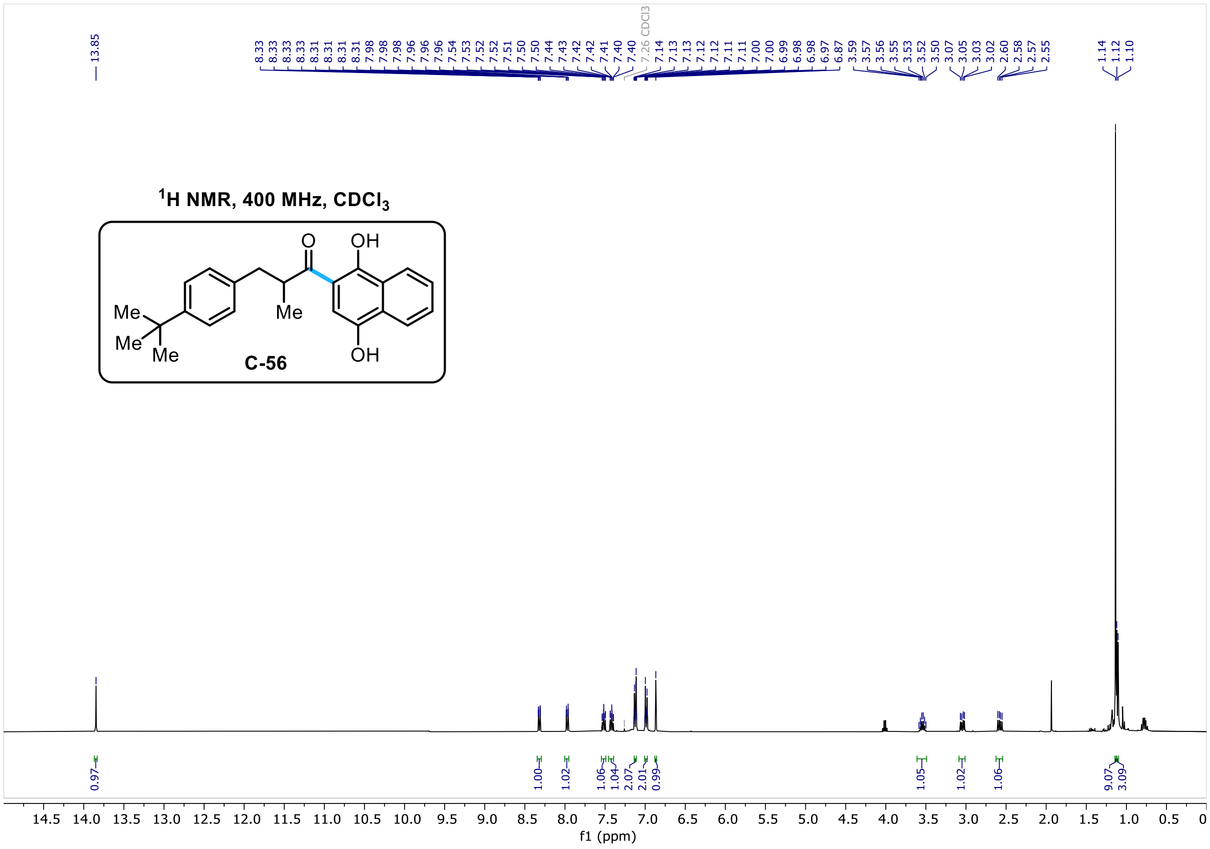

1H NMR, 400 MHz, CDCl3
C-56
f1 (ppm)

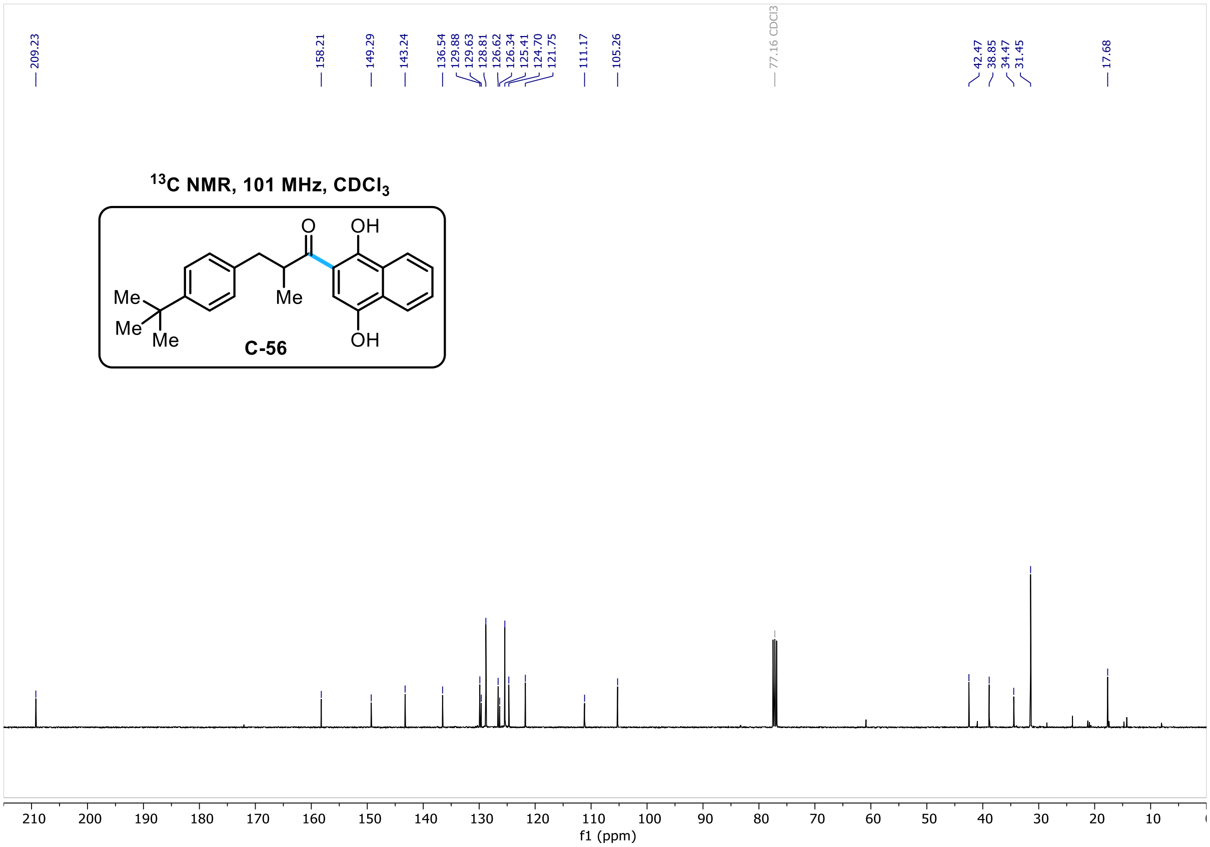

13C NMR, 101 MHz, CDCl3
C-56
f1 (ppm)

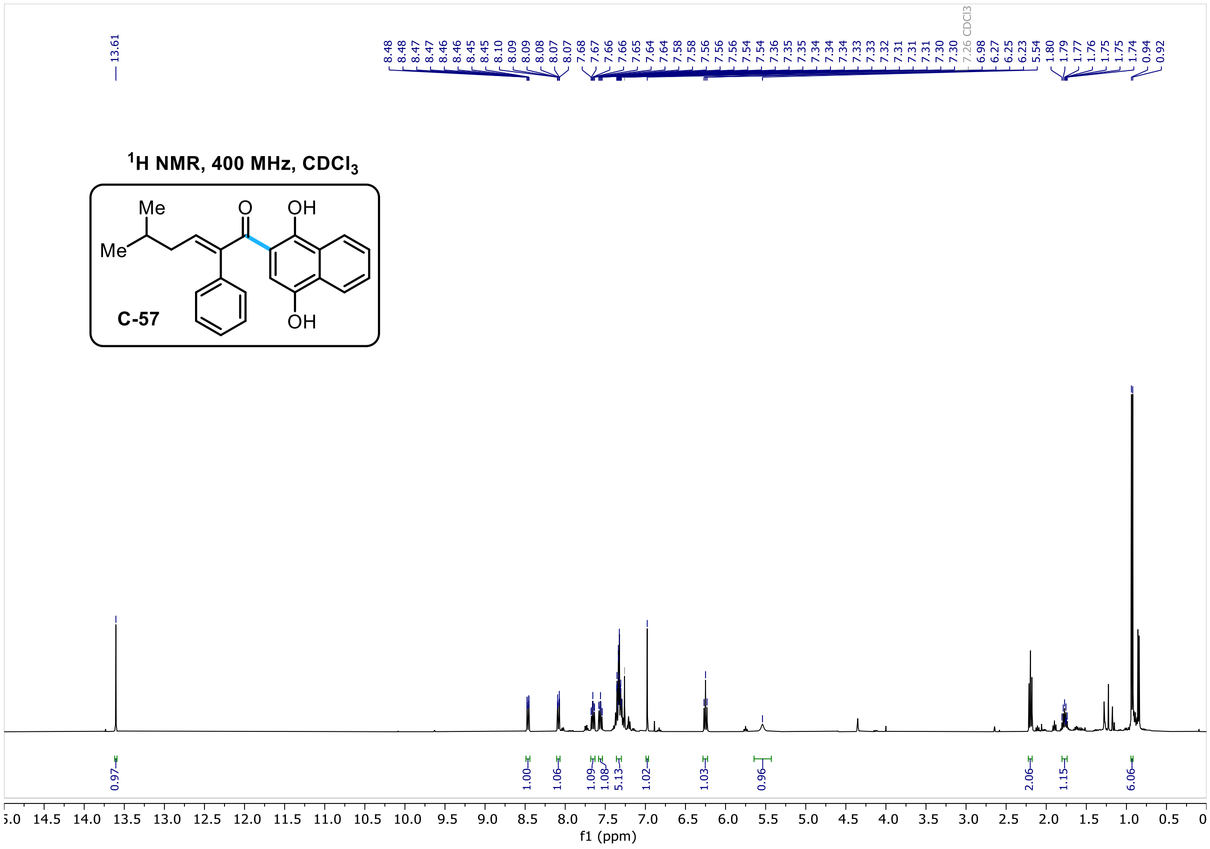
1H NMR, 400 MHz, CDCl3
C-57
Me
Me
O
OH
OH
13.61
8.48 8.48 8.47 8.47 8.46 8.46 8.45 8.45 8.10 8.09 8.09 8.08 8.07 8.07 7.68 7.67 7.66 7.66 7.65 7.64 7.64 7.58 7.58 7.56 7.56 7.56 7.54 7.54 7.36 7.35 7.35 7.34 7.34 7.33 7.33 7.32 7.31 7.31 7.30 7.30 7.26 CDCl3 6.98 6.27 6.25 6.23 5.54 1.80 1.79 1.77 1.76 1.75 1.75 1.74 0.94 0.92
0.97 1.00 1.06 1.09 1.08 5.13 1.02 1.03 0.96 2.06 1.15 6.06
f1 (ppm)

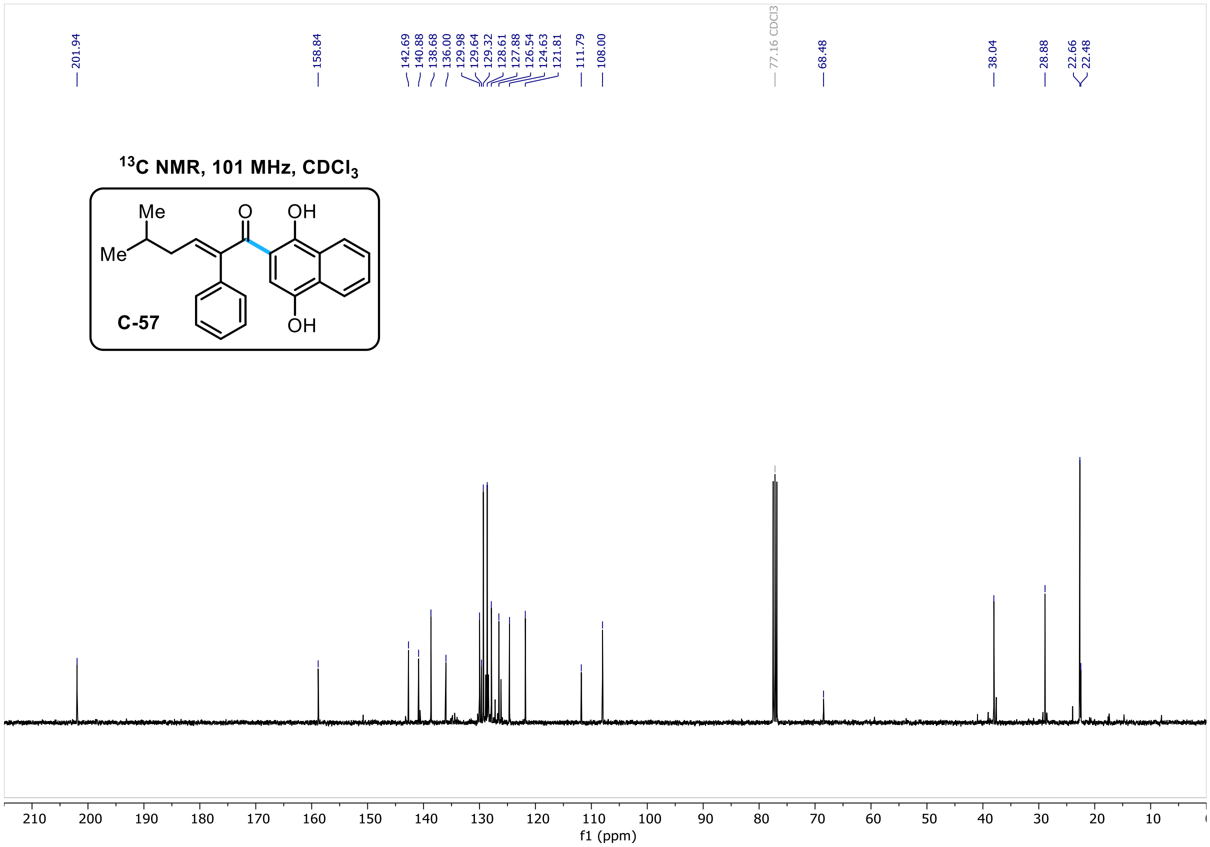
13C NMR, 101 MHz, CDCl3
C-57
Me
Me
O
OH
OH
201.94
158.84
142.69 140.88 138.68 136.00 129.98 129.64 129.32 128.61 127.88 126.54 124.63 121.81
111.79
108.00
77.16 CDCl3
68.48
38.04
28.88
22.66 22.48
f1 (ppm)

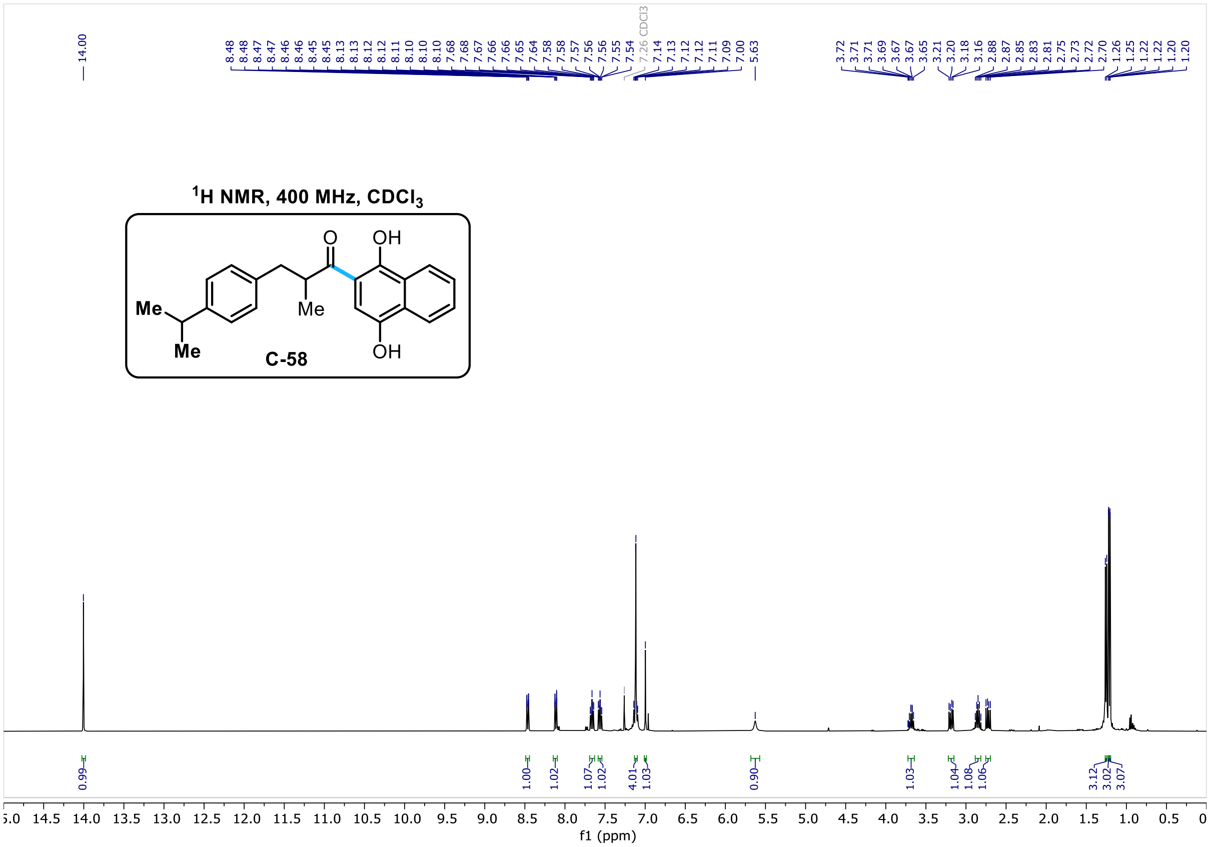

^{1}H NMR, 400 MHz, $CDCl_3$
C-58
14.00
8.48 8.48 8.47 8.47 8.46 8.46 8.45 8.45 8.13 8.13 8.12 8.12 8.11 8.10 8.10 8.10 7.68 7.67 7.66 7.66 7.65 7.64 7.58 7.58 7.57 7.56 7.56 7.55 7.54
7.26 CDCl3
7.14 7.13 7.12 7.12 7.11 7.09 7.00 5.63
3.72 3.71 3.71 3.69 3.67 3.67 3.65 3.21 3.20 3.18 3.16 2.88 2.87 2.85 2.83 2.81 2.75 2.73 2.72 2.70 1.26 1.25 1.22 1.22 1.20 1.20
0.99 1.00 1.02 1.07 1.02 4.01 1.03 0.90 1.03 1.04 1.08 1.06 3.12 3.02 3.07
f1 (ppm)

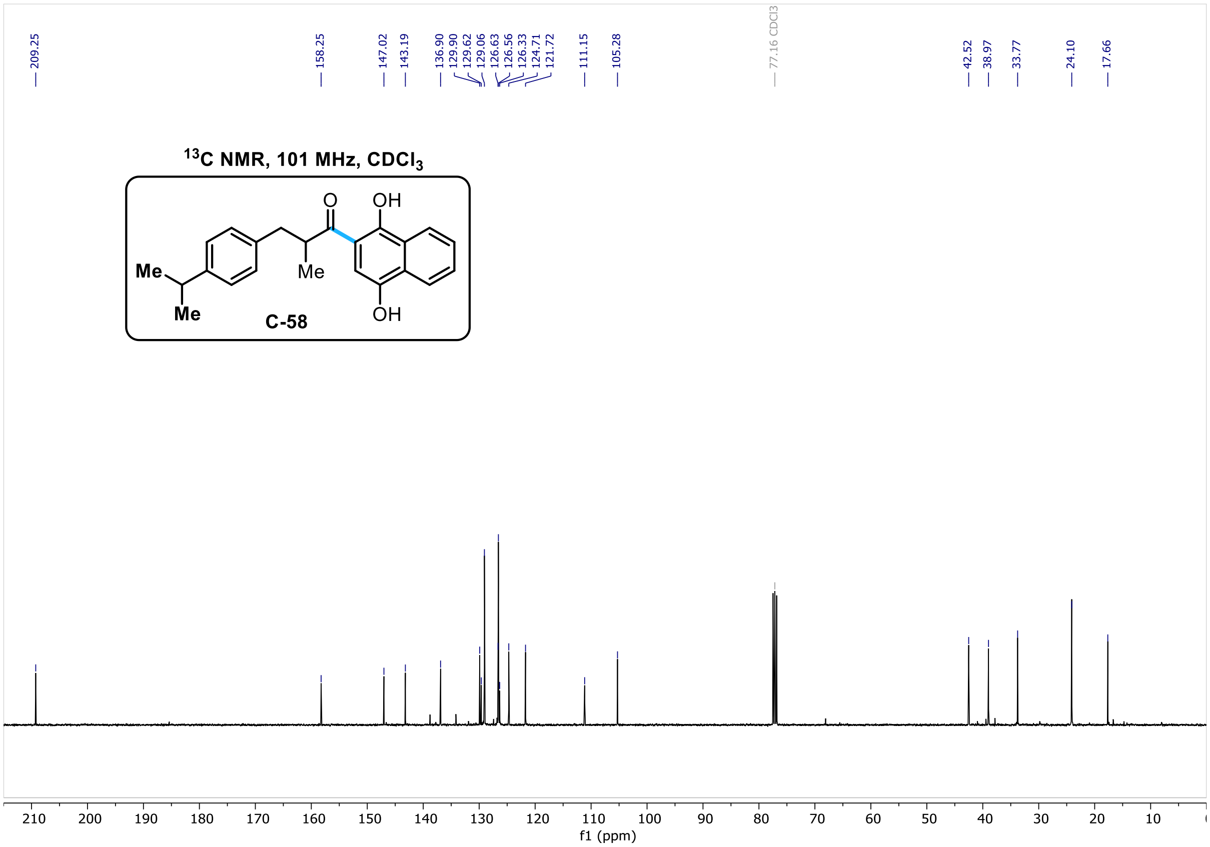

^{13}C NMR, 101 MHz, $CDCl_3$
C-58
209.25 158.25 147.02 143.19 136.90 129.90 129.62 129.06 126.63 126.56 126.33 124.71 121.72 111.15 105.28
77.16 CDCl3
42.52 38.97 33.77 24.10 17.66
f1 (ppm)

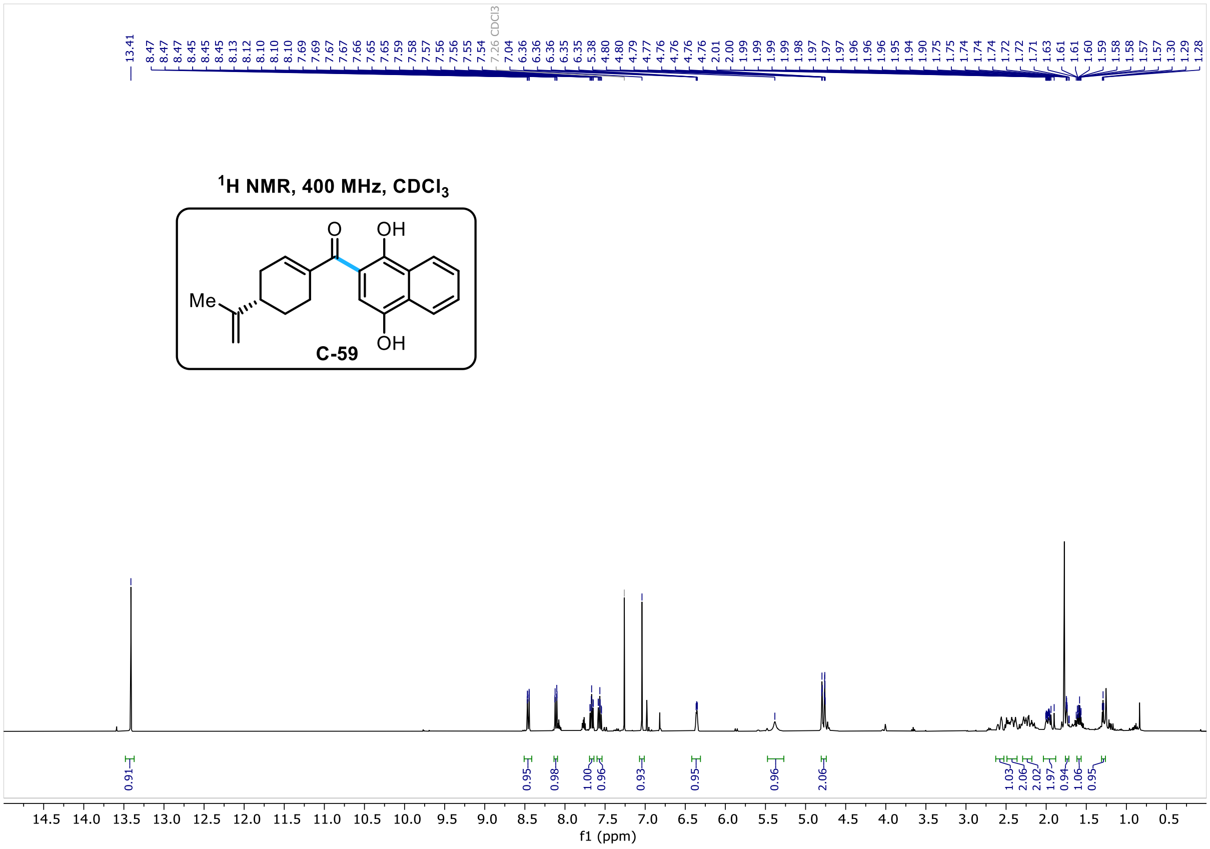
1H NMR, 400 MHz, CDCl3
O
OH
Me
OH
C-59
f1 (ppm)

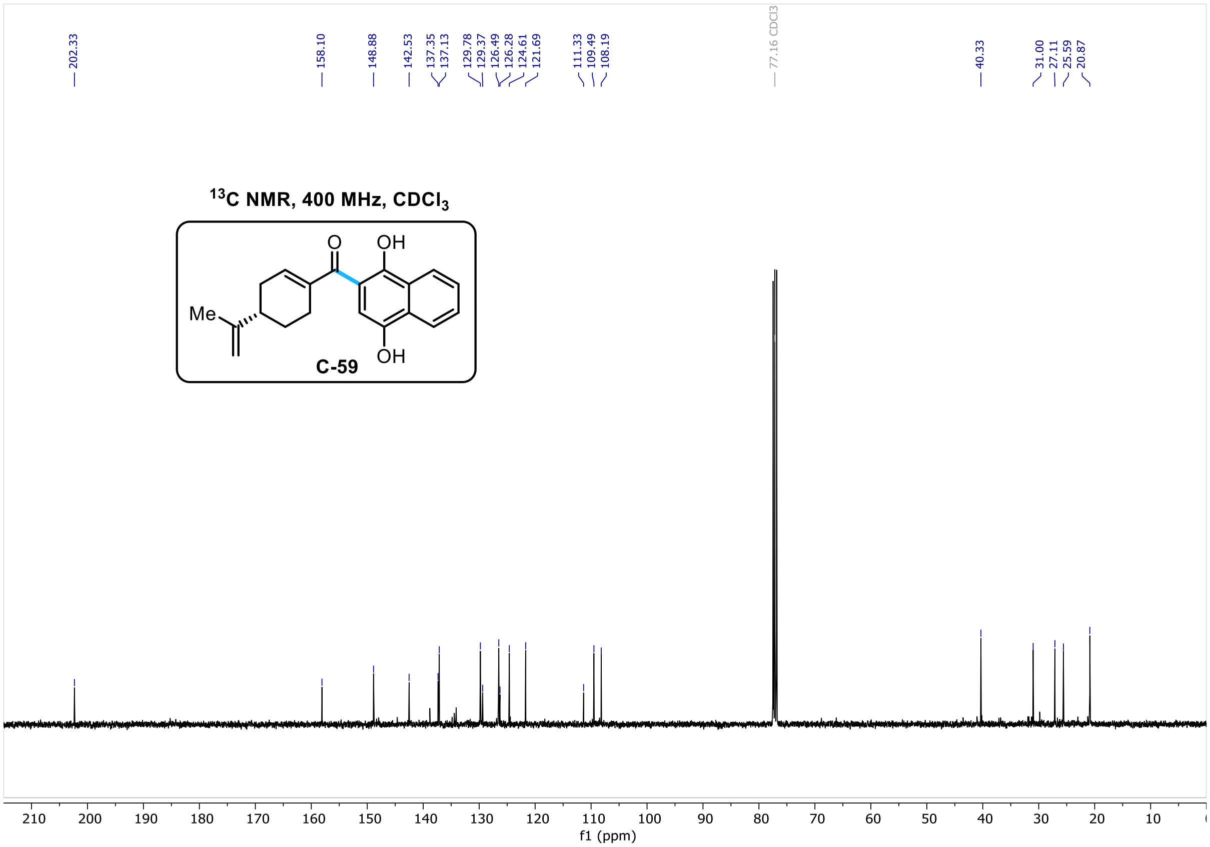
13C NMR, 400 MHz, CDCl3
O
OH
Me
OH
C-59
f1 (ppm)

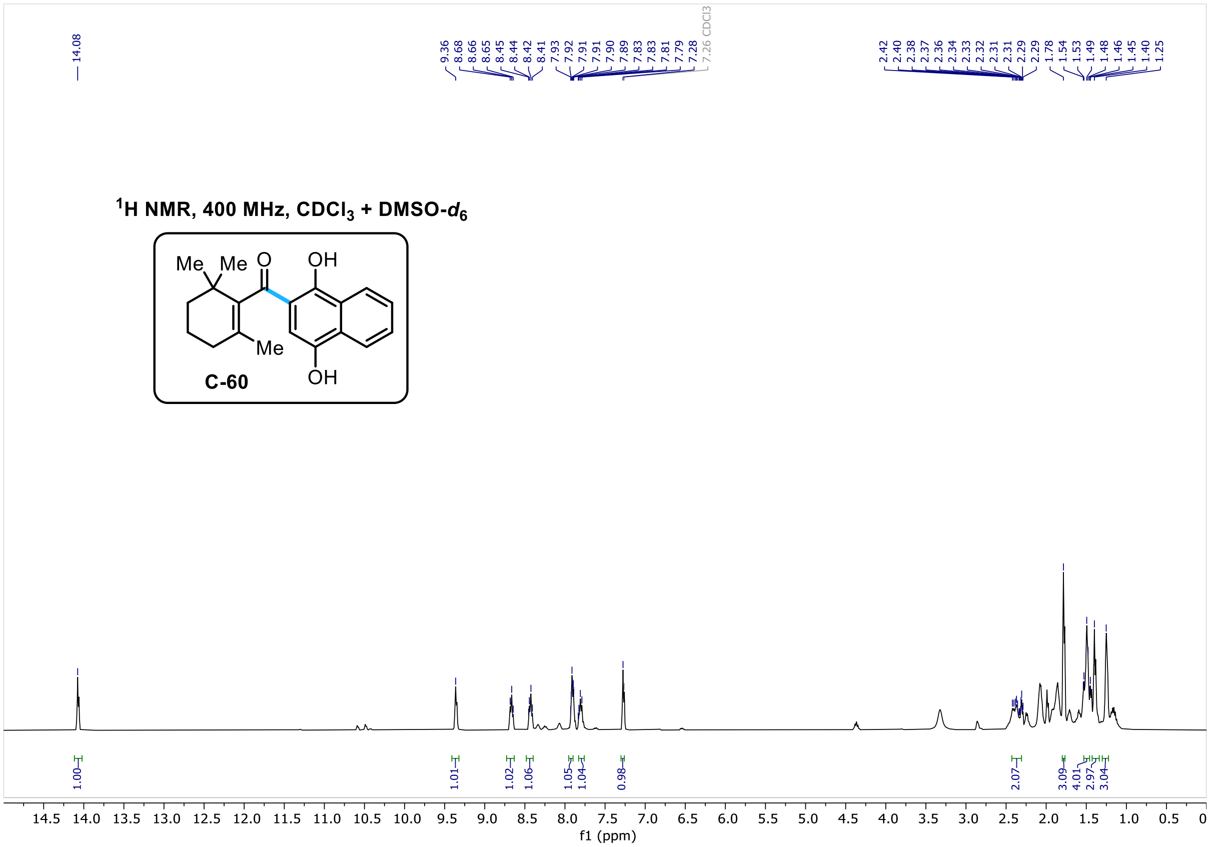
14.08
9.36
8.68
8.66
8.65
8.45
8.44
8.42
8.41
7.93
7.92
7.91
7.91
7.90
7.89
7.83
7.83
7.81
7.79
7.28
7.26 CDCl3
2.42
2.40
2.38
2.37
2.36
2.34
2.33
2.32
2.31
2.31
2.29
2.29
1.78
1.54
1.53
1.49
1.48
1.46
1.45
1.40
1.25
^{1}H NMR, 400 MHz, CDCl$_3$ + DMSO-d_6
Me
Me
O
OH
Me
OH
C-60
1.00
1.01
1.02
1.06
1.05
1.04
0.98
2.07
3.09
4.01
2.97
3.04
f1 (ppm)

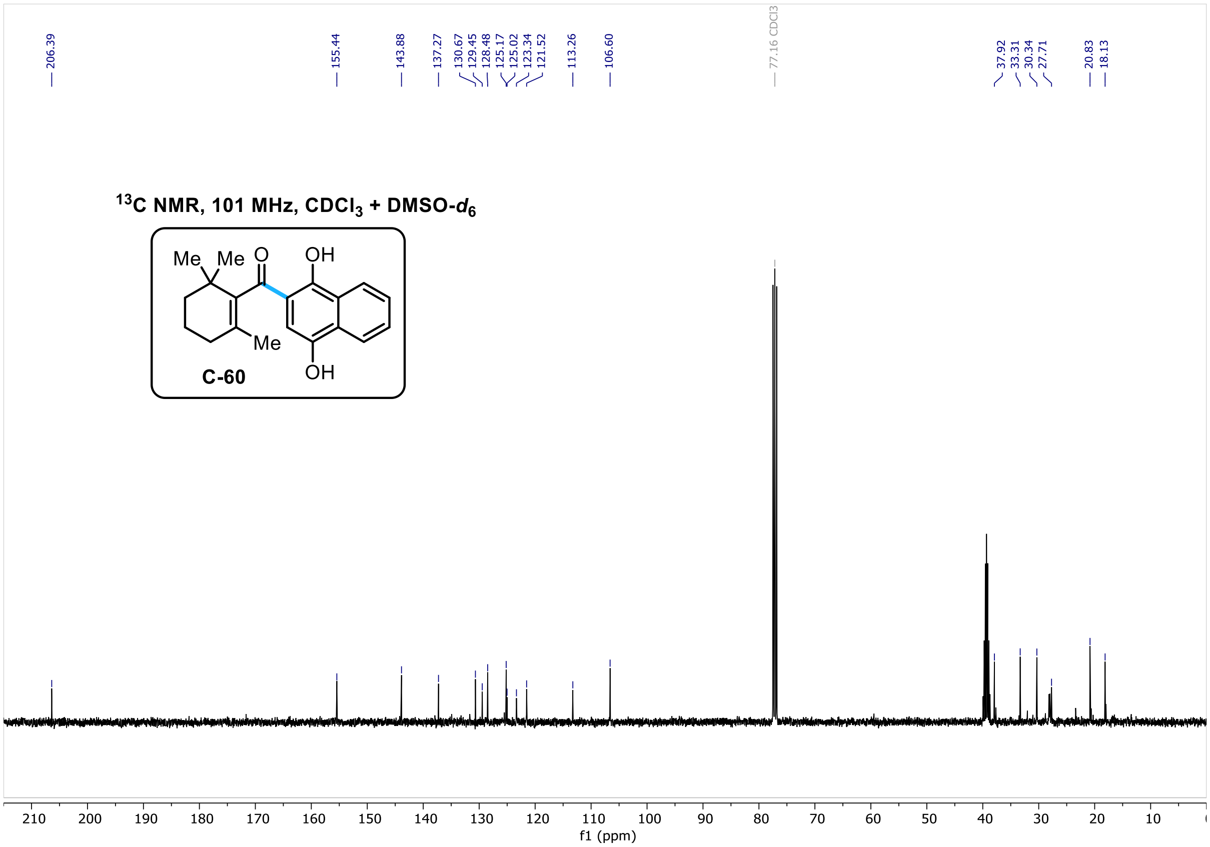
206.39
155.44
143.88
137.27
130.67
129.45
128.48
125.17
125.02
123.34
121.52
113.26
106.60
77.16 CDCl3
37.92
33.31
30.34
27.71
20.83
18.13
^{13}C NMR, 101 MHz, CDCl$_3$ + DMSO-d_6
Me
Me
O
OH
Me
OH
C-60
f1 (ppm)

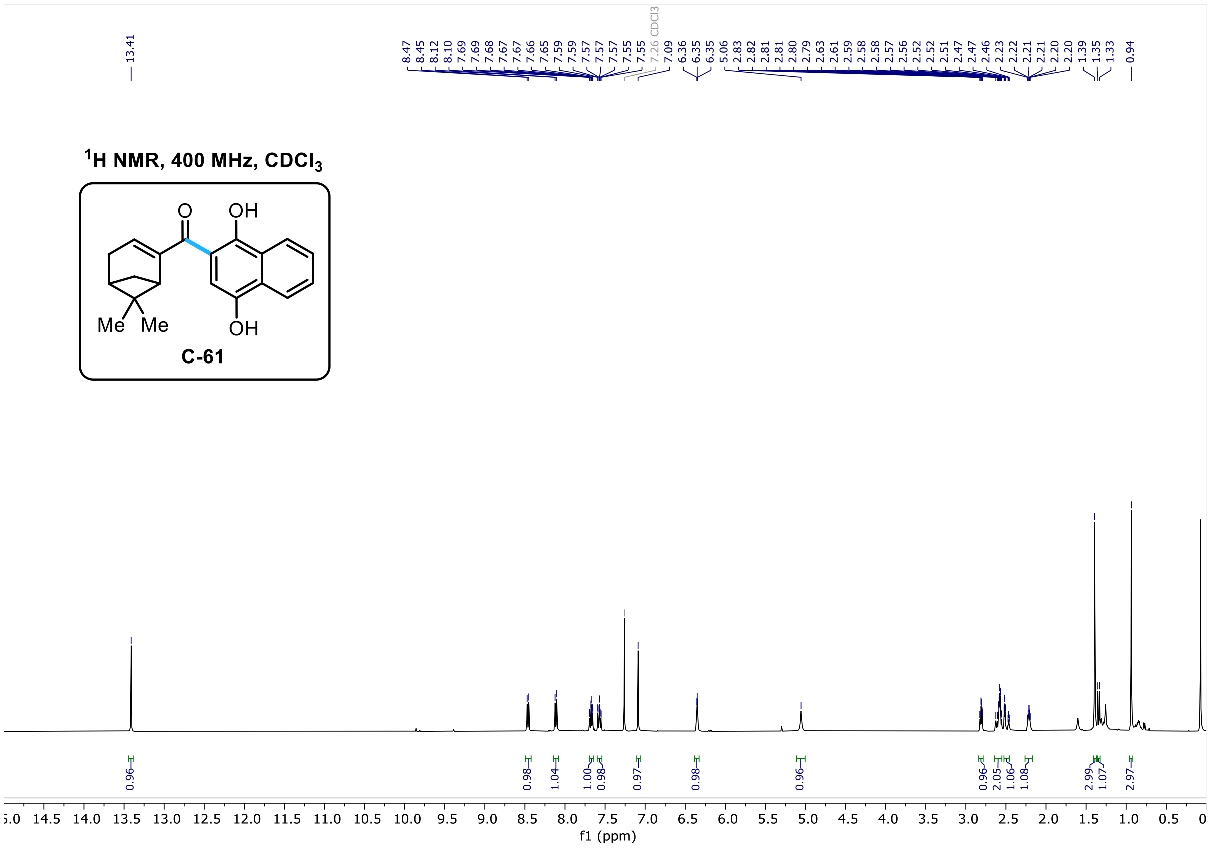

13.41
8.47 8.45 8.12 8.10 7.69 7.69 7.68 7.67 7.67 7.66 7.65 7.59 7.59 7.57 7.57 7.57 7.55 7.55
7.26 CDCl3
7.09 6.36 6.35 6.35 5.06 2.83 2.82 2.81 2.81 2.80 2.79 2.63 2.61 2.59 2.58 2.58 2.57 2.56 2.52 2.52 2.51 2.47 2.47 2.46 2.23 2.22 2.21 2.21 2.20 1.39 1.35 1.33 0.94
1H NMR, 400 MHz, CDCl3
O
OH
OH
Me
Me
C-61
0.96
0.98
1.04
1.00
0.98
0.97
0.98
0.96
0.96
2.05
1.06
1.08
2.99
1.07
2.97
f1 (ppm)

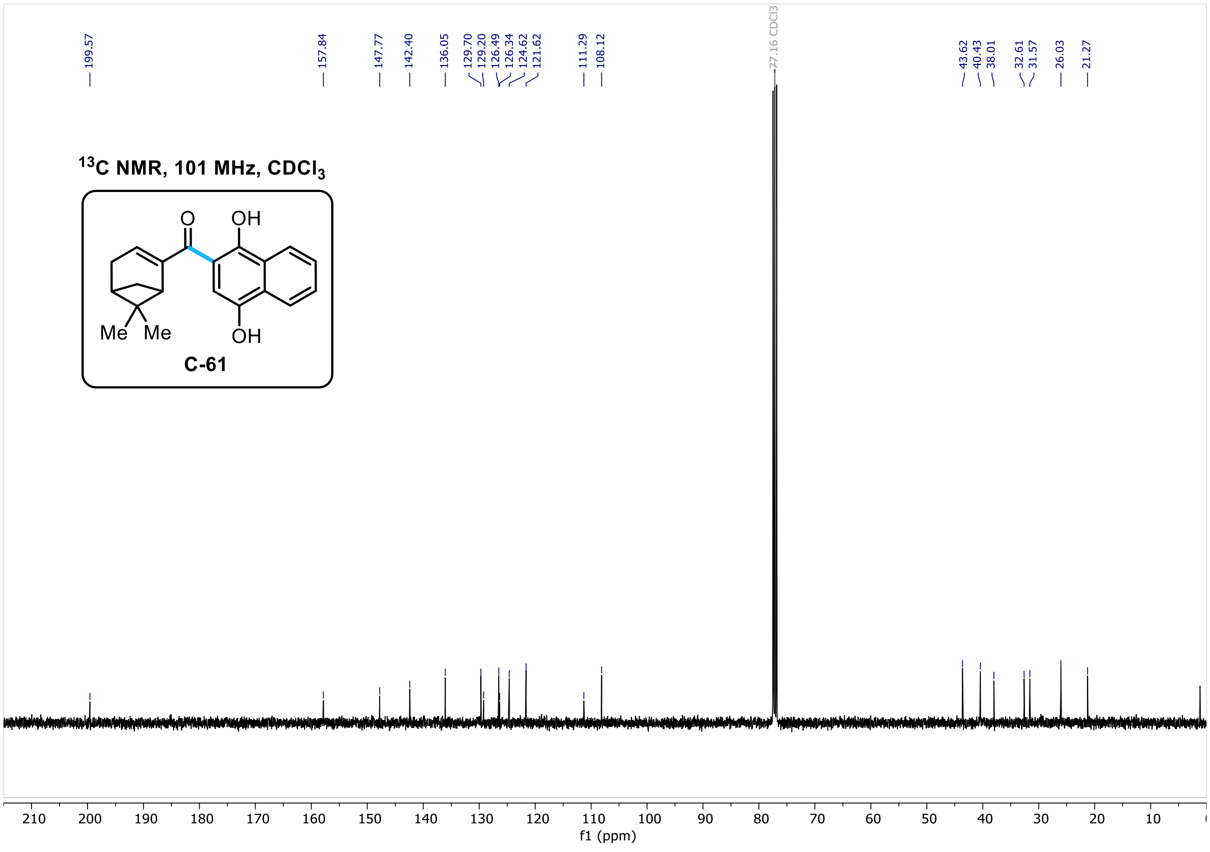

199.57
157.84
147.77
142.40
136.05
129.70 129.20 126.49 126.34 124.62 121.62
111.29
108.12
77.16 CDCl3
43.62 40.43 38.01
32.61 31.57
26.03
21.27
13C NMR, 101 MHz, CDCl3
O
OH
OH
Me
Me
C-61
f1 (ppm)

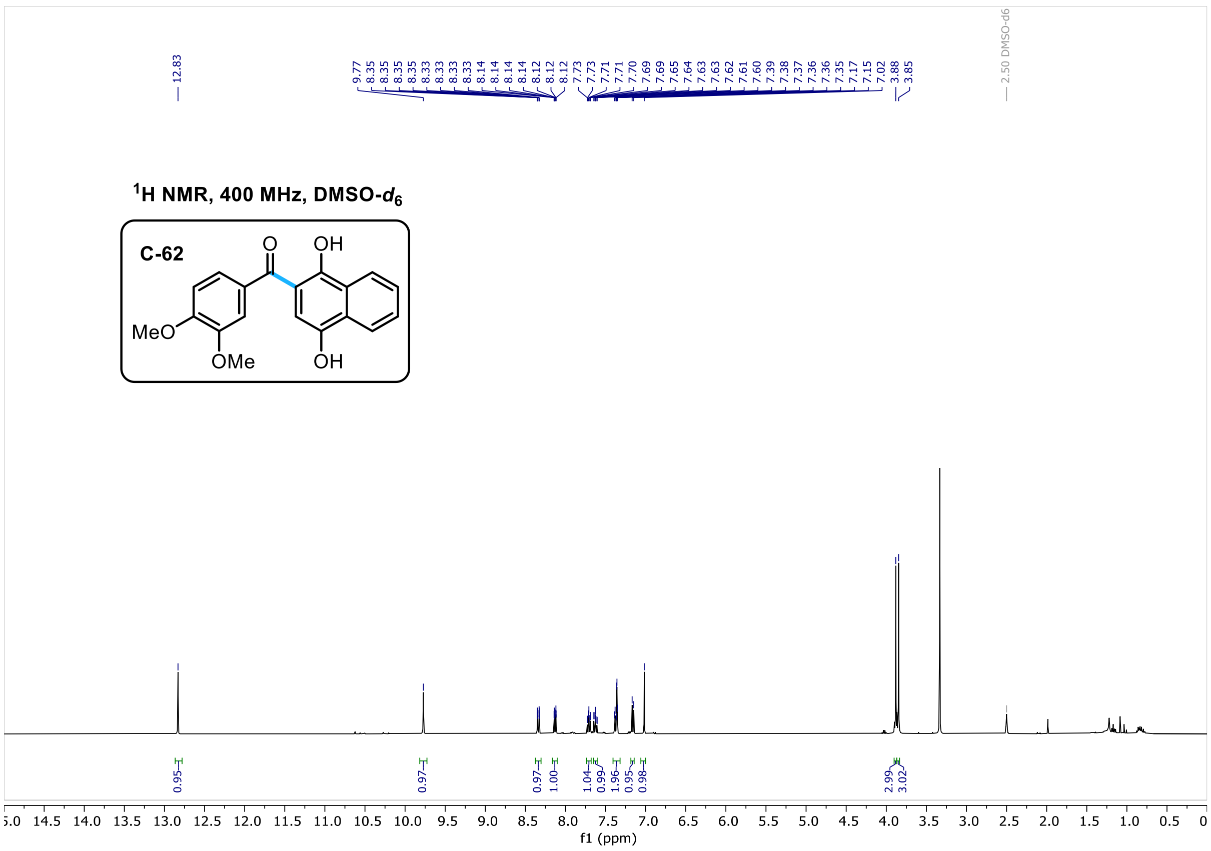

¹H NMR, 400 MHz, DMSO-d6
C-62
O
OH
MeO
OMe
OH
12.83
9.77
8.35
8.33
8.14
8.12
7.73
7.71
7.70
7.69
7.65
7.64
7.63
7.62
7.61
7.60
7.39
7.38
7.37
7.36
7.17
7.15
7.02
3.88
3.85
2.50 DMSO-d6
0.95
0.97
0.97
1.00
1.04
0.99
1.96
0.95
0.98
2.99
3.02
f1 (ppm)

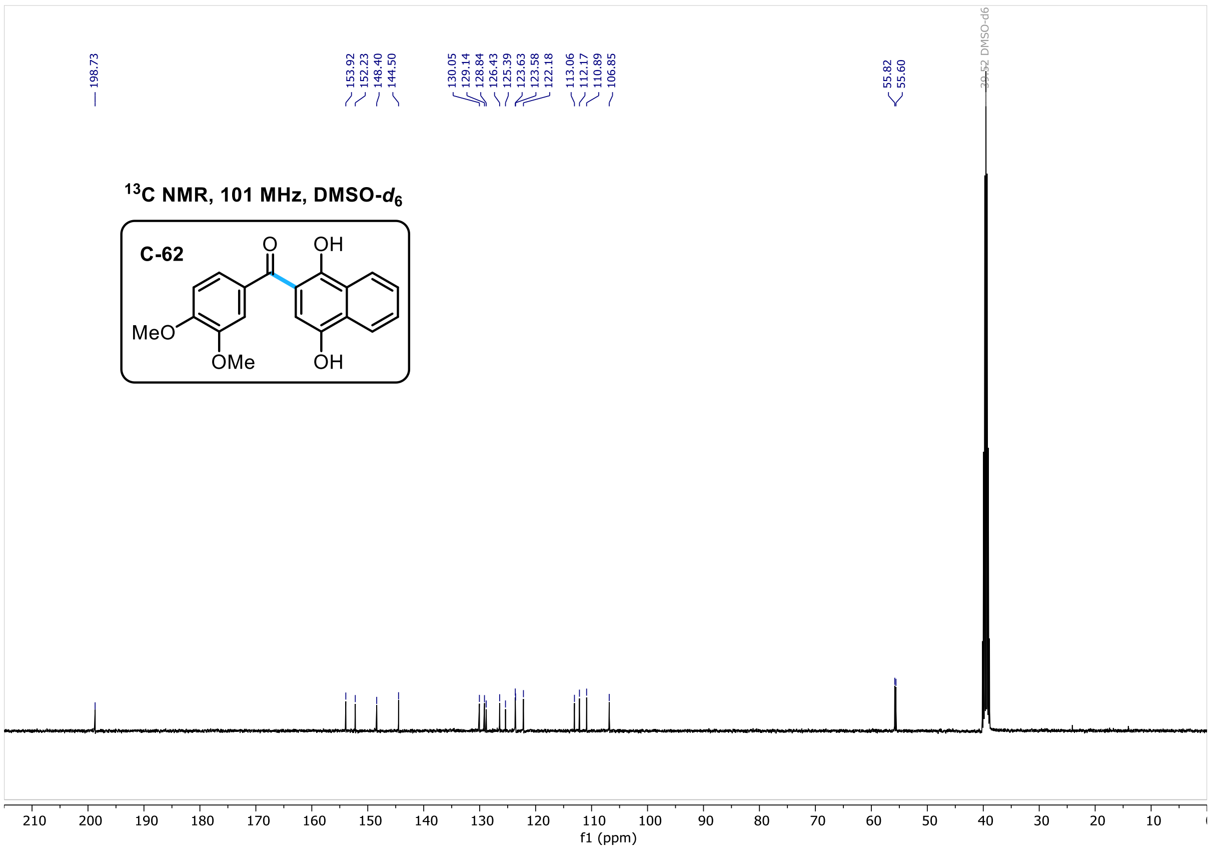

¹³C NMR, 101 MHz, DMSO-d6
C-62
O
OH
MeO
OMe
OH
198.73
153.92
152.23
148.40
144.50
130.05
129.14
128.84
126.43
125.39
123.63
123.58
122.18
113.06
112.17
110.89
106.85
55.82
55.60
39.52 DMSO-d6
f1 (ppm)

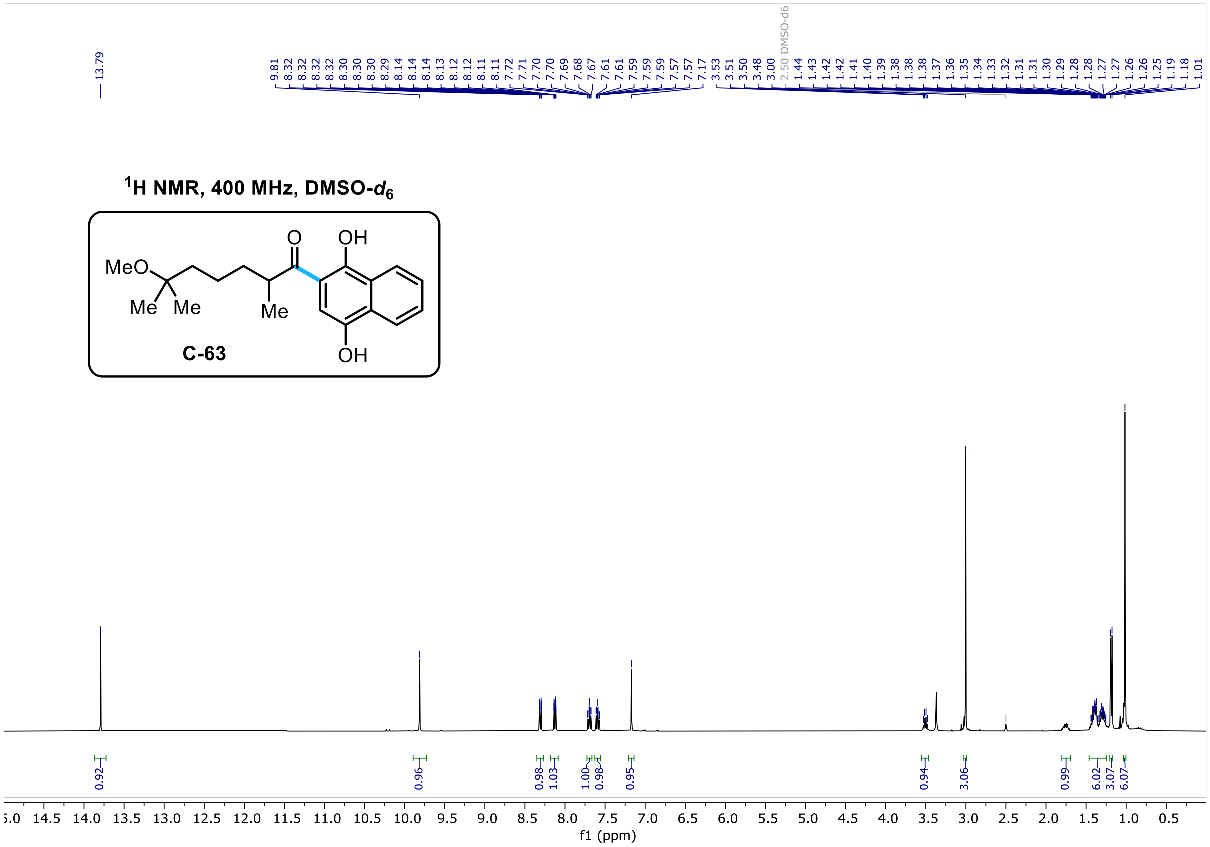

^{1}H NMR, 400 MHz, DMSO-d_6
MeO
Me
Me
Me
O
OH
OH
C-63
f1 (ppm)

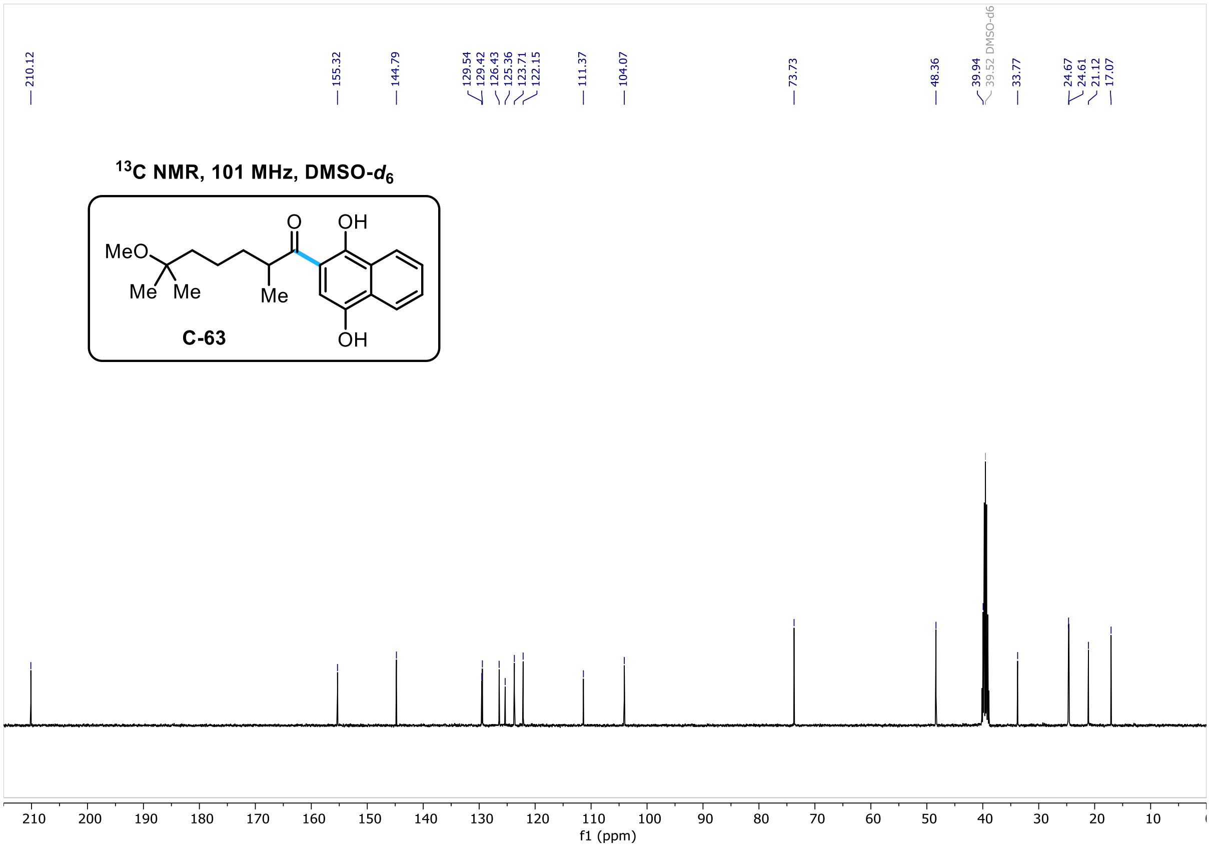

^{13}C NMR, 101 MHz, DMSO-d_6
MeO
Me
Me
Me
O
OH
OH
C-63
210.12
155.32
144.79
129.54
129.42
126.43
125.36
123.71
122.15
111.37
104.07
73.73
48.36
39.94
39.52 DMSO-d6
33.77
24.67
24.61
21.12
17.07
f1 (ppm)

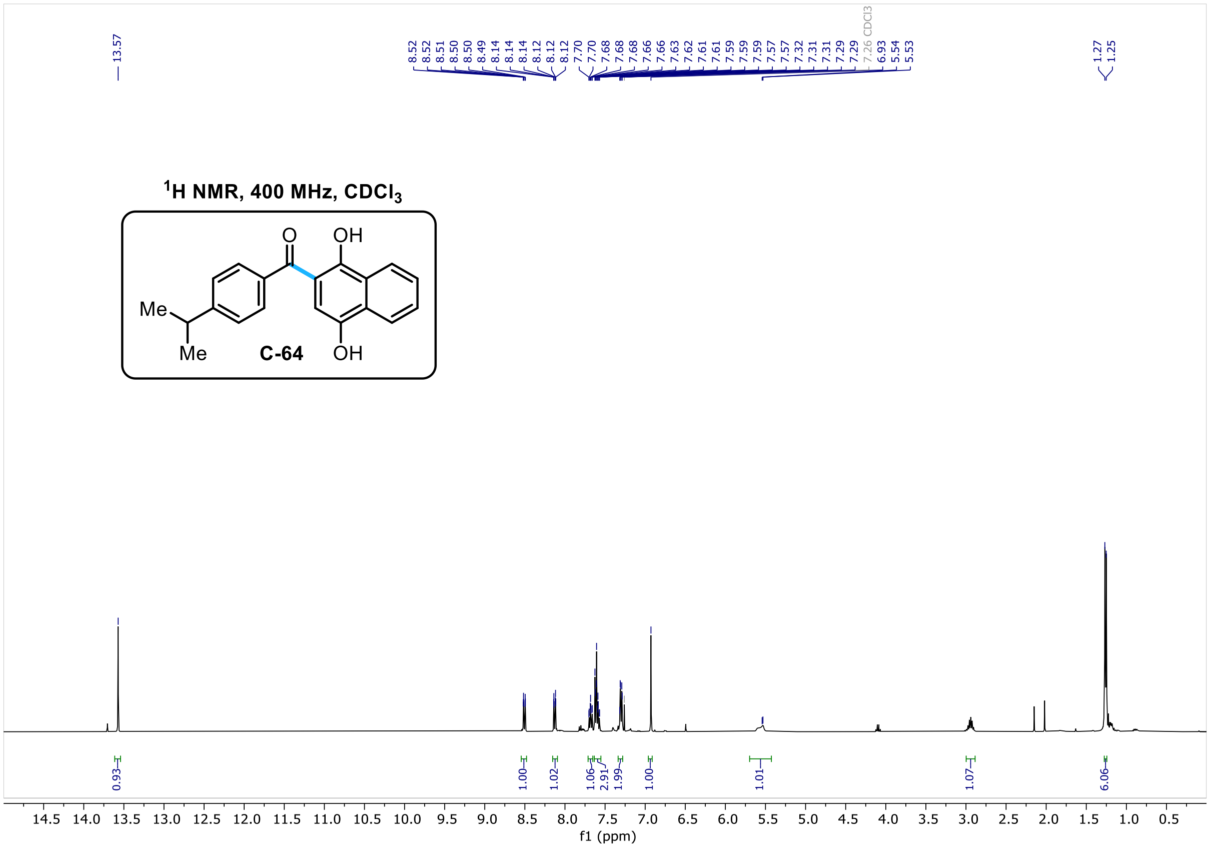
13.57
8.52 8.52 8.51 8.50 8.50 8.49 8.14 8.14 8.14 8.12 8.12 8.12 7.70 7.70 7.68 7.68 7.68 7.66 7.63 7.62 7.61 7.61 7.59 7.59 7.59 7.57 7.57 7.32 7.31 7.31 7.29 7.29 7.26 CDCl3 6.93 5.54 5.53
1.27 1.25
1H NMR, 400 MHz, CDCl3
O
OH
Me
Me
C-64
OH
0.93
1.00
1.02
1.06
2.91
1.99
1.00
1.01
1.07
6.06
14.5 14.0 13.5 13.0 12.5 12.0 11.5 11.0 10.5 10.0 9.5 9.0 8.5 8.0 7.5 7.0 6.5 6.0 5.5 5.0 4.5 4.0 3.5 3.0 2.5 2.0 1.5 1.0 0.5
f1 (ppm)

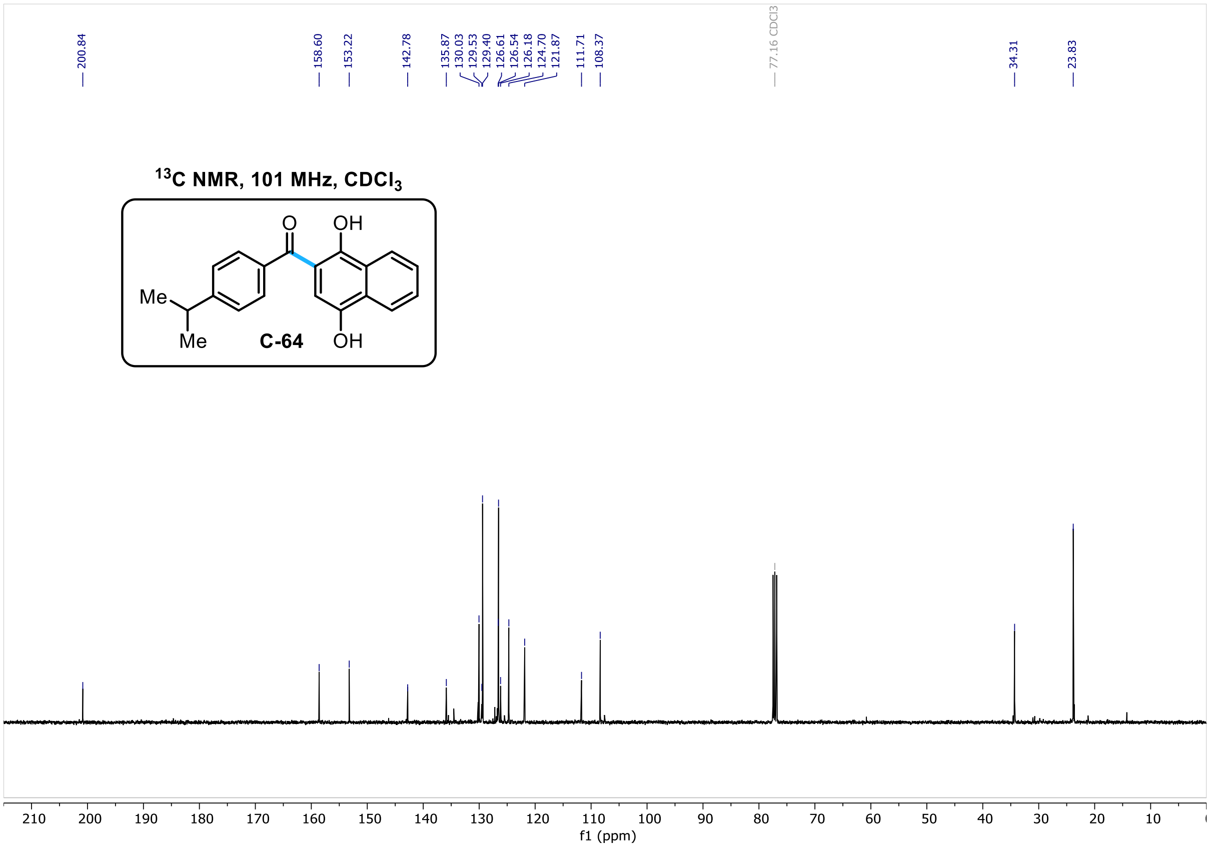
200.84
158.60
153.22
142.78
135.87 130.03 129.53 129.40 126.61 126.54 126.18 124.70 121.87
111.71
108.37
77.16 CDCl3
34.31
23.83
13C NMR, 101 MHz, CDCl3
O
OH
Me
Me
C-64
OH
210 200 190 180 170 160 150 140 130 120 110 100 90 80 70 60 50 40 30 20 10
f1 (ppm)

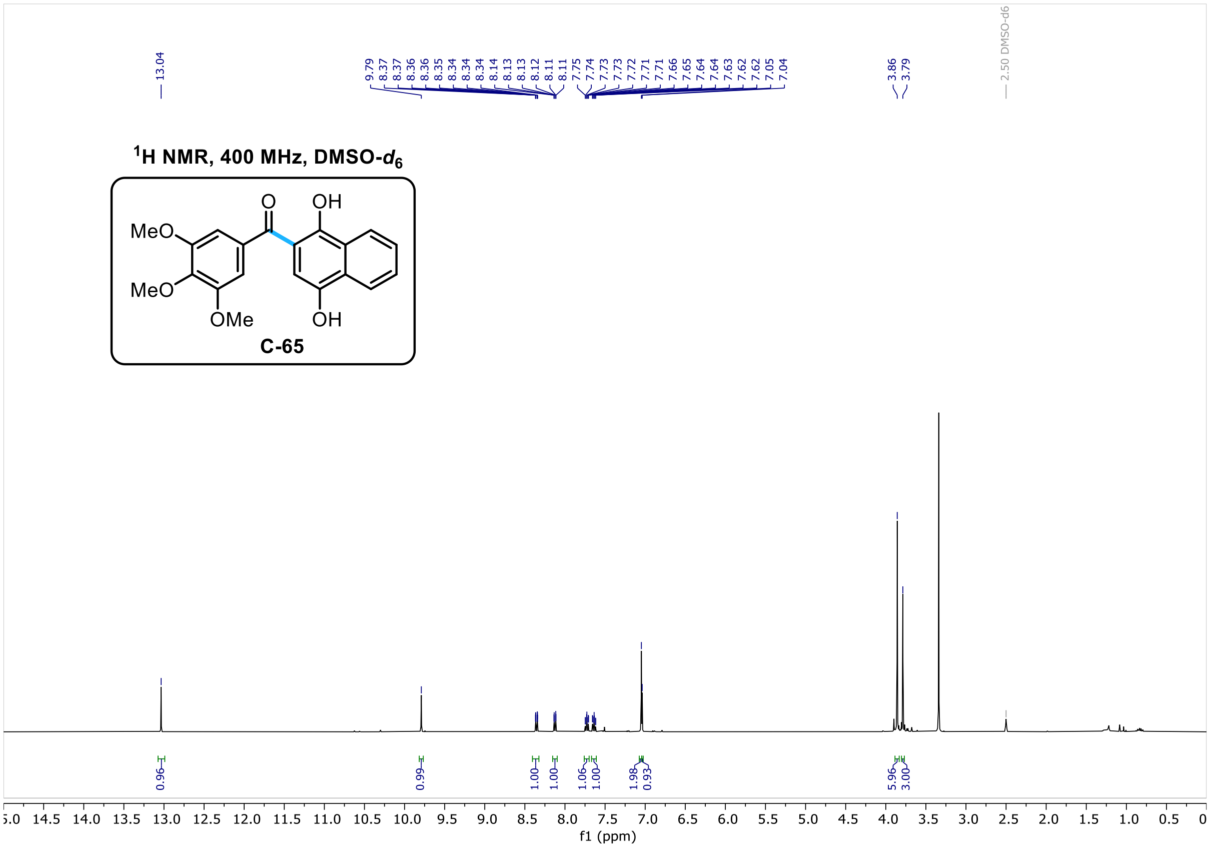

^{1}H NMR, 400 MHz, DMSO-d_6
MeO
MeO
OMe
O
OH
OH
C-65
13.04
9.79 8.37 8.37 8.36 8.36 8.35 8.34 8.34 8.34 8.14 8.13 8.13 8.12 8.11 8.11 7.75 7.74 7.73 7.73 7.72 7.71 7.71 7.66 7.65 7.64 7.64 7.63 7.62 7.62 7.05 7.04
3.86 3.79
2.50 DMSO-d6
0.96 0.99 1.00 1.00 1.06 1.00 1.98 0.93 5.96 3.00
f1 (ppm)

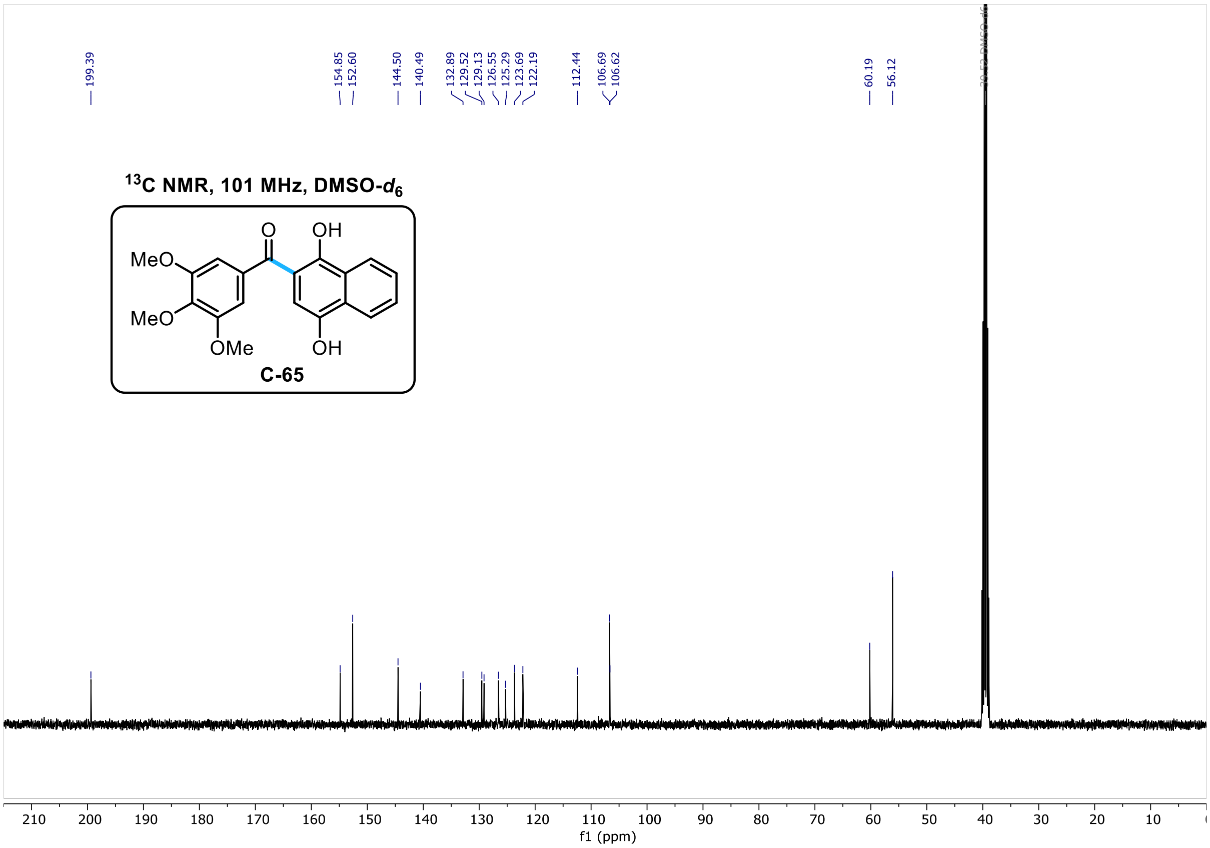

^{13}C NMR, 101 MHz, DMSO-d_6
MeO
MeO
OMe
O
OH
OH
C-65
199.39
154.85 152.60
144.50 140.49
132.89 129.52 129.13 126.55 125.29 123.69 122.19
112.44 106.69 106.62
60.19 56.12
f1 (ppm)

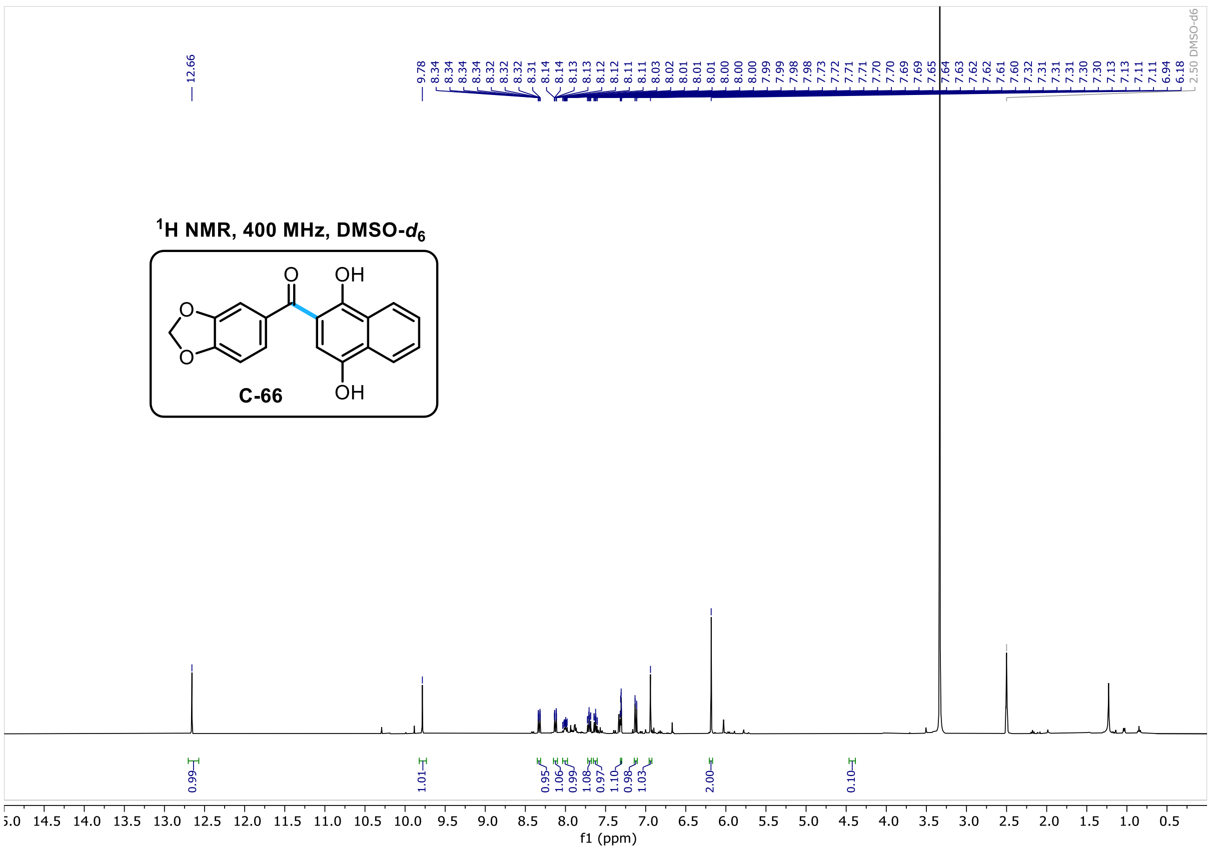

¹H NMR, 400 MHz, DMSO-d6
C-66
f1 (ppm)

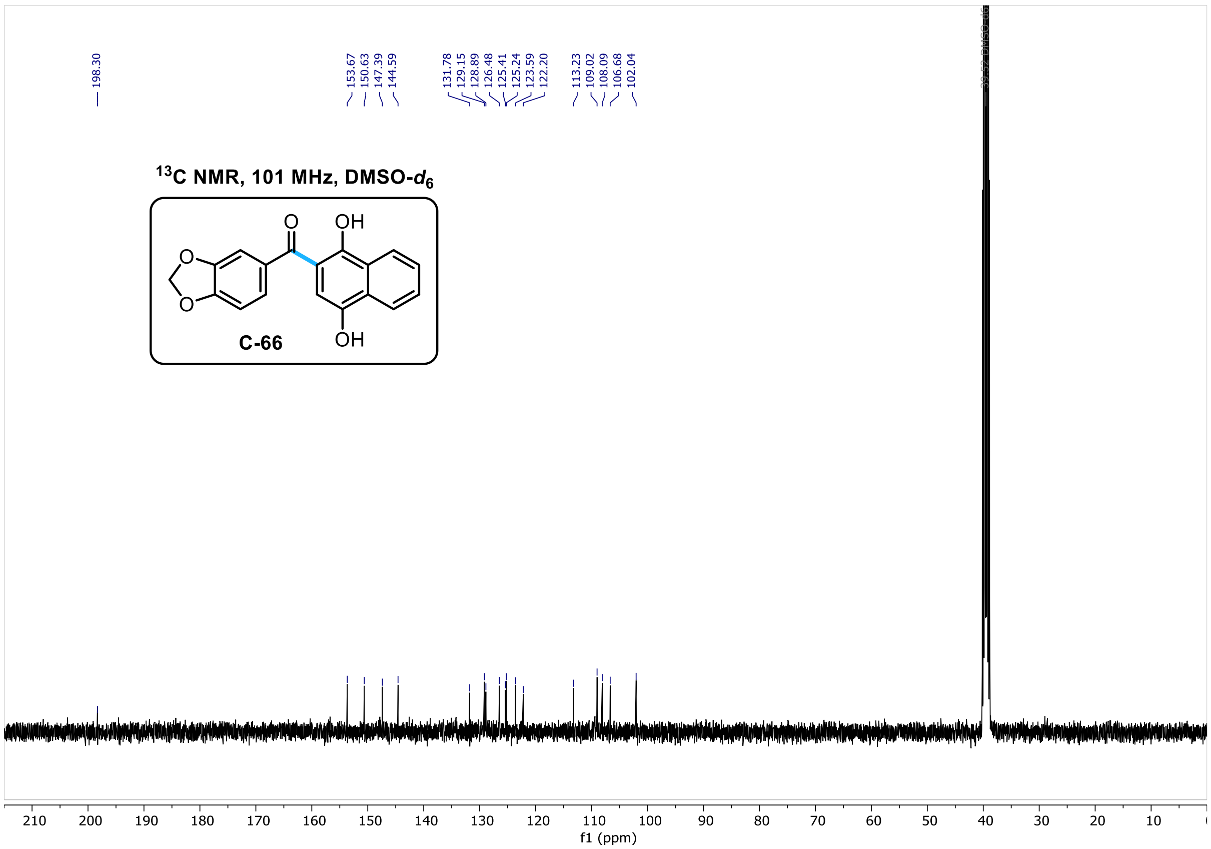

¹³C NMR, 101 MHz, DMSO-d6
C-66
198.30
153.67
150.63
147.39
144.59
131.78
129.15
128.89
126.48
125.41
125.24
123.59
122.20
113.23
109.02
108.09
106.68
102.04
f1 (ppm)

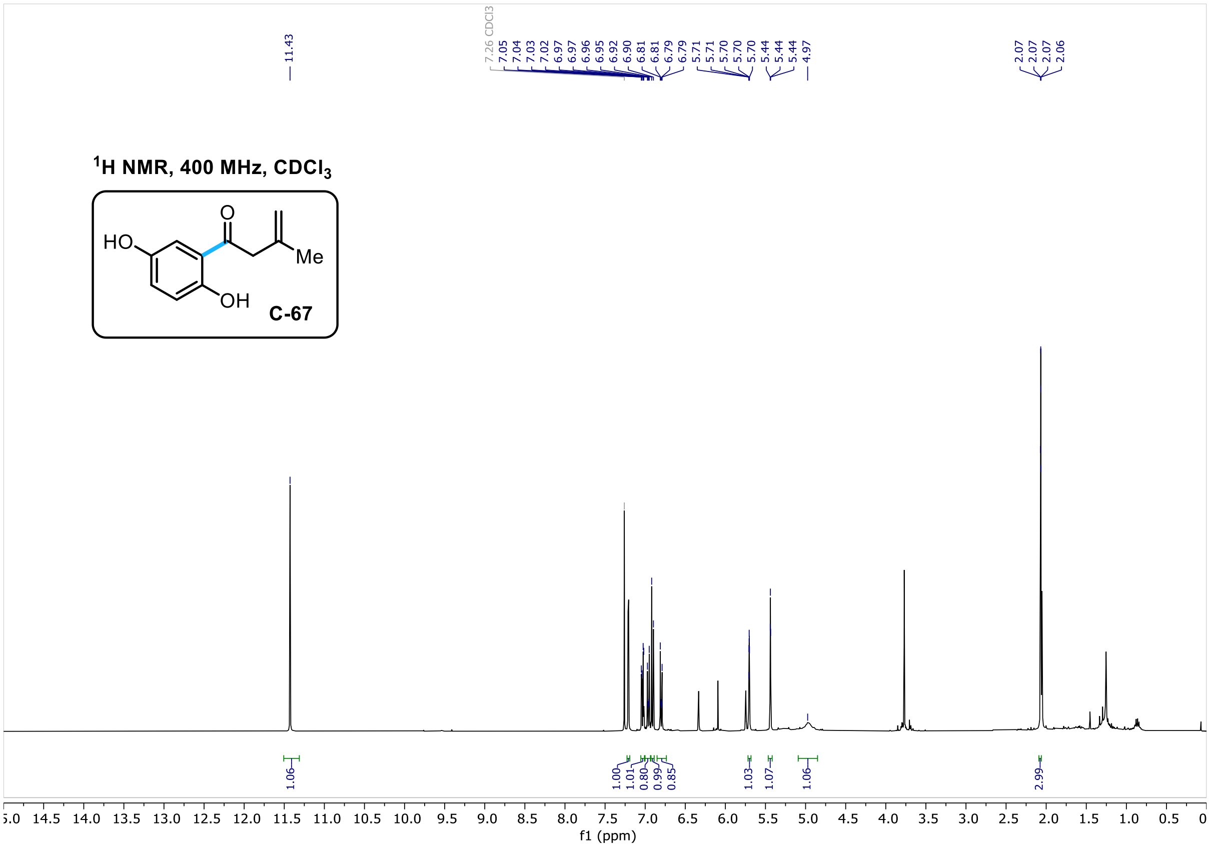
1H NMR, 400 MHz, CDCl3
HO
O
Me
OH
C-67
11.43
7.26 CDCl3
7.05
7.04
7.03
7.02
6.97
6.97
6.96
6.95
6.92
6.90
6.81
6.81
6.79
6.79
5.71
5.71
5.70
5.70
5.44
5.44
5.44
4.97
2.07
2.07
2.07
2.06
1.06
1.00
1.01
0.80
0.99
0.85
1.03
1.07
1.06
2.99
f1 (ppm)

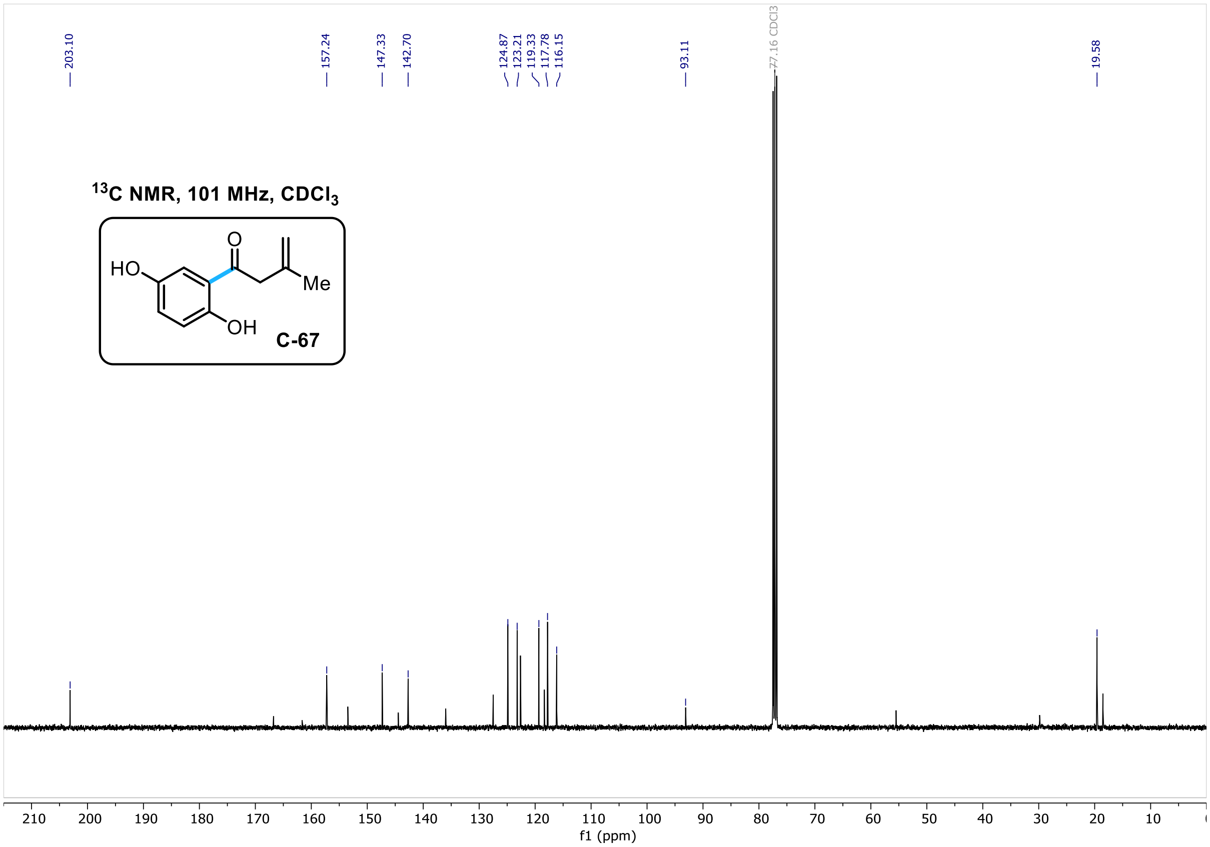
13C NMR, 101 MHz, CDCl3
HO
O
Me
OH
C-67
203.10
157.24
147.33
142.70
124.87
123.21
119.33
117.78
116.15
93.11
77.16 CDCl3
19.58
f1 (ppm)

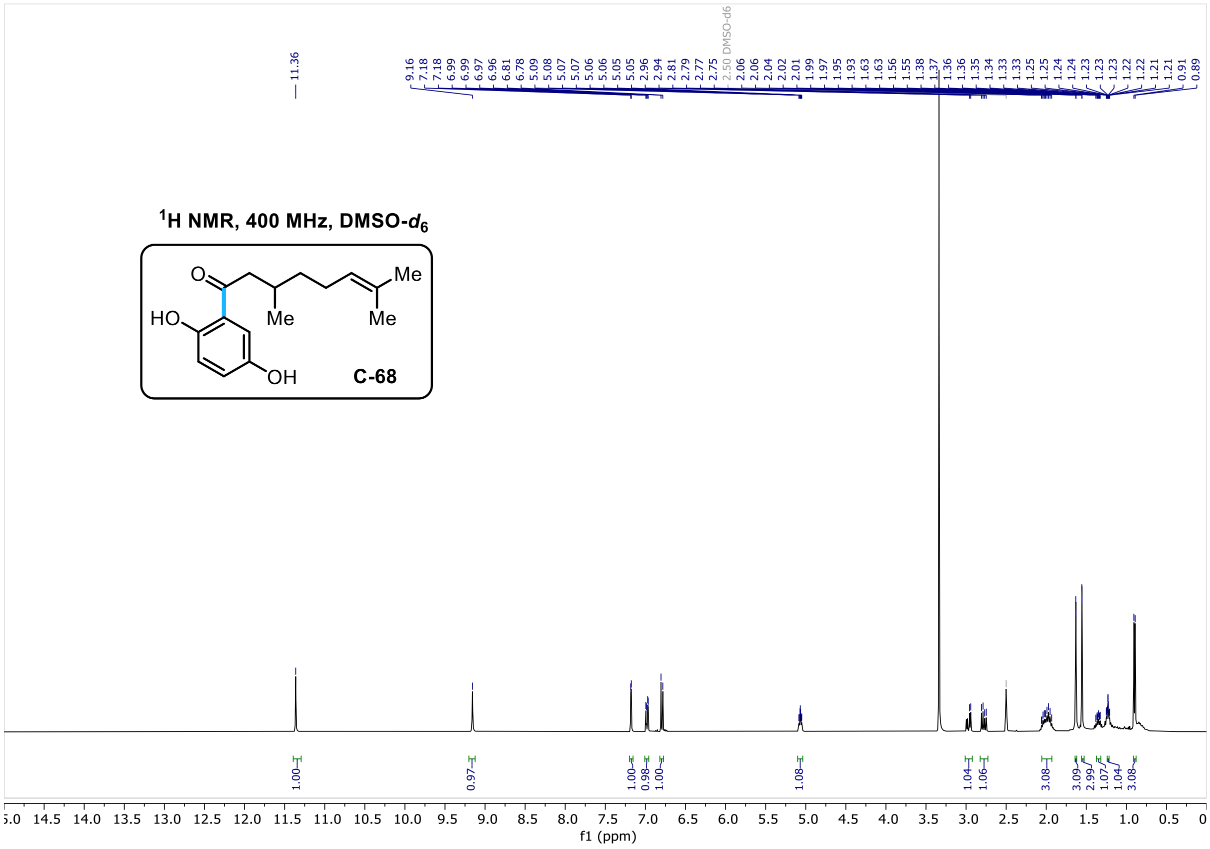

1H NMR, 400 MHz, DMSO-d6
C-68
f1 (ppm)

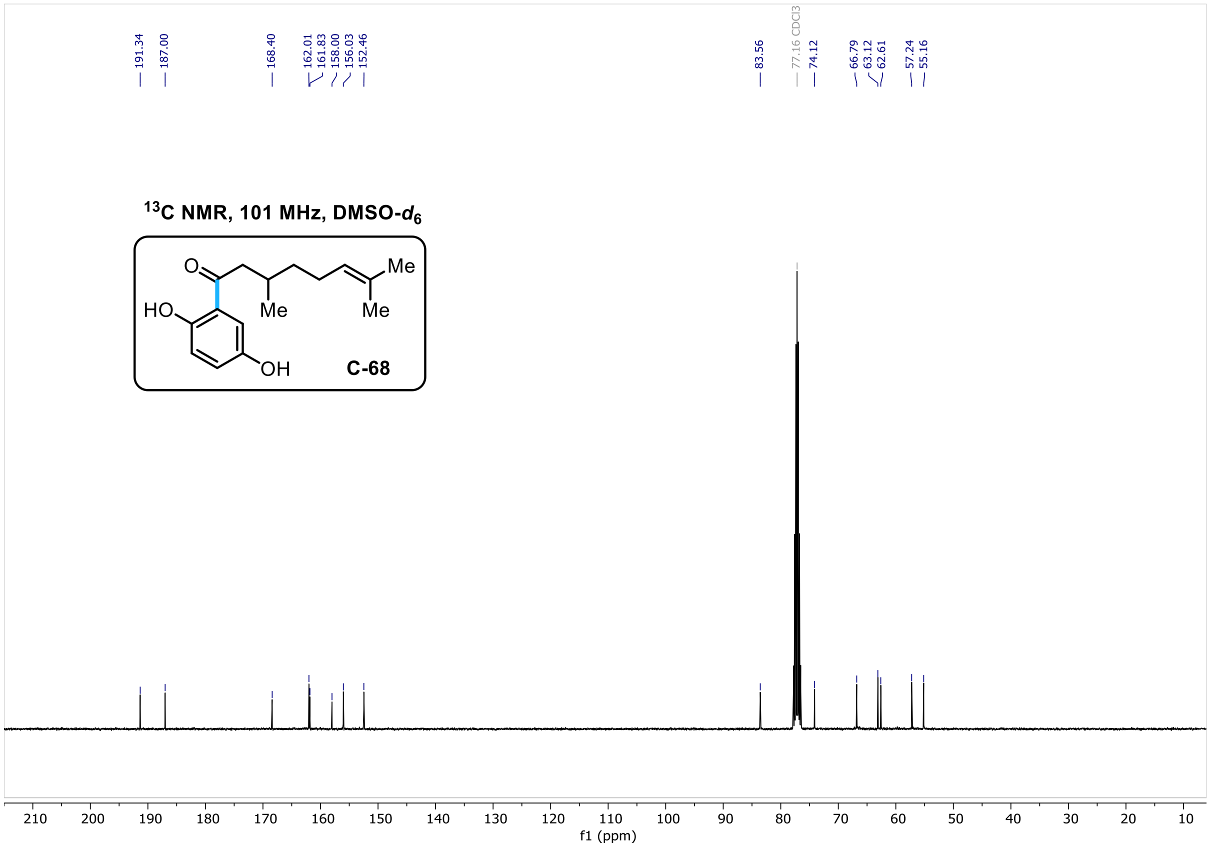

13C NMR, 101 MHz, DMSO-d6
C-68
f1 (ppm)

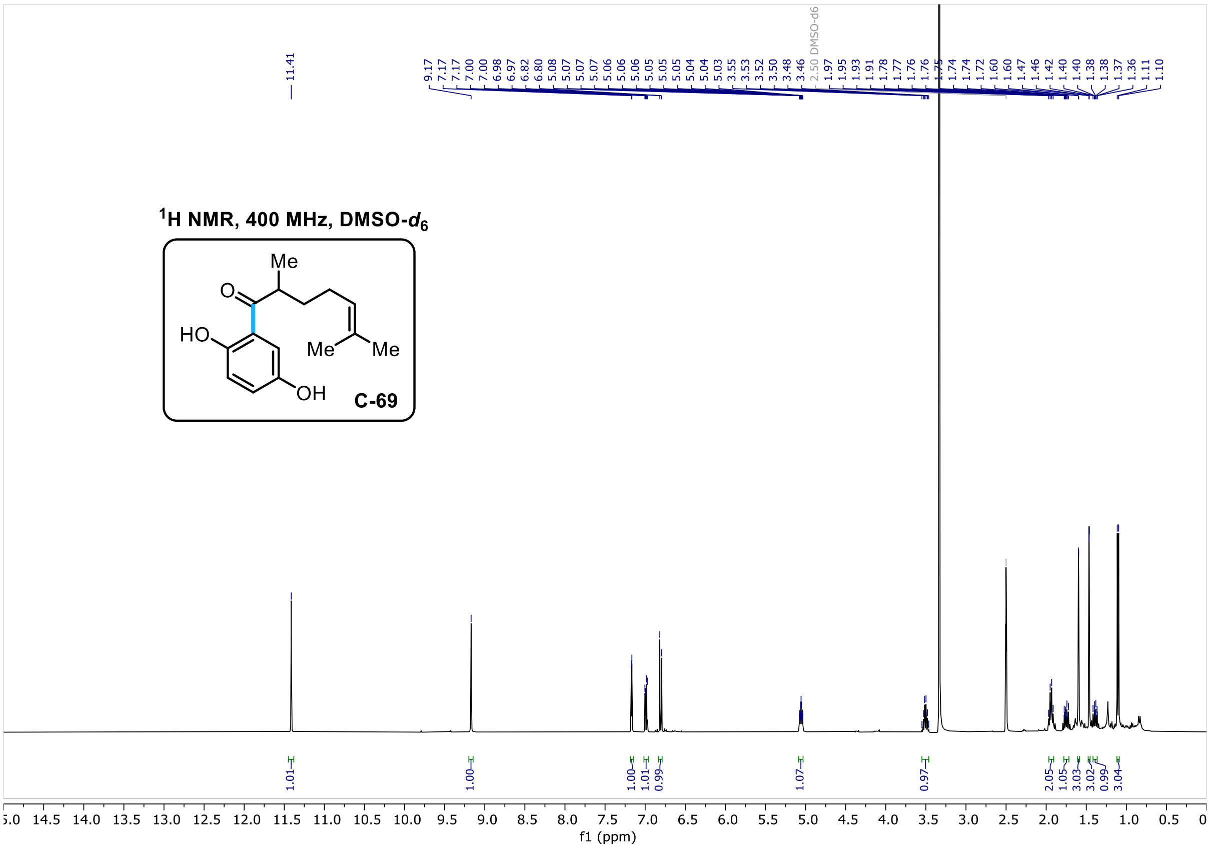

1H NMR, 400 MHz, DMSO-d6
Me
O
HO
Me
Me
OH
C-69
11.41
9.17
7.17
7.17
7.00
7.00
6.98
6.97
6.82
6.80
5.08
5.07
5.07
5.06
5.06
5.05
5.05
5.04
5.04
5.03
3.55
3.53
3.52
3.50
3.48
3.46
2.50 DMSO-d6
1.97
1.95
1.93
1.91
1.78
1.77
1.76
1.75
1.74
1.74
1.72
1.60
1.60
1.47
1.46
1.42
1.40
1.40
1.38
1.38
1.37
1.36
1.11
1.10
1.01
1.00
1.00
1.01
0.99
1.07
0.97
2.05
1.05
3.03
3.02
0.99
3.04
f1 (ppm)

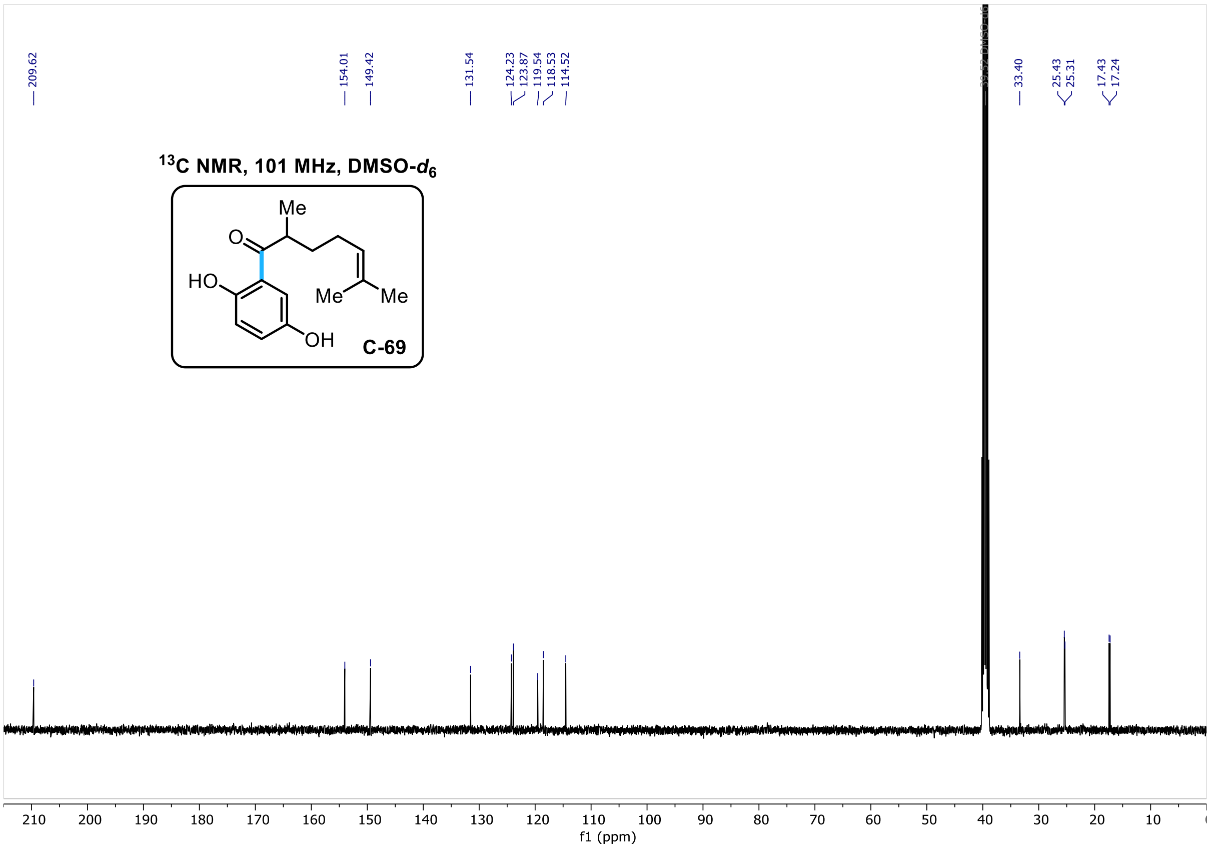

13C NMR, 101 MHz, DMSO-d6
Me
O
HO
Me
Me
OH
C-69
209.62
154.01
149.42
131.54
124.23
123.87
119.54
118.53
114.52
33.40
25.43
25.31
17.43
17.24
f1 (ppm)

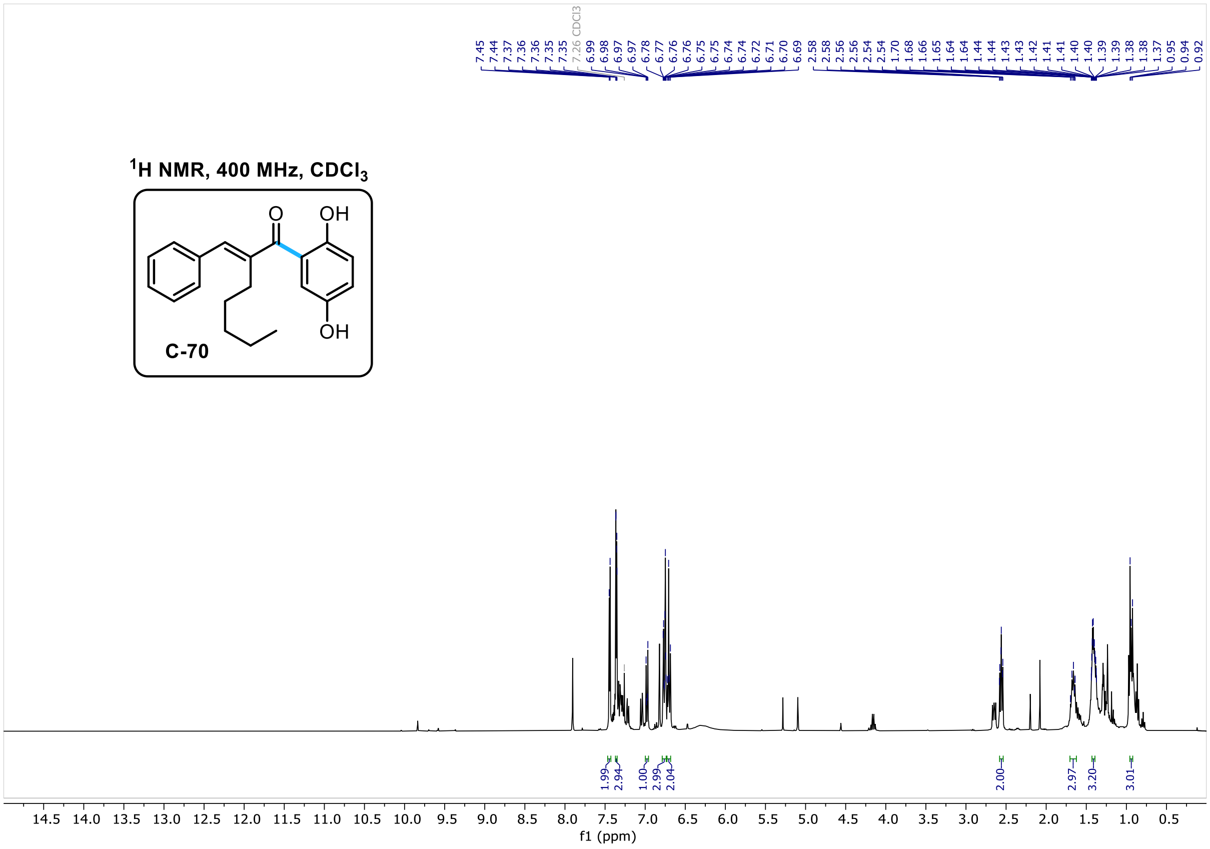
7.45 7.44 7.37 7.36 7.36 7.35 7.35 7.26 CDCl3 6.99 6.98 6.97 6.97 6.78 6.77 6.76 6.76 6.75 6.75 6.74 6.72 6.71 6.70 6.69 2.58 2.58 2.56 2.56 2.54 2.54 1.70 1.68 1.66 1.65 1.64 1.64 1.44 1.44 1.43 1.43 1.42 1.41 1.41 1.40 1.40 1.39 1.39 1.38 1.38 1.37 0.95 0.94 0.92
^{1}H NMR, 400 MHz, CDCl$_3$
O
OH
OH
C-70
1.99 2.94 1.00 2.99 2.04 2.00 2.97 3.20 3.01
f1 (ppm)

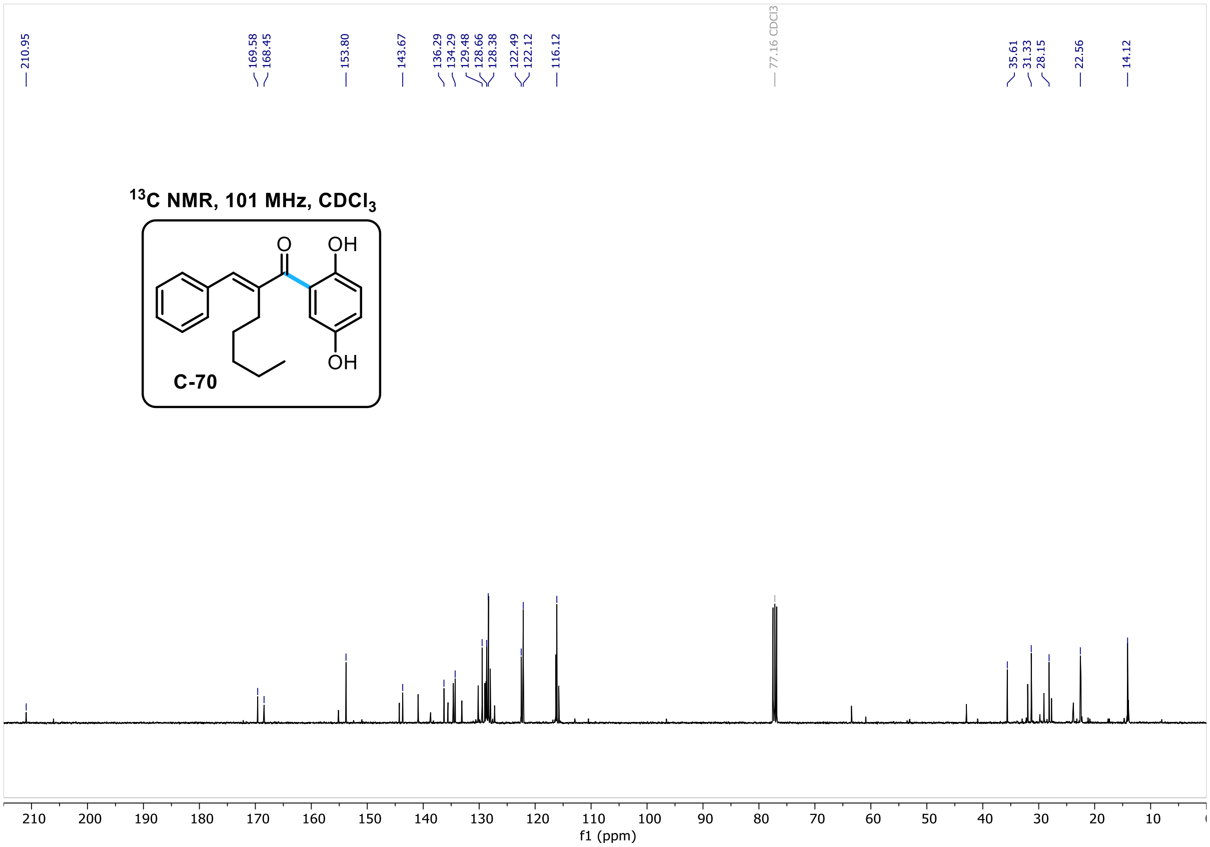
210.95 169.58 168.45 153.80 143.67 136.29 134.29 129.48 128.66 128.38 122.49 122.12 116.12 77.16 CDCl3 35.61 31.33 28.15 22.56 14.12
^{13}C NMR, 101 MHz, CDCl$_3$
O
OH
OH
C-70
f1 (ppm)

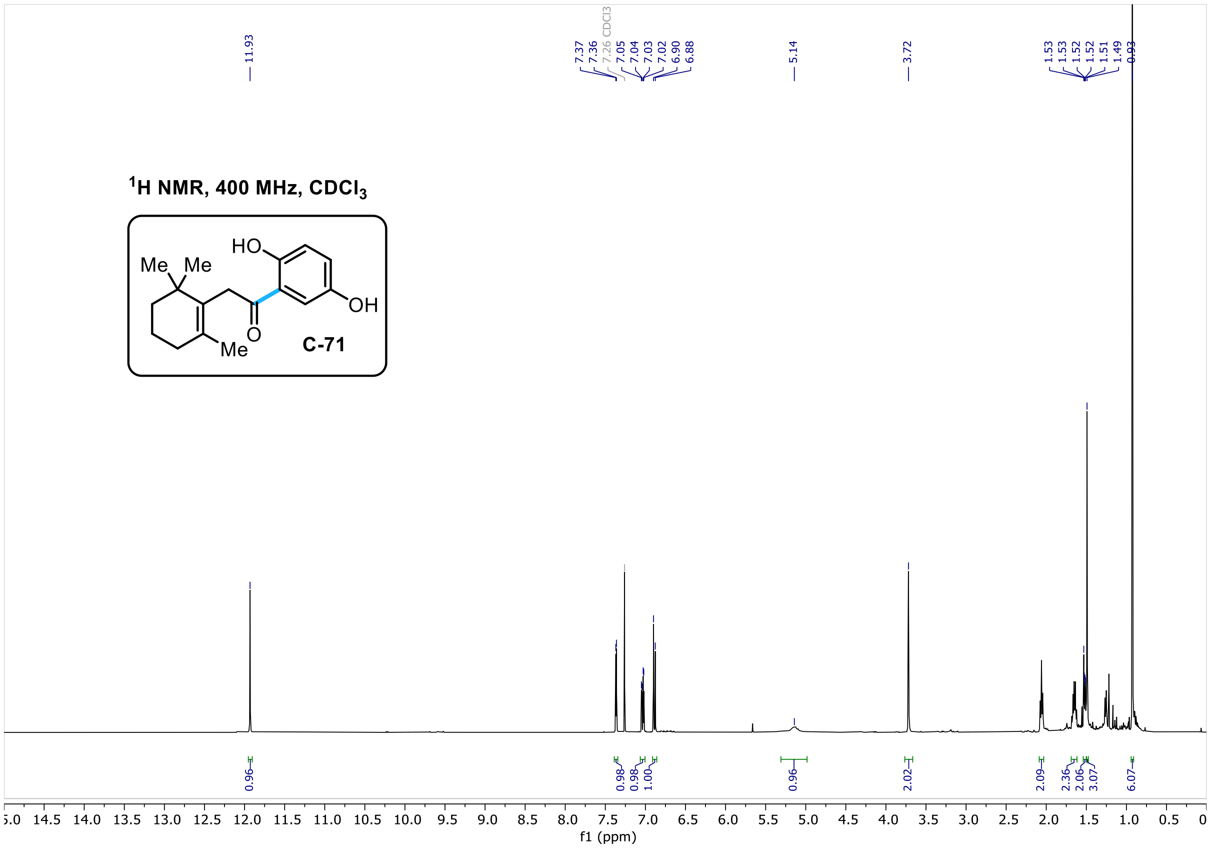

^{1}H NMR, 400 MHz, CDCl$_3$
HO
Me Me
OH
O
Me
C-71
11.93
7.37 7.36 7.26 CDCl3 7.05 7.04 7.03 7.02 6.90 6.88
5.14
3.72
1.53 1.53 1.52 1.52 1.51 1.49 0.93
0.96
0.98 0.98 1.00
0.96
2.02
2.09 2.36 2.06 3.07
6.07
f1 (ppm)

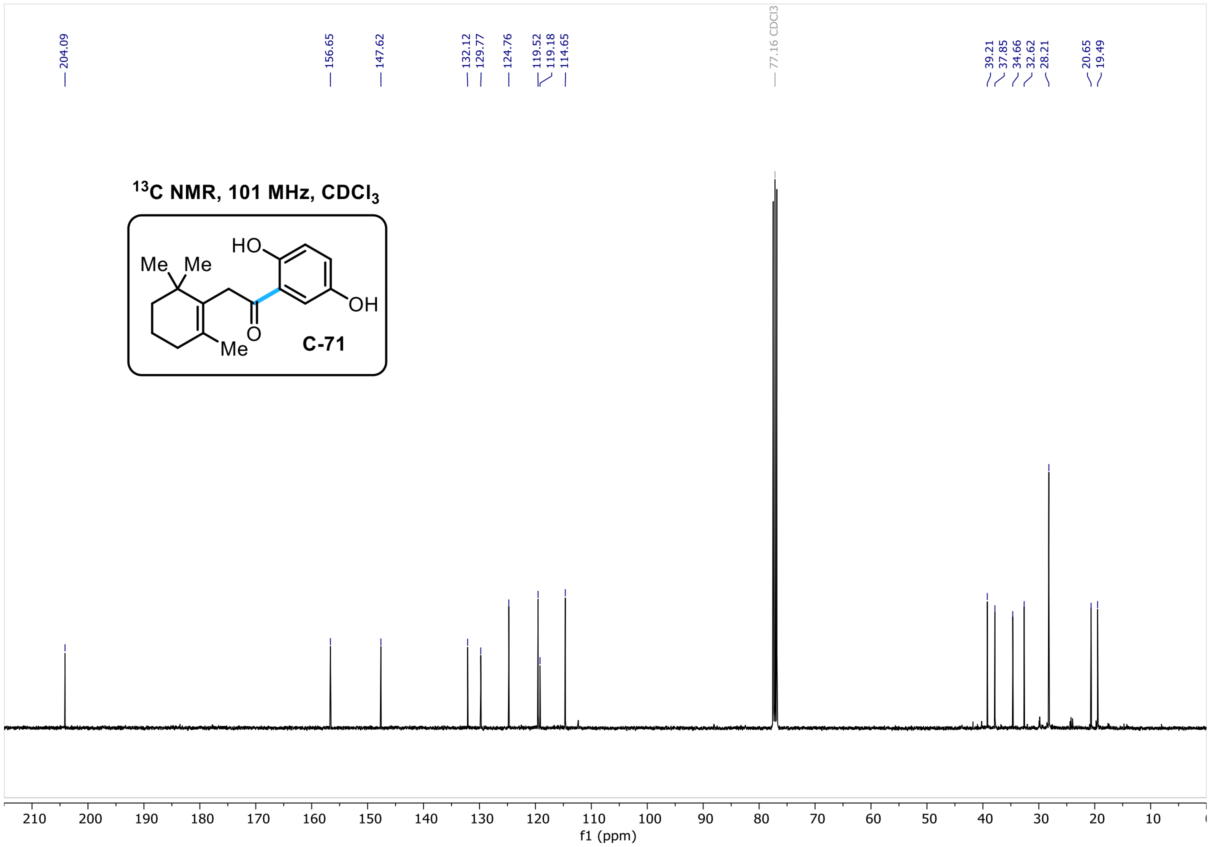

^{13}C NMR, 101 MHz, CDCl$_3$
HO
Me Me
OH
O
Me
C-71
204.09
156.65
147.62
132.12 129.77
124.76
119.52 119.18 114.65
77.16 CDCl3
39.21 37.85 34.66 32.62 28.21
20.65 19.49
f1 (ppm)

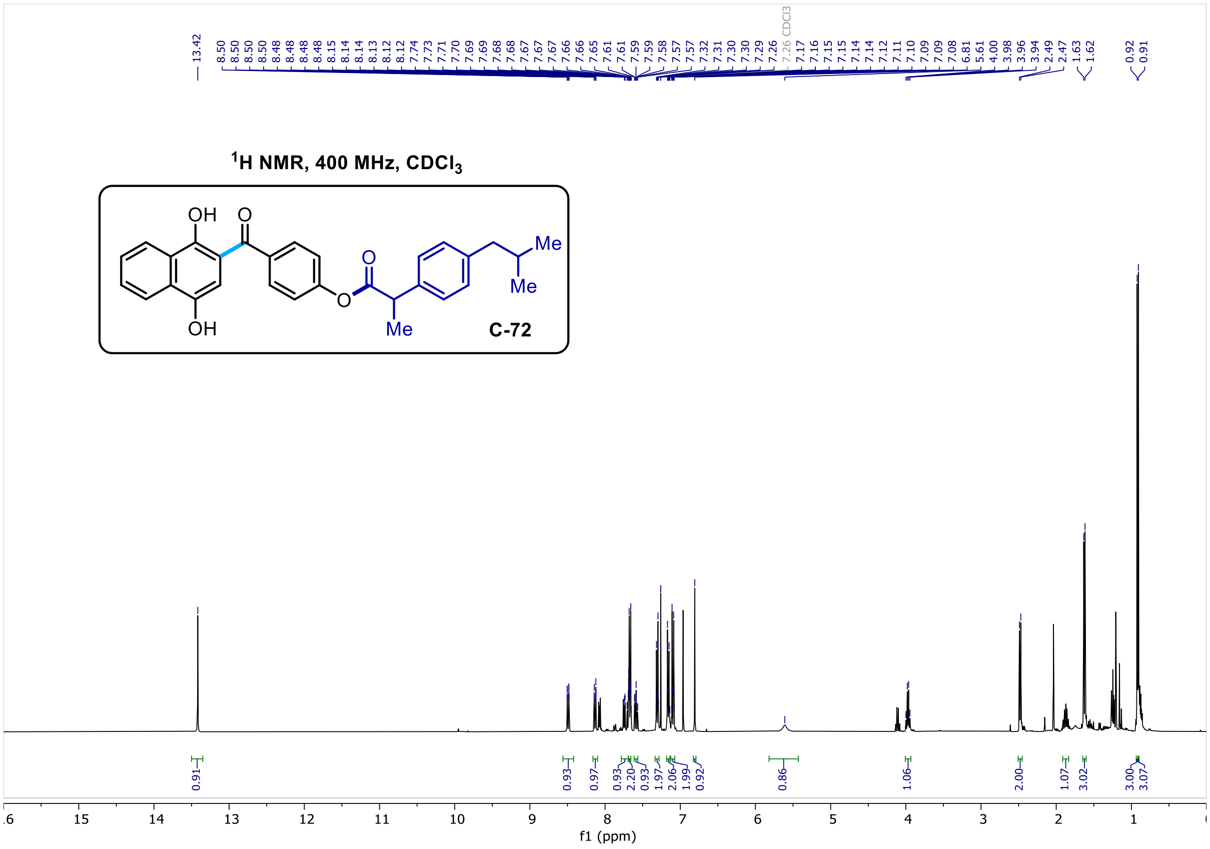

1H NMR, 400 MHz, CDCl3
OH
O
OH
O
O
Me
Me
Me
C-72
f1 (ppm)

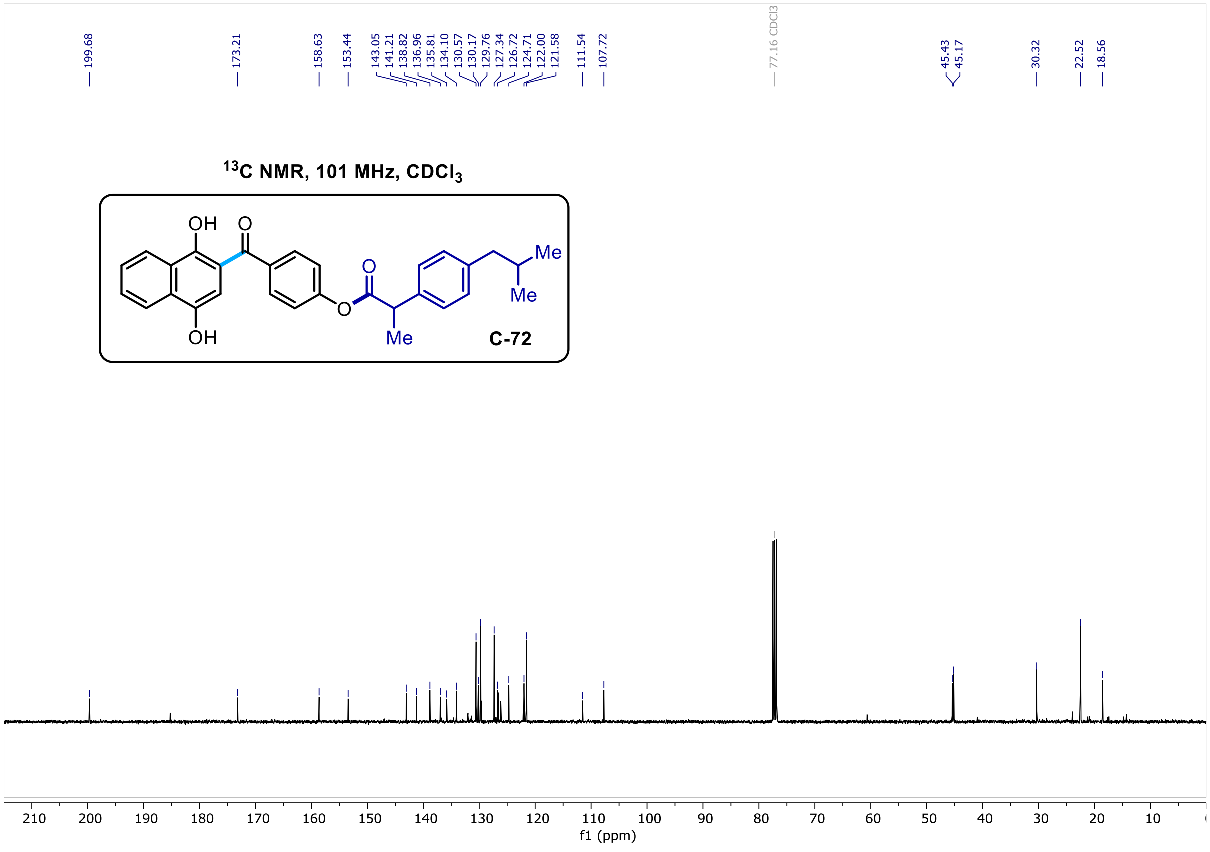

13C NMR, 101 MHz, CDCl3
OH
O
OH
O
O
Me
Me
Me
C-72
f1 (ppm)

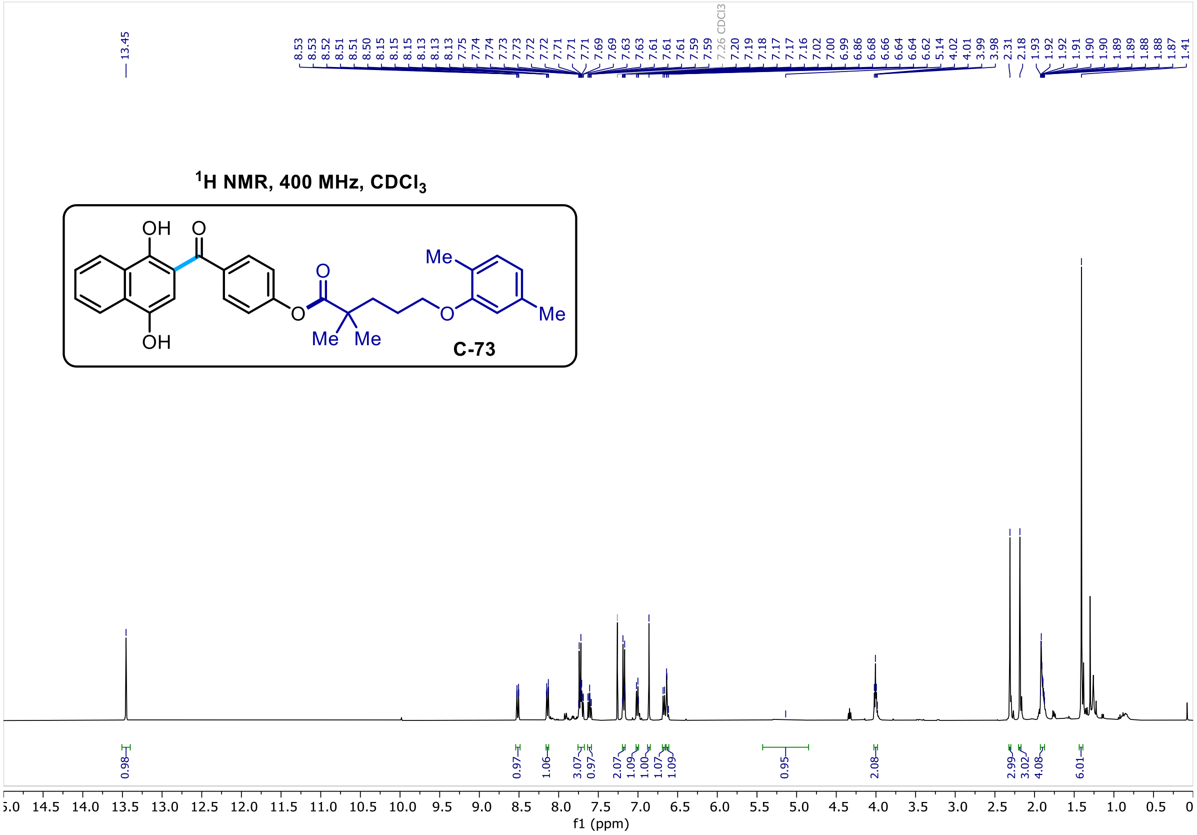

1H NMR, 400 MHz, CDCl3
C-73
f1 (ppm)

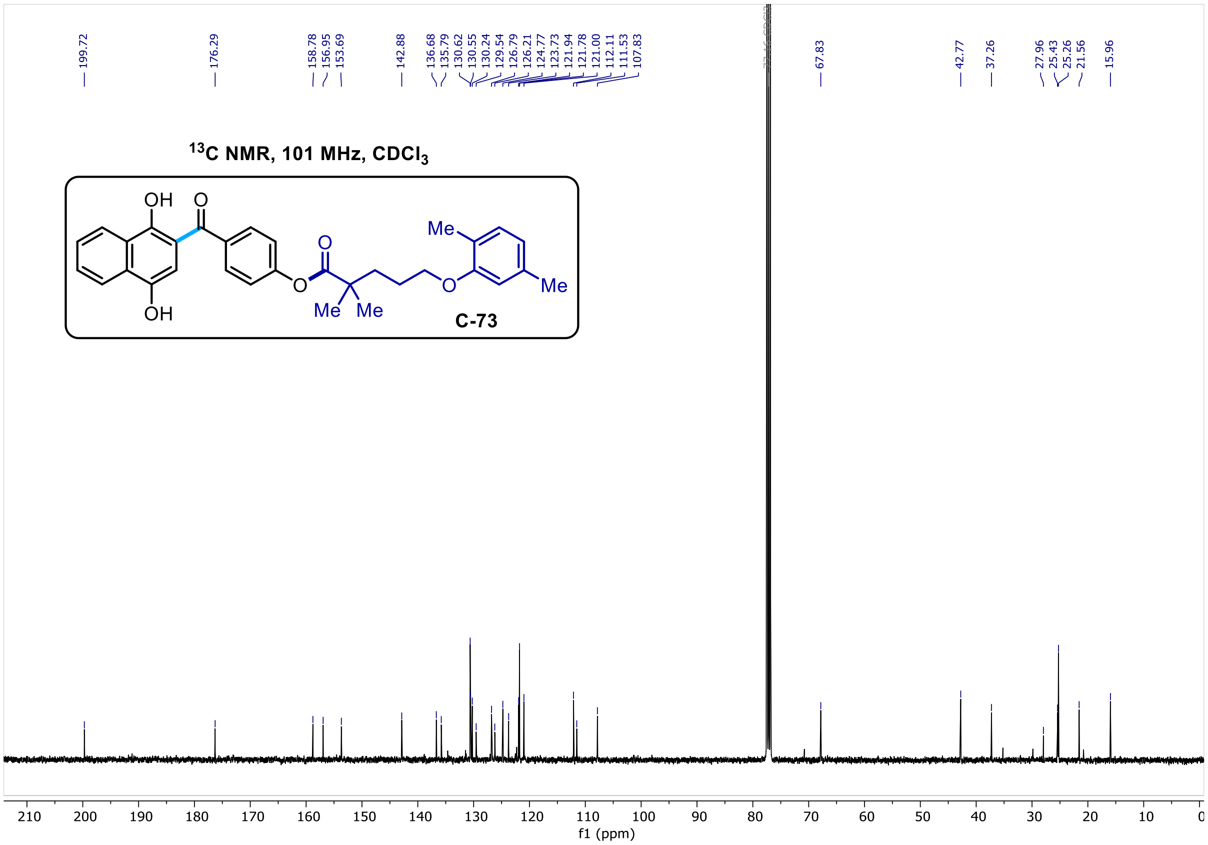

13C NMR, 101 MHz, CDCl3
C-73
f1 (ppm)

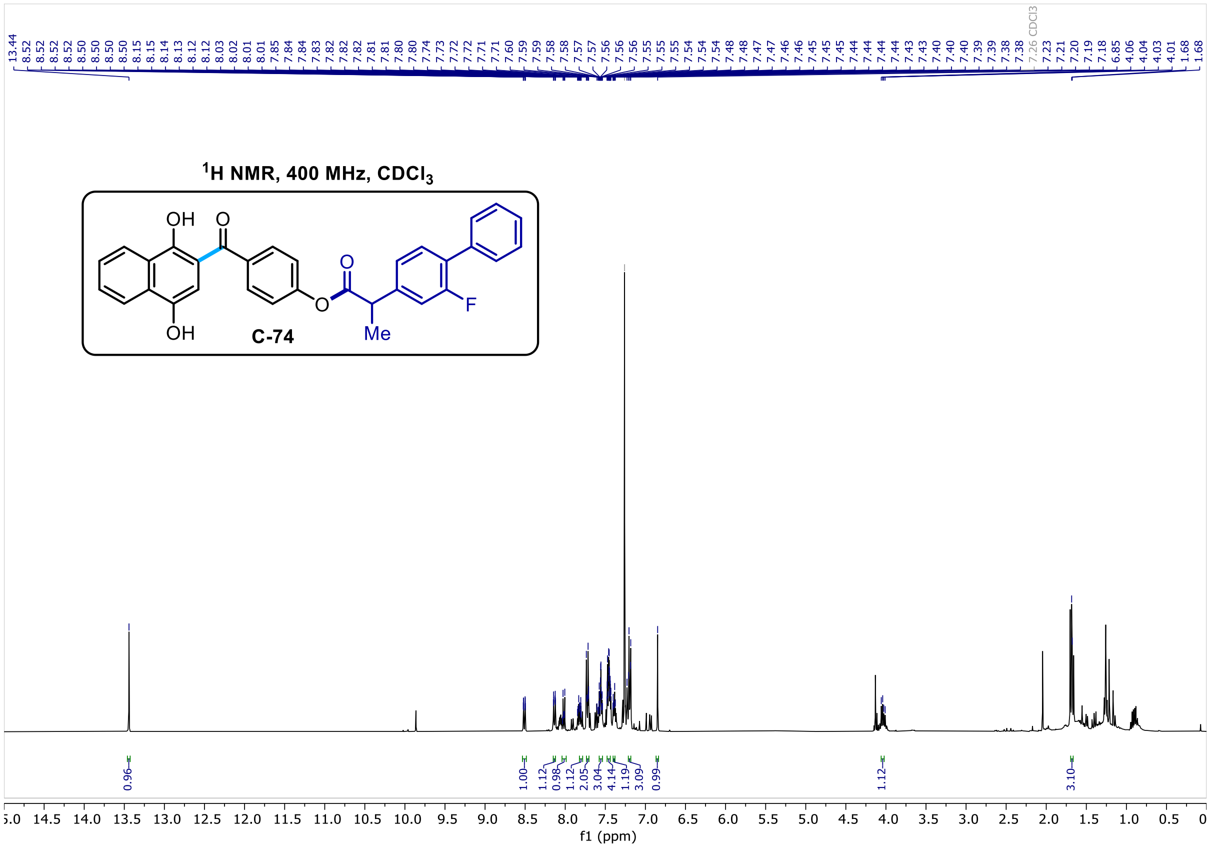

^{1}H NMR, 400 MHz, $CDCl_3$
OH
O
O
O
F
OH
C-74
Me
f1 (ppm)

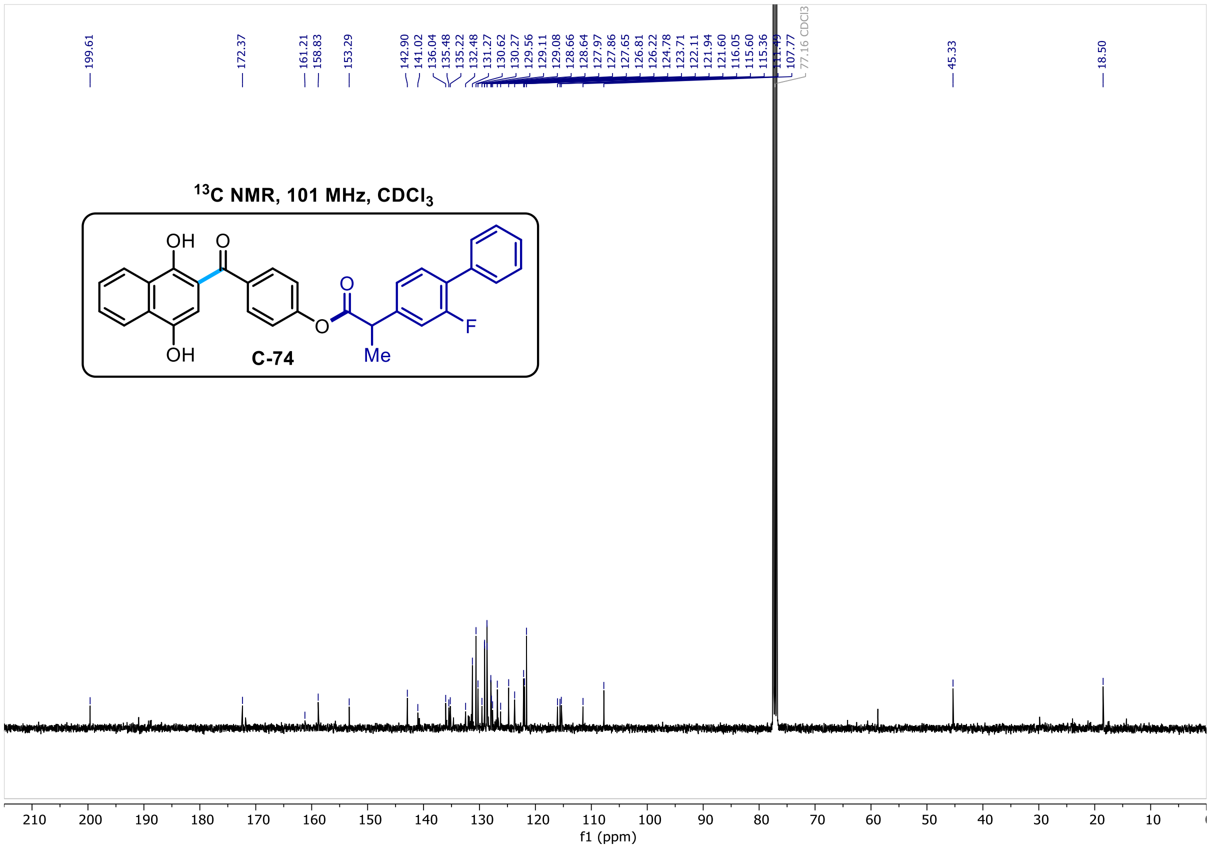

^{13}C NMR, 101 MHz, $CDCl_3$
OH
O
O
O
F
OH
C-74
Me
f1 (ppm)

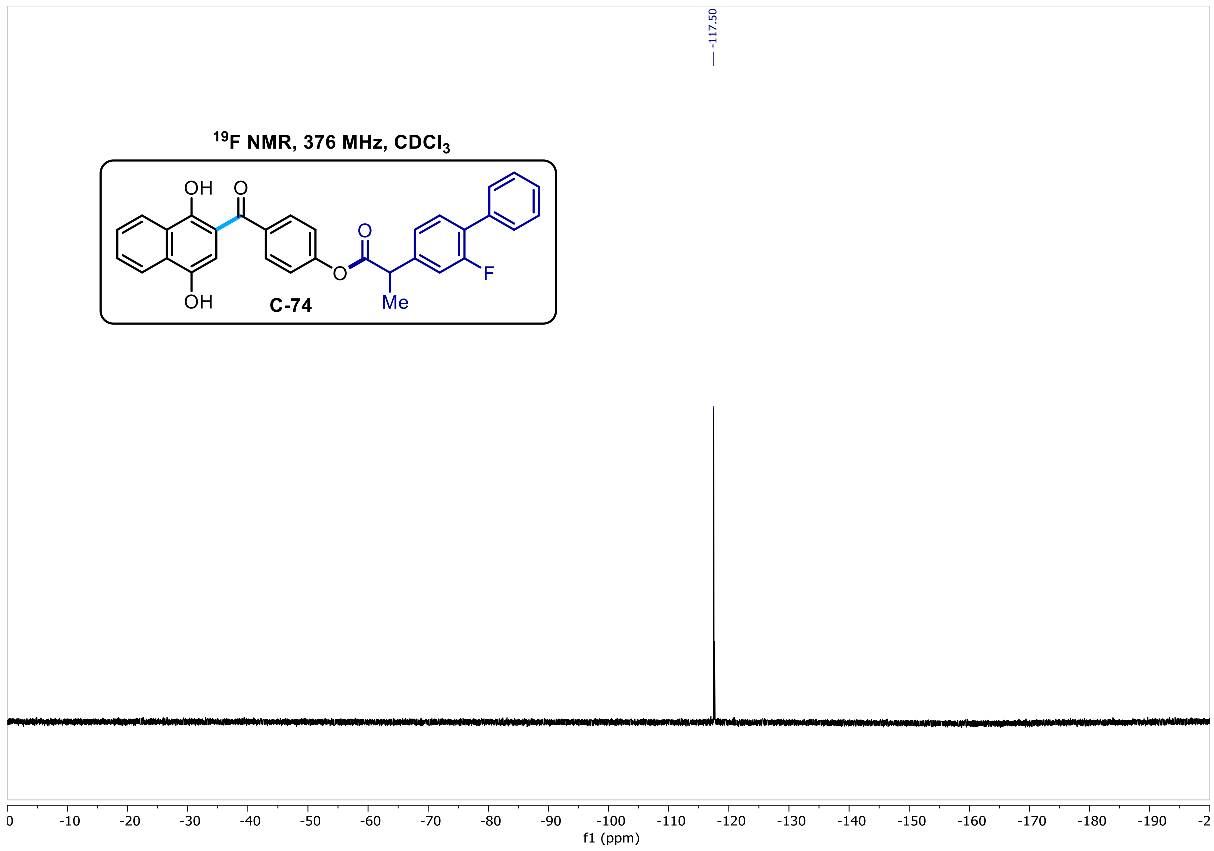

-117.50
19F NMR, 376 MHz, CDCl3
OH
O
O
O
OH
C-74
Me
F
0
-10
-20
-30
-40
-50
-60
-70
-80
-90
-100
-110
-120
-130
-140
-150
-160
-170
-180
-190
-2
f1 (ppm)

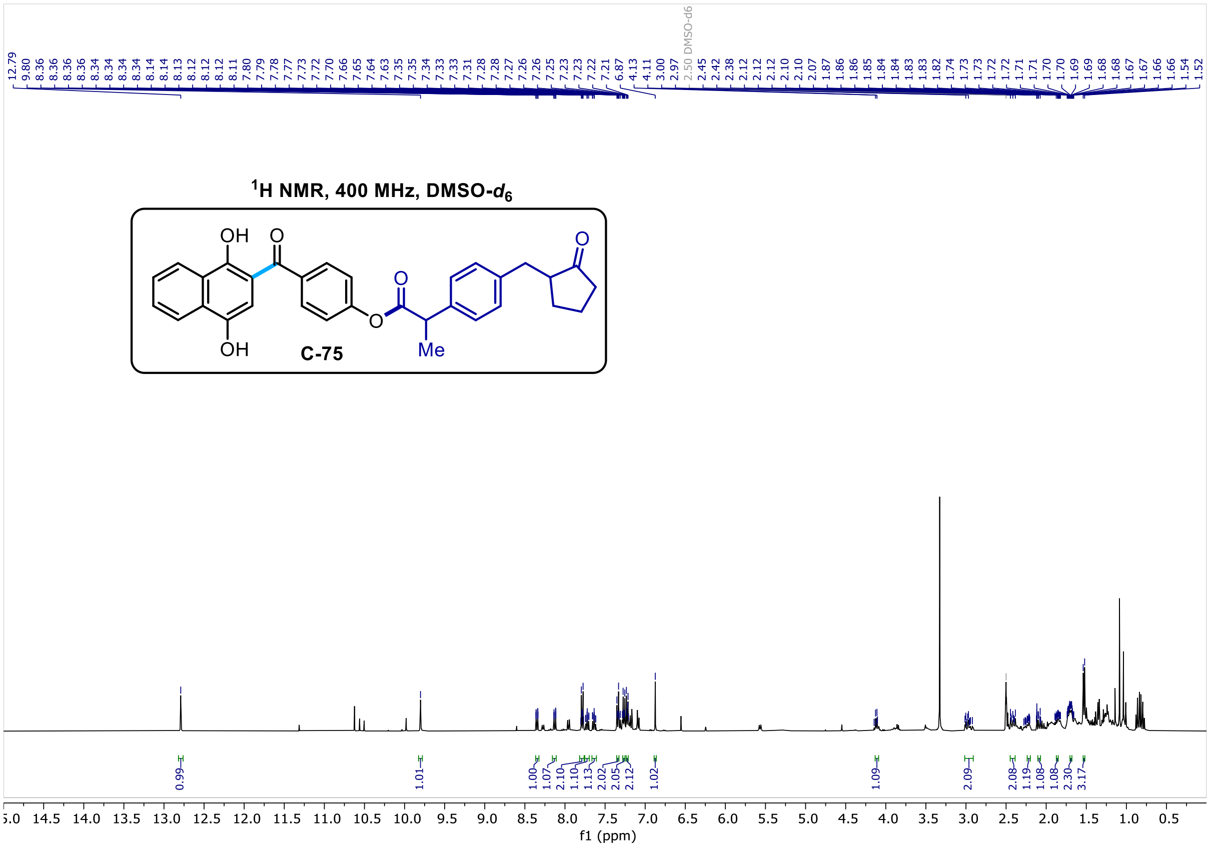

1H NMR, 400 MHz, DMSO-d6
C-75
OH
OH
O
O
O
O
Me
12.79
9.80
8.36
8.36
8.36
8.36
8.34
8.34
8.34
8.34
8.14
8.14
8.13
8.12
8.12
8.12
8.11
7.80
7.79
7.78
7.77
7.73
7.72
7.70
7.66
7.65
7.64
7.63
7.35
7.35
7.34
7.33
7.33
7.31
7.28
7.28
7.27
7.26
7.26
7.25
7.23
7.23
7.22
7.21
6.87
4.13
4.11
3.00
2.97
2.50 DMSO-d6
2.45
2.42
2.38
2.12
2.12
2.12
2.10
2.10
2.07
1.87
1.86
1.86
1.85
1.84
1.84
1.83
1.82
1.74
1.73
1.73
1.72
1.72
1.71
1.71
1.70
1.70
1.69
1.69
1.68
1.68
1.67
1.67
1.66
1.54
1.52
0.99
1.01
1.00
1.07
2.10
1.10
1.13
1.13
2.02
2.05
2.12
1.02
1.09
2.09
2.08
1.19
1.08
1.08
2.30
3.17
f1 (ppm)

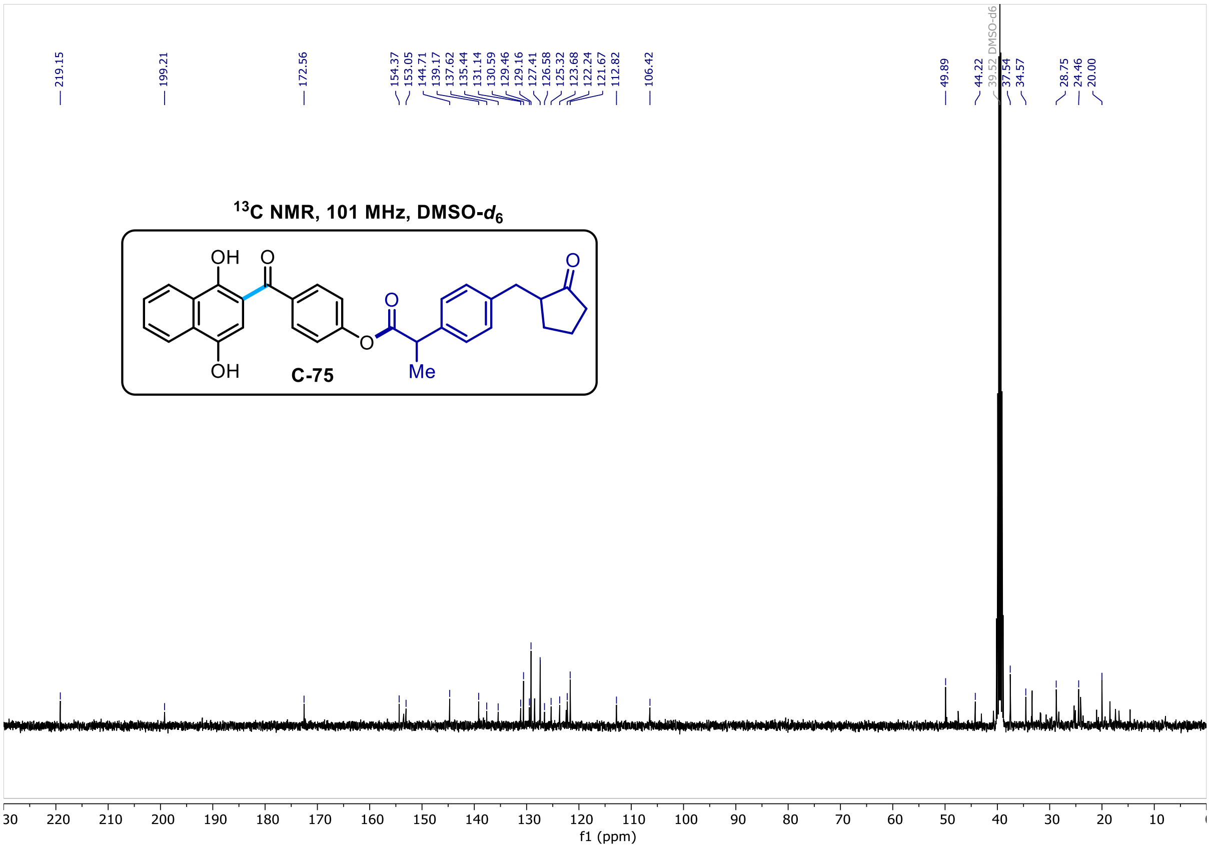

13C NMR, 101 MHz, DMSO-d6
C-75
OH
OH
O
O
O
O
Me
219.15
199.21
172.56
154.37
153.05
144.71
139.17
137.62
135.44
131.14
130.59
129.46
129.16
127.41
126.58
125.32
123.68
122.24
121.67
112.82
106.42
49.89
44.22
39.52 DMSO-d6
37.54
34.57
28.75
24.46
20.00
f1 (ppm)

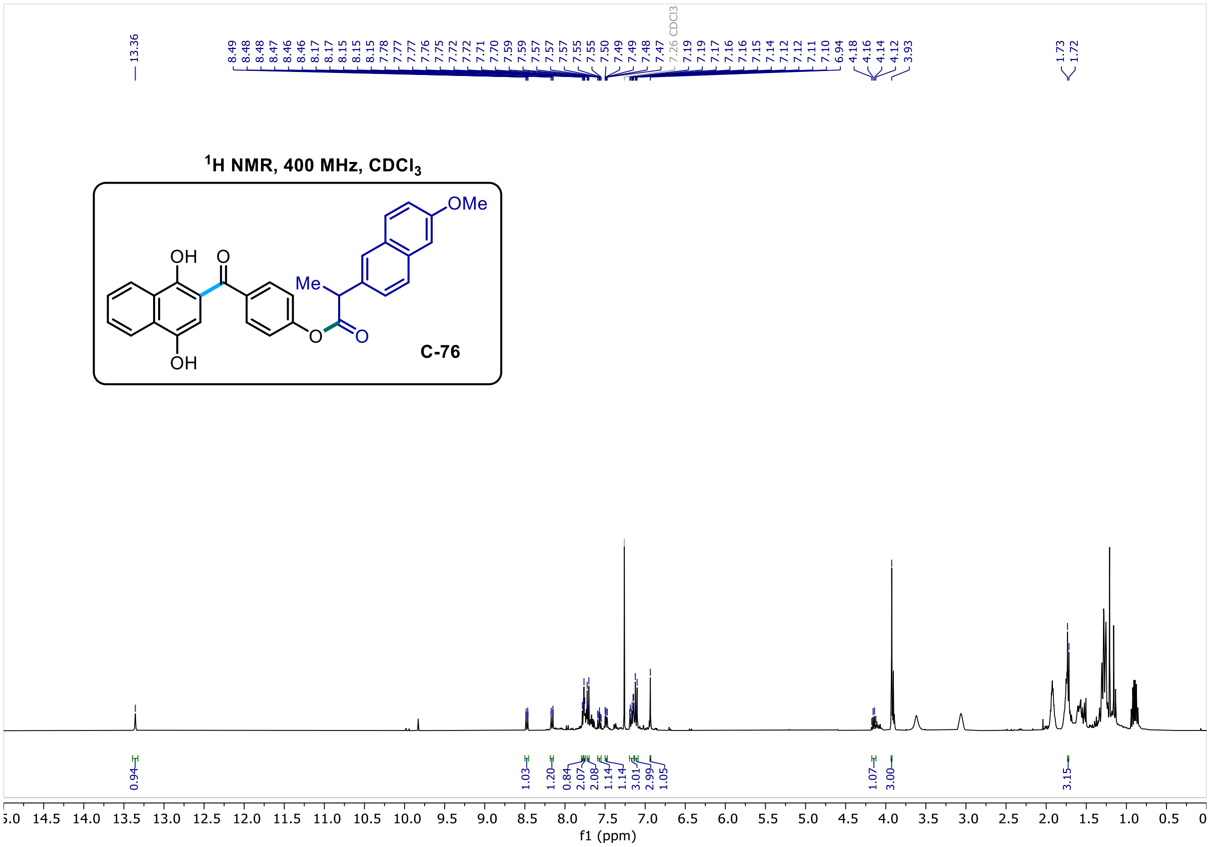
13.36
8.49 8.48 8.48 8.47 8.46 8.46 8.17 8.17 8.15 8.15 7.78 7.77 7.77 7.76 7.75 7.72 7.72 7.71 7.70 7.59 7.59 7.57 7.57 7.55 7.55 7.50 7.49 7.49 7.48 7.47 7.26 CDCl3 7.19 7.19 7.17 7.16 7.16 7.15 7.14 7.12 7.12 7.11 7.10 6.94 4.18 4.16 4.14 4.12 3.93
1.73 1.72
1H NMR, 400 MHz, CDCl3
OMe
OH
O
Me
O
O
OH
C-76
0.94
1.03 1.20 0.84 2.07 2.08 1.14 1.14 3.01 2.99 1.05
1.07 3.00
3.15
f1 (ppm)

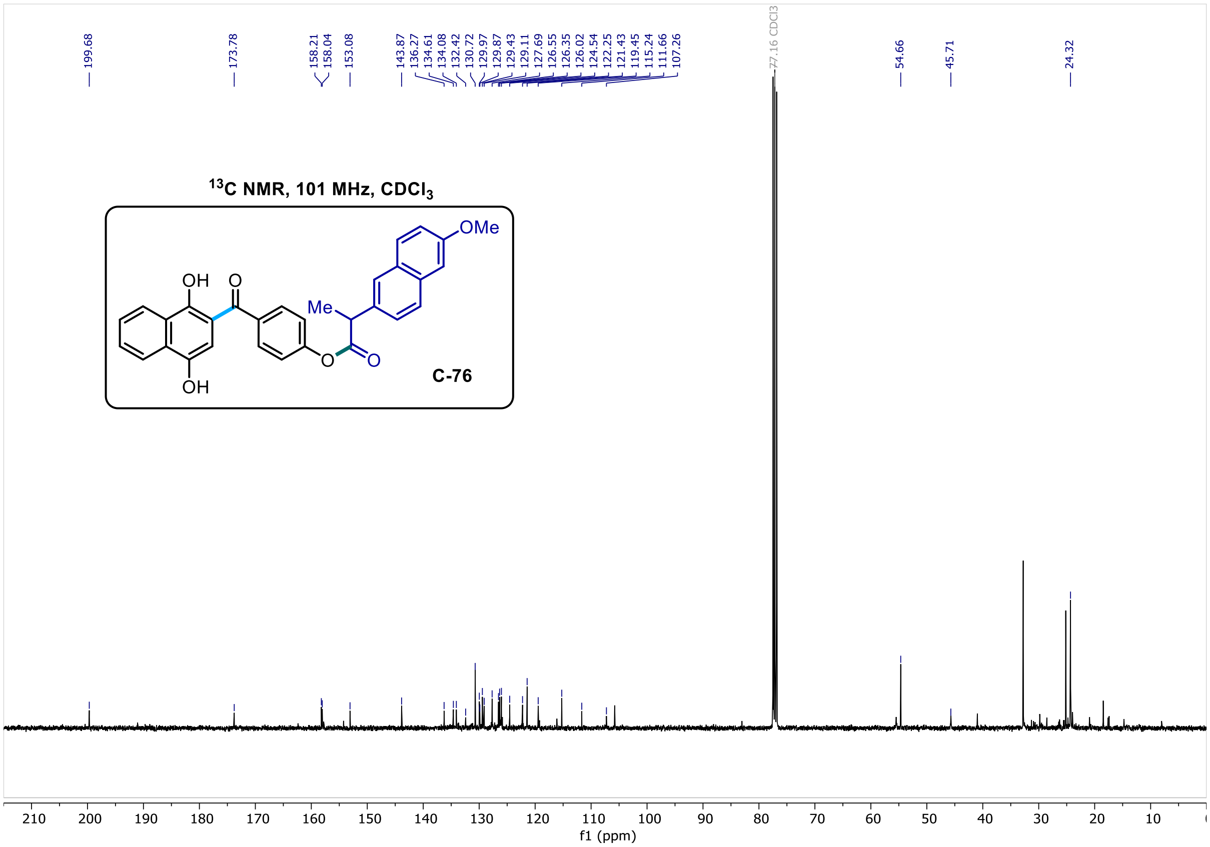
199.68
173.78
158.21 158.04
153.08
143.87 136.27 134.61 134.08 132.42 130.72 129.97 129.87 129.43 129.11 127.69 126.35 126.02 124.54 122.25 121.43 119.45 115.24 111.66 107.26
77.16 CDCl3
54.66
45.71
24.32
13C NMR, 101 MHz, CDCl3
OMe
OH
O
Me
O
O
OH
C-76
f1 (ppm)

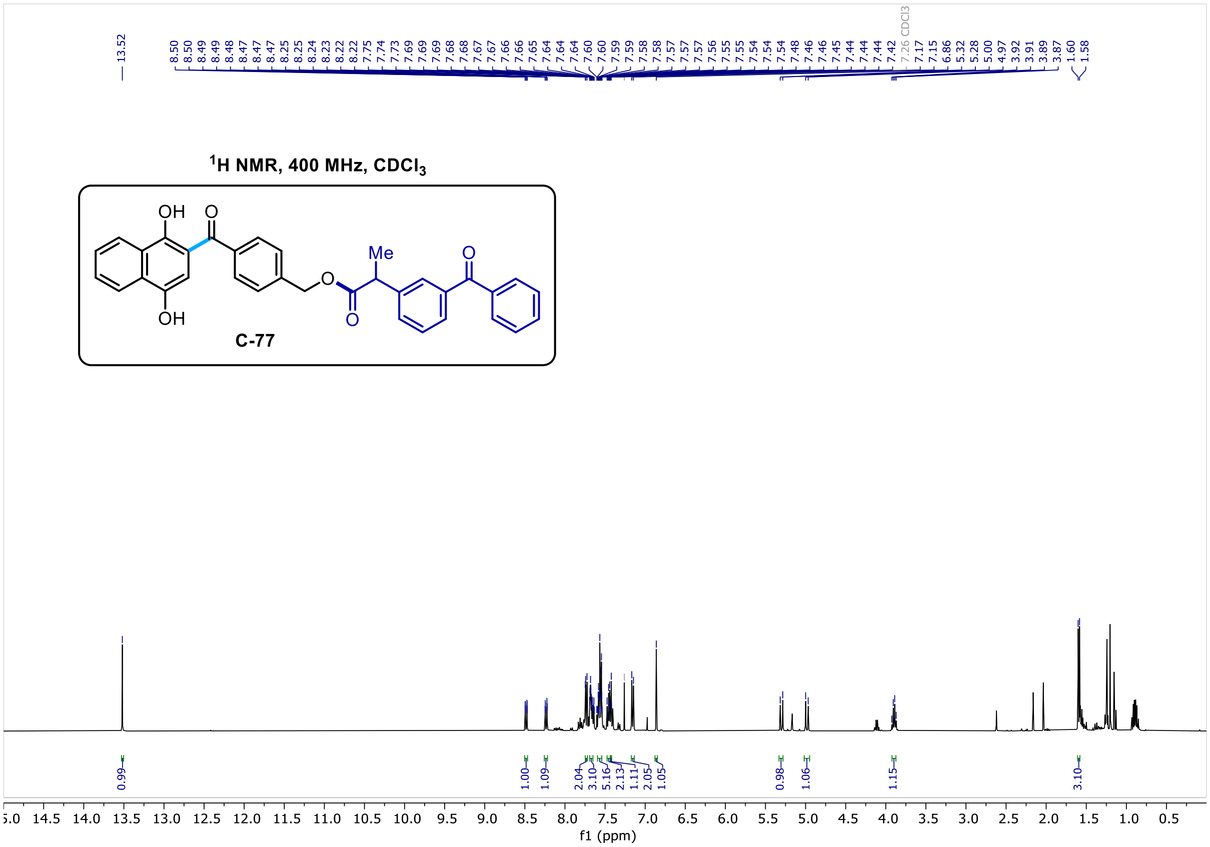
13.52
8.50 8.50 8.49 8.49 8.48 8.47 8.47 8.25 8.25 8.24 8.23 8.22 8.22 7.75 7.74 7.73 7.69 7.69 7.69 7.68 7.68 7.67 7.67 7.66 7.66 7.65 7.64 7.64 7.60 7.60 7.59 7.59 7.58 7.58 7.57 7.57 7.57 7.56 7.55 7.55 7.54 7.54 7.54 7.48 7.46 7.46 7.45 7.44 7.44 7.44 7.42 7.26 CDCl3 7.17 7.15 6.86 5.32 5.28 5.00 4.97 3.92 3.91 3.89 3.87 1.60 1.58
1H NMR, 400 MHz, CDCl3
OH
O
Me
O
O
O
OH
C-77
0.99 1.00 1.09 2.04 3.10 5.16 2.13 1.11 2.05 1.05 0.98 1.06 1.15 3.10
f1 (ppm)

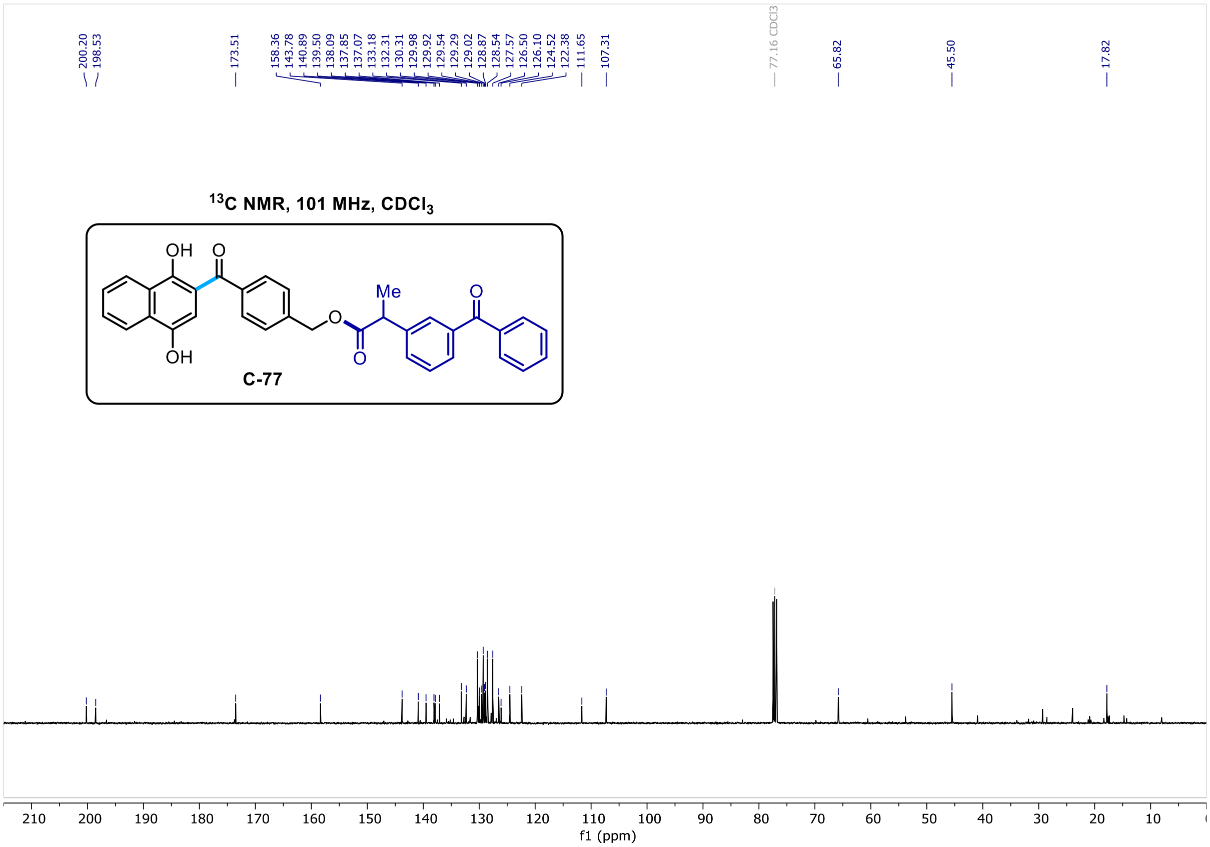
200.20 198.53 173.51 158.36 143.78 140.89 139.50 138.09 137.85 137.07 133.18 132.31 130.31 129.98 129.92 129.54 129.29 129.02 128.87 128.54 127.57 126.50 126.10 124.52 122.38 111.65 107.31 77.16 CDCl3 65.82 45.50 17.82
13C NMR, 101 MHz, CDCl3
OH
O
Me
O
O
O
OH
C-77
f1 (ppm)

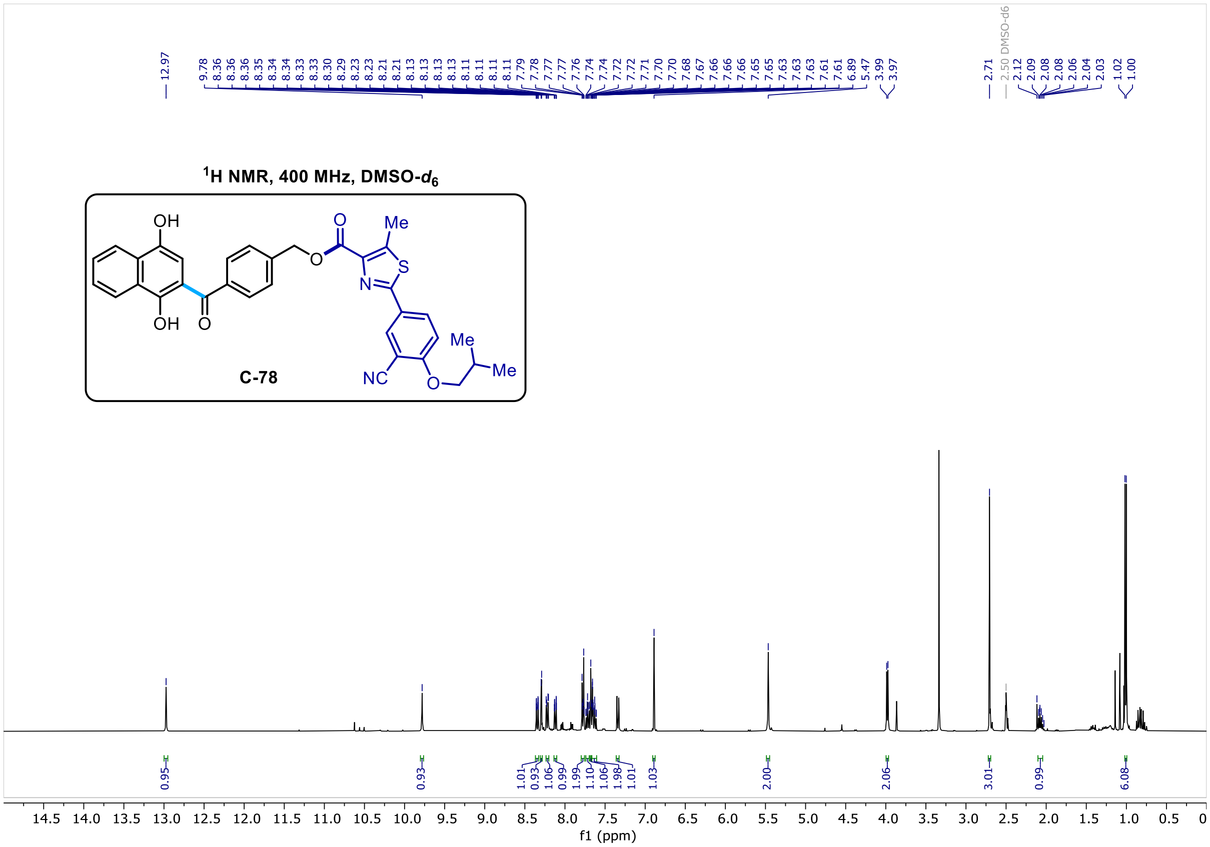
12.97
9.78 8.36 8.36 8.35 8.34 8.34 8.33 8.33 8.30 8.29 8.23 8.23 8.21 8.21 8.13 8.13 8.13 8.13 8.11 8.11 8.11 8.11 7.79 7.78 7.78 7.77 7.77 7.76 7.74 7.74 7.72 7.72 7.71 7.70 7.68 7.67 7.66 7.66 7.65 7.65 7.63 7.63 7.61 7.61 6.89 5.47 3.99 3.97
2.71
2.50 DMSO-d6
2.12 2.09 2.08 2.08 2.06 2.04 2.03 1.02 1.00
1H NMR, 400 MHz, DMSO-d6
OH
OH
O
O
O
Me
S
N
Me
Me
NC
O
C-78
0.95 0.93 1.01 0.93 1.06 0.99 1.99 1.10 1.06 1.98 1.01 1.03 2.00 2.06 3.01 0.99 6.08
14.5 14.0 13.5 13.0 12.5 12.0 11.5 11.0 10.5 10.0 9.5 9.0 8.5 8.0 7.5 7.0 6.5 6.0 5.5 5.0 4.5 4.0 3.5 3.0 2.5 2.0 1.5 1.0 0.5 0
f1 (ppm)

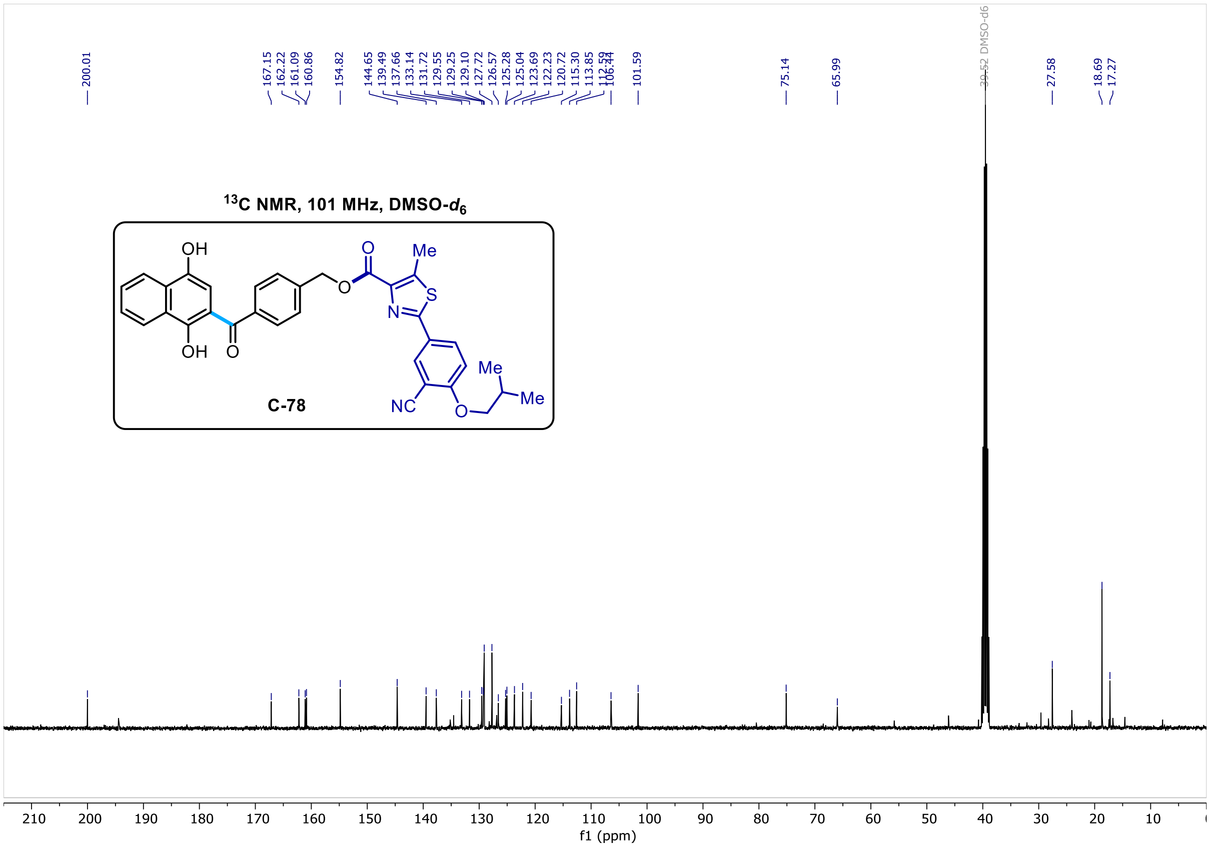
200.01
167.15 162.22 161.09 160.86
154.82
144.65 139.49 137.66 133.14 131.72 129.55 129.25 129.10 127.72 126.57 125.28 125.04 123.69 122.23 120.72 115.30 113.85
101.59
75.14
65.99
39.52 DMSO-d6
27.58
18.69 17.27
13C NMR, 101 MHz, DMSO-d6
OH
OH
O
O
O
Me
S
N
Me
Me
NC
O
C-78
210 200 190 180 170 160 150 140 130 120 110 100 90 80 70 60 50 40 30 20 10
f1 (ppm)

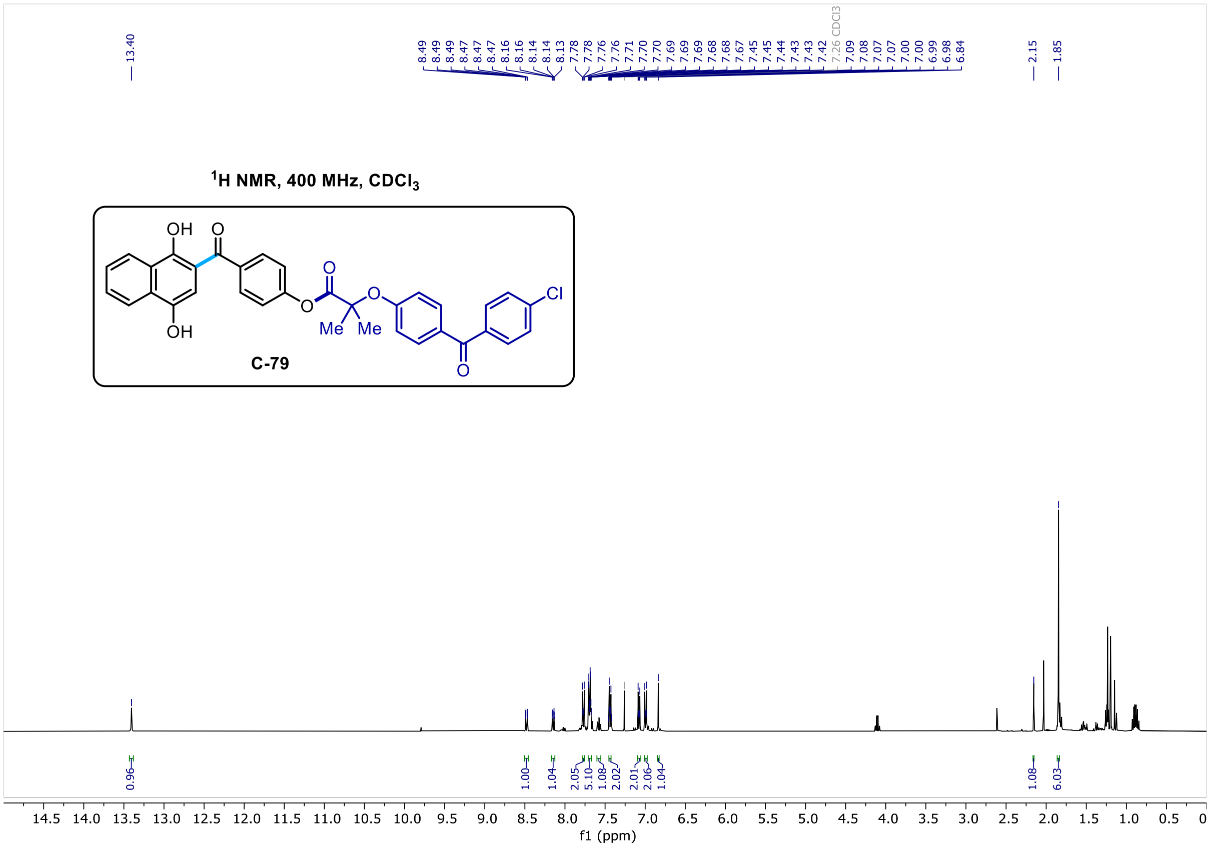
13.40
8.49 8.49 8.49 8.47 8.47 8.47 8.16 8.16 8.14 8.14 8.13 7.78 7.78 7.76 7.76 7.71 7.70 7.70 7.69 7.69 7.69 7.68 7.68 7.67 7.45 7.44 7.43 7.43 7.42
7.26 CDCl3
7.09 7.08 7.07 7.07 7.00 7.00 6.99 6.98 6.84
2.15
1.85
^{1}H NMR, 400 MHz, $CDCl_3$
OH
O
OH
O
O
O
Me
Me
Cl
O
C-79
0.96
1.00
1.04
2.05
5.10
1.08
2.02
2.01
2.06
1.04
1.08
6.03
14.5 14.0 13.5 13.0 12.5 12.0 11.5 11.0 10.5 10.0 9.5 9.0 8.5 8.0 7.5 7.0 6.5 6.0 5.5 5.0 4.5 4.0 3.5 3.0 2.5 2.0 1.5 1.0 0.5 0
f1 (ppm)

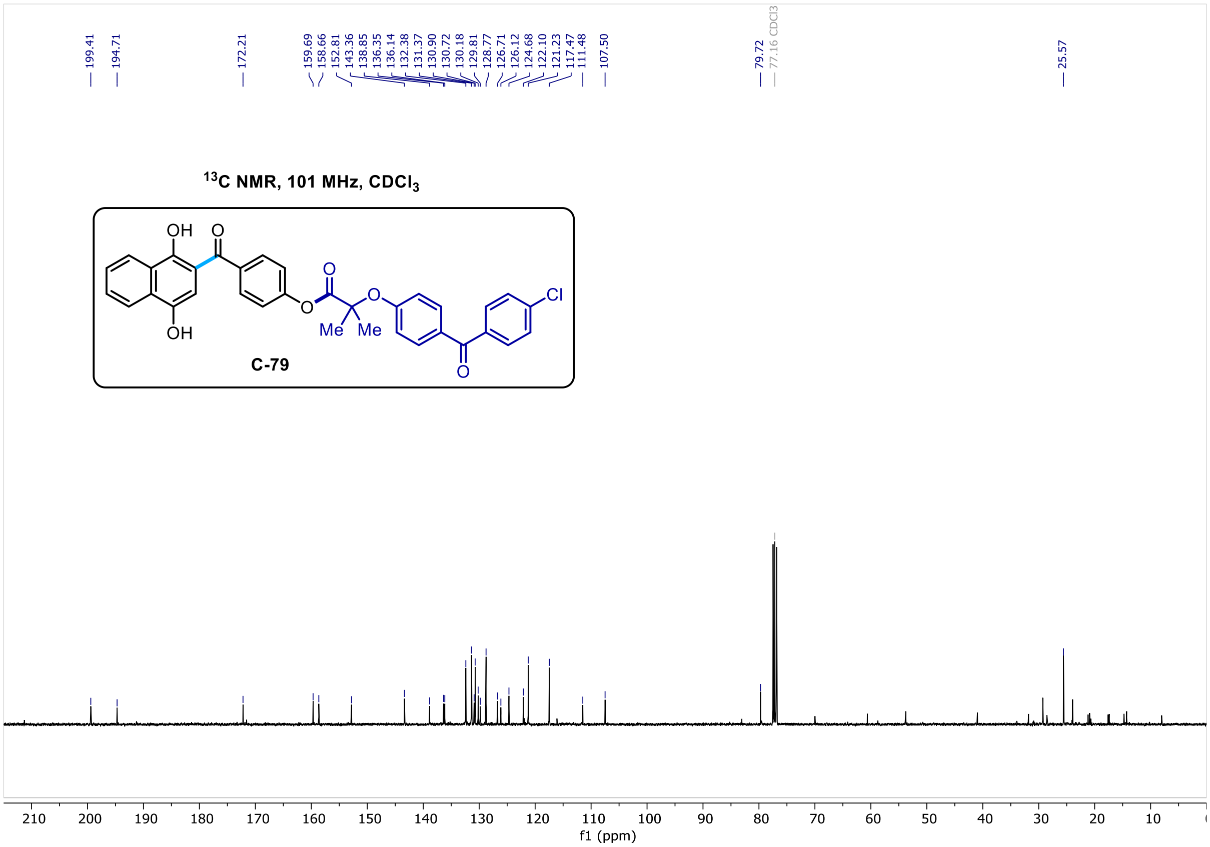
199.41
194.71
172.21
159.69 158.66 152.81 143.36 138.85 136.35 136.14 132.38 131.37 130.90 130.72 130.18 129.81 128.77 126.71 126.12 124.68 122.10 121.23 117.47 111.48
107.50
79.72
77.16 CDCl3
25.57
^{13}C NMR, 101 MHz, $CDCl_3$
OH
O
OH
O
O
O
Me
Me
Cl
O
C-79
210 200 190 180 170 160 150 140 130 120 110 100 90 80 70 60 50 40 30 20 10
f1 (ppm)

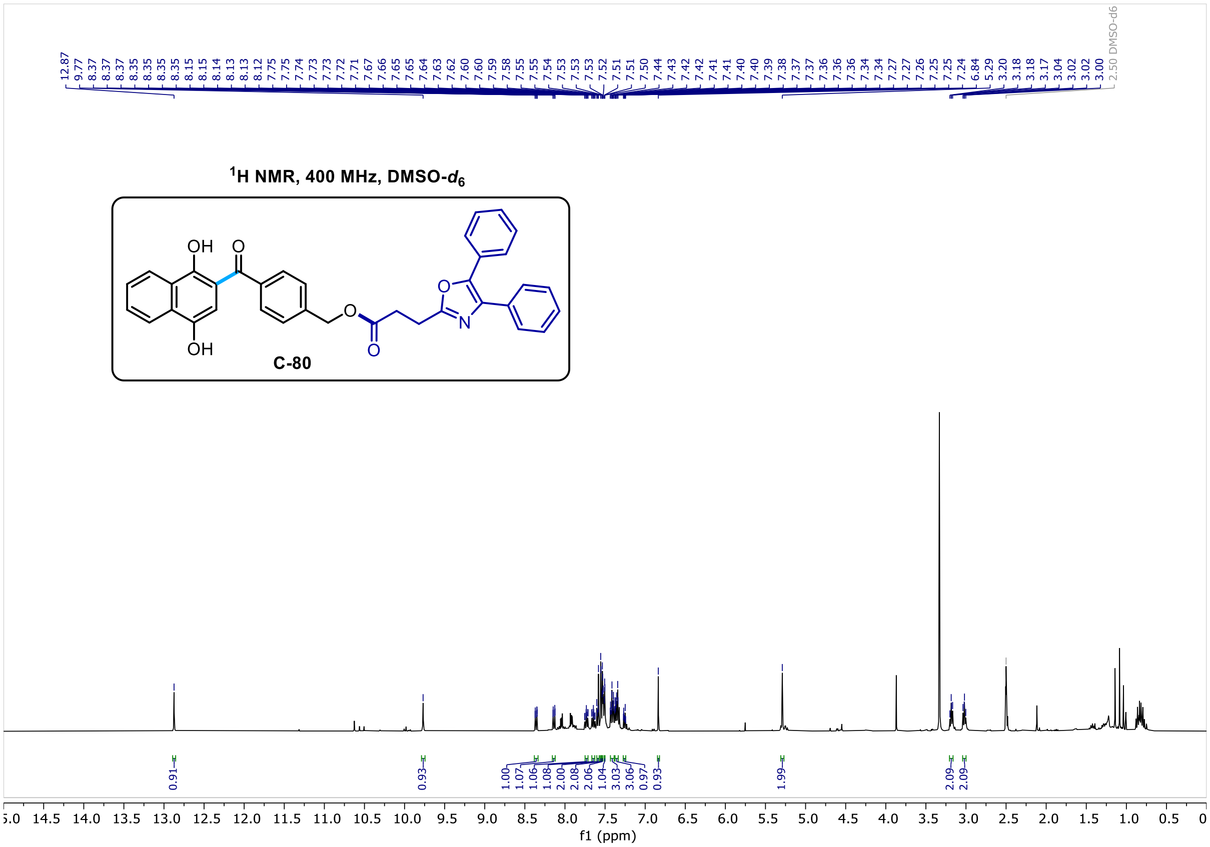
^{1}H NMR, 400 MHz, DMSO-d_6
OH
O
OH
O
O
N
O
C-80
f1 (ppm)
12.87
9.77
6.84
5.29
3.20
3.18
3.17
3.04
3.02
3.00
2.50 DMSO-d6

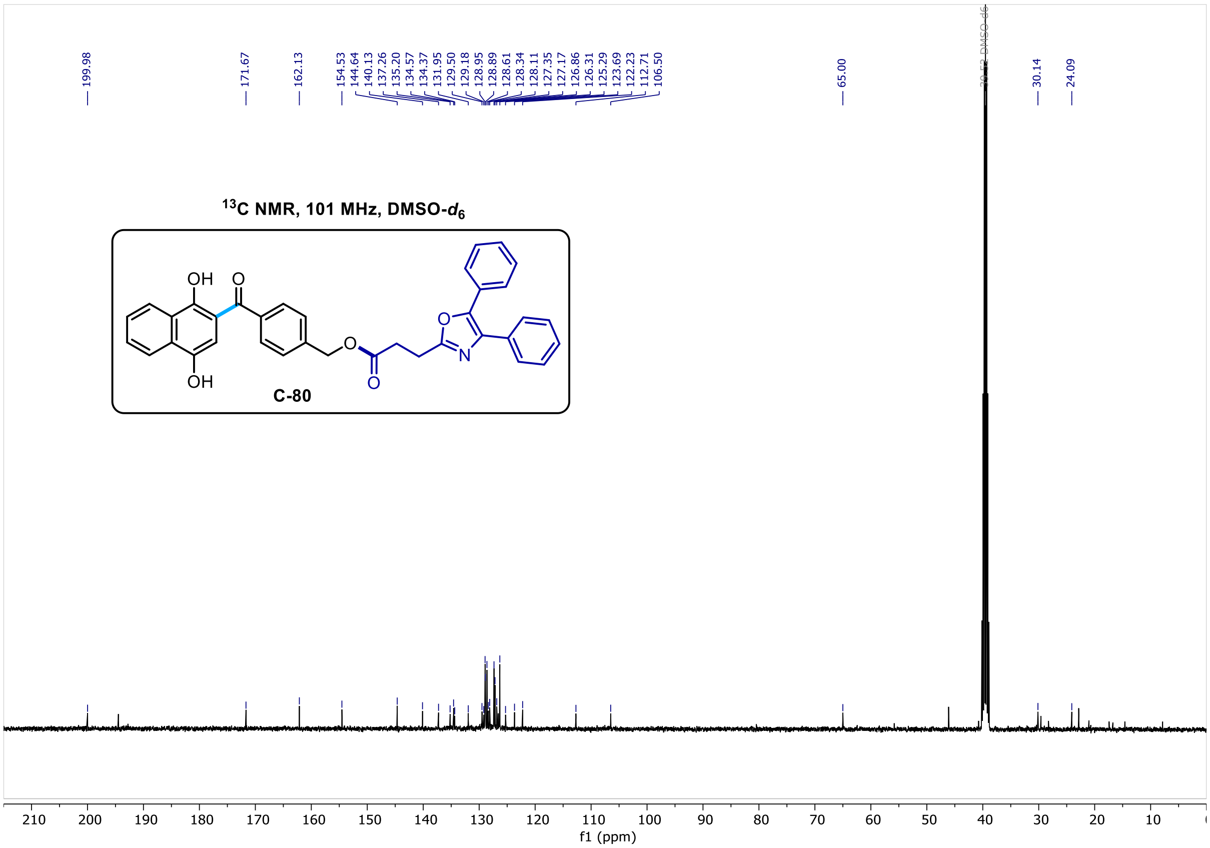
^{13}C NMR, 101 MHz, DMSO-d_6
OH
O
OH
O
O
N
O
C-80
f1 (ppm)
199.98
171.67
162.13
154.53
144.64
140.13
137.26
135.20
134.57
134.37
131.95
129.50
129.18
128.95
128.89
128.61
128.34
128.11
127.35
127.17
126.86
126.31
125.29
123.69
122.23
112.71
106.50
65.00
30.14
24.09

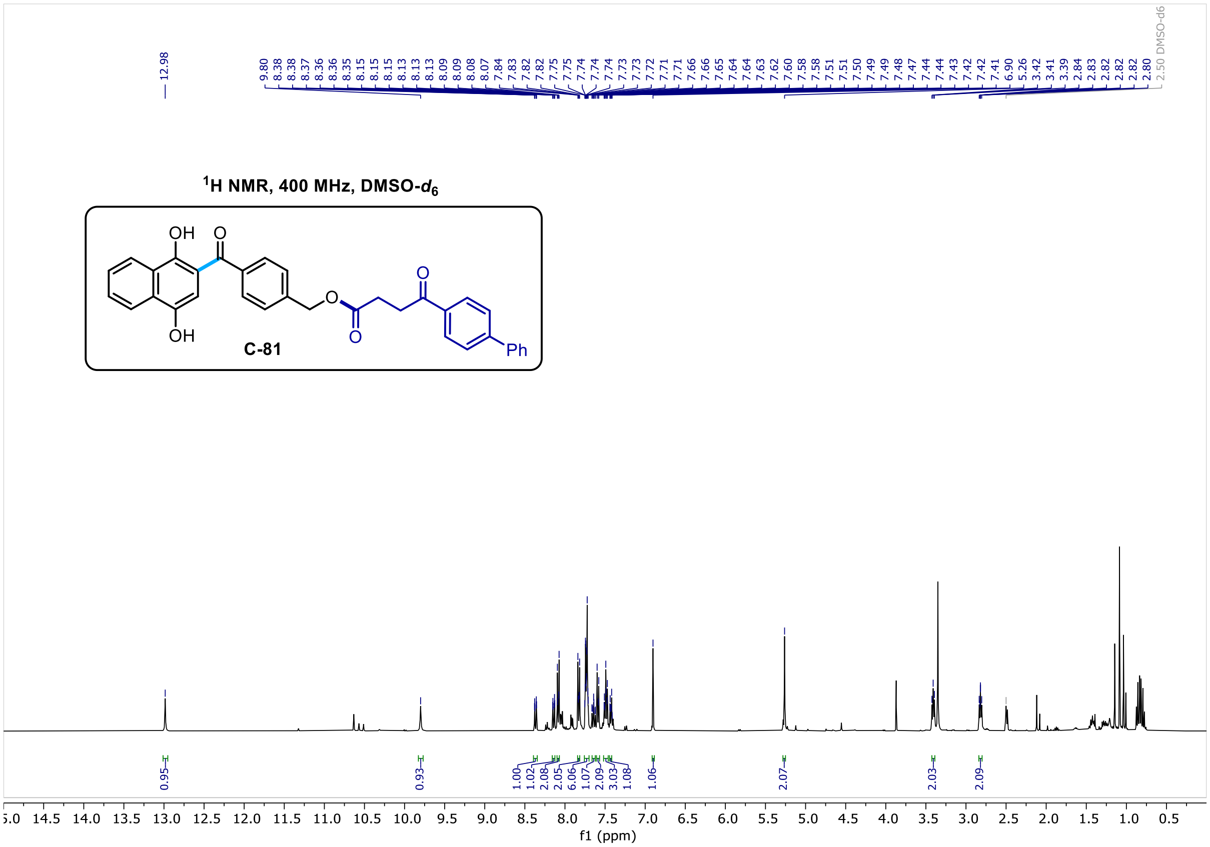
12.98
9.80 8.38 8.38 8.37 8.36 8.36 8.35 8.15 8.15 8.15 8.13 8.13 8.13 8.09 8.09 8.08 8.07 7.84 7.83 7.82 7.82 7.75 7.75 7.74 7.74 7.74 7.73 7.73 7.72 7.71 7.71 7.66 7.65 7.64 7.64 7.63 7.62 7.60 7.58 7.58 7.51 7.50 7.49 7.49 7.48 7.47 7.44 7.44 7.43 7.42 7.42 7.41 6.90 5.26 3.42 3.41 3.39 2.84 2.83 2.82 2.82 2.80
2.50 DMSO-d6
1H NMR, 400 MHz, DMSO-d6
OH
O
O
O
OH
O
C-81
Ph
0.95 0.93 1.00 1.02 2.08 2.05 6.06 1.07 2.09 3.03 1.08 1.06 2.07 2.03 2.09
f1 (ppm)

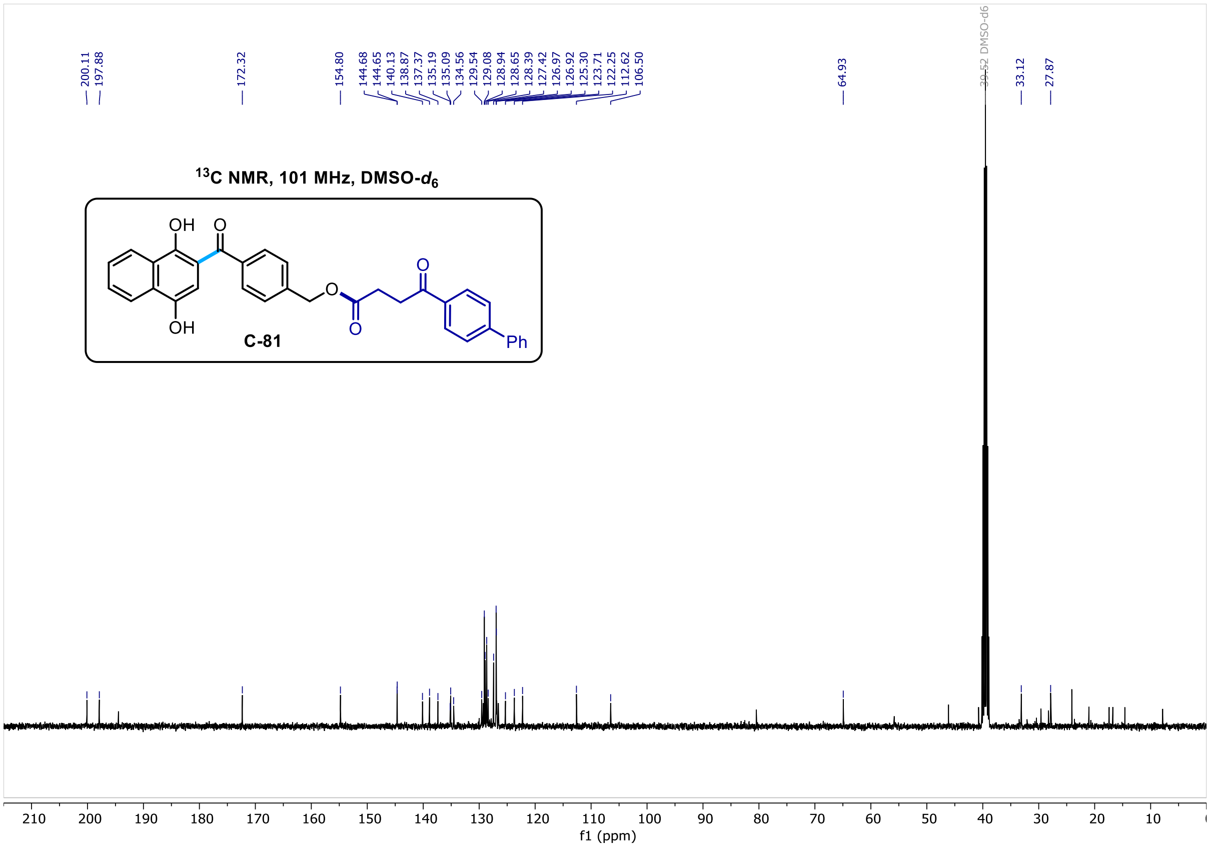
200.11 197.88
172.32
154.80 144.68 144.65 140.13 138.87 137.37 135.19 135.09 134.56 129.54 129.08 128.94 128.65 128.39 127.42 126.97 126.92 125.30 123.71 122.25 112.62 106.50
64.93
39.52 DMSO-d6
33.12
27.87
13C NMR, 101 MHz, DMSO-d6
OH
O
O
O
OH
O
C-81
Ph
f1 (ppm)

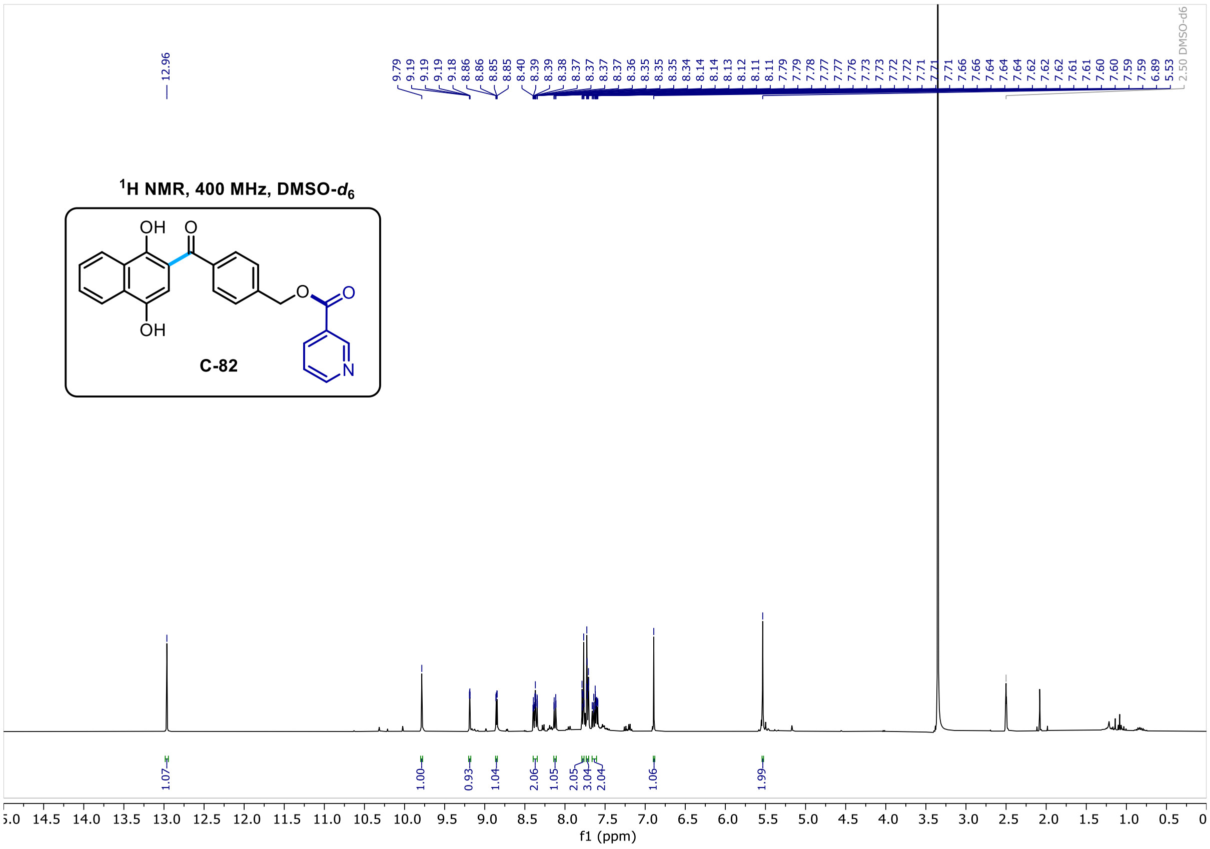

12.96
9.79 9.19 9.19 9.19 9.18 8.86 8.86 8.85 8.85 8.40 8.39 8.39 8.38 8.37 8.37 8.37 8.37 8.36 8.35 8.35 8.35 8.34 8.14 8.14 8.13 8.12 8.11 8.11 7.79 7.79 7.78 7.77 7.77 7.76 7.73 7.73 7.72 7.72 7.71 7.71 7.71 7.66 7.66 7.64 7.64 7.62 7.62 7.61 7.60 7.59 7.59 6.89 5.53
2.50 DMSO-d6
1H NMR, 400 MHz, DMSO-d6
OH
O
O
O
OH
N
C-82
1.07 1.00 0.93 1.04 2.06 1.05 2.05 3.04 2.04 1.06 1.99
15.0 14.5 14.0 13.5 13.0 12.5 12.0 11.5 11.0 10.5 10.0 9.5 9.0 8.5 8.0 7.5 7.0 6.5 6.0 5.5 5.0 4.5 4.0 3.5 3.0 2.5 2.0 1.5 1.0 0.5 0
f1 (ppm)

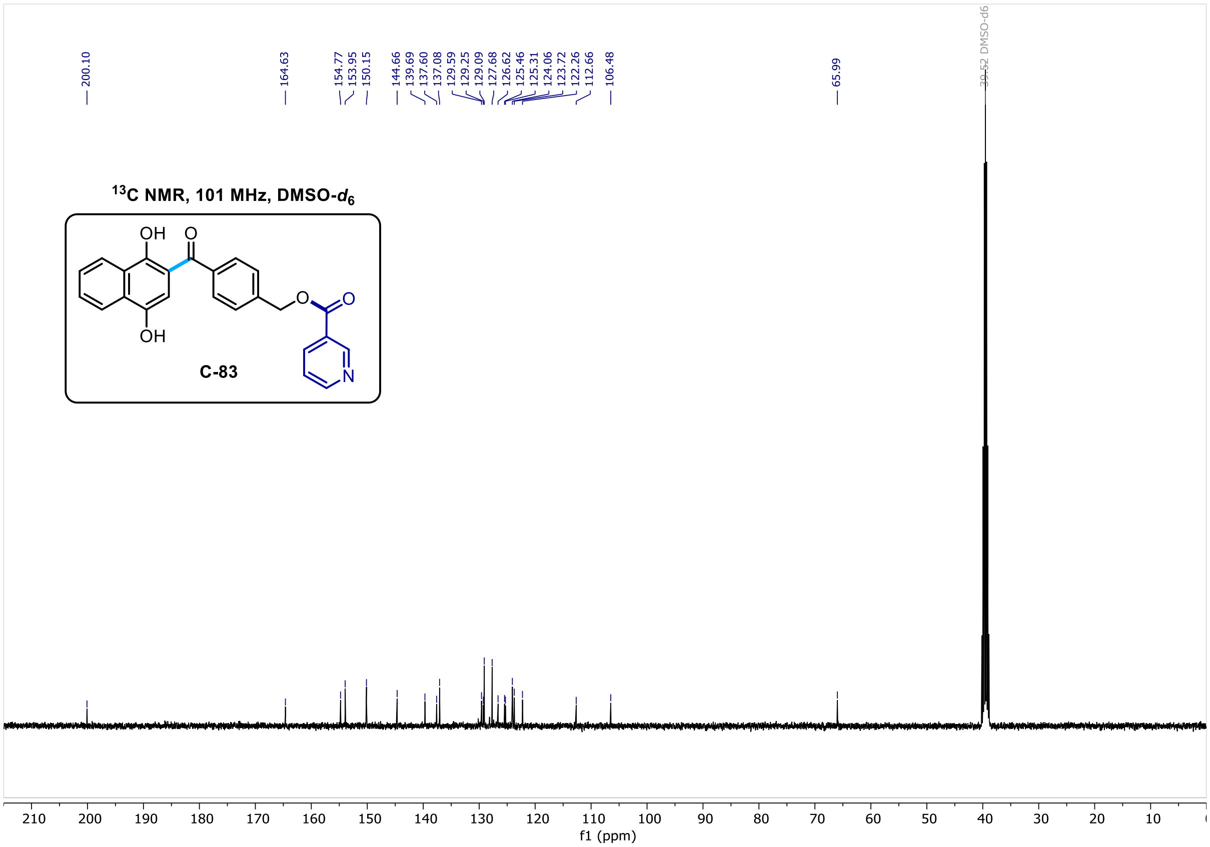

200.10
164.63
154.77 153.95 150.15
144.66 139.69 137.60 137.08 129.59 129.25 129.09 127.68 126.62 125.46 125.31 124.06 123.72 122.26 112.66 106.48
65.99
39.52 DMSO-d6
13C NMR, 101 MHz, DMSO-d6
OH
O
O
O
OH
N
C-83
210 200 190 180 170 160 150 140 130 120 110 100 90 80 70 60 50 40 30 20 10 0
f1 (ppm)

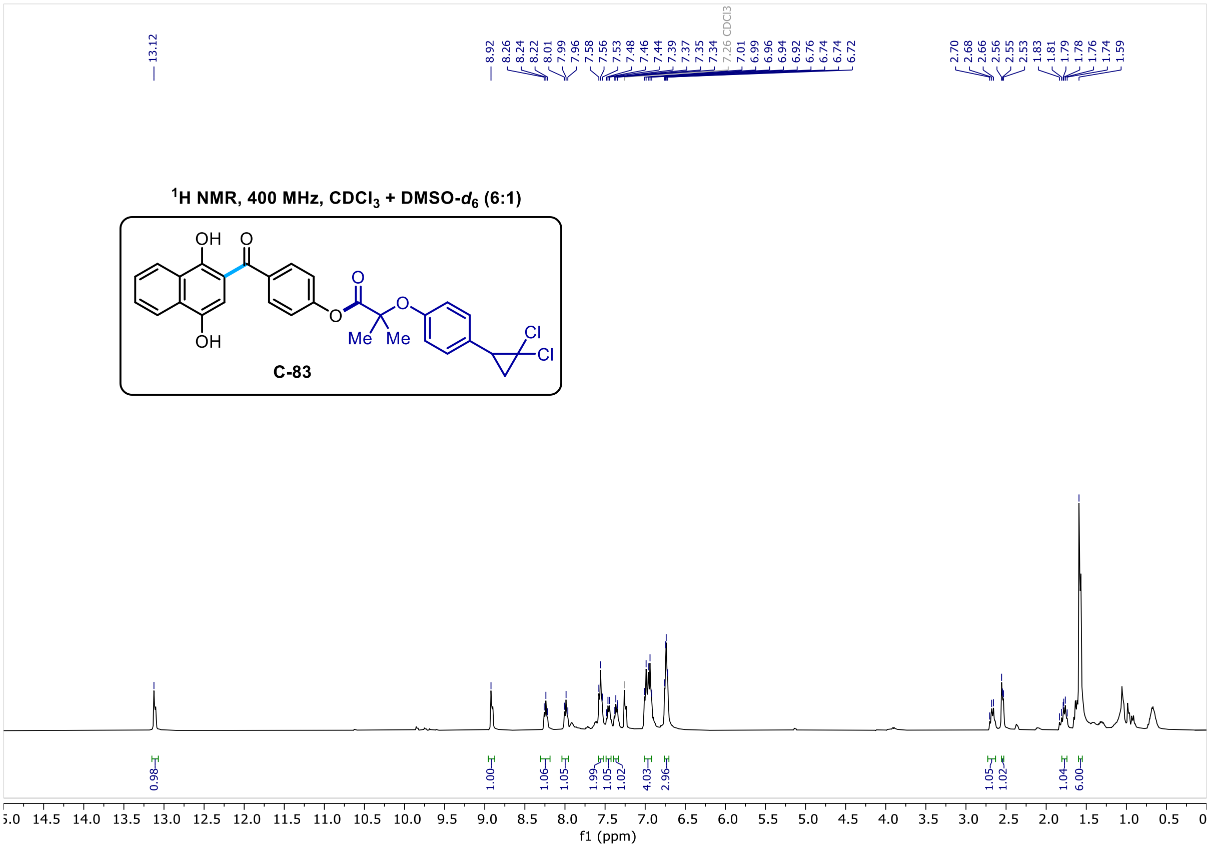
1H NMR, 400 MHz, CDCl3 + DMSO-d6 (6:1)
C-83
OH
O
OH
O
O
O
Me
Me
Cl
Cl
13.12
8.92 8.26 8.24 8.22 8.01 7.99 7.96 7.58 7.56 7.53 7.48 7.46 7.44 7.39 7.37 7.35 7.34 7.26 CDCl3 7.01 6.99 6.96 6.94 6.92 6.76 6.74 6.74 6.72
2.70 2.68 2.66 2.56 2.55 2.53 1.83 1.81 1.79 1.78 1.76 1.74 1.59
0.98 1.00 1.06 1.05 1.99 1.05 1.02 4.03 2.96 1.05 1.02 1.04 6.00
f1 (ppm)

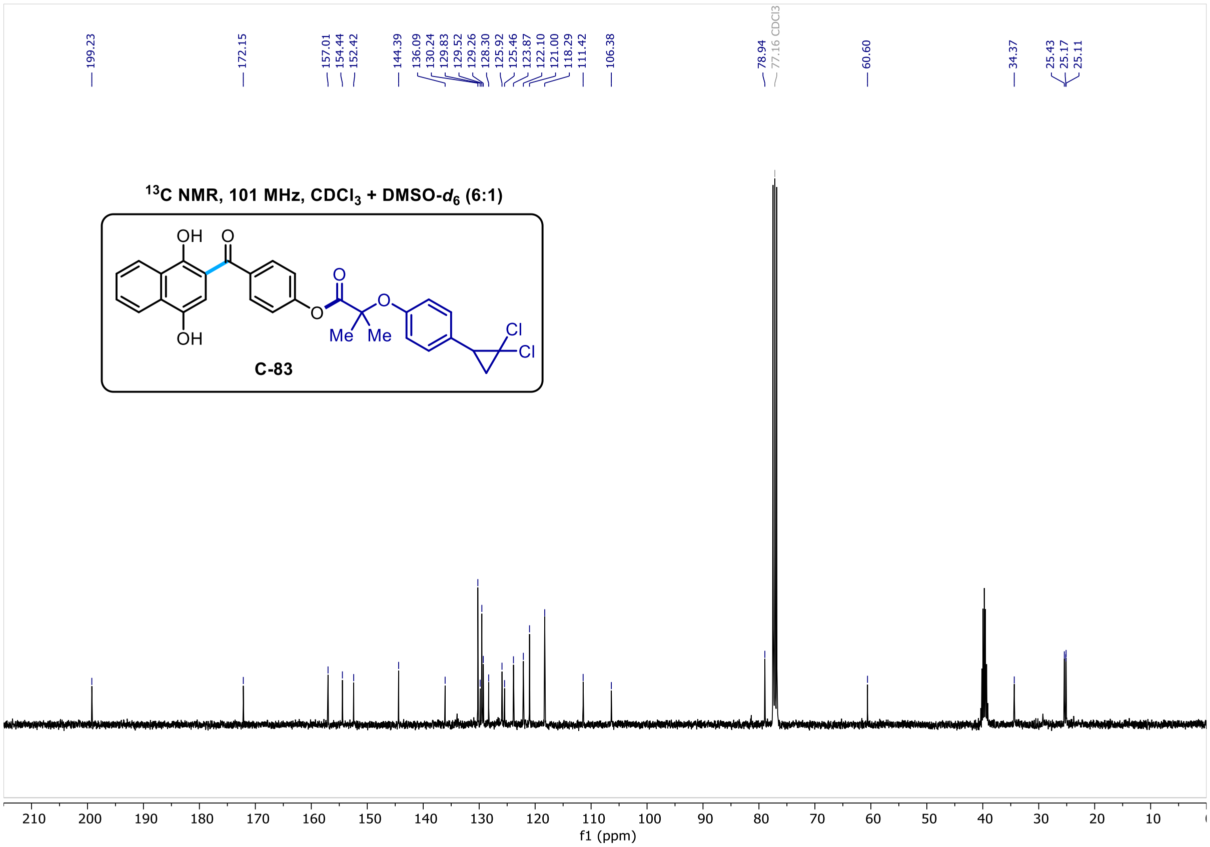
13C NMR, 101 MHz, CDCl3 + DMSO-d6 (6:1)
C-83
OH
O
OH
O
O
O
Me
Me
Cl
Cl
199.23
172.15
157.01 154.44 152.42
144.39 136.09 130.24 129.83 129.52 129.26 128.30 125.92 125.46 123.87 122.10 121.00 118.29 111.42 106.38
78.94 77.16 CDCl3
60.60
34.37
25.43 25.17 25.11
f1 (ppm)

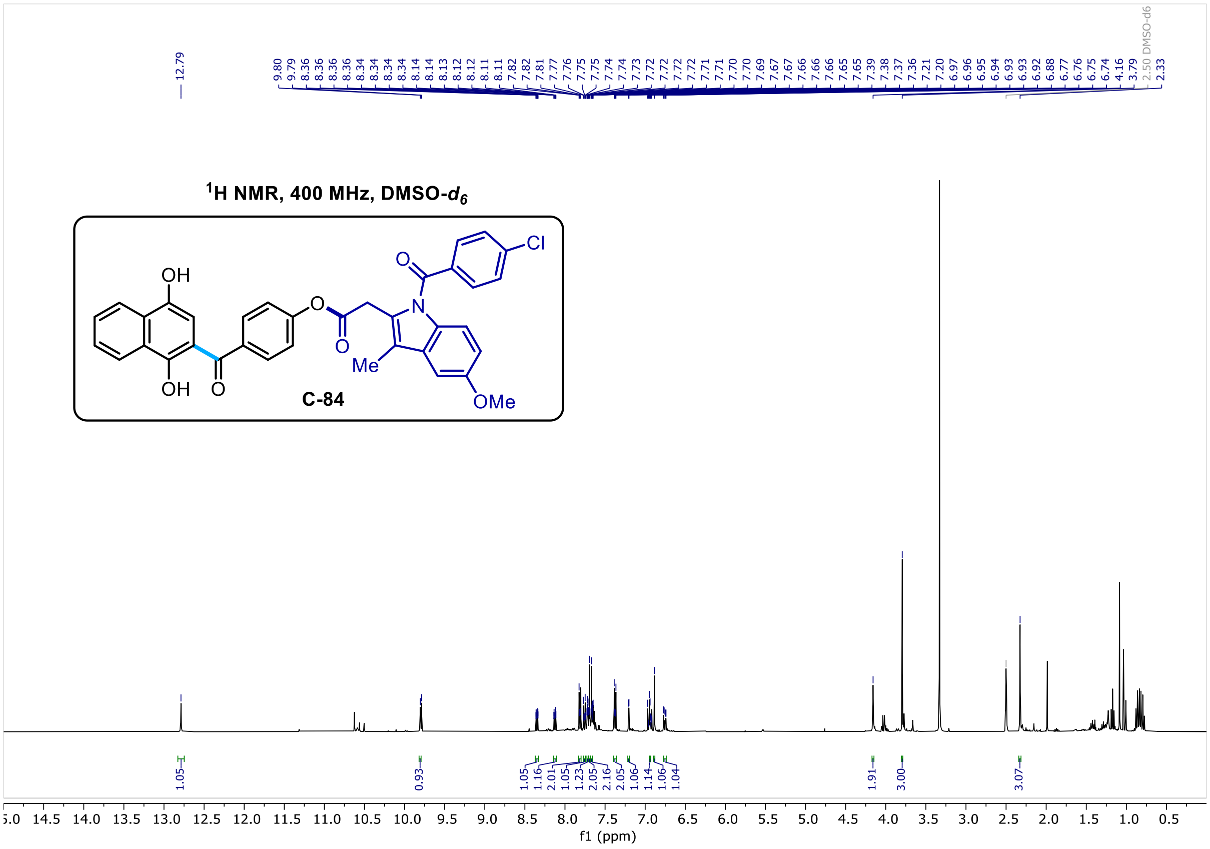

1H NMR, 400 MHz, DMSO-d6
C-84
f1 (ppm)

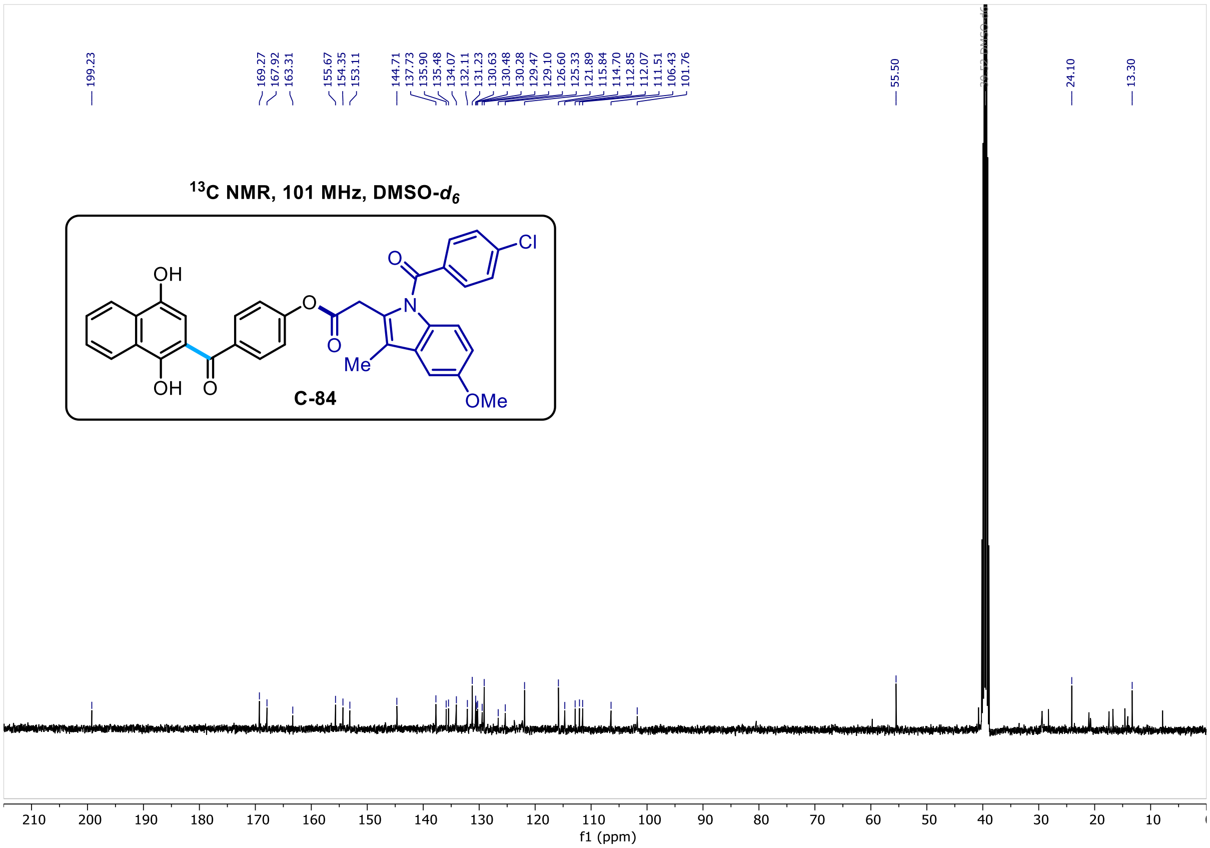

13C NMR, 101 MHz, DMSO-d6
C-84
f1 (ppm)

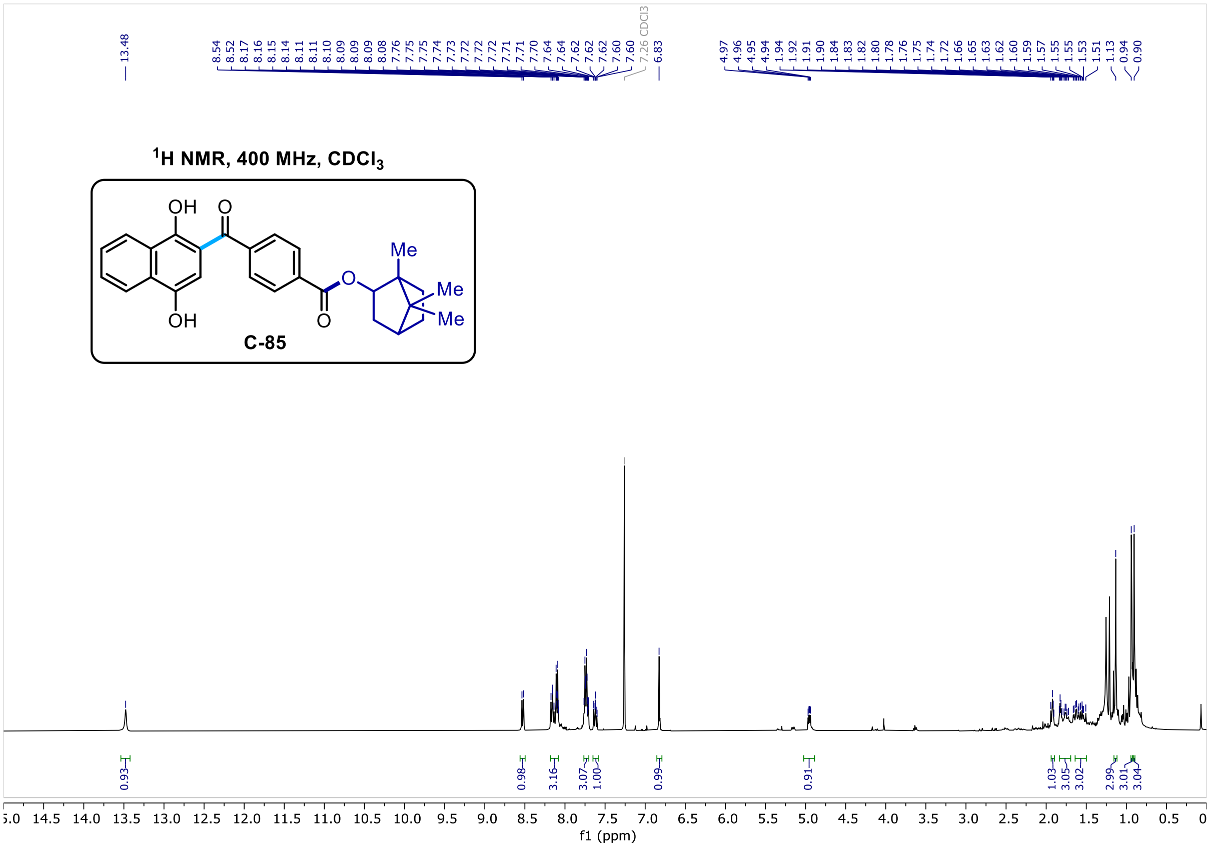

13.48
8.54 8.52 8.17 8.16 8.15 8.14 8.11 8.11 8.10 8.09 8.09 8.08 7.76 7.75 7.75 7.74 7.73 7.72 7.72 7.71 7.71 7.70 7.64 7.62 7.62 7.62 7.60 7.60
7.26 CDCl3
6.83
4.97 4.96 4.95 4.94 1.94 1.92 1.91 1.90 1.84 1.83 1.82 1.80 1.78 1.76 1.75 1.74 1.72 1.66 1.65 1.63 1.62 1.60 1.59 1.57 1.55 1.55 1.53 1.51 1.13 0.94 0.90
^{1}H NMR, 400 MHz, CDCl$_3$
OH
O
Me
Me
Me
OH
C-85
0.93 0.98 3.16 3.07 1.00 0.99 0.91 1.03 3.05 3.02 2.99 3.01 3.04
f1 (ppm)

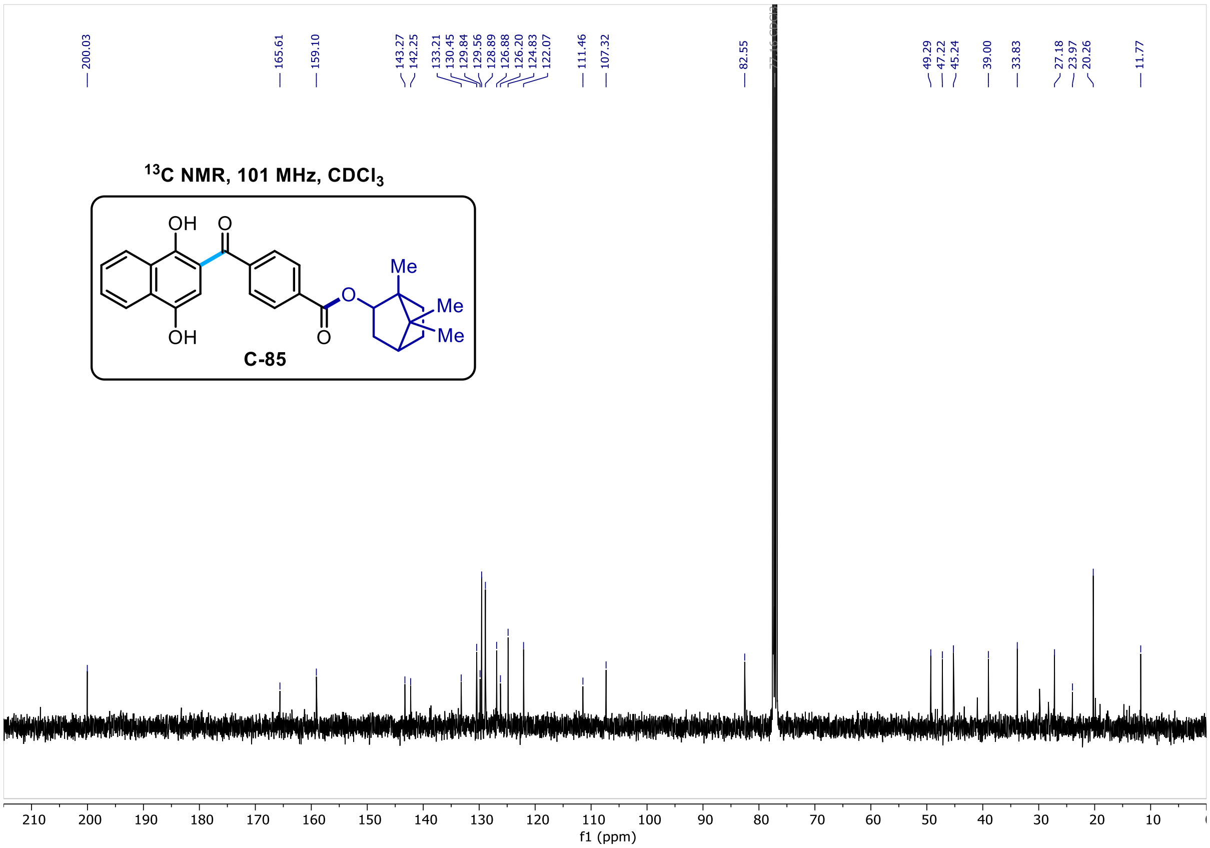

200.03
165.61
159.10
143.27 142.25
133.21 130.45 129.84 129.56 128.89 126.88 126.20 124.83 122.07
111.46
107.32
82.55
49.29 47.22 45.24
39.00
33.83
27.18 23.97 20.26
11.77
^{13}C NMR, 101 MHz, CDCl$_3$
OH
O
Me
Me
Me
OH
C-85
f1 (ppm)

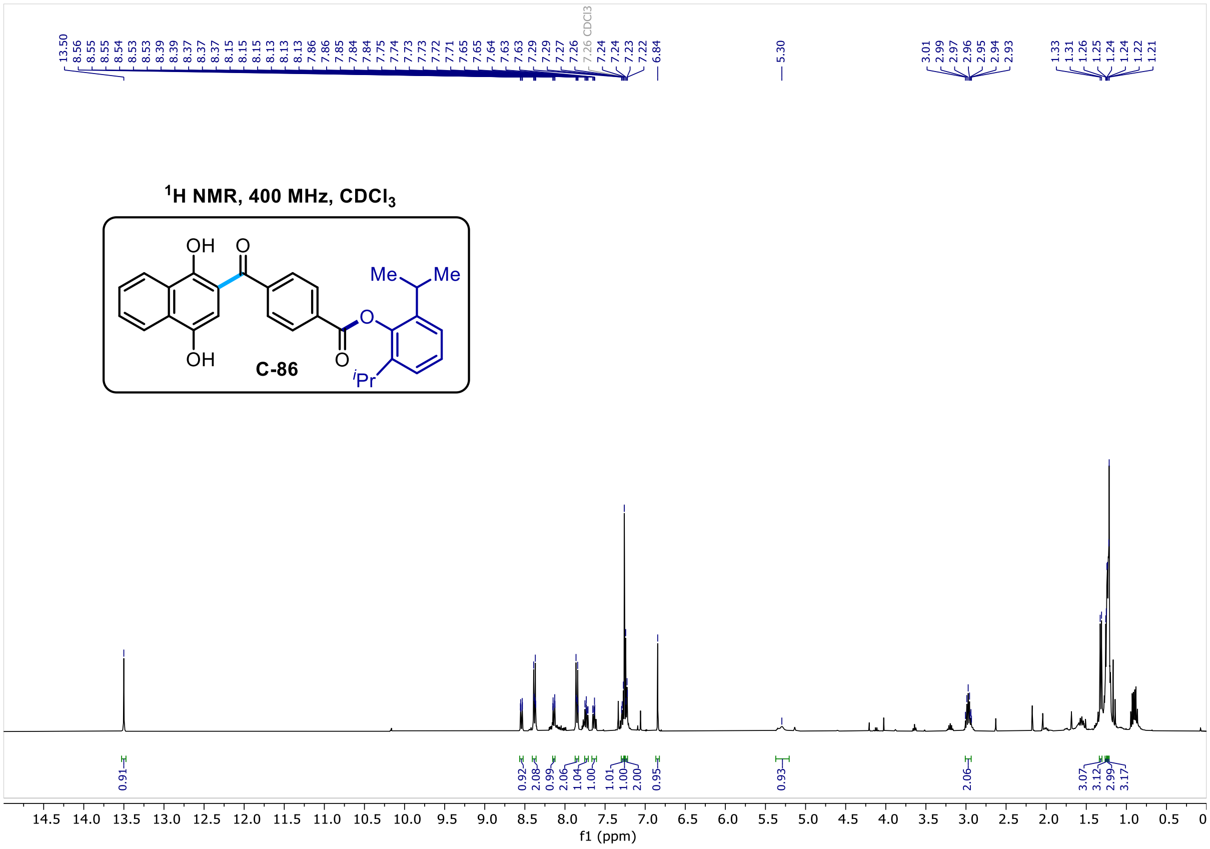

1H NMR, 400 MHz, CDCl3
C-86
f1 (ppm)

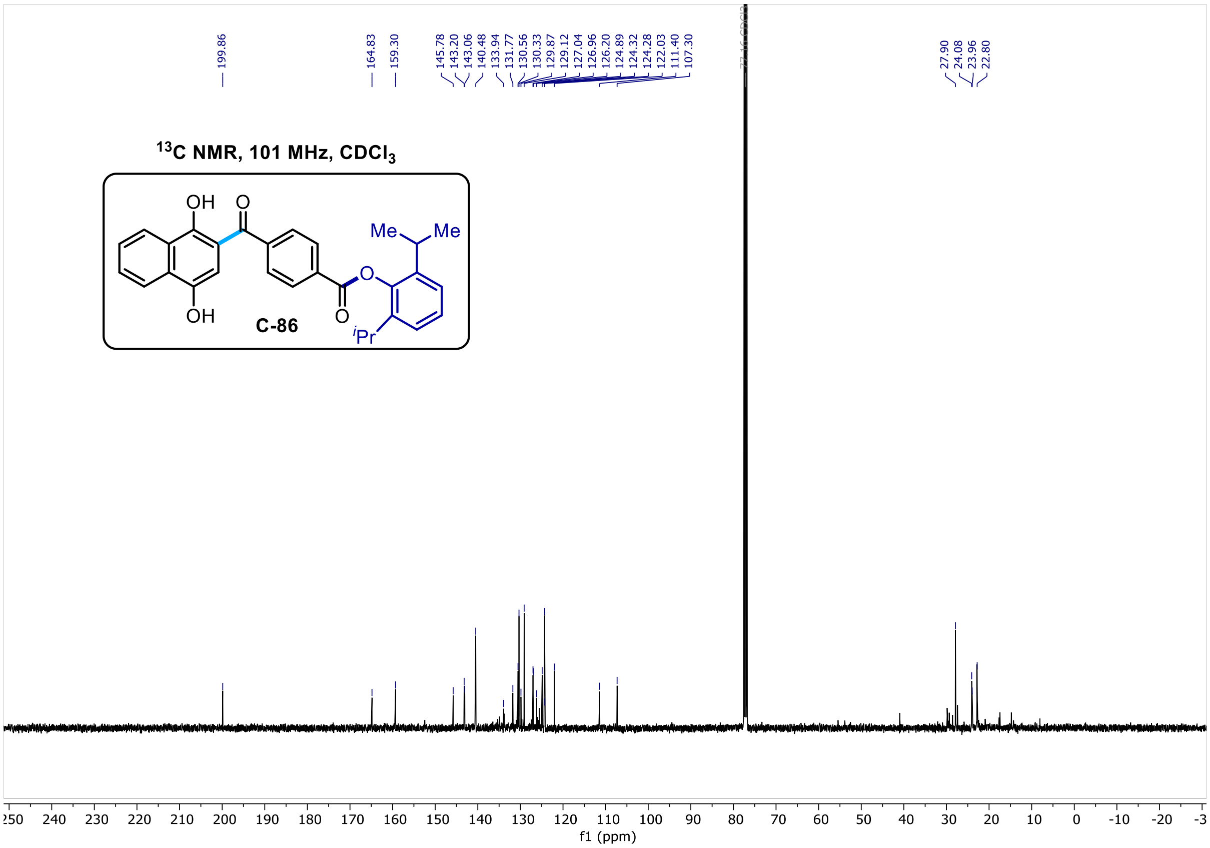

13C NMR, 101 MHz, CDCl3
C-86
f1 (ppm)

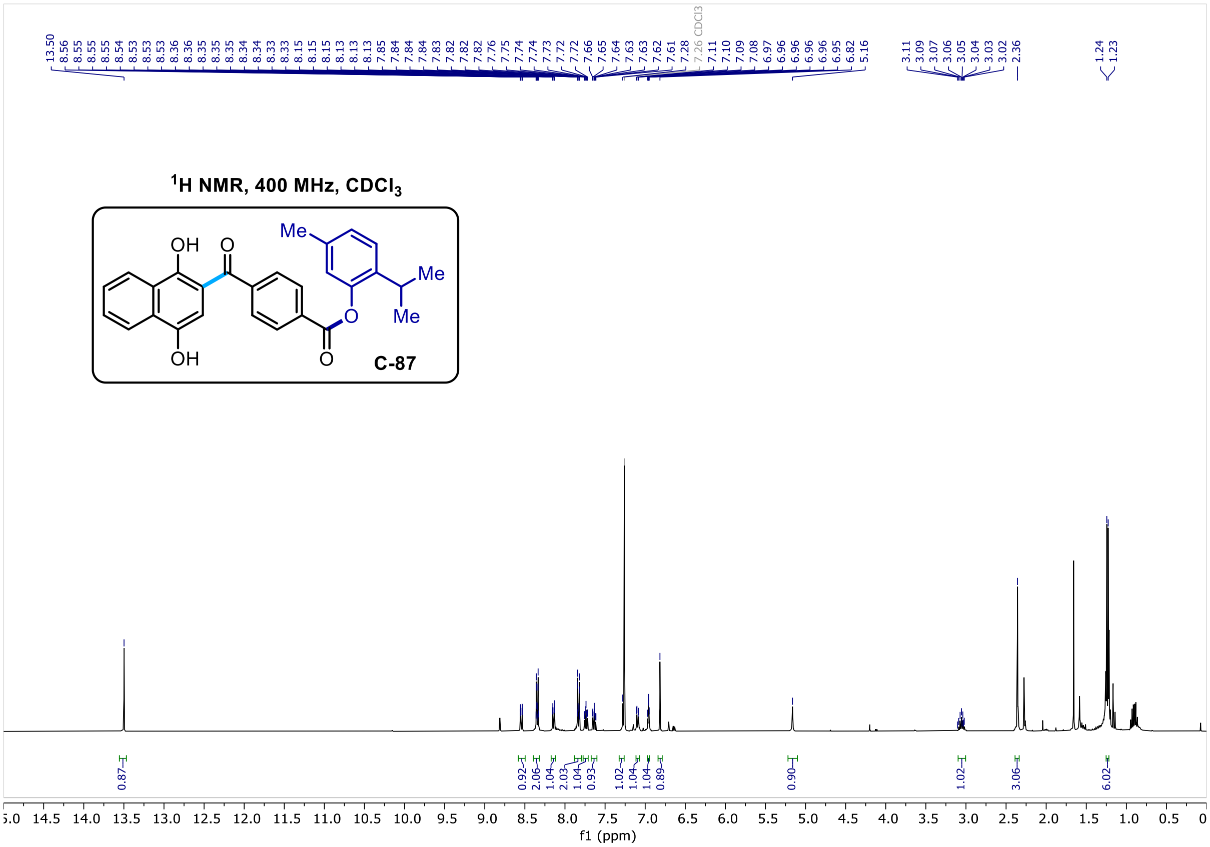

1H NMR, 400 MHz, CDCl3
OH
O
Me
Me
Me
O
O
OH
C-87
f1 (ppm)

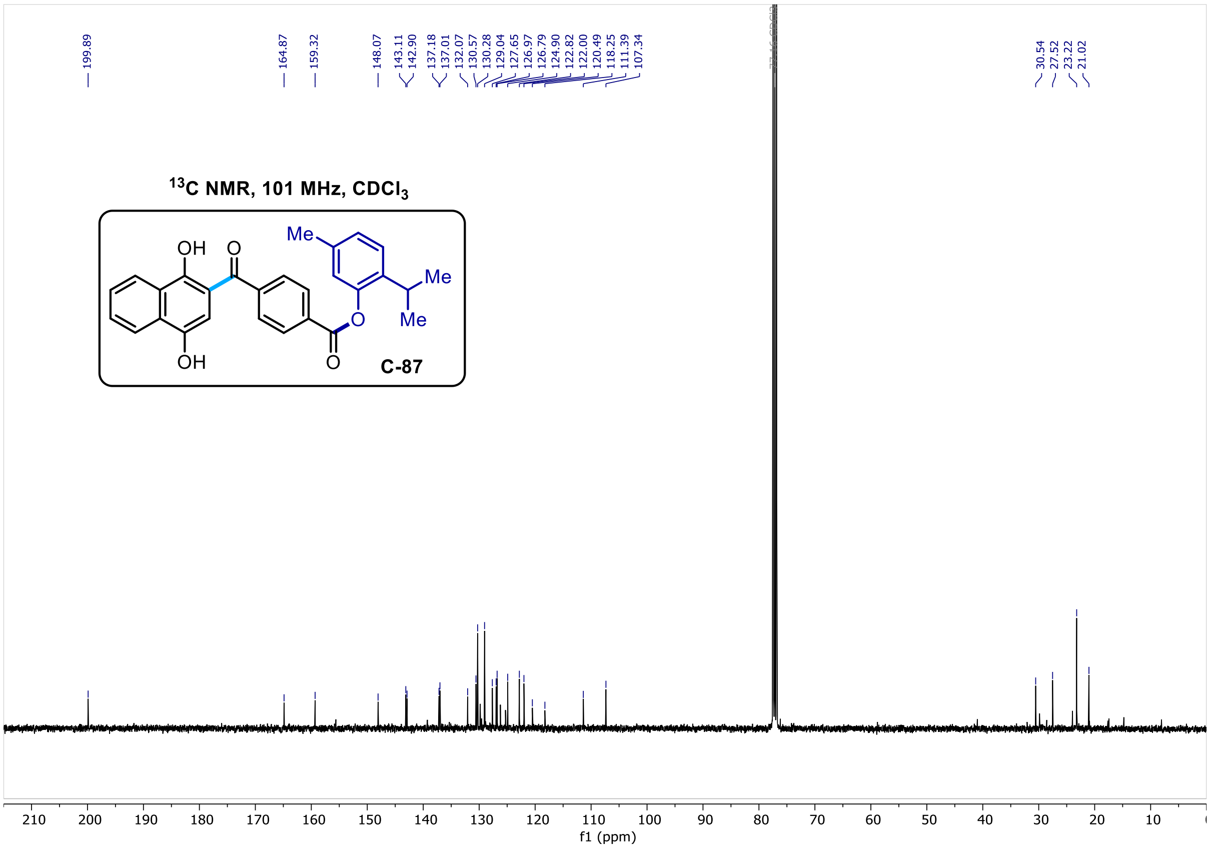

13C NMR, 101 MHz, CDCl3
OH
O
Me
Me
Me
O
O
OH
C-87
f1 (ppm)

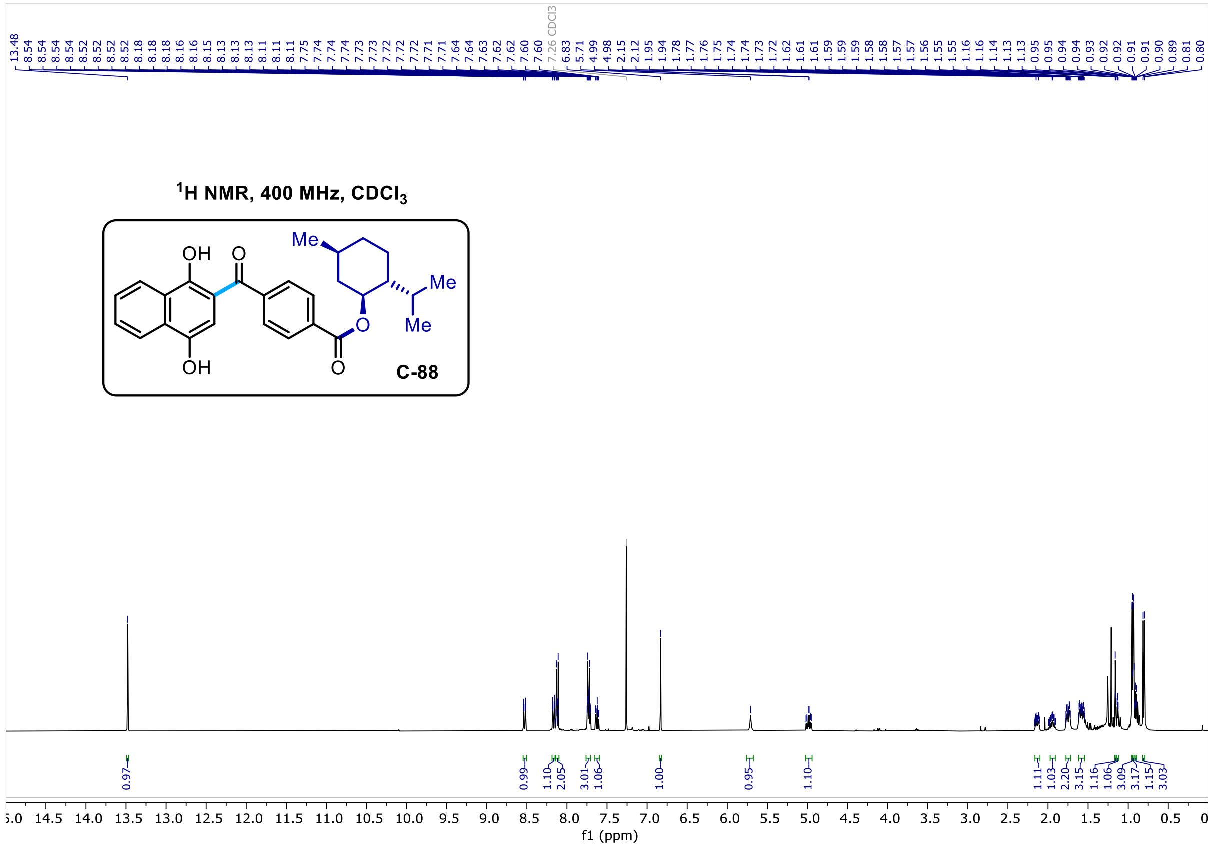
13.48
8.54
8.54
8.54
8.54
8.52
8.52
8.52
8.52
8.18
8.18
8.18
8.16
8.16
8.15
8.13
8.13
8.13
8.11
8.11
8.11
7.75
7.74
7.74
7.74
7.73
7.73
7.73
7.72
7.72
7.72
7.71
7.71
7.64
7.63
7.62
7.62
7.60
7.60
7.26 CDCl3
6.83
5.71
4.99
4.98
2.15
2.12
1.95
1.94
1.78
1.77
1.76
1.75
1.74
1.74
1.73
1.72
1.62
1.61
1.61
1.59
1.59
1.59
1.58
1.58
1.57
1.56
1.55
1.55
1.16
1.16
1.14
1.13
1.13
0.95
0.95
0.94
0.94
0.93
0.92
0.92
0.91
0.91
0.90
0.89
0.81
0.80
1H NMR, 400 MHz, CDCl3
OH
O
Me
Me
O
Me
OH
O
C-88
0.97
0.99
1.10
2.05
3.01
1.06
1.00
0.95
1.10
1.11
1.03
2.20
3.15
1.16
1.06
3.09
3.17
1.15
3.03
f1 (ppm)

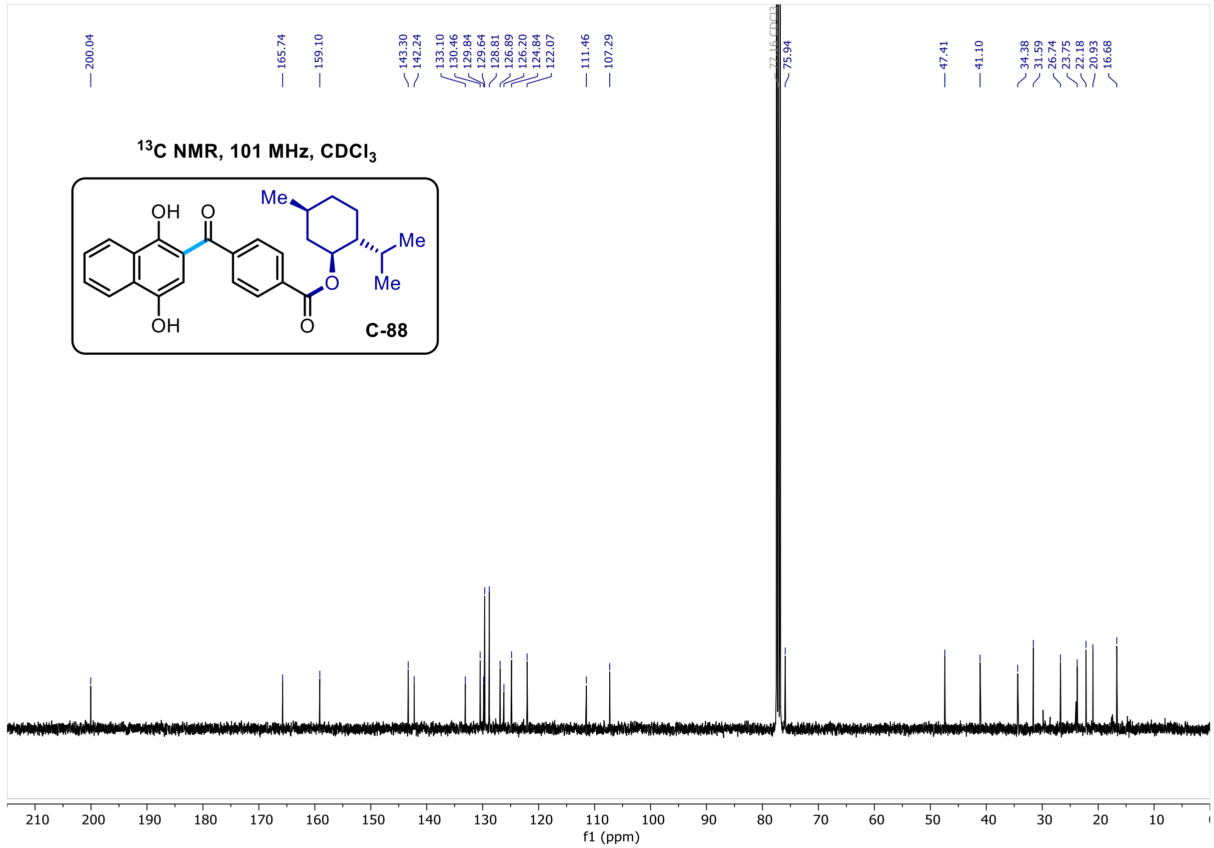
200.04
165.74
159.10
143.30
142.24
133.10
130.46
129.84
129.64
128.81
126.89
126.20
124.84
122.07
111.46
107.29
77.16 CDCl3
75.94
47.41
41.10
34.38
31.59
26.74
23.75
22.18
20.93
16.68
13C NMR, 101 MHz, CDCl3
OH
O
Me
Me
O
Me
OH
O
C-88
f1 (ppm)

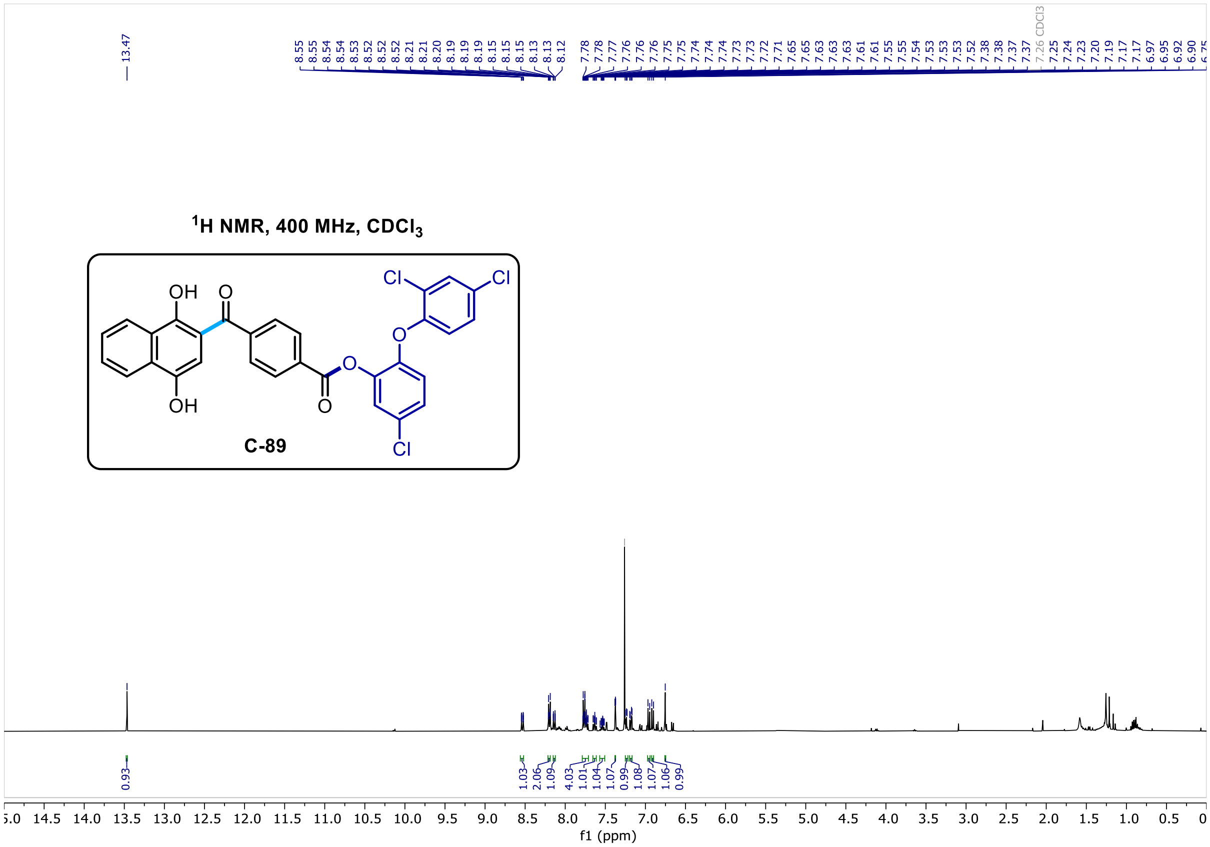

1H NMR, 400 MHz, CDCl3
C-89
f1 (ppm)

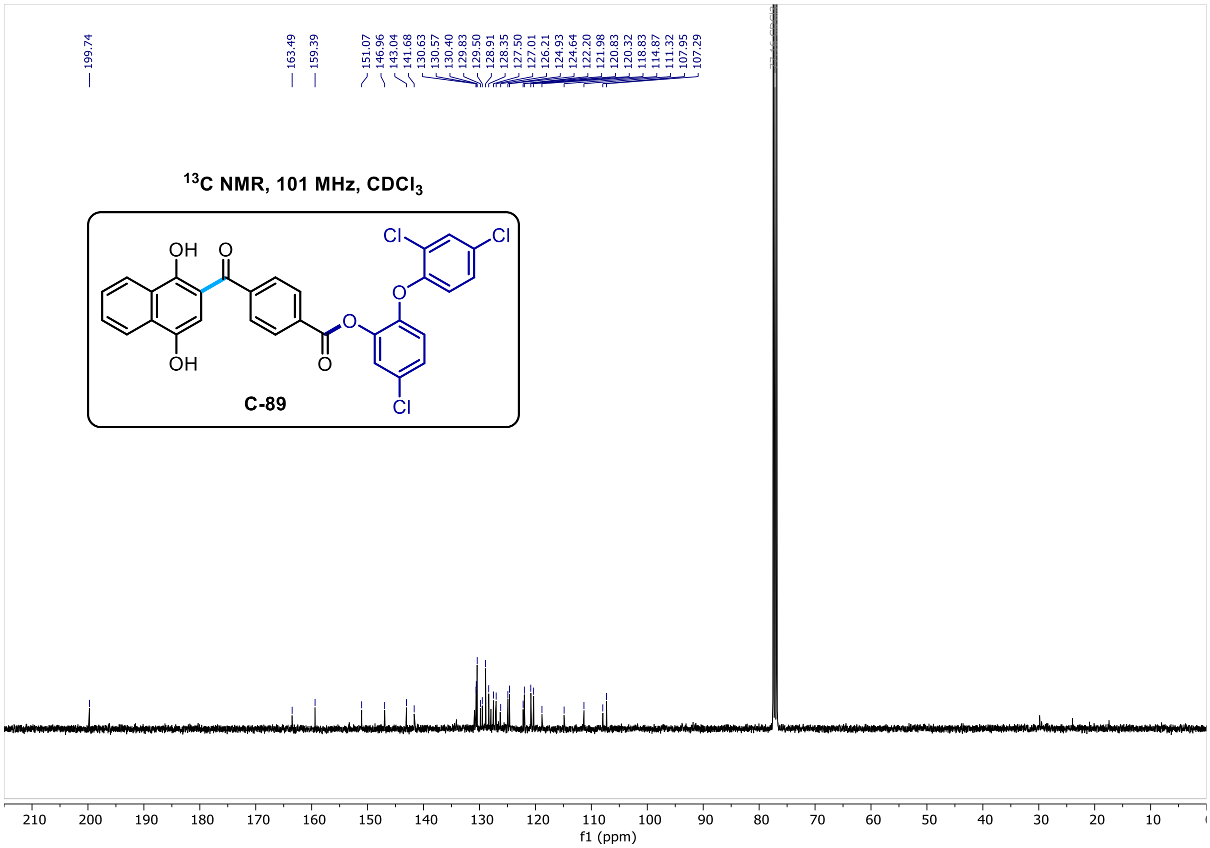

13C NMR, 101 MHz, CDCl3
C-89
f1 (ppm)

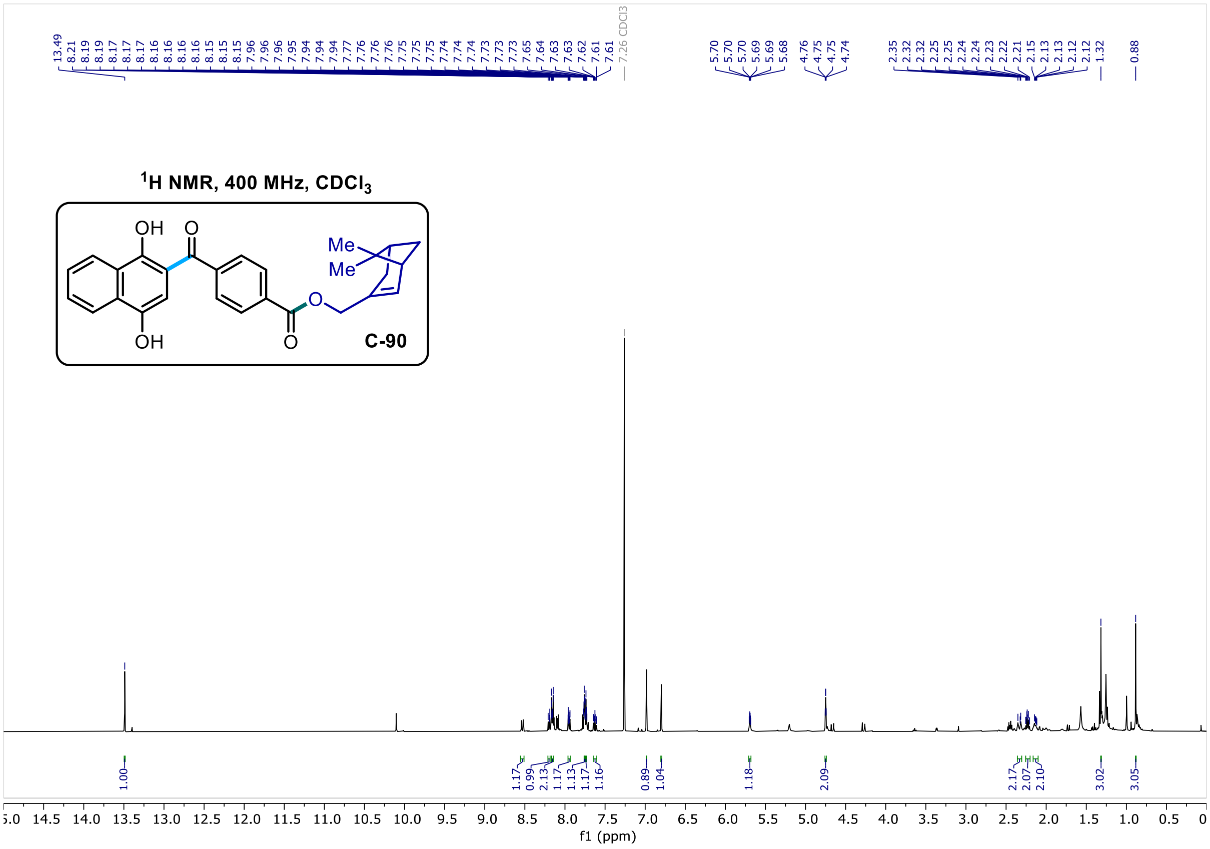

13.49 8.21 8.19 8.19 8.17 8.17 8.17 8.16 8.16 8.16 8.15 8.15 7.96 7.96 7.95 7.94 7.94 7.94 7.77 7.76 7.76 7.75 7.75 7.74 7.74 7.73 7.73 7.73 7.65 7.64 7.63 7.63 7.62 7.61 7.61
7.26 CDCl3
5.70 5.70 5.70 5.69 5.69 5.68
4.76 4.75 4.75 4.74
2.35 2.32 2.32 2.25 2.25 2.24 2.24 2.23 2.22 2.21 2.15 2.13 2.13 2.12 2.12
1.32
0.88
1H NMR, 400 MHz, CDCl3
OH
O
OH
Me
Me
O
O
C-90
1.00
1.17 0.99 2.13 1.17 1.13 1.17 1.16
0.89 1.04
1.18
2.09
2.17 2.07 2.10
3.02
3.05
f1 (ppm)

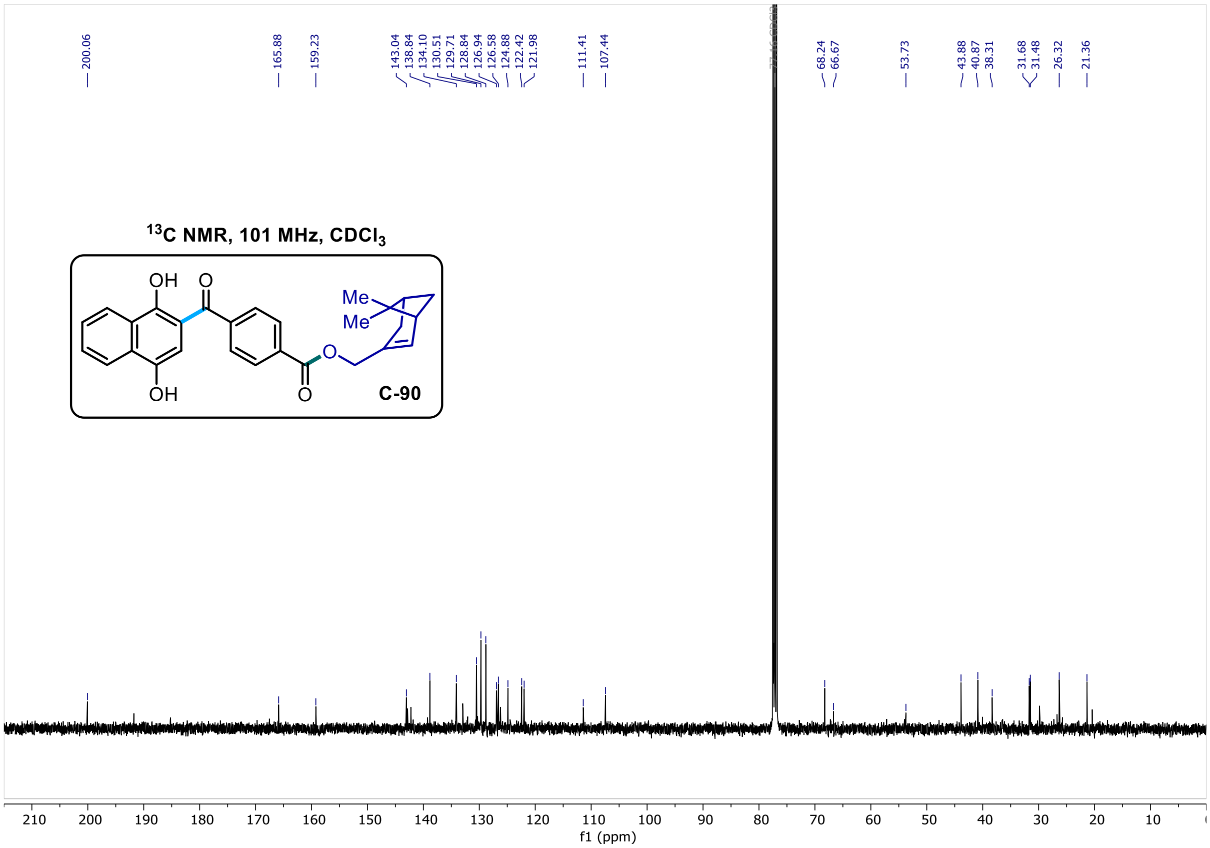

200.06
165.88
159.23
143.04 138.84 134.10 130.51 129.71 128.84 126.94 126.58 124.88 122.42 121.98
111.41 107.44
68.24 66.67
53.73
43.88 40.87 38.31
31.68 31.48 26.32 21.36
13C NMR, 101 MHz, CDCl3
OH
O
OH
Me
Me
O
O
C-90
f1 (ppm)

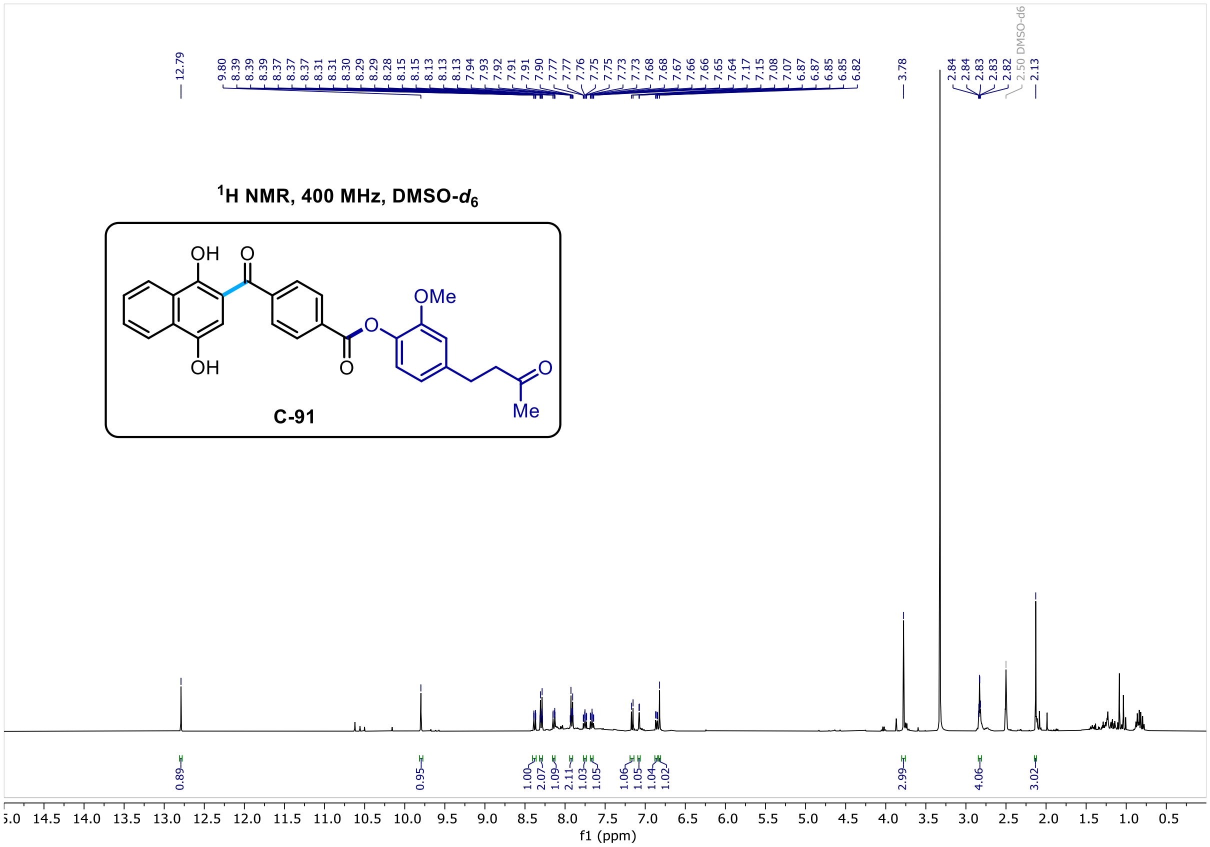
1H NMR, 400 MHz, DMSO-d6
OH
O
OMe
O
O
OH
O
Me
C-91
12.79
9.80 8.39 8.39 8.39 8.37 8.37 8.37 8.31 8.31 8.30 8.29 8.29 8.28 8.15 8.15 8.13 8.13 8.13 7.94 7.93 7.92 7.91 7.91 7.90 7.77 7.77 7.76 7.75 7.75 7.73 7.73 7.68 7.67 7.66 7.65 7.64 7.17 7.15 7.08 7.07 6.87 6.87 6.85 6.85 6.82
3.78
2.84 2.84 2.83 2.83 2.82
2.50 DMSO-d6
2.13
0.89 0.95 1.00 2.07 1.09 2.11 1.03 1.05 1.06 1.05 1.04 1.02 2.99 4.06 3.02
f1 (ppm)

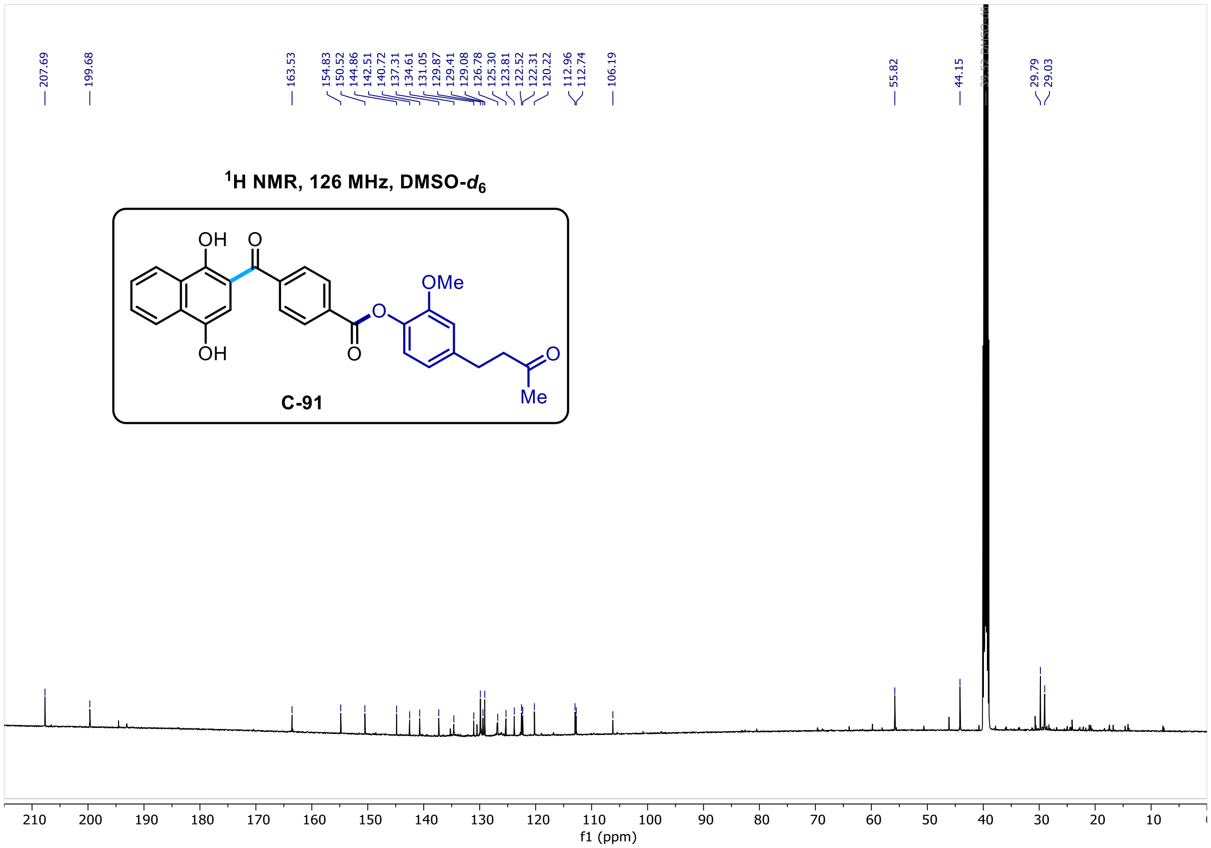
1H NMR, 126 MHz, DMSO-d6
OH
O
OMe
O
O
OH
O
Me
C-91
207.69
199.68
163.53
154.83 150.52 144.86 142.51 140.72 137.31 134.61 131.05 129.87 129.41 129.08 126.78 125.30 123.81 122.52 122.31 120.22
112.96 112.74
106.19
55.82
44.15
29.79 29.03
f1 (ppm)

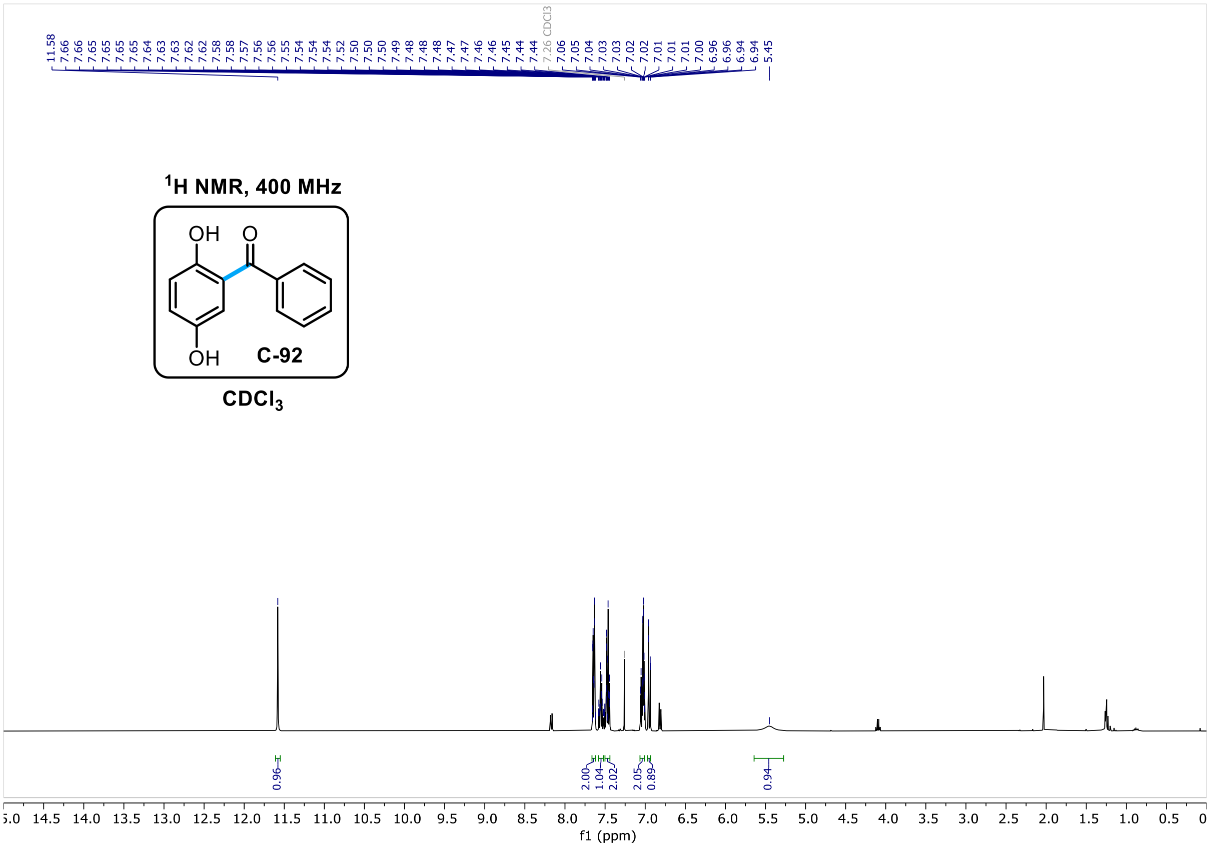

11.58
7.66
7.65
7.64
7.63
7.62
7.58
7.57
7.56
7.55
7.54
7.52
7.50
7.49
7.48
7.47
7.46
7.45
7.44
7.26 CDCl3
7.06
7.05
7.04
7.03
7.02
7.01
7.00
6.96
6.94
5.45
^1H NMR, 400 MHz
OH
O
OH
C-92
$CDCl_3$
0.96
2.00
1.04
2.02
2.05
0.89
0.94
f1 (ppm)

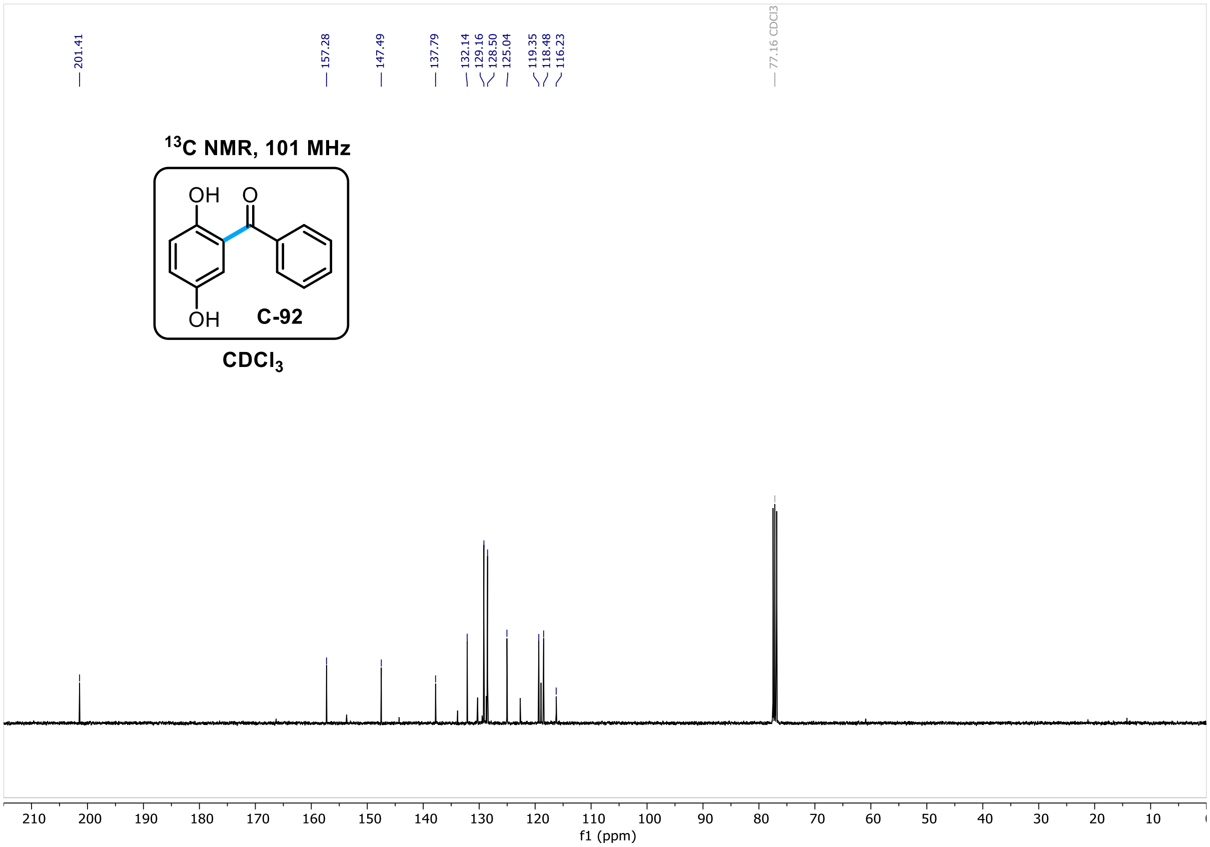

201.41
157.28
147.49
137.79
132.14
129.16
128.50
125.04
119.35
118.48
116.23
77.16 CDCl3
^{13}C NMR, 101 MHz
OH
O
OH
C-92
$CDCl_3$
f1 (ppm)

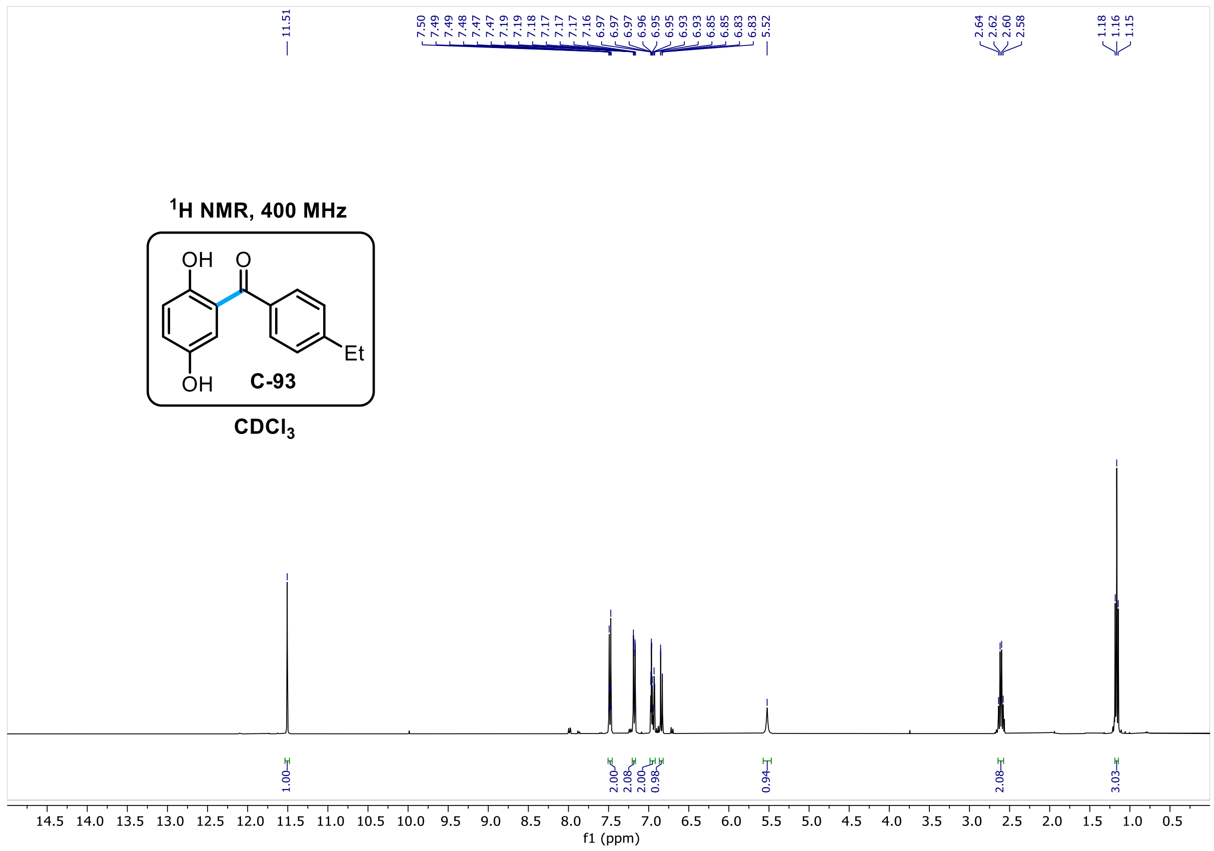
11.51
7.50
7.49
7.49
7.48
7.47
7.47
7.19
7.19
7.18
7.17
7.17
7.17
7.16
6.97
6.97
6.96
6.95
6.95
6.93
6.93
6.85
6.85
6.83
6.83
5.52
2.64
2.62
2.60
2.58
1.18
1.16
1.15
¹H NMR, 400 MHz
OH
O
Et
OH
C-93
CDCl3
1.00
2.00
2.08
2.00
0.98
0.94
2.08
3.03
f1 (ppm)

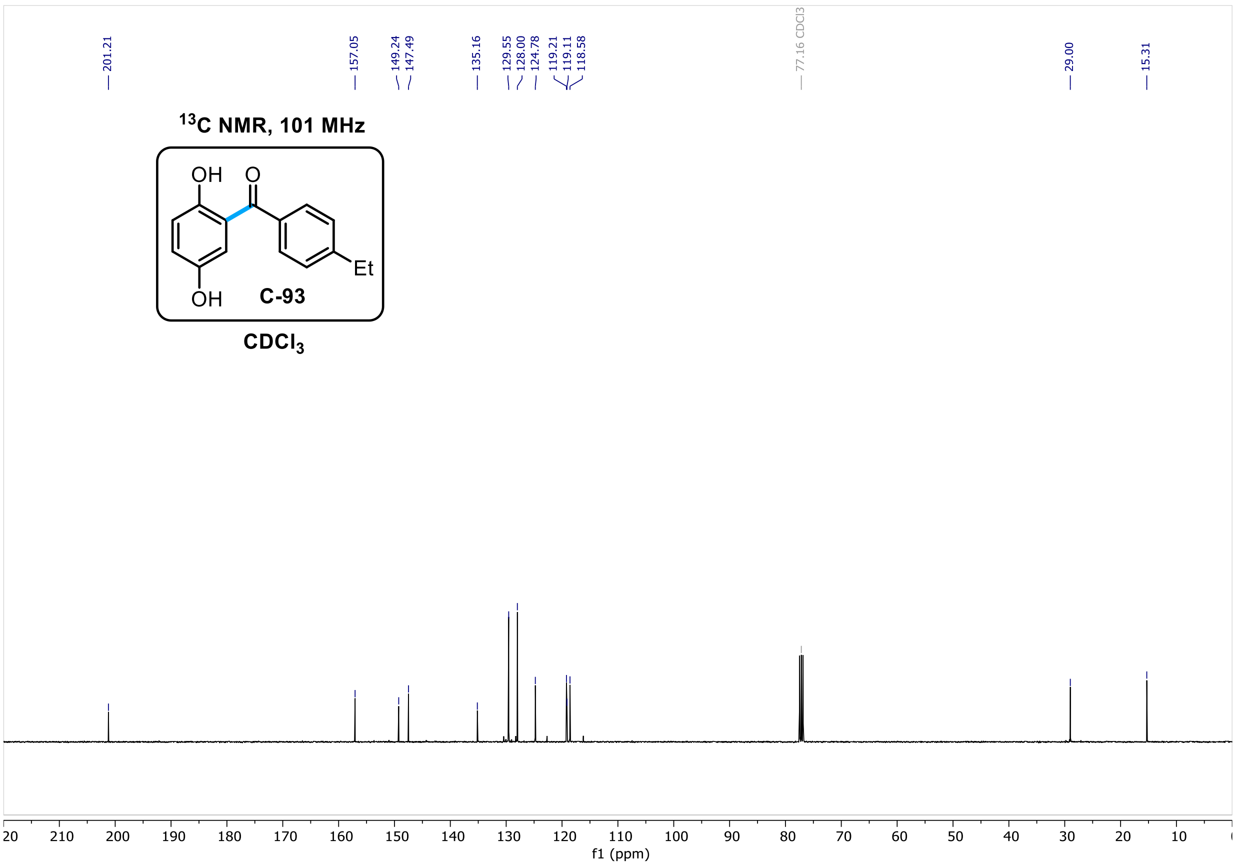
201.21
157.05
149.24
147.49
135.16
129.55
128.00
124.78
119.21
119.11
118.58
77.16 CDCl3
29.00
15.31
¹³C NMR, 101 MHz
OH
O
Et
OH
C-93
CDCl3
f1 (ppm)

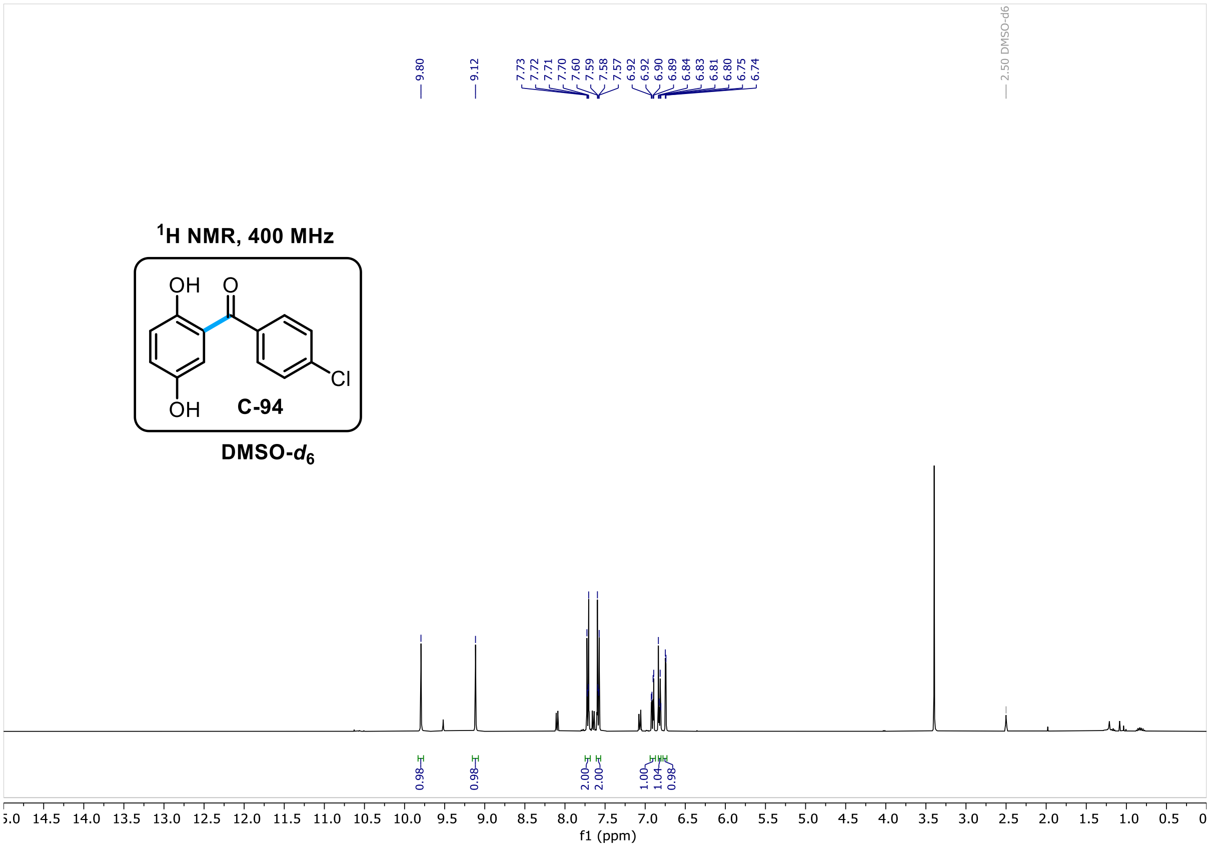

9.80
9.12
7.73
7.72
7.71
7.70
7.60
7.59
7.58
7.57
6.92
6.92
6.90
6.89
6.84
6.83
6.81
6.80
6.75
6.74
2.50 DMSO-d6
^{1}H NMR, 400 MHz
OH
O
Cl
OH
C-94
DMSO-d_6
0.98
0.98
2.00
2.00
1.00
1.04
0.98
f1 (ppm)

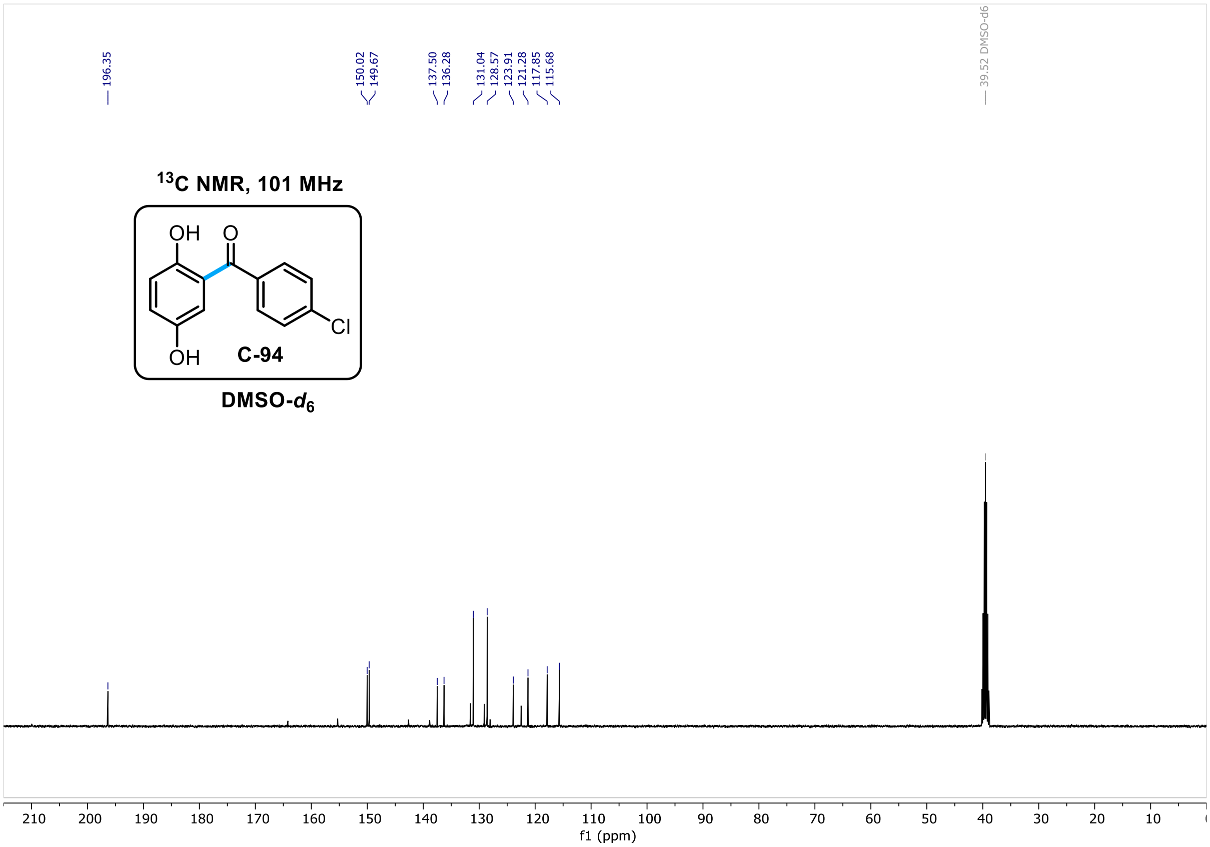

196.35
150.02
149.67
137.50
136.28
131.04
128.57
123.91
121.28
117.85
115.68
39.52 DMSO-d6
^{13}C NMR, 101 MHz
OH
O
Cl
OH
C-94
DMSO-d_6
f1 (ppm)

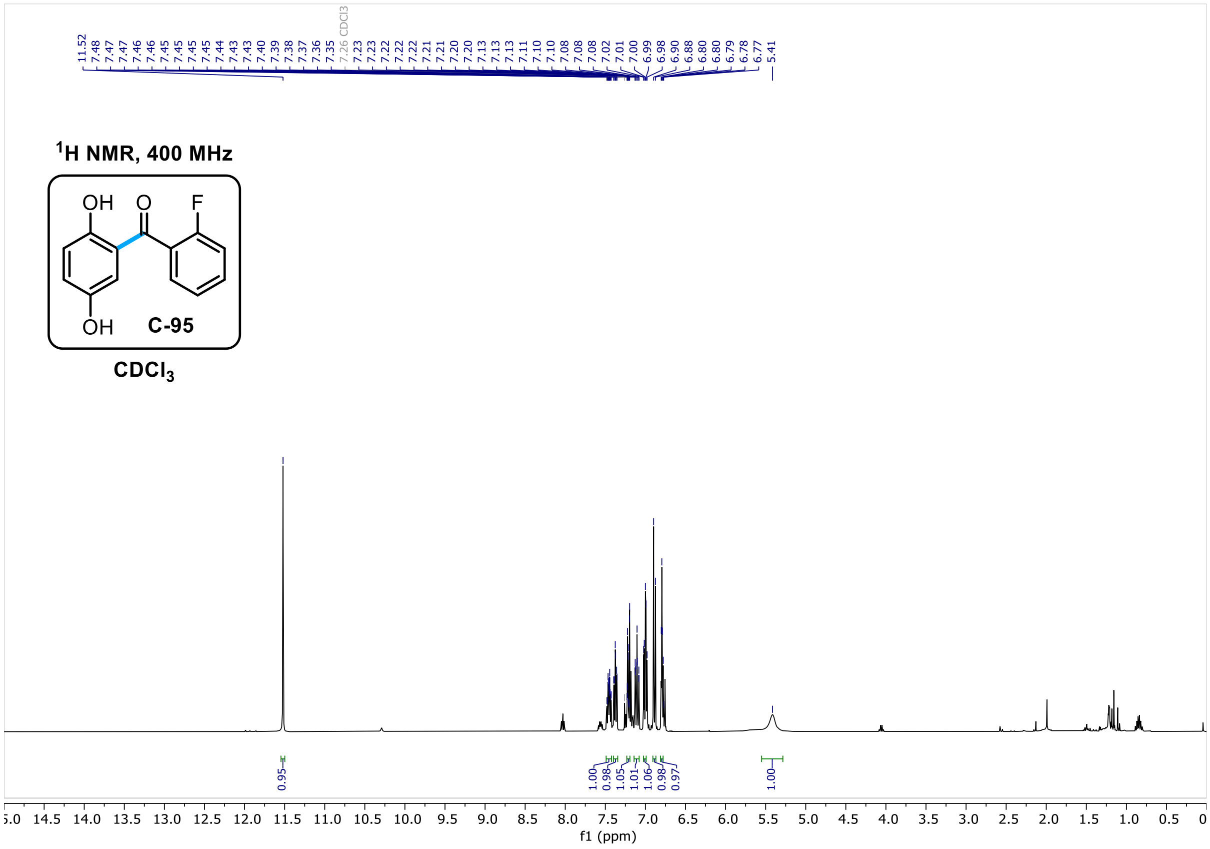
1H NMR, 400 MHz
OH
O
F
OH
C-95
CDCl3
f1 (ppm)

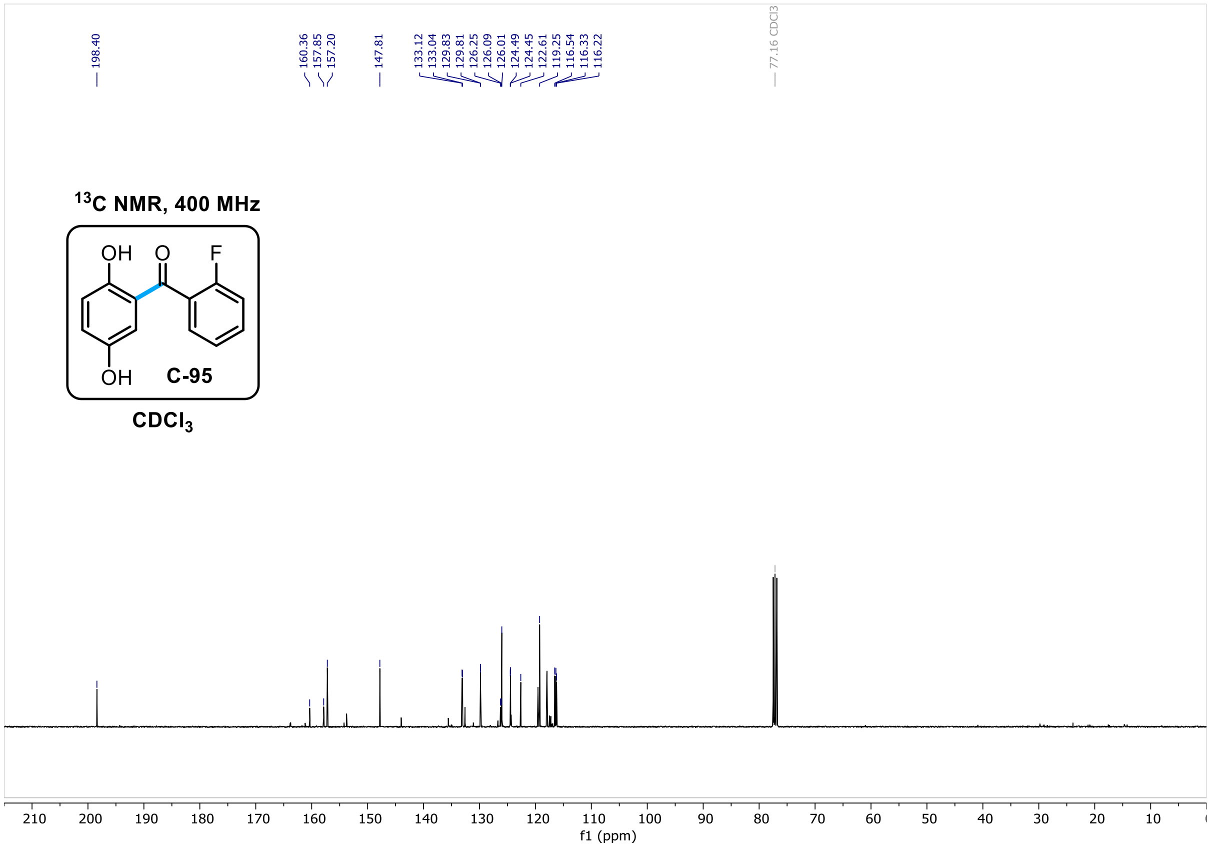
13C NMR, 400 MHz
OH
O
F
OH
C-95
CDCl3
198.40
160.36
157.85
157.20
147.81
77.16 CDCl3
f1 (ppm)

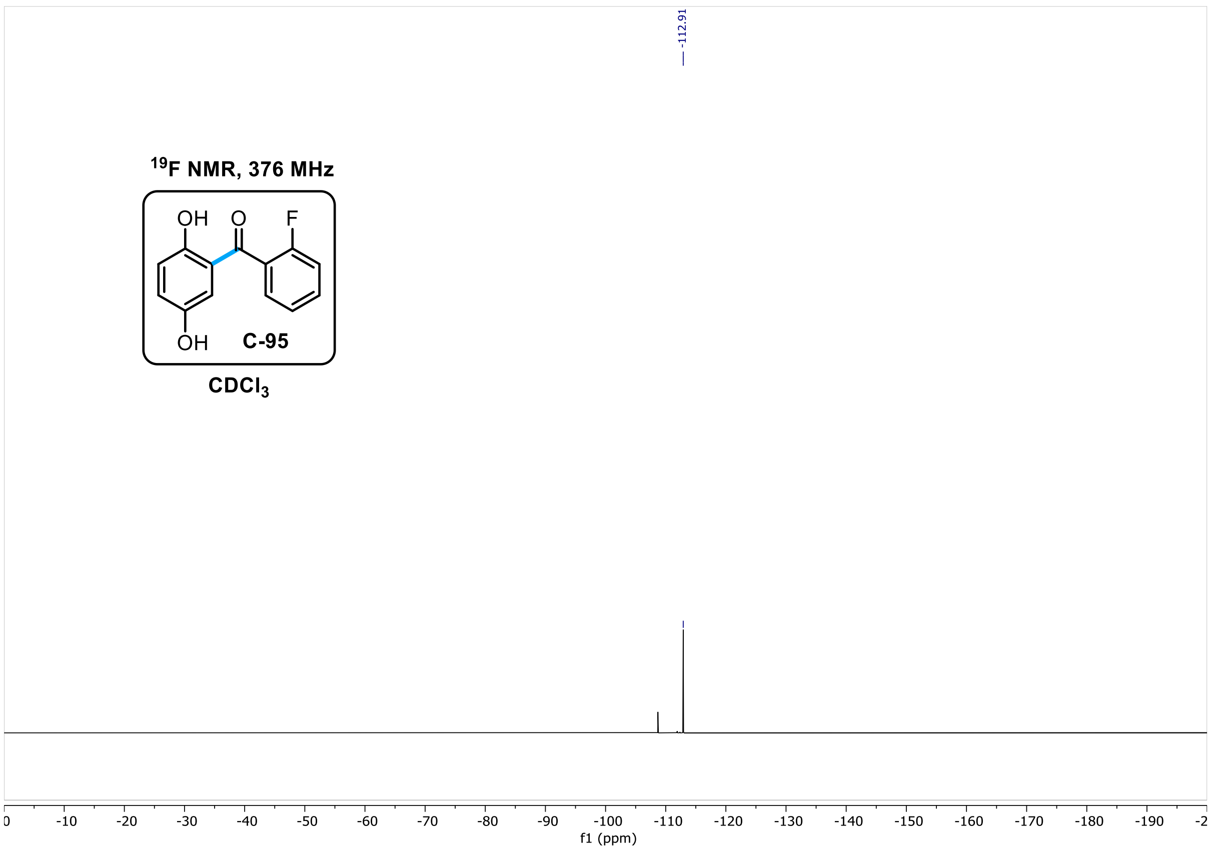
-112.91
19F NMR, 376 MHz
OH
O
F
OH
C-95
CDCl3
0 -10 -20 -30 -40 -50 -60 -70 -80 -90 -100 -110 -120 -130 -140 -150 -160 -170 -180 -190 -2
f1 (ppm)

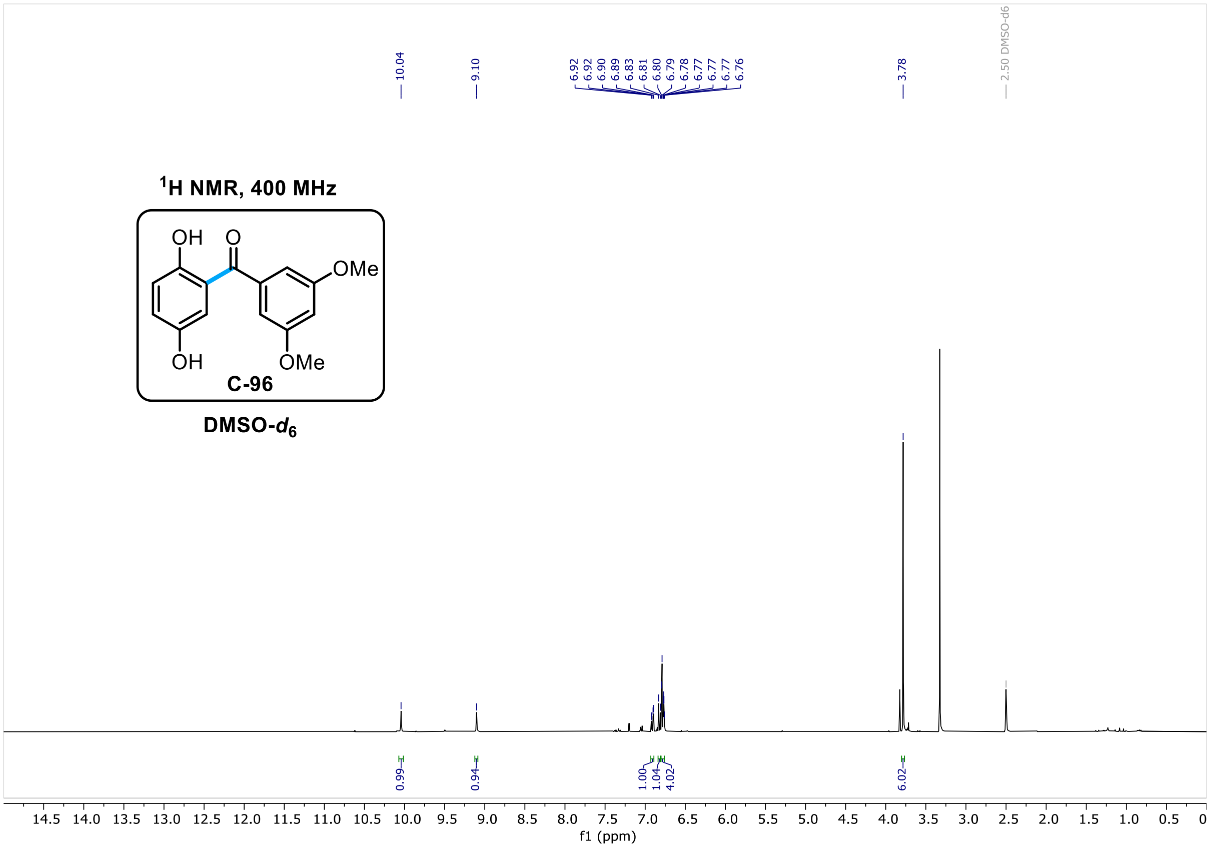

10.04
9.10
6.92
6.92
6.90
6.89
6.83
6.81
6.80
6.79
6.78
6.77
6.77
6.77
6.76
3.78
2.50 DMSO-d6
1H NMR, 400 MHz
OH
O
OMe
OH
OMe
C-96
DMSO-d6
0.99
0.94
1.00
1.04
4.02
6.02
14.5 14.0 13.5 13.0 12.5 12.0 11.5 11.0 10.5 10.0 9.5 9.0 8.5 8.0 7.5 7.0 6.5 6.0 5.5 5.0 4.5 4.0 3.5 3.0 2.5 2.0 1.5 1.0 0.5 0
f1 (ppm)

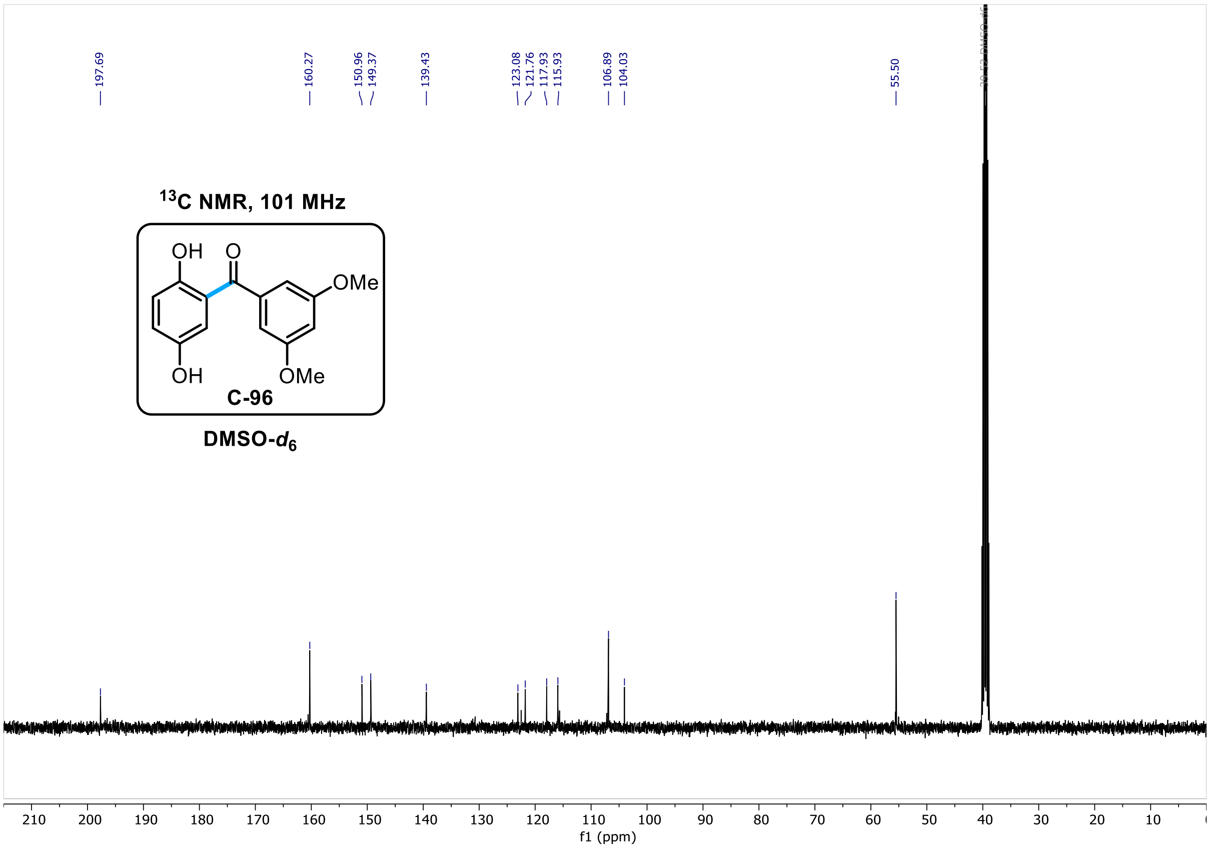

197.69
160.27
150.96
149.37
139.43
123.08
121.76
117.93
115.93
106.89
104.03
55.50
13C NMR, 101 MHz
OH
O
OMe
OH
OMe
C-96
DMSO-d6
210 200 190 180 170 160 150 140 130 120 110 100 90 80 70 60 50 40 30 20 10
f1 (ppm)

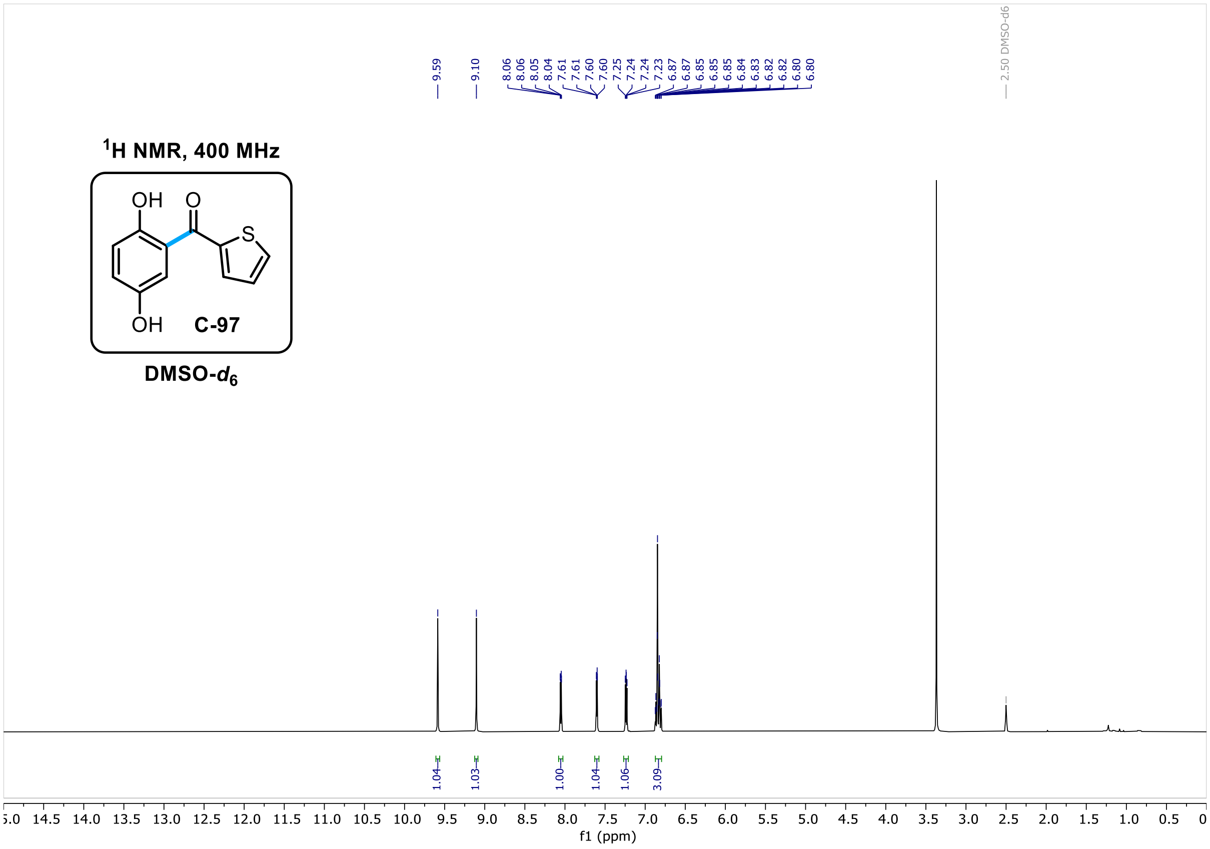

9.59
9.10
8.06
8.06
8.05
8.04
7.61
7.61
7.60
7.60
7.25
7.24
7.24
7.23
6.87
6.87
6.85
6.85
6.84
6.83
6.82
6.80
6.80
2.50 DMSO-d6
^{1}H NMR, 400 MHz
OH
O
S
OH
C-97
DMSO-d_6
1.04
1.03
1.00
1.04
1.06
3.09
f1 (ppm)

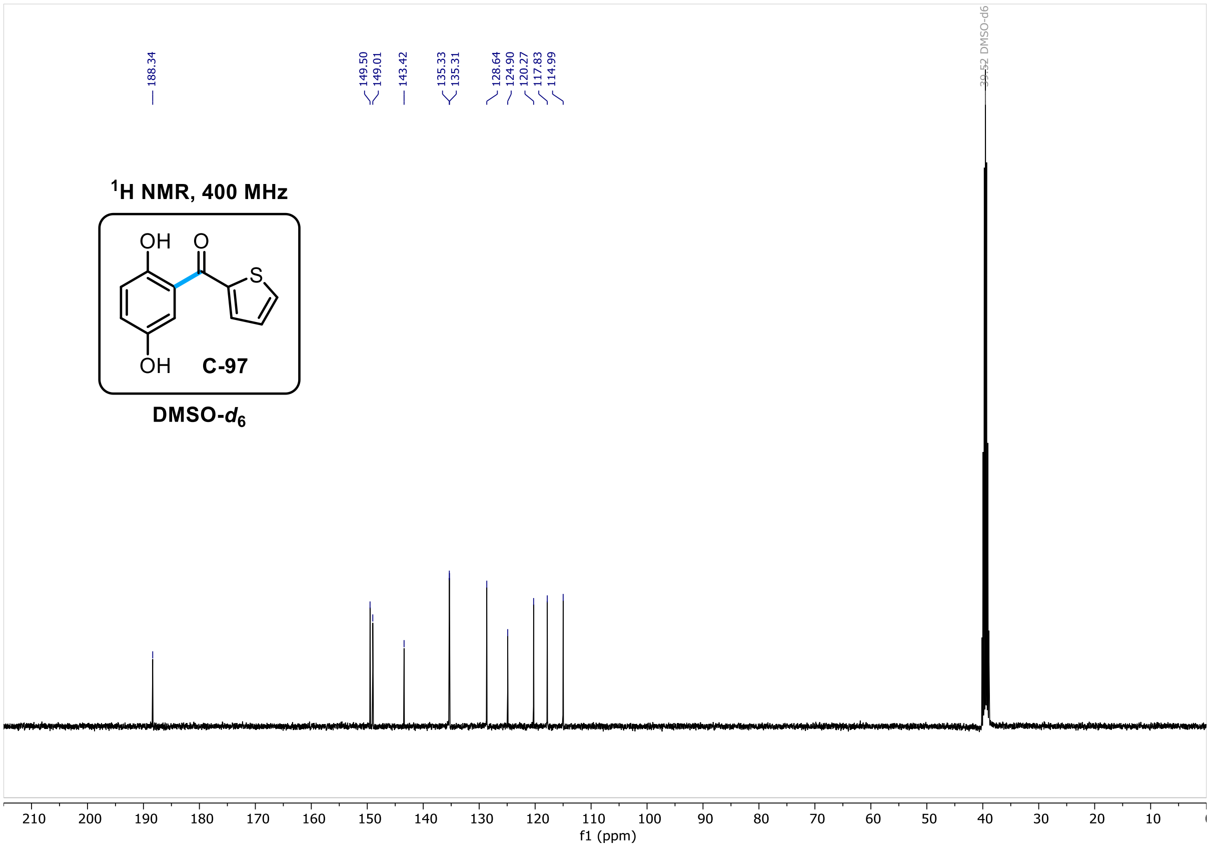

188.34
149.50
149.01
143.42
135.33
135.31
128.64
124.90
120.27
117.83
114.99
39.52 DMSO-d6
^{1}H NMR, 400 MHz
OH
O
S
OH
C-97
DMSO-d_6
f1 (ppm)

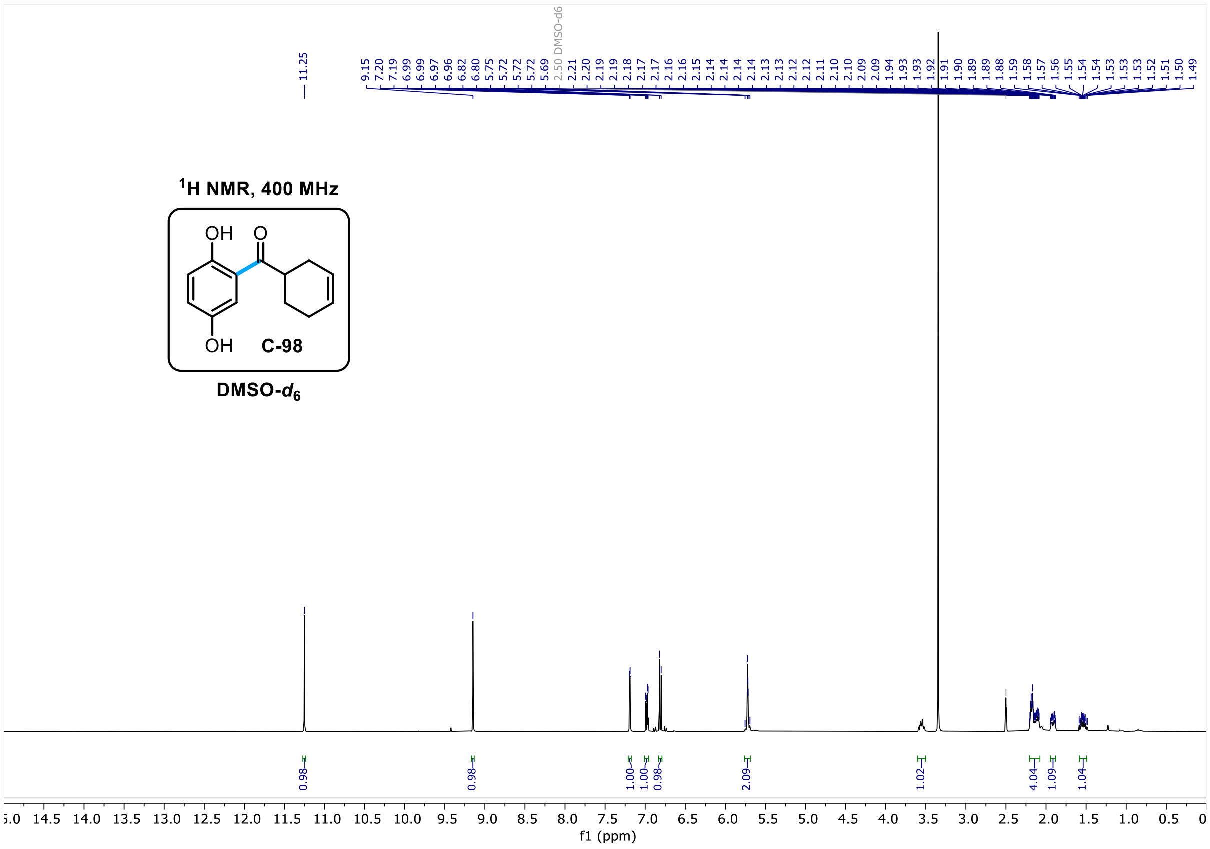

1H NMR, 400 MHz
C-98
DMSO-d6
f1 (ppm)

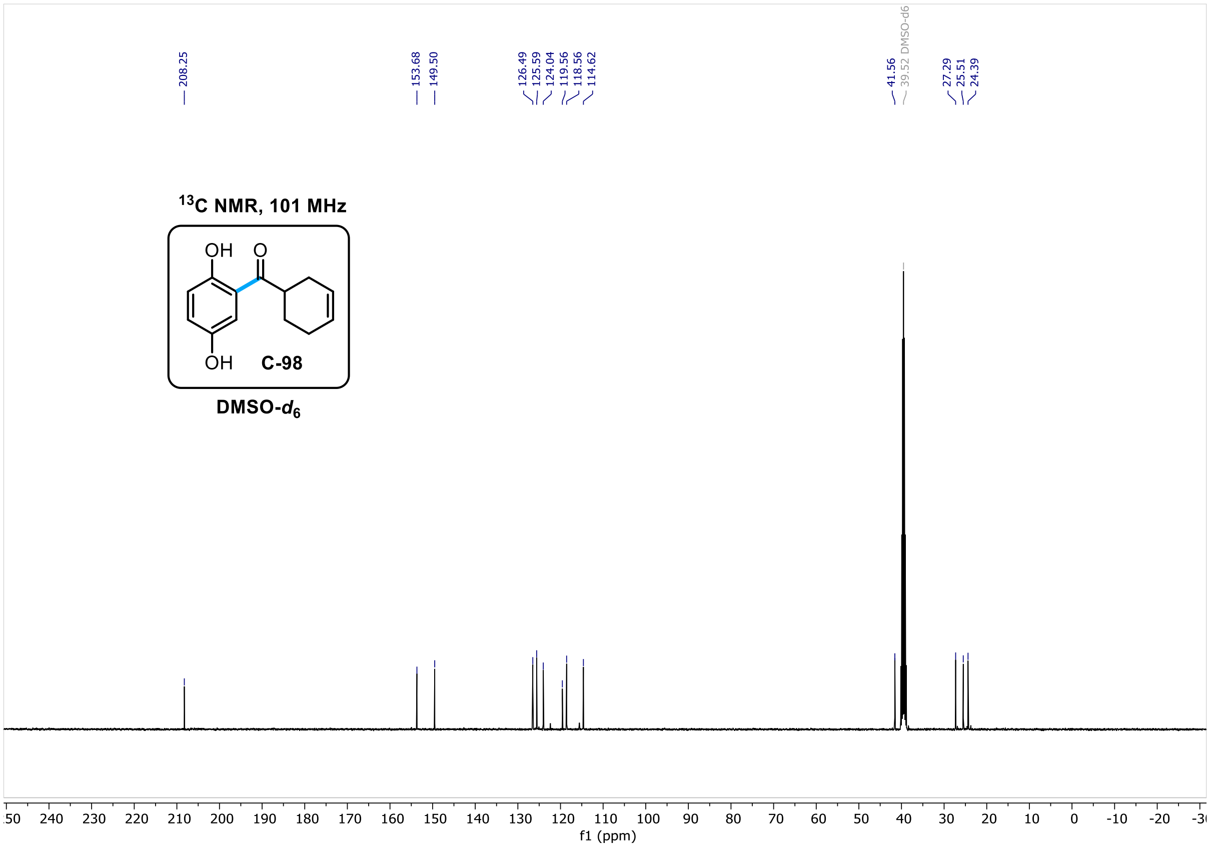

13C NMR, 101 MHz
C-98
DMSO-d6
208.25
153.68
149.50
126.49
125.59
124.04
119.56
118.56
114.62
41.56
39.52 DMSO-d6
27.29
25.51
24.39
f1 (ppm)

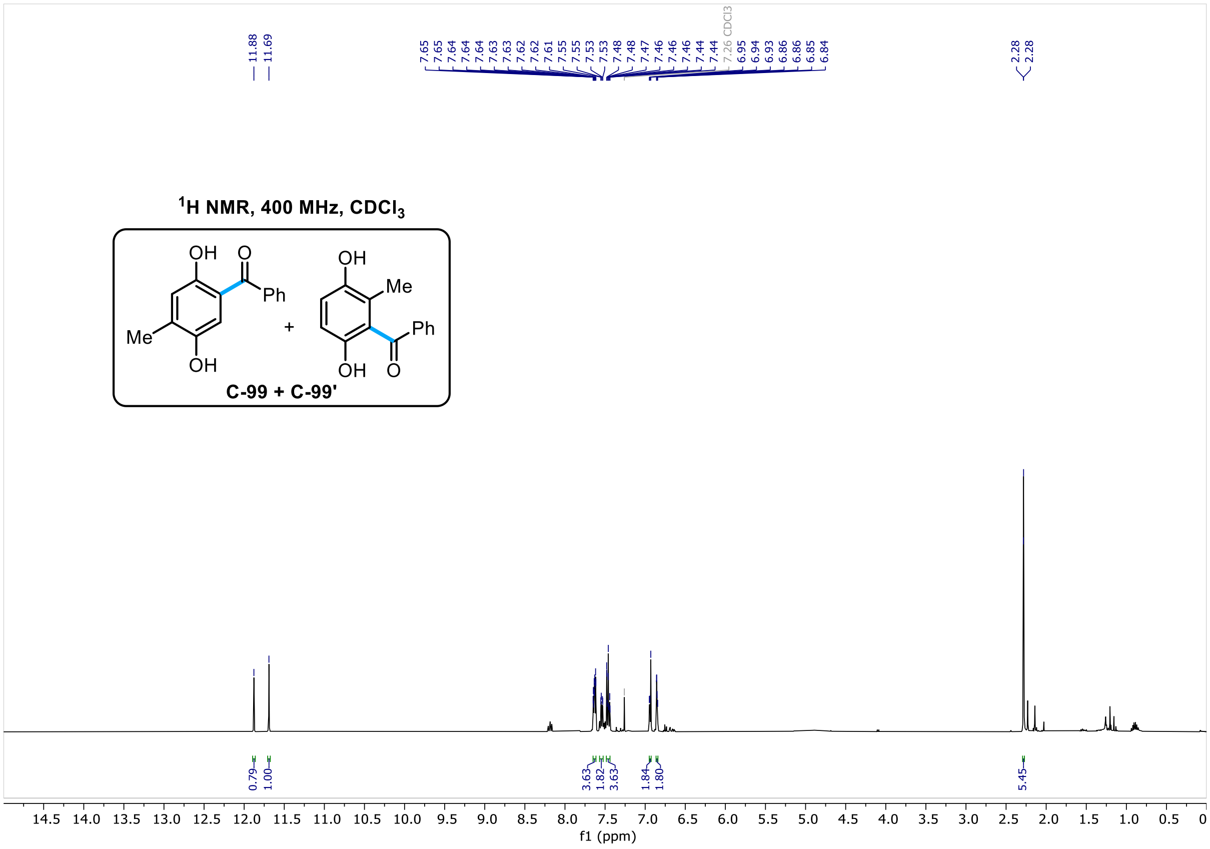

11.88
11.69
7.65 7.65 7.64 7.64 7.64 7.63 7.63 7.62 7.62 7.61 7.55 7.55 7.53 7.53 7.48 7.48 7.47 7.46 7.46 7.46 7.44 7.44
7.26 CDCl3
6.95 6.94 6.93 6.86 6.86 6.85 6.84
2.28
2.28
^{1}H NMR, 400 MHz, $CDCl_3$
OH
O
Ph
Me
OH
+
OH
Me
Ph
OH
O
C-99 + C-99'
0.79
1.00
3.63
1.82
3.63
1.84
1.80
5.45
f1 (ppm)

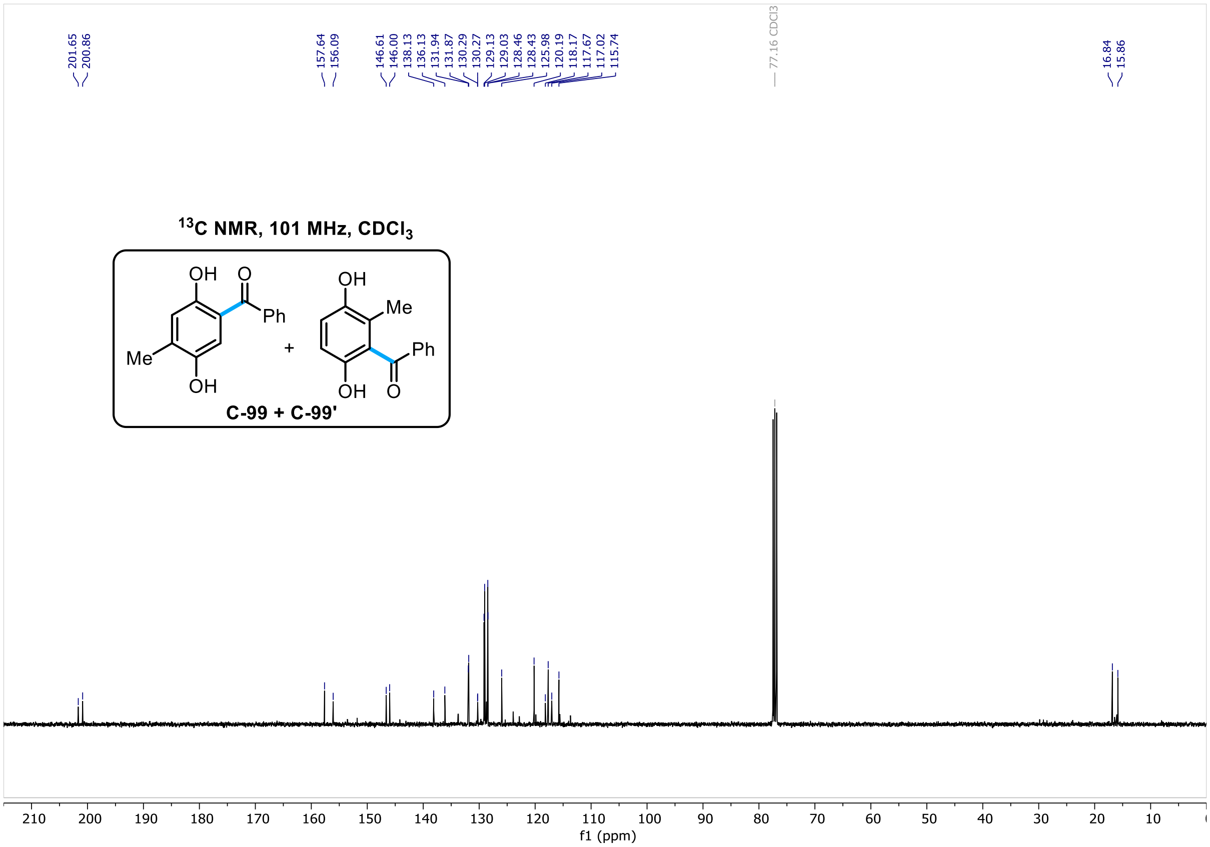

201.65
200.86
157.64
156.09
146.61 146.00 138.13 136.13 131.94 131.87 130.29 130.27 129.13 129.03 128.46 128.43 125.98 120.19 118.17 117.67 117.02 115.74
77.16 CDCl3
16.84
15.86
^{13}C NMR, 101 MHz, $CDCl_3$
C-99 + C-99'
f1 (ppm)

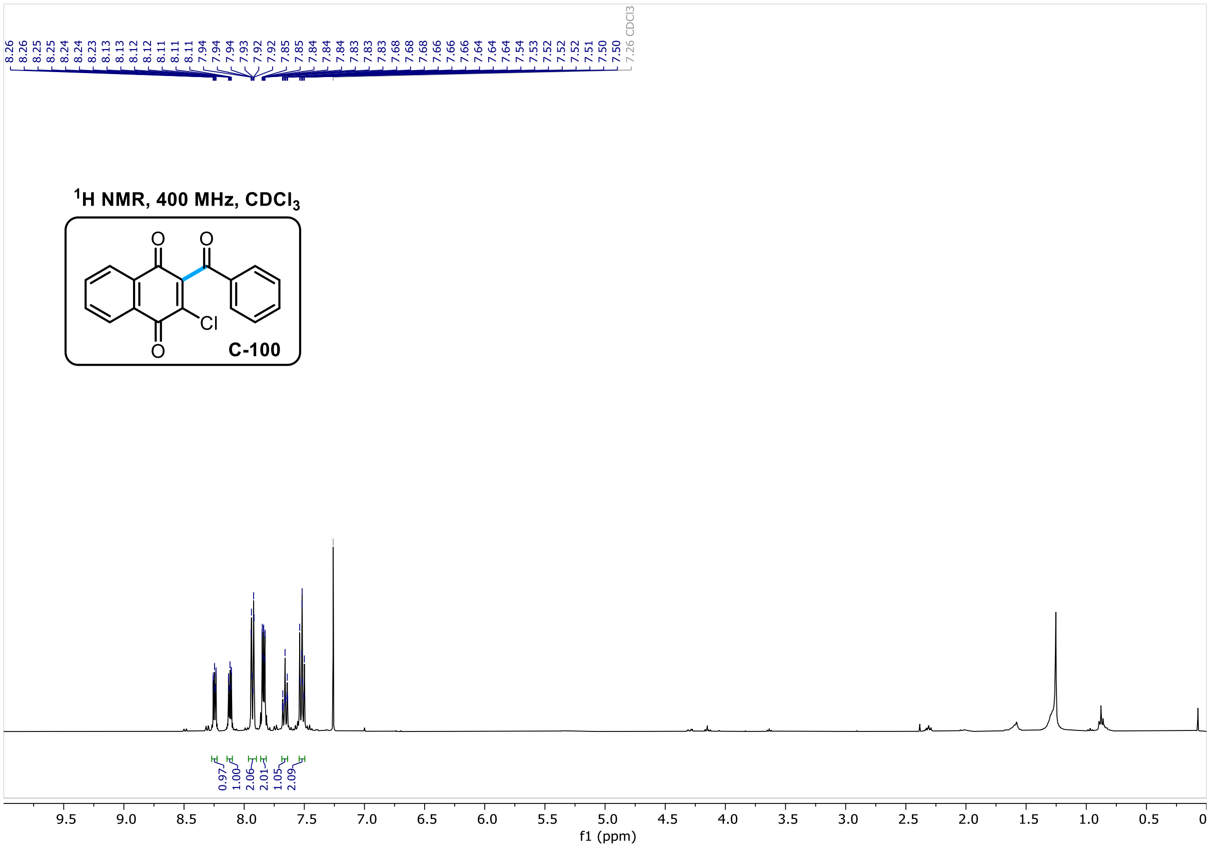
8.26
8.26
8.25
8.25
8.24
8.24
8.23
8.13
8.13
8.12
8.12
8.11
8.11
8.11
7.94
7.94
7.94
7.93
7.92
7.92
7.85
7.85
7.84
7.84
7.84
7.83
7.83
7.68
7.68
7.68
7.66
7.66
7.64
7.64
7.64
7.54
7.53
7.52
7.52
7.51
7.50
7.26 CDCl3
1H NMR, 400 MHz, CDCl3
O
O
Cl
O
C-100
0.97
1.00
2.06
2.01
1.05
2.09
9.5 9.0 8.5 8.0 7.5 7.0 6.5 6.0 5.5 5.0 4.5 4.0 3.5 3.0 2.5 2.0 1.5 1.0 0.5 0
f1 (ppm)

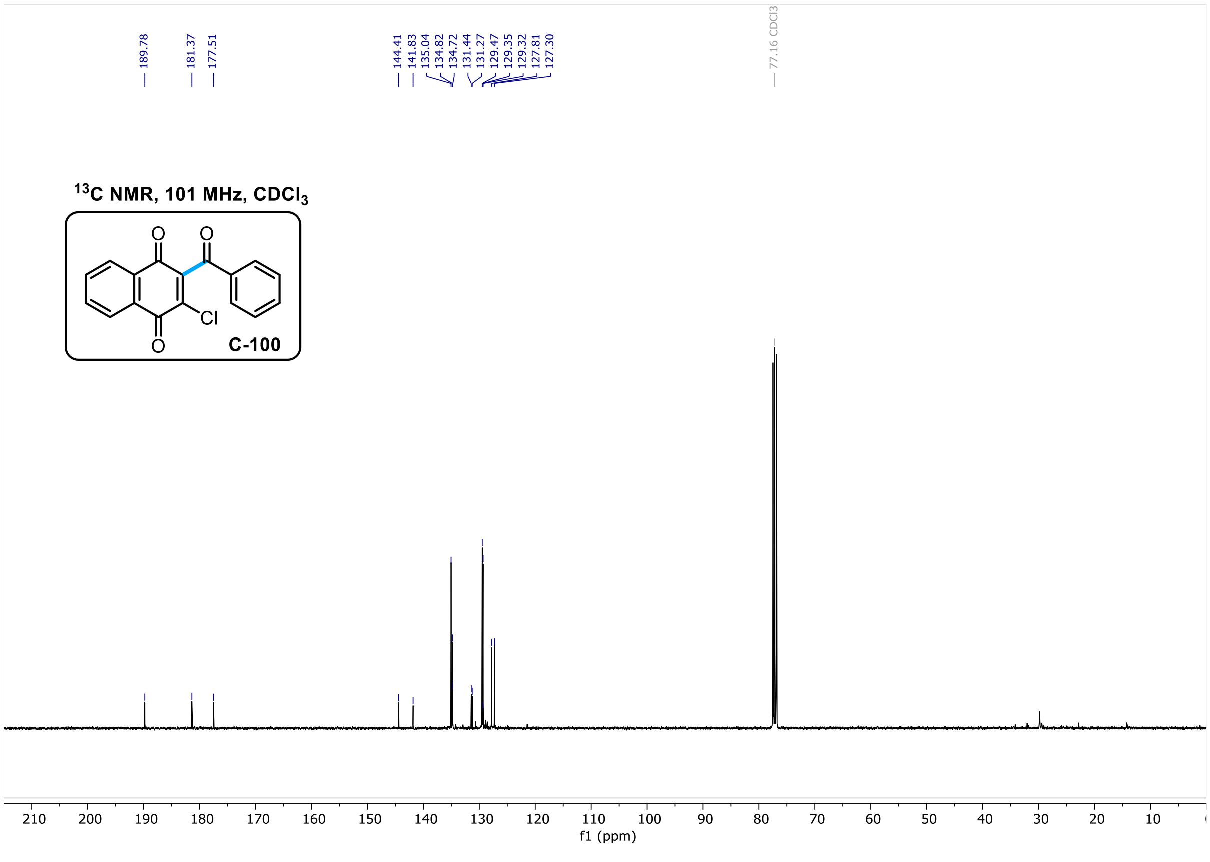
189.78
181.37
177.51
144.41
141.83
135.04
134.82
134.72
131.44
131.27
129.47
129.35
129.32
127.81
127.30
77.16 CDCl3
13C NMR, 101 MHz, CDCl3
O
O
Cl
O
C-100
210 200 190 180 170 160 150 140 130 120 110 100 90 80 70 60 50 40 30 20 10
f1 (ppm)

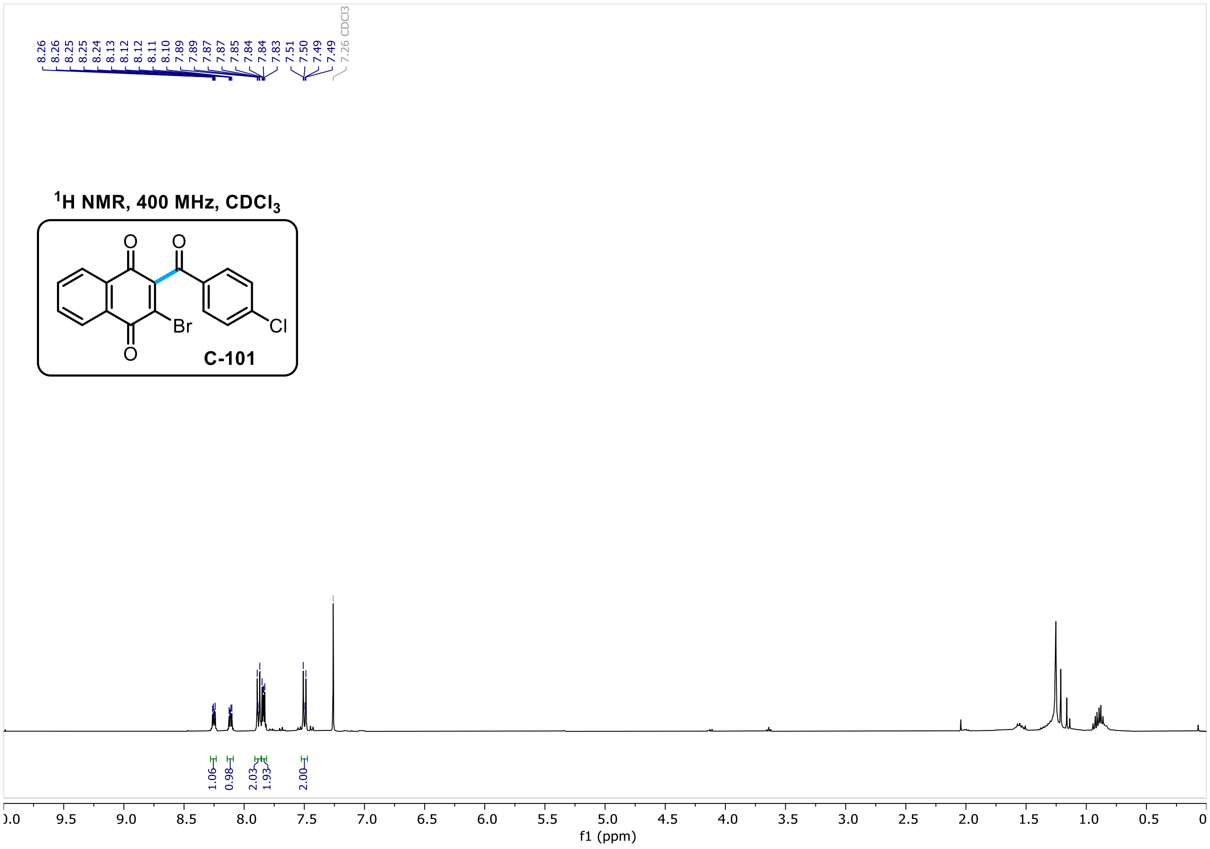
8.26
8.26
8.25
8.25
8.24
8.13
8.12
8.12
8.11
8.10
7.89
7.89
7.87
7.87
7.85
7.84
7.84
7.83
7.51
7.50
7.49
7.49
7.26 CDCl3
1H NMR, 400 MHz, CDCl3
O
O
Br
Cl
O
C-101
1.06
0.98
2.03
1.93
2.00
f1 (ppm)

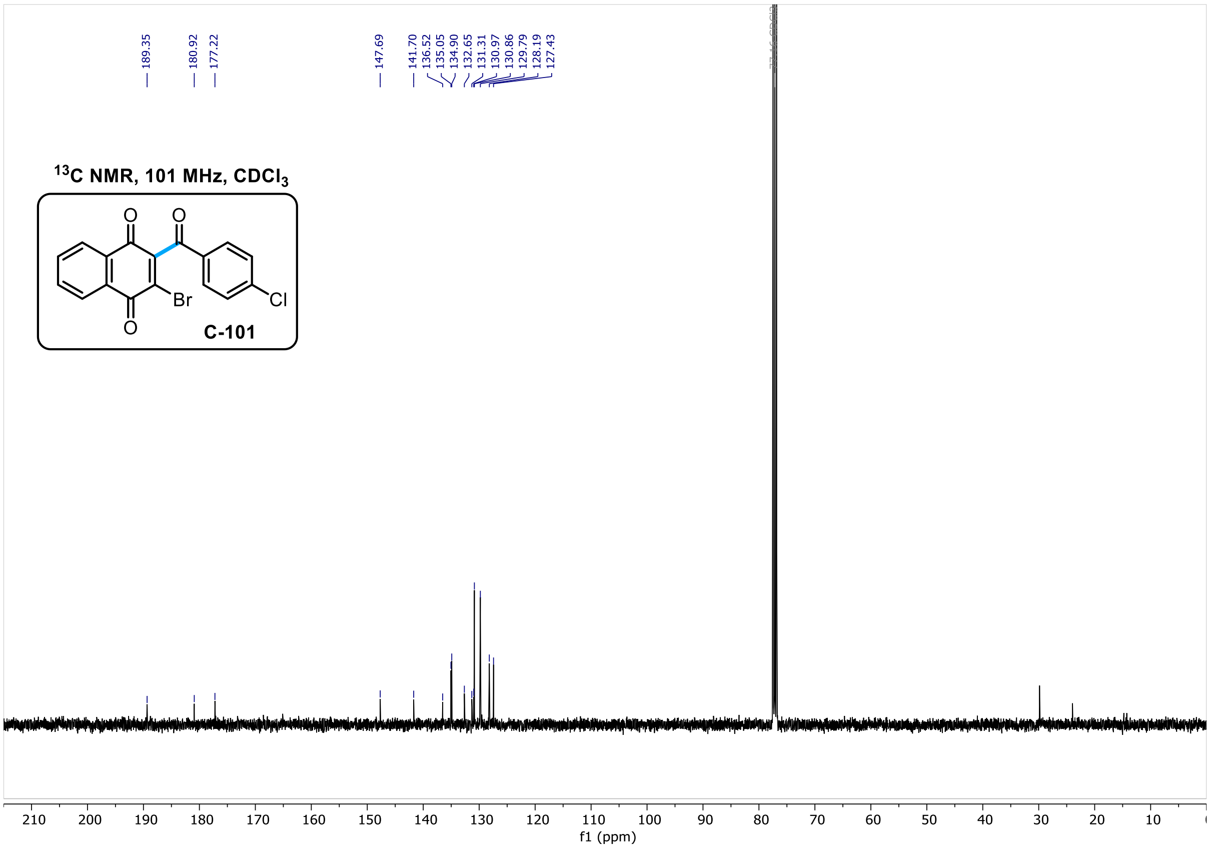
189.35
180.92
177.22
147.69
141.70
136.52
135.05
134.90
132.65
131.31
130.97
130.86
129.79
128.19
127.43
13C NMR, 101 MHz, CDCl3
O
O
Br
Cl
O
C-101
f1 (ppm)

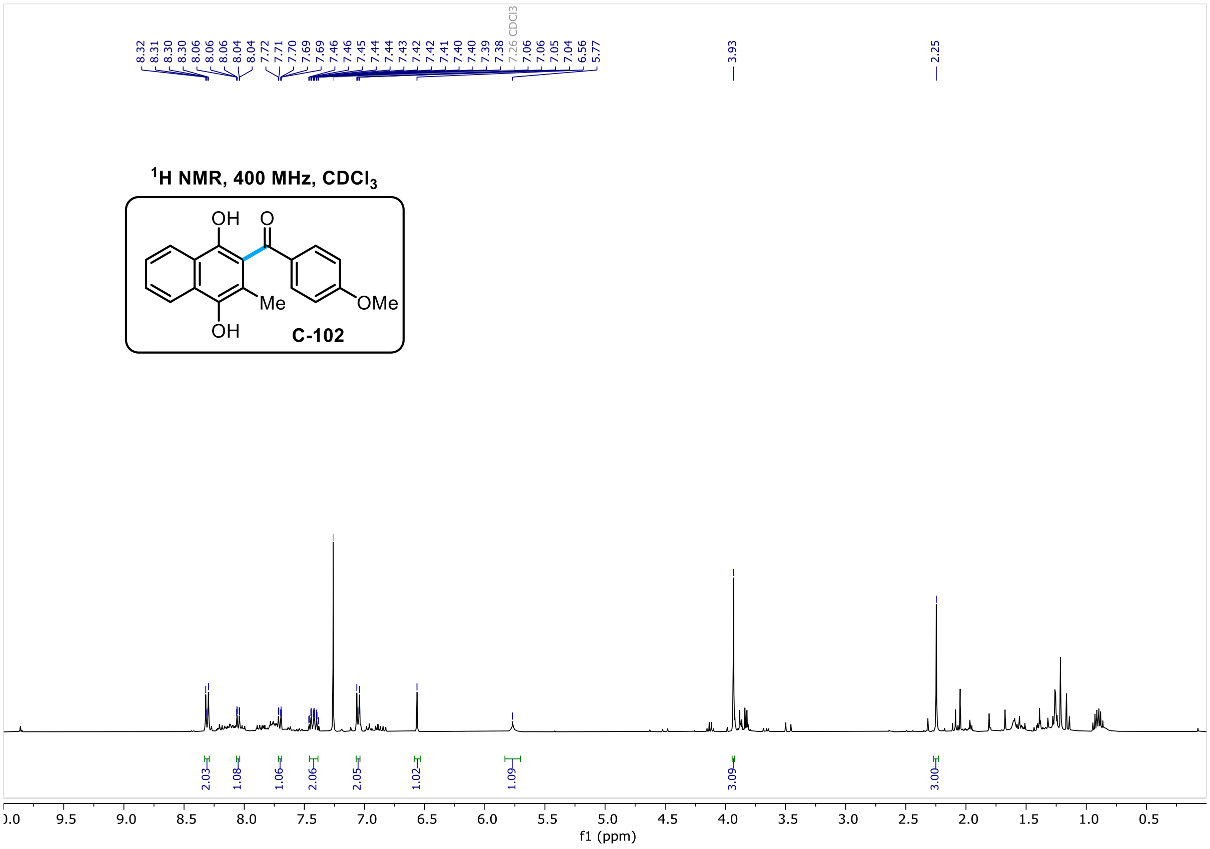
8.32
8.31
8.30
8.30
8.06
8.06
8.06
8.04
8.04
7.72
7.71
7.70
7.69
7.69
7.46
7.46
7.45
7.44
7.44
7.43
7.42
7.42
7.41
7.40
7.40
7.39
7.38
7.26 CDCl3
7.06
7.06
7.05
7.04
6.56
5.77
3.93
2.25
^{1}H NMR, 400 MHz, $CDCl_3$
OH
O
Me
OMe
OH
C-102
2.03
1.08
1.06
2.06
2.05
1.02
1.09
3.09
3.00
f1 (ppm)

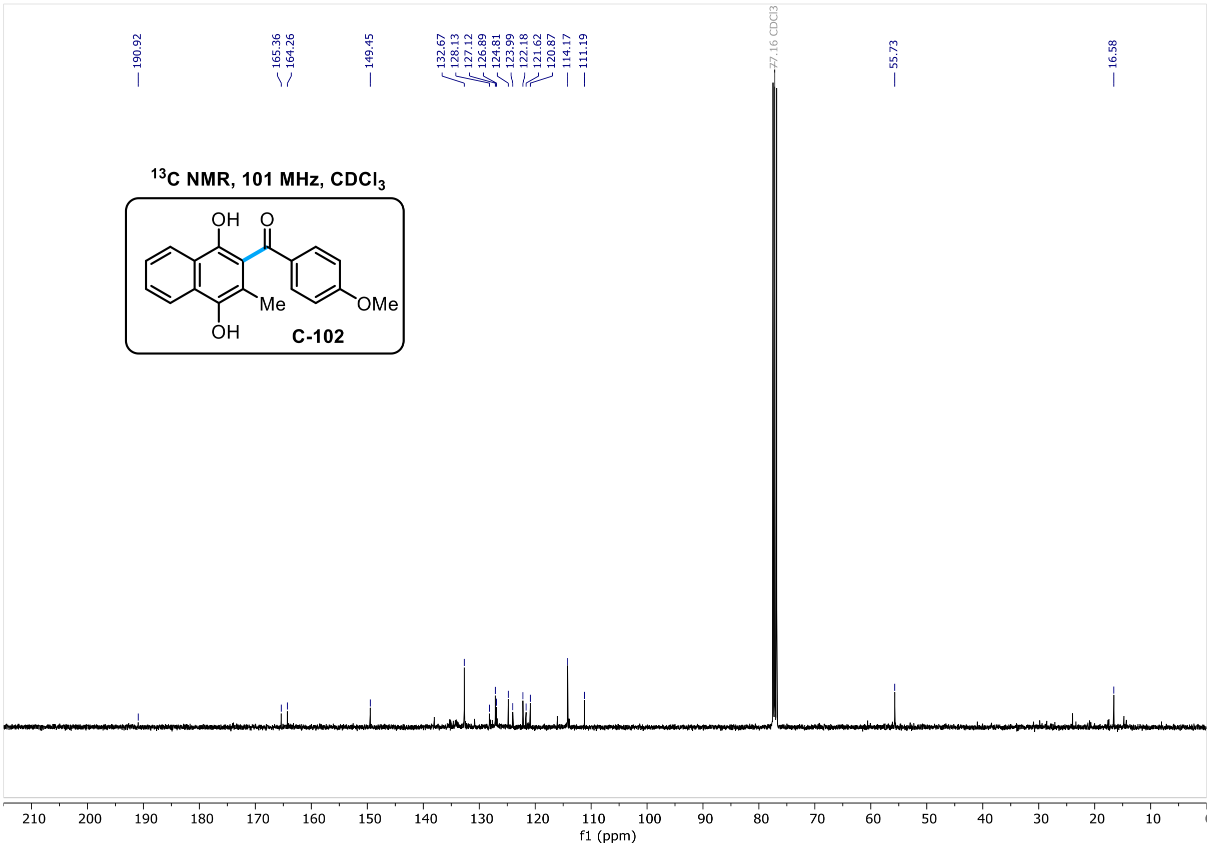
190.92
165.36
164.26
149.45
132.67
128.13
127.12
126.89
124.81
123.99
122.18
121.62
120.87
114.17
111.19
77.16 CDCl3
55.73
16.58
^{13}C NMR, 101 MHz, $CDCl_3$
OH
O
Me
OMe
OH
C-102
f1 (ppm)

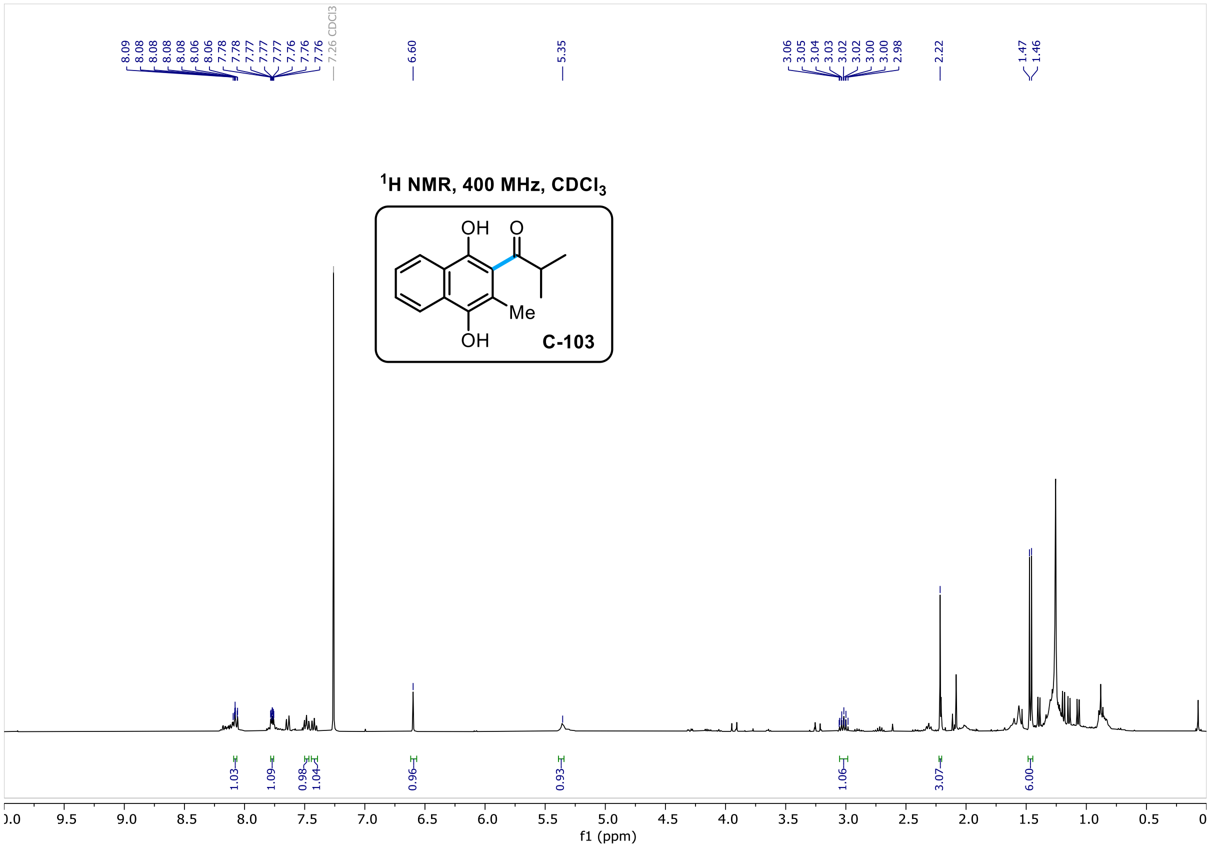

8.09
8.08
8.08
8.08
8.08
8.06
8.06
7.78
7.78
7.77
7.77
7.77
7.76
7.76
7.26 CDCl3
6.60
5.35
3.06
3.05
3.04
3.03
3.02
3.02
3.00
3.00
2.98
2.22
1.47
1.46
^{1}H NMR, 400 MHz, CDCl$_3$
OH
O
Me
OH
C-103
1.03
1.09
0.98
1.04
0.96
0.93
1.06
3.07
6.00
f1 (ppm)

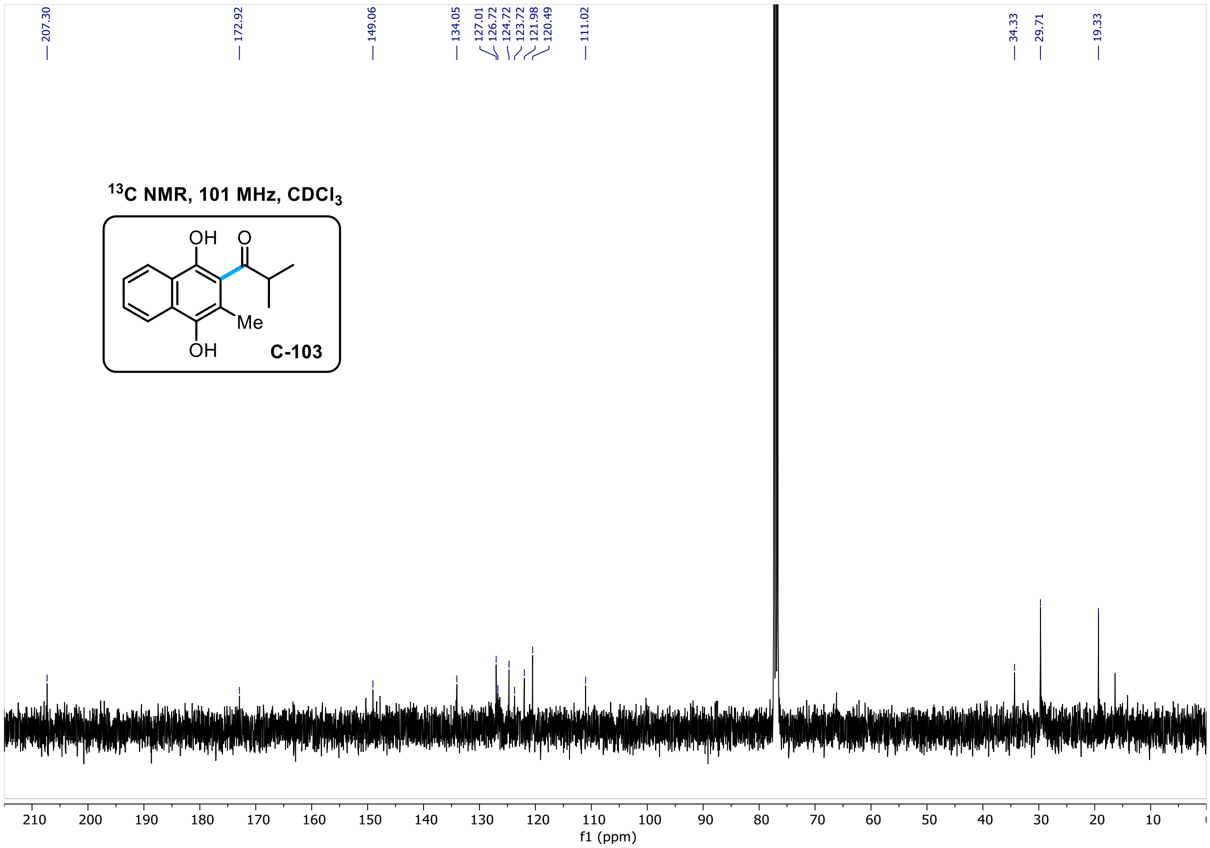

207.30
172.92
149.06
134.05
127.01
126.72
124.72
123.72
121.98
120.49
111.02
34.33
29.71
19.33
^{13}C NMR, 101 MHz, CDCl$_3$
OH
O
Me
OH
C-103
f1 (ppm)

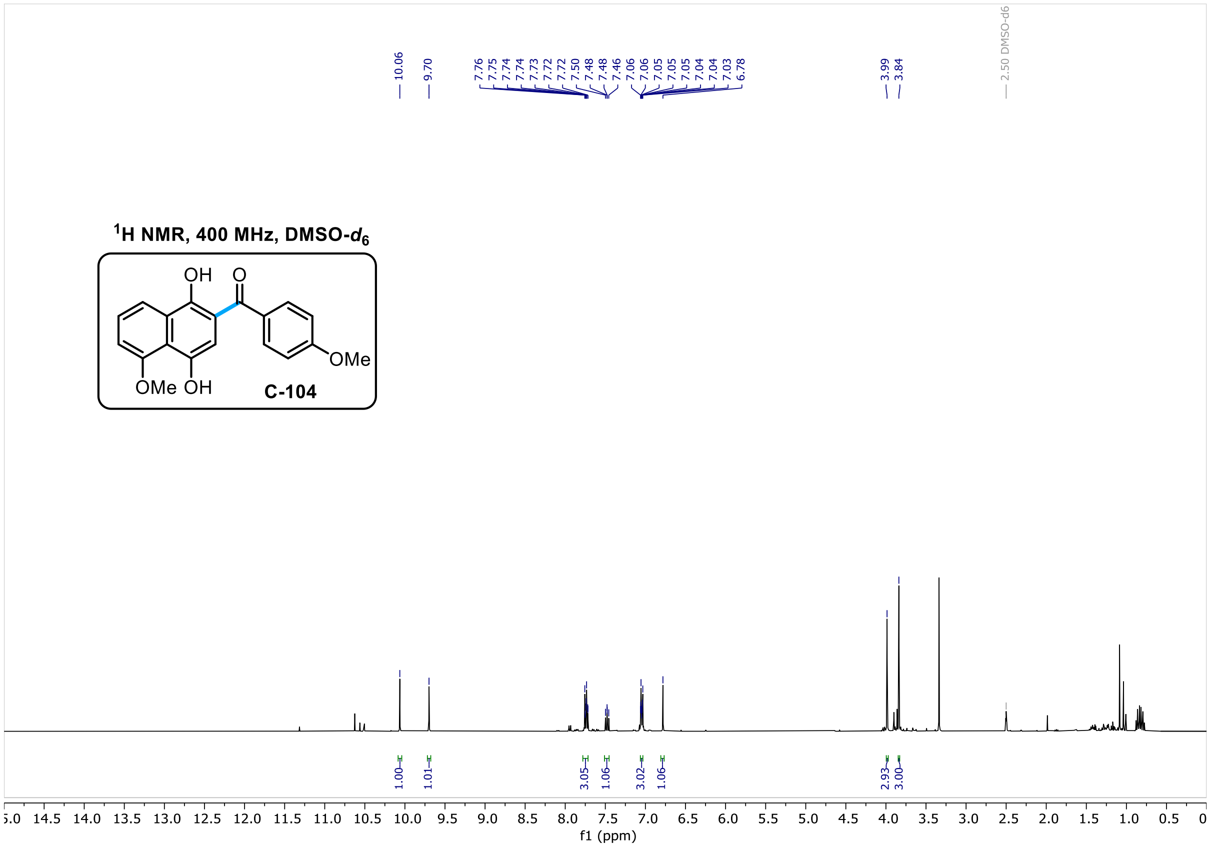
10.06
9.70
7.76 7.75 7.74 7.74 7.73 7.72 7.72 7.50 7.48 7.48 7.46 7.06 7.06 7.05 7.05 7.05 7.04 7.03 6.78
3.99
3.84
2.50 DMSO-d6
1H NMR, 400 MHz, DMSO-d6
OH
O
OMe
OMe OH
C-104
1.00
1.01
3.05
1.06
3.02
1.06
2.93
3.00
f1 (ppm)

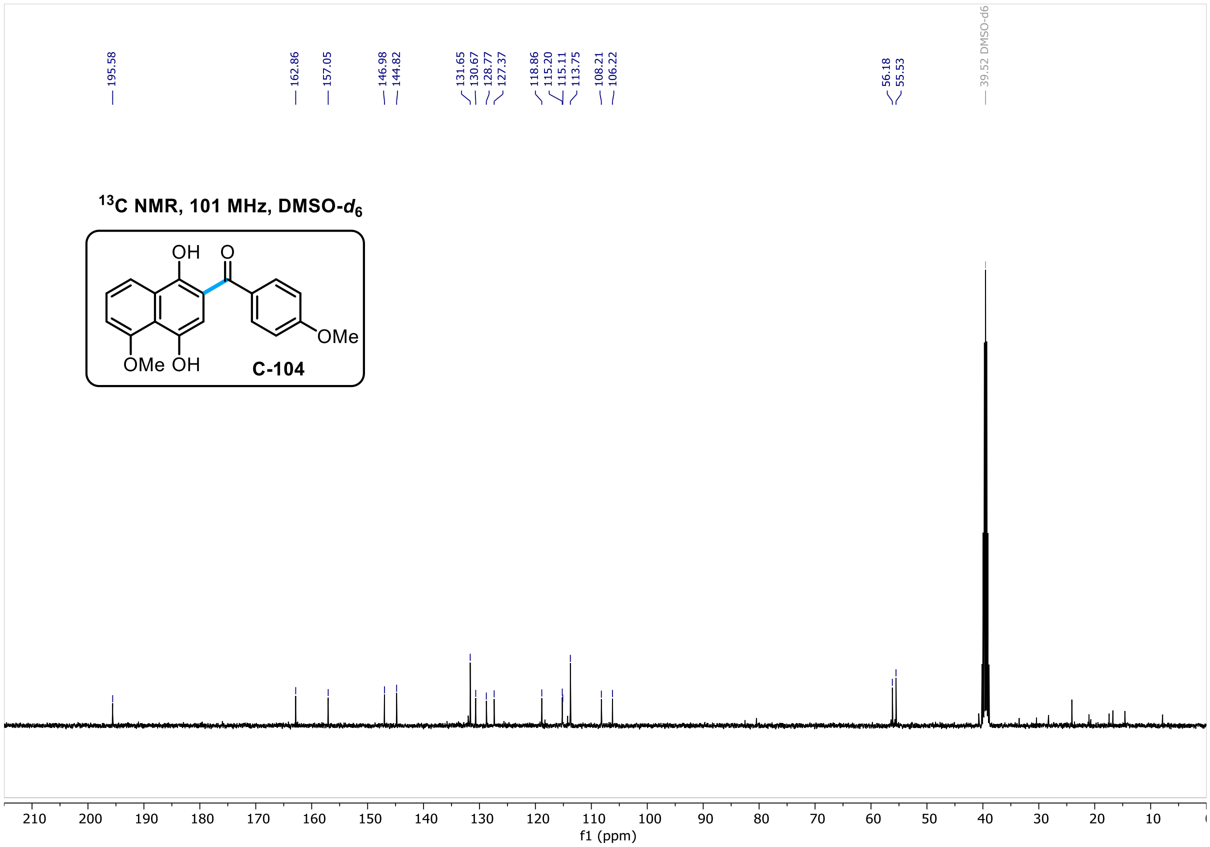
195.58
162.86
157.05
146.98
144.82
131.65 130.67 128.77 127.37
118.86 115.20 115.11 113.75
108.21
106.22
56.18
55.53
39.52 DMSO-d6
13C NMR, 101 MHz, DMSO-d6
OH
O
OMe
OMe OH
C-104
f1 (ppm)

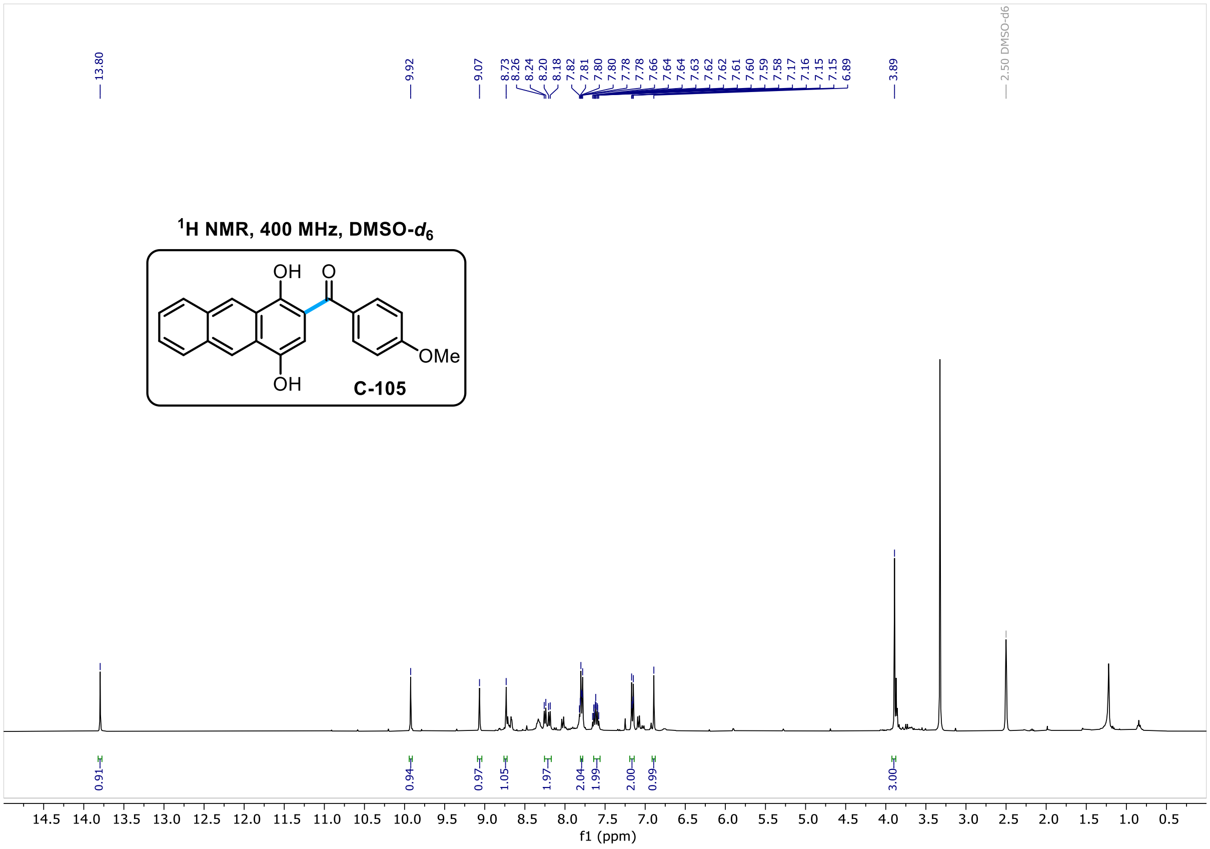
13.80
9.92
9.07
8.73
8.26
8.24
8.20
8.18
7.82
7.81
7.80
7.80
7.78
7.78
7.66
7.64
7.64
7.63
7.62
7.62
7.61
7.60
7.59
7.58
7.17
7.16
7.15
7.15
6.89
3.89
2.50 DMSO-d6
1H NMR, 400 MHz, DMSO-d6
OH
O
OMe
OH
C-105
0.91
0.94
0.97
1.05
1.97
2.04
1.99
2.00
0.99
3.00
14.5 14.0 13.5 13.0 12.5 12.0 11.5 11.0 10.5 10.0 9.5 9.0 8.5 8.0 7.5 7.0 6.5 6.0 5.5 5.0 4.5 4.0 3.5 3.0 2.5 2.0 1.5 1.0 0.5
f1 (ppm)

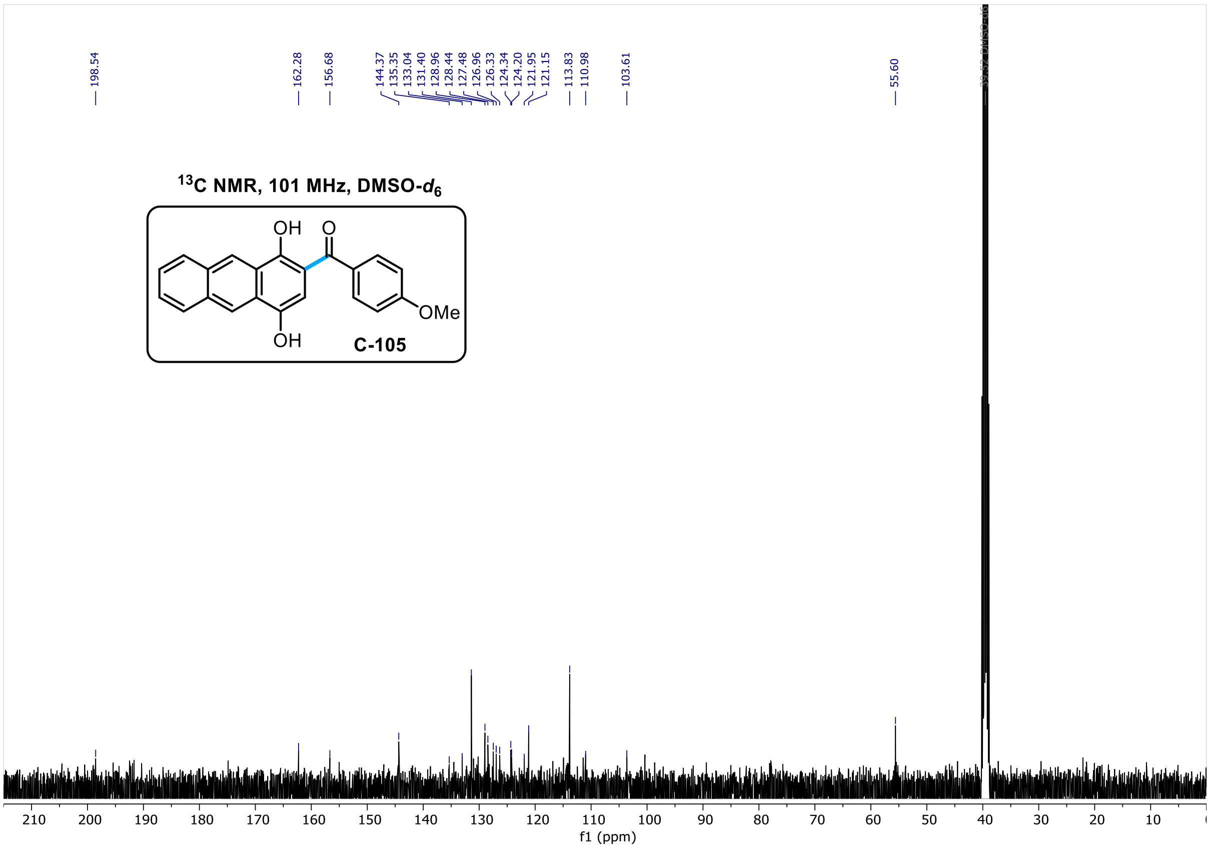
198.54
162.28
156.68
144.37
135.35
133.04
131.40
128.96
128.44
127.48
126.96
126.33
124.34
124.20
121.95
121.15
113.83
110.98
103.61
55.60
13C NMR, 101 MHz, DMSO-d6
OH
O
OMe
OH
C-105
210 200 190 180 170 160 150 140 130 120 110 100 90 80 70 60 50 40 30 20 10
f1 (ppm)

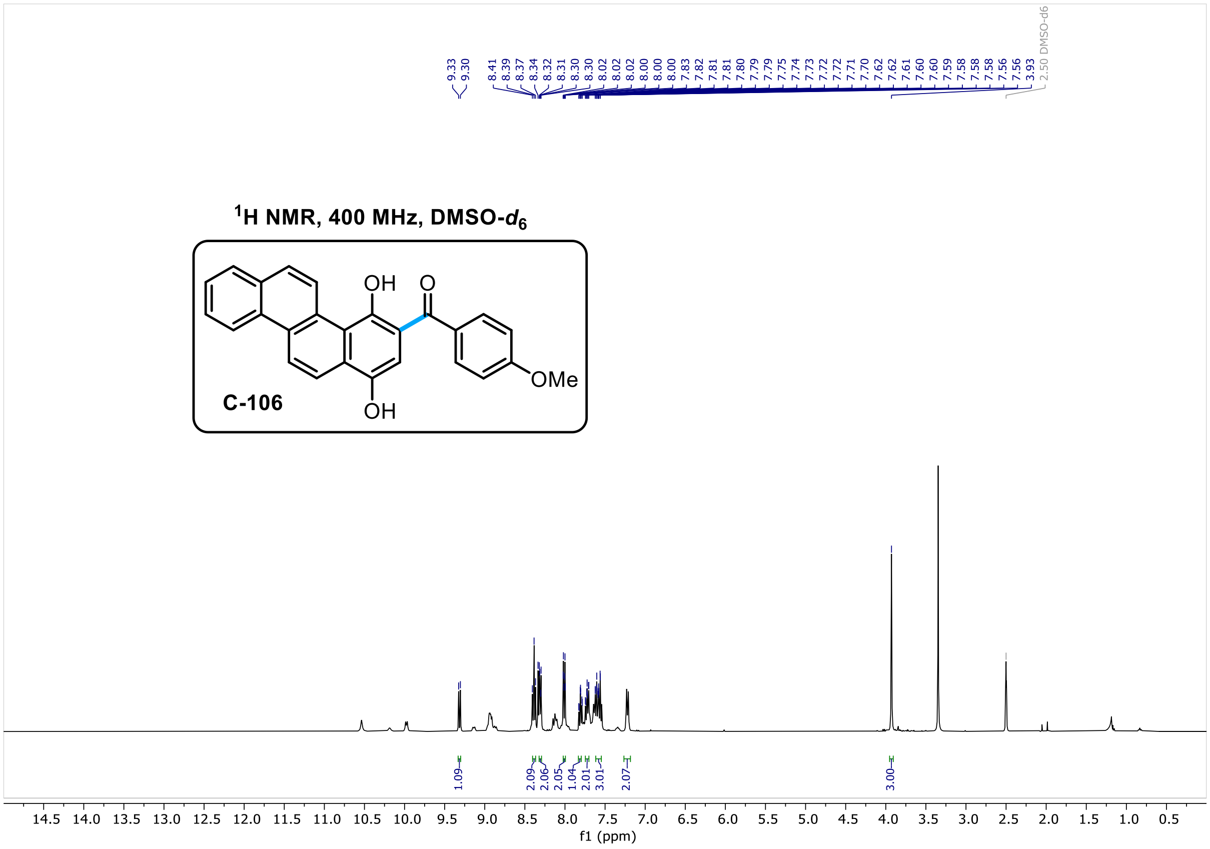

9.33
9.30
8.41
8.39
8.37
8.34
8.32
8.31
8.30
8.30
8.02
8.02
8.02
8.00
8.00
7.83
7.82
7.81
7.81
7.80
7.79
7.79
7.75
7.74
7.73
7.72
7.72
7.71
7.70
7.62
7.62
7.61
7.60
7.59
7.58
7.58
7.56
7.56
3.93
2.50 DMSO-d6
^{1}H NMR, 400 MHz, DMSO-d_6
OH
O
OMe
C-106
OH
1.09
2.09
2.06
2.05
1.04
2.01
3.01
2.07
3.00
14.5 14.0 13.5 13.0 12.5 12.0 11.5 11.0 10.5 10.0 9.5 9.0 8.5 8.0 7.5 7.0 6.5 6.0 5.5 5.0 4.5 4.0 3.5 3.0 2.5 2.0 1.5 1.0 0.5
f1 (ppm)

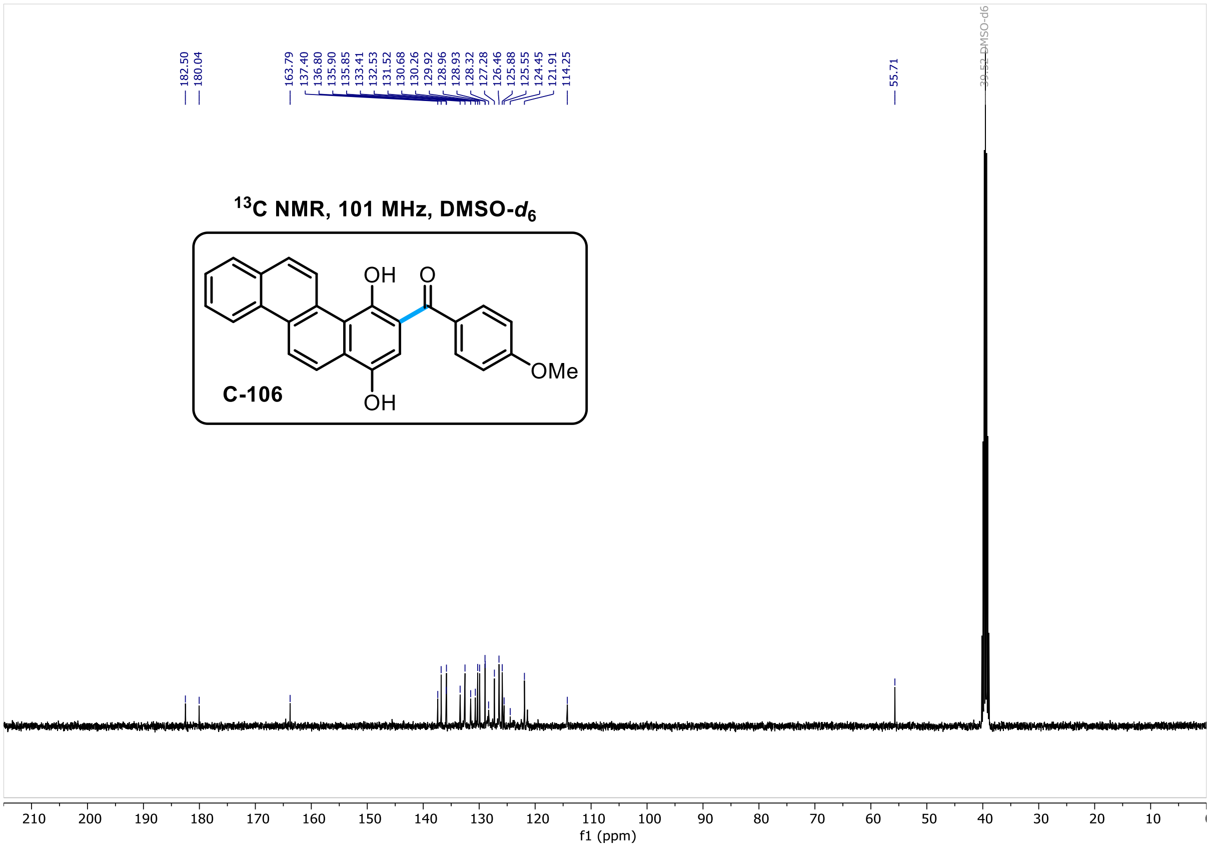

182.50
180.04
163.79
137.40
136.80
135.90
135.85
133.41
132.53
131.52
130.68
130.26
129.92
128.96
128.93
128.32
127.28
126.46
125.88
125.55
124.45
121.91
114.25
55.71
39.52 DMSO-d6
^{13}C NMR, 101 MHz, DMSO-d_6
OH
O
OMe
C-106
OH
210 200 190 180 170 160 150 140 130 120 110 100 90 80 70 60 50 40 30 20 10
f1 (ppm)

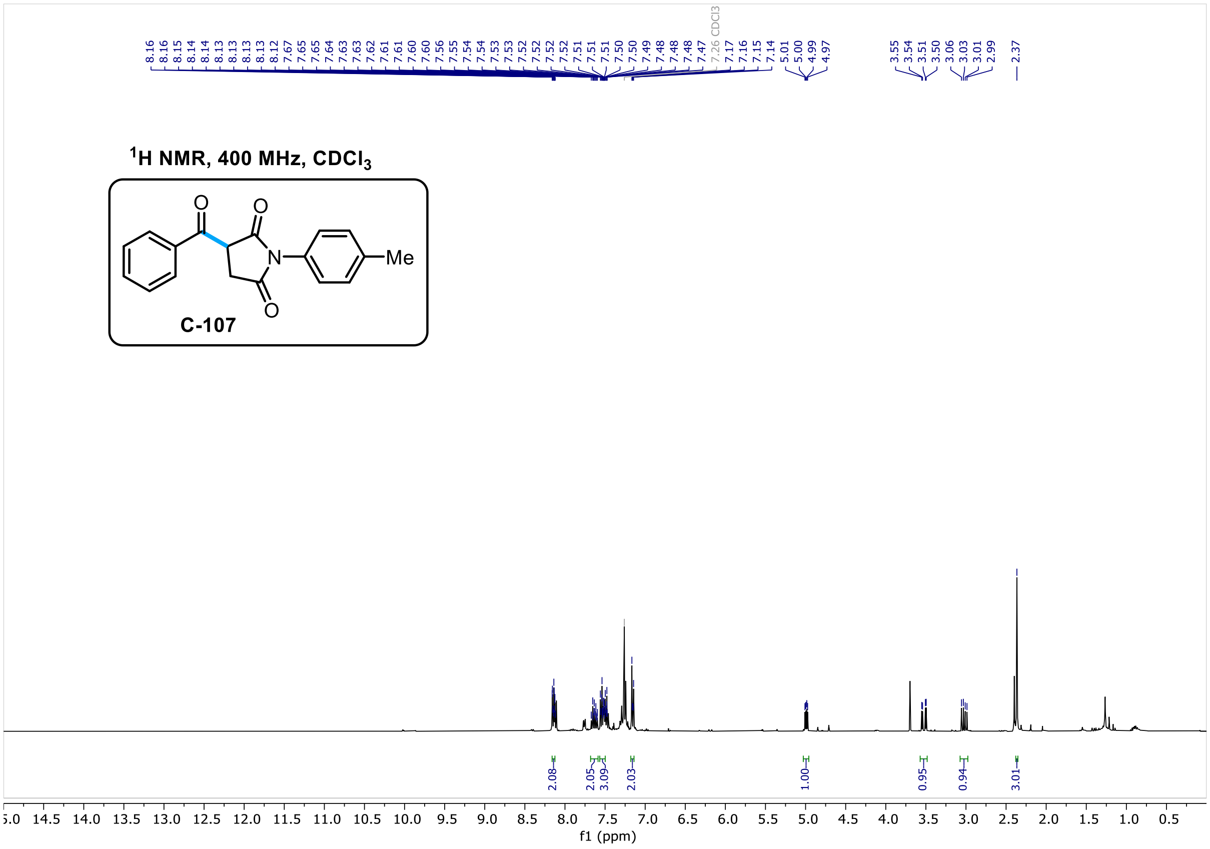

1H NMR, 400 MHz, CDCl3
Me
C-107
f1 (ppm)

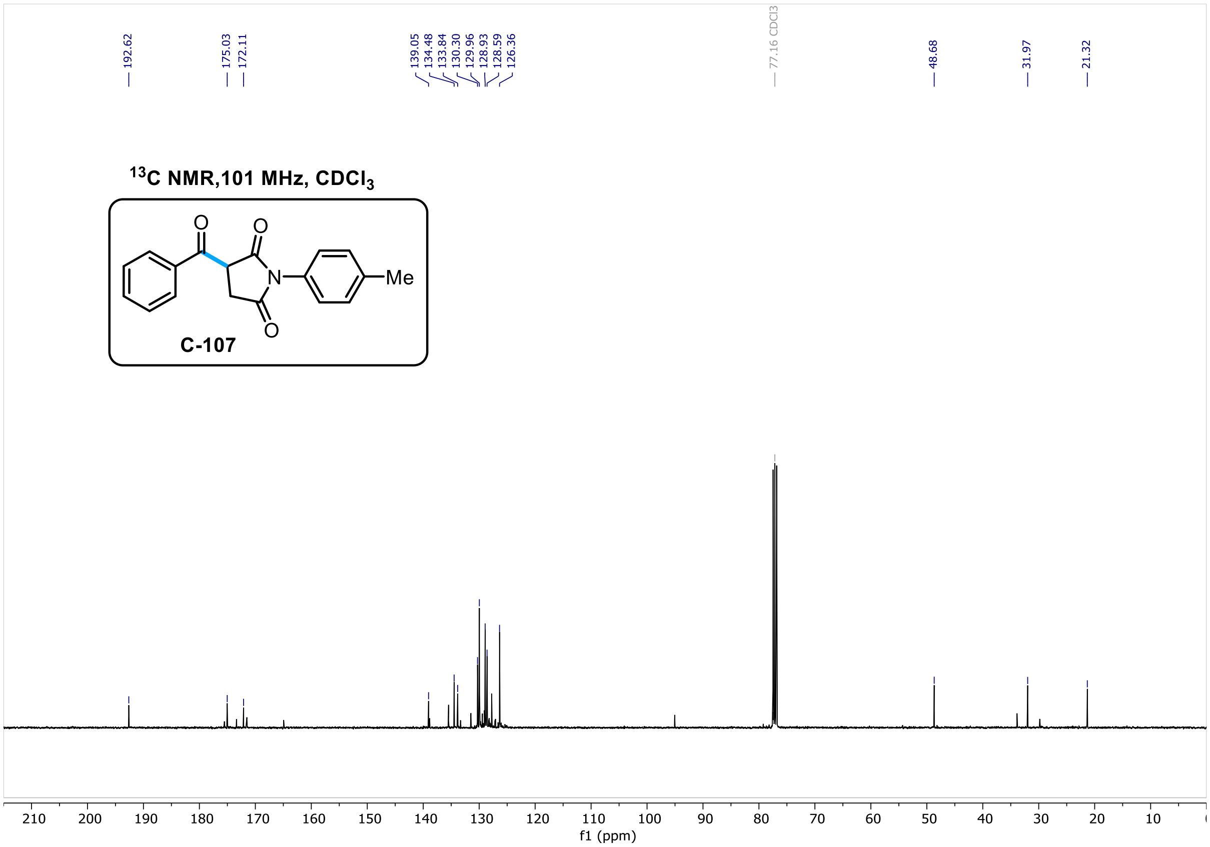

13C NMR,101 MHz, CDCl3
Me
C-107
f1 (ppm)

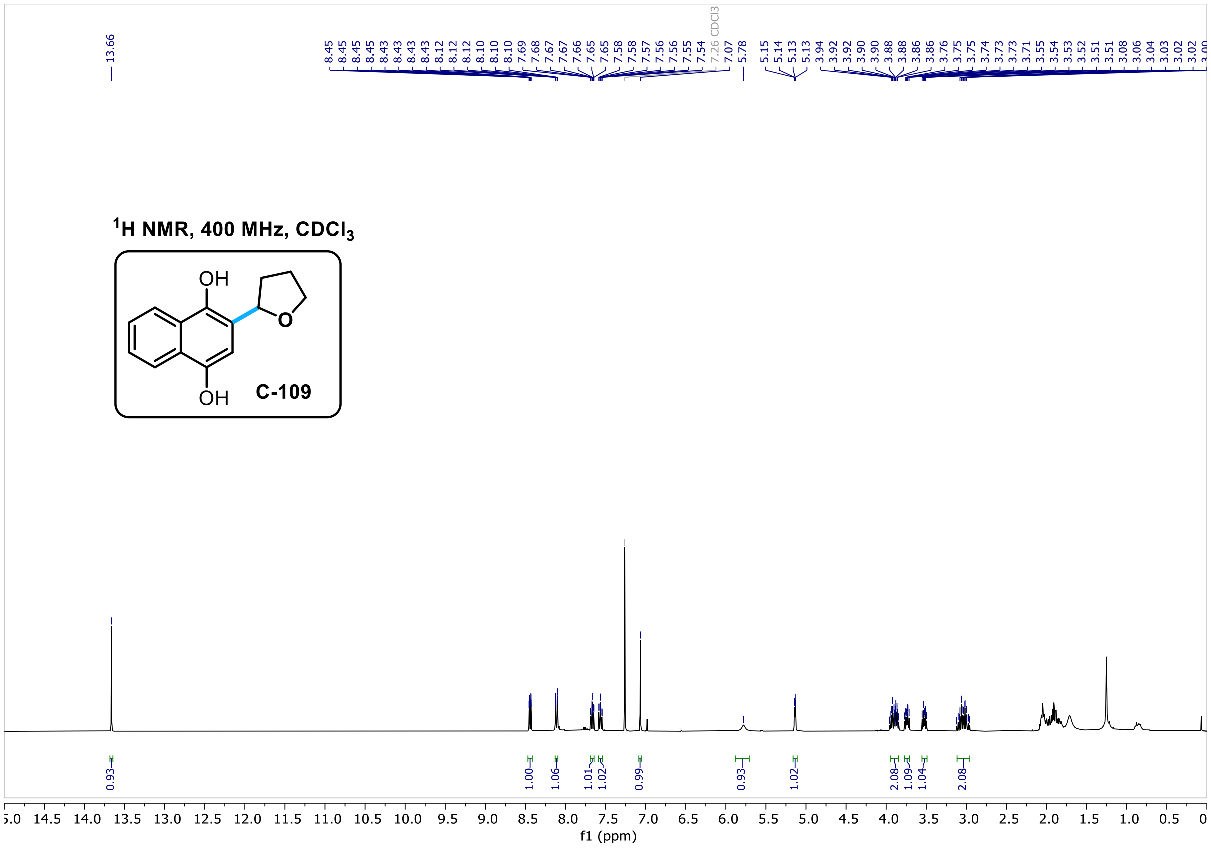

^{1}H NMR, 400 MHz, CDCl$_3$
OH
O
OH
C-109
f1 (ppm)

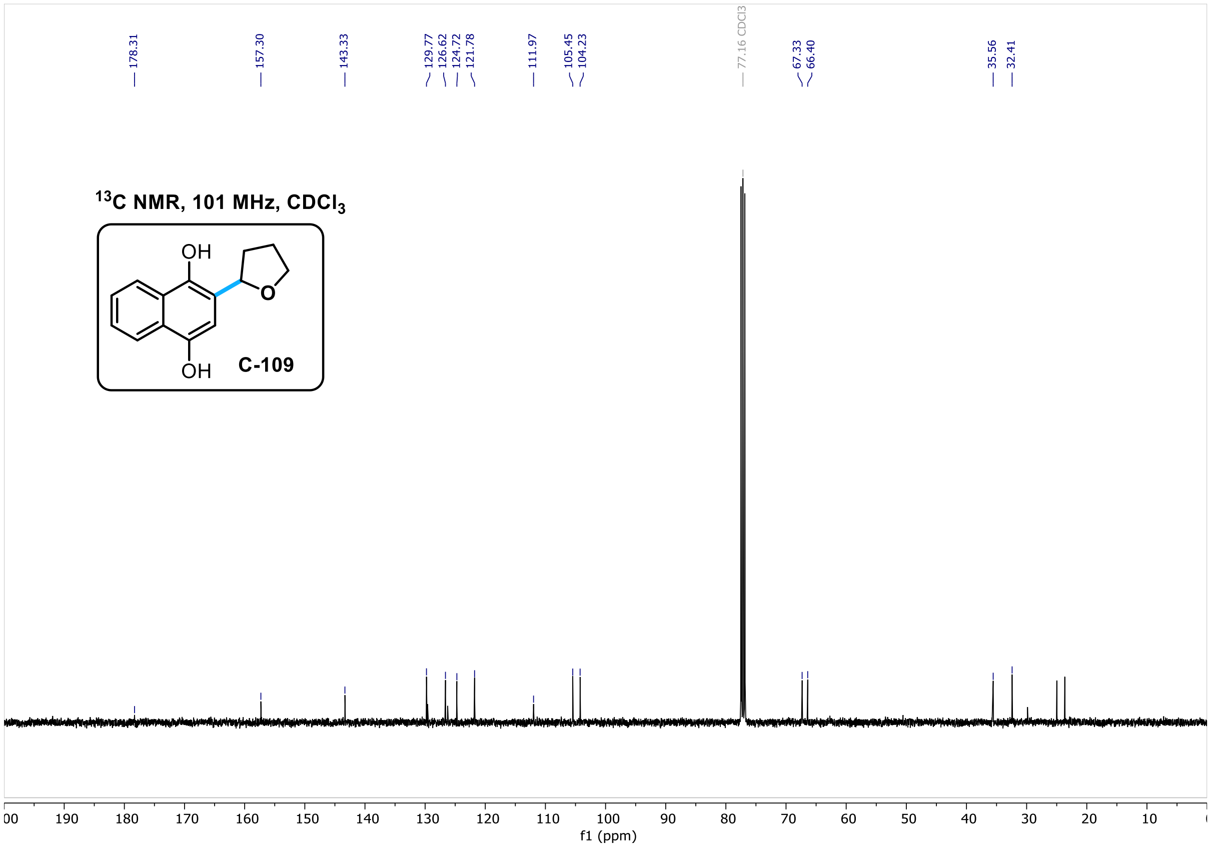

^{13}C NMR, 101 MHz, CDCl$_3$
178.31
157.30
143.33
129.77
126.62
124.72
121.78
111.97
105.45
104.23
77.16 CDCl3
67.33
66.40
35.56
32.41
OH
O
OH
C-109
f1 (ppm)

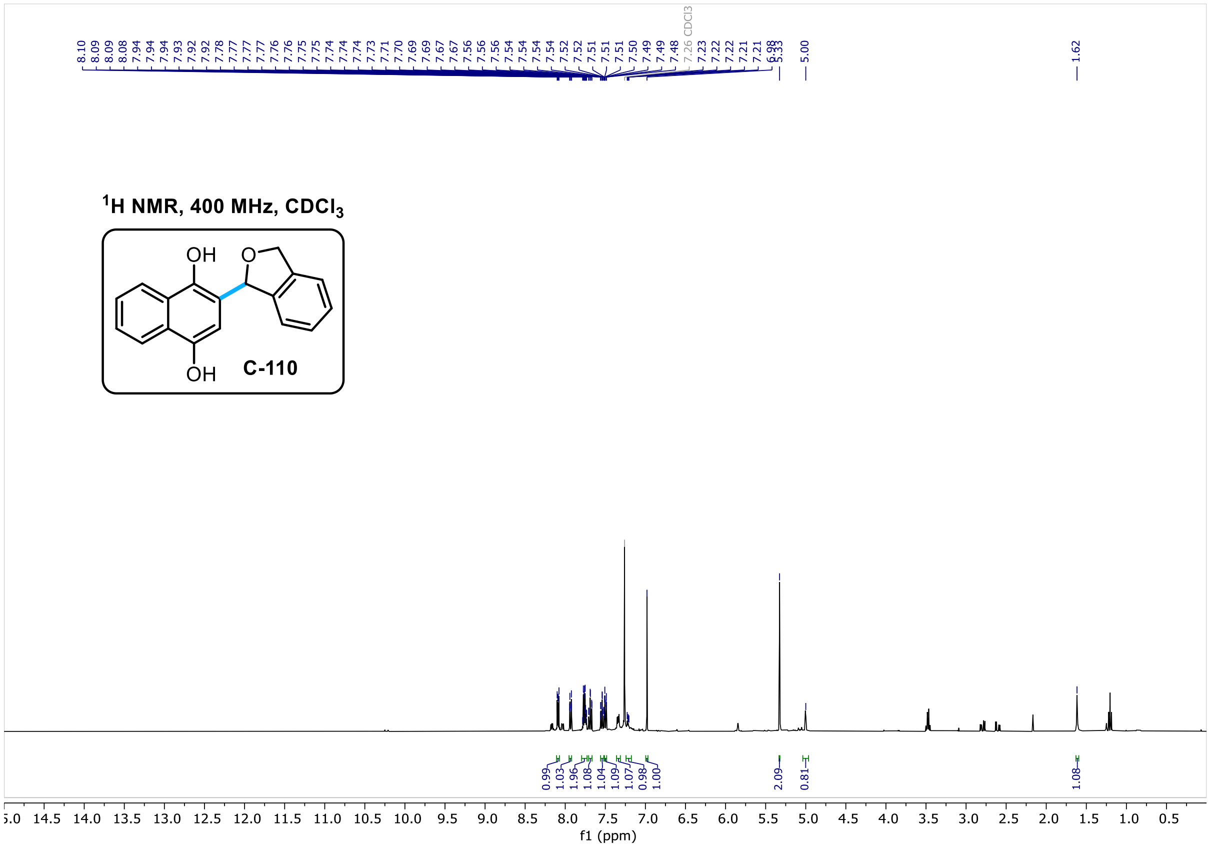
8.10 8.09 8.09 8.08 7.94 7.94 7.93 7.92 7.78 7.77 7.77 7.76 7.76 7.75 7.75 7.74 7.74 7.73 7.71 7.70 7.69 7.67 7.67 7.56 7.56 7.54 7.54 7.54 7.52 7.52 7.51 7.51 7.51 7.50 7.49 7.48 7.26 CDCl3 7.23 7.22 7.22 7.21 7.21 6.98 5.33 5.00 1.62
1H NMR, 400 MHz, CDCl3
OH
O
OH
C-110
0.99 1.03 1.96 1.08 1.04 1.09 1.07 0.98 1.00 2.09 0.81 1.08
f1 (ppm)

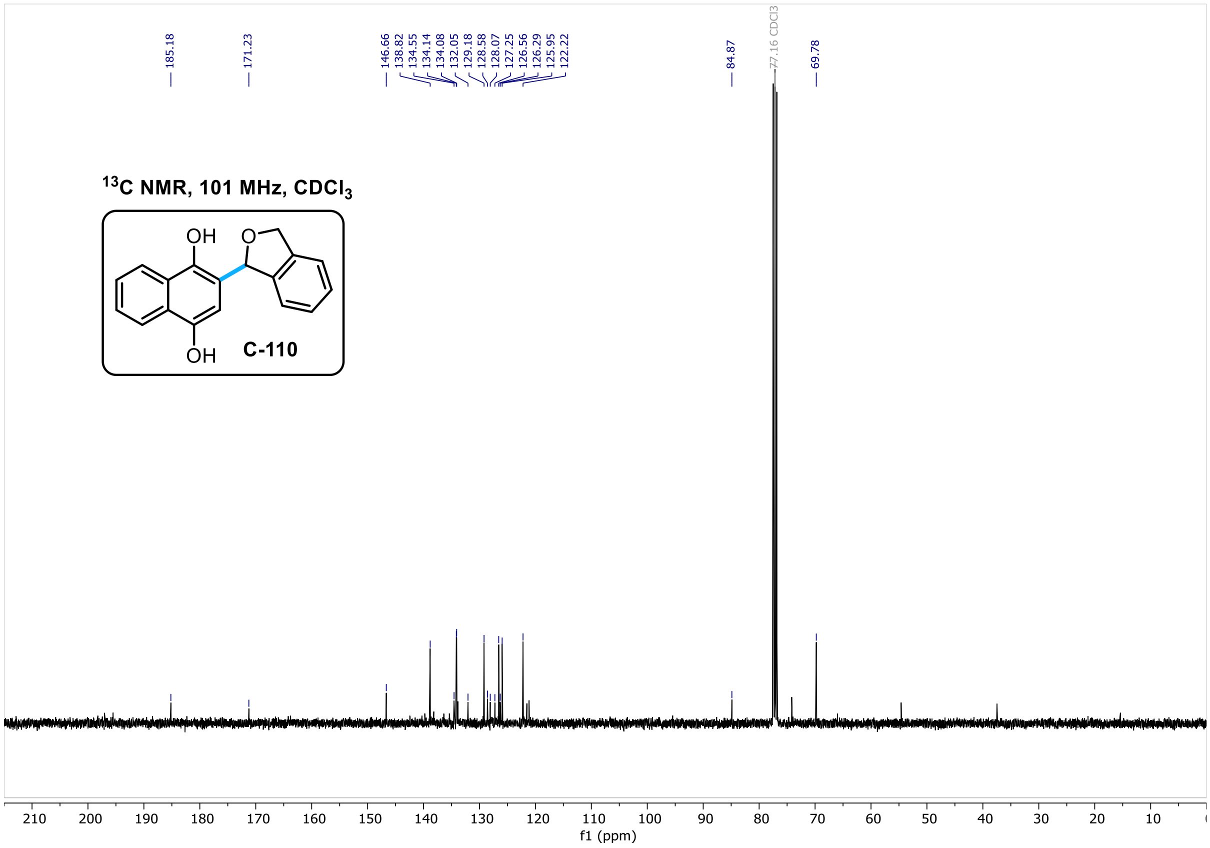
185.18 171.23 146.66 138.82 134.55 134.14 134.08 132.05 129.18 128.58 128.07 127.25 126.56 126.29 125.95 122.22 84.87 77.16 CDCl3 69.78
13C NMR, 101 MHz, CDCl3
OH
O
OH
C-110
f1 (ppm)

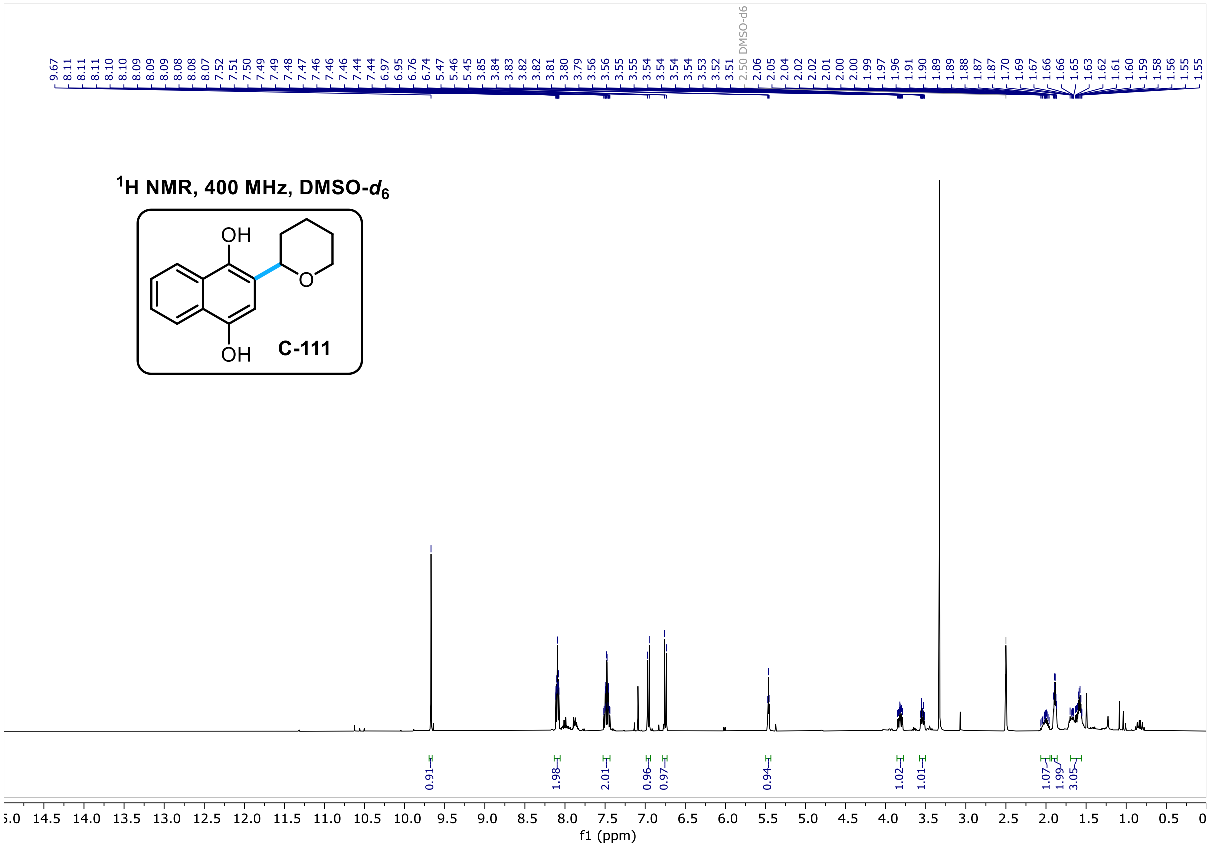
9.67 8.11 8.11 8.11 8.10 8.09 8.09 8.09 8.08 8.07 7.52 7.51 7.50 7.49 7.49 7.48 7.47 7.46 7.46 7.44 6.97 6.95 6.76 6.74 5.47 5.46 5.45 3.85 3.84 3.83 3.82 3.82 3.81 3.80 3.79 3.56 3.56 3.55 3.55 3.54 3.54 3.54 3.54 3.53 3.52 3.51 2.50 DMSO-d6 2.06 2.05 2.04 2.02 2.02 2.01 2.00 2.00 1.99 1.97 1.96 1.91 1.90 1.89 1.89 1.88 1.87 1.87 1.70 1.69 1.67 1.66 1.66 1.65 1.63 1.62 1.61 1.60 1.59 1.58 1.56 1.55 1.55
1H NMR, 400 MHz, DMSO-d6
OH
O
OH
C-111
0.91 1.98 2.01 0.96 0.97 0.94 1.02 1.01 1.07 1.99 3.05
f1 (ppm)

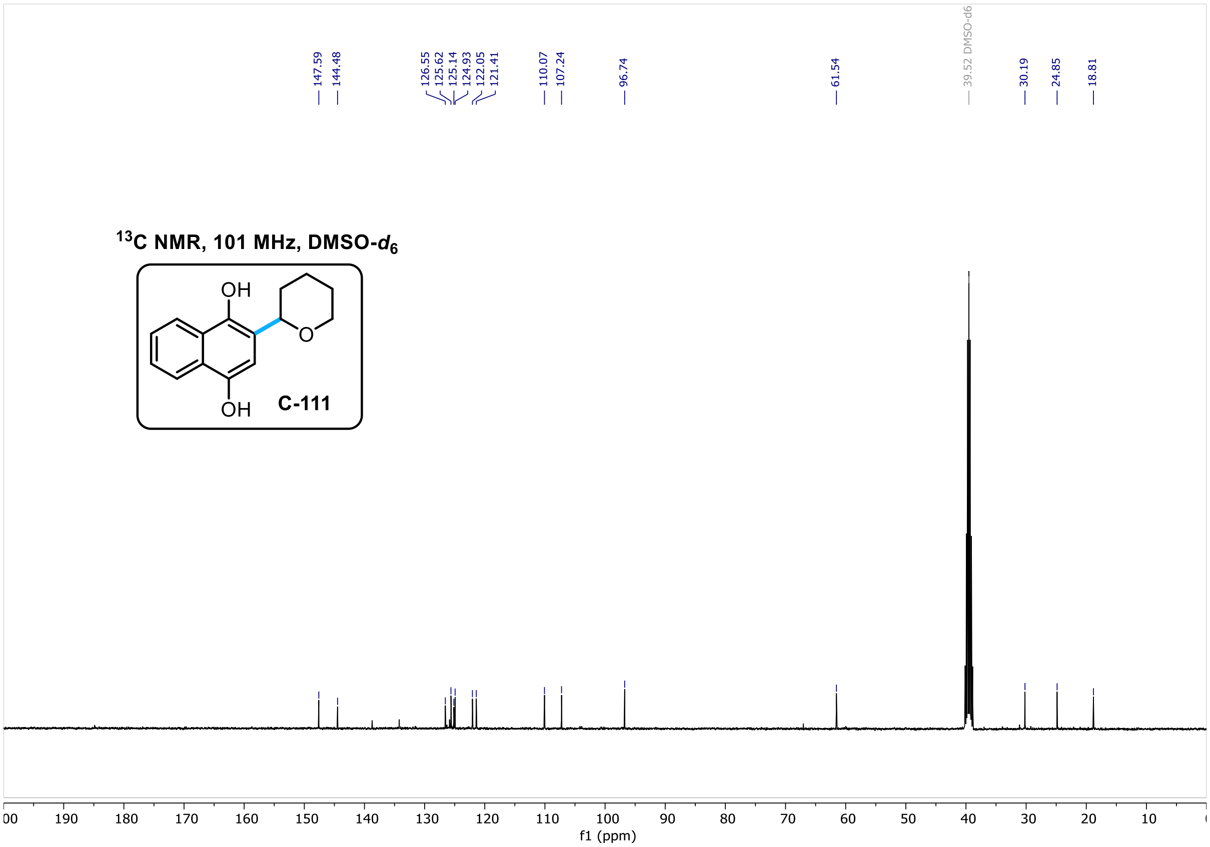
147.59 144.48 126.55 125.62 125.14 124.93 122.05 121.41 110.07 107.24 96.74 61.54 39.52 DMSO-d6 30.19 24.85 18.81
13C NMR, 101 MHz, DMSO-d6
OH
O
OH
C-111
f1 (ppm)

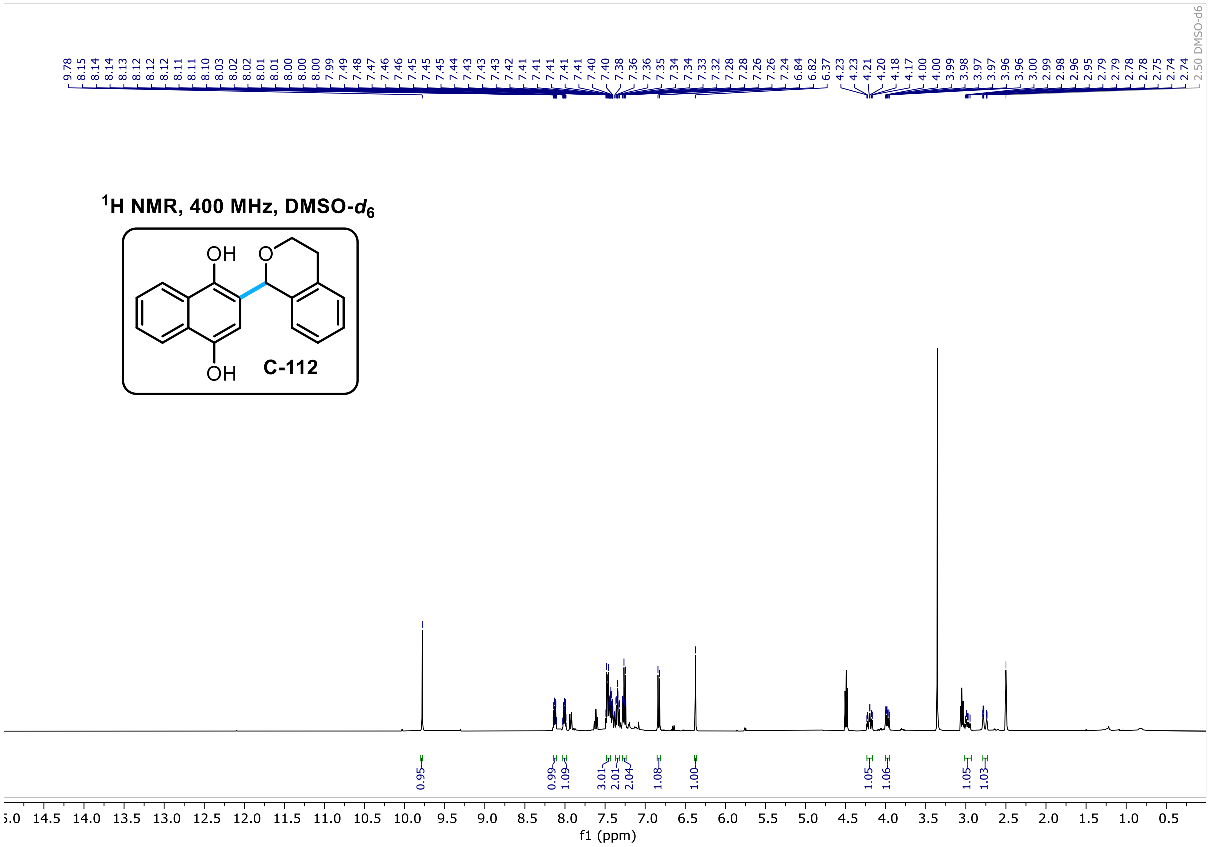
9.78 8.15 8.14 8.14 8.13 8.12 8.12 8.12 8.11 8.11 8.10 8.03 8.02 8.02 8.01 8.01 8.00 8.00 8.00 7.99 7.49 7.48 7.47 7.46 7.46 7.45 7.45 7.45 7.44 7.43 7.43 7.43 7.42 7.41 7.41 7.41 7.41 7.41 7.40 7.40 7.38 7.36 7.36 7.35 7.34 7.34 7.33 7.32 7.28 7.28 7.26 7.26 7.24 6.84 6.82 6.37 4.23 4.23 4.21 4.20 4.18 4.17 4.00 4.00 3.99 3.98 3.97 3.97 3.96 3.96 3.00 2.99 2.98 2.96 2.95 2.79 2.79 2.78 2.78 2.75 2.74 2.74
2.50 DMSO-d6
1H NMR, 400 MHz, DMSO-d6
OH
O
OH
C-112
0.95 0.99 1.09 3.01 2.01 2.04 1.08 1.00 1.05 1.06 1.05 1.03
f1 (ppm)

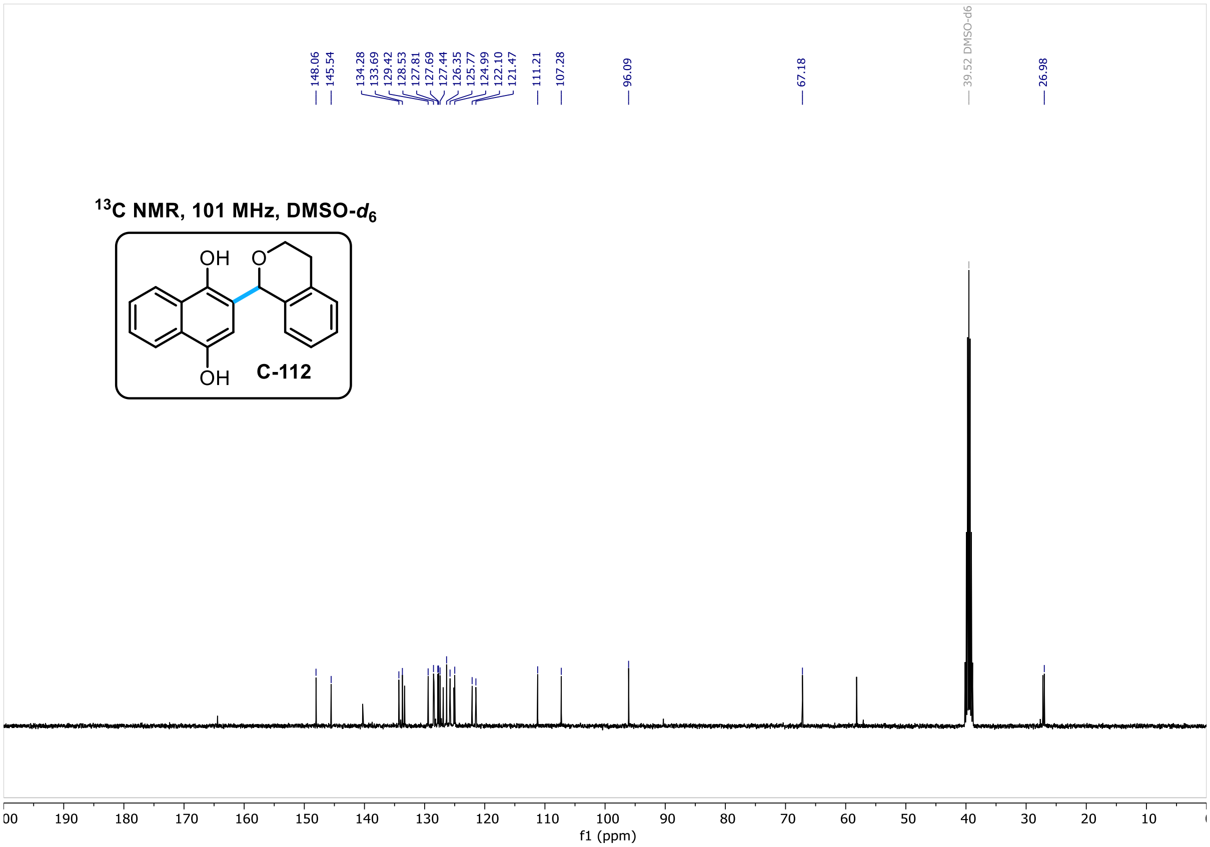
148.06 145.54 134.28 133.69 129.42 128.53 127.81 127.69 127.44 126.35 125.77 124.99 122.10 121.47 111.21 107.28 96.09 67.18
39.52 DMSO-d6
26.98
13C NMR, 101 MHz, DMSO-d6
OH
O
OH
C-112
f1 (ppm)

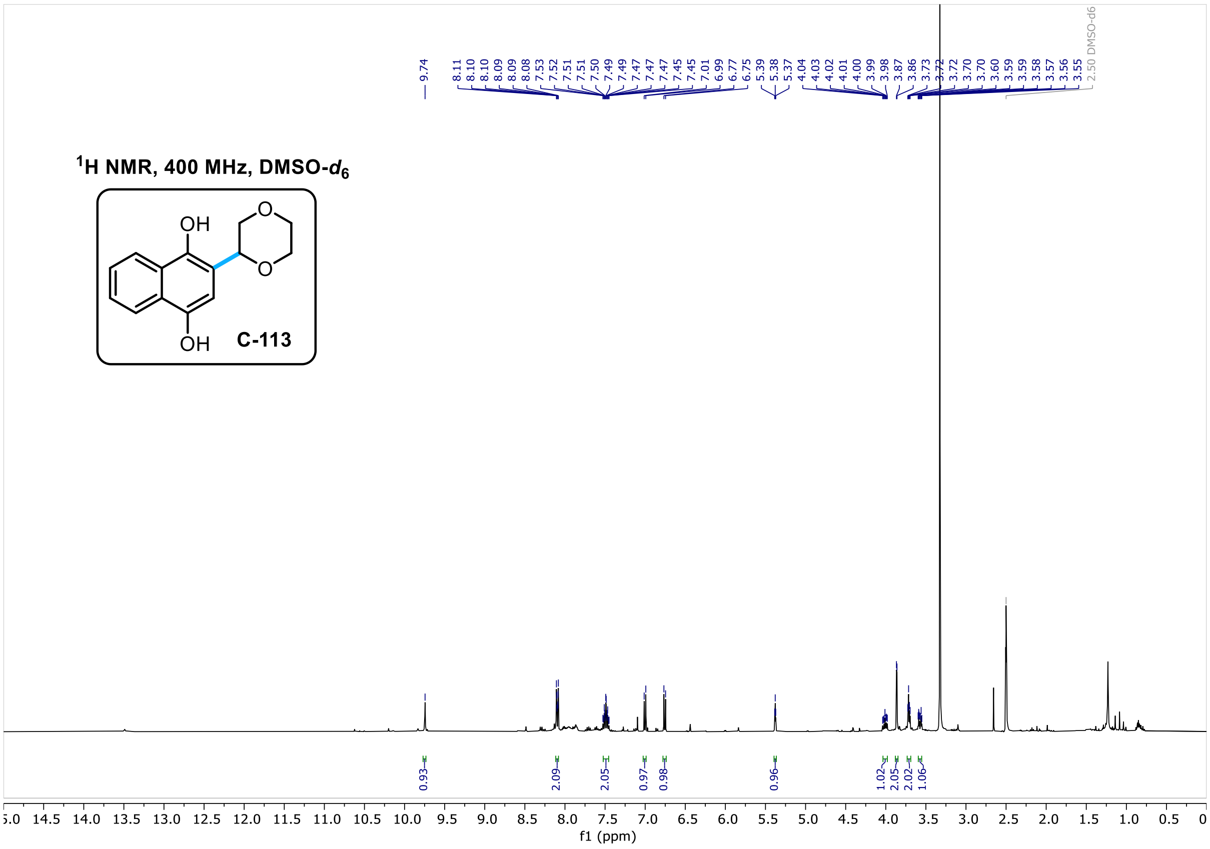

1H NMR, 400 MHz, DMSO-d6
OH
O
O
OH
C-113
f1 (ppm)

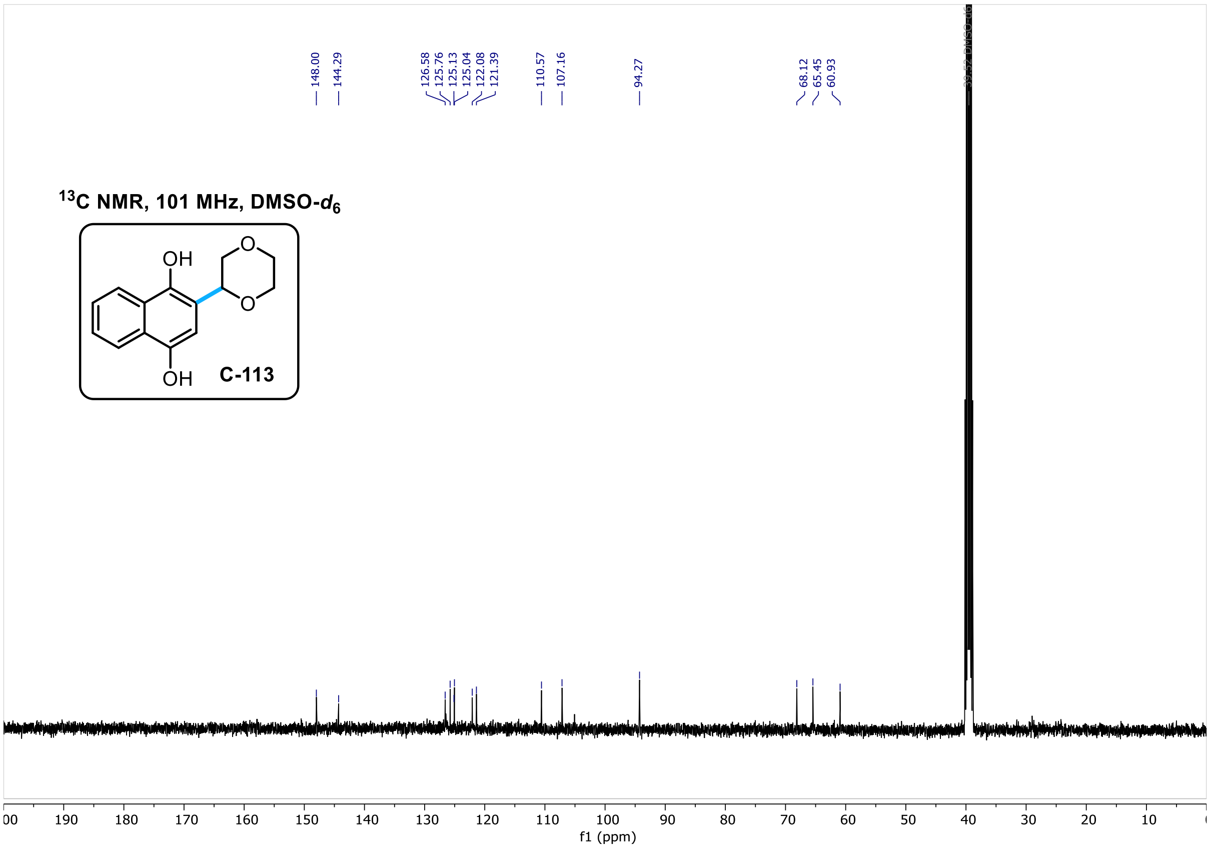

13C NMR, 101 MHz, DMSO-d6
OH
O
O
OH
C-113
148.00
144.29
126.58
125.76
125.13
125.04
122.08
121.39
110.57
107.16
94.27
68.12
65.45
60.93
f1 (ppm)

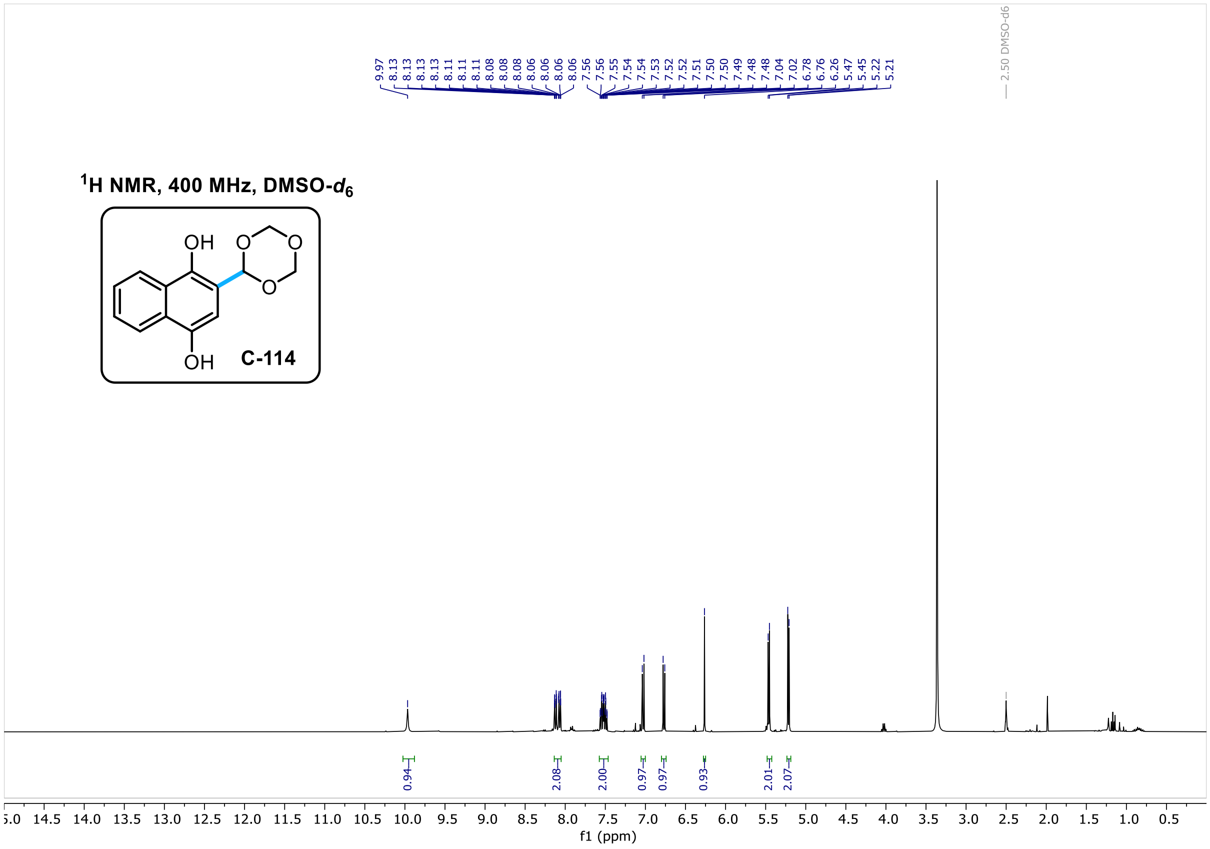

9.97 8.13 8.13 8.13 8.13 8.11 8.11 8.11 8.08 8.08 8.08 8.06 8.06 8.06 7.56 7.56 7.55 7.54 7.54 7.53 7.52 7.52 7.51 7.50 7.50 7.49 7.48 7.48 7.04 7.02 6.78 6.76 6.26 5.47 5.45 5.22 5.21
2.50 DMSO-d6
^{1}H NMR, 400 MHz, DMSO-d_6
OH
O
O
O
OH
C-114
0.94 2.08 2.00 0.97 0.97 0.93 2.01 2.07
f1 (ppm)

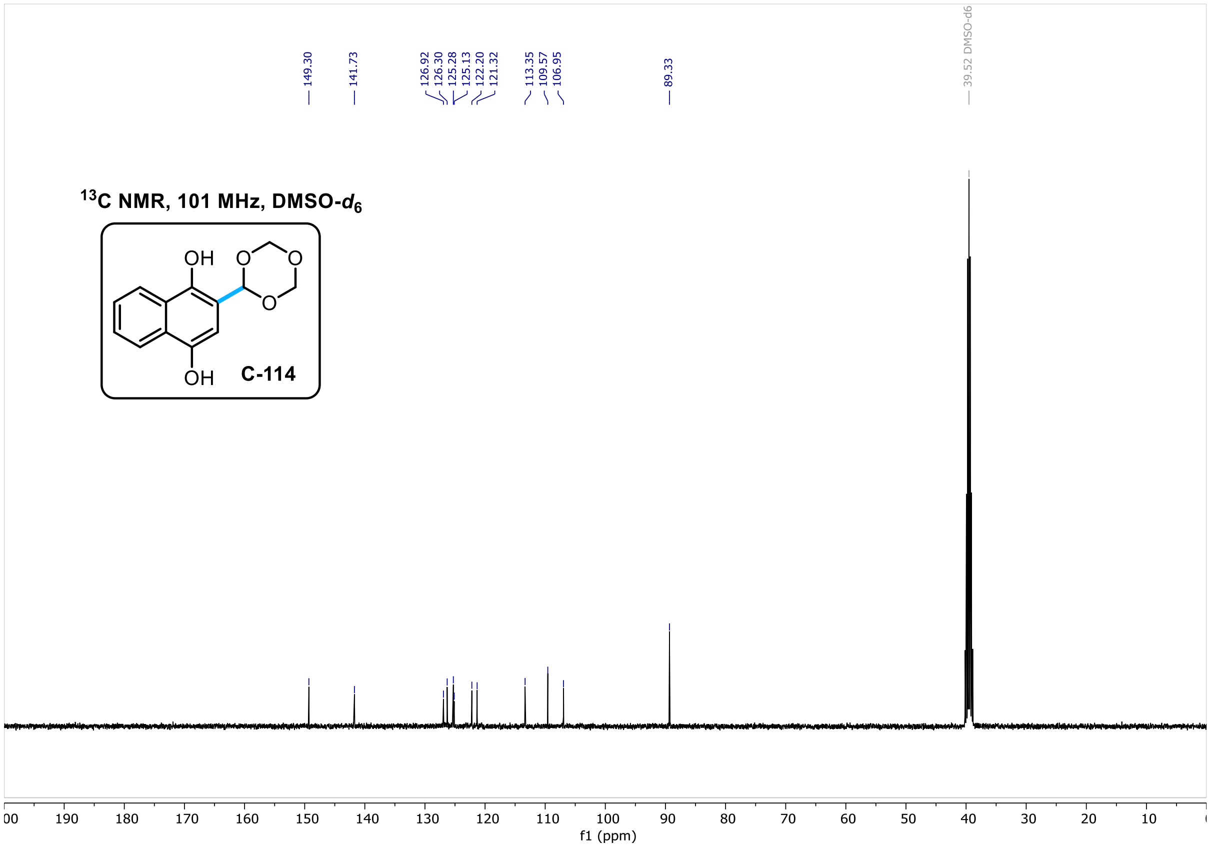

149.30 141.73 126.92 126.30 125.28 125.13 122.20 121.32 113.35 109.57 106.95 89.33
39.52 DMSO-d6
^{13}C NMR, 101 MHz, DMSO-d_6
OH
O
O
O
OH
C-114
f1 (ppm)

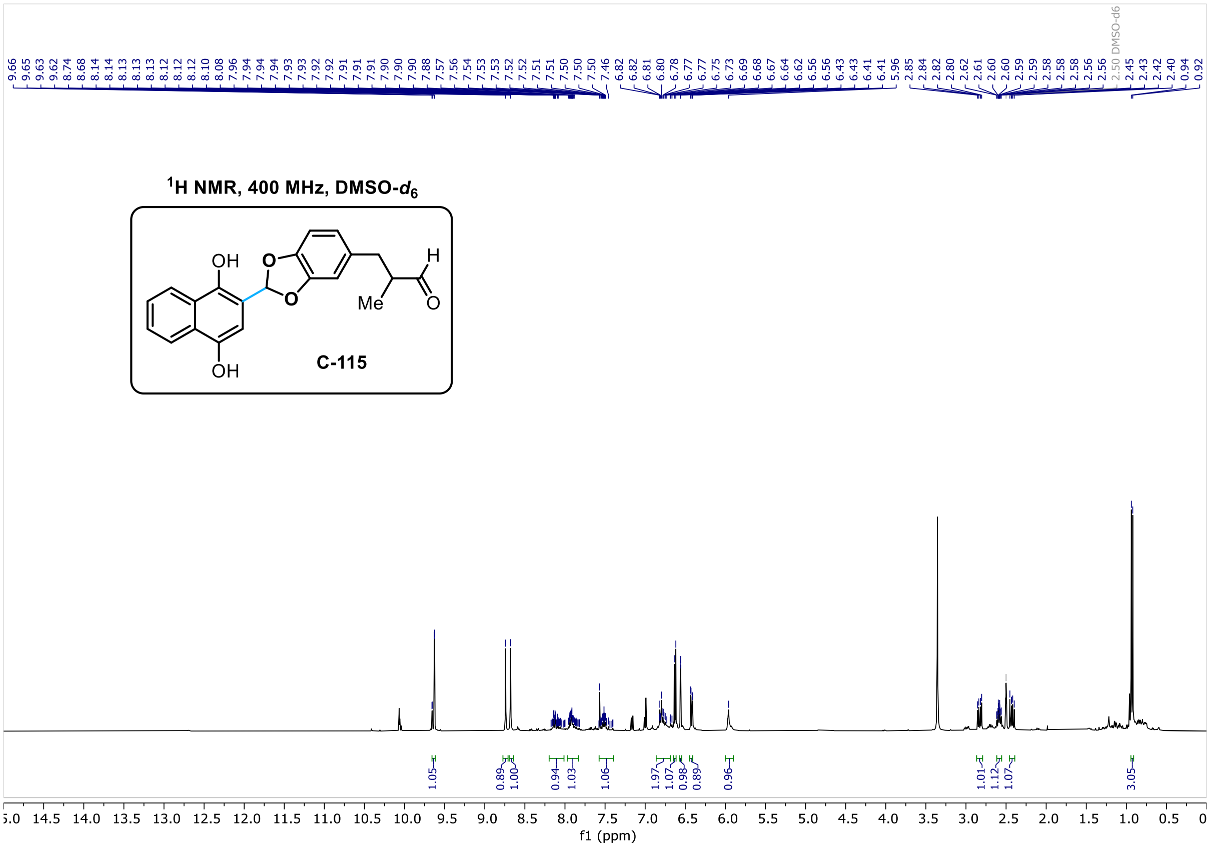
9.66 9.65 9.63 9.62 8.74 8.68 8.14 8.14 8.13 8.13 8.13 8.12 8.12 8.10 8.08 7.96 7.94 7.94 7.93 7.93 7.92 7.92 7.91 7.91 7.91 7.90 7.90 7.90 7.88 7.57 7.56 7.54 7.53 7.53 7.52 7.52 7.51 7.51 7.50 7.50 7.50 7.46 6.82 6.81 6.80 6.78 6.77 6.77 6.75 6.73 6.69 6.68 6.67 6.64 6.62 6.56 6.56 6.43 6.43 6.41 5.96 2.85 2.84 2.82 2.80 2.62 2.61 2.60 2.60 2.59 2.59 2.58 2.58 2.58 2.56 2.50 DMSO-d6 2.45 2.43 2.42 2.40 0.94 0.92
^{1}H NMR, 400 MHz, DMSO-d_6
OH
O
O
H
Me
O
OH
C-115
1.05 0.89 1.00 0.94 1.03 1.06 1.97 1.07 0.98 0.89 0.96 1.01 1.12 1.07 3.05
f1 (ppm)

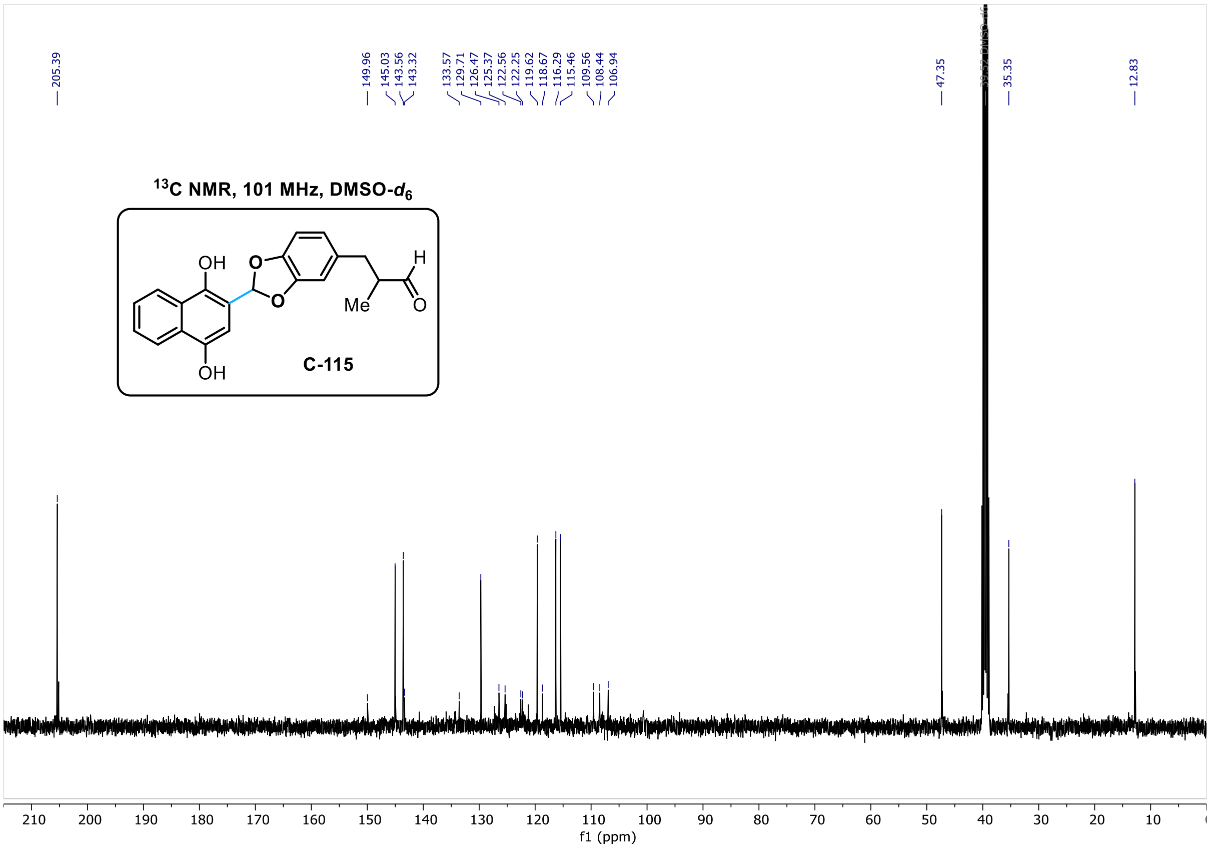
205.39 149.96 145.03 143.56 143.32 133.57 129.71 126.47 125.37 122.56 122.25 119.62 118.67 116.29 115.46 109.56 108.44 106.94 47.35 35.35 12.83
^{13}C NMR, 101 MHz, DMSO-d_6
OH
O
O
H
Me
O
OH
C-115
f1 (ppm)

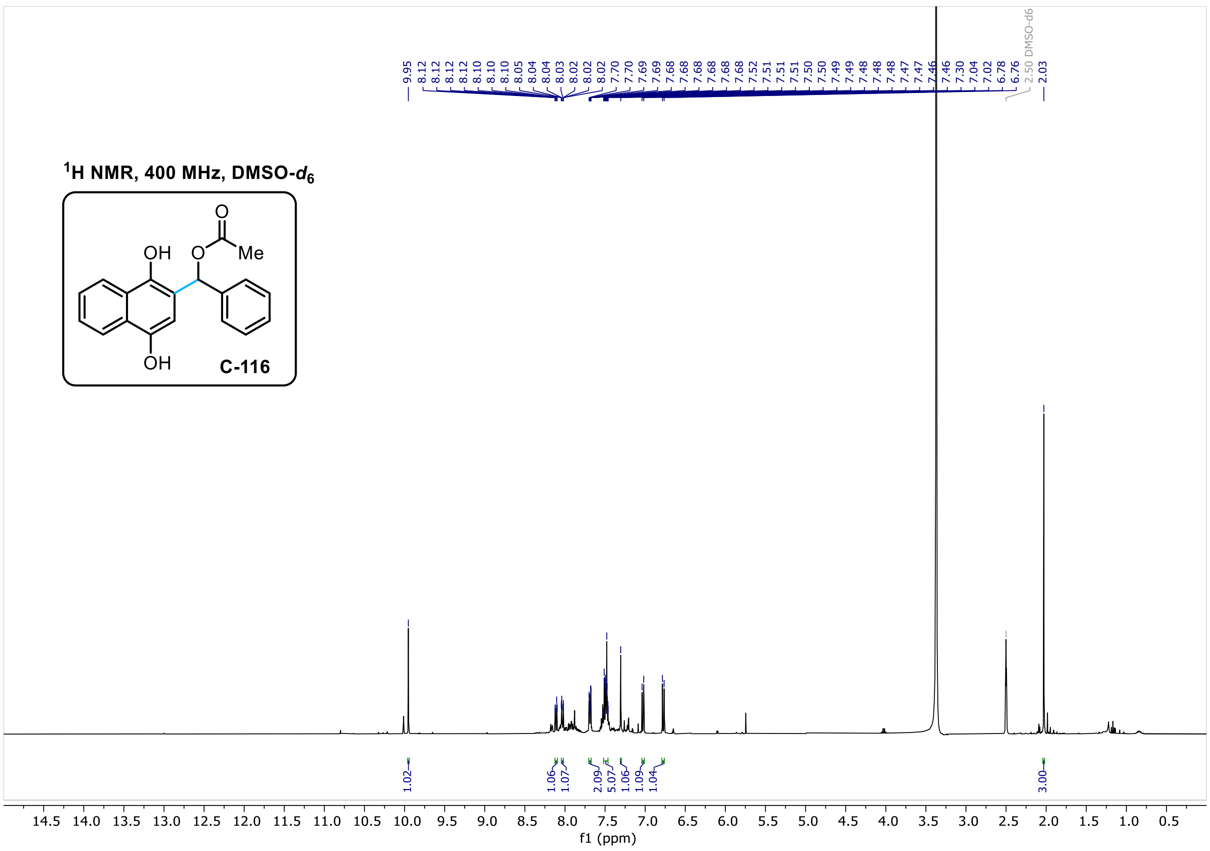
9.95
8.12
8.12
8.12
8.12
8.10
8.10
8.10
8.05
8.04
8.03
8.02
8.02
8.02
7.70
7.70
7.69
7.69
7.68
7.68
7.68
7.68
7.68
7.68
7.52
7.51
7.51
7.51
7.50
7.50
7.49
7.49
7.48
7.48
7.48
7.47
7.46
7.46
7.30
7.04
7.04
7.02
6.78
6.76
2.50 DMSO-d6
2.03
¹H NMR, 400 MHz, DMSO-d6
OH
O
O
Me
OH
C-116
1.02
1.06
1.07
2.09
5.07
1.06
1.09
1.04
3.00
14.5 14.0 13.5 13.0 12.5 12.0 11.5 11.0 10.5 10.0 9.5 9.0 8.5 8.0 7.5 7.0 6.5 6.0 5.5 5.0 4.5 4.0 3.5 3.0 2.5 2.0 1.5 1.0 0.5
f1 (ppm)

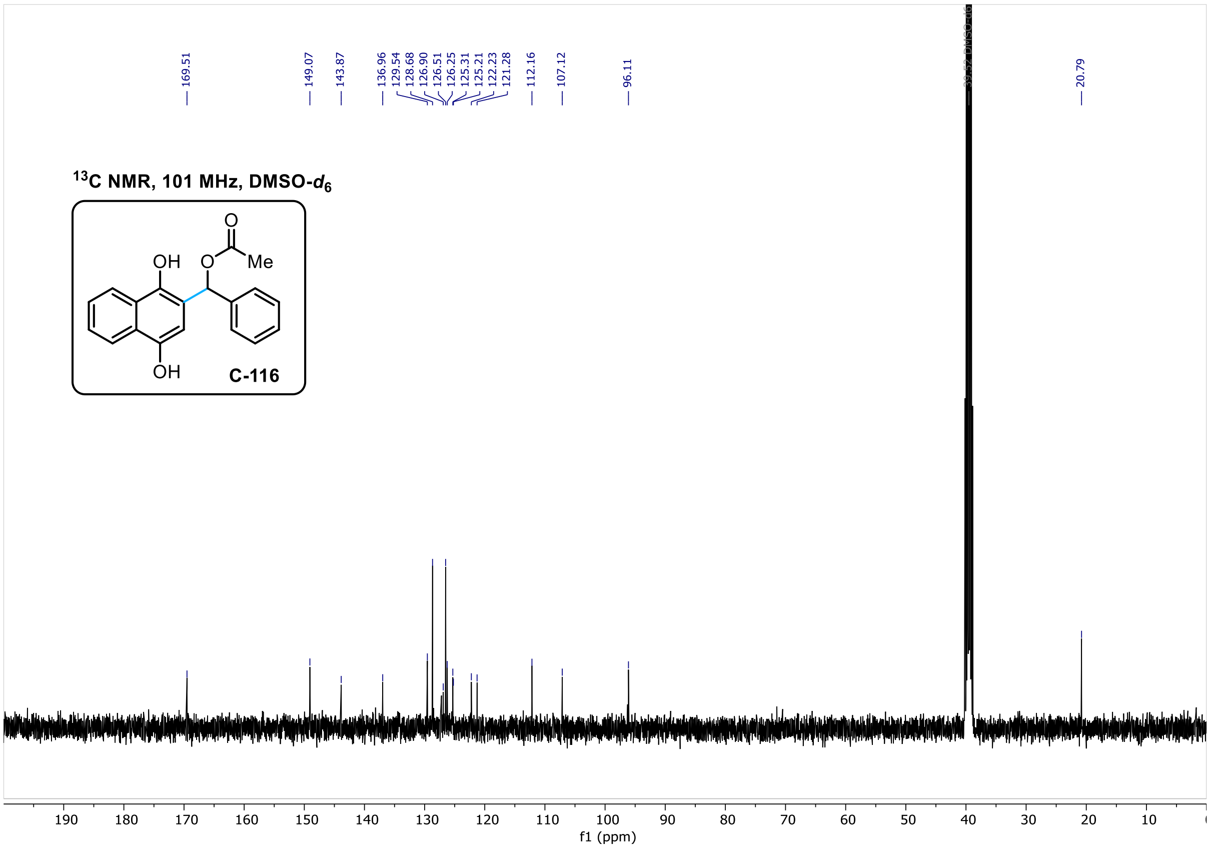
169.51
149.07
143.87
136.96
129.54
128.68
126.90
126.51
126.25
125.31
125.21
122.23
121.28
112.16
107.12
96.11
20.79
¹³C NMR, 101 MHz, DMSO-d6
OH
O
O
Me
OH
C-116
190 180 170 160 150 140 130 120 110 100 90 80 70 60 50 40 30 20 10
f1 (ppm)

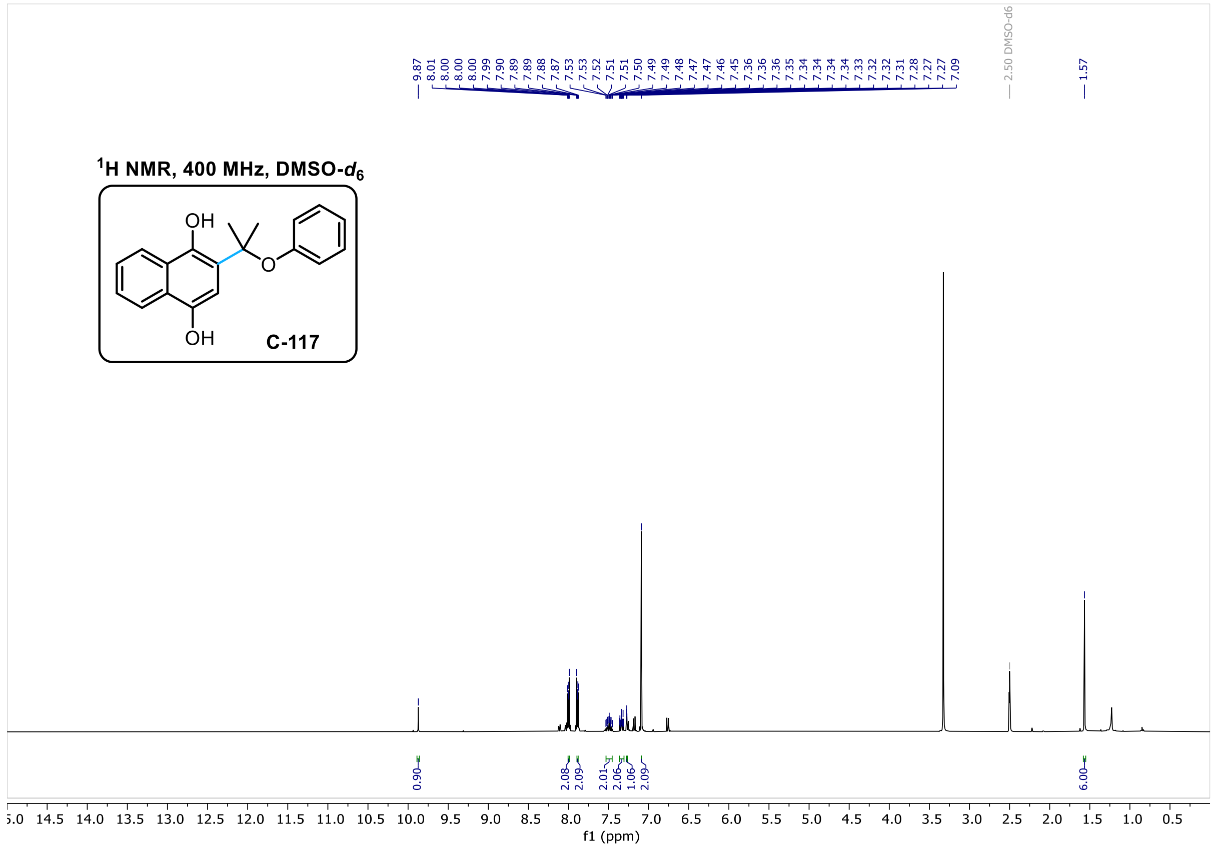
9.87
8.01
8.00
8.00
8.00
7.99
7.90
7.89
7.89
7.88
7.87
7.53
7.53
7.52
7.51
7.51
7.50
7.49
7.49
7.48
7.47
7.47
7.46
7.45
7.36
7.36
7.36
7.35
7.34
7.34
7.34
7.34
7.33
7.32
7.32
7.31
7.28
7.27
7.27
7.09
2.50 DMSO-d6
1.57
¹H NMR, 400 MHz, DMSO-d6
OH
O
OH
C-117
0.90
2.08
2.09
2.01
2.06
1.06
2.09
6.00
f1 (ppm)

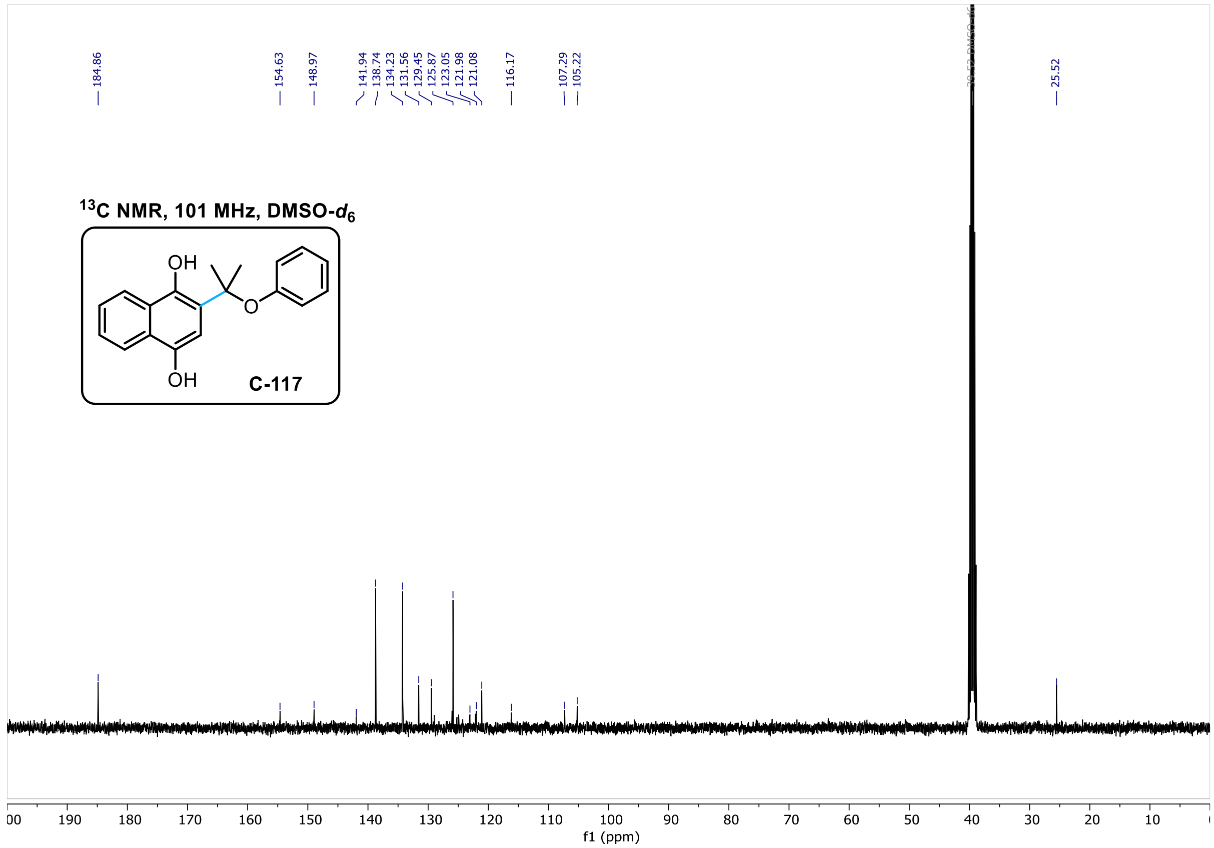
184.86
154.63
148.97
141.94
138.74
134.23
131.56
129.45
125.87
123.05
121.98
121.08
116.17
107.29
105.22
25.52
¹³C NMR, 101 MHz, DMSO-d6
OH
O
OH
C-117
f1 (ppm)

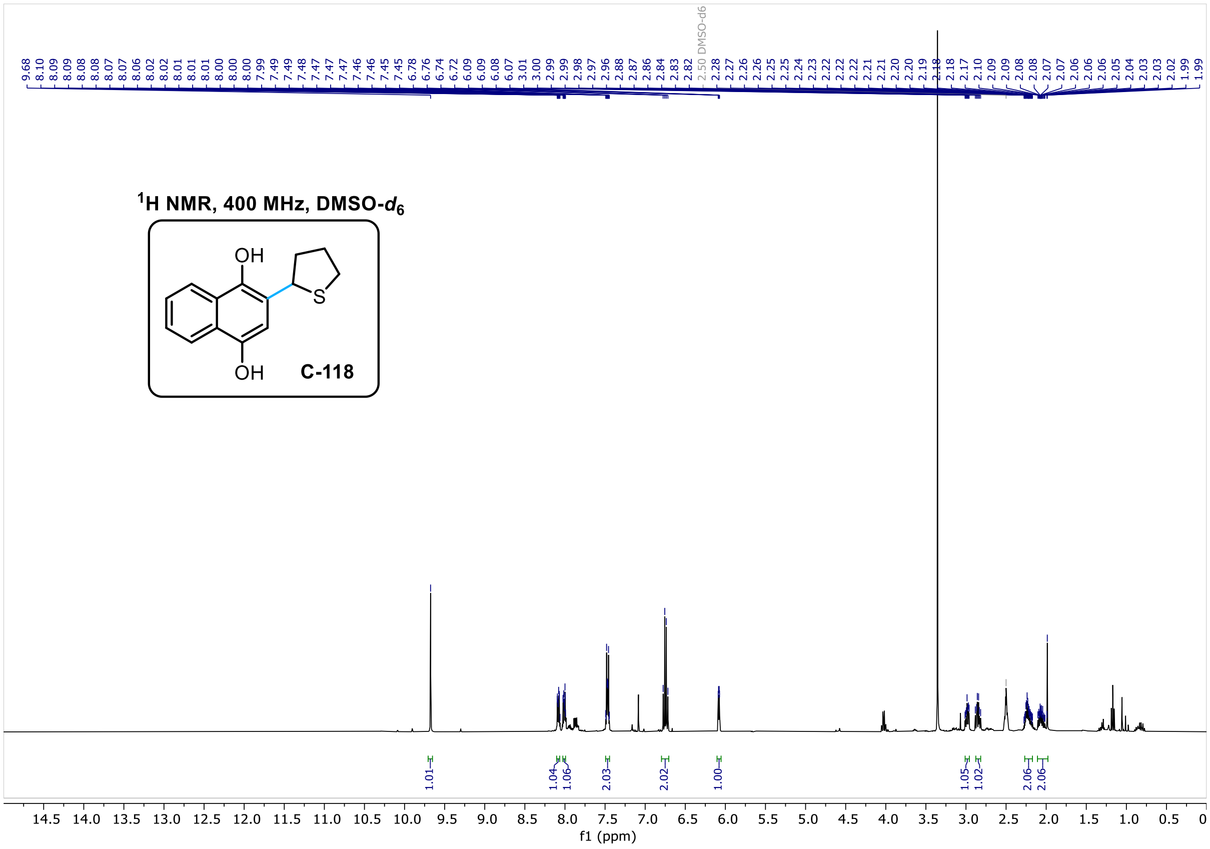
9.68 8.10 8.09 8.09 8.08 8.08 8.07 8.06 8.02 8.02 8.01 8.01 8.00 8.00 7.99 7.49 7.49 7.48 7.47 7.47 7.47 7.46 7.45 7.45 6.78 6.76 6.74 6.72 6.09 6.09 6.08 6.07 3.01 3.00 2.99 2.99 2.98 2.97 2.96 2.88 2.87 2.86 2.84 2.83 2.82 2.50 DMSO-d6 2.28 2.27 2.26 2.26 2.25 2.25 2.24 2.23 2.22 2.22 2.22 2.21 2.21 2.20 2.20 2.19 2.18 2.18 2.17 2.10 2.09 2.09 2.08 2.08 2.07 2.07 2.06 2.06 2.06 2.05 2.04 2.03 2.03 2.02 1.99 1.99
1H NMR, 400 MHz, DMSO-d6
OH
S
OH
C-118
1.01 1.04 1.06 2.03 2.02 1.00 1.05 1.02 2.06 2.06
f1 (ppm)

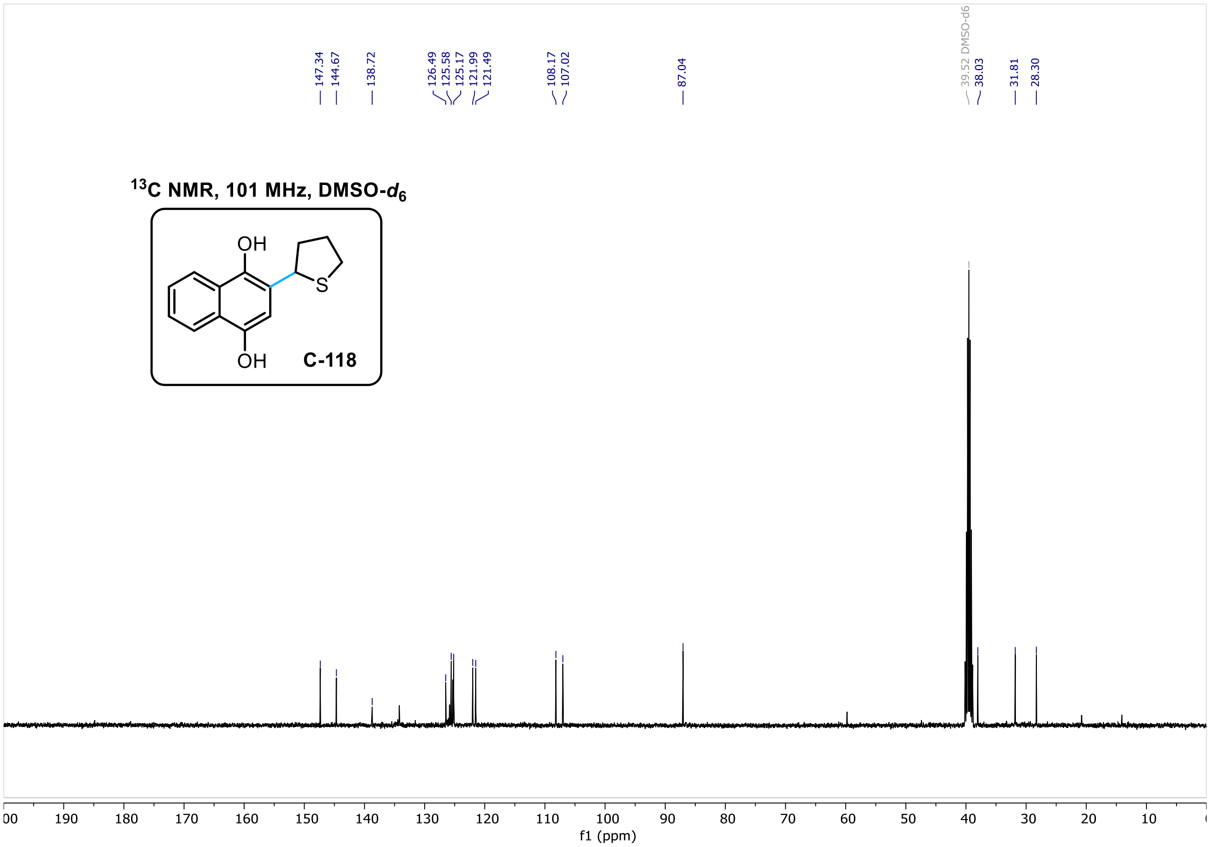
147.34 144.67 138.72 126.49 125.58 125.17 121.99 121.49 108.17 107.02 87.04 39.52 DMSO-d6 38.03 31.81 28.30
13C NMR, 101 MHz, DMSO-d6
OH
S
OH
C-118
f1 (ppm)

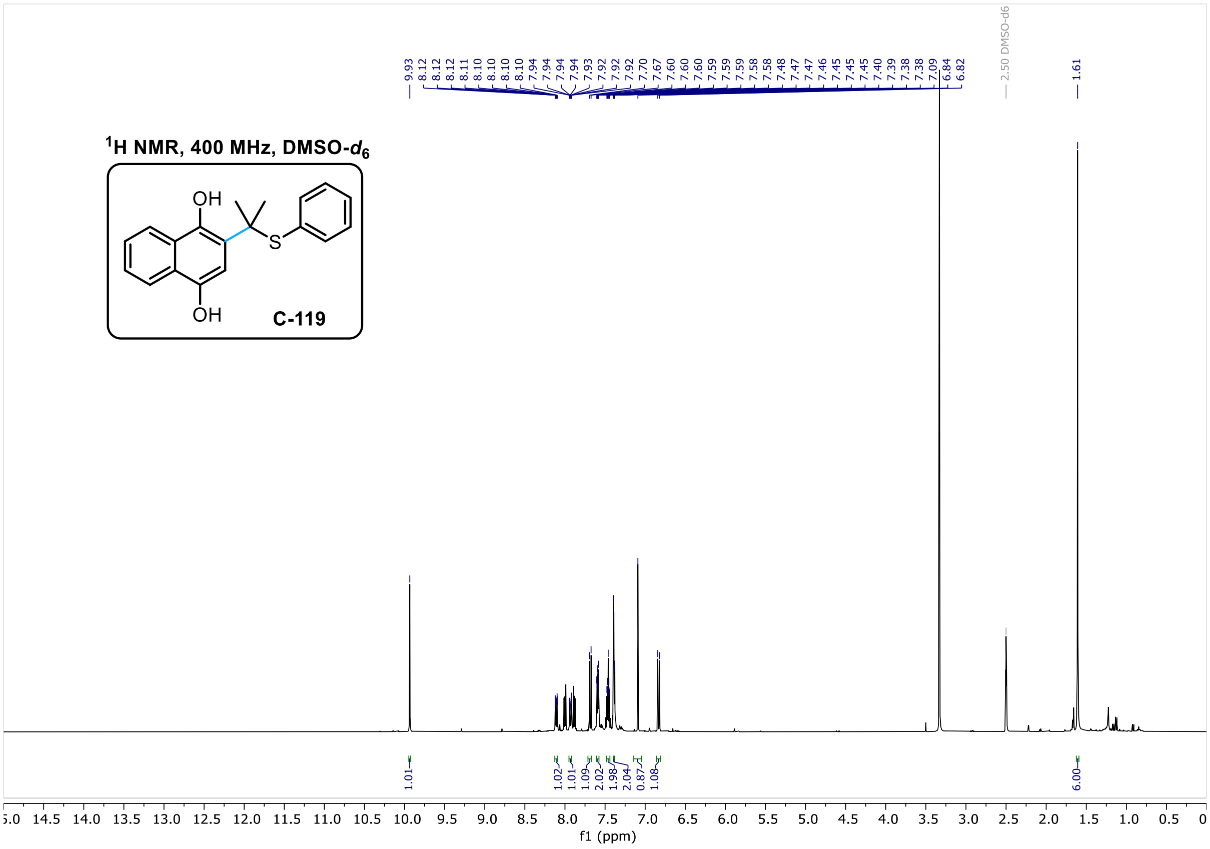

1H NMR, 400 MHz, DMSO-d6
OH
S
OH
C-119
9.93 8.12 8.12 8.11 8.10 8.10 8.10 8.10 7.94 7.94 7.94 7.93 7.92 7.92 7.92 7.92 7.70 7.67 7.60 7.60 7.59 7.59 7.59 7.58 7.58 7.48 7.47 7.46 7.45 7.45 7.45 7.40 7.39 7.38 7.38 7.09 6.84 6.82
2.50 DMSO-d6
1.61
1.01 1.02 1.01 1.09 2.02 1.98 2.04 0.87 1.08 6.00
f1 (ppm)

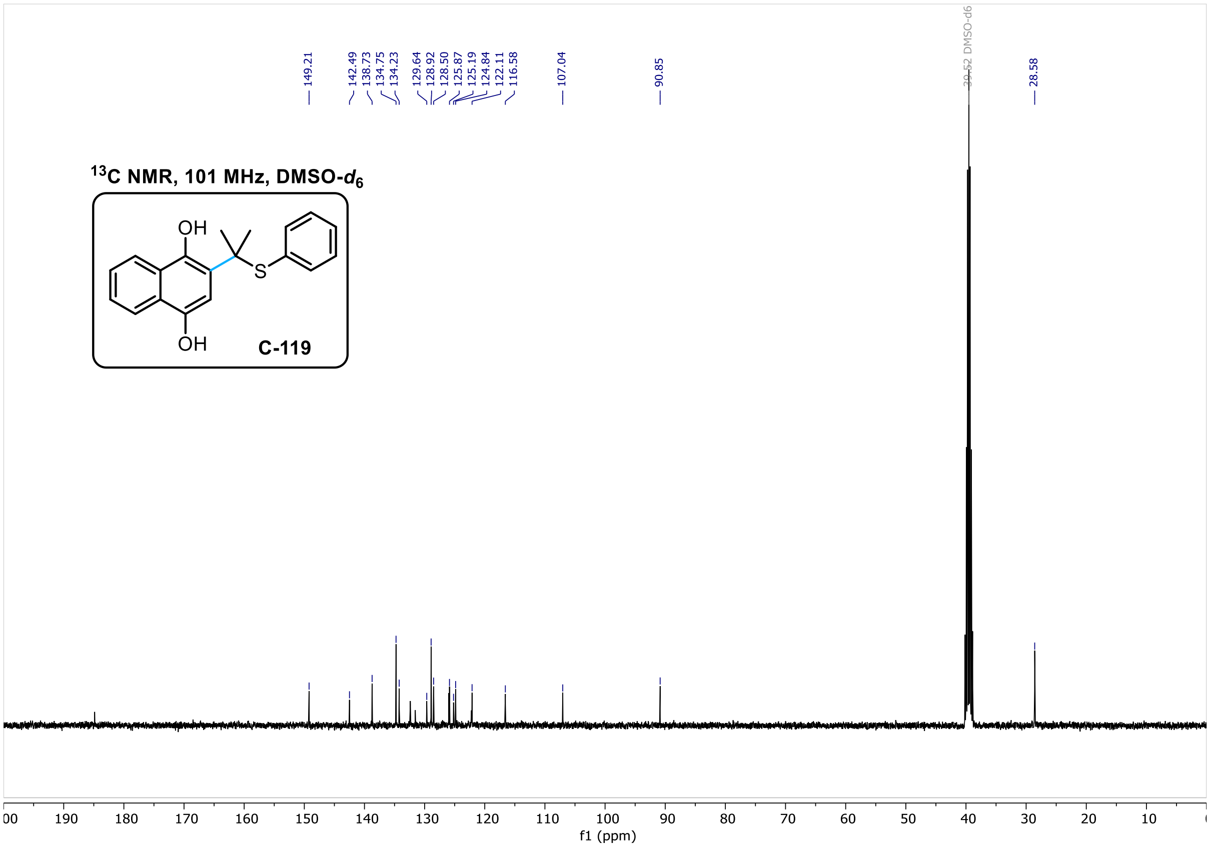

13C NMR, 101 MHz, DMSO-d6
OH
S
OH
C-119
149.21 142.49 138.73 134.75 134.23 129.64 128.92 128.50 125.87 125.19 124.84 122.11 116.58 107.04 90.85
39.52 DMSO-d6
28.58
f1 (ppm)

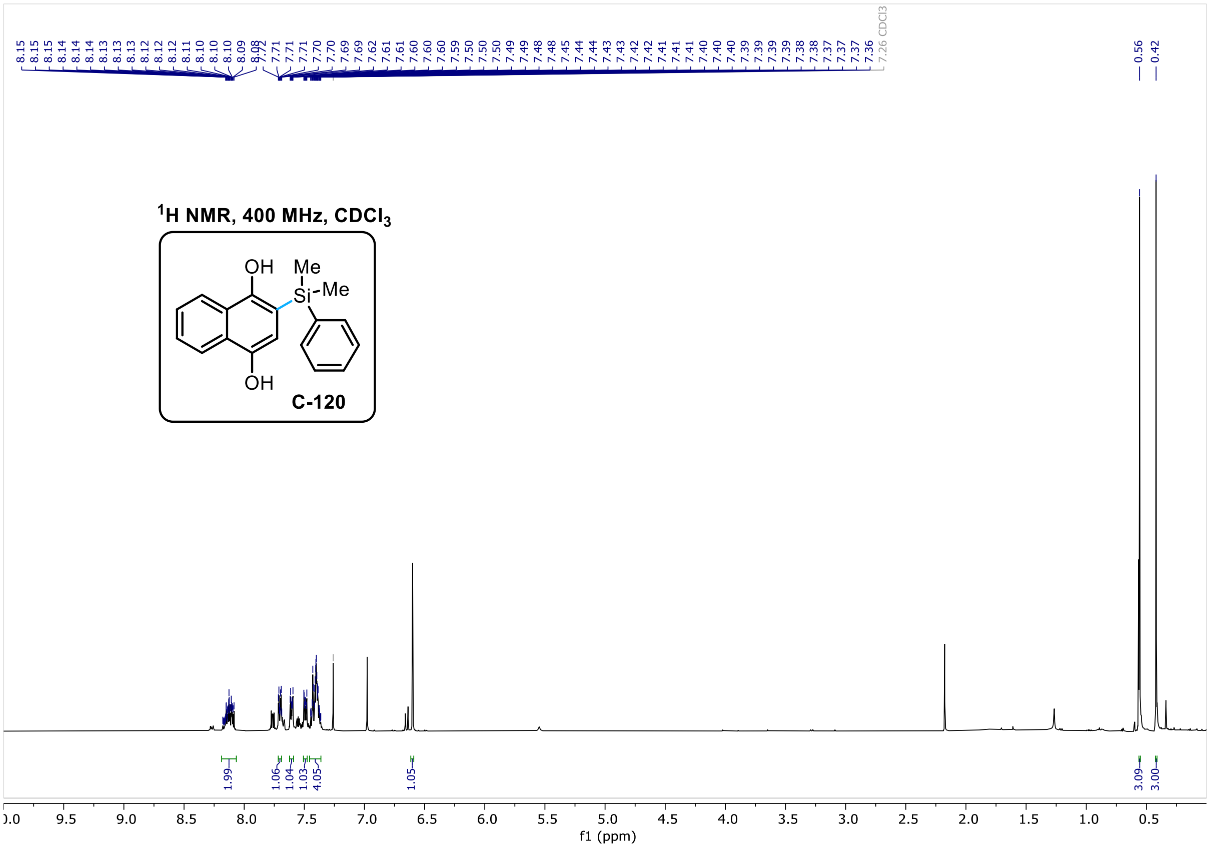
0.56
0.42
1H NMR, 400 MHz, CDCl3
OH
Me
Si
Me
OH
C-120
1.99
1.06
1.04
1.03
4.05
1.05
3.09
3.00
f1 (ppm)

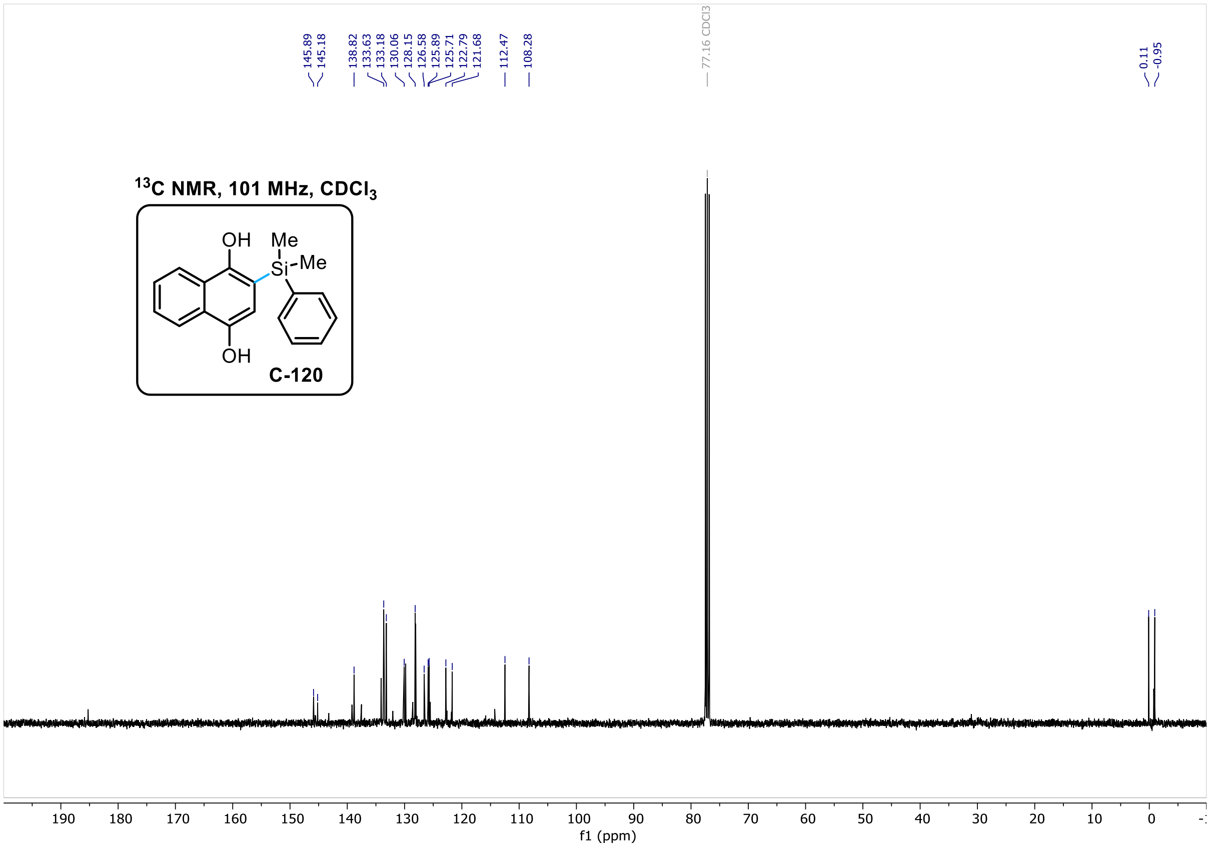
145.89
145.18
138.82
133.63
133.18
130.06
128.15
126.58
125.89
125.71
122.79
121.68
112.47
108.28
77.16 CDCl3
0.11
-0.95
13C NMR, 101 MHz, CDCl3
OH
Me
Si
Me
OH
C-120
f1 (ppm)

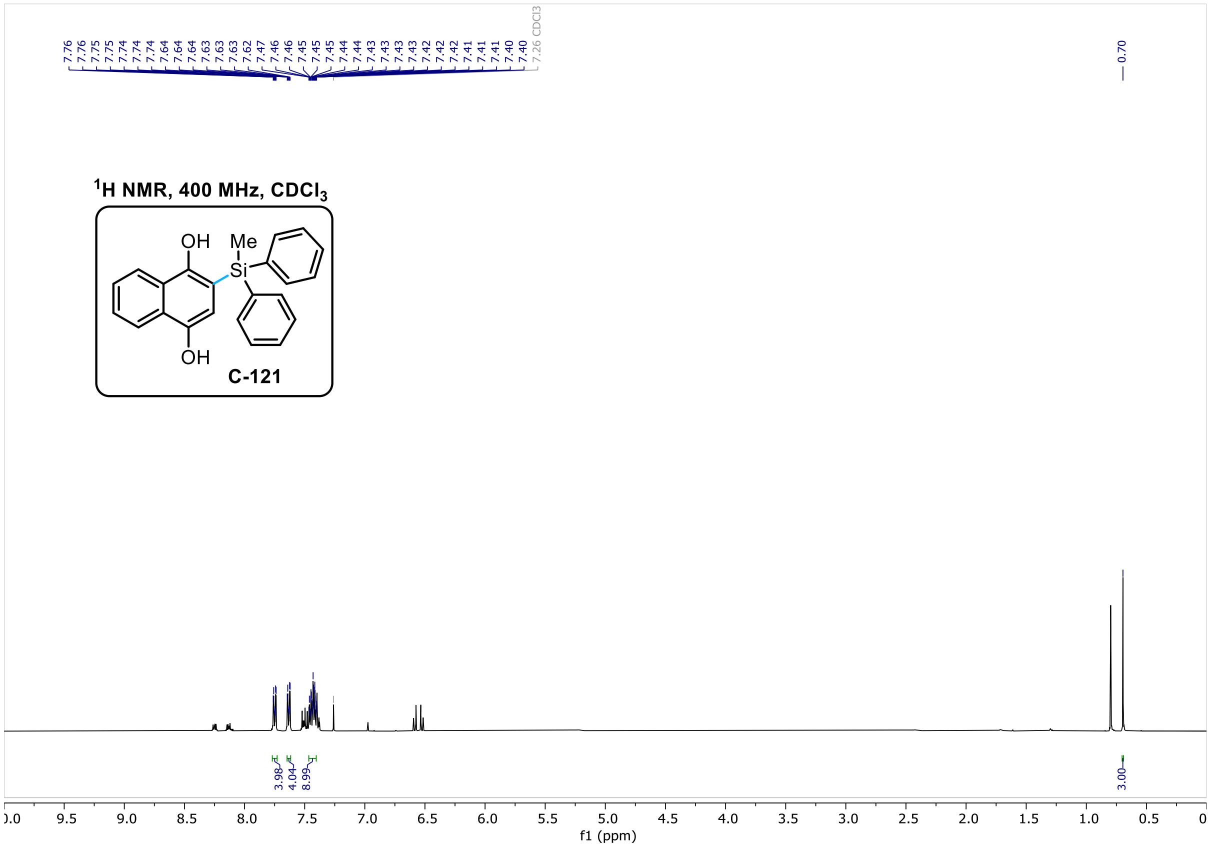
7.76
7.76
7.75
7.75
7.74
7.74
7.74
7.64
7.64
7.63
7.63
7.63
7.62
7.47
7.46
7.46
7.45
7.45
7.45
7.44
7.44
7.43
7.43
7.43
7.43
7.42
7.42
7.42
7.41
7.41
7.41
7.40
7.40
7.26 CDCl3
0.70
1H NMR, 400 MHz, CDCl3
OH
Me
Si
OH
C-121
3.98
4.04
8.99
3.00
f1 (ppm)

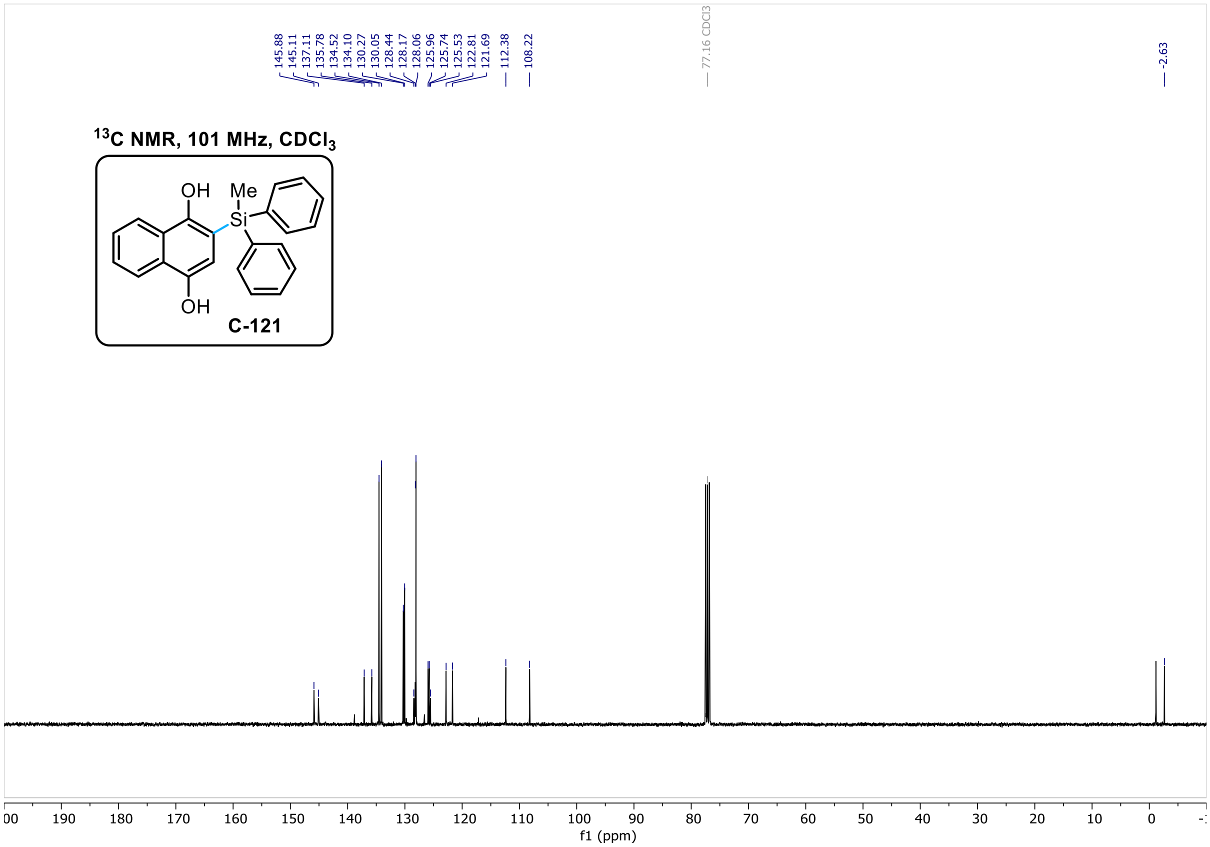
145.88
145.11
137.11
135.78
134.52
134.10
130.27
130.05
128.44
128.17
128.06
125.96
125.74
125.53
122.81
121.69
112.38
108.22
77.16 CDCl3
-2.63
13C NMR, 101 MHz, CDCl3
OH
Me
Si
OH
C-121
f1 (ppm)

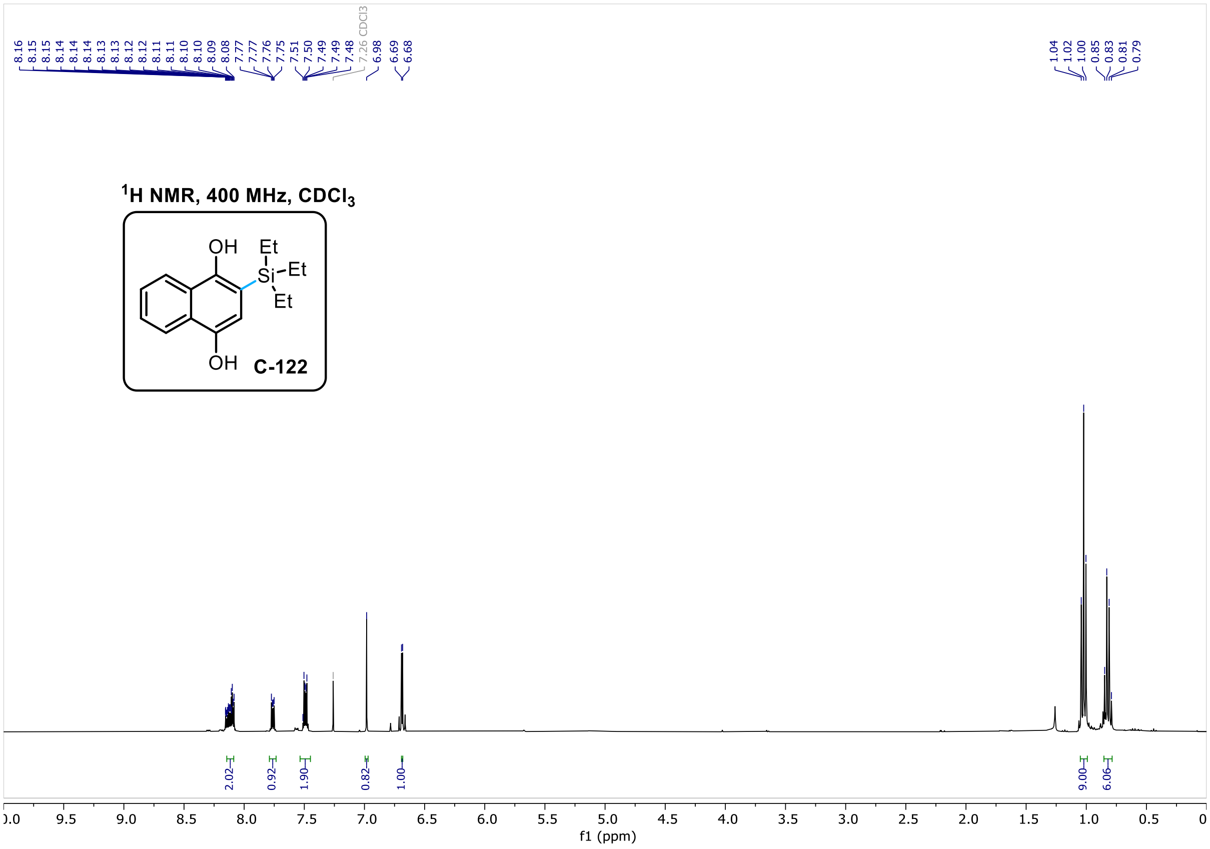
8.16
8.15
8.15
8.14
8.14
8.14
8.13
8.13
8.12
8.12
8.11
8.11
8.10
8.10
8.09
8.08
7.77
7.77
7.76
7.75
7.51
7.50
7.49
7.49
7.48
7.26 CDCl3
6.98
6.69
6.68
1.04
1.02
1.00
0.85
0.83
0.81
0.79
1H NMR, 400 MHz, CDCl3
OH
Et
Si
Et
Et
OH
C-122
2.02
0.92
1.90
0.82
1.00
9.00
6.06
f1 (ppm)

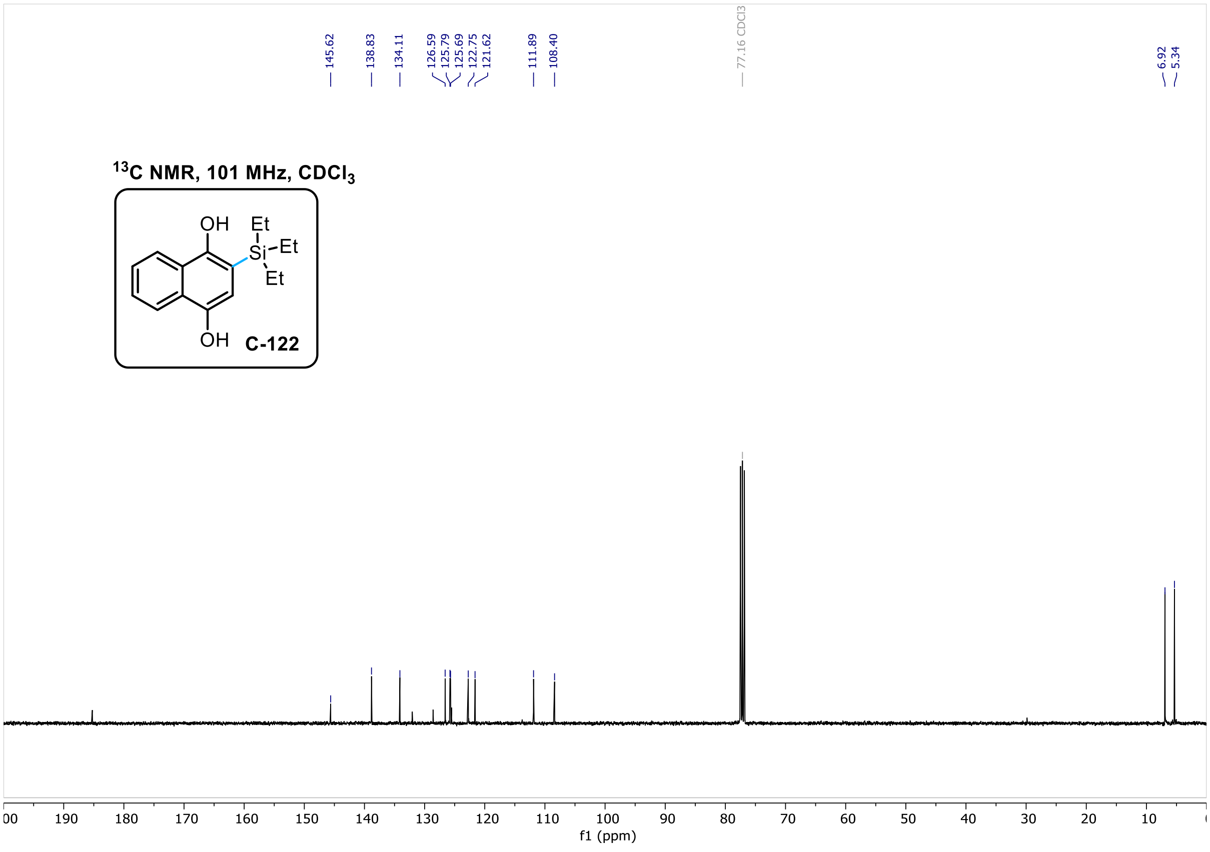
145.62
138.83
134.11
126.59
125.79
125.69
122.75
121.62
111.89
108.40
77.16 CDCl3
6.92
5.34
13C NMR, 101 MHz, CDCl3
OH
Et
Si
Et
Et
OH
C-122
f1 (ppm)

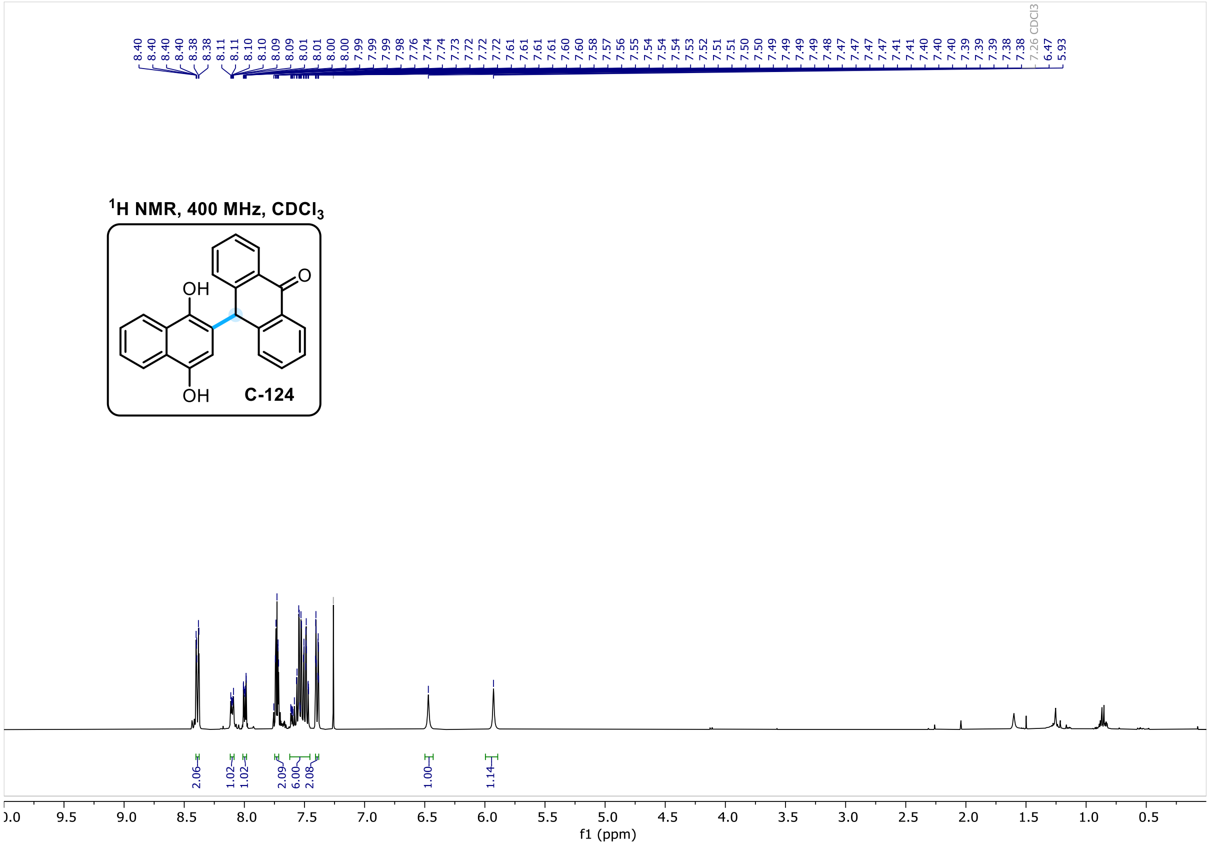

8.40 8.40 8.40 8.38 8.38 8.11 8.11 8.10 8.10 8.09 8.09 8.01 8.01 8.00 8.00 7.99 7.99 7.99 7.98 7.76 7.74 7.74 7.73 7.72 7.72 7.72 7.61 7.61 7.61 7.61 7.60 7.60 7.58 7.57 7.56 7.55 7.54 7.54 7.54 7.53 7.52 7.51 7.51 7.50 7.50 7.49 7.49 7.49 7.49 7.48 7.47 7.47 7.47 7.47 7.41 7.41 7.40 7.40 7.40 7.39 7.39 7.39 7.38 7.26 CDCl3 6.47 5.93
1H NMR, 400 MHz, CDCl3
OH
O
OH
C-124
2.06 1.02 1.02 2.09 6.00 2.08 1.00 1.14
f1 (ppm)

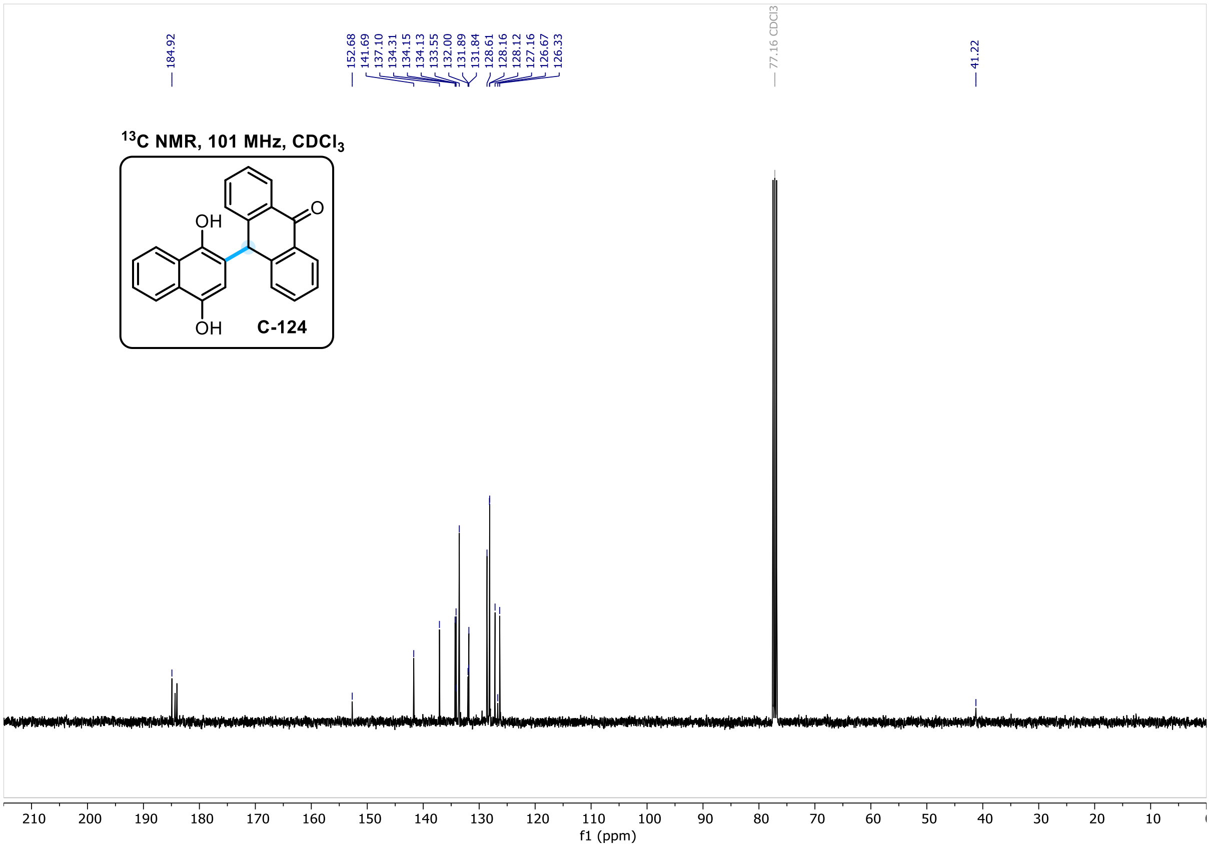

184.92
152.68 141.69 137.10 134.31 134.15 134.13 133.55 132.00 131.89 131.84 128.61 128.16 128.12 127.16 126.67 126.33
77.16 CDCl3
41.22
13C NMR, 101 MHz, CDCl3
OH
O
OH
C-124
f1 (ppm)

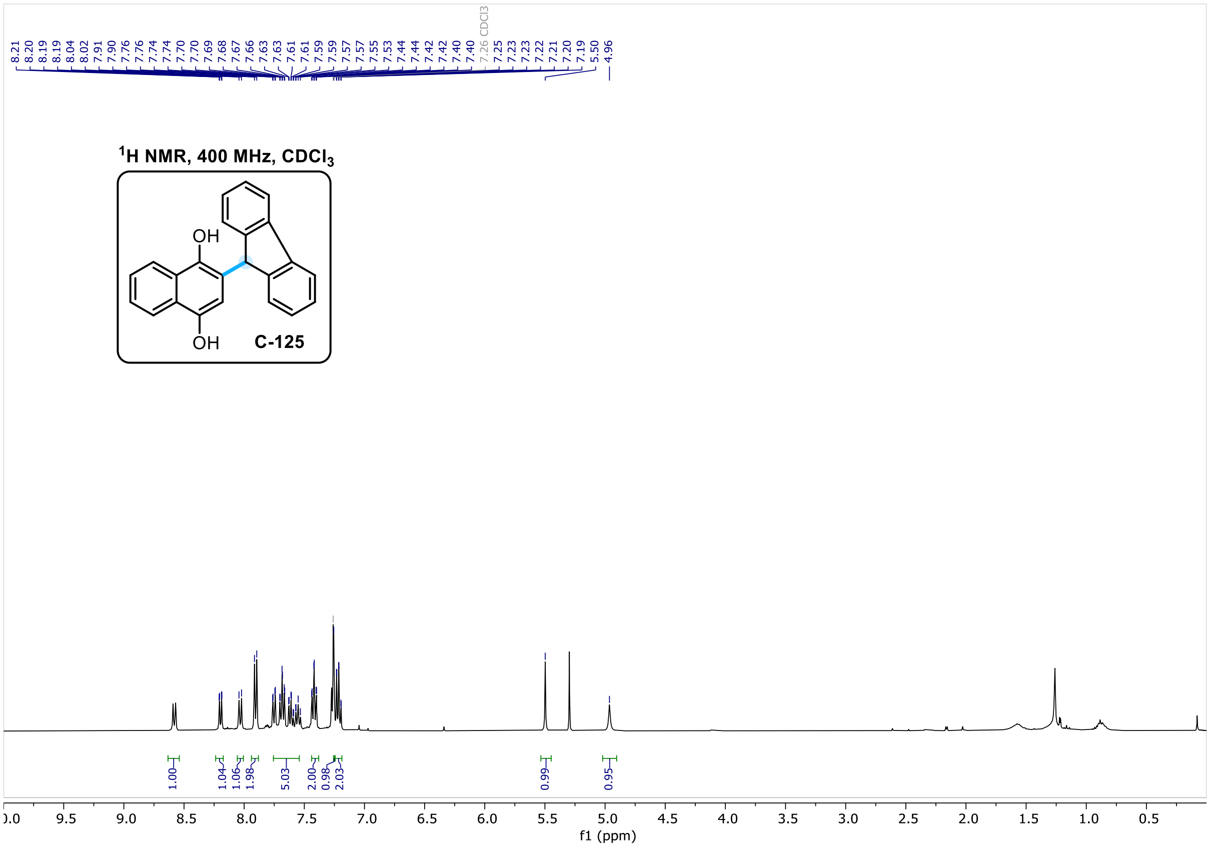
8.21 8.20 8.19 8.19 8.04 8.02 7.91 7.90 7.76 7.76 7.74 7.74 7.70 7.70 7.69 7.68 7.67 7.66 7.63 7.63 7.61 7.61 7.59 7.59 7.57 7.57 7.55 7.53 7.44 7.44 7.42 7.42 7.40 7.40 7.26 CDCl3 7.25 7.23 7.23 7.22 7.21 7.20 7.19 5.50 4.96
1H NMR, 400 MHz, CDCl3
OH
OH
C-125
1.00 1.04 1.06 1.98 5.03 2.00 0.98 2.03 0.99 0.95
f1 (ppm)

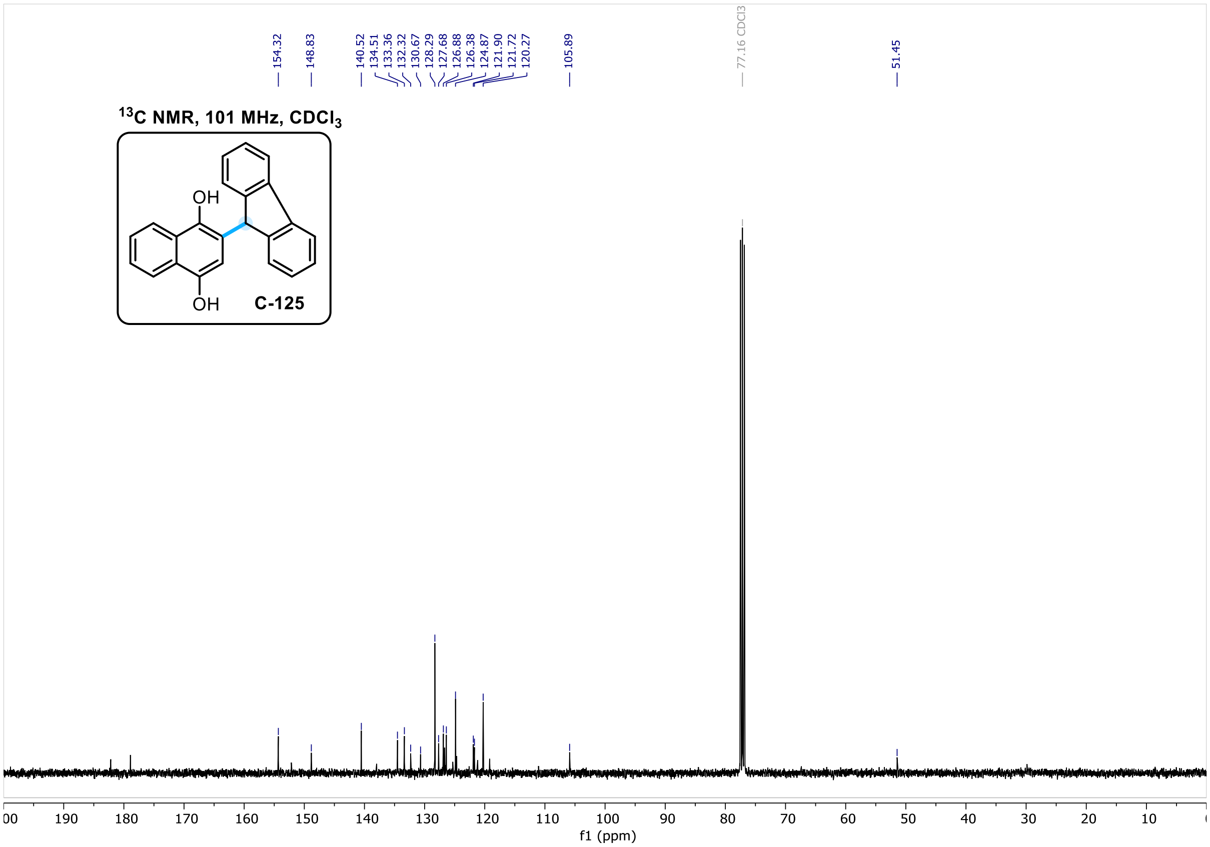
154.32 148.83 140.52 134.51 133.36 132.32 130.67 128.29 127.68 126.88 126.38 124.87 121.90 121.72 120.27 105.89 77.16 CDCl3 51.45
13C NMR, 101 MHz, CDCl3
OH
OH
C-125
f1 (ppm)

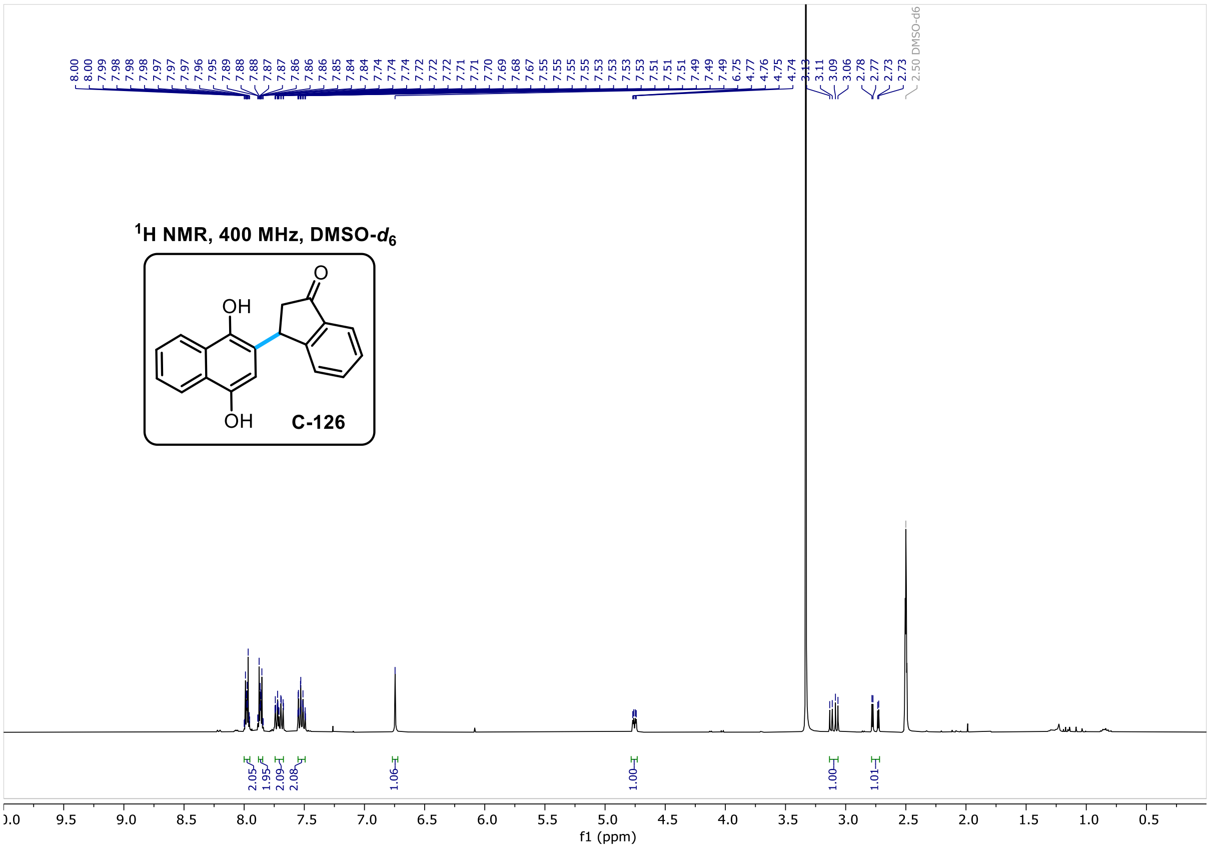
8.00 8.00 7.99 7.98 7.98 7.98 7.97 7.97 7.97 7.96 7.95 7.89 7.88 7.88 7.87 7.87 7.86 7.86 7.86 7.85 7.84 7.84 7.74 7.74 7.74 7.72 7.72 7.72 7.71 7.71 7.70 7.69 7.68 7.67 7.55 7.55 7.55 7.55 7.53 7.53 7.53 7.53 7.51 7.51 7.49 7.49 7.49 6.75 4.77 4.76 4.75 4.74 3.13 3.11 3.09 3.06 2.78 2.77 2.73 2.73
2.50 DMSO-d6
1H NMR, 400 MHz, DMSO-d6
OH
O
OH
C-126
2.05 1.95 2.09 2.08 1.06 1.00 1.00 1.01
f1 (ppm)

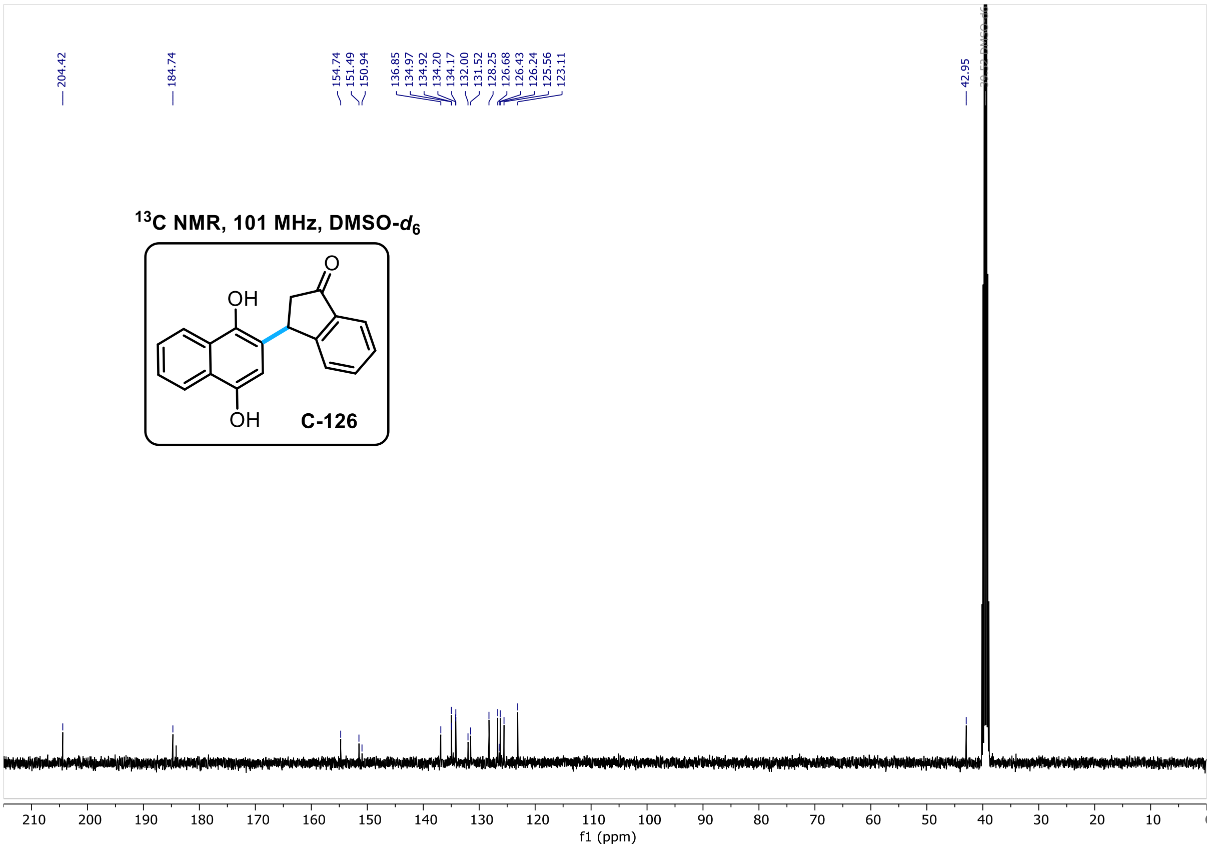
204.42
184.74
154.74 151.49 150.94
136.85 134.97 134.92 134.20 134.17 132.00 131.52 128.25 126.68 126.43 126.24 125.56 123.11
42.95
13C NMR, 101 MHz, DMSO-d6
OH
O
OH
C-126
f1 (ppm)

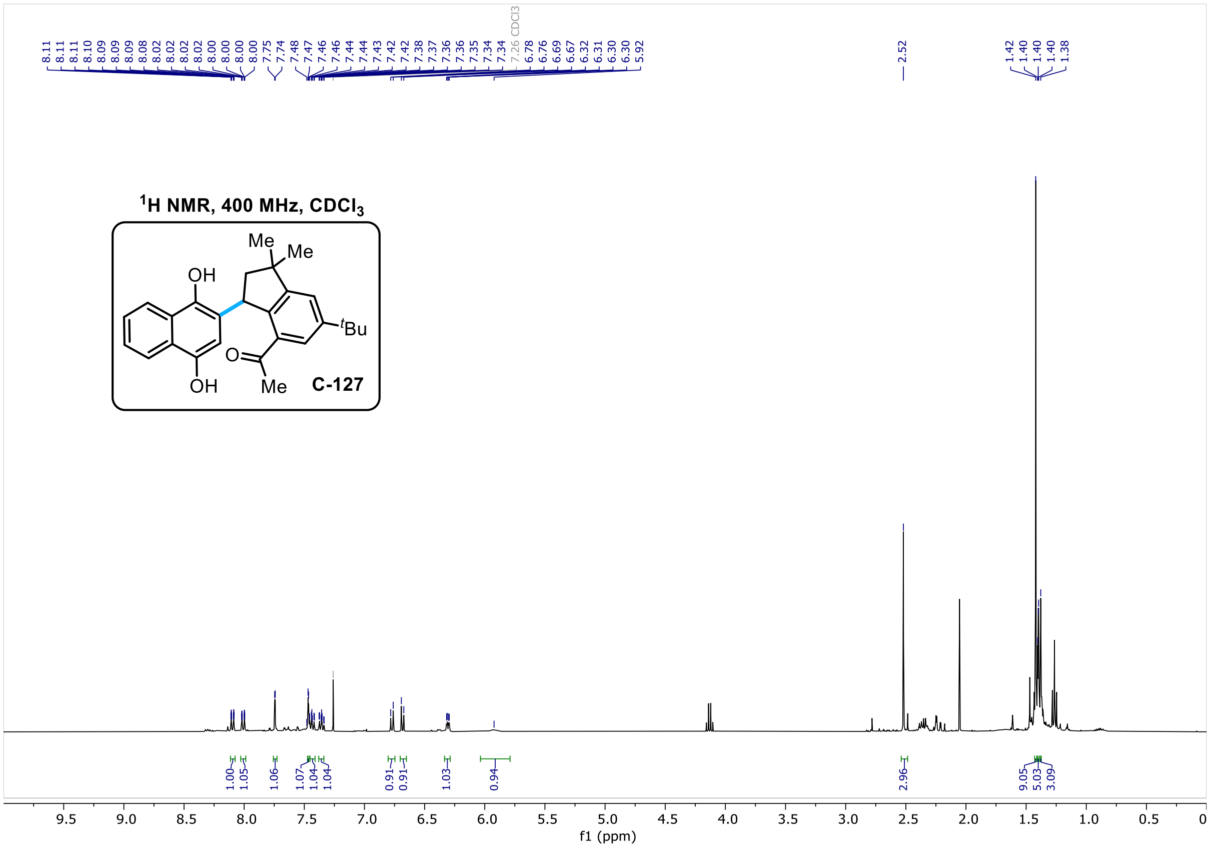
8.11 8.11 8.11 8.10 8.09 8.09 8.09 8.08 8.02 8.02 8.02 8.02 8.00 8.00 8.00 7.75 7.74 7.48 7.47 7.46 7.46 7.44 7.44 7.43 7.42 7.42 7.38 7.37 7.36 7.36 7.35 7.34 7.34 7.26 CDCl3 6.78 6.76 6.69 6.67 6.32 6.31 6.30 6.30 5.92
2.52
1.42 1.40 1.40 1.40 1.38
1H NMR, 400 MHz, CDCl3
Me Me OH tBu O Me OH C-127
1.00 1.05 1.06 1.07 1.04 1.04 0.91 0.91 1.03 0.94 2.96 9.05 5.03 3.09
9.5 9.0 8.5 8.0 7.5 7.0 6.5 6.0 5.5 5.0 4.5 4.0 3.5 3.0 2.5 2.0 1.5 1.0 0.5 0
f1 (ppm)

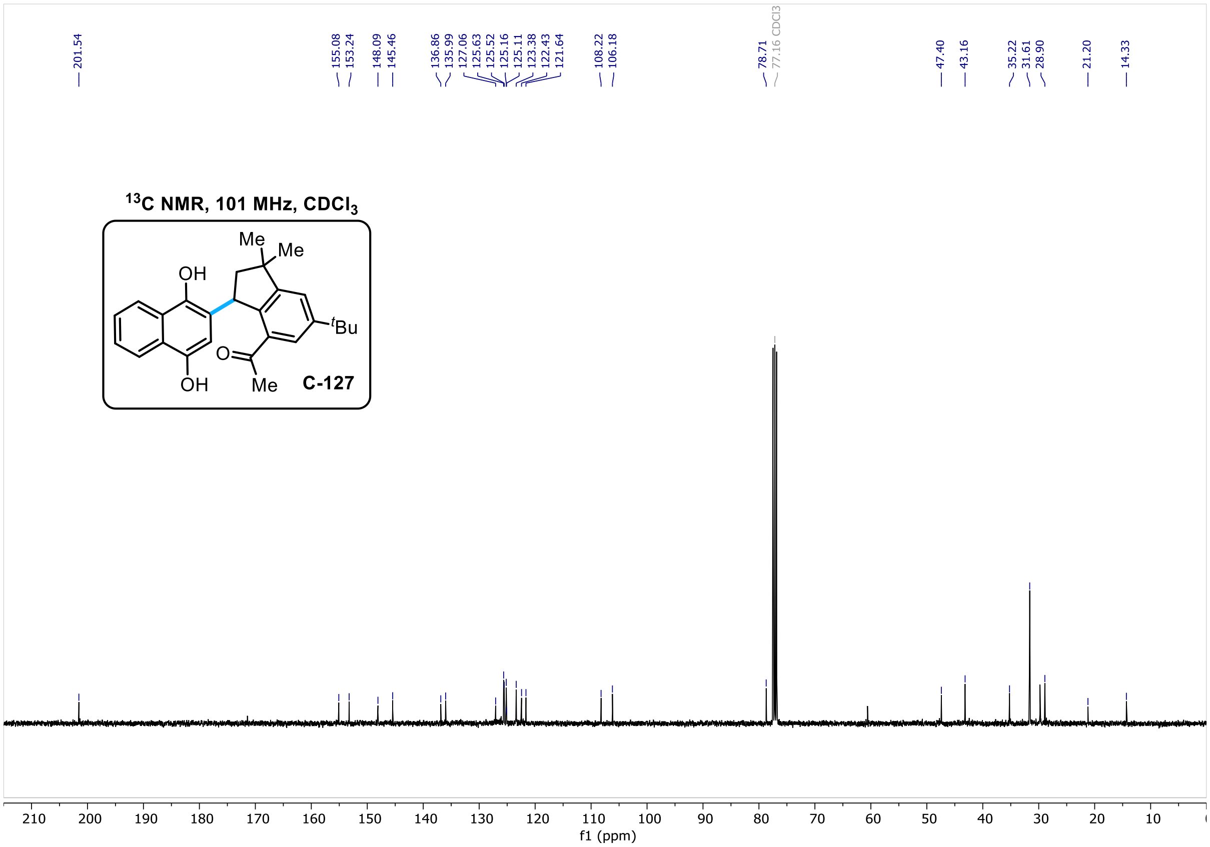
201.54
155.08 153.24 148.09 145.46
136.86 135.99 127.06 125.63 125.52 125.16 125.11 123.38 122.43 121.64
108.22 106.18
78.71 77.16 CDCl3
47.40 43.16 35.22 31.61 28.90 21.20 14.33
13C NMR, 101 MHz, CDCl3
Me Me OH tBu O Me OH C-127
210 200 190 180 170 160 150 140 130 120 110 100 90 80 70 60 50 40 30 20 10
f1 (ppm)

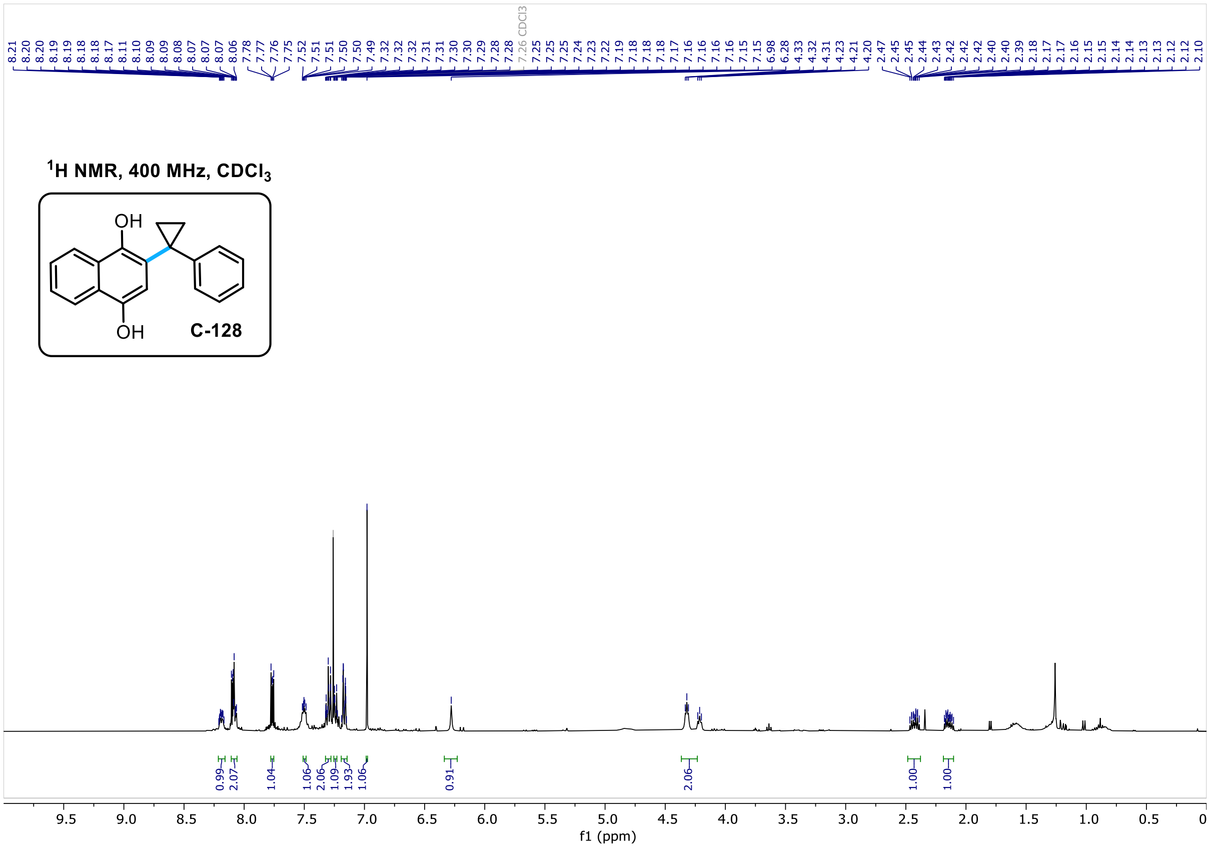

^{1}H NMR, 400 MHz, CDCl$_3$
C-128
f1 (ppm)

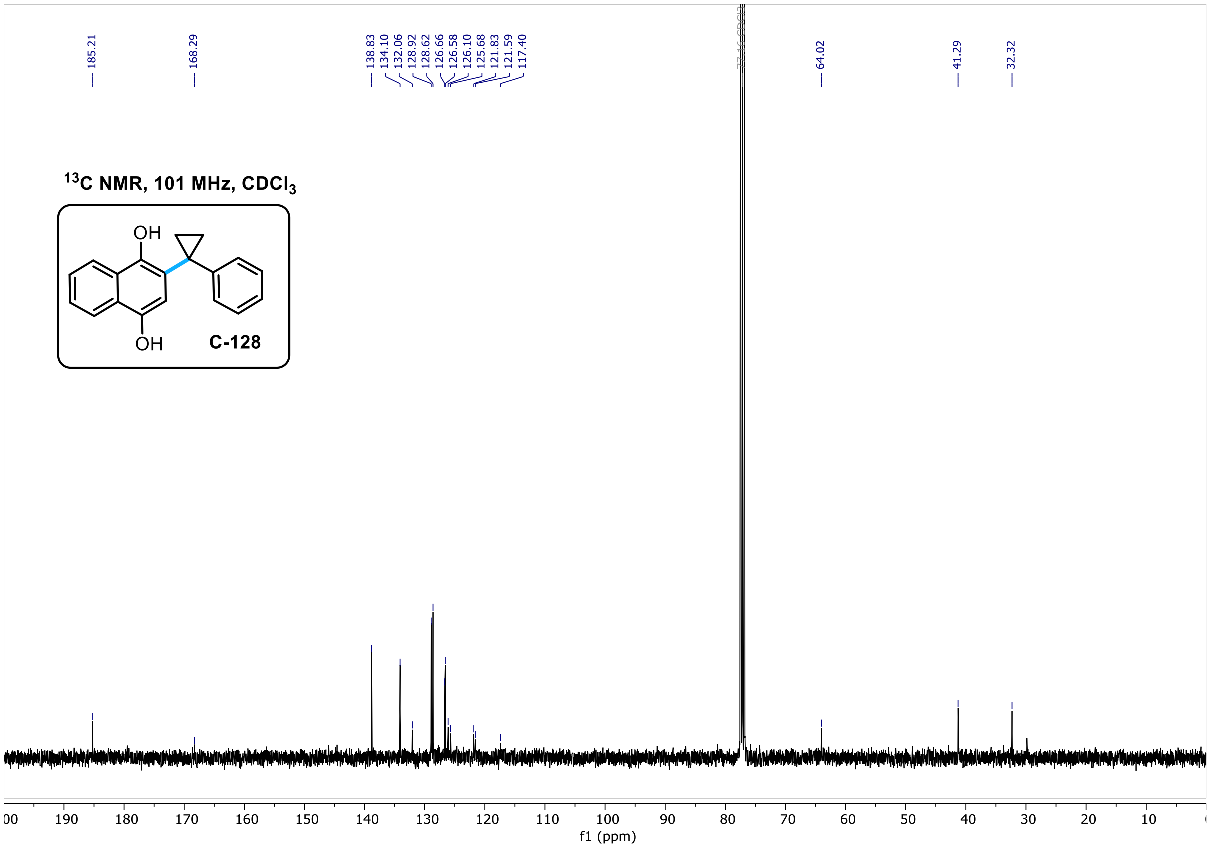

^{13}C NMR, 101 MHz, CDCl$_3$
C-128
185.21
168.29
138.83
134.10
132.06
128.92
128.62
126.66
126.58
126.10
125.68
121.83
121.59
117.40
64.02
41.29
32.32
f1 (ppm)

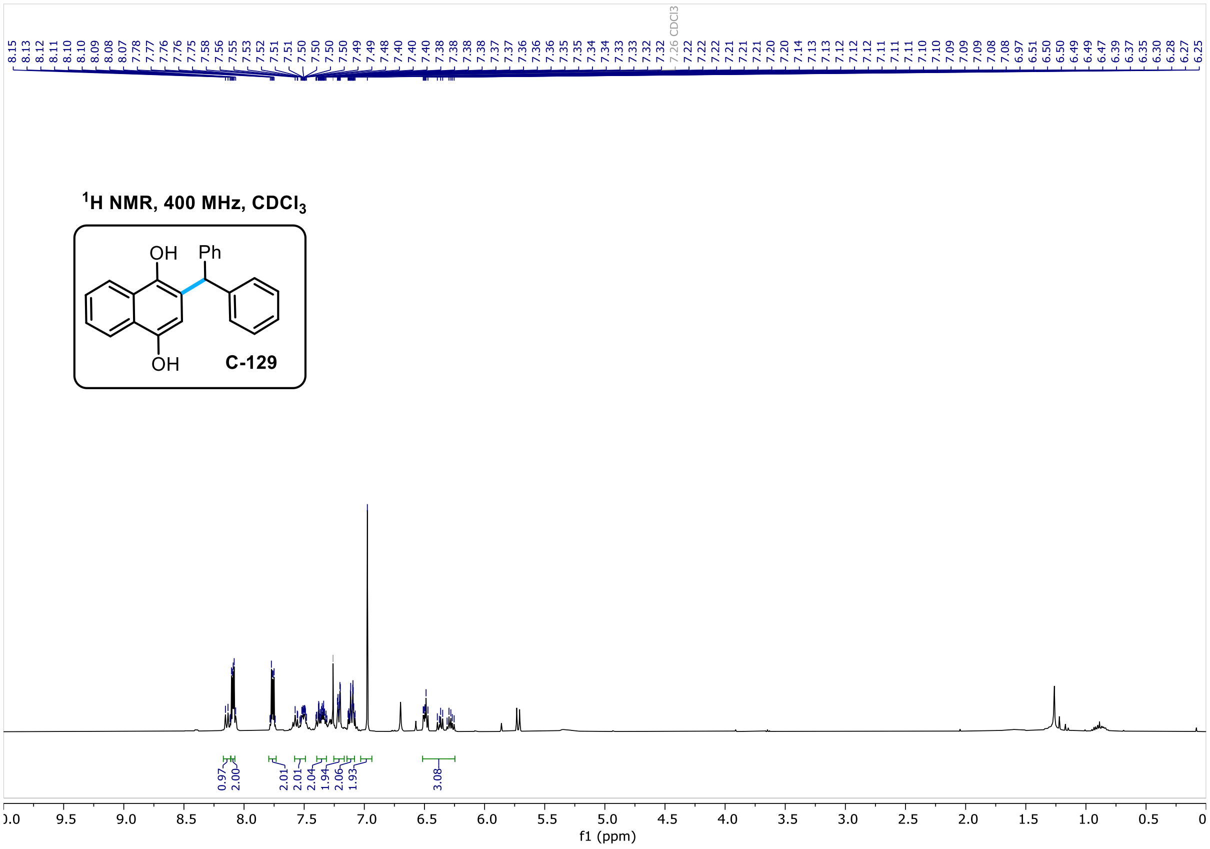
1H NMR, 400 MHz, CDCl3
OH
Ph
OH
C-129
f1 (ppm)

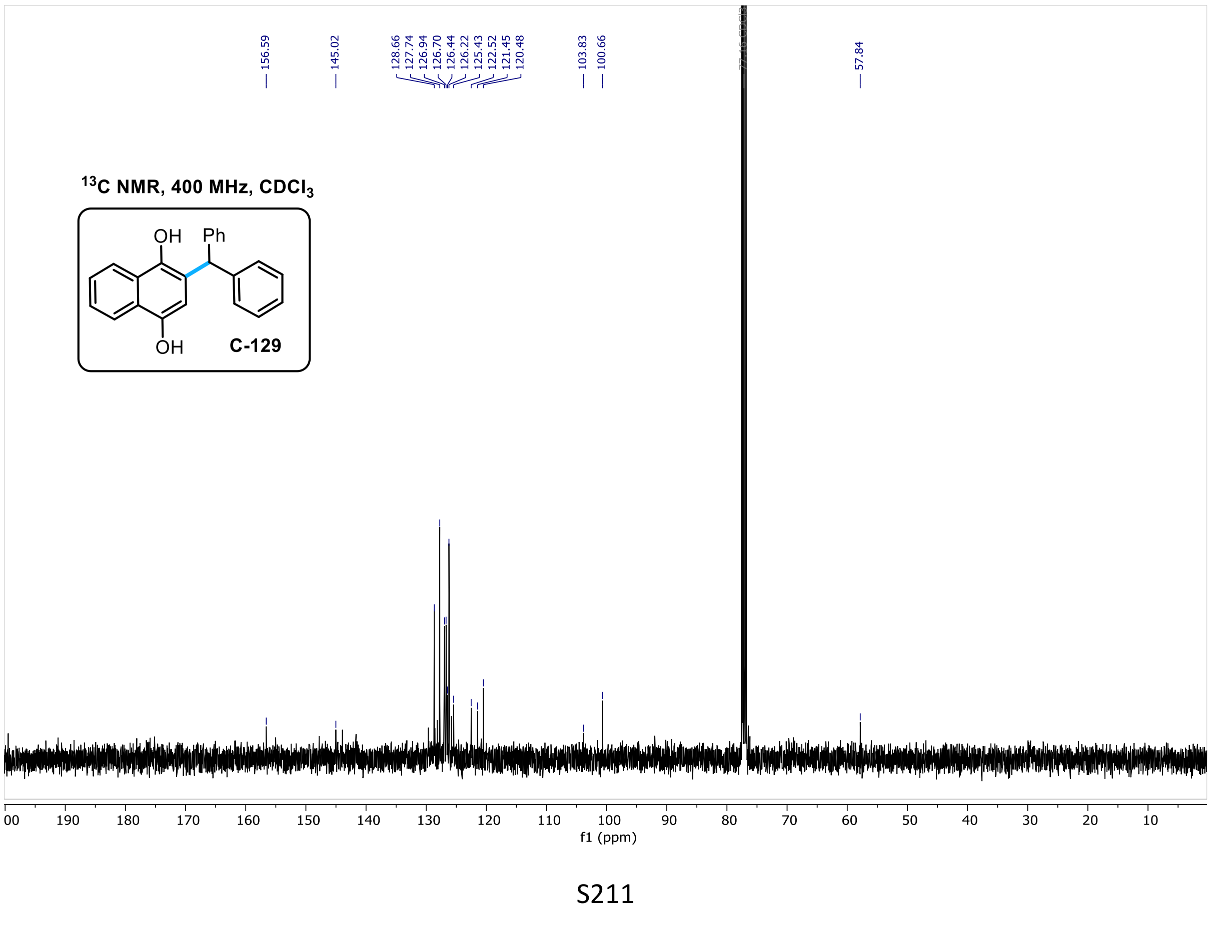
156.59
145.02
128.66
127.74
126.94
126.70
126.44
126.22
125.43
122.52
121.45
120.48
103.83
100.66
57.84
13C NMR, 400 MHz, CDCl3
OH
Ph
OH
C-129
f1 (ppm)

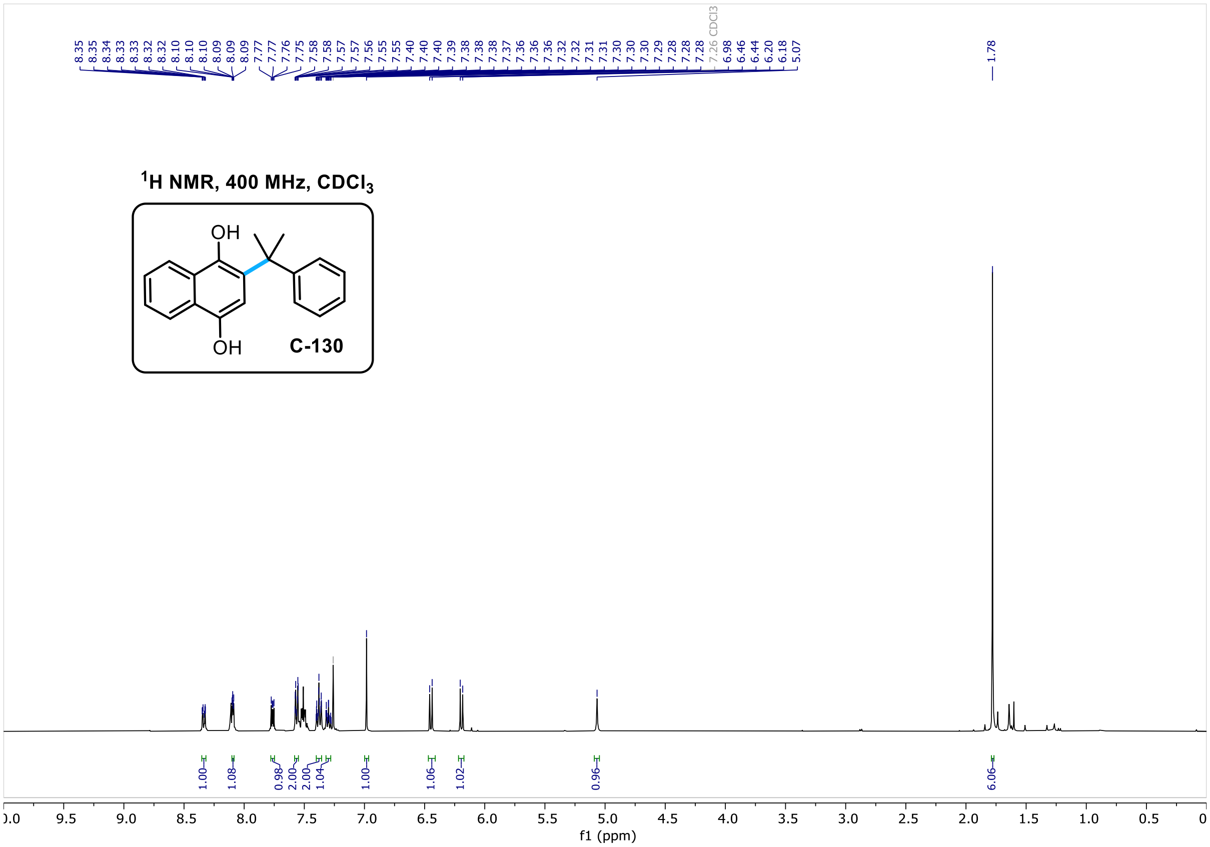

8.35 8.35 8.34 8.33 8.33 8.32 8.32 8.10 8.10 8.09 8.09 7.77 7.77 7.76 7.75 7.58 7.58 7.57 7.57 7.56 7.55 7.55 7.40 7.40 7.39 7.38 7.38 7.38 7.37 7.36 7.36 7.36 7.32 7.32 7.31 7.31 7.30 7.30 7.30 7.29 7.28 7.28 7.28
7.26 CDCl3
6.98 6.46 6.44 6.20 6.18 5.07
1.78
^{1}H NMR, 400 MHz, CDCl$_3$
OH
OH
C-130
1.00 1.08 0.98 2.00 2.00 1.04 1.00 1.06 1.02 0.96 6.06
f1 (ppm)

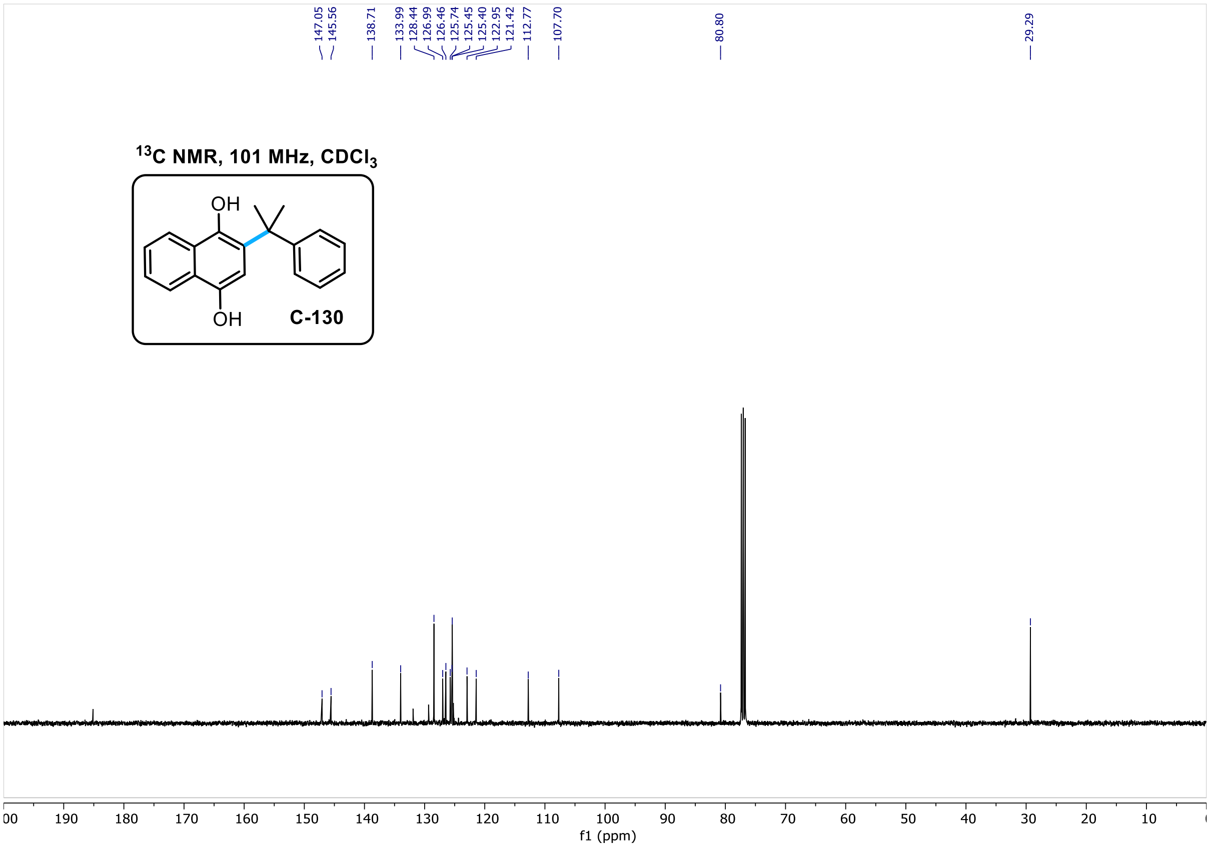

147.05 145.56 138.71 133.99 128.44 126.99 126.46 125.74 125.45 125.40 122.95 121.42 112.77 107.70 80.80 29.29
^{13}C NMR, 101 MHz, CDCl$_3$
OH
OH
C-130
f1 (ppm)

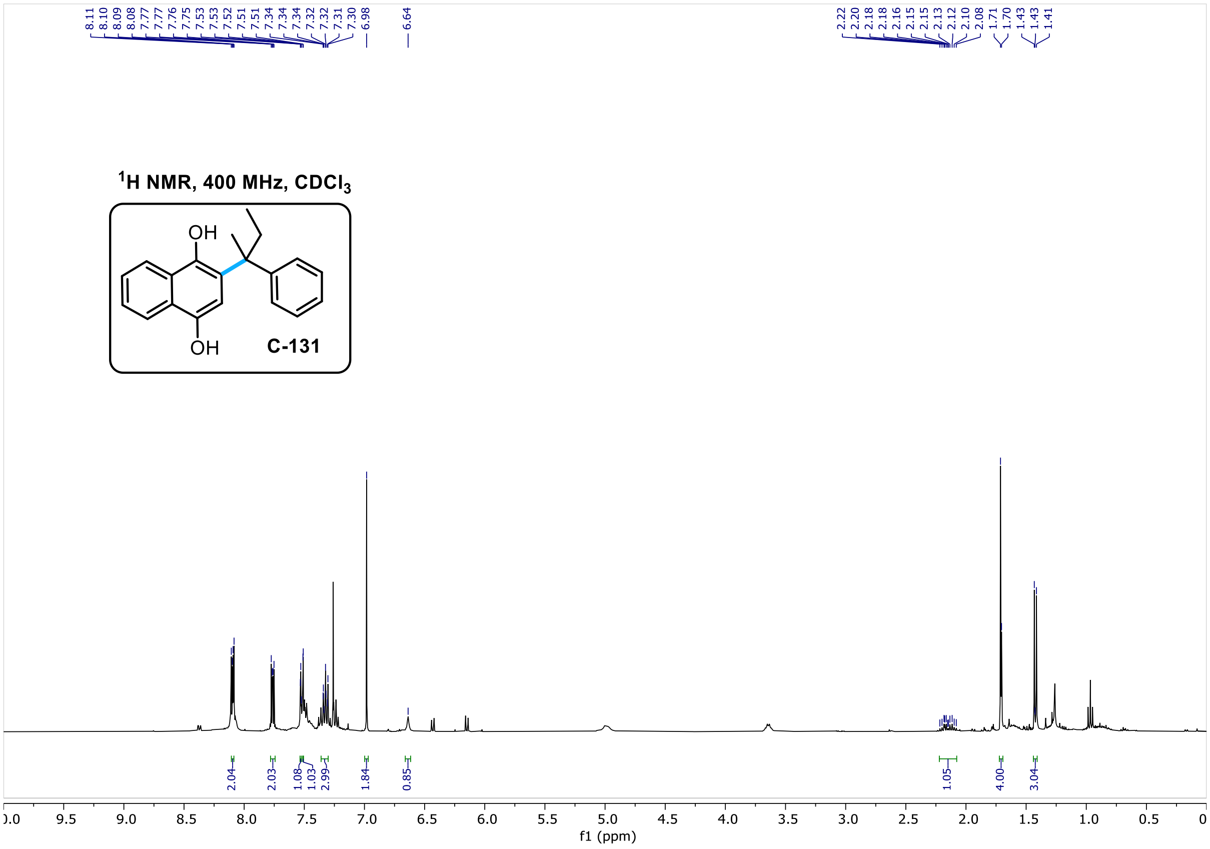
8.11 8.10 8.09 8.08 7.77 7.77 7.76 7.75 7.53 7.53 7.52 7.51 7.51 7.34 7.34 7.34 7.32 7.32 7.31 7.30 6.98 6.64
2.22 2.20 2.18 2.18 2.16 2.15 2.15 2.13 2.12 2.10 2.08 1.71 1.70 1.43 1.43 1.41
^{1}H NMR, 400 MHz, CDCl$_3$
OH
OH
C-131
2.04 2.03 1.08 1.03 2.99 1.84 0.85 1.05 4.00 3.04
f1 (ppm)

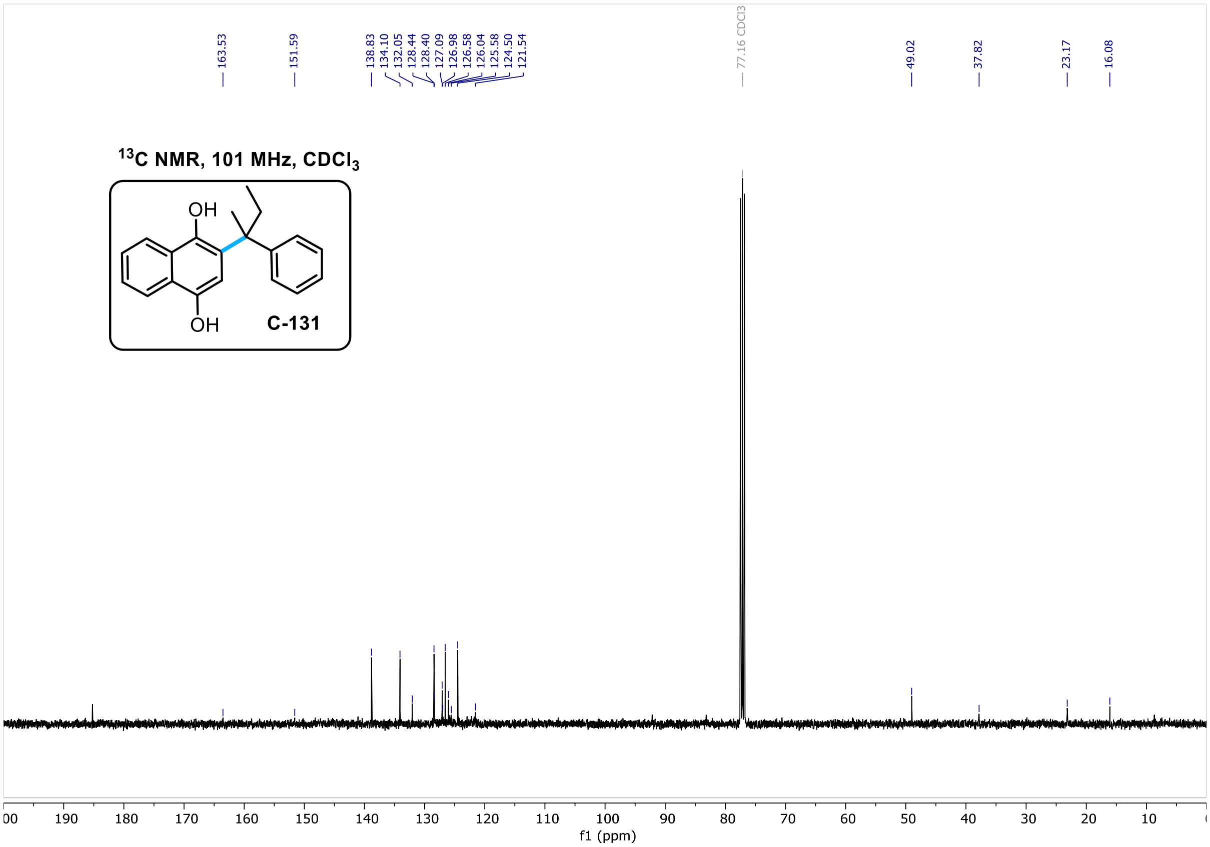
163.53
151.59
138.83 134.10 132.05 128.44 128.40 127.09 126.98 126.58 126.04 125.58 124.50 121.54
77.16 CDCl3
49.02
37.82
23.17
16.08
^{13}C NMR, 101 MHz, CDCl$_3$
OH
OH
C-131
f1 (ppm)

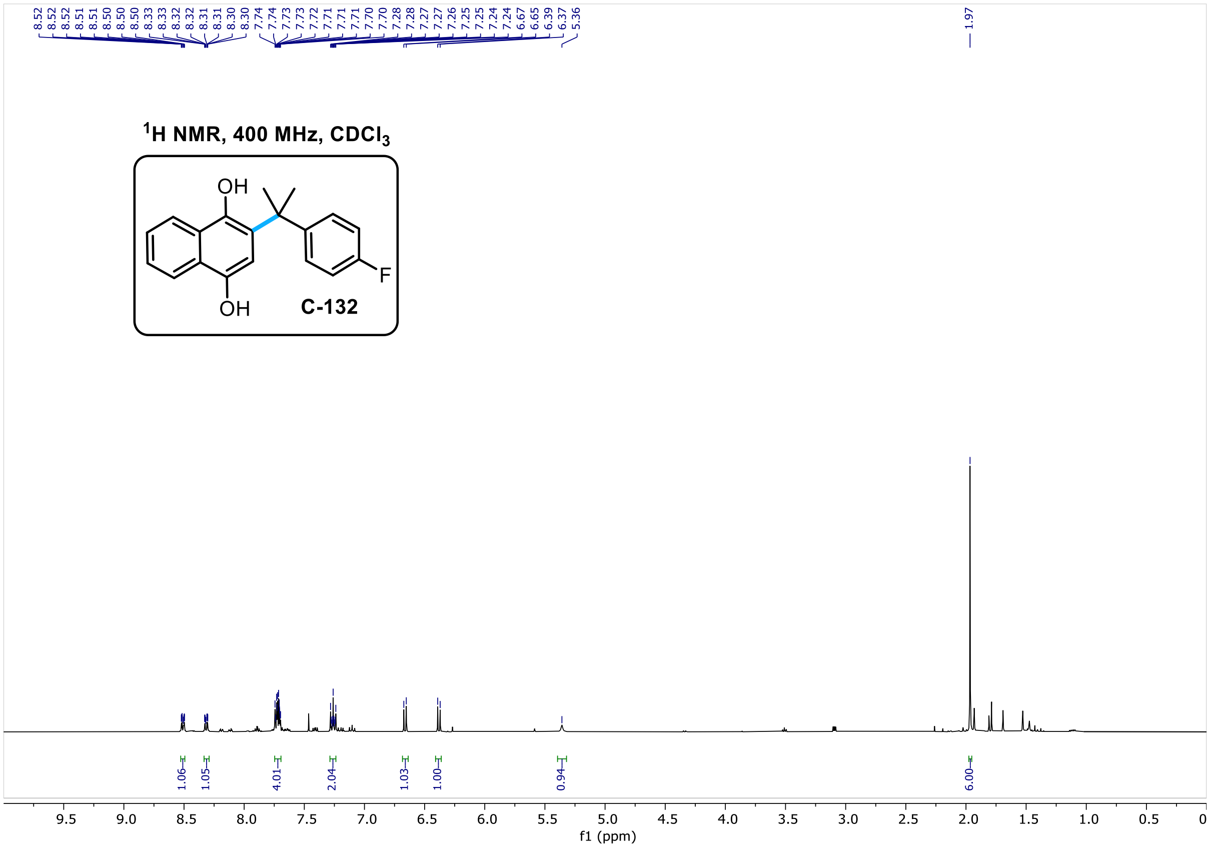
8.52 8.52 8.52 8.51 8.51 8.50 8.50 8.50 8.33 8.33 8.32 8.32 8.32 8.31 8.31 8.30 8.30 7.74 7.74 7.73 7.73 7.72 7.71 7.71 7.71 7.70 7.70 7.28 7.28 7.27 7.27 7.26 7.25 7.25 7.24 7.24 6.67 6.65 6.39 6.37 5.36
1.97
^{1}H NMR, 400 MHz, CDCl$_3$
OH
OH
F
C-132
1.06 1.05 4.01 2.04 1.03 1.00 0.94 6.00
9.5 9.0 8.5 8.0 7.5 7.0 6.5 6.0 5.5 5.0 4.5 4.0 3.5 3.0 2.5 2.0 1.5 1.0 0.5 0
f1 (ppm)

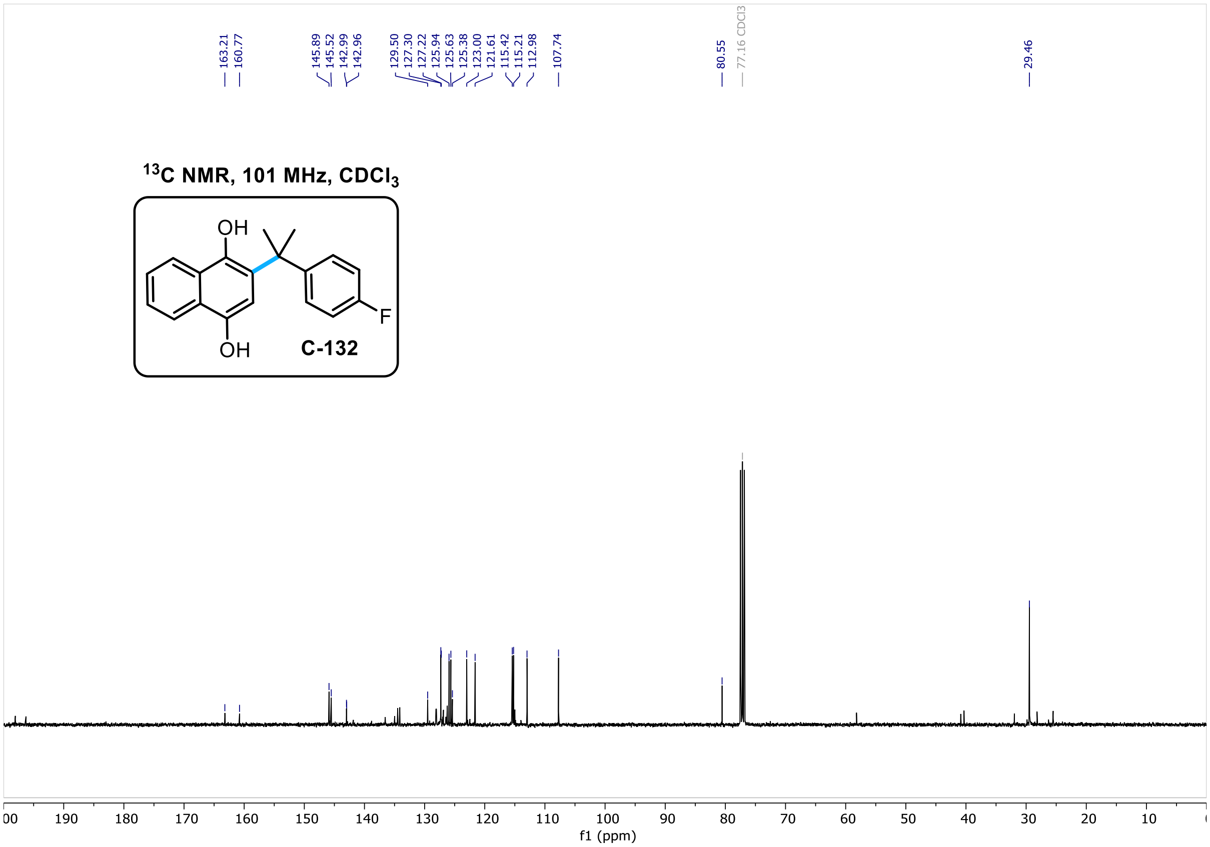
163.21 160.77
145.89 145.52 142.99 142.96
129.50 127.30 127.22 125.94 125.63 125.38 123.00 121.61 115.42 115.21 112.98 107.74
80.55
77.16 CDCl3
29.46
^{13}C NMR, 101 MHz, CDCl$_3$
OH
OH
F
C-132
190 180 170 160 150 140 130 120 110 100 90 80 70 60 50 40 30 20 10
f1 (ppm)

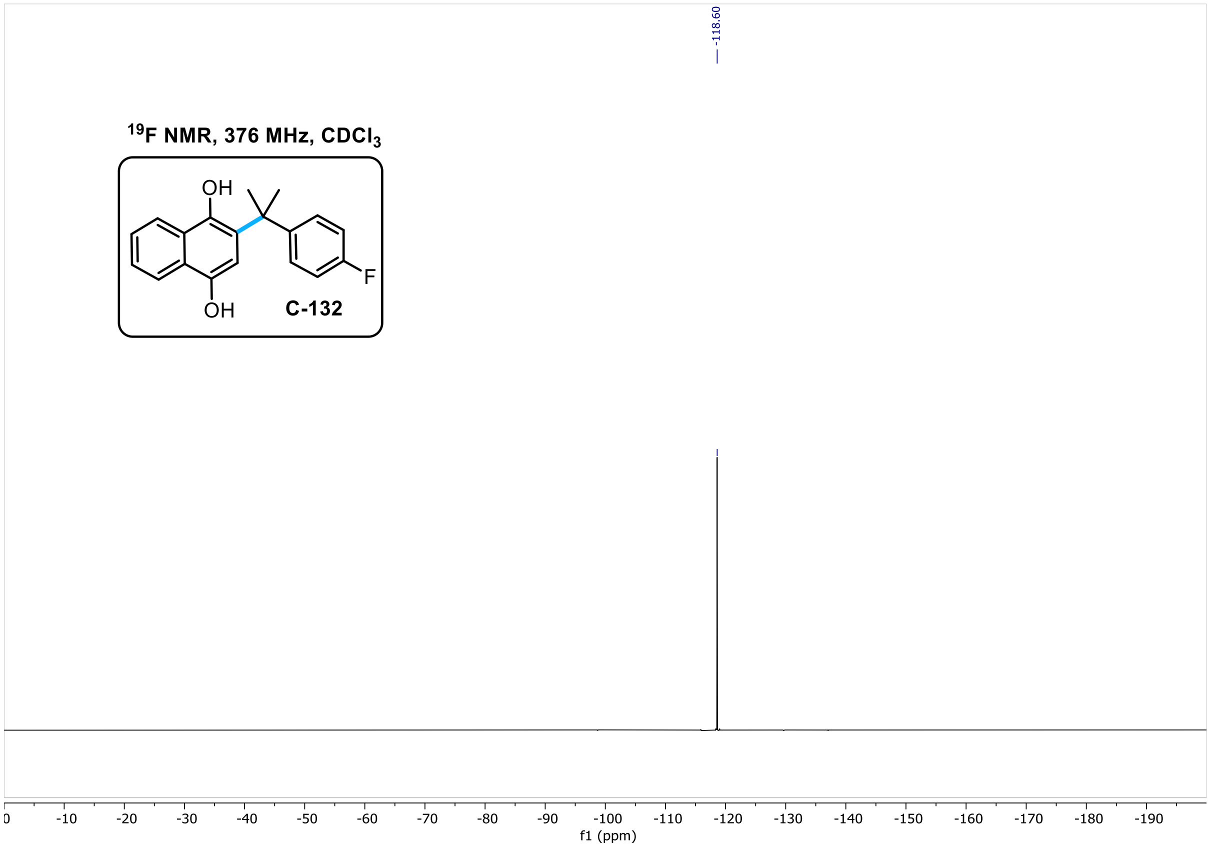
-118.60
19F NMR, 376 MHz, CDCl3
OH
OH
F
C-132
0
-10
-20
-30
-40
-50
-60
-70
-80
-90
-100
-110
-120
-130
-140
-150
-160
-170
-180
-190
f1 (ppm)

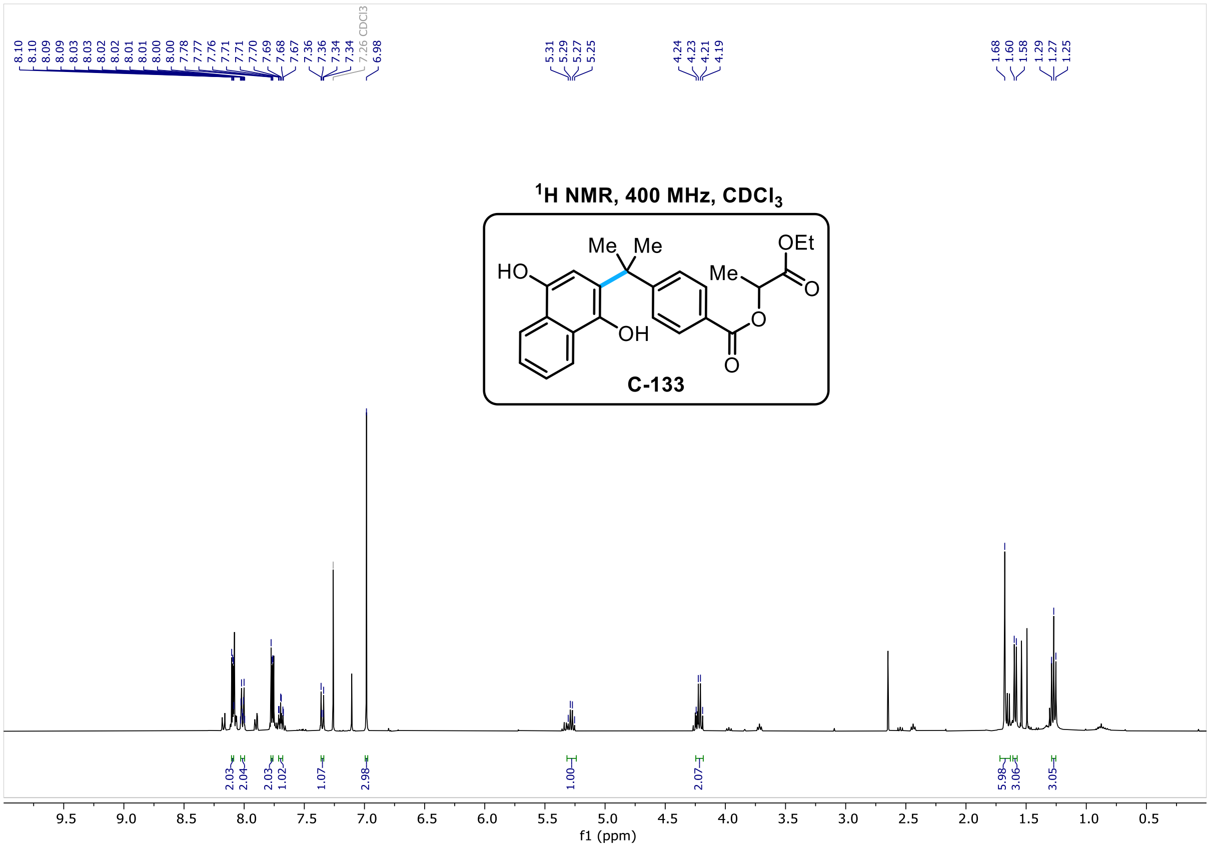

8.10 8.10 8.09 8.09 8.03 8.03 8.02 8.02 8.01 8.01 8.00 8.00 7.78 7.77 7.76 7.71 7.71 7.70 7.69 7.68 7.67 7.36 7.36 7.34 7.34 7.26 CDCl3 6.98
5.31 5.29 5.27 5.25
4.24 4.23 4.21 4.19
1.68 1.60 1.58 1.29 1.27 1.25
^{1}H NMR, 400 MHz, $CDCl_3$
Me Me
HO
OEt
Me
O
O
OH
O
C-133
2.03 2.04 2.03 1.02 1.07 2.98 1.00 2.07 5.98 3.06 3.05
9.5 9.0 8.5 8.0 7.5 7.0 6.5 6.0 5.5 5.0 4.5 4.0 3.5 3.0 2.5 2.0 1.5 1.0 0.5
f1 (ppm)

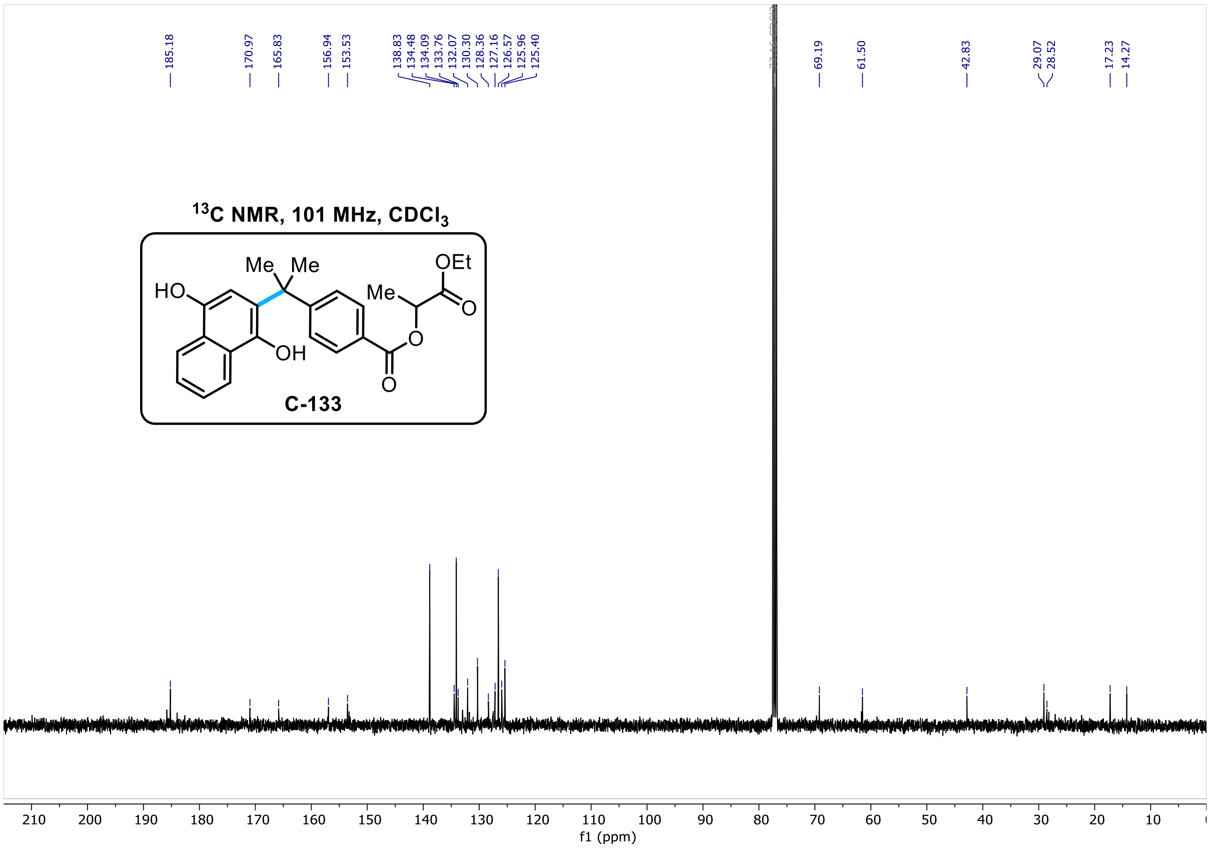

185.18
170.97
165.83
156.94
153.53
138.83 134.48 134.09 133.76 132.07 130.30 128.36 127.16 126.57 125.96 125.40
69.19
61.50
42.83
29.07 28.52
17.23
14.27
^{13}C NMR, 101 MHz, $CDCl_3$
Me Me
HO
OEt
Me
O
O
OH
O
C-133
210 200 190 180 170 160 150 140 130 120 110 100 90 80 70 60 50 40 30 20 10
f1 (ppm)

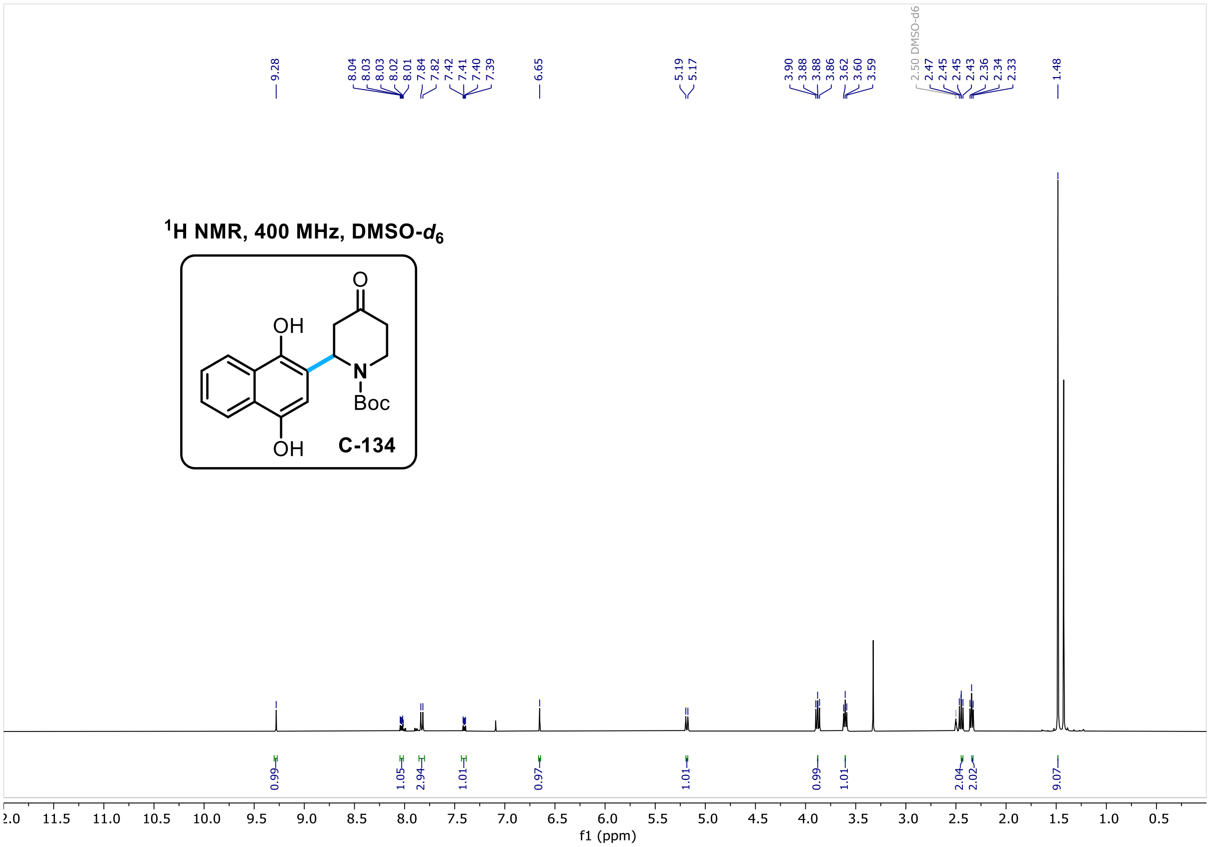
9.28
8.04
8.03
8.03
8.02
8.01
7.84
7.82
7.42
7.41
7.40
7.39
6.65
5.19
5.17
3.90
3.88
3.88
3.86
3.62
3.60
3.59
2.50 DMSO-d6
2.47
2.45
2.45
2.43
2.36
2.34
2.33
1.48
1H NMR, 400 MHz, DMSO-d6
OH
O
N
Boc
OH
C-134
0.99
1.05
2.94
1.01
0.97
1.01
0.99
1.01
2.04
2.02
9.07
f1 (ppm)

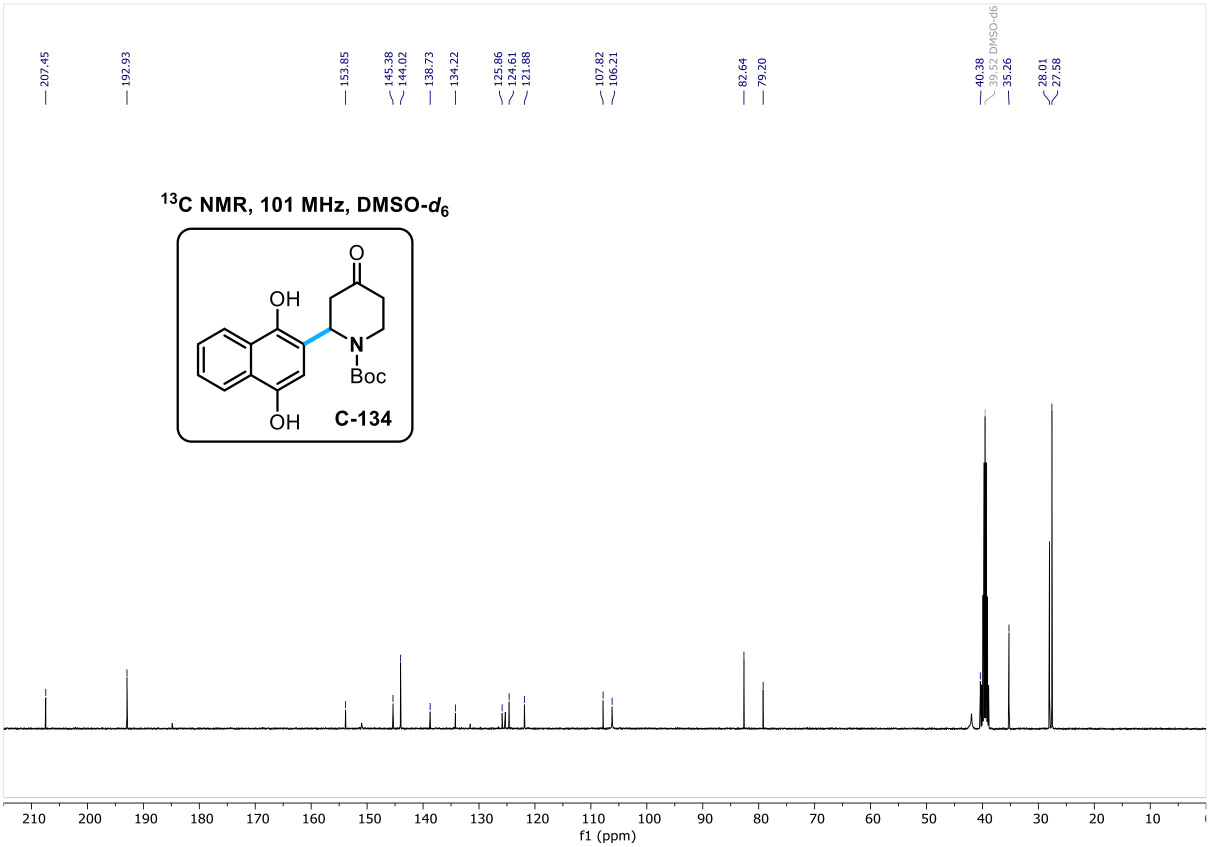
207.45
192.93
153.85
145.38
144.02
138.73
134.22
125.86
124.61
121.88
107.82
106.21
82.64
79.20
40.38
39.52 DMSO-d6
35.26
28.01
27.58
13C NMR, 101 MHz, DMSO-d6
OH
O
N
Boc
OH
C-134
f1 (ppm)

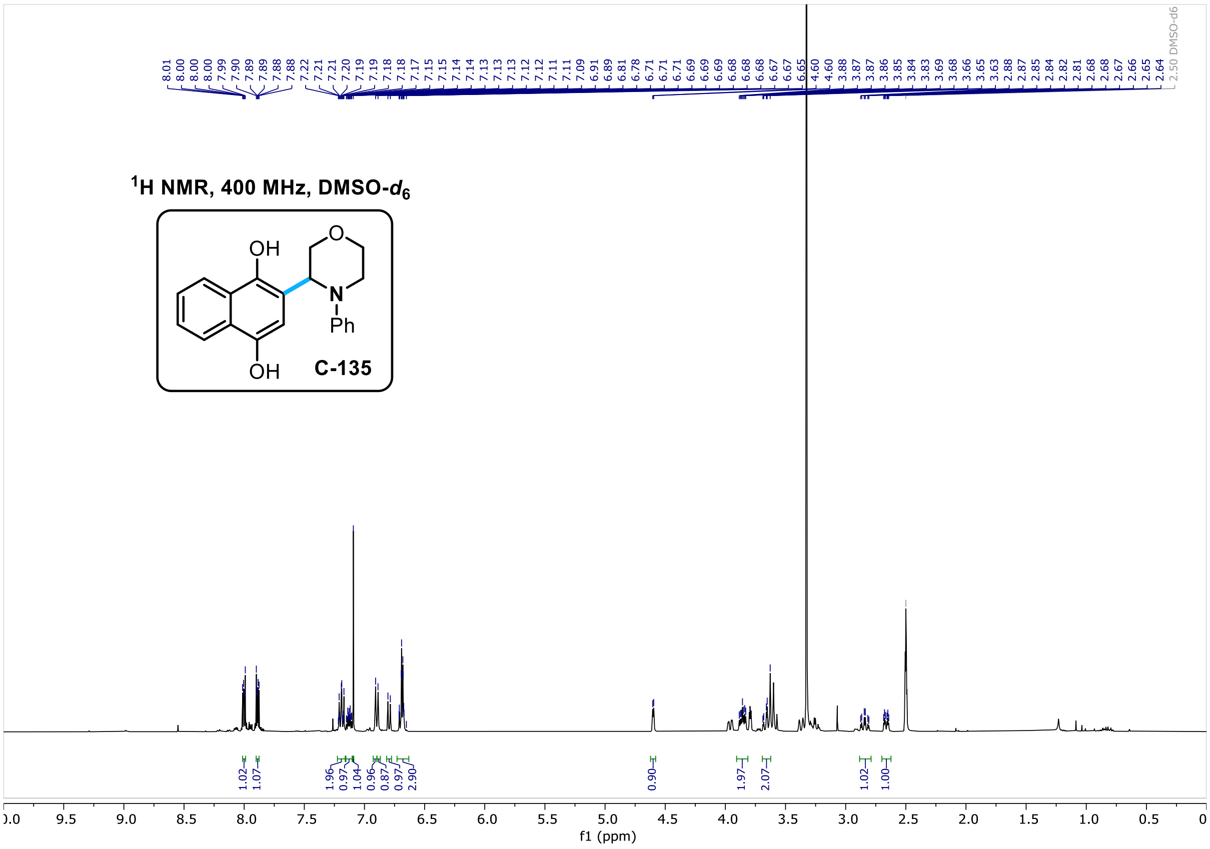

8.01 8.00 8.00 7.99 7.90 7.89 7.89 7.88 7.88 7.22 7.21 7.21 7.20 7.19 7.19 7.18 7.18 7.17 7.15 7.15 7.14 7.14 7.13 7.13 7.12 7.12 7.11 7.11 7.09 6.91 6.89 6.81 6.78 6.71 6.71 6.69 6.69 6.68 6.68 6.68 6.67 6.65 4.60 3.88 3.87 3.87 3.86 3.85 3.84 3.83 3.69 3.68 3.66 3.65 3.63 2.88 2.87 2.85 2.84 2.82 2.81 2.68 2.68 2.67 2.66 2.65 2.64
2.50 DMSO-d6
^{1}H NMR, 400 MHz, DMSO-d_6
OH
O
N
Ph
OH
C-135
1.02 1.07 1.96 0.97 1.04 0.96 0.87 0.97 2.90 0.90 1.97 2.07 1.02 1.00
f1 (ppm)

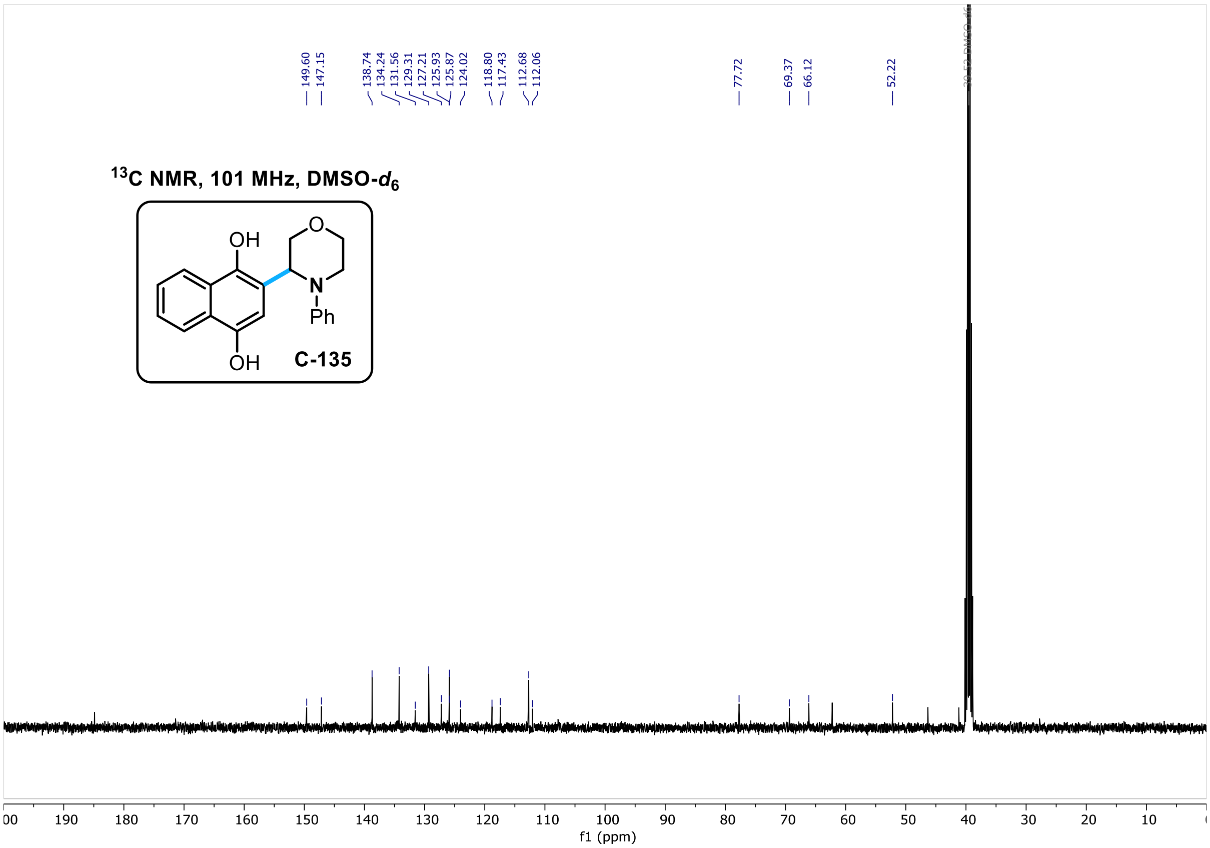

149.60 147.15 138.74 134.24 131.56 129.31 127.21 125.93 125.87 124.02 118.80 117.43 112.68 112.06 77.72 69.37 66.12 52.22
^{13}C NMR, 101 MHz, DMSO-d_6
OH
O
N
Ph
OH
C-135
f1 (ppm)

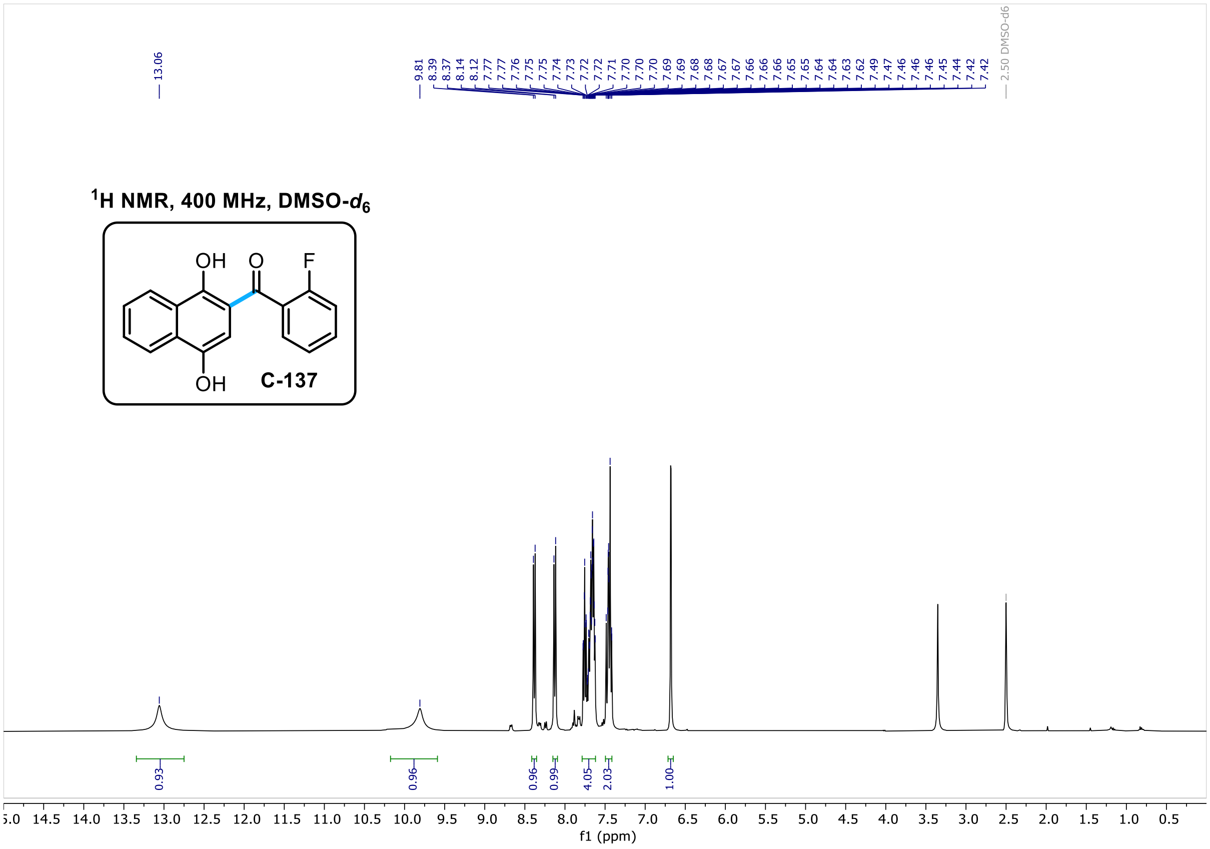
13.06
9.81 8.39 8.37 8.14 8.12 7.77 7.77 7.76 7.75 7.75 7.74 7.73 7.72 7.72 7.71 7.70 7.70 7.69 7.69 7.68 7.68 7.67 7.67 7.66 7.66 7.66 7.65 7.65 7.64 7.64 7.63 7.62 7.49 7.47 7.46 7.46 7.45 7.44 7.42 7.42
2.50 DMSO-d6
^{1}H NMR, 400 MHz, DMSO-d_6
OH
O
F
OH
C-137
0.93
0.96
0.96
0.99
4.05
2.03
1.00
f1 (ppm)

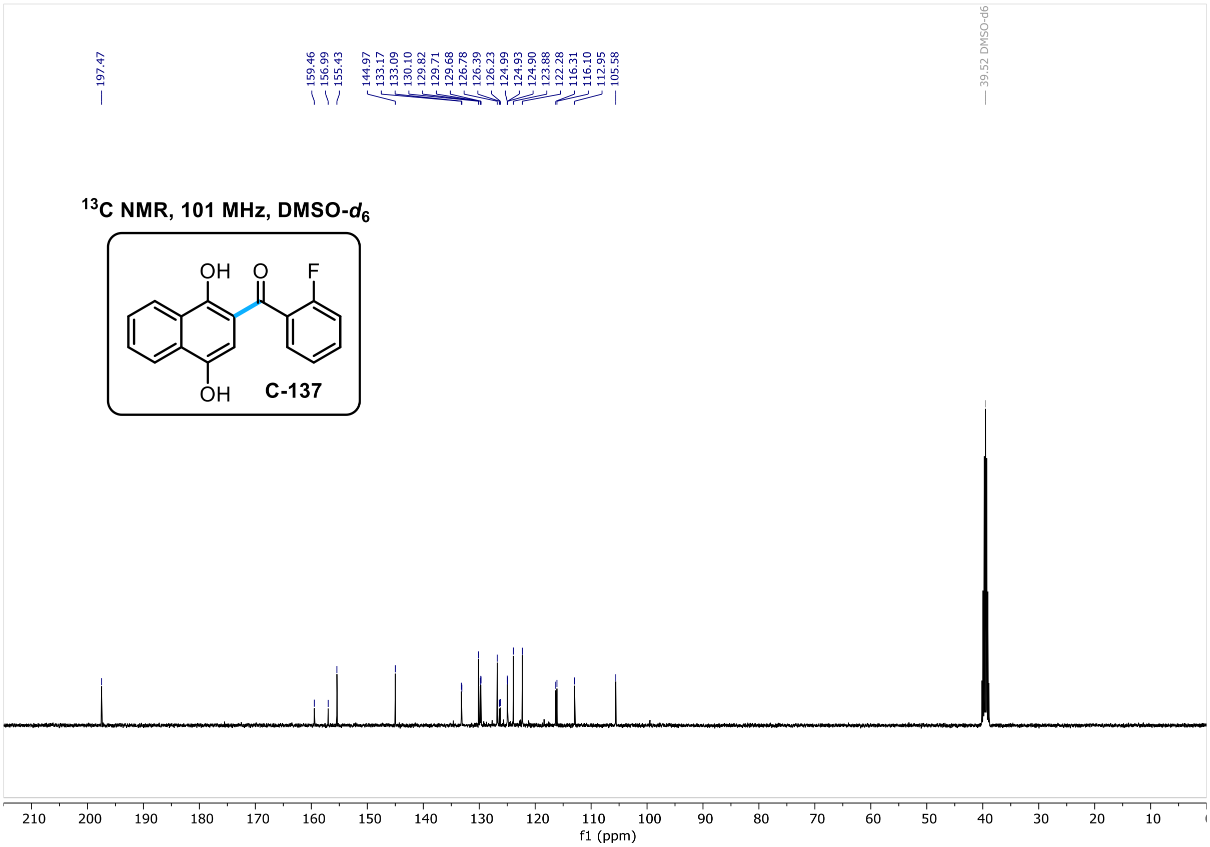
197.47
159.46 156.99 155.43
144.97 133.17 133.09 130.10 129.82 129.71 129.68 126.78 126.39 126.23 124.99 124.93 124.90 123.88 122.28 116.31 116.10 112.95 105.58
39.52 DMSO-d6
^{13}C NMR, 101 MHz, DMSO-d_6
OH
O
F
OH
C-137
f1 (ppm)

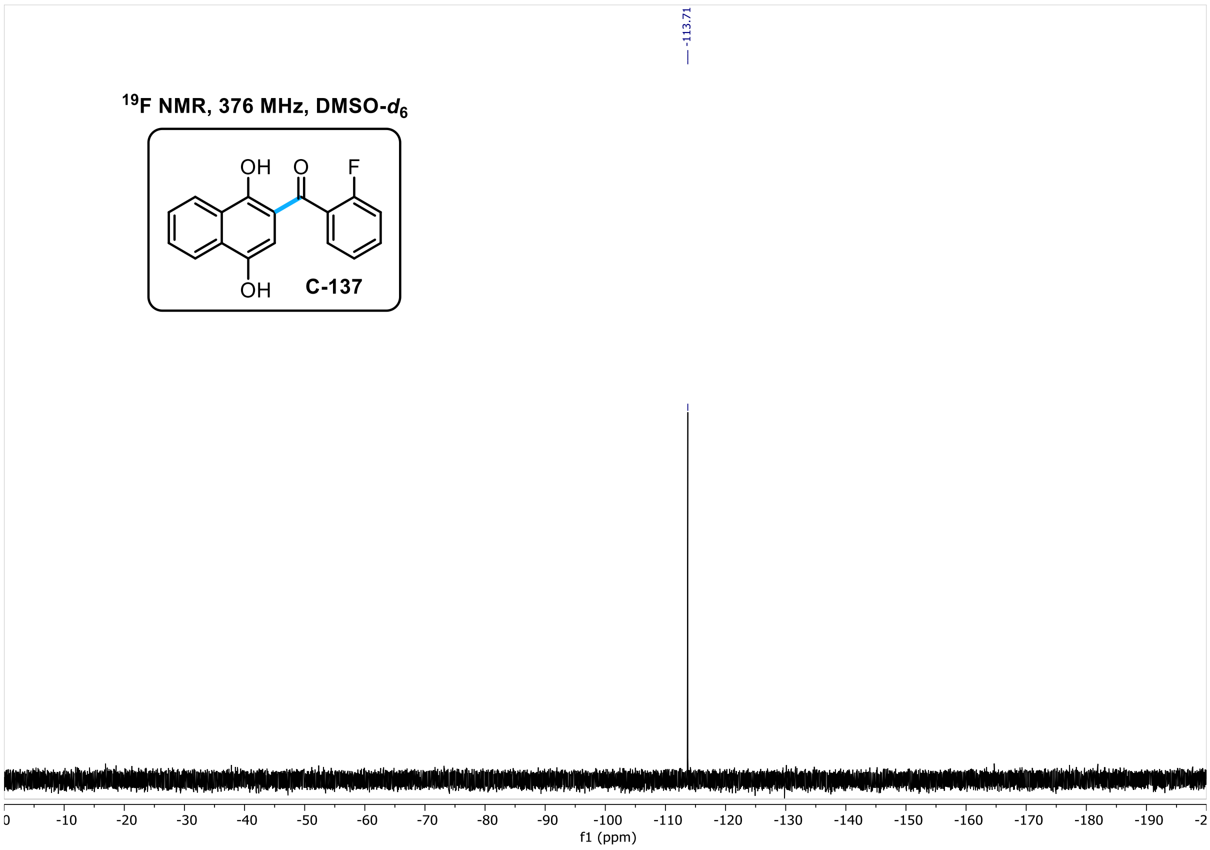
-113.71
19F NMR, 376 MHz, DMSO-d6
OH
O
F
OH
C-137
0
-10
-20
-30
-40
-50
-60
-70
-80
-90
-100
-110
-120
-130
-140
-150
-160
-170
-180
-190
-2
f1 (ppm)

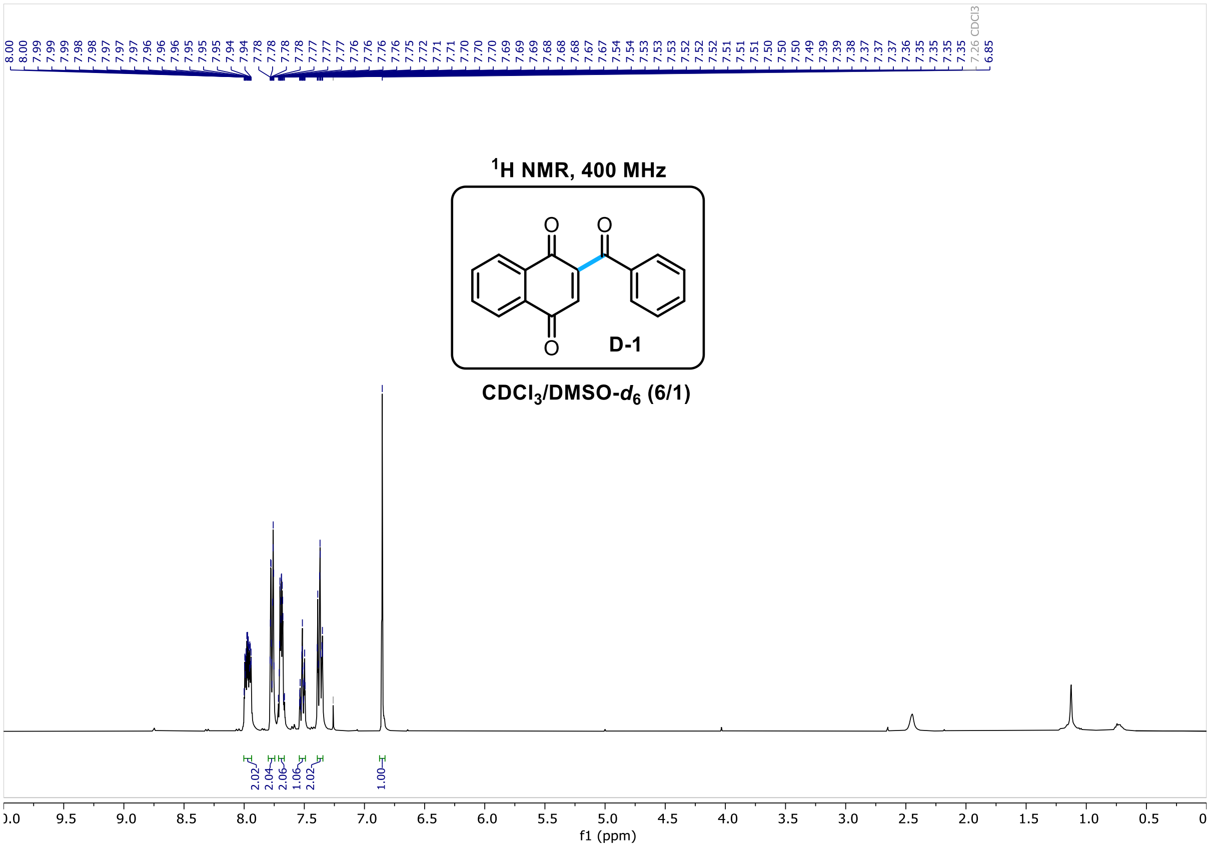

8.00
8.00
7.99
7.99
7.98
7.98
7.97
7.97
7.97
7.96
7.96
7.96
7.95
7.95
7.95
7.94
7.94
7.78
7.78
7.78
7.78
7.77
7.77
7.76
7.76
7.76
7.75
7.72
7.71
7.71
7.70
7.70
7.70
7.69
7.69
7.69
7.68
7.68
7.68
7.67
7.54
7.54
7.53
7.53
7.53
7.52
7.52
7.52
7.51
7.51
7.51
7.50
7.50
7.50
7.49
7.39
7.39
7.38
7.37
7.37
7.37
7.36
7.35
7.35
7.35
7.35
7.26 CDCl3
6.85
¹H NMR, 400 MHz
D-1
CDCl3/DMSO-d6 (6/1)
2.02
2.04
2.06
1.06
2.02
1.00
f1 (ppm)

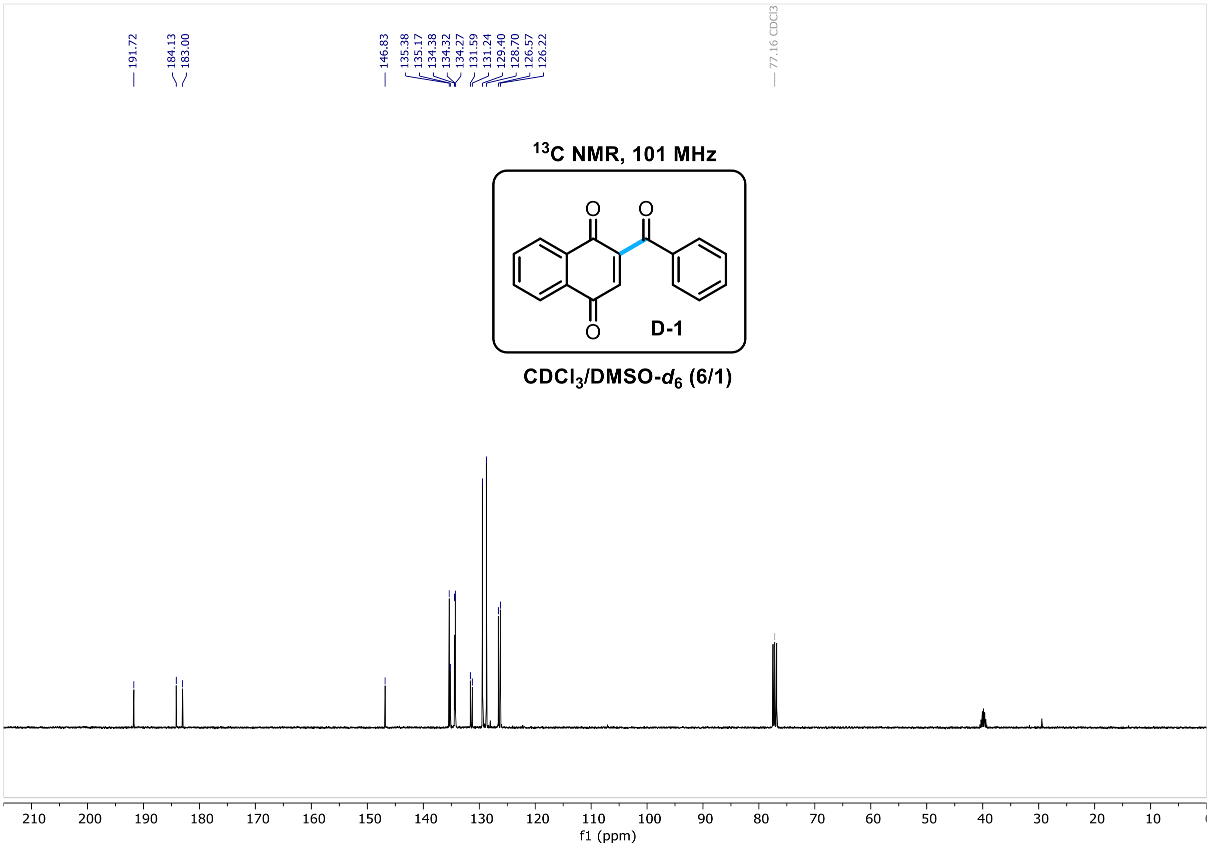

191.72
184.13
183.00
146.83
135.38
135.17
134.38
134.32
134.27
131.59
131.24
129.40
128.70
126.57
126.22
77.16 CDCl3
13C NMR, 101 MHz
D-1
CDCl3/DMSO-d6 (6/1)
f1 (ppm)

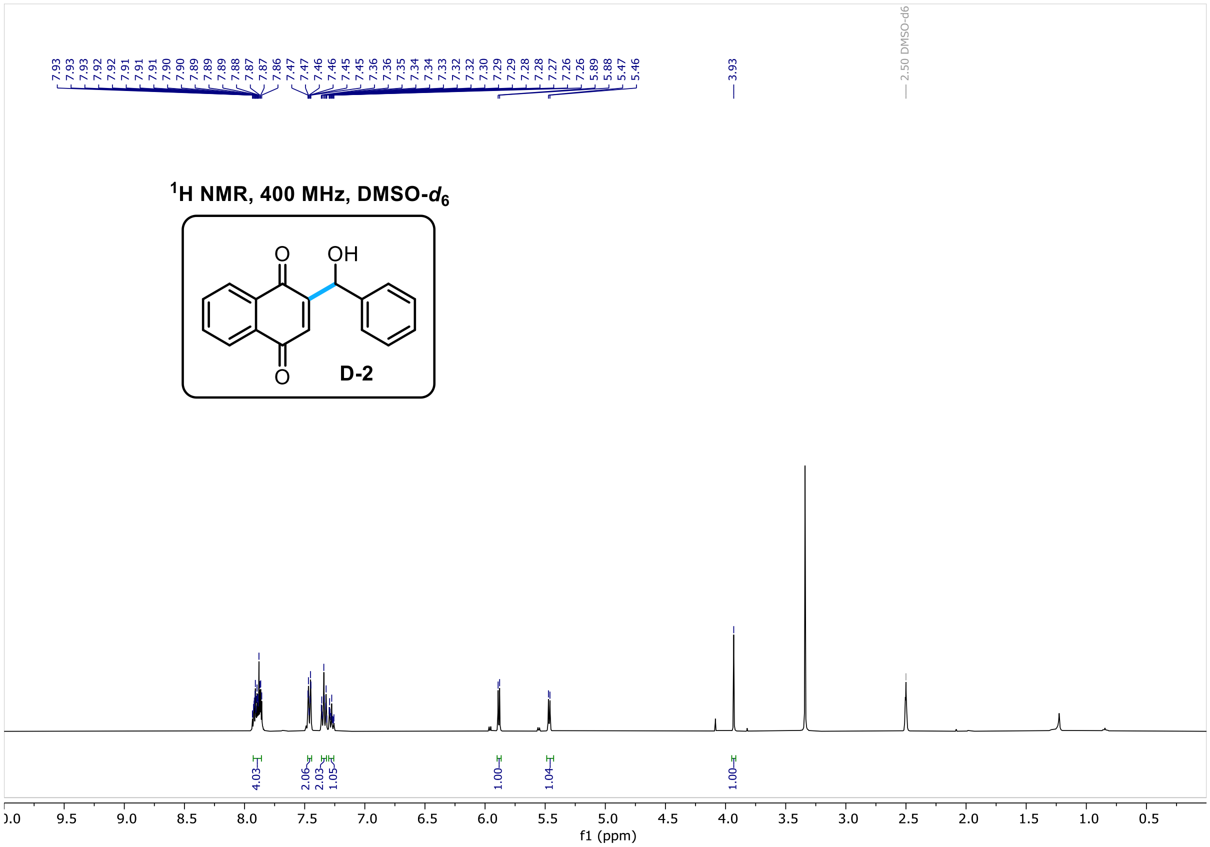
7.93
7.93
7.92
7.92
7.91
7.91
7.90
7.90
7.89
7.89
7.88
7.87
7.87
7.86
7.47
7.47
7.46
7.46
7.45
7.45
7.36
7.35
7.34
7.34
7.33
7.32
7.32
7.30
7.29
7.29
7.28
7.28
7.27
7.26
7.26
5.89
5.88
5.47
5.46
3.93
2.50 DMSO-d6
1H NMR, 400 MHz, DMSO-d6
O
OH
O
D-2
4.03
2.06
2.03
1.05
1.00
1.04
1.00
f1 (ppm)

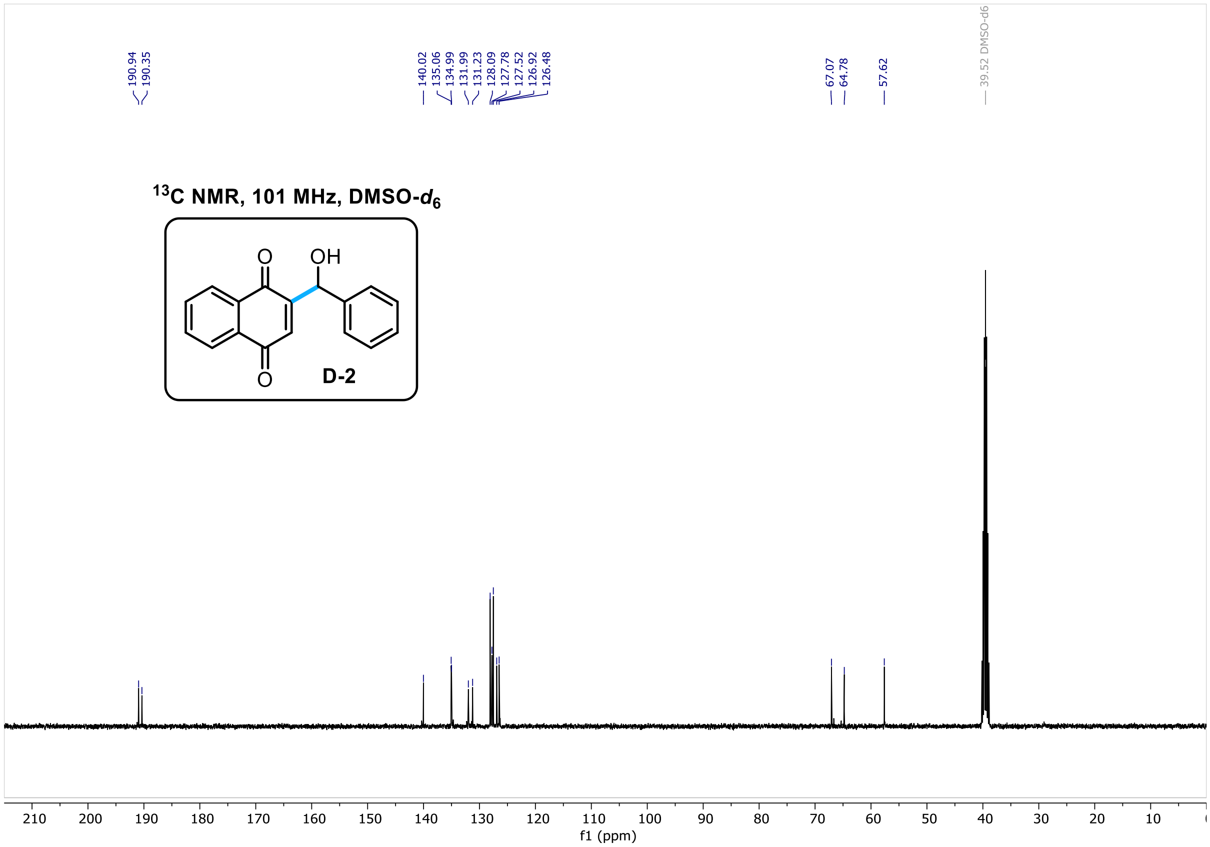
190.94
190.35
140.02
135.06
134.99
131.99
131.23
128.09
127.78
127.52
126.92
126.48
67.07
64.78
57.62
39.52 DMSO-d6
13C NMR, 101 MHz, DMSO-d6
O
OH
O
D-2
f1 (ppm)

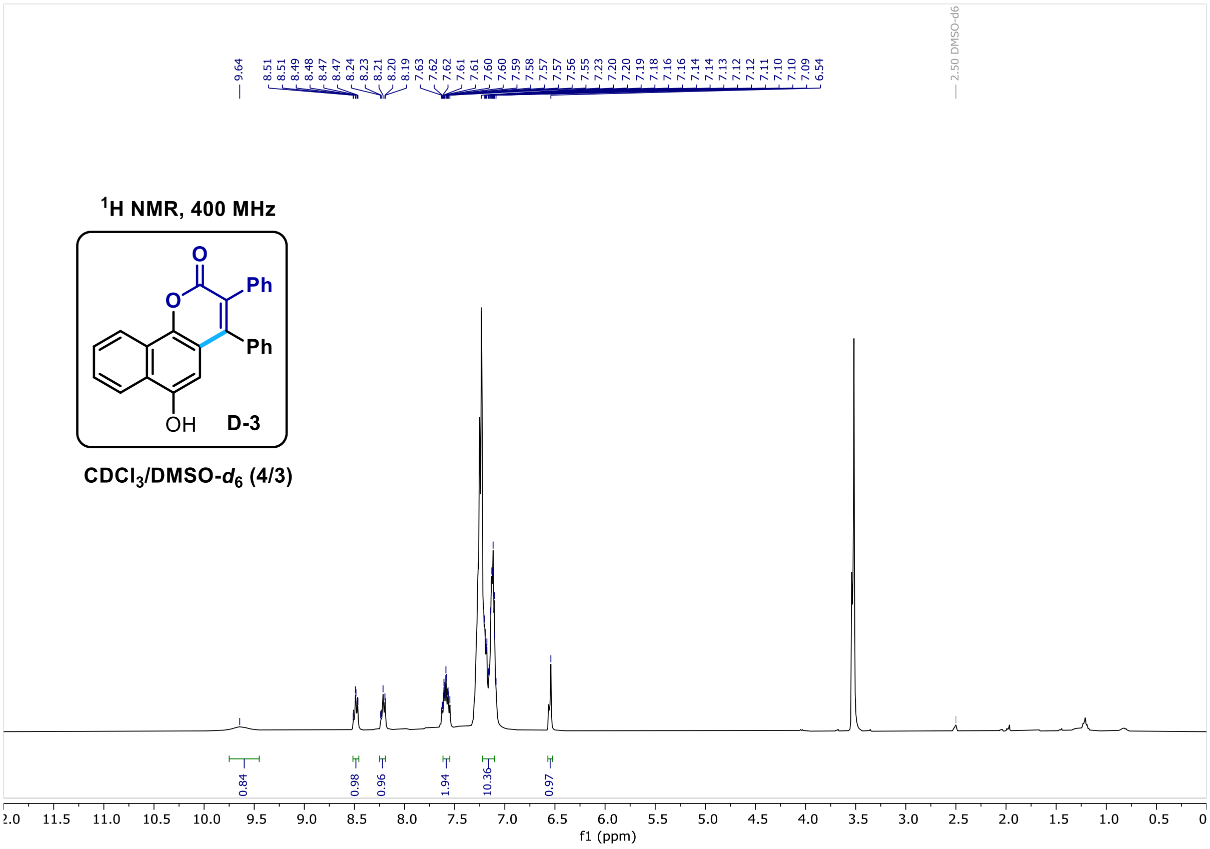
9.64
8.51
8.51
8.49
8.48
8.47
8.47
8.24
8.23
8.21
8.20
8.19
7.63
7.62
7.61
7.61
7.60
7.60
7.59
7.58
7.57
7.57
7.56
7.55
7.23
7.20
7.20
7.19
7.18
7.16
7.16
7.14
7.14
7.13
7.12
7.12
7.11
7.10
7.10
7.09
6.54
2.50 DMSO-d6
1H NMR, 400 MHz
O
O
Ph
Ph
OH
D-3
CDCl3/DMSO-d6 (4/3)
0.84
0.98
0.96
1.94
10.36
0.97
f1 (ppm)

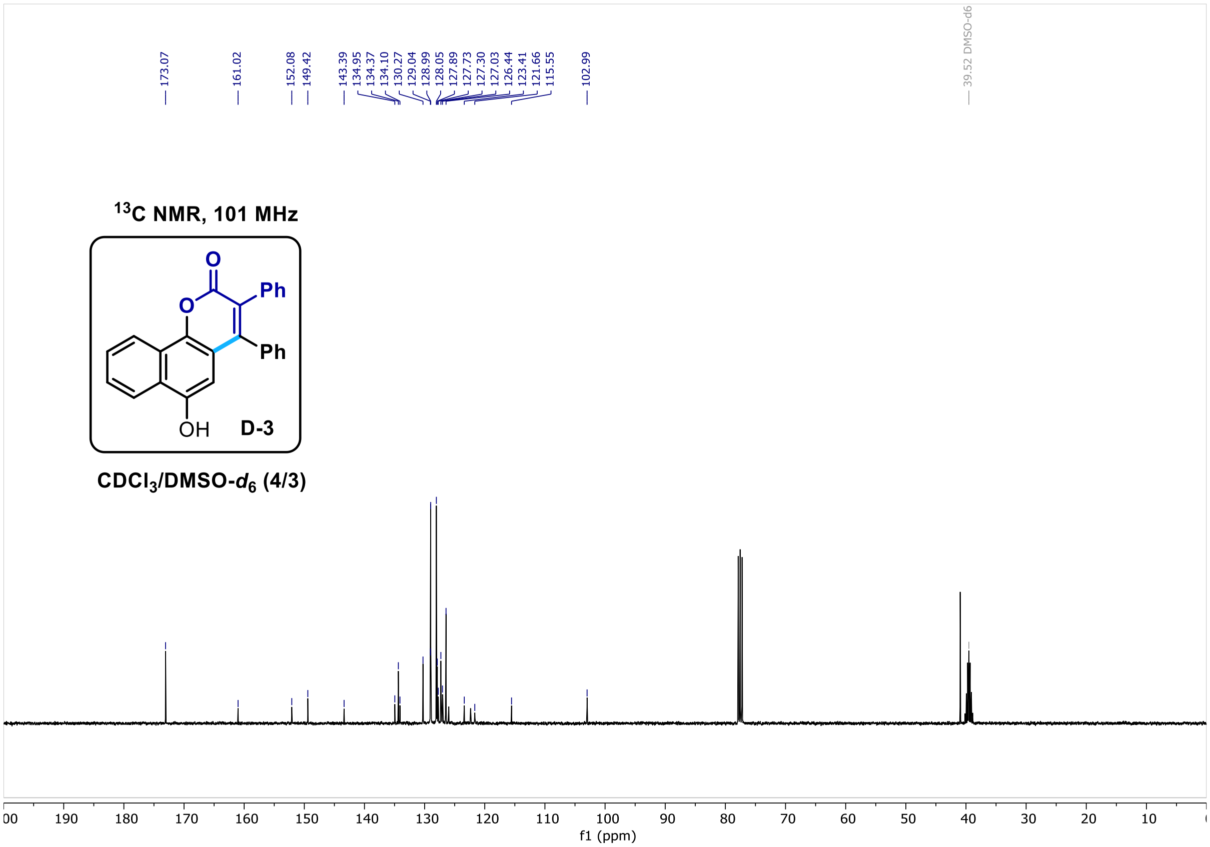
173.07
161.02
152.08
149.42
143.39
134.95
134.37
134.10
130.27
129.04
128.99
128.05
127.89
127.73
127.30
127.03
126.44
123.41
121.66
115.55
102.99
39.52 DMSO-d6
13C NMR, 101 MHz
O
O
Ph
Ph
OH
D-3
CDCl3/DMSO-d6 (4/3)
f1 (ppm)

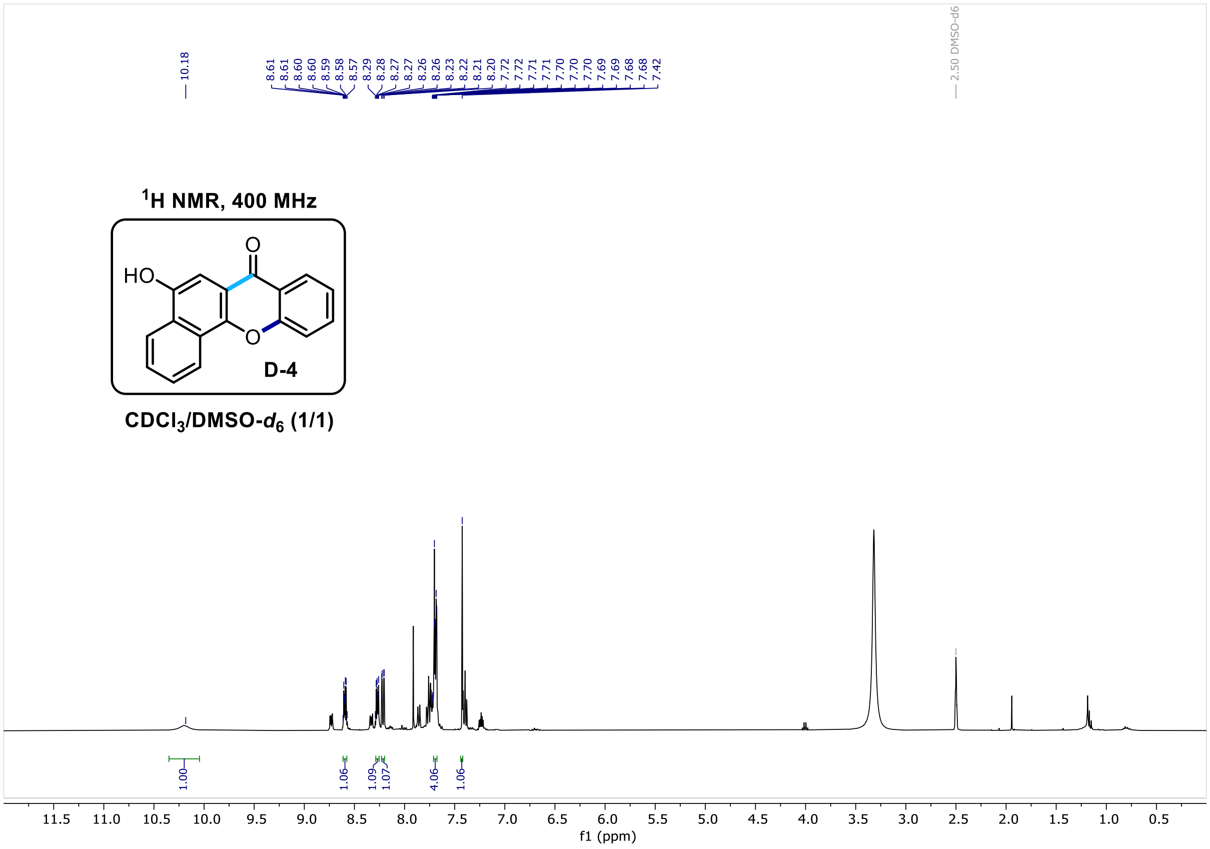
10.18
8.61 8.61 8.60 8.60 8.59 8.58 8.57 8.29 8.28 8.27 8.27 8.26 8.26 8.23 8.22 8.21 8.20 7.72 7.72 7.71 7.71 7.70 7.70 7.69 7.69 7.68 7.68 7.42
2.50 DMSO-d6
1H NMR, 400 MHz
HO
O
O
D-4
CDCl3/DMSO-d6 (1/1)
1.00 1.06 1.09 1.07 4.06 1.06
f1 (ppm)

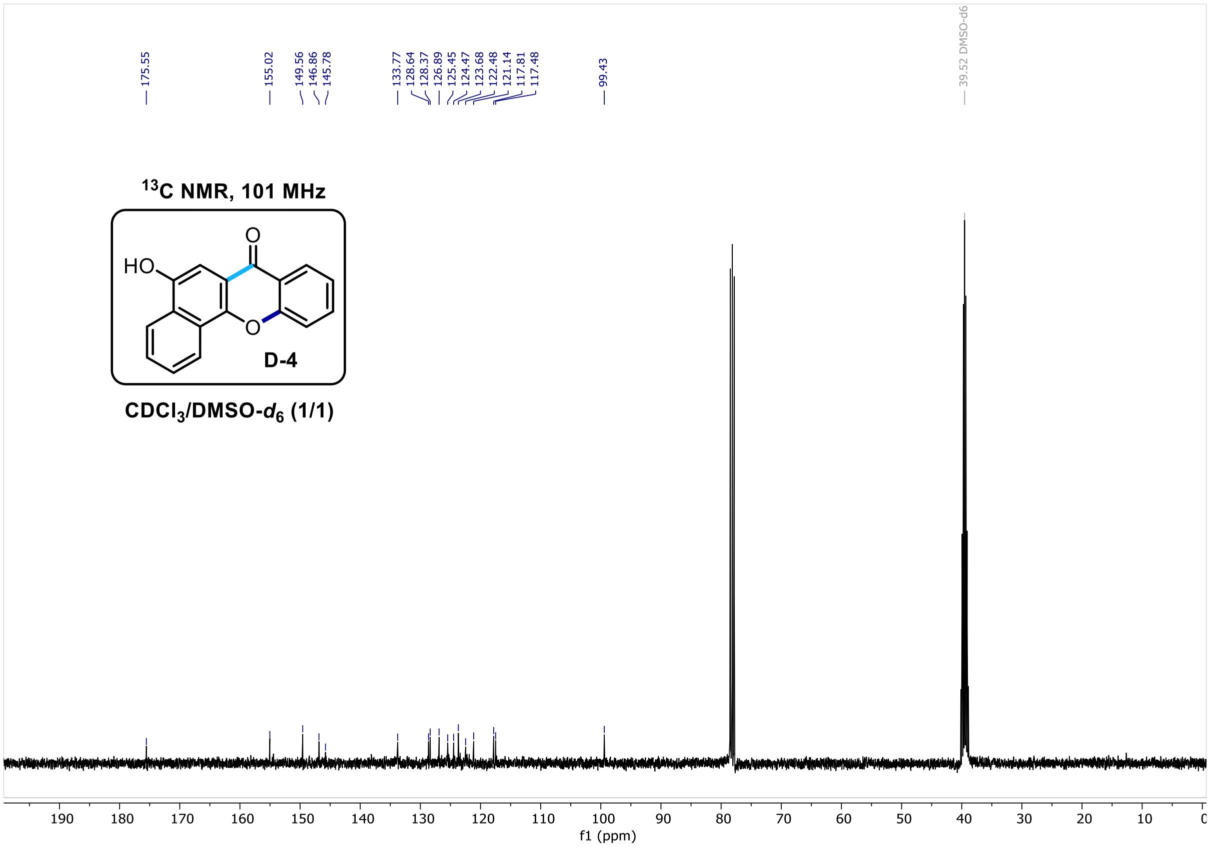
175.55
155.02 149.56 146.86 145.78
133.77 128.64 128.37 126.89 125.45 124.47 123.68 122.48 121.14 117.81 117.48
99.43
39.52 DMSO-d6
13C NMR, 101 MHz
HO
O
O
D-4
CDCl3/DMSO-d6 (1/1)
f1 (ppm)

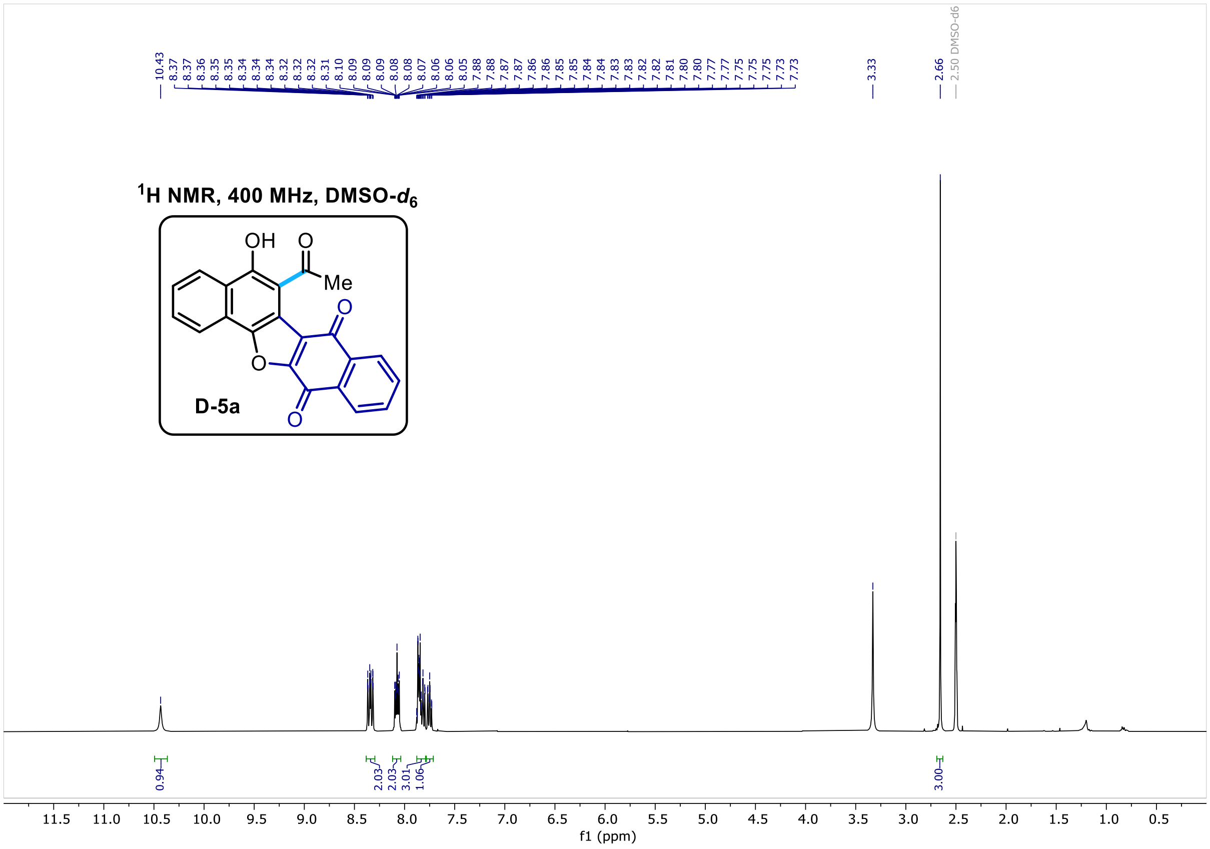
1H NMR, 400 MHz, DMSO-d6
D-5a
OH
O
Me
O
O
O
f1 (ppm)

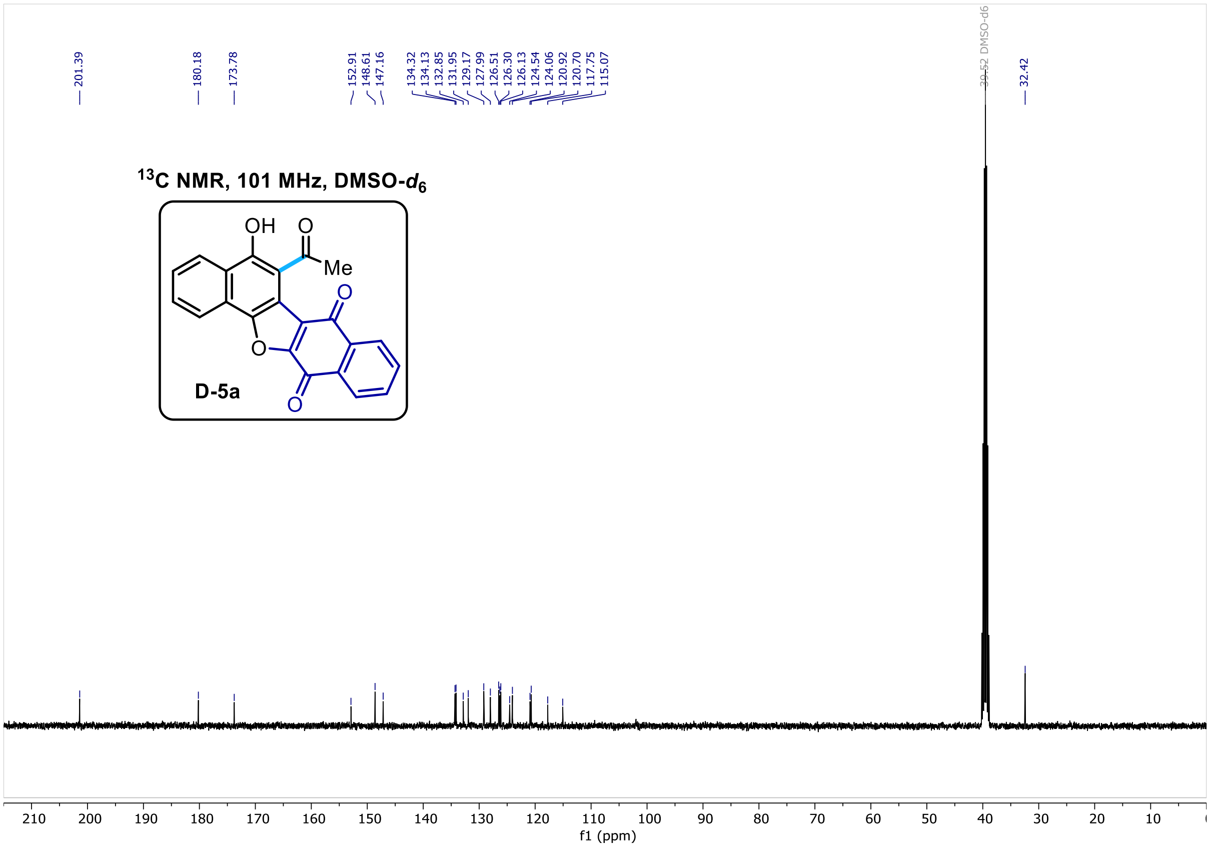
13C NMR, 101 MHz, DMSO-d6
D-5a
OH
O
Me
O
O
O
f1 (ppm)

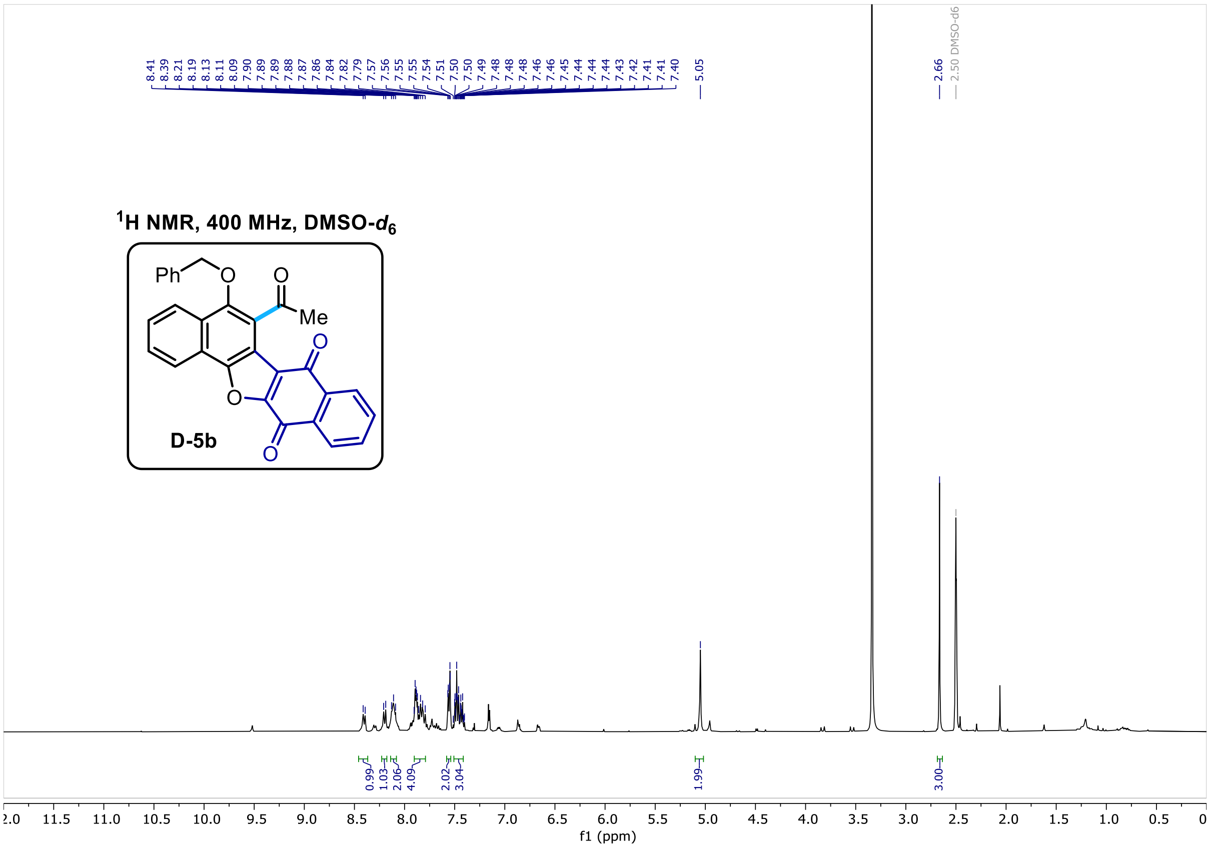
1H NMR, 400 MHz, DMSO-d6
D-5b
Ph
O
O
Me
O
O
O
8.41 8.39 8.21 8.19 8.13 8.11 8.09 7.90 7.89 7.89 7.88 7.87 7.86 7.84 7.82 7.79 7.57 7.56 7.55 7.55 7.54 7.51 7.50 7.50 7.49 7.48 7.48 7.48 7.46 7.45 7.44 7.44 7.44 7.43 7.42 7.41 7.41 7.40
5.05
2.66
2.50 DMSO-d6
0.99 1.03 2.06 4.09 2.02 3.04 1.99 3.00
f1 (ppm)

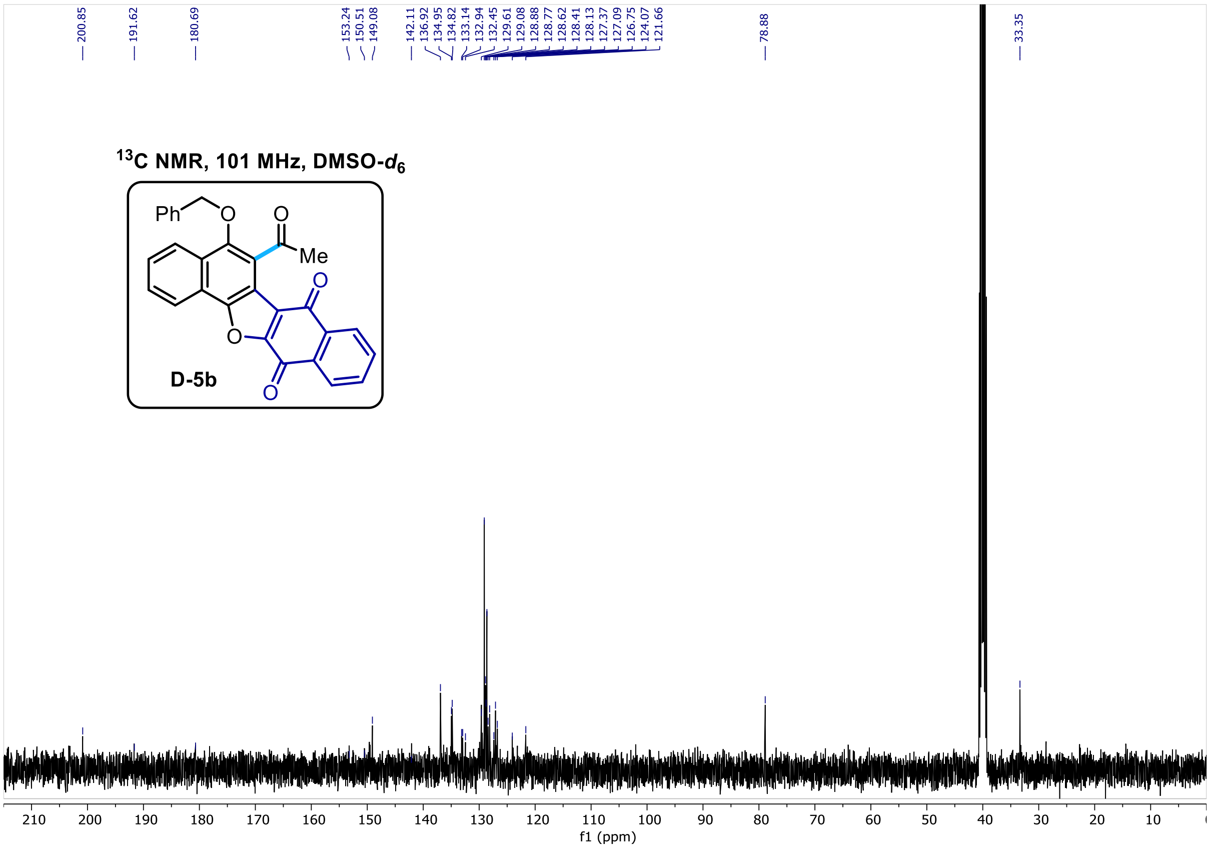
13C NMR, 101 MHz, DMSO-d6
D-5b
Ph
O
O
Me
O
O
O
200.85
191.62
180.69
153.24 150.51 149.08
142.11 136.92 134.95 134.82 133.14 132.94 132.45 129.61 129.08 128.88 128.77 128.62 128.41 128.13 127.37 127.09 126.75 124.07 121.66
78.88
33.35
f1 (ppm)

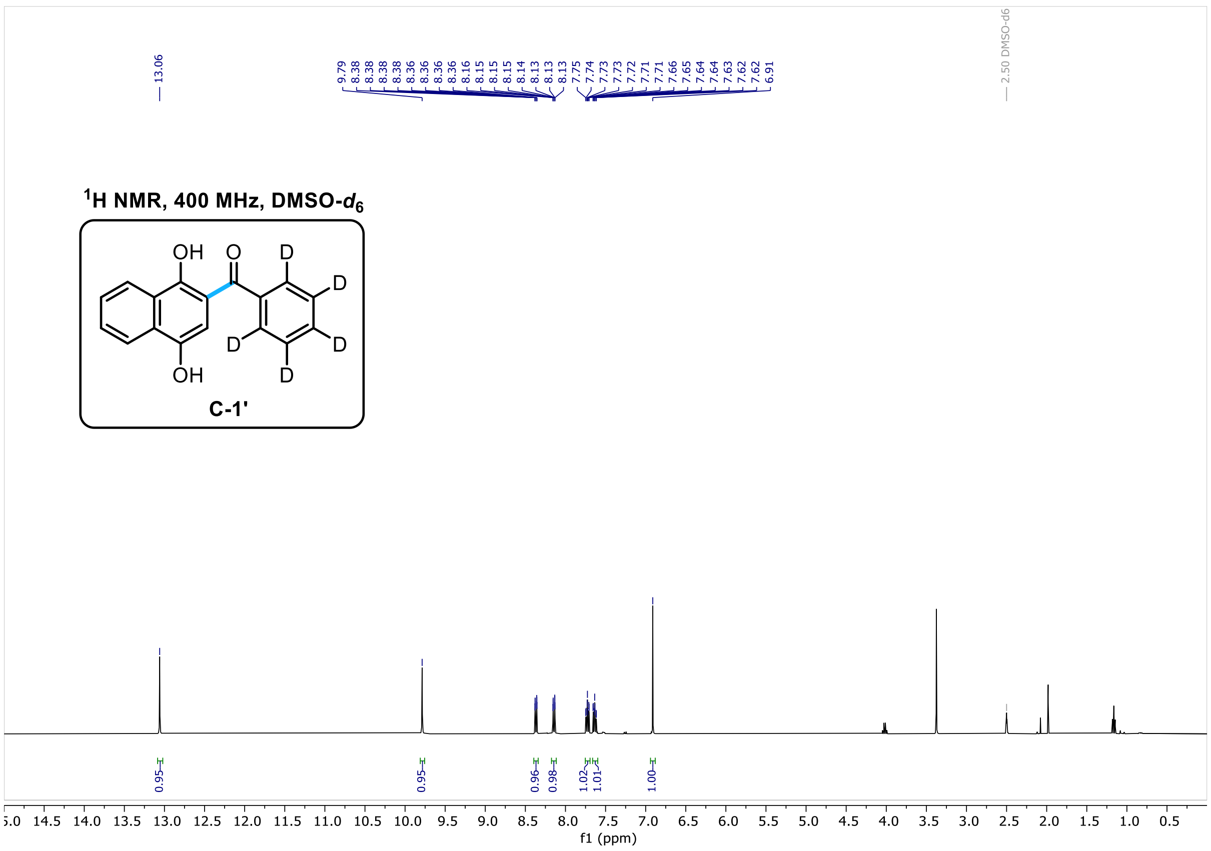

13.06
9.79
8.38
8.38
8.38
8.38
8.36
8.36
8.36
8.16
8.15
8.15
8.15
8.14
8.13
8.13
8.13
7.75
7.74
7.73
7.73
7.72
7.71
7.71
7.66
7.65
7.64
7.64
7.63
7.62
7.62
6.91
2.50 DMSO-d6
^{1}H NMR, 400 MHz, DMSO-d_6
OH
O
D
D
D
D
D
OH
C-1'
0.95
0.95
0.96
0.98
1.02
1.01
1.00
f1 (ppm)

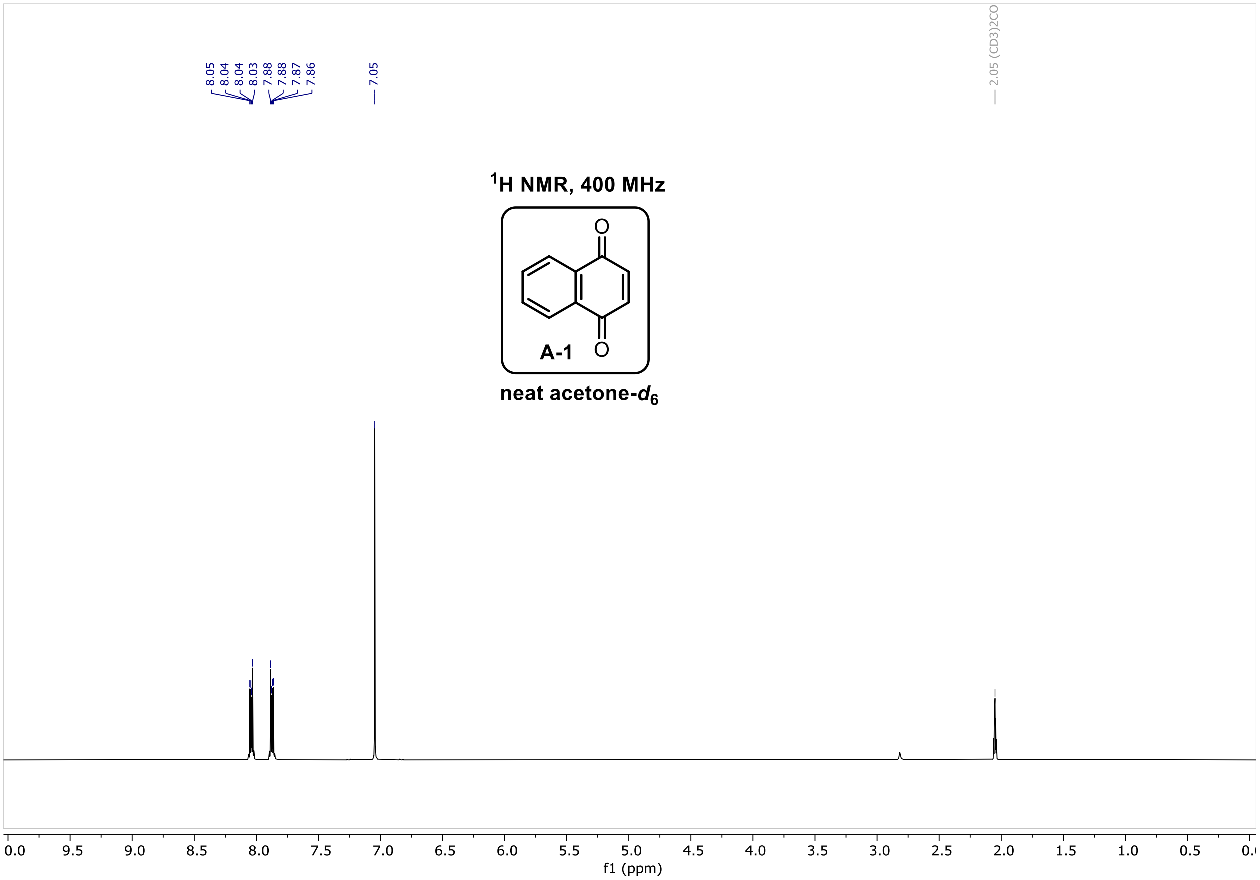
8.05
8.04
8.04
8.03
7.88
7.88
7.87
7.86
7.05
2.05 (CD3)2CO
^{1}H NMR, 400 MHz
A-1
neat acetone-d_6
0.0 9.5 9.0 8.5 8.0 7.5 7.0 6.5 6.0 5.5 5.0 4.5 4.0 3.5 3.0 2.5 2.0 1.5 1.0 0.5
f1 (ppm)

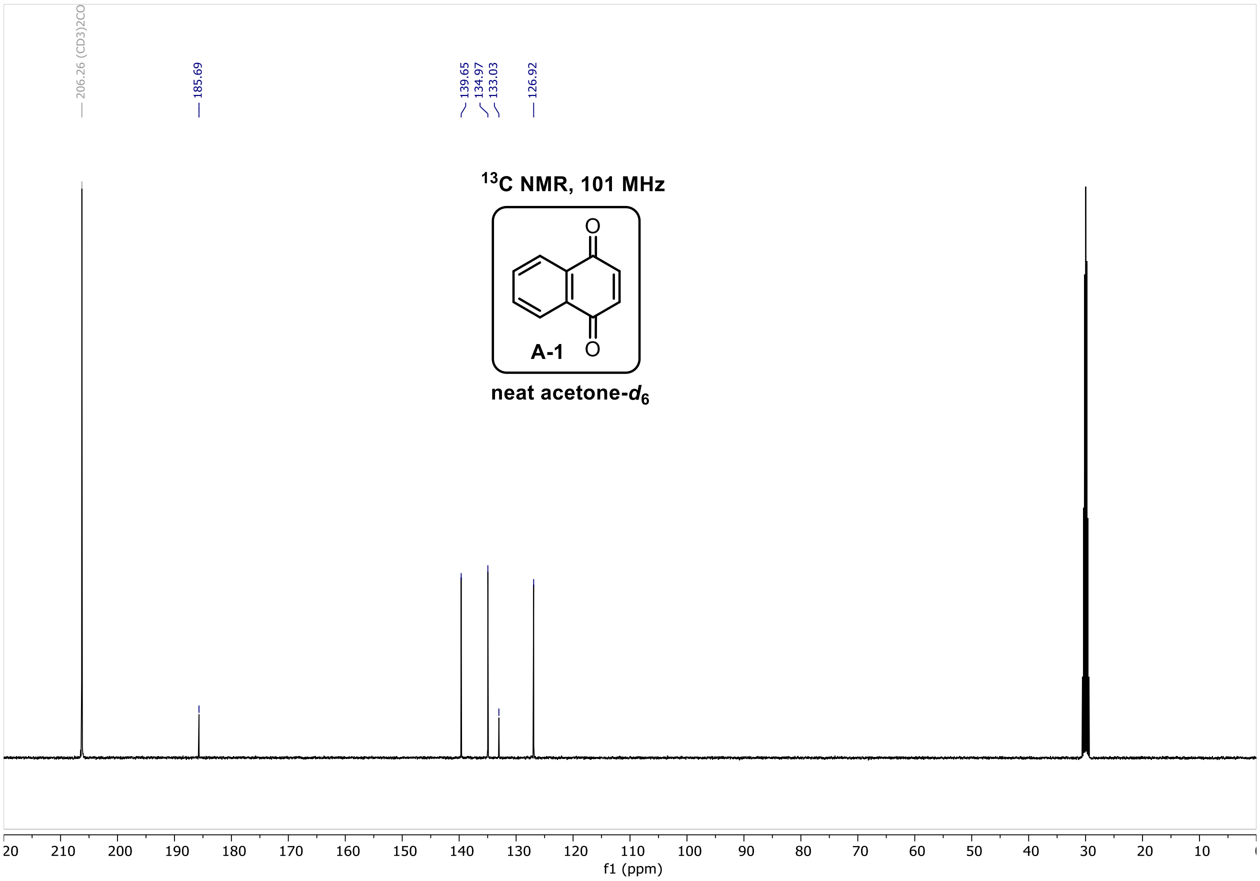
206.26 (CD3)2CO
185.69
139.65
134.97
133.03
126.92
^{13}C NMR, 101 MHz
A-1
neat acetone-d_6
210 200 190 180 170 160 150 140 130 120 110 100 90 80 70 60 50 40 30 20 10
f1 (ppm)

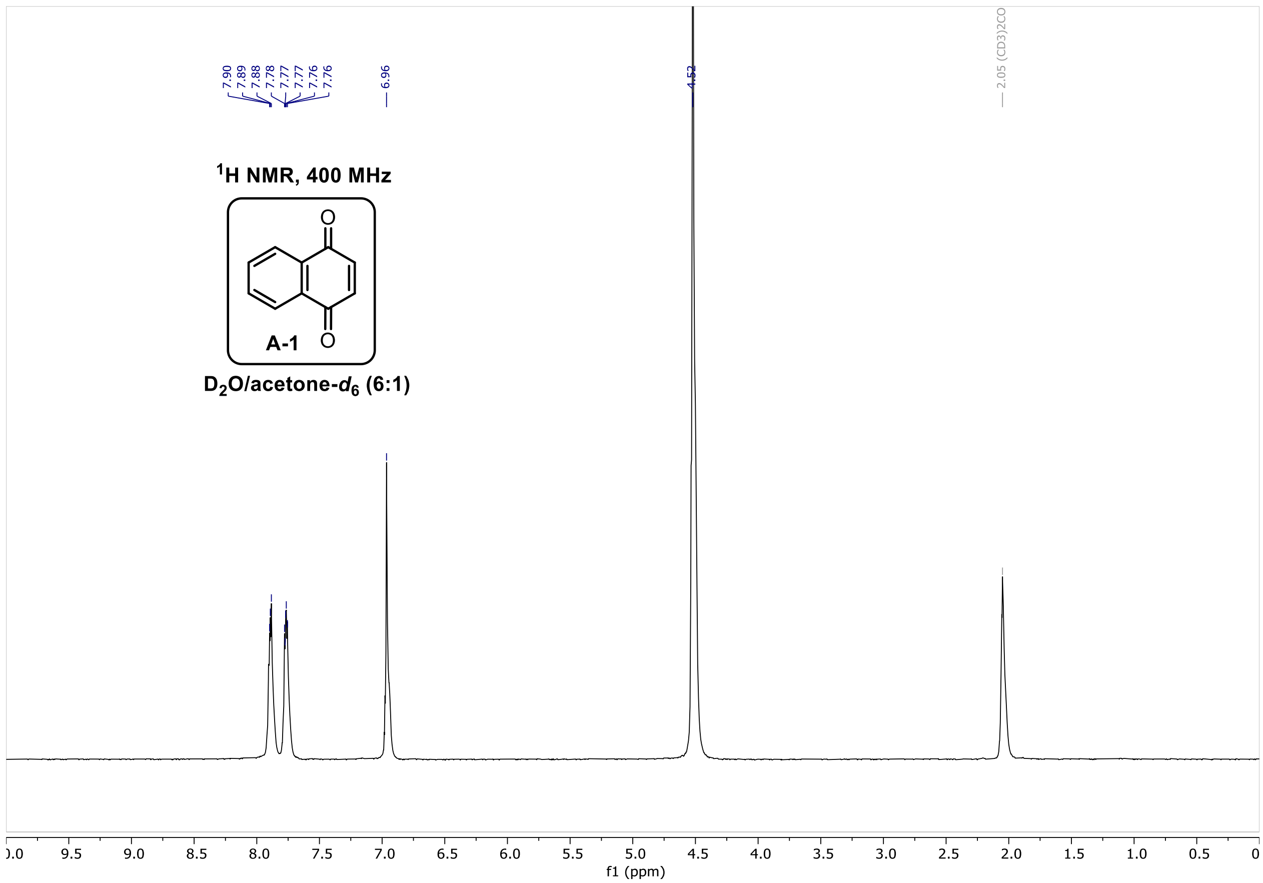
7.90
7.89
7.88
7.78
7.77
7.77
7.76
7.76
6.96
4.52
2.05 (CD3)2CO
1H NMR, 400 MHz
A-1
D2O/acetone-d6 (6:1)
f1 (ppm)

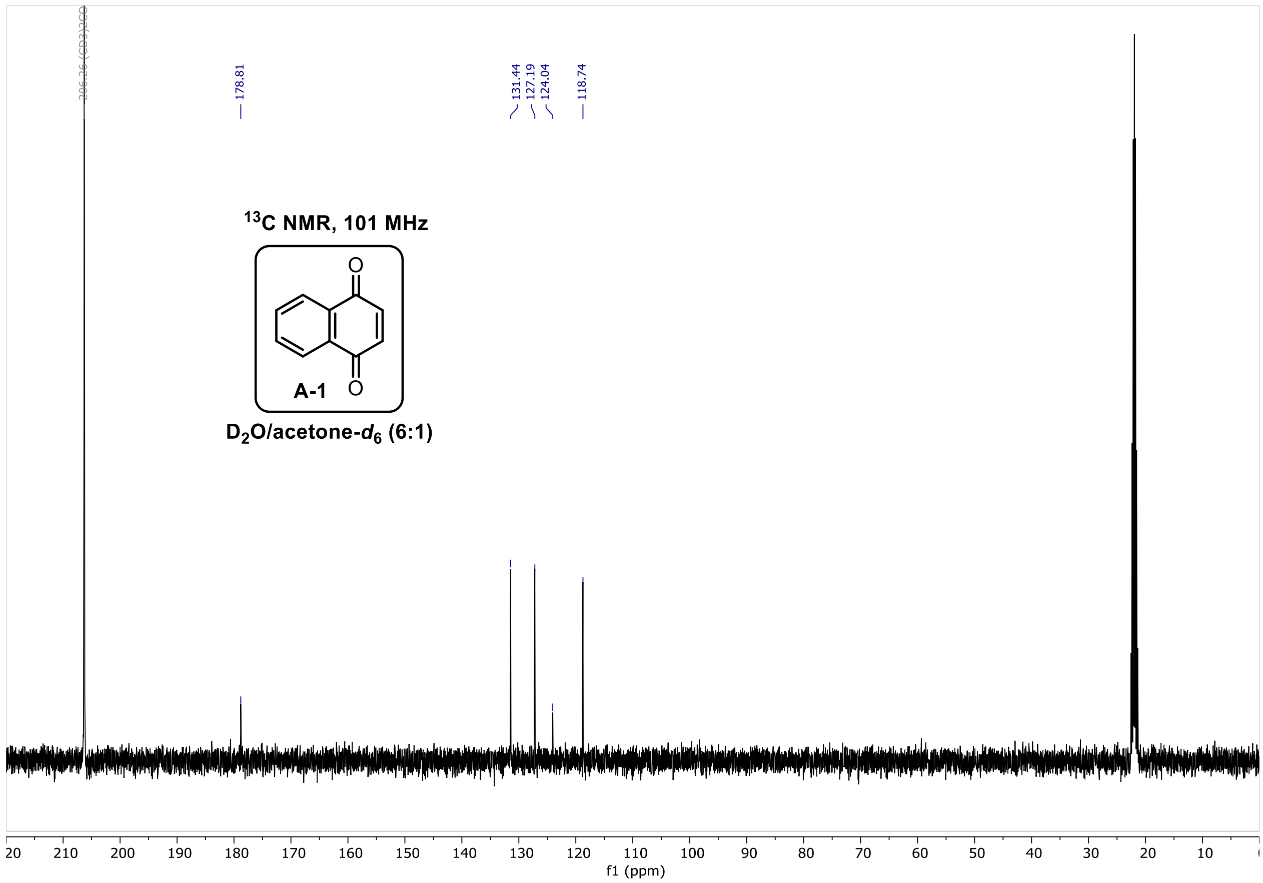
206.26 (CD3)2CO
178.81
131.44
127.19
124.04
118.74
13C NMR, 101 MHz
A-1
D2O/acetone-d6 (6:1)
f1 (ppm)

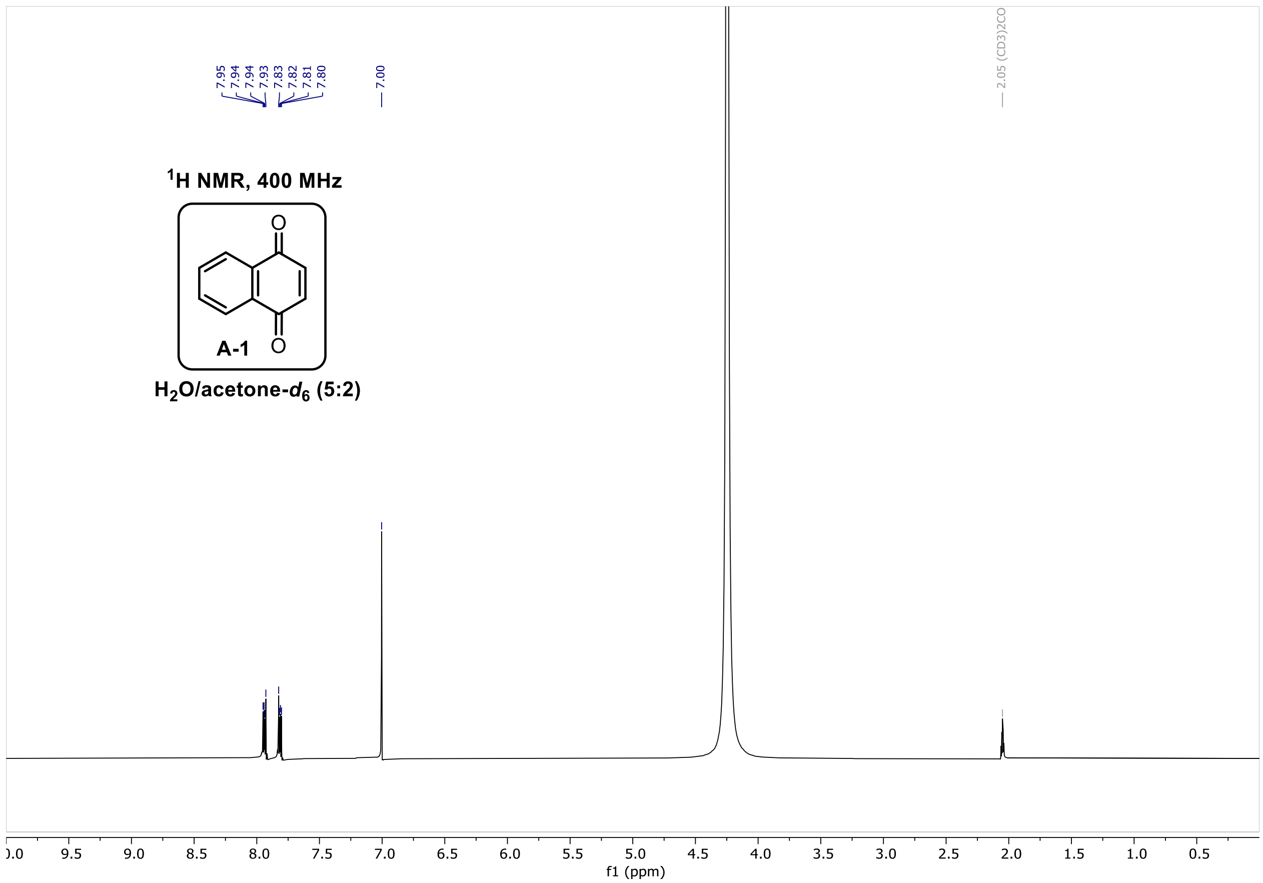

7.95
7.94
7.94
7.93
7.83
7.82
7.81
7.80
7.00
2.05 (CD3)2CO
1H NMR, 400 MHz
A-1
H2O/acetone-d6 (5:2)
f1 (ppm)

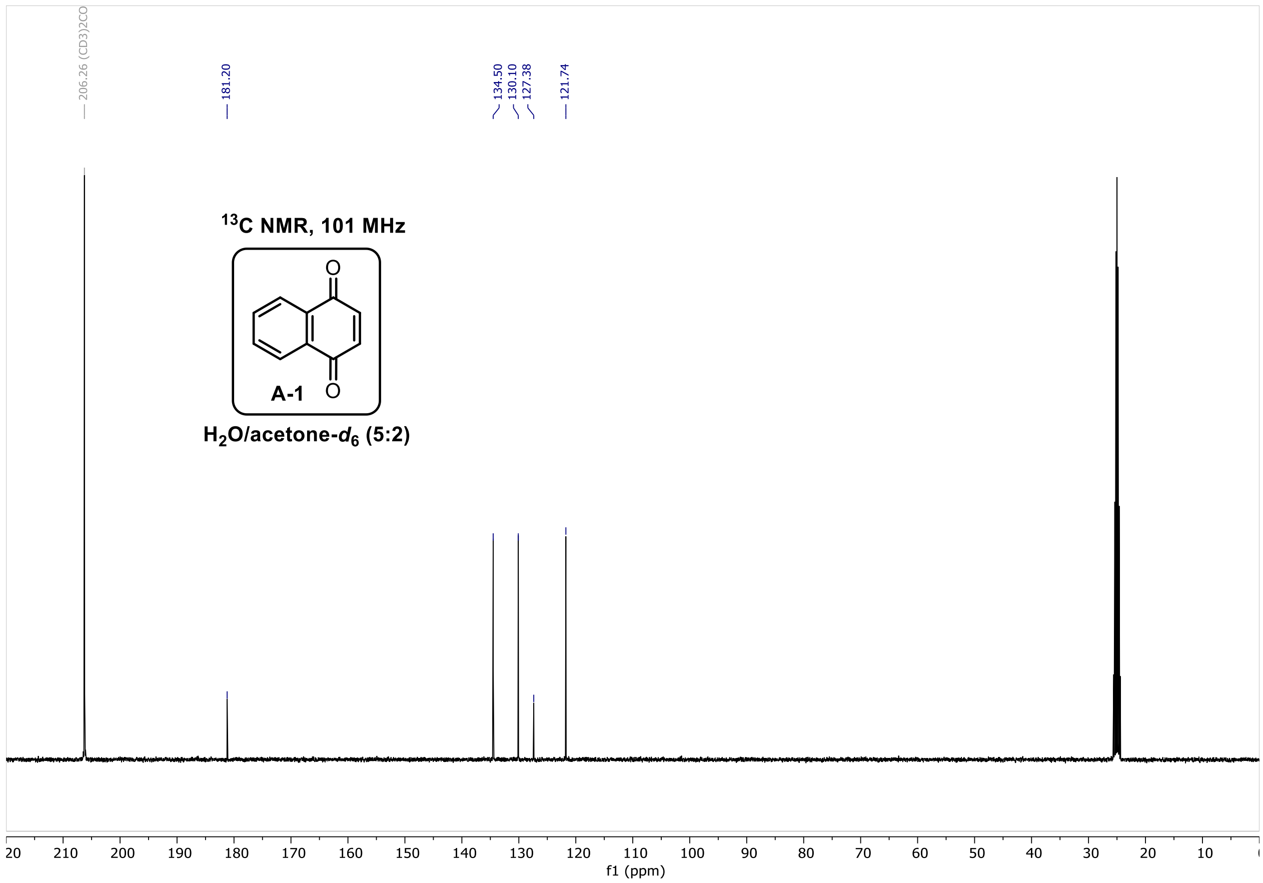

206.26 (CD3)2CO
181.20
134.50
130.10
127.38
121.74
13C NMR, 101 MHz
A-1
H2O/acetone-d6 (5:2)
f1 (ppm)

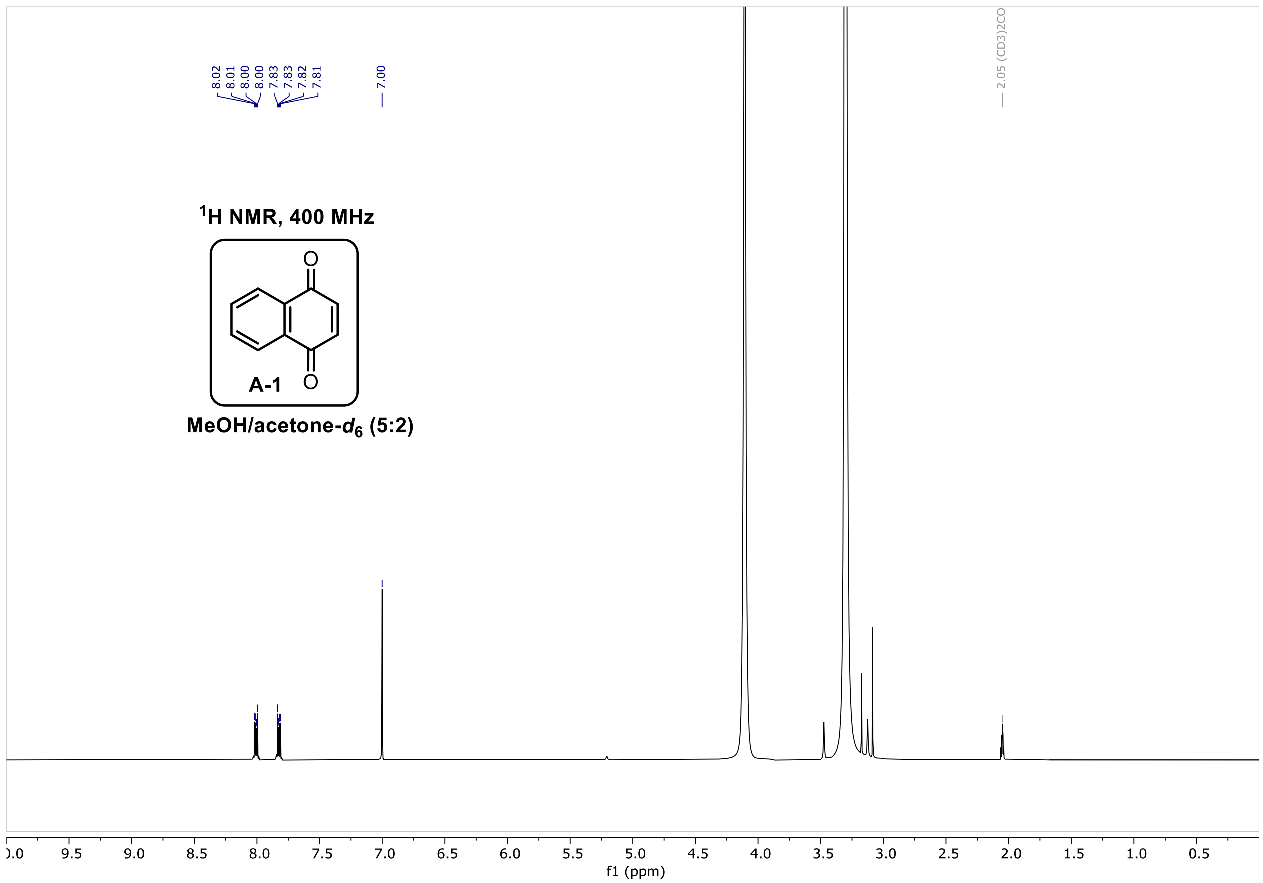

8.02
8.01
8.00
8.00
7.83
7.83
7.82
7.81
7.00
2.05 (CD3)2CO
1H NMR, 400 MHz
A-1
MeOH/acetone-d6 (5:2)
f1 (ppm)

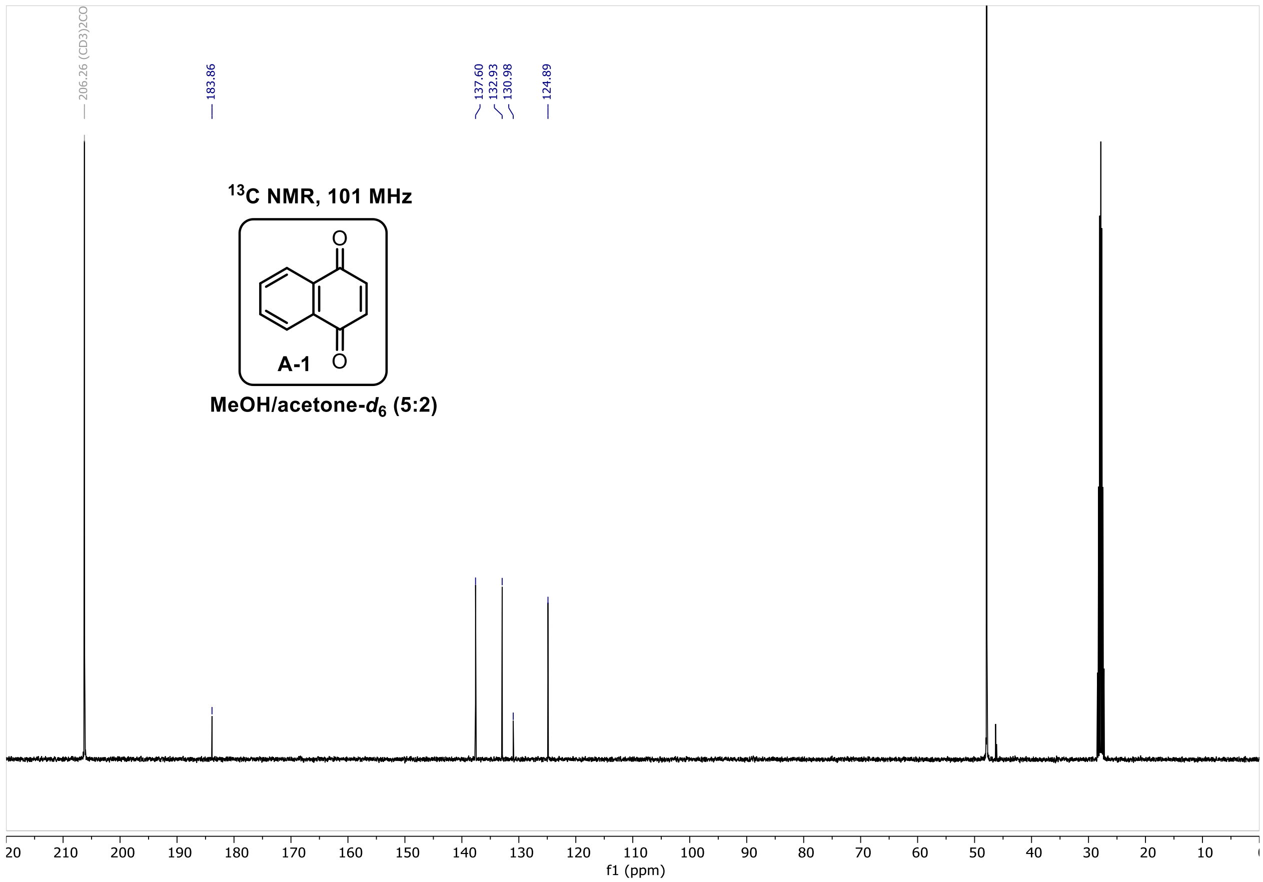

206.26 (CD3)2CO
183.86
137.60
132.93
130.98
124.89
13C NMR, 101 MHz
A-1
MeOH/acetone-d6 (5:2)
f1 (ppm)

11. References:

1 Das, K., Saha, N., Li, Z., Ghosh, I. & Konig, B. Generalizing Vinyl Halide Cross-Coupling Reactions with Photoredox and Photoredox/Nickel Dual Catalysis. *Angew. Chem. Int. Ed.* **64**, e202510715, doi:10.1002/anie.202510715 (2025).

2 Mandal, T., Mallick, S., Kumari, N. & De Sarkar, S. Visible-Light-Mediated Synthesis of Phenanthrenes through Successive Photosensitization and Photoredox by a Single Organocatalyst. *Org. Lett.* **24**, 8452-8457, doi:10.1021/acs.orglett.2c03612 (2022).

3 Mitchell, L. J., Lewis, W. & Moody, C. J. Solar photochemistry: optimisation of the photo Friedel–Crafts acylation of naphthoquinones. *Green Chem.* **15**, 2830, doi:10.1039/c3gc41477a (2013).

4 Uchikura, T. *et al.* Radical Hydroalkylation and Hydroacylation of Alkenes by the Use of Benzothiazoline under Thermal Conditions. *J. Org. Chem.* **85**, 12715-12723, doi:10.1021/acs.joc.0c01872 (2020).

5 Iribarra, J. *et al.* Synthesis and antitumor evaluation of 6-aryl-substituted benzo[j]phenanthridine- and benzo[g]pyrimido[4,5-c]isoquinolinequinones. *Molecules* **17**, 11616-11629, doi:10.3390/molecules171011616 (2012).

6 Jha, R. K. *et al.* Light-Driven Carbon-Carbon Coupling of alpha-sp(3)-CH of Aliphatic Alcohols with sp(2)-CH Bond of 1,4-Naphthoquinones. *Org. Lett.* **24**, 7605-7610, doi:10.1021/acs.orglett.2c03066 (2022).

7 Ma, B. *et al.* Synthesis of Acylhydroquinones through Visible-Light-Mediated Hydroacylation of Quinones with alpha-Keto Acids. *J. Org. Chem.* **89**, 1669-1680, doi:10.1021/acs.joc.3c02361 (2024).

8 Oelgemöller, M., Schiel, C., Fröhlich, R. & Mattay, J. The "Photo-Friedel−Crafts Acylation" of 1,4-Naphthoquinones. *Eur. J. Org. Chem.* **2002**, 2465, doi:10.1002/1099-0690(200208)2002:15<2465::aid-ejoc2465>3.0.co;2-o (2002).

9 Lin, Z. Y., Chen, Y. L., Lee, C. S. & Chuang, C. P. Metal Salt Mediated Radical Reactions of 2-Substituted-1,4-Naphthoquinones. *Eur. J. Org. Chem.* **2010**, 3876-3882, doi:10.1002/ejoc.201000272 (2010).

10 Dam, J., Bode, M. L. & de Koning, C. B. Ceric Ammonium Sulfate (CAS) Mediated Oxidations of Benzophenones Possessing a Phenolic Substituent for the Synthesis of Xanthones and Related Products. *J. Org. Chem.* **84**, 150-160, doi:10.1021/acs.joc.8b02503 (2019).

11 Padwal, J., Lewis, W. & Moody, C. J. Synthesis of balsaminone A, a naturally occurring pentacyclic dinaphthofuran quinone. *J. Org. Chem.* **76**, 8082-8087, doi:10.1021/jo201395n (2011).

12 Plenert, A. C., Mendez-Vega, E. & Sander, W. Micro- vs Macrosolvation in Reichardt's Dyes. *J. Am. Chem. Soc.* **143**, 13156-13166, doi:10.1021/jacs.1c04680 (2021).

13 Santana-García, R., Aviña-Verduzco, J., Herrera-Bucio, R. & Navarro-Santos, P. S_EAr Mechanism of the Products of 1,2-Dimethoxybenzene and a Captodative Olefin: A Theoretical Approach. *Computational Chemistry* **12**, 57-74, doi:10.4236/cc.2024.123003 (2024).

14 Chakraborty, M. & Panda, A. K. Spectral behaviour of eosin Y in different solvents and aqueous surfactant media. *Spectrochim Acta A Mol Biomol Spectrosc* **81**, 458-465, doi:10.1016/j.saa.2011.06.038 (2011).

15 Rittner, T. *et al.*, doi:10.26434/chemrxiv-2025-hx8h0 (2025).

16 Rodriguez, H. B., San Roman, E., Duarte, P., Machado, I. F. & Ferreira, L. F. Eosin Y triplet state as a probe of spatial heterogeneity in microcrystalline cellulose. *Photochem Photobiol* **88**, 831-839, doi:10.1111/j.1751-1097.2012.01152.x (2012).

17 Zhou, W., Gu, K., Schultz, F. & Liu, C. Solvent Effect on the Behavior of Single Eosin Y Photoredox Catalyst. *Chem Biomed Imaging* **3**, 599-604, doi:10.1021/cbmi.5c00008 (2025).
